# Factors Governing Reactivity and Selectivity in Hydrogen Atom Transfer from C(sp^3^)–H Bonds of Nitrogen-Containing Heterocycles to the Cumyloxyl Radical

**DOI:** 10.1021/acs.joc.2c00955

**Published:** 2022-05-24

**Authors:** Marco Galeotti, Chiara Trasatti, Sergio Sisti, Michela Salamone, Massimo Bietti

**Affiliations:** Dipartimento di Scienze e Tecnologie Chimiche, Università“Tor Vergata”, Via Della Ricerca Scientifica, 1, Rome I-00133, Italy

## Abstract

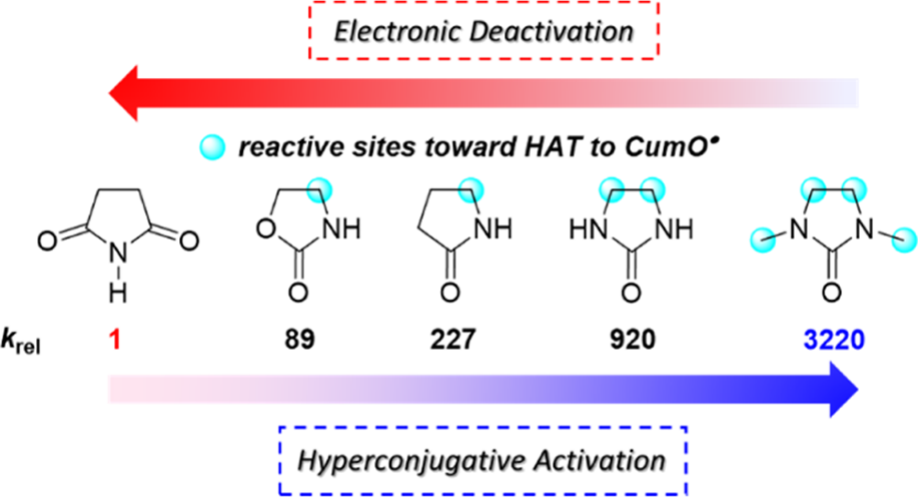

A kinetic study of the hydrogen atom transfer (HAT) reactions from nitrogen-containing heterocycles (secondary and tertiary lactams, 2-imidazolidinones, 2-oxazolidinones, and succinimides) to the cumyloxyl radical has been carried out employing laser flash photolysis with ns time resolution. HAT occurs from the C–H bonds that are α to nitrogen, activated by hyperconjugative overlap with the N–C=O π system. In the lactam series, the second-order HAT rate constant (*k*_H_) was observed to decrease by a factor of ∼4 going from the five- and six-membered ring derivatives to the eight-membered ones, a behavior that was rationalized on the basis of a reduced extent of hyperconjugative activation associated to the greater flexibility of the larger rings compared to the smaller ones. In the five-membered-ring substrate series, the *k*_H_ values were observed to increase by >3 orders of magnitude on going from succinimide to 2-imidazolidinones, a behavior that was explained in terms of the divergent contribution of hyperconjugative activation and deactivating electronic effects determined by ring functionalities. The results are discussed in the framework of the development of HAT-based C–H bond functionalization procedures.

## Introduction

**Figure 1:**
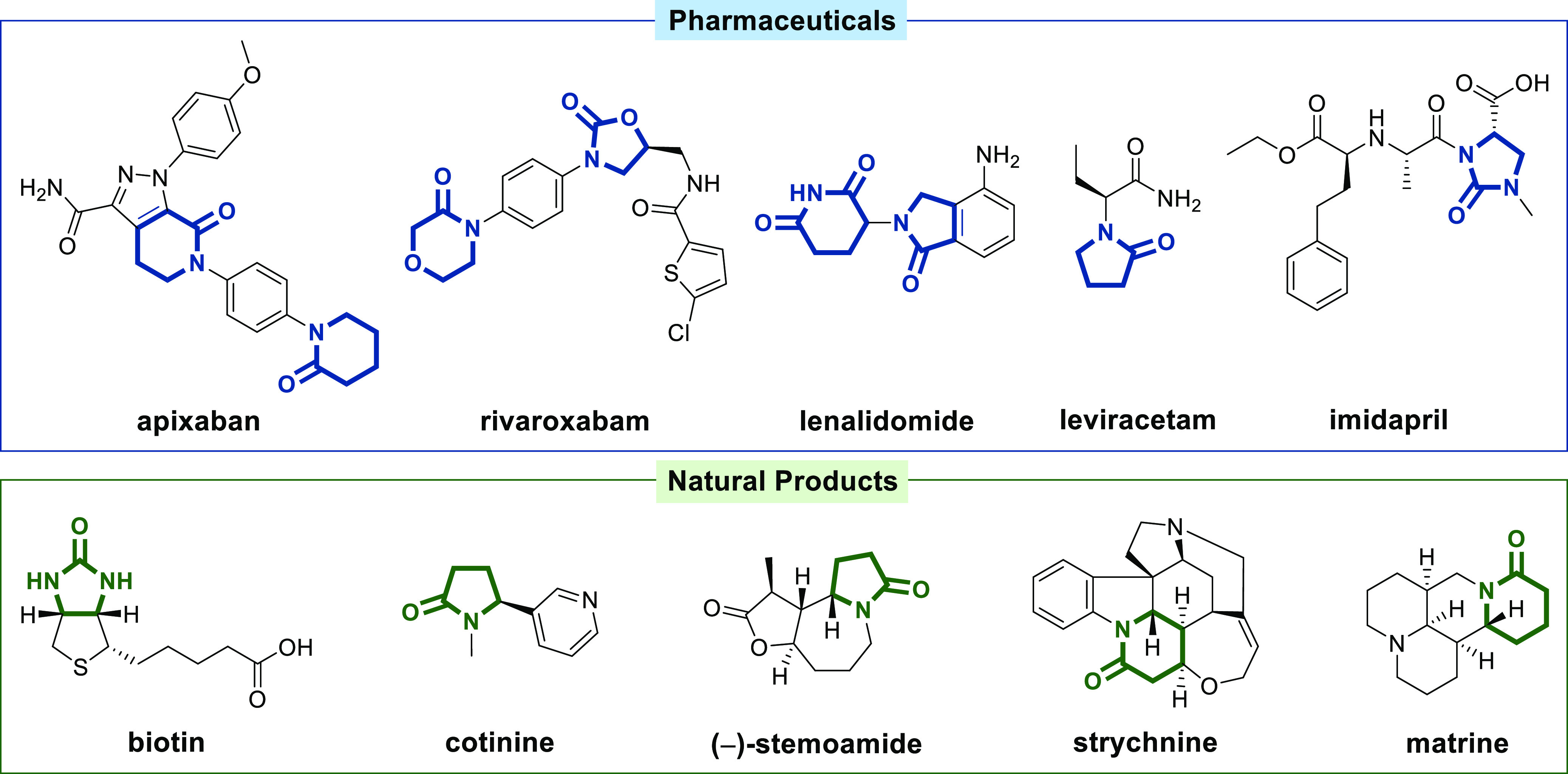
Examples of pharmaceuticals and natural products bearing nitrogen-containing heterocyclic structural motifs.

Lactams and other nitrogen-containing heterocyclic compounds such as imidazolidinones, oxazolidinones, and imides are key structural motifs that are found in a large number of natural products and pharmaceuticals, selected examples of which are displayed in Figure 1.

The anticoagulants apixaban and rivaroxabam and the oncologic drug lenalidomide, displaying γ- and δ-lactam, 2-oxazolidinone, and glutarimide structures, are ranked as number 1, 4, and 2, respectively, among the top 200 small-molecule pharmaceuticals in terms of retail sales for the year 2020.^1^ The anticonvulsant drug leviracetam displays a γ-lactam motif. The antihypertensive drug imidapril displays a 2-imidazolidinone ring, a structural feature that is also found in vitamin B7 (biotin). The tobacco alkaloid cotinine and stemoamide-type alkaloids contain a γ-lactam ring, while the alkaloids strychnine and matrine bear a δ-lactam ring embedded into the polycyclic framework.

Because of the importance of these structural motifs, divergent synthetic approaches can offer the opportunity to access novel or modified structures of pharmaceutical interest to be studied for improved potency or new activities. In this context, several intermolecular and intramolecular procedures for the synthesis of nitrogen-containing heterocyclic compounds from readily available precursors have been reported.^2^

An alternative approach that can provide straightforward access to new derivatives is represented by late-stage C(sp^3^)–H bond functionalization of preexisting substrates, currently a mainstream topic of modern synthetic chemistry.^3^ This approach offers the opportunity to diversify complex structures through the direct introduction of functional groups in place of H without resorting to *de novo* synthesis, providing significant advantages both in terms of atom and step economy.^4^ In the framework of late-stage C(sp^3^)–H bond functionalization of nitrogen-containing heterocycles, two recent studies reported on hydrogen atom transfer (HAT)-initiated C–H bond methylation promoted by the Mn(CF_3_PDP)/H_2_O_2_^5^ system or by *tert*-alkoxyl radicals in combination with Ni catalysts.^6^ These methods were applied to the functionalization of a variety of natural products and pharmaceuticals. In both studies, functionalization typically occurred at activated sites (benzylic and C–H bonds that are α to nitrogen).

**Chart 1:**
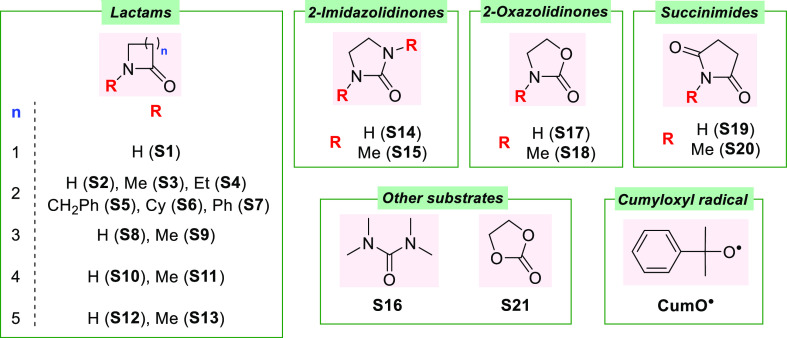
Structure of the Cumyloxyl Radical (CumO^•^) and of the Substrates Employed in this Study

On the basis of these results and in keeping with the importance of nitrogen-containing heterocyclic structures in natural products and pharmaceuticals, we reasoned that a deeper understanding of the reactivities and site-selectivities observed in HAT from the C–H bonds of these structures would provide useful information that could allow to better exploit the potential of HAT-based late-stage C(sp^3^)–H functionalization strategies. Along this line, by taking into account the current knowledge on the factors that govern reactivity and site-selectivity in HAT-based C–H bond functionalizations^4c,4f,7^ and in order to obtain new information on the role of substrate structures in these reactions, herein we report the results of a detailed kinetic study on the reactions of the cumyloxyl radical (PhC(CH_3_)_2_O^•^, CumO^•^) with an extended series of lactams (**S1–S13**) and with 2-imidazolidinones (**S14** and **S15**), 2-oxazolidinones (**S17** and **S18**), and succinimides (**S19** and **S20**), the structures for which are displayed in Chart 1. As a matter of comparison, the reaction of CumO^•^ with tetramethylurea (**S16**) and with an oxygen-containing heterocyclic compound such as ethylene carbonate (**S21**) was also investigated.

## Results and Discussion

**Figure 2:**
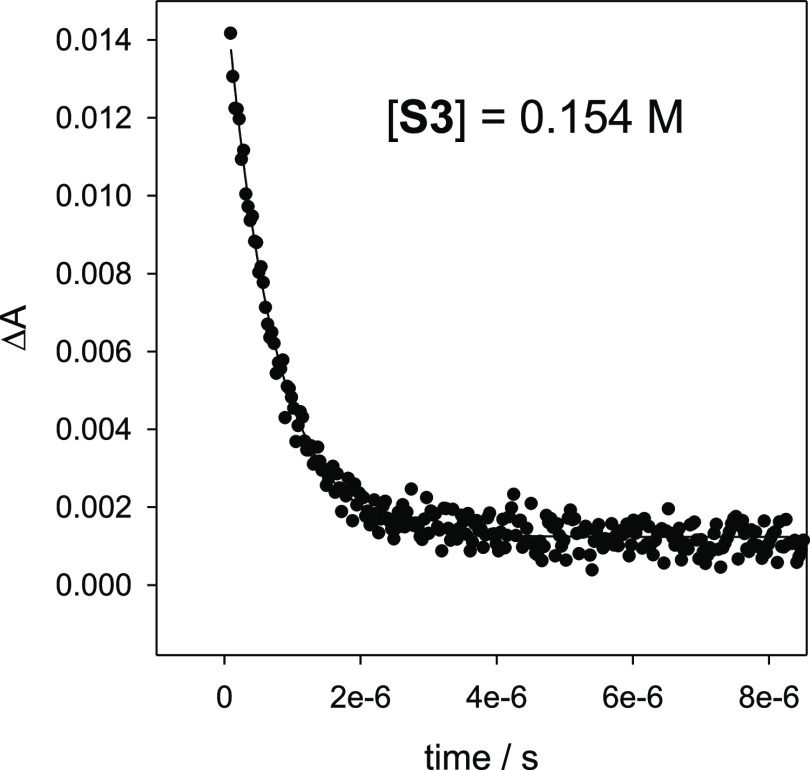
Plot of the change in absorbance (Δ*A*) monitored at 490 nm *vs* time for the reaction of CumO^•^ with 1-methyl-2-pyrrolidone (**S3**) 0.154 M, measured in an Ar-saturated MeCN solution at *T* = 25 °C.

CumO^•^ was generated at *T* = 25 °C by 355 nm laser flash photolysis (LFP) of argon-saturated acetonitrile solution containing 1.0 M dicumyl peroxide. Under these conditions, CumO^•^ is characterized by a visible absorption band centered at 485 nm that decays on the μs timescale mainly by C–CH_3_ β-scission.^8^ The reactions of CumO^•^ with the substrates displayed in Chart 1 were studied employing the LFP technique. In the presence of a hydrogen atom-donor substrate, bimolecular HAT to CumO^•^ competes with unimolecular β-scission, and the second-order rate constant *k*_H_ can be determined from the slope of the linear correlation obtained from the observed rate constant (*k*_obs_) *versus* [substrate] plot, where in turn the *k*_obs_ values are measured following the decay of CumO^•^ as a function of the concentration of the added substrate. As an example, the plot of the change in absorbance (Δ*A*) monitored at 490 nm *versus* time for the reaction of CumO^•^ with **S3** (0.154 M) is displayed in Figure 2.

Representative *k*_obs_*versus* [substrate] plots for the reactions of CumO^•^ with 1-methyl-2-pyrrolidone (**S3**, black circles), 1,3-dimethyl-2-imidazolidinone (**S15**, red circles), 3-methyl-2-oxazolidinone (**S18**, green circles), and *N*-methylsuccinimide (**S20**, yellow circles) are displayed in Figure 3. Additional plots for the reactions of the other substrates are displayed in the Supporting Information (Figures SI.1–SI.21).

**Figure 3:**
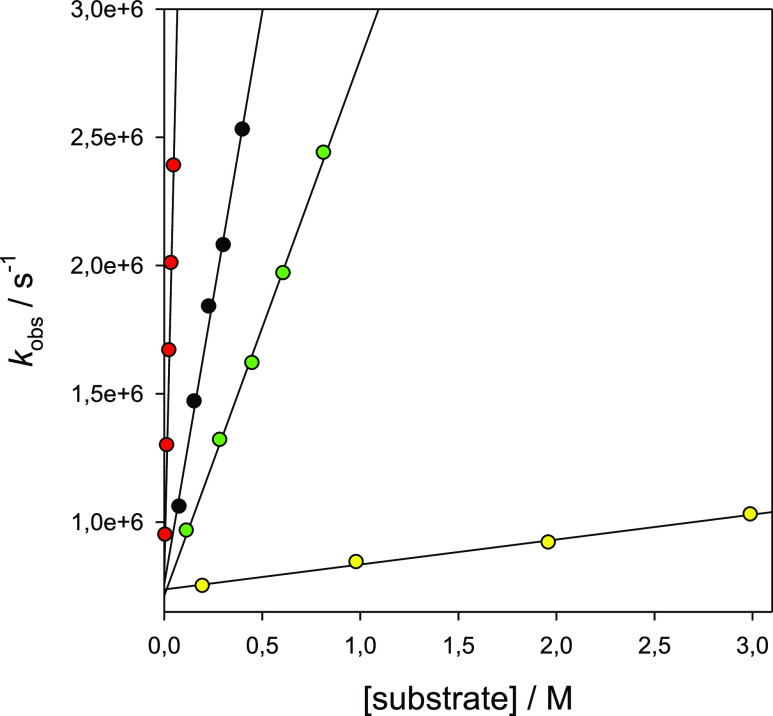
Plots of the observed rate constant (*k*_obs_) against [substrate] for the reactions of the cumyloxyl radical (CumO^•^) with 1-methyl-2-pyrrolidone (**S3**, black circles), 1,3-dimethyl-2-imidazolidinone (**S15**, red circles), 3-methyl-2-oxazolidinone (**S18**, green circles), and *N*-methylsuccinimide (**S20**, yellow circles), measured in an argon-saturated MeCN solution at *T* = 25 °C following the decay of CumO^•^ at 490 nm. From the linear regression analysis: **S3**: intercept: 7.57 × 10^5^ s^–1^, *k*_H_ = 4.47 × 10^6^ M^–1^ s^–1^, and *r*^2^ = 0.9943. **S15**: intercept: 7.89 × 10^5^ s^–1^, *k*_H_ = 3.30 × 10^7^ M^–1^ s^–1^, and *r*^2^ = 0.9982. **S18**: intercept: 7.13 × 10^5^ s^–1^, *k*_H_ = 2.09 × 10^6^ M^–1^ s^–1^, and *r*^2^ = 0.9984. **S20**: intercept: 7.37 × 10^5^ s^–1^, *k*_H_ = 9.73 × 10^4^ M^–1^ s^–1^, and *r*^2^ = 0.9946.

Because of the poor solubility of succinimide (**S19**) in acetonitrile, the reaction of CumO^•^ with this substrate was studied in DMSO. As a matter of comparison, the reactions of 2-pyrrolidone (**S2**), 1-methyl-2-pyrrolidone (**S3**), 2-imidazolidinone (**S14**), and 1,3-dimethyl-2-imidazolidinone (**S15**) were also studied in this solvent. The measured *k*_H_ values for reactions of CumO^•^ with the substrates displayed in Chart 1 are collected in Table 1.

**Table 1:**
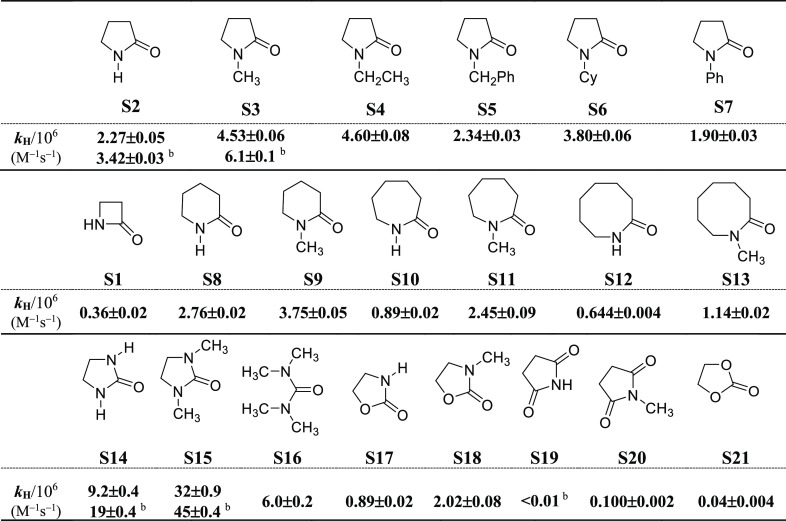
Second-Order Rate Constants (*k*_H_) for Reaction of the Cumyloxyl Radical (CumO^•^) With Lactams and Other Nitrogen-Containing Heterocycles^a^

a Measured in argon-saturated acetonitrile solution at *T* = 25 °C employing 355 nm LFP: [dicumyl peroxide] = 1.0 M. Average of at least two independent experiments.

b Measured in DMSO solution.

Previous studies on the reactions of CumO^•^ with *N*-alkylalkanamides and *N*,*N*-dialkylalkanamides have shown that HAT predominantly occurs from the C–H bonds that are α to nitrogen, with negligible contribution from the C–H bonds that are α to the carbonyl group.^9,10^ The former sites are activated by hyperconjugative overlap between the amide π system and the σ* orbital of the α-C–H bond. The latter C–H bonds are electronically deactivated toward HAT to the electrophilic CumO^•^ by the electron-withdrawing carbonyl group. For example, the *k*_H_ values measured previously in acetonitrile solution for the reactions of CumO^•^ with *N*-methylacetamide (NMA) and *N*,*N*-dimethylacetamide (DMA) (*k*_H_ = 3.18 × 10^5^ and 1.24 × 10^6^ M^–1^s^–1^, respectively) were assigned to HAT from the *N*-methyl groups.^9,11^ The fourfold decrease in *k*_H_ measured on going from the tertiary to secondary amide (twofold on a per-hydrogen basis) was rationalized on the basis of configurational effects. With DMA, the α-C–H bond BDEs were calculated to increase by ∼2 kcal mol^–1^ going from the *trans-N*-methyl group to the *cis* one. With NMA, the *N*-methyl group is held in a *cis*-configuration with respect to the carbonyl group, and this effect accounts for the observed decrease in reactivity.^10^

Along these lines, the *k*_H_ values displayed in Table 1 measured for reaction of CumO^•^ with 2-pyrrolidone (**S2**) and 1-methyl-2-pyrrolidone (**S3**) (*k*_H_ = 2.27 × 10^6^ and 4.53 × 10^6^ M^–1^ s^–1^, respectively) can be again assigned to selective HAT from the C–H bonds that are α to nitrogen, in full agreement with the results of previous studies on C–H bond functionalization of these substrates promoted by different HAT reagents.^12^ Comparison of these values with those measured previously for the corresponding reactions of NMA and DMA evidences a significantly higher reactivity for the α-C–H bonds of the pyrrolidones as compared to the acyclic amides. Such a behavior can be explained on the basis of the operation of stereoelectronic effects. In **S2** and **S3**, the endocyclic α-C–H bonds are held in a conformation that allows for optimal overlap with the amide π system. The twofold increase in *k*_H_ measured going from **S2** to **S3** reasonably reflects the electronic activation of the endocyclic α-C–H bonds and the increased number of reactive sites determined by N-methylation. Unfortunately, the difficulties that are typically associated to product studies of HAT reactions promoted by CumO^•^ do not allow to establish the relative contribution to *k*_H_ derived from abstraction at these two sites.^13^ It is however important to point out that previous studies on the reactions of **S3** with different HAT reagents have shown that functionalization predominantly or almost exclusively occurs at the endocyclic over the exocyclic α-C–H bonds,^12^ indicating that HAT from the N–CH_3_ group contributes to a limited extent to the increase in *k*_H_ measured on going from **S2** to **S3**.

With **S2**, **S3**, **S14**, and **S15**, an increase in *k*_H_ between 1.4- and 2.1-fold was measured going from acetonitrile to DMSO. A comparable increase in reactivity was previously observed in the reactions of CumO^•^ with other substrates. With substrates bearing hydrogen bond donor (HBD) functional groups such as alkanols, 1,2-alkanediols,^14^ and primary alkanediamines,^15^ this behavior was rationalized on the basis of the stronger hydrogen bond acceptor (HBA) ability of DMSO compared to acetonitrile^16^ that, by engaging in hydrogen bonding with the HBD functionality, increases the electron density at this group, leading to an increase in the α-C–H bond reactivity toward the electrophilic HAT reagent CumO^•^. With substrates bearing HBA functional groups such as tertiary alkylamines and alkanediamines,^15^ DMF and DMA,^17^ the greater HBD ability of MeCN compared to DMSO^16^ was put forward to account for the observed behavior. The transition state for HAT from C(sp^3^)–H bonds to oxygen-centered radicals has been described on the basis of a charge-separated structure, characterized by positive and negative charge development at the incipient carbon radical and oxygen center, respectively.^18^ Solvent hydrogen bonding to the HBA functionality will decrease the electron density at the incipient carbon radical center, leading to a destabilization of the transition state and to a corresponding decrease in *k*_H_ as compared to non-HBD solvents. Both explanations can be reasonably invoked to explain the abovementioned increase in reactivity observed in the reactions of **S2**, **S3**, **S14**, and **S15**, on going from acetonitrile to DMSO, with a slightly higher kinetic solvent effect observed for **S2** and **S14**, compared to **S3** and **S15**, respectively, that can be ascribed to the HBD ability of the former two substrates.

Interestingly, an analogous C–H bond activation promoted by HBA additives was recently exploited to promote site-selectivity in the HAT-based functionalization of substrates bearing HBD functional groups such as alkanols and saccharides.^19,20^

Within the 2-pyrrolidone series, the effect of the *N* substituent was also investigated. The *k*_H_ value was observed to increase by a factor of 2.4 on going from the least reactive 1-phenyl-2-pyrrolidone (**S7**) to the most reactive 1-ethyl-2-pyrrolidone (**S4**) (*k*_H_ = 1.90 × 10^6^ and 4.60 × 10^6^ M^–1^ s^–1^, respectively). These results reflect once again the contribution to *k*_H_ derived from HAT from the C–H bonds that are α to nitrogen. With **S4** and 1-benzyl-2-pyrrolidone (**S5**), previous studies on C–H bond functionalization promoted by the *tert*-butoxyl radical and the sulfate radical anion, respectively,^12c,12d^ indicate that HAT-based functionalization exclusively occurs at the endocyclic α-C–H bonds. With 1-cyclohexyl-2-pyrrolidone (**S6**), competitive HAT from the cyclohexane ring positions can be envisaged. Also, with the latter three substrates, electronic effects determined by the *N*-substituent are expected to play an important role in determining site-selectivity, but the kinetic analysis does not provide conclusive information in this respect. The almost identical values measured for the reactions of **S2** and of 1-benzyl-2-pyrrolidone (**S5**) (*k*_H_ = 2.27× 10^6^ and 2.34 × 10^6^ M^–1^ s^–1^, respectively) and the higher values measured for **S3** and **S4** (*k*_H_ = 4.53 × 10^6^ and 4.60 × 10^6^ M^–1^ s^–1^, respectively), despite similar inductive effects exerted by alkyl and benzyl groups,^21^ point toward a lack of benzylic activation for the exocyclic α-C–H bonds of **S5**, an observation that is supported by the results of product studies discussed above.^12c^ This behavior can reflect the operation of deactivating stereoelectronic effects, where the phenyl group increases the energy barrier required to reach the conformation in which the benzylic C–H bond is perpendicular to the plane of the amide, thus preventing, or at least limiting, the contribution of competitive HAT from this site. Within this framework, it is also important to point out that a recent time-resolved kinetic study on HAT from the C(sp^3^)–H bonds of an extended set of substrates to CumO^•^ has shown that the *k*_H_ values measured for HAT from benzylic and allylic C–H bonds are significantly lower than what would be expected on the basis of their relatively low BDE values.^22^ This behavior has been explained in terms of Bernasconi’s principle of nonperfect synchronization,^23^ with the relative importance of benzylic or allylic resonance stabilization that develops late along the reaction coordinate, increasing on going from the HAT transition state to the product radical. Along this line, in order to account for the lack of benzylic activation observed in the reaction of **S5**, also this explanation can be put forward in addition to the abovementioned operation of deactivating stereoelectronic effects.

The data displayed in Table 1 for the secondary and tertiary *N*-methyl lactam derivatives allow an analysis of the effect of ring size on the HAT reactivity. Among all derivatives, the lowest *k*_H_ value was measured in the reaction of CumO^•^ with 2-azetidinone (**S1**, for which *k*_H_ = 3.60 × 10^5^ M^–1^ s^–1^), a behavior that can be accounted for on the basis of C–H bond strengths and of the geometry of the four-membered ring that prevents optimal hyperconjugative α-C–H bond activation. The comparison between the five- to eight-membered derivatives is displayed in Table 2.

**Table 2:**
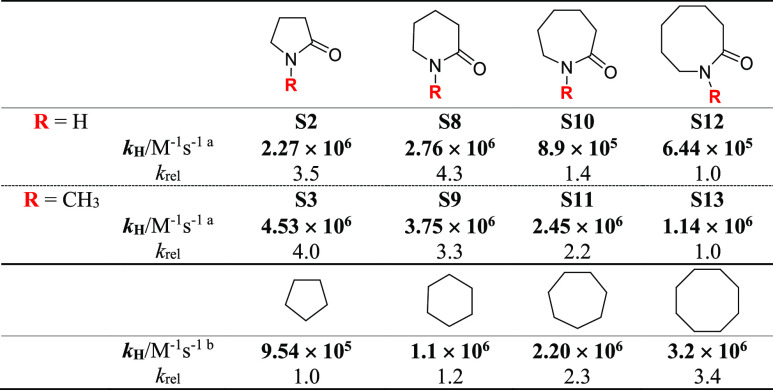
Effect of Ring-Size on the HAT Reactivity of Secondary and Tertiary (*N*-Methyl) Lactams and Cycloalkanes

a Measured in argon-saturated acetonitrile solution at *T* = 25 °C employing 355 nm LFP: [dicumyl peroxide] = 1.0 M. Average of at least two independent experiments.

b Reference (22).

Although within the two series, comparable reactivities were observed for five- and six-membered ring derivatives, the *k*_H_ value was then observed to decrease with increasing ring size, approaching a ∼fourfold decrease in reactivity on going from **S8** to **S12** and from **S3** to **S13**.

It is also interesting to compare these results with those obtained previously from the corresponding reactions of CumO^•^ with cycloalkanes,^22^ where the *k*_H_ value was instead observed to increase with increasing ring size, approaching a factor 3.4 going from cyclopentane to cyclooctane. The increase in *k*_H_ with increasing ring size observed along the cycloalkane series can be rationalized on the basis of the increase in the number of abstractable C–H bonds and the lower BDE associated to the C–H bonds of cycloheptane and cyclooctane as compared to cyclohexane and, to a lesser extent, cyclopentane.^22^ The normalized rate constants (*k*_H_′) for HAT from a single C–H bond of these substrates can be derived as *k*_H_′ = 9.54 × 10^4^, 9.2 × 10^4^, 1.57 × 10^5^, and 2.0 × 10^5^ M^–1^ s^–1^, for cyclopentane, cyclohexane, cycloheptane, and cyclooctane, respectively. The opposite reactivity trends observed along the secondary and tertiary *N*-methyl lactam series suggest that ring positions other than the one that is α to nitrogen do not contribute to a significant extent to the overall reactivity. These trends indicate moreover that the reactivity of the α-C–H bonds progressively decreases on going from the five- and six-membered-ring derivatives to the corresponding seven- and eight-membered ones. It seems reasonable to propose that this decrease in reactivity reflects the greater flexibility of the latter ring systems as compared to the former ones, a structural feature that prevents optimal hyperconjugation between the α-C–H bonds and the lactam π system. Theoretical calculations and studies on other cyclic substrate series may provide information that can allow to better rationalize this intriguing observation.

It is finally very interesting to compare the results obtained with the five-membered-ring substrates, classified on the basis of the four substrate types 2-pyrrolidones, 2-imidazolidinones, 2-oxazolidinones, and succinimides, the analysis of which provides important information on the role of substrate structure on HAT reactivity (Table 3). All substrates display a rigid five-membered-ring framework that should ensure hyperconjugative activation of the C–H bonds that are adjacent to nitrogen, with carbonyl groups and additional ring heteroatoms that, on the other hand, are expected to tune the extent of electronic deactivation. In all cases, *k*_H_ was observed to increase on going from the secondary to the corresponding tertiary (*N*-methyl) derivative, in line with the abovementioned electronic activation of the endocyclic α-C−H bonds determined by *N*-methylation, and the increase in the number of activated α-C–H bonds.

**Table 3:**
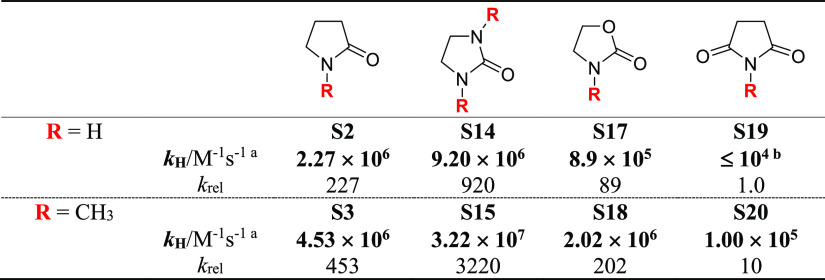
Effect of the Substrate Structure on the HAT Reactivity of Five-Membered-Ring Nitrogen-Containing Heterocycles

a Measured in argon-saturated acetonitrile solution at *T* = 25 °C employing 355 nm LFP: [dicumyl peroxide] = 1.0 M. Average of at least two independent experiments.

b Measured in DMSO solution.

The lowest reactivity was observed with succinimide (**S19**), for which only an upper limit for *k*_H_ could be obtained (*k*_H_ ≤ 10^4^ M^–1^ s^–1^). This substrate lacks the presence of C–H bonds that are α to nitrogen and is essentially non-reactive toward CumO^•^, in line with the strong electronic deactivation of all C–H bonds exerted by the two carbonyl groups.^7a^

**Scheme 1:**
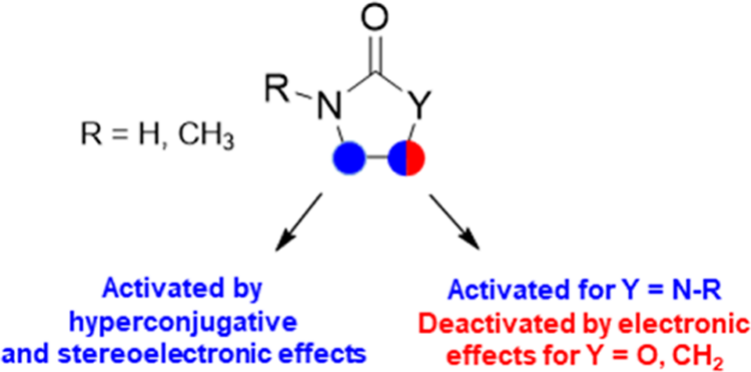
Factors Governing Reactivity and Selectivity in HAT to CumO^•^

An at least one order of magnitude increase in reactivity (*k*_rel_ ≥ 10) was observed following N-methylation as in **S20**, indicating that HAT now selectively occurs from the *N*-methyl group, in line with the results of previous studies on the radical-based oxidation of this substrate.^24^ However, the relatively low *k*_H_ value reflects the divergent contribution of deactivating electronic effects and hyperconjugative activation. Significantly higher *k*_H_ values were measured for the other six derivatives displayed in Table 3, with *k*_rel_ values that were observed to progressively increase going from the 2-oxazolidinone to the 2-pyrrolidone and 2-imidazolidinone substrate groups, ranging between 89 for 2-oxazolidinone (**S17**) and 3220 for 1,3-dimethyl-2-imidazolidinone (**S15**). Within the latter three substrate groups and taking **S19** as the reference compound, an increase in *k*_H_ that exceeds two orders of magnitude was observed for the 2-pyrrolidones **S2** and **S3** (*k*_rel_ = 227 and 453, respectively). The observed increase in reactivity reflects the replacement of a deactivating carbonyl group in **S19** and **S20** with methylene, with the two C–H bonds that are α to nitrogen that can now benefit from hyperconjugative activation provided by the amide functionality. A weaker activation was instead observed for the 2-oxazolidinones **S17** and **S18** (*k*_rel_ = 89 and 202, respectively), a behavior that can be accounted for on the basis of the inductive electron-withdrawing effect exerted by the remote endocyclic oxygen atom that, as compared to a methylene group, slightly deactivates the C–H bonds that are α to nitrogen.^14^ The ∼22-fold increase in *k*_H_ measured on going from ethylene carbonate (**S21**, not shown in Table 3) to **S17** (*k*_H_ = 4.0 × 10^4^ and 8.9 × 10^5^ M^–1^ s^–1^, respectively) can be explained accordingly on the basis of the replacement of NH for O that reduces hyperconjugative activation of the C–H bonds that are adjacent to the heteroatoms. Along similar lines, a very large increase in *k*_H_ was observed with the 2-imidazolidinones **S14** and **S15**, approaching or exceeding three orders of magnitude (*k*_rel_ = 920 and 3220, respectively). With these substrates, all C–H bonds are now adjacent to an amide-type nitrogen atom and are thus activated toward HAT to CumO^•^. Comparison between the *k*_H_ values measured for **S15** and for the structurally analogous acyclic substrate tetramethylurea (**S16**) evidences a greater than fivefold decrease in reactivity on going from the former to the latter substrate. This result highlights once again the importance of a rigid framework that ensures optimal overlap between the abstractable C–H bonds and the amide π system, maximizing activation at these sites. Overall, the data displayed in Table 3 clearly show that by leveraging on hyperconjugative activation and deactivating electronic effects, differences in reactivity that exceed three orders of magnitude can be achieved, highlighting the role played by these effects in HAT from the C(sp^3^)–H bonds of these five-membered heterocycles, exemplified graphically in Scheme 1.

Taken together, the results presented in this work provide useful information on the role of structural effects in C(sp^3^)–H bond reactivity. Hyperconjugative activation of C–H bonds that are adjacent to amide functionalities can be tuned by varying ring size and rigidity, with the extent of this activation that is highest with five- and six-membered rings and then decreases with increasing ring size. Electronic effects, promoted by endocyclic carbonyl groups and (remote) oxygen atoms, exert instead deactivating effects that can compensate or override the abovementioned activating effects. This information can be possibly employed to implement site-selectivity in HAT-based late-stage C–H bond functionalization of molecules bearing nitrogen heterocyclic structures.

## Experimental Section

### Materials

All the substrates employed in the time-resolved kinetic studies were commercially available and were used as received or purified by ordinary techniques in order to reach a ≥99% purity. Commercially available dicumyl peroxide (≥98%) was used without further purification. Spectroscopic- or HPLC-grade acetonitrile (MeCN) and dimethylsulfoxide (DMSO) were employed for the time-resolved kinetic studies.

### LFP Studies

LFP experiments were carried out with a laser kinetic spectrometer using the third harmonic (355 nm) of a Q-switched Nd:YAG laser, delivering 8 ns pulses. The laser energy was adjusted to ≤10 mJ/pulse by the use of an appropriate filter. A 3.5 mL Suprasil quartz cell (10 mm × 10 mm) was used in all experiments. Nitrogen-saturated MeCN solutions of dicumyl peroxide (1.0 M) were employed. Because of the very low solubility displayed by succinimide (**S19**) in MeCN, the kinetic study of the reaction of the cumyloxyl radical (CumO^•^) with **S19** was carried out in DMSO. As a matter of comparison, the reactions of 2-pyrrolidone (**S2**), 1-methyl-2-pyrrolidone (**S3**), 2-imidazolidinone (**S14**), and 1,3-dimethyl-2-imidazolidinone (**S15**) were also studied in this solvent. All the experiments were carried out at *T* = 25 ± 0.5 °C under magnetic stirring. The primary kinetic data are provided in the Supporting Information as tables of absorbance *versus* time. The observed rate constants (*k*_obs_) were obtained by averaging three–five individual values and were reproducible to within 5%. Representative plots of the change in absorbance *versus* time at different substrate concentrations for the reactions of CumO^•^ with **S3** and **S10**, employed for *k*_obs_ determination, are displayed in the Supporting Information. Second-order rate constants (*k*_H_) for the reactions of CumO^•^ with substrates **S1–S21** were obtained from the slopes of the *k*_obs_ (measured following the decay of the cumyloxyl radical visible absorption band at 490 nm) *versus* [substrate] plots. Fresh solutions were used for every substrate concentration. Correlation coefficients were in all cases >0.99. The *k*_H_ values are the average of at least two independent experiments, typical errors being ≤10%.

## Supporting Information Available

Primary kinetic data and plots of *k*_obs_*versus* substrate concentration for the reactions of CumO^•^ with different substrates (PDF)
