# Supplementary material for: Factors Governing Reactivity and Selectivity in Hydrogen Atom Transfer from C(sp3)–H Bonds of Nitrogen-Containing Heterocycles to the Cumyloxyl Radical
Source: J Org Chem. 2022 May 24;87(11):7456–63. doi: 10.1021/acs.joc.2c00955 (PMC9171822; doi:10.1021/acs.joc.2c00955)
Supplement: Supplementary file 1 — jo2c00955_si_001.pdf [file jo2c00955_si_001.pdf]

SUPPORTING INFORMATION

Factors Governing Reactivity and Selectivity in Hydrogen Atom Transfer from C(*sp*^{3})–H Bonds of Nitrogen Containing Heterocycles to the Cumyloxyl Radical.

Marco Galeotti, Chiara Trasatti, Sergio Sisti, Michela Salamone,* and Massimo Bietti*

Dipartimento di Scienze e Tecnologie Chimiche, Università "Tor Vergata", Via della Ricerca Scientifica, 1 I-00133 Rome, Italy.

E-mail: michela.salamone@uniroma2.it, bietti@uniroma2.it

**1)**	**Chart of the substrates**	**S2**
**2)**	**Laser flash photolysis studies**	**S3**
	**2.1) k_{obs} vs [substrate] plots**	**S3**
	**2.1a) Absorbance vs. time plots for the reaction of S3 with CumO$^•$**	**S5**
	**2.1b) Absorbance vs. time plots for the reaction of S10 with CumO$^•$**	**S14**
	**2.2) Primary kinetic data**	**S26**

1) Chart of the substrates

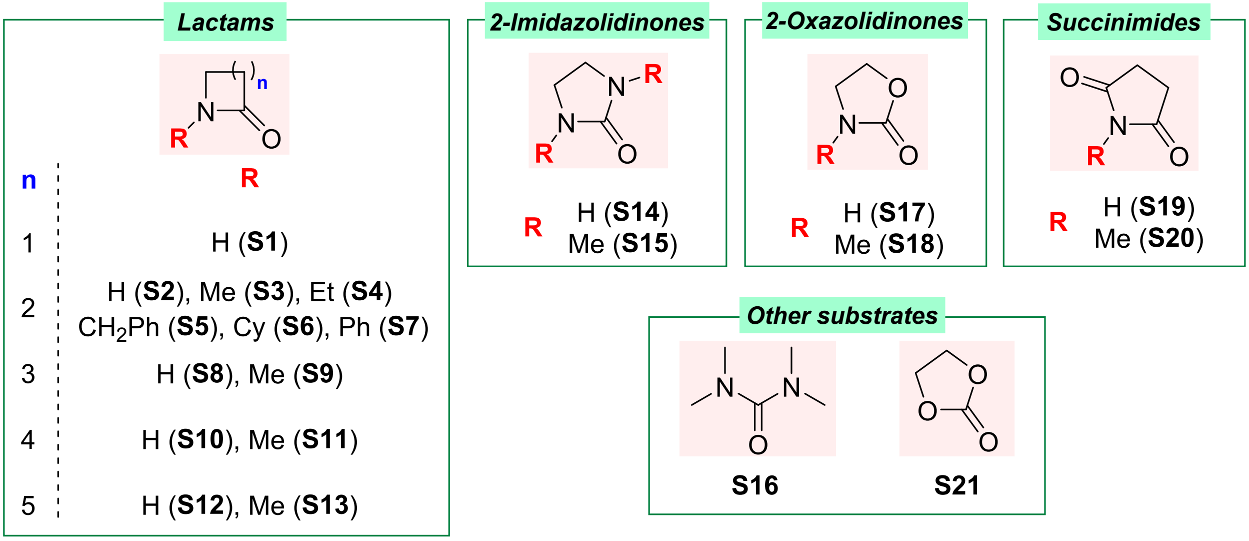

2) Laser flash photolysis studies

2.1) k_{obs} vs [substrate] plots

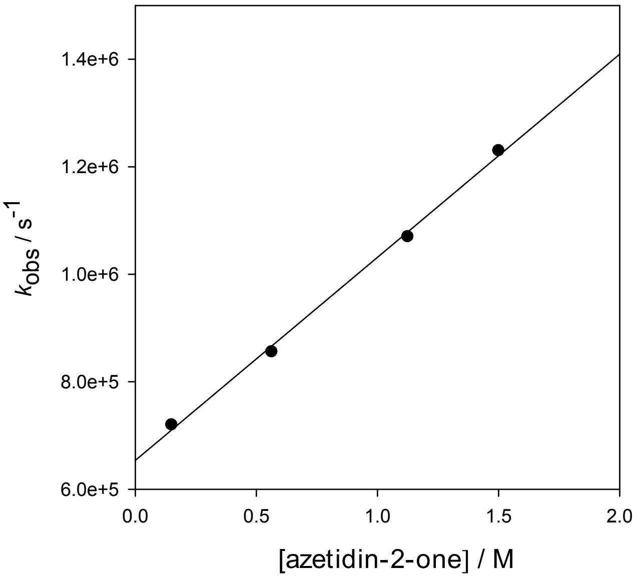

Figure SI.1. Plot of the observed rate constant (k_{obs}) against [substrate] for the reaction of 2-azetidinone (**S1**) with CumO• generated by 355 nm LFP of an Ar-saturated MeCN solution containing 1.0 M dicumyl peroxide measured at T = 25 °C following the decay of CumO• at 490 nm. From the linear regression analysis: intercept = 6.54×10^5 s^{-1}, $k_H = 3.78 \times 10^5$ M^{-1} s^{-1}, r^2 = 0.9976.

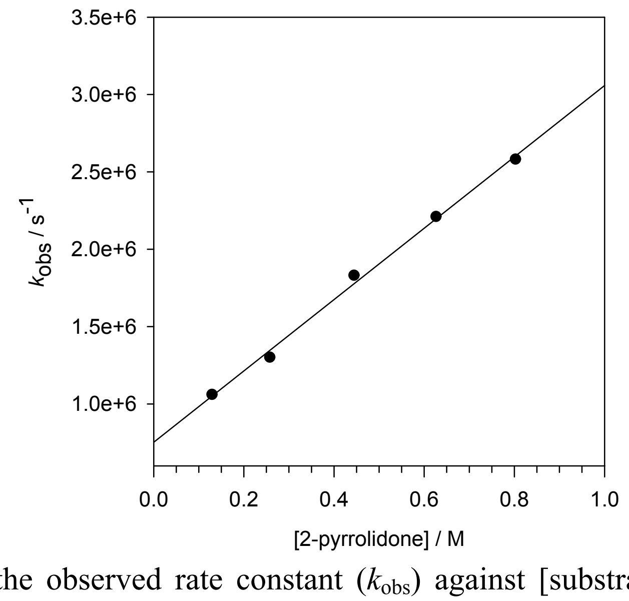

Figure SI.2A. Plot of the observed rate constant (k_{obs}) against [substrate] for the reaction of 2-pyrrolidone (**S2**) with CumO• generated by 355 nm LFP of an Ar-saturated MeCN solution containing 1.0 M dicumyl peroxide measured at T = 25 °C following the decay of CumO• at 490 nm. From the linear regression analysis: intercept = 7.52×10^5 s^{-1}, $k_H = 2.31 \times 10^6$ M^{-1} s^{-1}, r^2 = 0.9964.

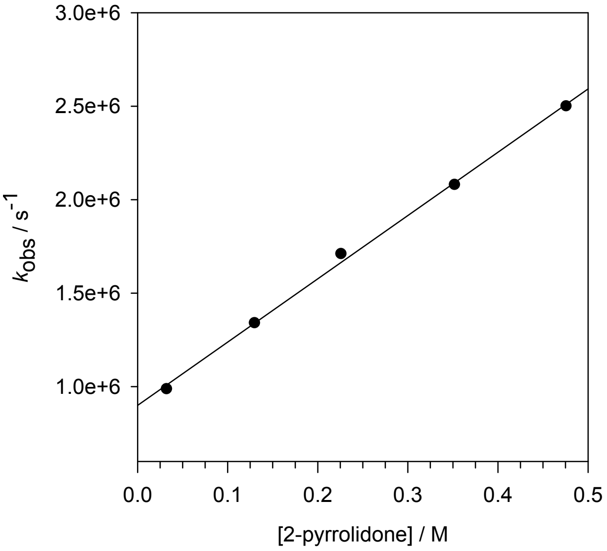

Figure SI.2B. Plot of the observed rate constant (k_{obs}) against [substrate] for the reaction of 2-pyrrolidone (**S2**) with CumO• generated by 355 nm LFP of an Ar-saturated DMSO solution containing 1.0 M dicumyl peroxide measured at T = 25 °C following the decay of CumO• at 490 nm. From the linear regression analysis: intercept = 9.00×10^5 s^{-1}, k_H = 3.39×10^6 M^{-1} s^{-1}, r^2 = 0.9980.

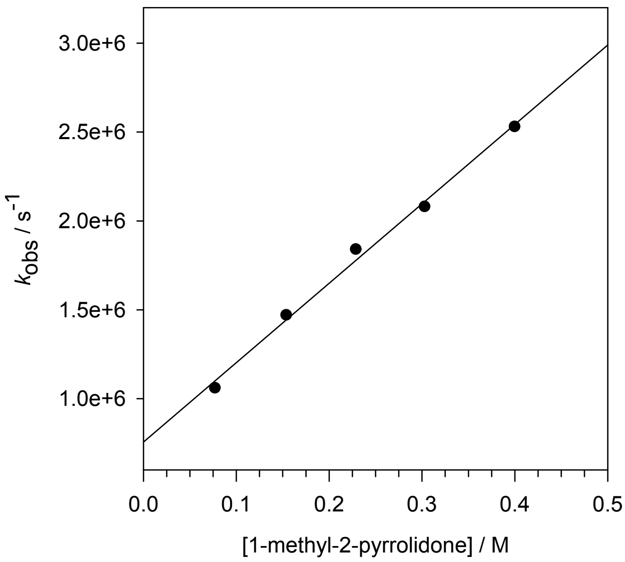

Figure SI.3A. Plot of the observed rate constant (k_{obs}) against [substrate] for the reaction of 1-methyl-2-pyrrolidone (**S3**) with CumO• generated by 355 nm LFP of an Ar-saturated MeCN solution containing 1.0 M dicumyl peroxide measured at T = 25 °C following the decay of CumO• at 490 nm. From the linear regression analysis: intercept = 7.57×10^5 s^{-1}, k_H = 4.47×10^6 M^{-1} s^{-1}, r^2 = 0.9943.

2.1a) Absorbance vs. time plots for the reaction of S3 with CumO• (MeCN)

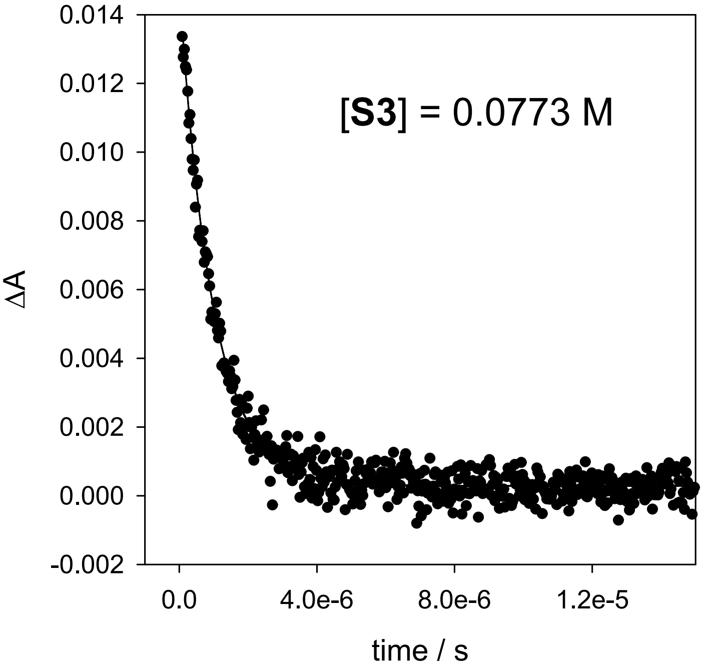

Figure SI.3A-1 Plot of the change in absorbance (ΔA) monitored at 490 nm vs. time for the reaction of 1-methyl-2-pyrrolidone (**S3**) 0.0773 M with CumO• measured in Ar-saturated MeCN solution at T = 25 °C.

Nonlinear Regression
Equation: Exponential Decay, Single, 3 Parameter
f=y0+a*exp(-b*x)

**R**	**Rsqr**	**Adj Rsqr**	**Standard Error of Estimate**
0.846	0.9693	0.9692	0.0004

	**Coefficient**	**Std. Error**	**t**	**P**	**VIF**
y0	0.0003	2.1613E-005	12.9918	<0.0001	1.3493
a	0.0146	0.0002	84.3541	<0.0001	2.3632
b	1036351.2897	17326.7200	59.8123	<0.0001	2.7702

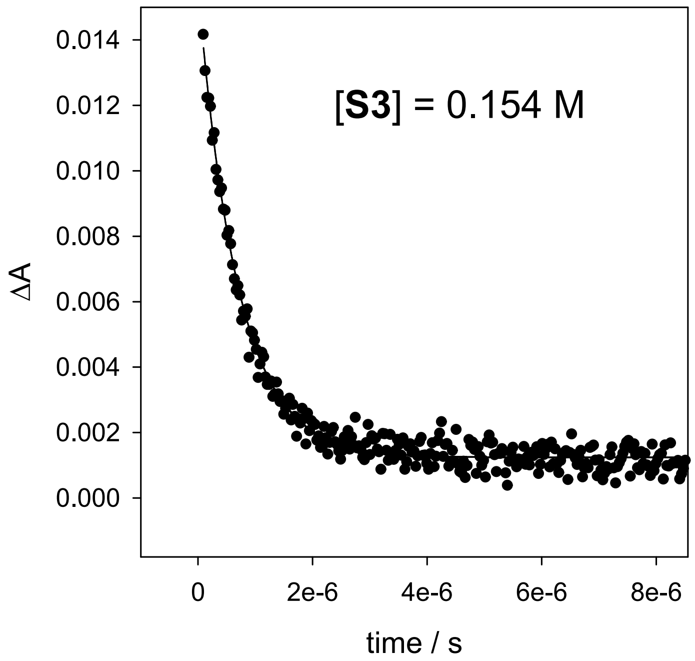

Figure SI.3A-2. Plot of the change in absorbance (ΔA) monitored at 490 nm vs. time for the reaction of 1-methyl-2-pyrrolidone (**S3**) 0.154 M with CumO• measured in Ar-saturated MeCN solution at T = 25 °C.

Nonlinear Regression

Equation: Exponential Decay, Single, 3 Parameter
f=y0+a*exp(-b*x)

R	Rsqr	Adj Rsqr	Standard Error of Estimate
0.9896	0.9793	0.9791	0.0003

	Coefficient	Std. Error	t	P	VIF
y0	0.0012	2.6049E-005	47.4988	<0.0001	1.4871
a	0.0144	0.0002	76.3337	<0.0001	2.5285
b	1445822.9819	26572.3476	54.4108	<0.0001	3.1280

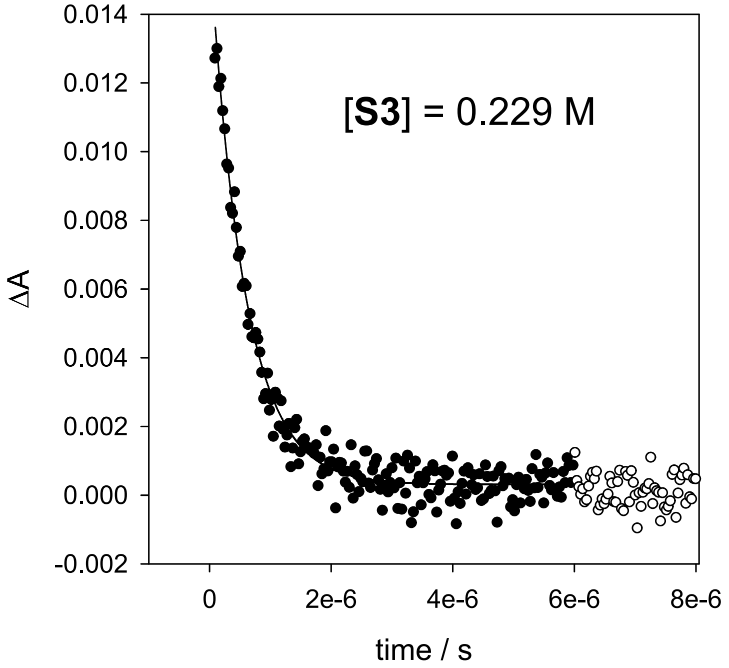

Figure SI.3A-3. Plot of the change in absorbance (ΔA) monitored at 490 nm vs. time for the reaction of 1-methyl-2-pyrrolidone (**S3**) 0.229 M with CumO• measured in Ar-saturated MeCN solution at T = 25 °C. The white data points have been excluded from the nonlinear regression analysis.

Nonlinear Regression

Equation: Exponential Decay, Single, 3 Parameter
f=y0+a*exp(-b*x)

R	Rsqr	Adj Rsqr	Standard Error of Estimate
0.9862	0.9725	0.9722	0.0005

	Coefficient	Std. Error	t	P	VIF
y0	0.0003	4.2654E-005	7.2177	<0.0001	1.6193
a	0.0158	0.0003	54.6468	<0.0001	2.6695
b	1766296.8946	45292.8456	38.9973	<0.0001	3.4634

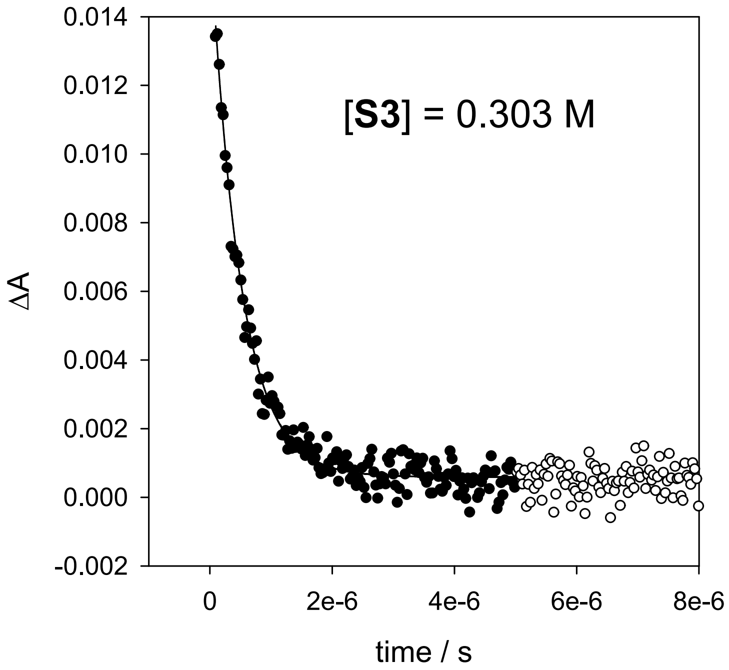

Figure SI.3A-4. Plot of the change in absorbance (ΔA) monitored at 490 nm vs. time for the reaction of 1-methyl-2-pyrrolidone (**S3**) 0.303 M with CumO• measured in Ar-saturated MeCN solution at T = 25 °C. The white data points have been excluded from the nonlinear regression analysis.

Nonlinear Regression

Equation: Exponential Decay, Single, 3 Parameter
f=y0+a*exp(-b*x)

R	Rsqr	Adj Rsqr	Standard Error of Estimate
0.9890	0.9781	0.9778	0.0004

	Coefficient	Std. Error	t	P	VIF
y0	0.0006	4.2357E-005	14.0155	<0.0001	1.6548
a	0.0160	0.0003	54.7828	<0.0001	2.7995
b	2050697.4746	51574.8701	39.7616	<0.0001	3.6687

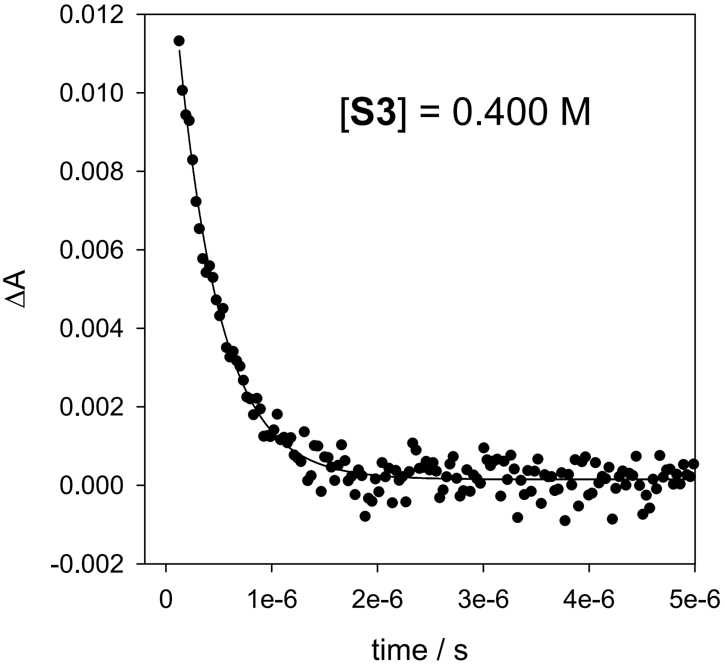

Figure SI.3A-5. Plot of the change in absorbance (ΔA) monitored at 490 nm vs. time for the reaction of 1-methyl-2-pyrrolidone (**S3**) 0.400 M with CumO$^{\bullet}$ measured in Ar-saturated MeCN solution at T = 25 °C.

Nonlinear Regression

Equation: Exponential Decay, Single, 3 Parameter
f=y0+a*exp(-b*x)

R	Rsqr	Adj Rsqr	Standard Error of Estimate
0.9832	0.9667	0.9663	0.0004

	Coefficient	Std. Error	t	P	VIF
y0	0.0001	3.8745E-005	3.8609	0.0002	1.4847
a	0.0150	0.0004	38.4952	<0.0001	3.5169
b	2504513.4144	77406.3885	32.3554	<0.0001	4.2740

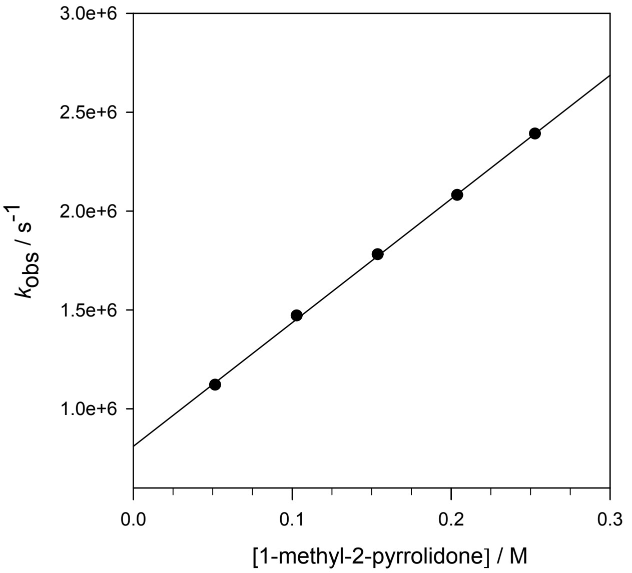

Figure SI.3B. Plot of the observed rate constant (k_{obs}) against [substrate] for the reaction of 1-methyl-2-pyrrolidone (**S3**) with CumO$^{\bullet}$ generated by 355 nm LFP of an Ar-saturated DMSO solution containing 1.0 M dicumyl peroxide measured at T = 25 °C following the decay of CumO$^{\bullet}$ at 490 nm. From the linear regression analysis: intercept = 8.10×10^5 s^{-1}, $k_H = 6.26 \times 10^6$ M^{-1} s^{-1}, r^2 = 0.9995.

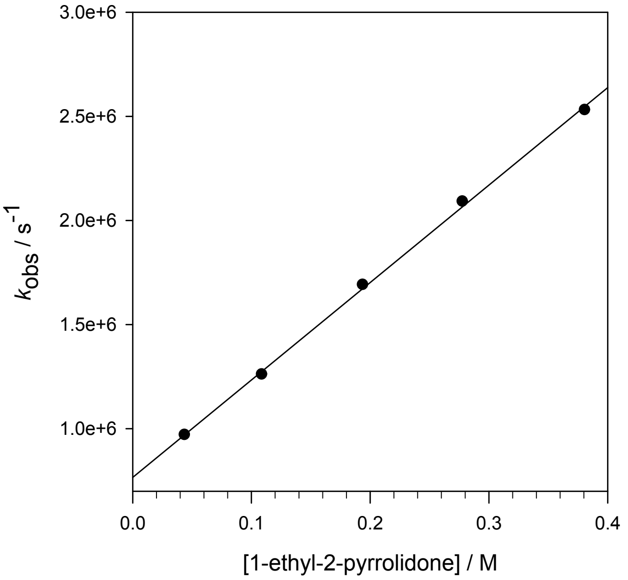

Figure SI.4. Plot of the observed rate constant (k_{obs}) against [substrate] for the reaction of 1-ethyl-2-pyrrolidone (**S4**) with CumO$^{\bullet}$ generated by 355 nm LFP of an Ar-saturated MeCN solution containing 1.0 M dicumyl peroxide measured at T = 25 °C following the decay of CumO$^{\bullet}$ at 490 nm. From the linear regression analysis: intercept = 7.66×10^5 s^{-1}, $k_H = 4.68 \times 10^6$ M^{-1} s^{-1}, r^2 = 0.9991.

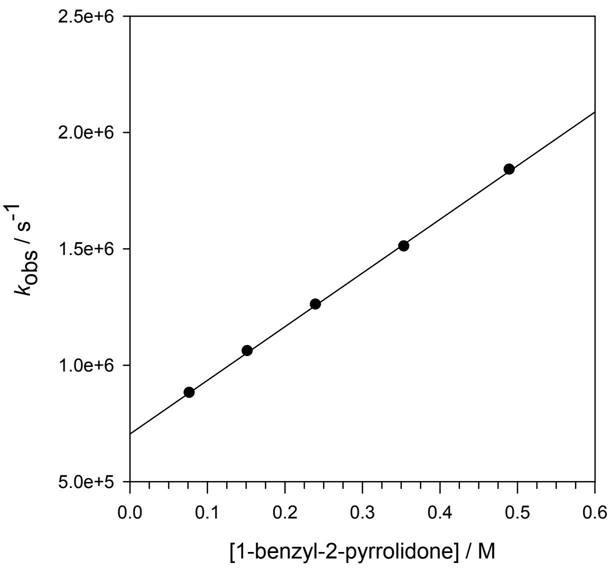

Figure SI.5. Plot of the observed rate constant (k_{obs}) against [substrate] for the reaction of 1-benzyl-2-pyrrolidone (**S5**) with CumO$^{\bullet}$ generated by 355 nm LFP of an Ar-saturated MeCN solution containing 1.0 M dicumyl peroxide measured at T = 25 °C following the decay of CumO$^{\bullet}$ at 490 nm. From the linear regression analysis: intercept = 7.05 × 10^5 s^{-1}, k_H = 2.31 × 10^6 M^{-1} s^{-1}, r^2 = 0.9997.

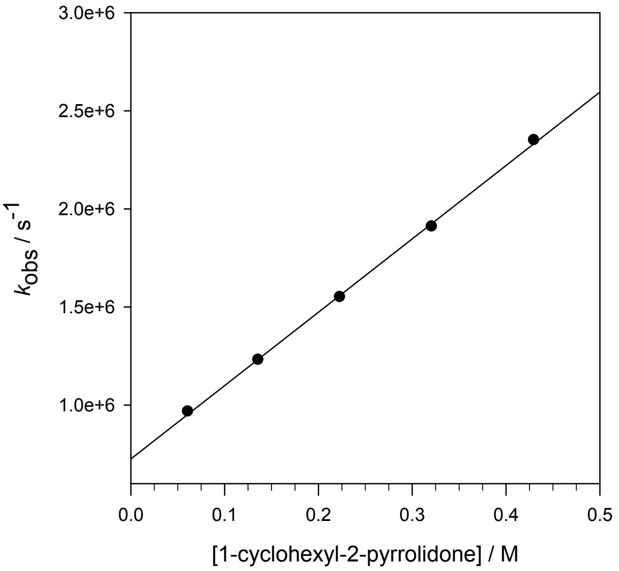

Figure SI.6. Plot of the observed rate constant (k_{obs}) against [substrate] for the reaction of 1-cyclohexyl-2-pyrrolidone (**S6**) with CumO$^{\bullet}$ generated by 355 nm LFP of an Ar-saturated MeCN solution containing 1.0 M dicumyl peroxide measured at T = 25 °C following the decay of CumO$^{\bullet}$ at 490 nm. From the linear regression analysis: intercept = 7.25 × 10^5 s^{-1}, k_H = 3.74 × 10^6 M^{-1} s^{-1}, r^2 = 0.9993.

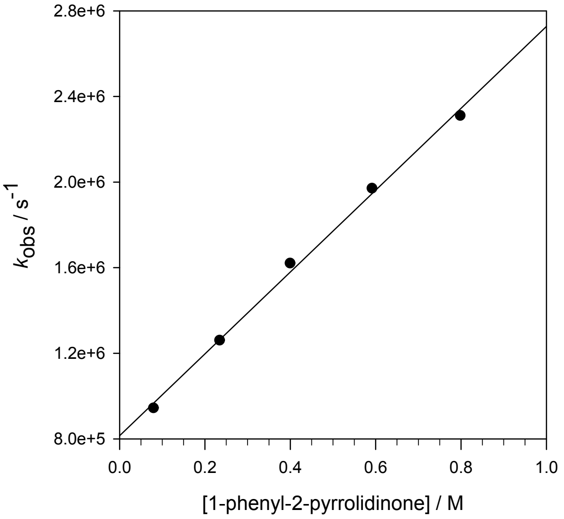

Figure SI.7. Plot of the observed rate constant (k_{obs}) against [substrate] for the reaction of 1-phenyl-2-pyrrolidone (**S7**) with $CumO^•$ generated by 355 nm LFP of an Ar-saturated MeCN solution containing 1.0 M dicumyl peroxide measured at T = 25 °C following the decay of $CumO^•$ at 490 nm. From the linear regression analysis: intercept = 8.15×10^5 s^{-1}, $k_H = 1.91 \times 10^6$ M^{-1} s^{-1}, r^2 = 0.9967.

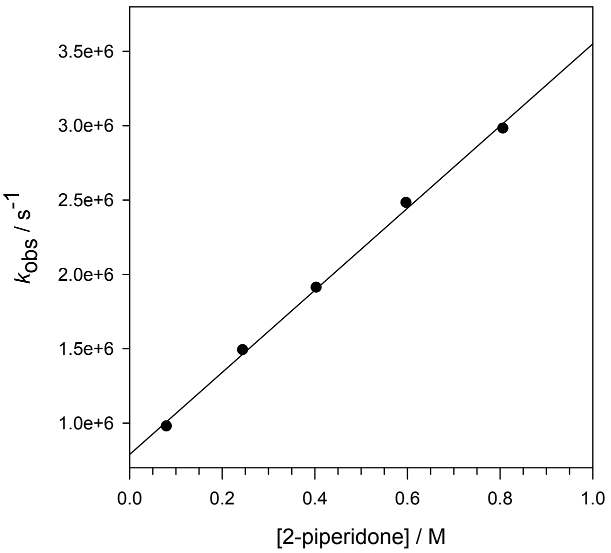

Figure SI.8. Plot of the observed rate constant (k_{obs}) against [substrate] for the reaction of 2-piperidinone (**S8**) with $CumO^•$ generated by 355 nm LFP of an Ar-saturated MeCN solution containing 1.0 M dicumyl peroxide measured at T = 25 °C following the decay of $CumO^•$ at 490 nm. From the linear regression analysis: intercept = 7.88×10^5 s^{-1}, $k_H = 2.76 \times 10^6$ M^{-1} s^{-1}, r^2 = 0.9981.

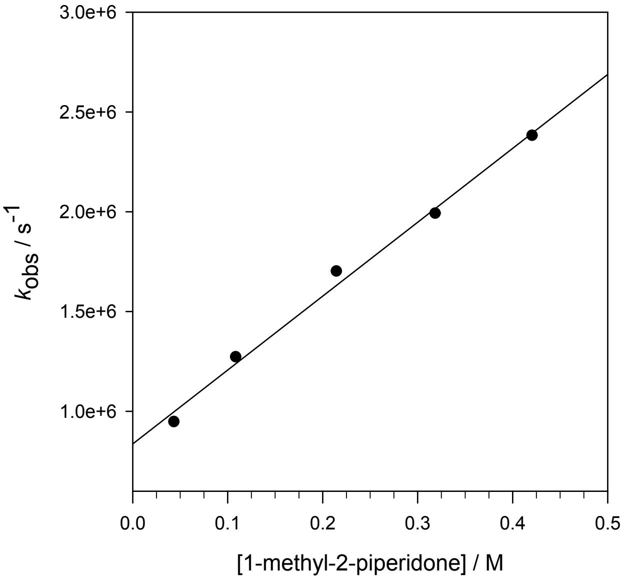

Figure SI.9. Plot of the observed rate constant (k_{obs}) against [substrate] for the reaction of 1-methyl-2-piperidinone (**S9**) with CumO• generated by 355 nm LFP of an Ar-saturated MeCN solution containing 1.0 M dicumyl peroxide measured at T = 25 °C following the decay of CumO• at 490 nm. From the linear regression analysis: intercept = 8.37×10^5 s^{-1}, $k_H = 3.70 \times 10^6$ M^{-1} s^{-1}, r^2 = 0.9928.

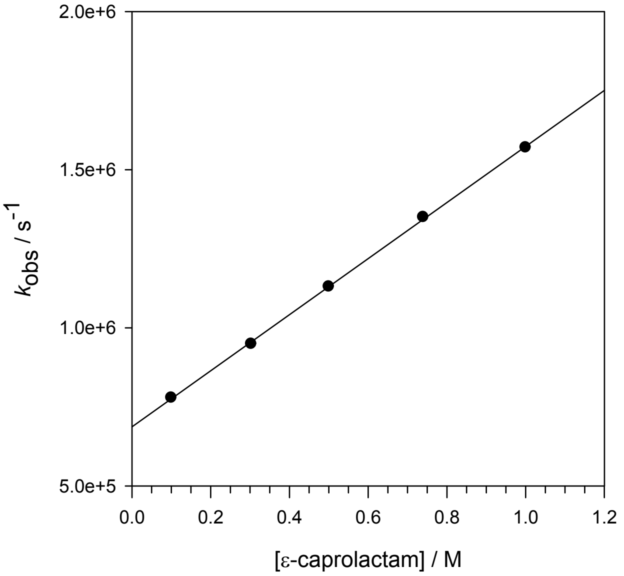

Figure SI.10. Plot of the observed rate constant (k_{obs}) against [substrate] for the reaction of ε-caprolactam (**S10**) with CumO• generated by 355 nm LFP of an Ar-saturated MeCN solution containing 1.0 M dicumyl peroxide measured at T = 25 °C following the decay of CumO• at 490 nm. From the linear regression analysis: intercept = 6.87×10^5 s^{-1}, $k_H = 8.86 \times 10^5$ M^{-1} s^{-1}, r^2 = 0.9997.

2.1b) Absorbance vs. time plots for the reaction of S10 with CumO•

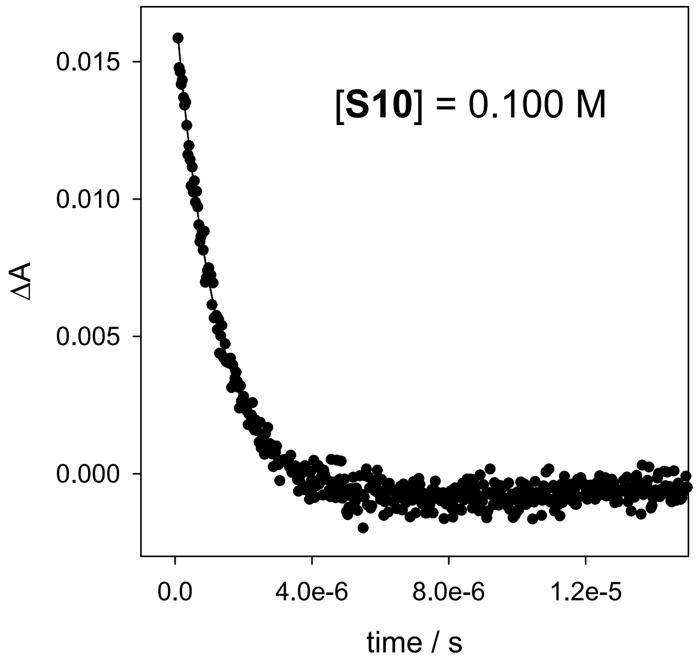

Figure SI.10-1. Plot of the change in absorbance (ΔA) monitored at 490 nm vs. time for the reaction of ε-caprolactam (**S10**) 0.100 M with CumO• measured in Ar-saturated MeCN solution at T = 25 °C.

Nonlinear Regression

Equation: Exponential Decay, Single, 3 Parameter
f=y0+a*exp(-b*x)

**R**	**Rsqr**	**Adj Rsqr**	**Standard Error of Estimate**
0.9916	0.9833	0.9833	0.0004

	**Coefficient**	**Std. Error**	**t**	**P**	**VIF**
y0	-0.0008	2.2435E-005	-34.1576	<0.0001	1.4648
a	0.0179	0.0002	119.0124	<0.0001	2.2920
b	845254.0020	10416.5545	81.1453	<0.0001	2.8195

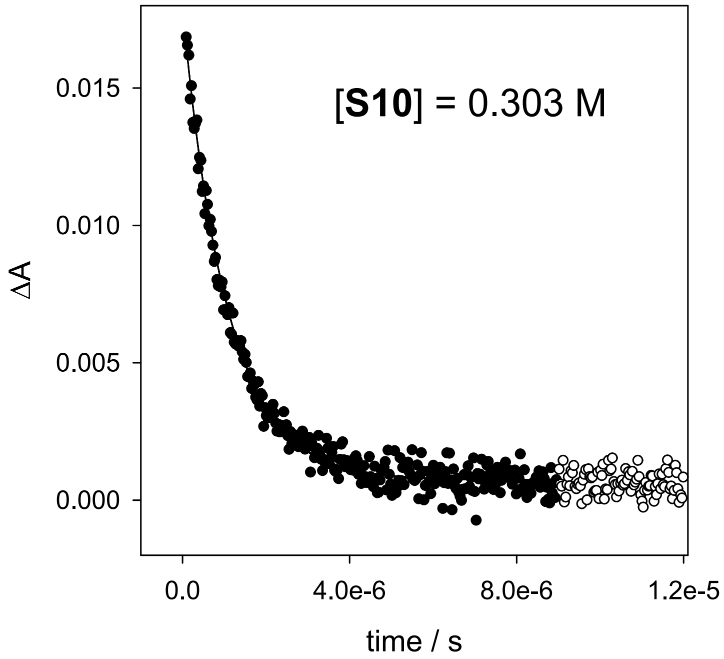

Figure SI.10-2. Plot of the change in absorbance (ΔA) monitored at 490 nm vs. time for the reaction of ε-caprolactam (**S10**) 0.303 M with CumO• measured in Ar-saturated MeCN solution at T = 25 °C. The white data points have been excluded from the nonlinear regression analysis.

Nonlinear Regression

Equation: Exponential Decay, Single, 3 Parameter
f=y0+a*exp(-b*x)

**R**	**Rsqr**	**Adj Rsqr**	**Standard Error of Estimate**
0.9927	0.9855	0.9854	0.0004

	**Coefficient**	**Std. Error**	**t**	**P**	**VIF**
y0	0.0007	3.4330E-005	21.2640	<0.0001	1.9123
a	0.0172	0.0002	102.9571	<0.0001	2.3303
b	934959.3194	14350.4176	65.1521	<0.0001	3.3752

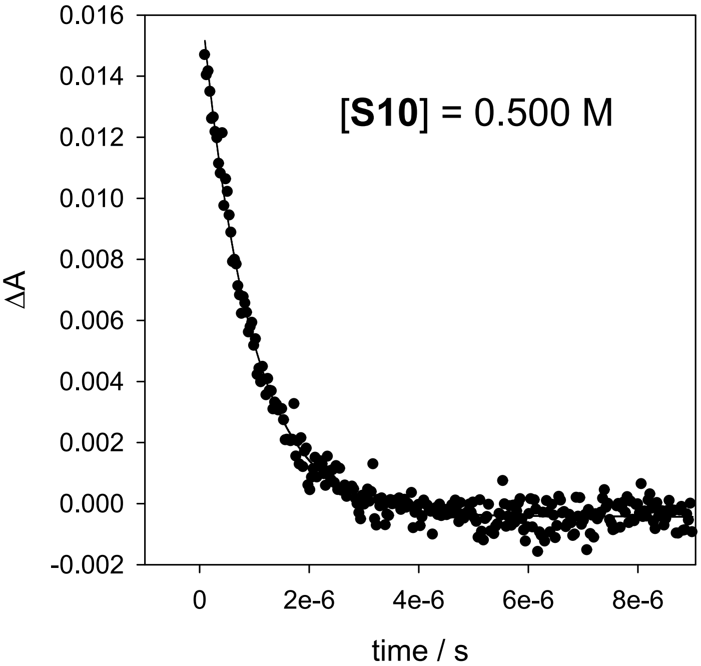

Figure SI.10-3. Plot of the change in absorbance (ΔA) monitored at 490 nm vs. time for the reaction of ε-caprolactam (**S10**) 0.500 M with CumO$^{\bullet}$ measured in Ar-saturated MeCN solution at T = 25 °C.

Nonlinear Regression

Equation: Exponential Decay, Single, 3 Parameter
f=y0+a*exp(-b*x)

R	Rsqr	Adj Rsqr	Standard Error of Estimate
0.9918	0.9836	0.9835	0.0004

	Coefficient	Std. Error	t	P	VIF
y0	-0.0004	3.1768E-005	-13.4199	<0.0001	1.6620
a	0.0174	0.0002	92.7970	<0.0001	2.4018
b	1123698.9191	18031.5659	62.3184	<0.0001	3.1821

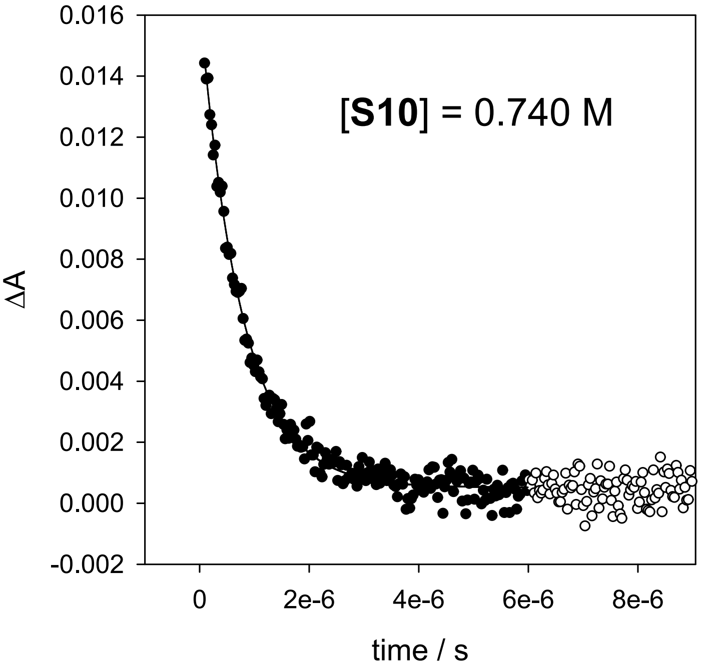

Figure SI.10-4. Plot of the change in absorbance (ΔA) monitored at 490 nm vs. time for the reaction of ε-caprolactam (**S10**) 0.740 M with CumO$^{\bullet}$ measured in Ar-saturated MeCN solution at T = 25 °C. The white data points have been excluded from the nonlinear regression analysis.

Nonlinear Regression

Equation: Exponential Decay, Single, 3 Parameter
f=y0+a*exp(-b*x)

R	Rsqr	Adj Rsqr	Standard Error of Estimate
0.9931	0.9862	0.9860	0.0004

	Coefficient	Std. Error	t	P	VIF
y0	0.0005	3.8921E-005	13.2621	<0.0001	2.0345
a	0.0158	0.0002	83.7675	<0.0001	2.4880
b	1319462.1992	24583.9699	53.6716	<0.0001	3.7343

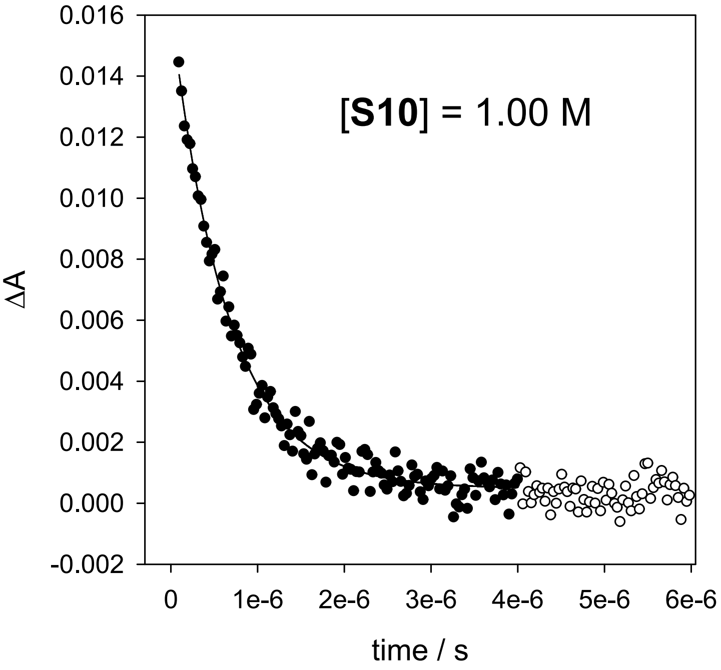

Figure SI.10-5. Plot of the change in absorbance (ΔA) monitored at 490 nm vs. time for the reaction of ε-caprolactam (**S10**) 1.00 M with CumO• measured in Ar-saturated MeCN solution at T = 25 °C. The white data points have been excluded from the nonlinear regression analysis.

Nonlinear Regression

Equation: Exponential Decay, Single, 3 Parameter
f=y0+a*exp(-b*x)

R	Rsqr	Adj Rsqr	Standard Error of Estimate
0.9917	0.9835	0.9832	0.0004

	Coefficient	Std. Error	t	P	VIF
y0	0.0005	6.4476E-005	7.5461	<0.0001	2.7732
a	0.0157	0.0002	64.0085	<0.0001	2.5951
b	1538360.5937	40746.9335	37.7540	<0.0001	4.7545

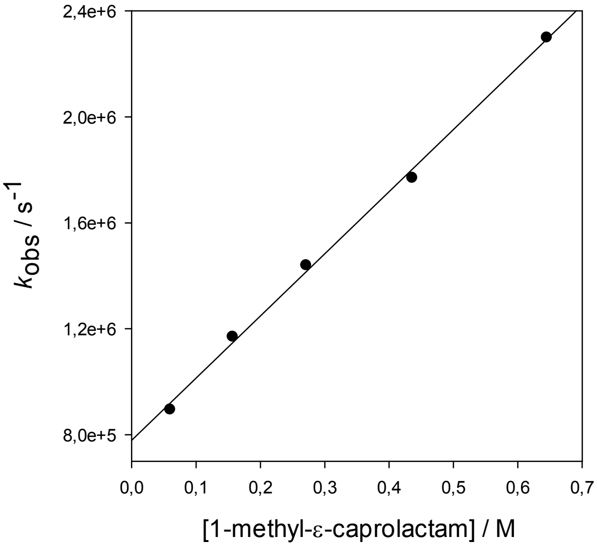

Figure SI.11. Plot of the observed rate constant (k_{obs}) against [substrate] for the reaction of 1-methyl-ε-caprolactam (**S11**) with CumO$^{\bullet}$ generated by 355 nm LFP of an Ar-saturated MeCN solution containing 1.0 M dicumyl peroxide measured at T = 25 °C following the decay of CumO$^{\bullet}$ at 490 nm. From the linear regression analysis: intercept = 7.79×10^5 s^{-1}, $k_H = 2.35 \times 10^6$ M^{-1} s^{-1}, $r^2 = 0.9976$.

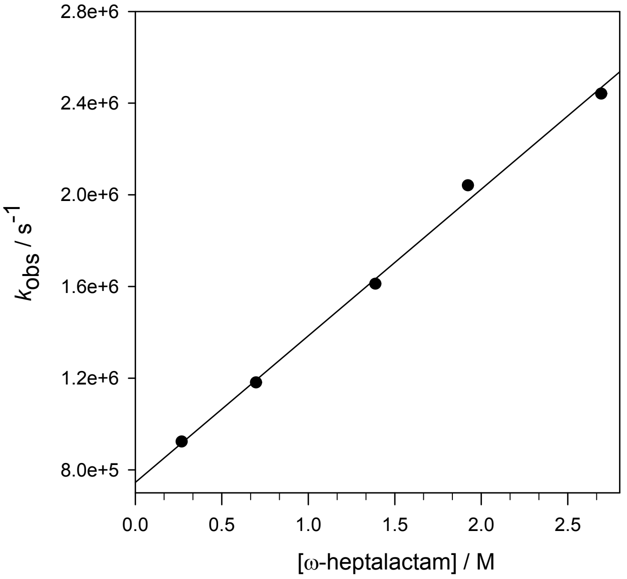

Figure SI.12. Plot of the observed rate constant (k_{obs}) against [substrate] for the reaction of ω-heptalactam (**S12**) with CumO$^{\bullet}$ generated by 355 nm LFP of an Ar-saturated MeCN solution containing 1.0 M dicumyl peroxide measured at T = 25 °C following the decay of CumO$^{\bullet}$ at 490 nm. From the linear regression analysis: intercept = 7.45×10^5 s^{-1}, $k_H = 6.38 \times 10^5$ M^{-1} s^{-1}, $r^2 = 0.9963$.

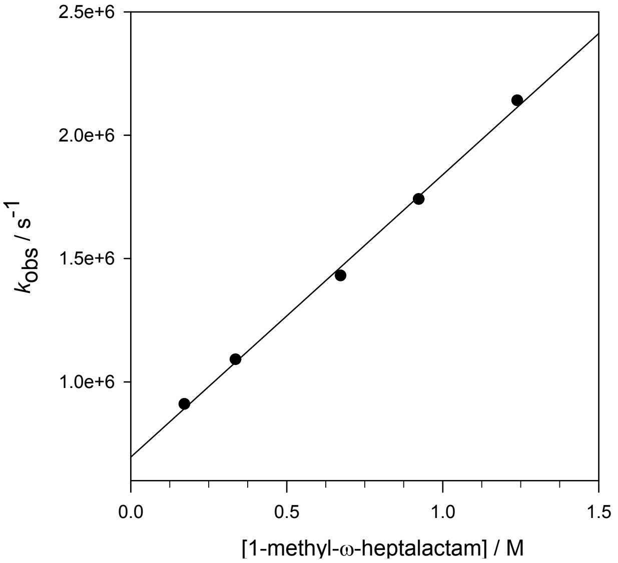

Figure SI.13. Plot of the observed rate constant (k_{obs}) against [substrate] for the reaction of 1-methyl-ω-heptalactam (**S13**) with CumO$^•$ generated by 355 nm LFP of an Ar-saturated MeCN solution containing 1.0 M dicumyl peroxide measured at T = 25 °C following the decay of CumO$^•$ at 490 nm. From the linear regression analysis: intercept = 6.95 × 10^5 s^{-1}, k_H = 1.14 × 10^6 M^{-1} s^{-1}, r^2 = 0.9975.

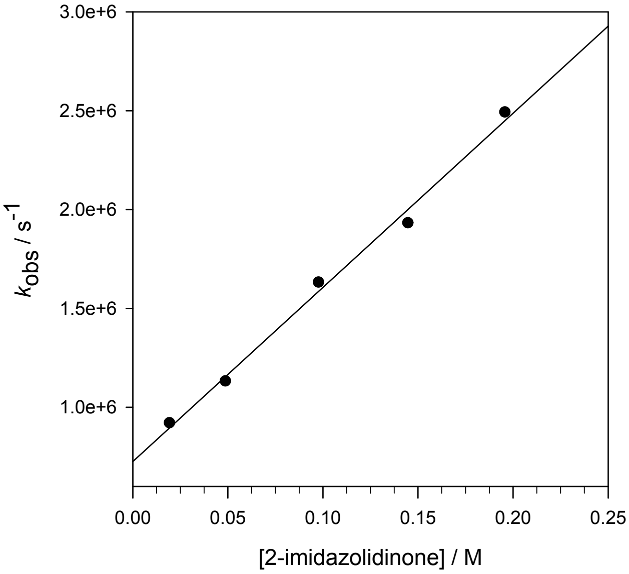

Figure SI.14A. Plot of the observed rate constant (k_{obs}) against [substrate] for the reaction of 2-imidazolidinone (**S14**) with CumO$^•$ generated by 355 nm LFP of an Ar-saturated MeCN solution containing 1.0 M dicumyl peroxide measured at T = 25 °C following the decay of CumO$^•$ at 490 nm. From the linear regression analysis: intercept = 7.26 × 10^5 s^{-1}, k_H = 8.81 × 10^6 M^{-1} s^{-1}, r^2 = 0.9939.

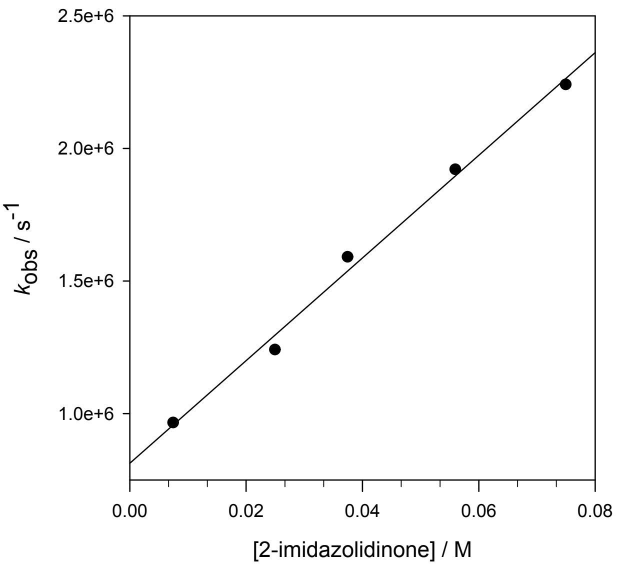

Figure SI.14B. Plot of the observed rate constant (k_{obs}) against [substrate] for the reaction of 2-imidazolidinone (**S14**) with $CumO^{\bullet}$ generated by 355 nm LFP of an Ar-saturated DMSO solution containing 1.0 M dicumyl peroxide measured at T = 25 °C following the decay of $CumO^{\bullet}$ at 490 nm. From the linear regression analysis: intercept = 8.13×10^{5} s^{-1}, k_H = 1.94×10^{7} M^{-1} s^{-1}, r^2 = 0.9933.

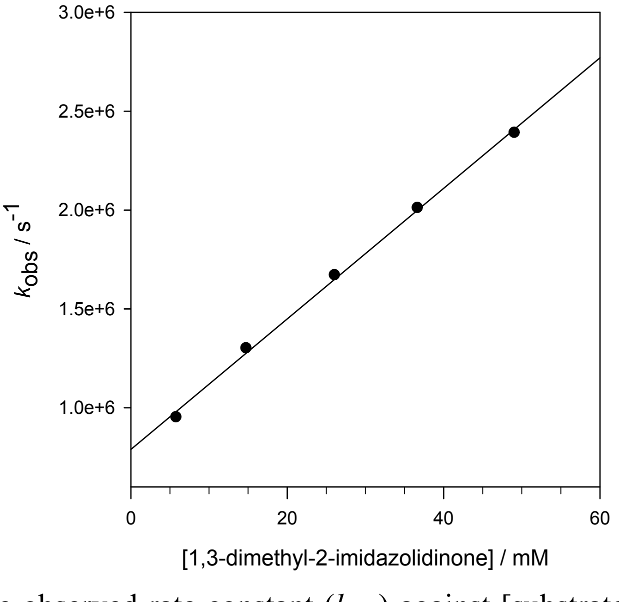

Figure SI.15A. Plot of the observed rate constant (k_{obs}) against [substrate] for the reaction of 1,3-dimethyl-2-imidazolidinone (**S15**) with $CumO^{\bullet}$ generated by 355 nm LFP of an Ar-saturated MeCN solution containing 1.0 M dicumyl peroxide measured at T = 25 °C following the decay of $CumO^{\bullet}$ at 490 nm. From the linear regression analysis: intercept = 7.89×10^{5} s^{-1}, k_H = 3.30×10^{7} M^{-1} s^{-1}, r^2 = 0.9982.

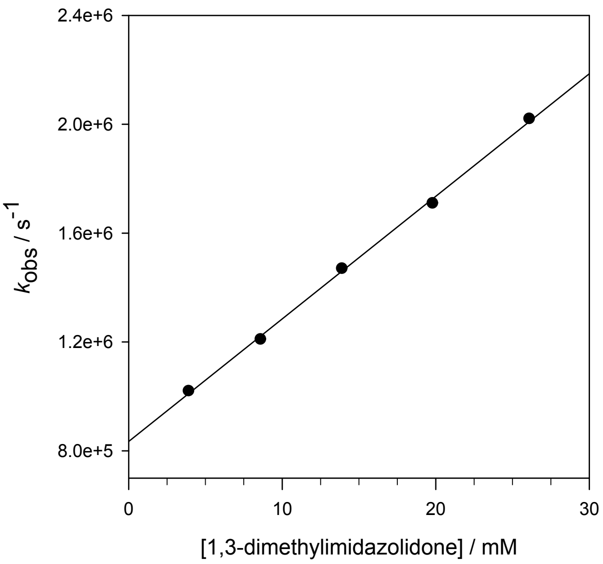

Figure SI.15B. Plot of the observed rate constant (k_{obs}) against [substrate] for the reaction of 1,3-dimethyl-2-imidazolidinone (**S15**) with CumO$^•$ generated by 355 nm LFP of an Ar-saturated DMSO solution containing 1.0 M dicumyl peroxide measured at T = 25 °C following the decay of CumO$^•$ at 490 nm. From the linear regression analysis: intercept = 8.35×10^5 s^{-1}, $k_H = 4.50 \times 10^7$ M^{-1} s^{-1}, $r^2 = 0.9989$.

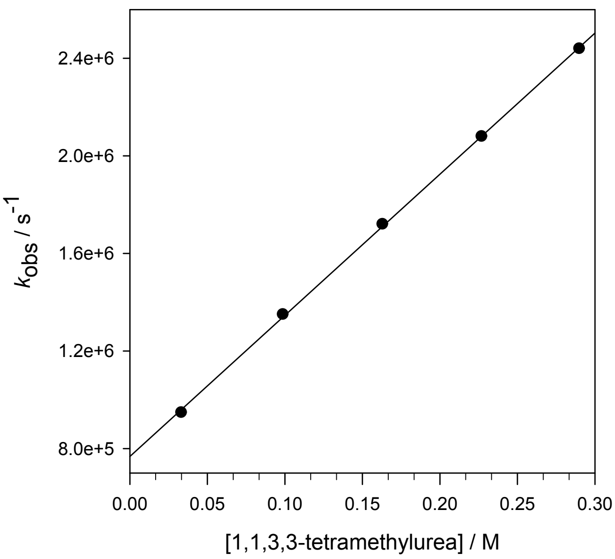

Figure SI.16. Plot of the observed rate constant (k_{obs}) against [substrate] for the reaction of 1,1,3,3-tetramethylurea (**S16**) with CumO$^•$ generated by 355 nm LFP of an Ar-saturated MeCN solution containing 1.0 M dicumyl peroxide measured at T = 25 °C following the decay of CumO$^•$ at 490 nm. From the linear regression analysis: intercept = 7.66×10^5 s^{-1}, $k_H = 5.79 \times 10^6$ M^{-1} s^{-1}, $r^2 = 0.9997$.

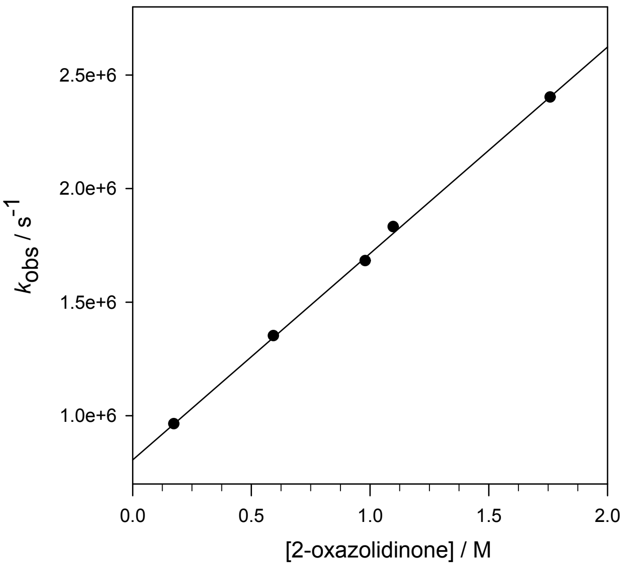

Figure SI.17. Plot of the observed rate constant (k_{obs}) against [substrate] for the reaction of 2-oxazolidinone (**S17**) with CumO$^•$ generated by 355 nm LFP of an Ar-saturated MeCN solution containing 1.0 M dicumyl peroxide measured at T = 25 °C following the decay of CumO$^•$ at 490 nm. From the linear regression analysis: intercept = 8.06 × 10^5 s^{-1}, k_H = 9.09 × 10^5 M^{-1} s^{-1}, r^2 = 0.9991.

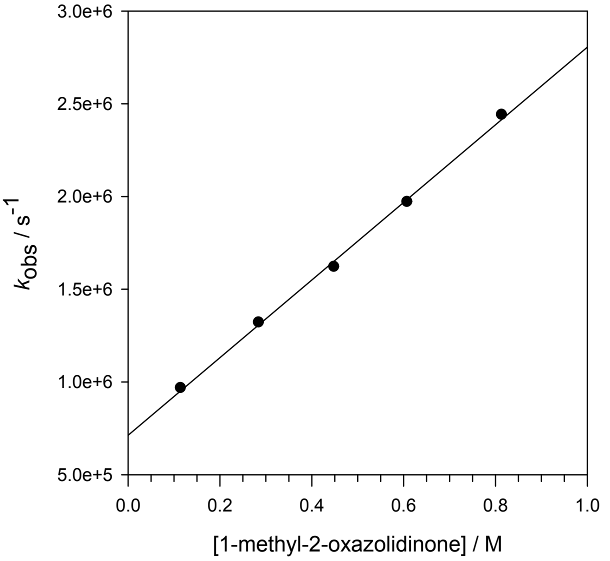

Figure SI.18. Plot of the observed rate constant (k_{obs}) against [substrate] for the reaction of 1-methyl-2-oxazolidinone (**S18**) with CumO$^•$ generated by 355 nm LFP of an Ar-saturated MeCN solution containing 1.0 M dicumyl peroxide measured at T = 25 °C following the decay of CumO$^•$ at 490 nm. From the linear regression analysis: intercept = 7.13 × 10^5 s^{-1}, k_H = 2.09 × 10^6 M^{-1} s^{-1}, r^2 = 0.9984.

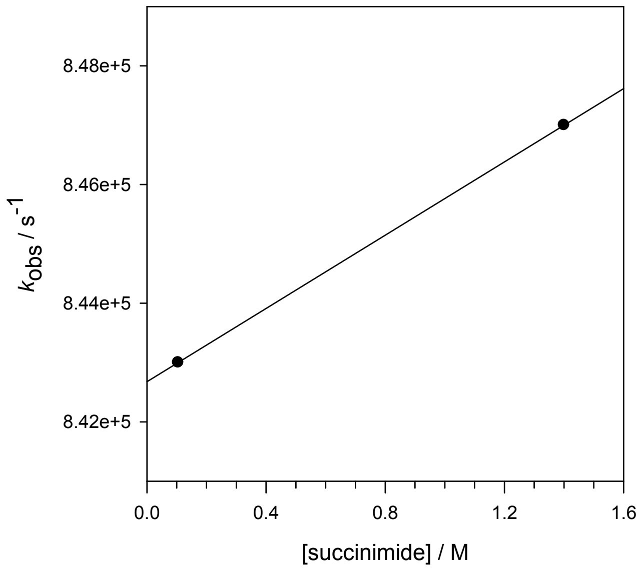

Figure SI.19. Plot of the observed rate constant (k_{obs}) against [substrate] for the reaction of succinimide (**S19**) with CumO$^\bullet$ generated by 355 nm LFP of an Ar-saturated DMSO solution containing 1.0 M dicumyl peroxide measured at T = 25 °C following the decay of CumO$^\bullet$ at 490 nm. From the linear regression analysis: intercept = 8.43×10^5 s^{-1}, $k_H = 3.09 \times 10^3$ M^{-1} s^{-1}, $r^2 = 1$. Only an upper limit was done: $k_H < 10^4$ M^{-1} s^{-1}.

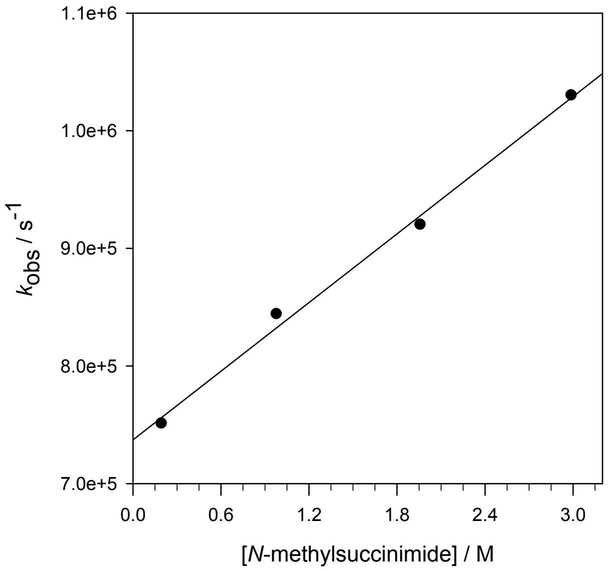

Figure SI.20. Plot of the observed rate constant (k_{obs}) against [substrate] for the reaction of *N*-methylsuccinimide (**S20**) with CumO$^\bullet$ generated by 355 nm LFP of an Ar-saturated MeCN solution containing 1.0 M dicumyl peroxide measured at T = 25 °C following the decay of CumO$^\bullet$ at 490 nm. From the linear regression analysis: intercept = 7.37×10^5 s^{-1}, $k_H = 9.73 \times 10^4$ M^{-1} s^{-1}, $r^2 = 0.9946$.

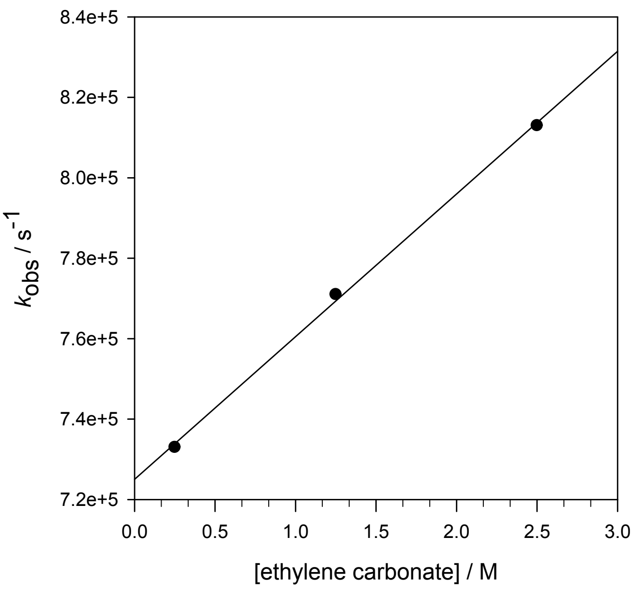

Figure SI.21. Plot of the observed rate constant (k_{obs}) against [substrate] for the reaction of ethylene carbonate (**S21**) with CumO$^•$ generated by 355 nm LFP of an Ar-saturated MeCN solution containing 1.0 M dicumyl peroxide measured at T = 25 °C following the decay of CumO$^•$ at 490 nm. From the linear regression analysis: intercept = 7.25×10^5 s^{-1}, $k_H = 3.55 \times 10^4$ M^{-1} s^{-1}, $r^2 = 0.9988$.

2.2) Primary kinetic data

Table SI.1: Primary kinetic data for the reaction of 2-azetidinone (**S1**) with CumO$^{\bullet}$ generated by 355 nm LFP in MeCN (**Figure SI.1**).

t / s	ΔA 2-azetidinone (S1)			
	**0.15M**	**0.563M**	**1.125M**	**1.5M**
-1E-06	0.00072	-0.0008	-0.0002	-0.0008
-1E-06	0.00132	-0.0009	9.5E-05	-0.0021
-9E-07	0.00066	-0.0007	-0.0012	-0.0011
-9E-07	0.00099	-0.0011	-0.0014	-0.0015
-9E-07	0.00052	-0.0015	-0.0007	-0.0014
-8E-07	-6E-05	-0.0003	-0.0003	-0.0019
-8E-07	0.00045	-0.0012	-0.0014	-0.0021
-8E-07	0.00035	-0.0006	-0.0004	-0.0017
-7E-07	0.00049	-0.0007	-0.0009	-0.0013
-7E-07	0.00064	-0.0015	-0.0012	-0.0023
-7E-07	-9E-05	-0.001	-0.0004	-0.0016
-6E-07	8E-05	-0.0017	-0.0014	-0.0019
-6E-07	0.00052	-0.0008	-0.0011	-0.002
-6E-07	-0.0002	-0.0009	-0.0004	-0.0013
-5E-07	-0.0006	-0.001	-0.0005	-0.0018
-5E-07	0.00069	-0.0006	-0.0011	-0.0013
-5E-07	0.00058	-0.0008	-0.0012	-0.0014
-4E-07	0.00032	-8E-05	-0.0008	-0.0014
-4E-07	-0.0002	0.00018	-0.0013	-0.0016
-4E-07	0.00076	-7E-05	-0.0003	-0.0009
-4E-07	0.00068	-0.0007	-0.0004	-0.0005
-3E-07	0.00084	-0.0004	-0.0006	-0.0014
-3E-07	0.00074	-0.0007	-0.0014	-0.0021
-3E-07	0.00059	-5E-06	-0.0006	-0.0028
-2E-07	0.00057	-0.0004	-6E-05	-0.0013
-2E-07	-9E-05	-0.0004	-0.0011	-0.0007
-2E-07	0.00014	-0.0003	-0.0006	-0.0021
-1E-07	-8E-05	-0.0004	-0.0002	-0.0009
-1E-07	-0.0003	-0.001	-0.0016	-0.001
-6E-08	0.00151	-0.0007	-0.0007	-0.0008
-3E-08	0.00092	-0.0002	-0.0011	-0.0015
-3E-10	-0.0024	-0.004	-0.0043	-0.0057
3.2E-08	-0.0107	-0.013	-0.0157	-0.0154
6.4E-08	0.01397	0.01475	0.01404	0.01337
9.6E-08	0.015	0.01693	0.01901	0.01952
1.3E-07	0.01519	0.01593	0.01771	0.01818
1.6E-07	0.01373	0.01508	0.01676	0.01657
1.9E-07	0.01396	0.01528	0.01544	0.01519
2.2E-07	0.01389	0.01549	0.01499	0.01459
2.6E-07	0.01343	0.01287	0.01412	0.01392
2.9E-07	0.01346	0.01372	0.01378	0.01261
3.2E-07	0.01372	0.01374	0.01281	0.01141
3.5E-07	0.01303	0.01252	0.01248	0.01125
3.8E-07	0.0117	0.01117	0.01219	0.01072
4.2E-07	0.01124	0.01194	0.01184	0.01026
4.5E-07	0.01218	0.01132	0.01165	0.01011
4.8E-07	0.01169	0.01035	0.01134	0.00954

5.1E-07	0.0112	0.00988	0.00996	0.008
5.4E-07	0.01062	0.01038	0.01002	0.00861
5.8E-07	0.01146	0.01007	0.01022	0.00788
6.1E-07	0.01065	0.00925	0.00985	0.00752
6.4E-07	0.01054	0.00887	0.00934	0.00869
6.7E-07	0.0101	0.00916	0.00887	0.00792
7E-07	0.00971	0.00868	0.00838	0.0074
7.4E-07	0.0099	0.00845	0.00796	0.00766
7.7E-07	0.00946	0.00829	0.00779	0.00771
8E-07	0.00917	0.00734	0.0079	0.00731
8.3E-07	0.00892	0.00753	0.00775	0.00646
8.6E-07	0.00841	0.00732	0.00757	0.00558
9E-07	0.0085	0.00708	0.00763	0.00617
9.3E-07	0.00814	0.00755	0.00651	0.00572
9.6E-07	0.00791	0.00698	0.00809	0.00635
9.9E-07	0.00836	0.00711	0.00725	0.00649
1E-06	0.00815	0.00759	0.00653	0.00488
1.1E-06	0.00801	0.00647	0.00633	0.00427
1.1E-06	0.00814	0.00618	0.00649	0.00495
1.1E-06	0.00724	0.00684	0.00615	0.00572
1.2E-06	0.00748	0.00718	0.00593	0.00539
1.2E-06	0.00743	0.00628	0.00576	0.00419
1.2E-06	0.00726	0.00549	0.00585	0.00515
1.2E-06	0.00679	0.00572	0.00447	0.0039
1.3E-06	0.00715	0.00587	0.00467	0.00355
1.3E-06	0.00623	0.00601	0.00603	0.00385
1.3E-06	0.00691	0.00533	0.0056	0.00461
1.4E-06	0.00645	0.00543	0.00541	0.00499
1.4E-06	0.00626	0.0053	0.0049	0.00424
1.4E-06	0.00615	0.00501	0.00554	0.00438
1.5E-06	0.00645	0.00555	0.0046	0.00357
1.5E-06	0.00625	0.00482	0.00523	0.00316
1.5E-06	0.00609	0.00456	0.00326	0.0034
1.6E-06	0.0059	0.00459	0.0048	0.00367
1.6E-06	0.00604	0.00342	0.00458	0.00293
1.6E-06	0.00617	0.0049	0.00474	0.00351
1.7E-06	0.00598	0.00518	0.00481	0.00284
1.7E-06	0.00562	0.00459	0.00361	0.00353
1.7E-06	0.0051	0.00454	0.00445	0.0019
1.8E-06	0.0052	0.00362	0.00428	0.00321
1.8E-06	0.00548	0.00352	0.00386	0.00326
1.8E-06	0.00536	0.00367	0.00396	0.00283
1.9E-06	0.00514	0.00361	0.00289	0.00229
1.9E-06	0.0047	0.00425	0.00284	0.00091
1.9E-06	0.00488	0.0037	0.00214	0.00291
2E-06	0.00463	0.00386	0.00337	0.00227
2E-06	0.00474	0.00364	0.00262	0.0024
2E-06	0.00456	0.00313	0.00346	0.00238
2E-06	0.00529	0.00419	0.00291	0.00247
2.1E-06	0.00494	0.00305	0.00229	0.00212
2.1E-06	0.00448	0.00332	0.00385	0.00225
2.1E-06	0.00442	0.00295	0.00313	0.00224
2.2E-06	0.00452	0.00292	0.00341	0.00231
2.2E-06	0.00417	0.00304	0.00257	0.00181

2.2E-06	0.00412	0.00264	0.00342	0.00139
2.3E-06	0.00428	0.00257	0.00309	0.00155
2.3E-06	0.0043	0.00331	0.00315	0.00055
2.3E-06	0.00385	0.00298	0.00312	0.00273
2.4E-06	0.00425	0.00199	0.0026	0.00216
2.4E-06	0.00412	0.00205	0.00292	0.00212
2.4E-06	0.00419	0.00287	0.00305	0.00139
2.5E-06	0.00353	0.00251	0.00257	0.00104
2.5E-06	0.00343	0.00324	0.00253	0.00254
2.5E-06	0.00472	0.00217	0.00213	0.00097
2.6E-06	0.00342	0.00155	0.00276	0.00147
2.6E-06	0.00416	0.00274	0.00314	0.00138
2.6E-06	0.00349	0.00289	0.00229	0.00048
2.7E-06	0.00345	0.00185	0.00284	0.002
2.7E-06	0.00327	0.00248	0.00171	0.00131
2.7E-06	0.00316	0.00166	0.00246	0.00127
2.8E-06	0.00258	0.00263	0.00257	0.00133
2.8E-06	0.0035	0.00264	0.00266	0.0007
2.8E-06	0.00386	0.00173	0.00253	0.00171
2.8E-06	0.00298	0.00236	0.00179	0.00084
2.9E-06	0.00307	0.00236	0.0022	0.00156
2.9E-06	0.00383	0.00246	0.00167	0.00111
2.9E-06	0.00296	0.00227	0.00243	0.00129
3E-06	0.00327	0.00134	0.00244	0.00184
3E-06	0.00306	0.00197	0.00114	0.00134
3E-06	0.00365	0.00231	0.00223	0.00162
3.1E-06	0.00299	0.00189	0.00317	0.00064
3.1E-06	0.0027	0.00122	0.00284	0.00209
3.1E-06	0.00269	0.00152	0.00198	0.00042
3.2E-06	0.00321	0.00158	0.0025	0.00145
3.2E-06	0.00241	0.00178	0.00124	0.00079
3.2E-06	0.00218	0.00221	0.00258	0.00011
3.3E-06	0.00191	0.00099	0.00236	0.00087
3.3E-06	0.00248	0.00156	0.00185	0.00081
3.3E-06	0.00268	0.00184	0.00239	0.00038
3.4E-06	0.00235	0.00132	0.00199	0.00063
3.4E-06	0.00244	0.00119	0.00236	0.00126
3.4E-06	0.00237	0.00158	0.00242	0.00062
3.5E-06	0.0034	0.00215	0.001	0.0011
3.5E-06	0.00257	0.00122	0.00147	0.00088
3.5E-06	0.00256	0.00155	0.00099	0.00099
3.6E-06	0.00254	0.00141	0.00113	0.00048
3.6E-06	0.00185	0.00043	0.00262	0.00075
3.6E-06	0.00204	0.00163	0.00154	0.00138
3.6E-06	0.00275	0.00174	0.00204	0.00023
3.7E-06	0.00224	0.00091	0.00239	0.00115
3.7E-06	0.00254	0.00089	0.00216	0.00141
3.7E-06	0.00218	0.00162	0.00156	0.00085
3.8E-06	0.00255	0.00089	0.00088	0.00135
3.8E-06	0.00195	0.00196	0.00104	0.00034
3.8E-06	0.00237	0.00206	0.00106	0.00033
3.9E-06	0.00201	0.0017	0.00032	0.00053
3.9E-06	0.00232	0.00074	0.00123	-3E-05
3.9E-06	0.00247	0.00124	0.0014	0.00134

4E-06	0.00208	0.00193	0.00117	-0.0002
4E-06	0.00269	0.00111	0.00035	0.00058
4E-06	0.00259	0.00144	0.00174	0.00077
4.1E-06	0.00254	0.00129	0.00163	-0.0002
4.1E-06	0.0018	0.00153	0.00098	4E-05
4.1E-06	0.00155	0.00104	0.00074	-0.0002
4.2E-06	0.00155	0.00054	0.00199	8E-05
4.2E-06	0.00159	0.00138	0.00218	0.00036
4.2E-06	0.00151	0.00028	0.00152	0.00036
4.3E-06	0.00193	0.00118	0.00102	-0.0003
4.3E-06	0.00175	0.001	0.0014	-5E-05
4.3E-06	0.00161	0.00057	0.00204	0.00015
4.4E-06	0.00101	0.00149	0.00109	-0.0001
4.4E-06	0.00134	0.00067	0.00073	-0.0003
4.4E-06	0.00163	0.00119	0.00104	-0.0002
4.4E-06	0.00177	0.00096	0.00135	0.00129
4.5E-06	0.00182	-1E-04	0.00166	0.00036
4.5E-06	0.00148	0.00053	0.00132	0.00054
4.5E-06	0.0017	0.00095	0.0013	-0.0005
4.6E-06	0.00145	0.00013	0.00106	0.00073
4.6E-06	0.00122	0.00081	0.00118	0.00166
4.6E-06	0.00149	0.00038	0.00124	0.00031
4.7E-06	0.00158	0.00073	0.00087	-0.0004
4.7E-06	0.00158	0.00099	0.00151	-0.001
4.7E-06	0.00105	0.0012	0.00072	0.00024
4.8E-06	0.00119	0.00082	0.00033	0.00064
4.8E-06	0.00158	0.00071	0.00106	0.00011
4.8E-06	0.0014	0.00086	0.00093	0.00051
4.9E-06	0.00142	0.00028	0.00039	0.00042
4.9E-06	0.00215	0.00068	0.00076	0.00051
4.9E-06	0.00147	0.00148	0.00101	-0.0005
5E-06	0.00127	0.00039	0.00158	0.00021
5E-06	0.00171	0.00097	0.00115	-6E-05
5E-06	0.00215	0.00091	0.00124	-0.0007
5.1E-06	0.00093	0.00096	0.00012	-0.0004
5.1E-06	0.00117	0.00123	0.0002	0.00093
5.1E-06	0.001	0.00061	0.00059	0.00027
5.2E-06	0.00232	0.00103	0.00111	-3E-05
5.2E-06	0.001	0.00036	0.00028	0.0003
5.2E-06	0.00154	0.00012	0.00041	-0.0001
5.2E-06	0.00193	0.00039	0.00127	4.6E-05
5.3E-06	0.00126	0.00165	0.0012	0.00016
5.3E-06	0.00201	0.00104	0.00143	-1E-04
5.3E-06	0.00134	0.00033	0.00099	0.0002
5.4E-06	0.0014	0.00065	0.00097	4.6E-05
5.4E-06	0.00107	-0.0007	0.00149	0.00035
5.4E-06	0.00048	0.00053	0.00109	-0.0003
5.5E-06	0.00152	0.001	0.00071	-0.0002
5.5E-06	0.00094	0.00142	0.00164	0.00048
5.5E-06	0.00118	0.00052	0.0002	0.00034
5.6E-06	0.00121	0.00023	0.00029	1.1E-05
5.6E-06	0.00156	0.00114	0.00134	0.00066
5.6E-06	0.00174	2.2E-05	0.00133	-0.0003
5.7E-06	0.00162	0.00041	0.00055	0.0005

5.7E-06	0.00182	0.0001	0.00106	-9E-05
5.7E-06	0.00147	0.00155	-9E-05	-0.0005
5.8E-06	0.00091	0.00073	0.00021	-0.0005
5.8E-06	0.00167	0.00041	0.00074	-0.0009
5.8E-06	0.00172	-0.0007	0.00011	0.00018
5.9E-06	0.00127	0.00048	0.0011	0.00127
5.9E-06	0.00177	0.00047	0.00057	0.00057
5.9E-06	0.00151	2.4E-05	0.00067	0.00059
6E-06	0.00174	0.00096	-0.0003	2.5E-05
6E-06	0.00089	0.0005	0.00081	0.00012
6E-06	0.00127	0.00024	0.00144	0.00022
6E-06	0.00106	0.00052	0.00015	0.00012
6.1E-06	0.00094	0.00067	0.00038	0.00041
6.1E-06	0.00178	0.0011	0.00044	6.3E-05
6.1E-06	0.00151	0.00019	0.00052	0.00086
6.2E-06	0.00117	0.0004	0.00073	0.00018
6.2E-06	0.00123	0.00051	0.00134	0.00024
6.2E-06	0.00134	-0.0001	0.00118	0.00025
6.3E-06	0.00188	0.00048	0.00084	-0.0007
6.3E-06	0.00191	0.00097	0.00145	-7E-05
6.3E-06	0.00182	0.00017	0.00133	-0.0011
6.4E-06	0.00178	0.00062	0.00033	0.00026
6.4E-06	0.00125	-0.0001	0.00018	-0.0002
6.4E-06	0.00095	-0.0005	0.00055	-4E-05
6.5E-06	0.00149	0.00041	0.00075	0.00032
6.5E-06	0.00159	0.00077	0.00121	-0.0004
6.5E-06	0.00172	9.4E-05	0.00042	0.0001
6.6E-06	0.00159	0.00044	0.00068	-0.0002
6.6E-06	0.0016	0.00051	0.00124	-0.0007
6.6E-06	0.00127	0.00088	-0.0003	0.00019
6.7E-06	0.00137	0.00026	0.00138	9.5E-05
6.7E-06	0.00143	-0.0004	0.00039	-0.0008
6.7E-06	0.00115	-8E-05	0.00113	1.1E-05
6.8E-06	0.00152	-0.0006	0.00039	-0.0003
6.8E-06	0.0013	0.00078	0.00152	0.00047
6.8E-06	0.00109	0.00075	-0.0001	0.0001
6.8E-06	0.00125	0.00089	0.00075	0.00013
6.9E-06	0.00136	0.00129	-0.0001	0.00081
6.9E-06	0.00182	0.00044	-7E-05	0.00056
6.9E-06	0.00172	0.00043	0.00011	0.00088
7E-06	0.00094	0.00086	0.00096	0.0007
7E-06	0.00134	-0.0002	0.00023	0.0005
7E-06	0.00147	0.00035	-0.0005	-0.0003
7.1E-06	0.00166	0.00067	-0.0009	-0.001
7.1E-06	0.00118	-1E-05	0.00057	0.00074
7.1E-06	0.002	0.00045	0.00032	-0.0003
7.2E-06	0.0018	0.00128	0.00056	0.00083
7.2E-06	0.00079	0.00099	0.00109	0.00027
7.2E-06	0.00158	-0.0001	0.00039	0.00036
7.3E-06	0.00184	0.00084	0.00123	0.00016
7.3E-06	0.00127	-0.0001	0.00023	8.5E-05
7.3E-06	0.0015	0.00047	0.00063	0.00039
7.4E-06	0.00219	0.0006	0.00099	0.00072
7.4E-06	0.00099	0.00073	0.00036	0.00028

7.4E-06	0.00107	0.00063	0.00147	-0.0002
7.5E-06	0.00164	-0.0002	0.00023	0.00054
7.5E-06	0.00181	-0.0003	0.00097	-0.0002
7.5E-06	0.00131	0.00073	0.00053	0.00043
7.6E-06	0.00168	-0.0004	-5E-05	0.00051
7.6E-06	0.00219	-9E-05	0.00074	0.00025
7.6E-06	0.00062	0.0005	0.00045	-0.0001
7.6E-06	0.00127	0.00013	0.00084	-0.0004
7.7E-06	0.001	0.00048	0.00047	0.00035
7.7E-06	0.002	0.00035	-0.0001	-0.0004
7.7E-06	0.00111	3.8E-05	0.00103	0.00084
7.8E-06	0.00163	-0.0004	0.00032	-0.0007
7.8E-06	0.00134	0.00078	0.00071	-0.0009
7.8E-06	0.00115	8.5E-05	0.00156	-0.0005
7.9E-06	0.00115	0.00071	0.00029	-0.0011
7.9E-06	0.00135	0.00035	0.00023	0.00098
7.9E-06	0.00154	5.3E-06	-8E-05	-0.0008
8E-06	0.00076	-3E-05	0.00049	-0.0003
8E-06	0.00121	0.0006	0.00101	-0.0001
8E-06	0.00072	0.00112	0.00078	-0.0007
8.1E-06	0.00136	0.0008	0.0005	-0.0005
8.1E-06	0.00092	0.00026	0.00078	-1E-05
8.1E-06	0.00122	-0.0003	-4E-05	-0.0009
8.2E-06	0.00106	-6E-05	-0.0004	-0.0009
8.2E-06	0.00129	0.00069	-0.0002	-3E-05
8.2E-06	0.00136	0.00049	0.00067	0.0001
8.3E-06	0.00113	0.00021	-0.0002	-0.0004
8.3E-06	0.00154	-0.0002	0.00069	0.00017
8.3E-06	0.00137	0.00033	-0.0001	-0.0005
8.4E-06	0.00127	0.00041	-0.0007	-3E-06
8.4E-06	0.00085	-0.0003	-0.0001	-0.0005
8.4E-06	0.00109	-0.0007	0.00127	0.00022
8.4E-06	0.00052	8.6E-05	0.00093	-0.0002
8.5E-06	0.00098	9.9E-05	9.3E-05	-0.0006
8.5E-06	0.00136	0.00049	0.00033	0.0005
8.5E-06	0.00074	0.00023	4.5E-05	0.00061
8.6E-06	0.00109	0.00035	0.00135	0.00028
8.6E-06	0.00115	3.4E-05	0.00127	0.00119
8.6E-06	0.00056	0.00057	0.00111	0.0007
8.7E-06	0.00107	-0.0002	0.00126	4.9E-06
8.7E-06	0.00081	0.00038	0.0009	0.00069
8.7E-06	0.0008	-0.0001	0.00102	-0.0001
8.8E-06	0.00138	0.00043	-0.0002	-0.0003
8.8E-06	0.0016	-0.0002	0.00027	-7E-05
8.8E-06	0.00185	0.00014	0.00088	0.00019
8.9E-06	0.00107	0.00044	0.00038	0.00022
8.9E-06	0.00106	0.00024	0.00122	0.00064
8.9E-06	0.00079	0.00117	0.00069	-8E-05
9E-06	0.00094	0.00064	0.00087	0.00052
9E-06	0.00126	0.00039	0.00105	-0.0004
9E-06	0.00094	0.00044	0.00132	-0.0003
9.1E-06	0.00094	0.00032	0.00103	-0.0003
9.1E-06	0.0012	0.00075	0.00036	0.00033
9.1E-06	0.00091	0.00012	0.00112	0.00027

9.2E-06	0.00115	1.4E-06	-4E-05	-0.0002
9.2E-06	0.00115	-1E-04	0.00018	0.00036
9.2E-06	0.00104	0.00019	0.00131	0.0001
9.2E-06	0.00137	0.00125	0.00047	-0.0006
9.3E-06	0.00095	0.00029	0.00109	0.0002
9.3E-06	0.0019	0.00053	0.00059	0.00033
9.3E-06	0.00101	-0.0003	0.00035	0.0002
9.4E-06	0.00162	-0.0001	0.0011	7.4E-05
9.4E-06	0.00079	-0.0002	0.00039	0.0004
9.4E-06	0.00147	0.00098	0.00061	0.00049
9.5E-06	0.00135	0.0004	-2E-05	-9E-05
9.5E-06	0.00177	0.00113	-0.0001	0.0009
9.5E-06	0.00097	0.00033	0.00077	0.00123
9.6E-06	0.00129	2.7E-05	0.00047	0.00056
9.6E-06	0.00124	-0.0003	0.00075	-0.0003
9.6E-06	0.001	0.00029	0.00117	0.00018
9.7E-06	0.00088	0.0004	0.00014	0.00146
9.7E-06	0.00135	6.1E-05	0.00017	0.00059
9.7E-06	0.00123	0.00087	0.00076	0.00094
9.8E-06	0.001	0.0006	0.00141	0.00125
9.8E-06	0.00174	-0.0004	0.00091	0.00011
9.8E-06	0.00088	0.00037	0.00136	0.00098
9.9E-06	0.00145	-0.0006	0.00077	-0.0003
9.9E-06	0.00121	0.0002	0.00067	0.00148
9.9E-06	0.00145	0.00036	0.0013	0.00032
1E-05	0.00122	0.0006	0.00091	0.00125
1E-05	0.00152	0.00095	0.00074	7.1E-05
1E-05	0.00159	0.00055	-0.0003	2.8E-06
1E-05	0.0019	0.00128	-0.0002	-0.0004
1E-05	0.00111	3.5E-05	0.00055	0.0002
1E-05	0.00167	0.00075	0.00024	-0.0011
1E-05	0.00156	-6E-06	0.00065	-0.0004
1E-05	0.00137	0.00036	0.00056	0.00017
1E-05	0.00181	5.3E-07	0.00076	0.00045
1E-05	0.00165	7.4E-05	0.00115	0.00011
1E-05	0.00114	0.00046	0.00033	-0.0001
1E-05	0.001	0.0006	0.00036	-0.0006
1E-05	0.00087	-0.0003	0.00047	0.00024
1E-05	0.00145	0.0002	-5E-05	-0.001
1E-05	0.00111	0.00024	0.00137	-8E-05
1E-05	0.00085	-0.0002	0.00079	-0.0004
1E-05	0.00108	-6E-05	0.00146	-0.0001
1.1E-05	0.00179	0.00073	7.1E-05	0.0001
1.1E-05	0.00082	-0.0001	0.00102	-0.0006
1.1E-05	0.00098	0.00159	0.00019	0.0006
1.1E-05	0.00111	0.00107	0.00093	0.00034
1.1E-05	0.00124	0.00097	0.00094	0.00051
1.1E-05	0.002	2.8E-06	-0.0002	8.7E-05
1.1E-05	0.00147	0.00044	0.00113	-0.0003
1.1E-05	0.00117	0.00025	0.00133	-0.0006
1.1E-05	0.00087	0.00011	0.00144	-0.0002
1.1E-05	0.00157	0.00053	0.0005	-0.0001
1.1E-05	0.00111	6.6E-05	0.00037	0.00018
1.1E-05	0.00083	0.00018	0.00173	-0.0007

1.1E-05	0.00112	0.00016	0.00069	0.00052
1.1E-05	0.00134	0.00039	0.00029	0.0006
1.1E-05	0.00071	0.00052	0.00121	8E-05
1.1E-05	0.00082	0.0005	0.00086	0.00011
1.1E-05	0.00083	0.00099	0.0004	-0.0001
1.1E-05	0.00118	0.00063	0.00035	0.00016
1.1E-05	0.00097	0.00101	0.00061	-0.0002
1.1E-05	0.00139	-0.0001	0.00116	0.00109
1.1E-05	0.00169	0.00069	0.00035	-0.0004
1.1E-05	0.00153	0.00013	0.00142	0.00049
1.1E-05	0.00112	-4E-05	0.0011	-0.001
1.1E-05	0.0019	-9E-05	0.00046	-0.0002
1.1E-05	0.00184	0.00047	0.00046	0.00014
1.1E-05	0.00178	0.0004	-3E-05	-0.0004
1.1E-05	0.00121	0.00018	0.00118	-0.0002
1.1E-05	0.00108	0.00077	0.00062	0.00013
1.1E-05	0.00141	-8E-05	0.00197	-0.0004
1.1E-05	0.00096	-0.0003	0.00041	0.00032
1.1E-05	0.00091	0.00058	0.00065	0.00033
1.1E-05	0.00118	0.00075	-0.0001	-0.0004
1.2E-05	0.00115	0.0008	0.00079	-0.0005
1.2E-05	0.0015	0.00074	0.00067	-0.0002
1.2E-05	0.00111	0.00098	0.00032	0.00108
1.2E-05	0.00128	0.00077	0.00038	-0.0004
1.2E-05	0.00127	6.9E-05	0.0006	0.00038
1.2E-05	0.00146	-0.0004	0.00021	0.00026
1.2E-05	0.00074	0.0007	0.00027	0.00059
1.2E-05	0.00159	0.00079	0.00016	0.00048
1.2E-05	0.001	0.00046	0.00018	0.00036
1.2E-05	0.00145	0.00029	0.00102	-4E-05
1.2E-05	0.00148	0.00059	-0.0001	4.7E-06
1.2E-05	0.0013	-0.0003	0.0006	-0.0007
1.2E-05	0.00168	0.00073	0.00045	0.00032
1.2E-05	0.00129	0.00034	0.00028	-4E-05
1.2E-05	0.0011	0.00077	0.00068	0.00086
1.2E-05	0.00166	0.00061	0.00053	0.00059
1.2E-05	0.00246	0.0004	0.00033	-0.0006
1.2E-05	0.00116	-3E-05	0.00157	0.00039
1.2E-05	0.00132	0.00048	0.00083	-0.0003
1.2E-05	0.00127	0.00017	9.6E-05	0.00094
1.2E-05	0.00171	-5E-06	0.00038	-0.0003
1.2E-05	0.00072	-0.0002	-0.0001	0.00072
1.2E-05	0.00142	0.00035	0.00047	6.3E-05
1.2E-05	0.00129	0.0011	0.00119	0.00011
1.2E-05	0.00139	0.00063	0.00031	-0.0003
1.2E-05	0.00105	0.00059	0.00015	0.0003
1.2E-05	0.00151	-0.0007	-2E-05	0.00043
1.2E-05	0.00063	0.00037	0.00032	0.00013
1.2E-05	0.00148	0.00108	0.00075	0.00059
1.2E-05	0.00089	-0.0003	0.00074	-0.0007
1.2E-05	0.00256	0.00033	0.00081	-0.0006
1.3E-05	0.00173	0.0004	0.0002	0.00033
1.3E-05	0.00163	0.00035	0.00105	-0.0002
1.3E-05	0.00119	0.00037	0.00065	0.00014

1.3E-05	0.00086	0.00053	0.00017	0.00033
1.3E-05	0.0017	0.00053	0.00081	-8E-05
1.3E-05	0.0012	-0.0001	0.00055	0.00015
1.3E-05	0.00115	0.00058	0.00094	-0.0001
1.3E-05	0.00116	0.00064	0.00143	-0.0007
1.3E-05	0.00122	0.00097	0.0005	0.00045
1.3E-05	0.00166	0.00063	0.00037	-0.0008
1.3E-05	0.00186	0.00052	0.00115	2.4E-05
1.3E-05	0.00112	0.00078	0.00063	0.0003
1.3E-05	0.00162	8.3E-05	-2E-05	0.00025
1.3E-05	0.00121	2.7E-05	0.00161	0.00024
1.3E-05	0.00126	0.00065	-0.0003	0.00029
1.3E-05	0.00168	0.0004	0.00055	0.00054
1.3E-05	0.00138	0.00059	0.00023	-0.0007
1.3E-05	0.00206	0.00071	2.2E-05	-0.0001
1.3E-05	0.00125	0.00061	0.00026	-7E-05
1.3E-05	0.0014	0.00083	0.00012	-0.0002
1.3E-05	0.0011	0.00033	0.00161	-0.0003
1.3E-05	0.00145	0.00071	0.001	-0.0004
1.3E-05	0.00122	0.00067	0.00069	0.00017
1.3E-05	0.00167	0.00042	0.00084	9.5E-05
1.3E-05	0.00167	-0.0004	0.00041	-0.0005
1.3E-05	0.00161	0.001	0.0007	0.00015
1.3E-05	0.00146	0.00119	0.00097	-0.0006
1.3E-05	0.00145	7.5E-05	0.00054	-0.0005
1.3E-05	0.0008	0.00039	0.0006	-0.0003
1.3E-05	0.00123	0.0005	0.00136	9.3E-05
1.3E-05	0.00119	0.00113	0.00112	0.00012
1.4E-05	0.00069	0.00043	0.00055	-0.0005
1.4E-05	0.0013	0.00034	0.00032	-0.0002
1.4E-05	0.00129	0.00098	0.00073	-0.0005
1.4E-05	0.0005	0.00043	0.00023	-4E-05
1.4E-05	0.00127	0.00081	0.00109	-0.0002
1.4E-05	0.00108	0.0002	0.00056	-0.0007
1.4E-05	0.00194	2.3E-05	0.00082	-0.0002
1.4E-05	0.00104	0.0006	0.00096	0.0012
1.4E-05	0.00128	5.4E-05	0.001	0.00088
1.4E-05	0.00177	0.00045	0.00063	0.00035
1.4E-05	0.00171	0.00084	0.00067	-0.0005
1.4E-05	1.8E-05	0.0005	0.00036	-0.0004
1.4E-05	0.00123	-8E-05	0.00141	0.00036
1.4E-05	0.00174	0.00087	0.00062	-0.0008
1.4E-05	0.00118	0.00056	0.00012	-0.0005
1.4E-05	0.00127	0.00118	0.0009	-0.0009
1.4E-05	0.00054	0.00078	0.00125	-0.0004
1.4E-05	0.00029	0.00115	0.00083	0.00031
1.4E-05	0.00048	0.0012	0.0011	0.00054
1.4E-05	0.00172	0.00067	0.00186	-0.0002
1.4E-05	0.00163	0.00045	0.00063	-0.0002
1.4E-05	0.00116	0.00093	0.00121	0.00042
1.4E-05	0.00062	0.0003	0.00013	-5E-05
1.4E-05	0.00102	0.00052	0.00083	-0.0002
1.4E-05	0.00202	0.00076	0.00142	0.00026
1.4E-05	0.00039	0.0006	0.00218	0.00038

1.4E-05	0.00066	0.00064	0.00072	0.0002
1.4E-05	0.00146	0.00075	8.4E-05	-0.0004
1.4E-05	0.00153	0.00047	0.0011	0.00065
1.4E-05	0.0008	0.00075	0.00126	-0.0001
1.4E-05	0.00069	0.00112	0.00066	0.00022
1.5E-05	0.0014	0.00048	0.0013	0.00029
1.5E-05	0.00119	0.00125	0.00087	0.00043
1.5E-05	0.00125	0.00083	0.00097	0.00048
1.5E-05	0.00097	0.00039	0.00047	0.00039
1.5E-05	0.00163	0.00024	0.00076	-0.0004
1.5E-05	0.00194	-0.0002	0.00102	0.0001
1.5E-05	0.00098	0.00058	0.00134	-0.0002
1.5E-05	0.00179	0.00023	0.00098	0.00017
1.5E-05	0.00181	0.0002	0.00136	-0.0007
1.5E-05	0.00161	-0.0004	0.00094	0.00019
1.5E-05	0.00155	0.00051	0.00021	0.00066
1.5E-05	0.00119	0.0003	0.0006	0.00056
1.5E-05	0.00093	0.00054	0.00087	0.00018
1.5E-05	0.0009	0.00078	-3E-05	-0.0004
1.5E-05	0.002	0.00016	0.00086	-0.0005
1.5E-05	0.00117	0.00062	0.00062	-0.0003

Table SI.2A: Primary kinetic data for the reaction of 2-pyrrolidone (**S2**) with CumO$^{\bullet}$ generated by 355 nm LFP in MeCN (**Figure SI.2A**).

**t / s**	**ΔA 2-pyrrolidinone (S2)**				
	**0.130M**	**0.258M**	**0.445M**	**0.627M**	**0.803M**
-1E-06	0.00066	0.00066	-7E-05	0.00045	-0.0008
-1E-06	-0.0008	0.00068	8.3E-05	-0.0002	-0.0005
-9E-07	-0.0005	0.00032	-0.0007	0.00027	6.5E-05
-9E-07	-0.0004	0.00088	-0.0005	-0.0003	-0.0002
-9E-07	0.00017	0.00118	-0.0009	-2E-05	-0.0004
-8E-07	-0.0006	0.00023	-0.0006	-0.0005	0.00018
-8E-07	-0.0011	0.00054	-0.0005	-2E-05	-0.0002
-8E-07	-0.0005	0.001	9.3E-05	0.0006	5E-05
-7E-07	-1E-05	0.00067	-0.0004	2.8E-05	-0.0007
-7E-07	-0.0012	0.00033	-0.0011	-0.0004	-0.0003
-7E-07	-0.0007	0.00101	-0.0009	-3E-05	-0.0002
-6E-07	-0.0006	0.00101	-0.0005	-0.0002	-0.0006
-6E-07	-0.0012	0.00087	-0.002	-0.0002	-0.001
-6E-07	-0.0004	0.00107	-0.001	0.00035	-0.0001
-5E-07	0.00058	0.00089	-0.0008	-8E-05	0.00013
-5E-07	-0.0003	0.00056	-0.0011	0.00045	-0.0006
-5E-07	-0.0006	0.00126	-0.0012	0.00067	-0.0008
-4E-07	-0.0004	0.00076	-0.0003	-9E-05	-0.0004
-4E-07	-0.0005	0.0003	-0.0011	-0.0005	0.00037
-4E-07	-0.0001	0.00101	-0.0007	-0.0001	-0.0005
-4E-07	-0.0007	0.00091	-0.0008	0.0003	-0.0001
-3E-07	0.00031	0.00068	-0.0007	0.00027	-0.0007
-3E-07	-1E-05	0.00111	-0.0003	-0.0001	-0.0005
-3E-07	-0.0005	0.0005	-0.0008	-0.0006	-0.0005
-2E-07	-0.0001	-0.0001	-0.0002	-0.0004	0.00011
-2E-07	0.00025	0.00066	-0.0007	-0.0003	-0.0002
-2E-07	-8E-05	0.00117	-0.0006	-7E-05	-0.0005
-1E-07	-0.0004	0.00108	-0.0006	-0.0001	-0.0004
-1E-07	-0.001	0.00089	-0.0005	1.2E-06	-0.0007
-6E-08	-0.0009	0.00094	-0.001	-0.0004	-0.0008
-3E-08	-0.0003	0.00099	-0.0004	-0.0002	-0.001
-2E-10	-0.0032	-0.003	-0.0049	-0.003	-0.003
3.2E-08	-0.0131	-0.0175	-0.0211	-0.0189	-0.0169
6.4E-08	0.01042	0.01026	0.00779	0.00642	0.00443
9.6E-08	0.01463	0.01687	0.0148	0.01442	0.01457
1.3E-07	0.01519	0.01627	0.01397	0.01309	0.01325
1.6E-07	0.01405	0.01516	0.01325	0.01194	0.01235
1.9E-07	0.01363	0.01466	0.0118	0.01146	0.01083
2.2E-07	0.01404	0.01475	0.01197	0.0111	0.01016
2.6E-07	0.01293	0.01406	0.01125	0.01035	0.01023
2.9E-07	0.01201	0.0129	0.01134	0.00983	0.00932
3.2E-07	0.01294	0.01243	0.01046	0.00853	0.00816
3.5E-07	0.01206	0.0113	0.00945	0.00816	0.00714
3.8E-07	0.01137	0.01112	0.00867	0.00829	0.00673
4.2E-07	0.01099	0.0109	0.00855	0.00705	0.00677
4.5E-07	0.0111	0.01102	0.00813	0.00657	0.00644
4.8E-07	0.00943	0.00998	0.00735	0.00682	0.00599
5.1E-07	0.00989	0.01023	0.00727	0.00591	0.00537
5.4E-07	0.00964	0.00976	0.00665	0.00538	0.0047

5.8E-07	0.00929	0.00962	0.007	0.00504	0.00373
6.1E-07	0.0086	0.00917	0.00596	0.00477	0.00399
6.4E-07	0.00801	0.00895	0.00602	0.00418	0.00347
6.7E-07	0.00779	0.00876	0.00573	0.00459	0.00351
7E-07	0.00789	0.0078	0.00383	0.0041	0.00353
7.4E-07	0.00798	0.00808	0.00471	0.00445	0.00326
7.7E-07	0.00777	0.00733	0.00522	0.0044	0.00229
8E-07	0.00783	0.00752	0.00449	0.00315	0.00269
8.3E-07	0.00703	0.00687	0.00381	0.00296	0.00192
8.6E-07	0.00641	0.0072	0.0037	0.00261	0.00212
9E-07	0.00579	0.00666	0.00323	0.003	0.00202
9.3E-07	0.0064	0.00674	0.004	0.00281	0.00177
9.6E-07	0.00537	0.00521	0.00394	0.00323	0.00221
9.9E-07	0.00458	0.00616	0.00336	0.00252	0.00107
1E-06	0.0056	0.00653	0.00277	0.00265	0.00125
1.1E-06	0.00567	0.00644	0.00242	0.00266	0.00112
1.1E-06	0.00499	0.00545	0.00296	0.00178	0.00088
1.1E-06	0.00549	0.0059	0.00249	0.00163	0.00166
1.2E-06	0.00479	0.00545	0.00251	0.00194	0.001
1.2E-06	0.00447	0.00495	0.00192	0.00156	0.00164
1.2E-06	0.00429	0.00513	0.0015	0.00116	0.00107
1.2E-06	0.00478	0.00436	0.0016	0.00219	0.00112
1.3E-06	0.00423	0.00376	0.00177	0.0022	0.00049
1.3E-06	0.00396	0.00513	0.0014	0.00213	0.00039
1.3E-06	0.00429	0.00471	0.00126	0.00154	0.00092
1.4E-06	0.00408	0.00511	0.00107	0.00108	0.00121
1.4E-06	0.00381	0.00444	0.00152	0.00098	0.00134
1.4E-06	0.00303	0.00439	0.00127	0.00071	0.00132
1.5E-06	0.00465	0.00403	0.00134	0.00105	0.00044
1.5E-06	0.00365	0.00408	0.0014	0.0012	0.00093
1.5E-06	0.00279	0.0044	0.00164	0.00088	0.00099
1.6E-06	0.00305	0.00399	0.00134	0.00081	0.00042
1.6E-06	0.00317	0.00391	0.0004	0.00157	0.00055
1.6E-06	0.00296	0.00374	0.00096	0.00101	0.00034
1.7E-06	0.00321	0.00322	0.0014	0.00097	0.00031
1.7E-06	0.00258	0.00308	0.00077	0.00048	0.00051
1.7E-06	0.00291	0.00279	0.00119	0.00061	0.00072
1.8E-06	0.00288	0.00229	0.00041	0.00063	0.00053
1.8E-06	0.00233	0.00354	0.00043	0.00082	-0.0003
1.8E-06	0.00304	0.00308	0.00102	0.00055	0.00032
1.9E-06	0.00228	0.00318	3.5E-05	0.00071	5.7E-06
1.9E-06	0.00272	0.00307	0.00043	0.00055	0.00053
1.9E-06	0.00262	0.00286	0.00056	0.00081	0.00047
2E-06	0.00259	0.00322	0.00121	0.0007	0.00058
2E-06	0.00192	0.00328	0.00109	0.00124	7.7E-05
2E-06	0.00233	0.00217	0.00079	0.00094	0.00026
2E-06	0.00258	0.00272	0.00045	0.00053	-7E-05
2.1E-06	0.00229	0.00324	0.0007	0.0007	-0.0006
2.1E-06	0.00222	0.00283	0.00082	0.00088	-0.0008
2.1E-06	0.00254	0.00233	3.2E-05	0.00055	-0.0004
2.2E-06	0.00161	0.00255	0.00061	0.00085	0.00099
2.2E-06	0.00191	0.00264	0.00038	0.00056	0.00112
2.2E-06	0.00217	0.00184	-7E-05	0.0006	0.0007
2.3E-06	0.00179	0.0023	0.0007	0.00027	0.00014

2.3E-06	0.00145	0.00223	5.1E-05	0.00053	-0.0002
2.3E-06	0.00151	0.00178	0.00078	0.00082	-3E-05
2.4E-06	0.00154	0.00184	0.00113	-0.0004	0.00057
2.4E-06	0.00044	0.00245	0.00048	0.00062	0.00063
2.4E-06	0.00167	0.00226	0.00079	0.00108	0.00058
2.5E-06	0.00124	0.00226	0.00074	0.00032	0.00022
2.5E-06	0.00187	0.00268	0.00052	0.00067	-0.0002
2.5E-06	0.00205	0.00229	0.00065	0.00131	0.00093
2.6E-06	0.00123	0.002	0.00053	0.00061	0.00036
2.6E-06	0.00122	0.00262	0.00015	0.0011	0.00047
2.6E-06	0.00131	0.00232	0.00057	-0.0005	0.00012
2.7E-06	0.00179	0.0022	0.00046	0.00081	0.00018
2.7E-06	0.00117	0.0023	-3E-06	0.00129	0.00057
2.7E-06	0.00123	0.00168	0.00079	0.00032	0.00042
2.8E-06	0.00128	0.00245	0.00089	0.00072	-0.0003
2.8E-06	0.00123	0.00232	-0.0002	0.00086	-0.0001
2.8E-06	0.00106	0.00225	0.00045	0.00043	-0.0002
2.8E-06	0.0017	0.00177	-0.0004	0.00033	-4E-05
2.9E-06	0.00095	0.00146	0.00029	0.00079	0.00042
2.9E-06	0.00098	0.0018	7.8E-05	0.00015	-6E-05
2.9E-06	0.00105	0.00143	0.00075	0.00136	-0.0002
3E-06	0.0014	0.00165	-8E-05	0.00177	0.00051
3E-06	0.00076	0.0007	0.00073	0.00128	0.00047
3E-06	0.00094	0.00127	0.00104	-1E-05	0.001
3.1E-06	0.00129	0.00077	-0.0002	0.00125	0.00012
3.1E-06	0.00136	0.00217	0.00062	0.00036	0.00011
3.1E-06	0.00069	0.0017	-0.0003	-0.0003	-5E-05
3.2E-06	0.00086	0.00241	-0.0002	0.00075	-0.0001
3.2E-06	0.00054	0.00178	0.00029	0.00041	0.00041
3.2E-06	0.00037	0.00197	0.00076	0.0013	0.00032
3.3E-06	0.00092	0.00129	0.00028	0.00042	0.00038
3.3E-06	0.00011	0.00246	9.6E-05	-2E-05	0.00033
3.3E-06	0.00106	0.00099	0.00019	0.00025	0.00041
3.4E-06	0.00061	0.00132	0.00055	0.00091	0.00075
3.4E-06	0.00113	0.00154	-0.0002	0.00077	0.00039
3.4E-06	0.00113	0.00215	-0.0003	0.0008	-0.0003
3.5E-06	0.0005	0.00243	0.00065	0.0005	0.00036
3.5E-06	0.00124	0.00221	0.00063	0.00129	0.00036
3.5E-06	0.00108	0.00131	6.2E-05	-5E-05	0.00063
3.6E-06	0.00035	0.00198	0.00032	0.00081	-0.0002
3.6E-06	0.00073	0.00246	6.9E-05	0.00044	0.00063
3.6E-06	0.00013	0.00219	0.00028	-0.0004	0.00048
3.6E-06	0.00052	0.00139	-0.0008	0.00069	0.00067
3.7E-06	-8E-05	0.00038	0.00019	-9E-05	-0.0002
3.7E-06	0.00091	0.00144	-0.0008	0.00041	0.00055
3.7E-06	0.00023	0.00159	-0.0006	0.0006	0.00019
3.8E-06	0.0003	0.00159	-0.0002	0.00024	8.6E-05
3.8E-06	-0.0008	0.00155	-0.0002	0.0018	-1E-05
3.8E-06	6.8E-05	0.00143	0.00027	0.00011	-0.0006
3.9E-06	0.00079	0.00139	-0.0002	-0.001	0.00046
3.9E-06	0.00104	0.00193	-0.0002	-0.0008	0.00029
3.9E-06	0.00034	0.00208	-3E-05	0.00029	0.00031
4E-06	-5E-05	0.00232	-0.0005	-0.0004	0.00047
4E-06	0.00061	0.00227	-0.0001	0.00061	-0.0003

4E-06	0.00064	0.0021	-0.0003	0.00081	0.00067
4.1E-06	0.00043	0.00147	0.00017	0.00047	-0.0004
4.1E-06	0.00083	0.00108	-0.0008	0.00054	8.5E-05
4.1E-06	0.00024	0.00169	-0.0004	0.00036	0.00041
4.2E-06	0.00073	0.00033	-0.0003	0.0006	-0.0005
4.2E-06	0.00066	0.00182	-0.0002	0.00023	-3E-05
4.2E-06	0.00087	0.0015	0.00074	3.8E-05	0.0004
4.3E-06	0.00054	0.00166	0.00059	0.00157	-0.0002
4.3E-06	-0.0005	0.00198	0.00078	0.00136	3.7E-05
4.3E-06	0.00079	0.00112	0.00025	0.00093	0.00025
4.4E-06	0.00095	0.00151	8.1E-05	0.00065	-3E-05
4.4E-06	0.00029	0.00063	-7E-05	-0.0002	-0.0004
4.4E-06	0.0001	0.00083	-0.0002	-0.0003	0.00027
4.4E-06	0.00039	0.0016	-0.0002	0.00052	0.00066
4.5E-06	-4E-05	0.00152	-0.0004	0.00124	-8E-05
4.5E-06	0.00063	0.00103	9.6E-05	0.00016	0.00043
4.5E-06	0.0009	0.00128	-0.0006	0.00068	0.00021
4.6E-06	-0.0003	0.00143	0.00014	0.00013	0.00039
4.6E-06	0.00089	0.00186	-0.0005	0.0006	0.00045
4.6E-06	0.00018	0.00186	-0.0007	-0.0002	0.00049
4.7E-06	-0.0004	0.00127	0.00032	0.00015	-0.0002
4.7E-06	0.00056	0.00136	-8E-05	0.0002	-0.0003
4.7E-06	-0.0004	0.00128	0.00023	0.00037	0.0004
4.8E-06	0.00031	0.00179	0.00054	0.00083	0.00082
4.8E-06	0.00022	0.00128	0.00034	0.00091	0.00064
4.8E-06	0.0005	0.00205	0.0007	0.00022	0.00061
4.9E-06	0.00017	0.00146	-0.0003	0.00061	0.00069
4.9E-06	0.00035	0.00238	0.00057	0.00099	0.0009
4.9E-06	-0.0004	0.00137	0.00078	-0.0006	2.7E-05
5E-06	0.00058	0.00124	0.00032	0.00037	0.00038
5E-06	0.00086	0.0008	-0.0002	0.0008	0.00028
5E-06	7.4E-05	0.0015	2.3E-05	0.00074	0.00018
5.1E-06	0.00082	0.0018	-0.0007	0.00042	0.00011
5.1E-06	-7E-05	0.0012	8.1E-05	0.00082	9.5E-05
5.1E-06	-0.0007	0.00137	0.00051	0.00107	0.00075
5.2E-06	2.9E-05	0.00106	-2E-05	0.00034	0.00107
5.2E-06	0.00092	0.00046	0.00057	0.00012	0.00047
5.2E-06	0.00022	0.00141	-0.0004	-0.0001	-4E-05
5.2E-06	0.00089	0.00158	-0.0005	0.00022	-0.0004
5.3E-06	0.00026	0.00136	0.00024	0.00072	0.00028
5.3E-06	0.00058	0.00152	0.00052	0.00121	0.00027
5.3E-06	2.7E-05	0.00148	0.00034	2.2E-05	0.00029
5.4E-06	0.00019	0.00091	0.00021	-5E-06	0.0002
5.4E-06	-0.0006	0.00196	0.0008	-0.0002	-0.0003
5.4E-06	7.7E-05	0.00155	-0.0005	0.0002	0.00103
5.5E-06	0.00076	0.00097	0.00065	-3E-05	0.00036
5.5E-06	-0.0006	0.00106	0.00025	0.00016	0.00097
5.5E-06	0.00017	0.00183	0.00047	-0.0003	0.00022
5.6E-06	-1E-04	0.00081	-0.0003	-0.0004	0.0005
5.6E-06	-0.0005	0.00175	0.00011	0.00087	-0.0002
5.6E-06	-9E-05	0.00077	0.00108	0.00043	0.00032
5.7E-06	0.00028	0.00151	0.00068	0.00013	9E-05
5.7E-06	-3E-05	0.00152	0.00125	0.00058	0.00051
5.7E-06	-0.0005	0.00178	-4E-05	0.001	0.00065

5.8E-06	-0.0005	0.00175	-0.0008	-0.0005	0.00096
5.8E-06	-0.0008	0.00175	-0.0005	-0.0004	0.00134
5.8E-06	-0.0004	0.00125	0.00071	0.00057	0.00065
5.9E-06	-0.0003	0.00167	-0.0011	0.00101	0.00083
5.9E-06	-2E-05	0.00093	-1E-05	0.00026	0.00045
5.9E-06	-0.0002	0.00143	-0.0011	0.00028	-0.0005
6E-06	-4E-05	0.00152	0.00035	0.00026	-0.0005
6E-06	0.00021	0.00121	-8E-05	-2E-05	0.00042
6E-06	5.3E-05	0.00077	-7E-05	0.00038	0.00038
6E-06	-6E-05	0.00132	-0.0005	0.0007	0.00015
6.1E-06	0.0003	0.00138	0.00018	0.00058	-0.0004
6.1E-06	-1E-05	0.00085	0.00052	0.00057	0.00029
6.1E-06	0.00014	0.0008	0.00016	0.00045	0.00097
6.2E-06	-0.0003	0.00158	3E-05	0.0004	0.00064
6.2E-06	-3E-05	0.00129	4.4E-05	0.00086	0.00039
6.2E-06	-0.0003	0.00166	5.8E-05	0.00099	-7E-06
6.3E-06	0.00014	0.00137	-0.0001	0.00093	0.00078
6.3E-06	-0.0004	0.00149	0.00024	0.00036	0.00032
6.3E-06	0.00041	0.00145	0.00035	0.00022	0.00039
6.4E-06	5.3E-05	0.00161	-0.0003	-0.0003	0.00066
6.4E-06	0.00063	0.00132	0.00016	0.00047	-0.0001
6.4E-06	-0.0007	0.00138	0.00042	0.00047	-0.0003
6.5E-06	-4E-05	0.0014	-0.0006	0.00029	-0.0002
6.5E-06	0.00048	0.00136	0.00059	-0.0001	0.00106
6.5E-06	0.00014	0.0013	0.00022	0.00026	0.00025
6.6E-06	-7E-05	0.00156	-0.0002	0.00051	0.00088
6.6E-06	0.00062	0.00172	2.2E-05	-0.0001	-0.0001
6.6E-06	-0.0012	0.00051	-0.0003	8.8E-05	0.00062
6.7E-06	0.00037	0.00108	-0.0006	0.00023	0.00021
6.7E-06	-0.0002	0.00127	-0.0003	0.00051	0.00081
6.7E-06	0.00038	0.00219	-0.0001	0.00047	0.00061
6.8E-06	-0.0003	0.00067	-0.0003	0.00074	0.00077
6.8E-06	-0.0002	0.00156	0.00049	6.6E-05	-0.0002
6.8E-06	0.00013	0.00094	0.00071	-0.0005	0.00047
6.8E-06	0.00021	0.00136	0.00095	0.00019	0.00076
6.9E-06	0.00067	0.00084	0.00019	-0.0002	0.00054
6.9E-06	-8E-05	0.0012	-6E-05	0.00078	8.2E-05
6.9E-06	0.00047	0.00096	3.3E-05	4.6E-05	3.1E-05
7E-06	-0.0011	0.00085	0.0005	0.00028	0.0004
7E-06	-0.0001	0.00149	-5E-05	0.00068	0.00027
7E-06	-0.0005	0.00083	-0.0008	0.00143	-9E-05
7.1E-06	-0.0005	0.00144	-2E-05	0.00042	0.00078
7.1E-06	-6E-05	0.0013	0.00019	0.00103	-0.0002
7.1E-06	-0.0005	0.00142	0.0006	0.00019	0.00032
7.2E-06	0.00041	0.00118	0.00075	0.00049	0.00089
7.2E-06	-0.0003	0.00122	0.0005	0.00031	-0.0001
7.2E-06	-0.0005	0.00142	-0.0004	-0.0002	0.00089
7.3E-06	-0.0002	0.00101	0.00056	-0.0003	-4E-05
7.3E-06	-0.0006	0.00086	0.00036	0.00012	-4E-05
7.3E-06	-0.0003	0.00124	0.00015	1.7E-05	0.0005
7.4E-06	-0.0009	0.00123	0.00028	-7E-06	0.00055
7.4E-06	6E-05	0.00142	-0.0006	-0.0001	0.00053
7.4E-06	2.4E-05	0.00131	-0.0004	1.5E-05	0.00056
7.5E-06	0.00022	0.00092	-4E-05	0.00047	0.00026

7.5E-06	0.00049	0.00136	0.00052	-0.0002	0.00013
7.5E-06	-0.0009	0.00111	0.00025	0.00061	0.00105
7.6E-06	-0.0004	0.00159	0.00015	0.00027	0.0004
7.6E-06	0.00021	0.00134	0.00067	-5E-05	0.0002
7.6E-06	1.4E-05	0.00135	5.9E-05	0.0006	0.00094
7.6E-06	0.00021	0.00189	0.00011	0.00074	0.00027
7.7E-06	2.4E-05	0.00158	-0.0011	0.00013	0.00013
7.7E-06	0.00016	0.00142	0.00016	-0.0003	4.8E-05
7.7E-06	0.00026	0.00103	0.0004	-0.0003	0.00052
7.8E-06	-0.0002	0.00102	-0.0008	0.00034	-0.0003
7.8E-06	-0.0008	0.00158	-0.0005	0.00068	3.9E-06
7.8E-06	8.7E-05	0.00093	0.00095	0.00039	0.00077
7.9E-06	0.00077	0.00155	0.00036	-7E-05	0.00052
7.9E-06	-0.0004	0.00157	0.00053	0.00021	0.00047
7.9E-06	0.00017	0.00147	-4E-05	0.0007	0.00021
8E-06	0.00077	0.00108	-9E-05	-0.0001	-0.0004
8E-06	0.00018	0.00143	-0.0002	-0.0006	-0.0004
8E-06	0.00062	0.00126	0.00039	0.00068	0.00013
8.1E-06	-0.0002	0.00132	3.3E-05	0.00022	-0.0004
8.1E-06	0.0007	0.00065	5.5E-05	-0.0002	0.00016
8.1E-06	-0.0002	0.00065	0.00103	0.00018	0.00012
8.2E-06	5.2E-05	0.00122	0.00027	0.00061	0.00054
8.2E-06	-0.0001	0.00095	-0.0001	9.2E-05	-0.0003
8.2E-06	-0.0004	0.00124	0.00034	0.00124	0.00011
8.3E-06	0.0007	0.00117	0.00011	0.00026	0.00076
8.3E-06	0.00078	0.00079	0.00032	0.00039	0.00107
8.3E-06	0.00064	0.00139	0.00102	0.0015	-0.0001
8.4E-06	-0.0005	0.00095	0.00033	0.00081	0.00048
8.4E-06	3.6E-05	0.00195	0.00044	0.00026	0.00052
8.4E-06	0.00013	0.00079	0.00065	0.00046	0.00061
8.4E-06	-0.0003	0.00121	0.0004	-0.0004	-7E-05
8.5E-06	-0.0007	0.00122	0.00016	0.00031	0.00084
8.5E-06	-0.0003	0.00169	-7E-05	0.00043	0.00081
8.5E-06	0.00054	0.00089	-0.0005	0.00032	0.00075
8.6E-06	-0.0003	0.00061	-0.0001	-4E-05	0.00022
8.6E-06	0.00031	0.00125	0.00062	0.00084	0.00065
8.6E-06	-0.0003	0.00124	0.00039	0.00052	8.8E-05
8.7E-06	-0.0007	0.00125	0.00033	0.0007	0.00022
8.7E-06	-0.0005	0.00053	0.00023	-4E-05	0.00021
8.7E-06	-0.0002	0.00151	-0.0002	0.0001	0.00072
8.8E-06	-0.0007	0.00096	-0.0004	0.00084	-0.0001
8.8E-06	-0.0008	0.00097	-0.0003	0.00118	0.00029
8.8E-06	-0.0002	0.00133	0.00077	0.00094	0.00088
8.9E-06	-0.0006	0.00118	0.00075	0.00028	0.00016
8.9E-06	0.00088	0.00133	0.00025	0.00016	-5E-05
8.9E-06	0.00033	0.00061	0.00074	-0.0005	0.00021
9E-06	-0.0005	0.00115	0.00049	0.00046	0.00057
9E-06	-0.0003	0.00131	0.00014	0.00023	0.00062
9E-06	0.00027	0.00159	-5E-05	0.00107	0.0004
9.1E-06	0.00031	0.00091	0.00033	0.00026	0.00056
9.1E-06	-0.0009	0.00093	0.00031	0.0009	0.00073
9.1E-06	-0.0003	0.00121	0.00026	0.00045	0.0003
9.2E-06	-0.0008	0.00127	-0.0012	0.00093	0.0007
9.2E-06	0.00058	0.00083	-0.0007	-0.0002	0.00049

9.2E-06	0.00057	0.00129	0.00018	0.00048	9.3E-05
9.2E-06	0.00055	0.00098	0.00042	-7E-05	-0.0002
9.3E-06	0.00023	0.00091	-0.0006	-0.0008	0.00021
9.3E-06	-0.0004	0.00112	0.00013	0.00056	0.00012
9.3E-06	-0.0004	0.00126	-0.0002	0.00021	0.00055
9.4E-06	-0.0002	0.00066	0.00028	-2E-05	0.00022
9.4E-06	-0.0003	0.00054	-0.0005	0.00023	0.00056
9.4E-06	-4E-05	0.00131	0.0001	-0.0002	2.3E-05
9.5E-06	0.00062	0.00021	0.00022	-0.0005	-7E-05
9.5E-06	-0.001	0.00121	0.0004	0.00025	0.00059
9.5E-06	-0.0007	0.00046	0.00054	0.00108	-0.0002
9.6E-06	-1E-05	0.00131	0.00037	0.00029	0.00027
9.6E-06	0.00018	0.00089	-0.0004	-0.0004	0.00028
9.6E-06	-0.0005	0.0014	0.00043	9.8E-05	0.00048
9.7E-06	-0.0006	0.00124	-0.0004	0.00014	0.00075
9.7E-06	-0.0004	0.00094	0.00077	0.00037	8.2E-05
9.7E-06	0.00029	0.00079	0.00065	0.00015	-0.0004
9.8E-06	0.00058	0.00108	0.0007	0.0006	0.00061
9.8E-06	0.00015	0.00084	-0.0004	-0.0003	0.00013
9.8E-06	-0.0002	0.00112	-7E-05	-0.0005	0.00066
9.9E-06	-0.0008	0.00126	0.00069	0.00011	0.00043
9.9E-06	0.00036	0.00109	0.00036	-0.0004	0.00106
9.9E-06	-0.0005	0.00084	-0.0005	-0.0008	0.00083
1E-05	-0.0007	0.00104	0.00039	-0.0002	0.00066
1E-05	6.2E-05	0.00156	0.00081	-8E-05	0.00065
1E-05	-0.0005	0.00159	0.00047	0.00029	-0.0001
1E-05	-0.0006	0.0012	-0.0001	0.00023	0.00085
1E-05	-0.0008	0.00146	0.00066	3.3E-05	0.00081
1E-05	0.00057	0.00053	0.00062	1.3E-05	-0.0004
1E-05	-0.0014	0.00082	0.00047	0.00098	0.00026
1E-05	-0.0012	0.00087	0.00101	0.00058	0.00065
1E-05	-0.0005	0.00035	-4E-05	0.00099	0.00037
1E-05	-0.0001	0.00111	0.00031	0.00078	0.00106
1E-05	0.00033	0.00173	0.00015	0.00094	0.00084
1E-05	-0.0006	0.00041	0.00012	0.00038	0.00121
1E-05	0.00055	0.00048	-0.0002	0.00104	0.00037
1E-05	8.6E-06	0.00051	0.00102	0.00076	0.00086
1E-05	-0.0006	0.00099	-2E-05	0.00094	0.00109
1E-05	-0.0002	0.00109	-0.0004	0.00082	0.00102
1E-05	0.00035	0.00155	0.0002	-0.0004	0.00043
1.1E-05	-0.0002	0.00131	0.00066	0.0008	0.00095
1.1E-05	-0.0003	0.00083	0.0001	0.00076	0.00054
1.1E-05	-0.0004	0.00118	0.00054	0.00031	0.00064
1.1E-05	0.00105	0.00149	-0.0003	0.0006	0.00061
1.1E-05	0.00065	0.0015	0.00078	0.00055	0.00081
1.1E-05	0.00034	0.00073	0.00015	-6E-05	-0.0001
1.1E-05	-0.0001	0.00092	0.00027	0.00071	-1E-05
1.1E-05	-0.0005	0.00073	-0.0005	0.00024	0.00073
1.1E-05	0.00027	0.00179	0.00022	-0.0002	0.00109
1.1E-05	-8E-05	0.00157	5.5E-05	-7E-05	0.00075
1.1E-05	-0.0005	0.00152	0.00053	0.0001	0.00029
1.1E-05	-0.0002	0.00136	0.0004	0.00012	0.00054
1.1E-05	0.00053	0.00118	0.00018	0.00024	0.00017
1.1E-05	0.00039	0.00135	0.00032	2.2E-05	-0.0003

1.1E-05	0.00036	0.00211	-8E-05	-0.0006	0.0003
1.1E-05	9.9E-05	-0.0005	6.3E-05	-0.0001	0.00047
1.1E-05	0.00018	0.00097	0.00039	-0.0008	0.00084
1.1E-05	-9E-05	0.00186	-0.0004	0.00083	9E-05
1.1E-05	0.00036	0.00132	-0.0009	0.0014	-7E-05
1.1E-05	-0.0001	0.00075	0.00015	0.00015	0.00072
1.1E-05	0.00023	0.00058	-0.0002	0.00065	0.00018
1.1E-05	0.00028	0.00075	0.00024	-0.0006	0.00042
1.1E-05	-0.0001	0.0015	0.00037	-0.0004	0.00081
1.1E-05	-5E-05	0.00114	0.00015	0.00022	-0.0008
1.1E-05	0.00073	0.00102	0.00069	0.00022	1E-04
1.1E-05	-9E-07	0.00084	0.00013	0.00095	0.00015
1.1E-05	5.7E-06	0.0003	0.00045	-0.0002	-0.0002
1.1E-05	-0.0005	0.00145	-0.0003	0.0001	0.00048
1.1E-05	-0.0003	0.00045	0.0002	0.00068	0.00021
1.1E-05	0.00056	0.00214	-0.0002	0.00057	0.00048
1.1E-05	-0.0003	0.00191	-8E-05	-0.0001	0.00028
1.1E-05	-0.0009	0.00116	0.00071	0.00022	0.00074
1.2E-05	0.00027	0.0018	0.00065	-0.0002	0.00014
1.2E-05	-0.0002	0.00114	0.00059	-2E-05	0.00073
1.2E-05	-0.0003	0.00112	-0.0004	0.0003	0.00077
1.2E-05	-0.0001	0.00113	0.00082	0.00036	0.00055
1.2E-05	-0.0002	0.00153	0.00071	0.00098	0.00077
1.2E-05	-0.0003	0.00127	0.0003	-0.0002	0.00047
1.2E-05	-5E-05	0.00167	-0.0004	0.00059	-0.0004
1.2E-05	-0.0007	0.00116	-0.0001	-0.0002	0.00068
1.2E-05	0.00028	0.00145	2.8E-06	-6E-05	0.00095
1.2E-05	-0.0002	0.00058	0.00018	0.00037	0.00041
1.2E-05	0.00034	0.00044	-0.0006	0.00036	0.00038
1.2E-05	-0.0005	0.00117	2.9E-05	0.00063	0.00037
1.2E-05	0.00062	0.00133	8.8E-07	0.00023	0.00071
1.2E-05	0.00012	0.00085	0.00027	-0.0002	0.00095
1.2E-05	-0.0004	0.00112	0.00114	0.00083	0.0008
1.2E-05	0.00067	0.00043	0.00043	3.7E-05	0.00059
1.2E-05	-0.0005	0.00072	0.00038	-0.0001	2E-05
1.2E-05	0.00014	0.00132	0.00046	-8E-05	0.0004
1.2E-05	-0.0008	0.00117	0.00041	0.00063	-0.0002
1.2E-05	-9E-05	0.00112	0.00064	0.00046	0.00091
1.2E-05	0.00045	0.00102	0.00011	-3E-06	-0.0004
1.2E-05	-0.0006	0.00028	0.00039	0.00028	0.00011
1.2E-05	-2E-05	0.00018	0.00039	0.00041	0.00034
1.2E-05	-0.0002	0.00098	-0.0003	0.00144	0.00052
1.2E-05	-0.0004	0.00118	-0.0003	-0.0002	2.5E-05
1.2E-05	-0.0002	0.00052	6.6E-05	-9E-05	-0.0004
1.2E-05	-7E-05	0.00115	0.00061	0.0005	0.00029
1.2E-05	0.00023	0.00162	0.0006	0.00043	0.00077
1.2E-05	-0.0001	0.00119	0.00042	0.00079	0.00091
1.2E-05	-0.0006	0.00083	-0.0002	0.00097	0.00023
1.2E-05	-0.0007	0.00106	0.00032	-5E-05	8.5E-05
1.3E-05	-0.0004	0.00085	0.00062	-0.0006	0.0005
1.3E-05	5.4E-05	0.00096	3.5E-05	0.00049	0.00056
1.3E-05	-0.0002	0.00183	-0.0005	0.0006	0.00063
1.3E-05	0.00038	0.00177	0.00057	0.00037	0.00047
1.3E-05	-0.0005	0.00042	0.00064	0.00026	0.00054

1.3E-05	0.00048	0.0009	-1E-05	0.00032	0.00056
1.3E-05	0.00028	0.00168	9.3E-06	2.8E-05	-1E-04
1.3E-05	-4E-05	0.00108	-3E-08	0.00123	0.00052
1.3E-05	-0.0007	0.0013	0.0007	0.00042	-0.0002
1.3E-05	9.1E-05	0.00012	0.00013	0.00029	0.00105
1.3E-05	6.4E-05	0.00025	5.3E-05	0.00047	0.00089
1.3E-05	-0.0006	0.00081	0.00024	0.00095	0.00072
1.3E-05	-0.0001	0.00067	-0.0002	0.00097	0.00098
1.3E-05	-0.0008	0.00052	7.9E-06	0.00027	0.00013
1.3E-05	0.00087	-0.0003	0.00046	0.00108	0.00018
1.3E-05	0.00031	0.00024	0.00031	0.00031	0.00098
1.3E-05	0.00022	0.00134	0.00069	-0.0003	0.00043
1.3E-05	-0.0002	0.00068	0.0002	0.0005	0.00065
1.3E-05	-0.0005	0.00063	-0.0002	0.00062	0.0007
1.3E-05	-0.0004	0.00112	0.00011	0.00049	0.00029
1.3E-05	-0.0001	0.00023	0.00033	0.00036	0.00175
1.3E-05	-7E-05	0.00047	0.00054	-8E-06	0.00093
1.3E-05	0.00057	0.00158	-0.0005	-2E-05	0.0002
1.3E-05	8.9E-06	0.00071	-0.0004	0.00061	0.00027
1.3E-05	-0.0003	0.00073	-0.0007	1.7E-05	0.00052
1.3E-05	-1E-05	0.00106	-0.0003	0.00024	0.00044
1.3E-05	9.1E-05	0.00063	-0.0002	-6E-05	0.00086
1.3E-05	-0.0001	0.00175	-0.0008	0.0005	0.00094
1.3E-05	-0.0003	0.00177	-0.0001	0.00025	0.00039
1.3E-05	-0.0001	0.00093	9.2E-05	-0.0002	-0.0003
1.3E-05	-0.0003	0.00087	0.00034	0.00026	-0.0002
1.4E-05	-0.0002	0.00039	-0.0002	0.00022	0.0002
1.4E-05	-7E-05	7.2E-05	-0.0003	0.0004	-0.0006
1.4E-05	-0.0004	0.00028	0.00066	0.00023	0.00051
1.4E-05	-0.0001	0.00131	0.00041	0.00011	-0.0002
1.4E-05	-0.0006	0.00087	0.00066	3.5E-05	0.00035
1.4E-05	-0.0007	0.00187	-0.0002	0.00046	0.00024
1.4E-05	-0.0002	0.00032	-0.0002	0.00059	1.6E-06
1.4E-05	-0.0002	0.00133	0.00018	0.00076	0.00023
1.4E-05	-0.0005	0.00086	0.0006	1.6E-05	0.00046
1.4E-05	0.00012	0.0004	0.00031	0.00014	0.0001
1.4E-05	-0.0006	0.00139	-0.0005	0.00046	0.00028
1.4E-05	-3E-05	0.00154	0.00044	-0.0002	-0.0004
1.4E-05	-0.0007	0.00167	0.00034	0.00022	0.00015
1.4E-05	-5E-06	0.00109	-9E-05	0.00014	-0.0002
1.4E-05	-0.0002	0.00063	-0.0001	0.00033	0.00027
1.4E-05	-0.0001	0.00127	2.3E-05	0.00109	0.00026
1.4E-05	-0.0004	0.00011	2.8E-05	0.00034	0.00015
1.4E-05	0.00013	0.0012	0.0004	4.1E-05	0.00055
1.4E-05	-0.0003	0.00065	0.0003	0.00012	0.00045
1.4E-05	-0.0005	0.00059	0.00066	0.00043	-0.0005
1.4E-05	0.0002	0.0008	8.5E-05	0.00022	-0.0004
1.4E-05	0.00032	0.00051	0.00047	0.00047	0.00025
1.4E-05	0.00032	0.00084	0.00019	0.00061	0.00018
1.4E-05	-7E-05	0.00098	0.00068	0.00039	0.0002
1.4E-05	-0.0003	0.00133	-0.0007	5.4E-05	0.00025
1.4E-05	-0.0005	0.00111	-0.0002	0.00082	0.00049
1.4E-05	-0.0003	8.3E-05	-7E-05	0.00064	0.00029
1.4E-05	-0.0003	0.00065	0.00103	0.00051	3.6E-05

1.4E-05	-0.0004	0.00125	0.00012	-0.0006	0.00105
1.4E-05	-0.0005	0.00069	-0.0003	-5E-05	0.00017
1.4E-05	-0.0006	0.00112	-0.0004	0.00102	0.00047
1.5E-05	0.00012	0.00028	0.00014	-0.0003	-0.0005
1.5E-05	-0.0008	0.00083	-6E-05	-0.0004	-0.0005
1.5E-05	-5E-05	0.00066	-9E-05	-9E-06	-6E-06
1.5E-05	-0.0006	0.00107	-0.0001	0.00064	-1E-05
1.5E-05	-0.0002	0.00126	-0.0004	3.5E-05	-0.0002
1.5E-05	-0.001	0.00053	-4E-05	0.00042	0.00057
1.5E-05	-0.0003	0.00092	-0.0004	-6E-05	-0.0002
1.5E-05	-0.0008	0.00066	0.00012	0.00042	0.00039
1.5E-05	-0.0004	0.00051	-0.0004	0.00061	0.00044
1.5E-05	4.3E-05	0.00133	-0.0008	-4E-05	0.00066
1.5E-05	3.6E-05	3.5E-05	-0.0005	-0.0001	0.00017
1.5E-05	-0.0003	0.00123	-0.0001	0.00054	0.00096
1.5E-05	-0.0003	0.00074	-0.0006	0.0002	0.00066
1.5E-05	-0.0002	0.00037	-0.0002	0.00064	0.00031
1.5E-05	-0.0008	0.00077	-0.0003	-0.0002	0.00083
1.5E-05	-0.0002	-0.0004	-0.0002	0.00026	0.00061

Table SI.2B: Primary kinetic data for the reaction of 2-pyrrolidone (**S2**) with CumO$^{\bullet}$ generated by 355 nm LFP in DMSO (**Figure SI.2B**).

**t / s**	**ΔA 2-pyrrolidinone (S2)**				
	**0.0324M**	**0.130M**	**0.226M**	**0.352M**	**0.476M**
-1E-06	0.00081	-0.0006	0.00035	0.00037	-3E-05
-1E-06	0.00133	-0.0002	0.00012	-1E-05	0.00092
-9E-07	0.0007	-0.0011	-0.0004	-0.0003	0.00103
-9E-07	0.00061	-2E-05	-0.0012	0.00037	0.00055
-9E-07	0.00033	0.00089	-0.0002	0.00057	0.00082
-8E-07	0.00109	0.00034	0.00033	7.8E-05	0.0008
-8E-07	0.00116	0.00039	0.0006	-0.0001	0.00087
-8E-07	0.00078	-0.0008	-0.0003	0.00074	0.00071
-7E-07	0.00082	0.00036	0.00025	0.00029	0.00159
-7E-07	0.00075	-0.0003	0.00021	-0.0003	0.00084
-7E-07	-2E-05	-0.0007	-2E-05	6.1E-05	0.00099
-6E-07	0.0013	-0.0004	-0.0001	0.00057	0.0006
-6E-07	0.00018	7.9E-05	0.00035	-0.0003	0.00121
-6E-07	0.00035	0.00021	-0.0003	-0.0003	0.00102
-5E-07	0.0012	-0.0004	-0.0003	3.1E-06	0.00106
-5E-07	0.00047	0.00054	0.00038	-0.0002	0.00023
-5E-07	0.00057	0.0009	-0.0006	-0.0004	0.00087
-4E-07	0.00123	-0.0003	-0.0002	-0.0003	0.00161
-4E-07	0.00171	-0.0005	-7E-06	-0.0002	0.00075
-4E-07	0.0004	-2E-06	-0.0006	-0.0004	0.00096
-4E-07	0.00039	0.00025	0.00018	-6E-05	0.00086
-3E-07	0.00111	0.00045	-0.0001	0.00084	0.00057
-3E-07	0.00025	3.9E-05	0.00014	-0.0002	0.00035
-3E-07	0.00021	0.00032	0.00011	0.00011	0.00093
-2E-07	0.00056	-0.0002	-0.0002	-0.0006	0.00056
-2E-07	-0.0002	7.8E-05	-0.0002	-0.001	0.00042
-2E-07	0.00071	-0.0003	-0.0007	-5E-05	0.00079
-1E-07	0.00089	-3E-05	-0.0002	-0.0002	0.00029
-1E-07	0.00074	-0.0001	-0.0005	-0.0008	0.00023
-6E-08	0.00161	0.00066	-0.0008	-2E-05	0.00109
-3E-08	0.0009	-0.0002	-0.0008	-0.0004	0.00044
-2E-10	-0.0032	-0.0041	-0.0035	-0.003	-0.0024
3.2E-08	-0.0138	-0.019	-0.019	-0.0197	-0.0165
6.4E-08	0.02249	0.0154	0.01232	0.00811	0.00681
9.6E-08	0.0259	0.02197	0.02219	0.0208	0.02051
1.3E-07	0.02611	0.02163	0.0215	0.01915	0.01947
1.6E-07	0.02532	0.01985	0.02072	0.01781	0.01894
1.9E-07	0.02458	0.01946	0.01902	0.01728	0.01798
2.2E-07	0.02376	0.01943	0.01843	0.01581	0.01648
2.6E-07	0.02396	0.01896	0.01679	0.01551	0.01477
2.9E-07	0.02139	0.01751	0.01651	0.01447	0.01386
3.2E-07	0.02182	0.01715	0.01509	0.01385	0.01305
3.5E-07	0.02167	0.01609	0.01525	0.01226	0.01172
3.8E-07	0.02039	0.01565	0.01443	0.013	0.01188
4.2E-07	0.02055	0.01536	0.0135	0.0117	0.01055
4.5E-07	0.01946	0.01504	0.01343	0.01055	0.00913
4.8E-07	0.01858	0.01411	0.01243	0.0098	0.01023
5.1E-07	0.01727	0.01401	0.01117	0.00953	0.009
5.4E-07	0.01771	0.01342	0.01142	0.00901	0.00856

5.8E-07	0.01682	0.01242	0.01082	0.00801	0.00775
6.1E-07	0.01711	0.01274	0.0098	0.00753	0.00757
6.4E-07	0.01623	0.012	0.00978	0.00799	0.00591
6.7E-07	0.01663	0.01156	0.0085	0.00715	0.00637
7E-07	0.01607	0.01028	0.00863	0.00696	0.00609
7.4E-07	0.01507	0.01046	0.00857	0.00597	0.0058
7.7E-07	0.0146	0.01018	0.00814	0.00539	0.00538
8E-07	0.0139	0.0095	0.00737	0.00578	0.00628
8.3E-07	0.01489	0.00897	0.00696	0.00576	0.0048
8.6E-07	0.01355	0.00894	0.00711	0.00517	0.00479
9E-07	0.01322	0.00829	0.00669	0.00501	0.00528
9.3E-07	0.01317	0.00865	0.00595	0.00413	0.00453
9.6E-07	0.01272	0.00791	0.00592	0.00457	0.0043
9.9E-07	0.01157	0.00763	0.00568	0.00403	0.00366
1E-06	0.01181	0.00803	0.0055	0.00338	0.00431
1.1E-06	0.01233	0.00714	0.00526	0.00334	0.00375
1.1E-06	0.01179	0.0072	0.00484	0.00378	0.00317
1.1E-06	0.01153	0.00646	0.00482	0.00405	0.00343
1.2E-06	0.01065	0.00587	0.00522	0.00334	0.00298
1.2E-06	0.01107	0.00722	0.00455	0.00315	0.00328
1.2E-06	0.01052	0.00578	0.00343	0.00292	0.00242
1.2E-06	0.00922	0.00619	0.00394	0.00269	0.00314
1.3E-06	0.00961	0.00637	0.00393	0.00298	0.00297
1.3E-06	0.00877	0.00492	0.00422	0.00257	0.00292
1.3E-06	0.00923	0.00459	0.00351	0.00227	0.00268
1.4E-06	0.00956	0.00524	0.0036	0.00201	0.00248
1.4E-06	0.00901	0.0048	0.00369	0.00206	0.00224
1.4E-06	0.00905	0.00473	0.00274	0.00248	0.0029
1.5E-06	0.00851	0.00507	0.00223	0.00292	0.00339
1.5E-06	0.00867	0.00427	0.00279	0.00203	0.00243
1.5E-06	0.0083	0.00551	0.00225	0.00185	0.00282
1.6E-06	0.00692	0.00336	0.00288	0.00223	0.00253
1.6E-06	0.0075	0.00345	0.00258	0.00236	0.0024
1.6E-06	0.00779	0.00382	0.00282	0.00146	0.00173
1.7E-06	0.00742	0.00275	0.00231	0.00182	0.00293
1.7E-06	0.00749	0.00374	0.00232	0.00175	0.0032
1.7E-06	0.0074	0.00312	0.00184	0.00224	0.00328
1.8E-06	0.00679	0.00248	0.00262	0.00143	0.002
1.8E-06	0.00649	0.00324	0.00239	0.00241	0.00258
1.8E-06	0.00652	0.00273	0.00234	0.00157	0.00194
1.9E-06	0.00694	0.00341	0.00246	0.00154	0.00261
1.9E-06	0.00632	0.00346	0.00269	0.00114	0.00237
1.9E-06	0.00644	0.00363	0.00202	0.00197	0.00255
2E-06	0.00662	0.0018	0.00194	0.00215	0.00248
2E-06	0.00646	0.00276	0.00105	0.00238	0.00283
2E-06	0.00677	0.00288	0.00181	0.00135	0.00272
2E-06	0.00587	0.00177	0.00171	0.00237	0.00167
2.1E-06	0.00654	0.00248	0.00209	0.002	0.00155
2.1E-06	0.00565	0.00265	0.00153	0.00174	0.00192
2.1E-06	0.00632	0.00243	0.00164	0.00142	0.00166
2.2E-06	0.00516	0.00189	0.0022	0.00146	0.00207
2.2E-06	0.00486	0.00262	0.00204	0.00133	0.00195
2.2E-06	0.00453	0.00213	0.00167	0.00187	0.00212
2.3E-06	0.00513	0.00264	0.00256	0.00168	0.00235

2.3E-06	0.00487	0.00158	0.00179	0.00145	0.00183
2.3E-06	0.00498	0.00132	0.00216	0.0014	0.0018
2.4E-06	0.0043	0.00223	0.00246	0.00176	0.002
2.4E-06	0.00481	0.00192	0.00198	0.00127	0.00261
2.4E-06	0.00466	0.00299	0.00167	0.0016	0.00284
2.5E-06	0.00469	0.00252	0.00125	0.00157	0.00188
2.5E-06	0.00518	0.00167	0.00159	0.00244	0.00215
2.5E-06	0.00425	0.00155	0.00232	0.00189	0.00171
2.6E-06	0.00513	0.00171	0.00129	0.00111	0.0019
2.6E-06	0.00566	0.00194	0.00197	0.00146	0.00167
2.6E-06	0.00569	0.00197	0.00042	0.00156	0.00148
2.7E-06	0.00477	0.00191	0.00179	0.00185	0.00253
2.7E-06	0.00489	0.00138	0.00169	0.00128	0.00181
2.7E-06	0.00422	0.00147	0.00101	0.00147	0.00251
2.8E-06	0.00426	0.00166	0.00152	0.0018	0.00222
2.8E-06	0.00441	0.00221	0.0017	0.00114	0.00279
2.8E-06	0.00376	0.00197	0.0012	0.00081	0.00184
2.8E-06	0.00415	0.00279	0.00136	0.00078	0.00192
2.9E-06	0.00392	0.00095	0.00219	0.00126	0.00149
2.9E-06	0.00381	0.00114	0.00099	0.00179	0.00186
2.9E-06	0.0038	0.00145	0.00143	0.00086	0.00167
3E-06	0.00326	0.0009	0.00037	0.00151	0.00154
3E-06	0.0036	0.00156	0.00125	0.00102	0.00211
3E-06	0.00386	0.00181	0.00065	0.00057	0.00207
3.1E-06	0.00399	0.0025	0.0011	0.00119	0.00147
3.1E-06	0.00392	0.00107	0.00095	0.0011	0.00125
3.1E-06	0.0035	0.00108	0.00163	0.00098	0.00088
3.2E-06	0.00447	0.0016	0.00113	0.00122	0.00126
3.2E-06	0.00453	0.00249	0.00096	0.00064	0.00109
3.2E-06	0.00434	0.00185	0.00095	0.00155	0.00148
3.3E-06	0.00362	0.00174	0.00125	0.00143	0.00104
3.3E-06	0.0033	0.00181	0.00134	0.00114	0.00166
3.3E-06	0.00334	0.00097	0.00176	0.00165	0.00156
3.4E-06	0.00349	0.00235	0.00145	0.00115	0.0016
3.4E-06	0.00368	0.00151	0.00119	0.00118	0.00212
3.4E-06	0.00414	0.00145	0.00033	0.00052	0.00125
3.5E-06	0.00356	0.0018	0.00076	0.00027	0.00177
3.5E-06	0.00351	0.00097	0.00152	0.00119	0.00163
3.5E-06	0.00326	0.00194	0.00066	0.00022	0.00129
3.6E-06	0.00339	0.00148	0.00098	0.00043	0.00076
3.6E-06	0.00392	0.00166	0.00126	0.0016	0.00107
3.6E-06	0.00297	0.00096	0.00101	0.0015	0.00157
3.6E-06	0.00318	0.00247	0.00054	0.00025	0.00184
3.7E-06	0.00279	0.00086	0.00167	0.00063	0.00169
3.7E-06	0.00304	0.00091	0.00112	0.00057	0.00175
3.7E-06	0.00355	0.00117	0.00077	0.00061	0.00156
3.8E-06	0.00292	0.00135	0.00088	0.00069	0.00211
3.8E-06	0.0039	0.00113	0.00155	0.00067	0.00192
3.8E-06	0.00324	0.00096	0.00017	0.00106	0.00166
3.9E-06	0.0032	0.00144	0.00202	0.00107	0.00218
3.9E-06	0.0029	0.00114	0.00178	0.00096	0.00154
3.9E-06	0.00337	0.00099	0.00073	0.00115	0.00149
4E-06	0.00266	0.00163	0.00138	0.00185	0.00182
4E-06	0.00277	0.00122	0.00103	-6E-05	0.00172

4E-06	0.00347	0.00171	0.00092	0.00046	0.00151
4.1E-06	0.00265	0.00174	0.00111	0.00108	0.00153
4.1E-06	0.00275	0.00118	0.00057	0.00038	0.00184
4.1E-06	0.00337	0.0012	0.00131	0.00065	0.00165
4.2E-06	0.00364	0.00174	0.00102	0.00055	0.00203
4.2E-06	0.00315	0.0014	0.00119	0.00085	0.00174
4.2E-06	0.00337	0.00109	0.00159	0.00082	0.00124
4.3E-06	0.00354	0.00195	0.00119	0.00117	0.00094
4.3E-06	0.00328	0.00176	0.00097	0.00127	0.00214
4.3E-06	0.00276	0.00118	0.00065	0.00019	0.00144
4.4E-06	0.00308	0.00072	0.00151	0.00114	0.00182
4.4E-06	0.00265	0.00124	0.00111	0.00076	0.00145
4.4E-06	0.00239	0.00164	0.00068	0.00093	0.00163
4.4E-06	0.00211	0.00181	0.00149	0.00077	0.00138
4.5E-06	0.00155	0.00156	0.0014	0.00106	0.00169
4.5E-06	0.00334	0.0011	0.00175	0.00095	0.00136
4.5E-06	0.00231	0.00094	0.00064	0.00088	0.00179
4.6E-06	0.00178	0.00101	0.00093	0.0018	0.00114
4.6E-06	0.00288	0.00171	0.00162	0.001	0.00129
4.6E-06	0.003	0.00101	0.0017	0.00052	0.00146
4.7E-06	0.00239	0.00131	0.00111	0.00037	0.00196
4.7E-06	0.00244	0.00125	0.00132	0.00039	0.00138
4.7E-06	0.00331	0.00138	0.00044	-3E-05	0.00114
4.8E-06	0.00275	0.00117	0.0013	0.001	0.00127
4.8E-06	0.00243	0.00111	0.00155	0.00084	0.00107
4.8E-06	0.00237	0.00155	0.00134	0.00051	0.00119
4.9E-06	0.00199	0.00134	0.00135	0.00047	0.00168
4.9E-06	0.00215	0.00121	0.00109	0.00055	0.00189
4.9E-06	0.00281	0.00152	0.00059	0.00093	0.00141
5E-06	0.00255	0.00107	0.00104	0.00109	0.00172
5E-06	0.00308	0.00133	0.00146	0.0008	0.00207
5E-06	0.00242	0.0017	0.00158	0.00091	0.00128
5.1E-06	0.00342	0.00107	0.00084	0.00159	0.00192
5.1E-06	0.00308	0.00199	0.00039	0.0008	0.00126
5.1E-06	0.00244	0.00151	0.00085	0.00122	0.0014
5.2E-06	0.00329	0.00091	0.00061	0.00136	0.00123
5.2E-06	0.00317	0.00188	0.00115	0.00044	0.00126
5.2E-06	0.00261	0.00187	0.00148	0.00166	0.00195
5.2E-06	0.0029	0.00137	0.00149	0.00165	0.00192
5.3E-06	0.00131	0.00188	0.00085	0.00075	0.00263
5.3E-06	0.00246	0.00099	0.00096	0.00151	0.0011
5.3E-06	0.00165	0.00128	0.00122	0.00069	0.00122
5.4E-06	0.0029	0.00172	0.00131	0.00112	0.00147
5.4E-06	0.00261	0.00175	0.00153	0.00017	0.00146
5.4E-06	0.00302	0.0005	0.00057	0.00122	0.00155
5.5E-06	0.00309	0.00074	0.00075	0.00027	0.00181
5.5E-06	0.00264	0.00101	0.00112	0.00058	0.00157
5.5E-06	0.00197	0.00154	0.00065	1.1E-06	0.00152
5.6E-06	0.002	0.00066	0.00156	0.00115	0.00206
5.6E-06	0.00316	0.00126	0.00097	0.00087	0.00141
5.6E-06	0.00221	0.00088	0.00118	0.00096	0.00143
5.7E-06	0.00264	0.00136	0.0012	0.00106	0.00115
5.7E-06	0.00189	0.00124	0.0011	0.00015	0.00078
5.7E-06	0.00285	0.00093	0.0012	0.00138	0.00159

5.8E-06	0.00296	0.00162	0.00068	0.0009	0.00175
5.8E-06	0.00256	0.0013	0.00053	0.00037	0.00141
5.8E-06	0.00187	0.00063	0.00134	0.00031	0.00077
5.9E-06	0.00142	0.00112	0.00108	0.0008	0.00135
5.9E-06	0.00174	0.00116	2.3E-05	0.00106	0.00174
5.9E-06	0.00195	0.00119	0.00065	0.00115	0.00099
6E-06	0.00202	0.00102	0.00044	-0.0001	0.00207
6E-06	0.00174	0.00074	0.00034	0.00096	0.0017
6E-06	0.00222	0.0012	0.00144	0.00119	0.00188
6E-06	0.00157	0.00137	0.00154	0.00145	0.00123
6.1E-06	0.00285	0.00133	0.00076	0.00143	0.00141
6.1E-06	0.00276	0.00229	0.0006	0.00133	0.00155
6.1E-06	0.00209	0.00126	0.00064	0.00118	0.00123
6.2E-06	0.00225	0.00078	0.00119	0.00063	0.00145
6.2E-06	0.00217	0.00051	0.00124	0.00047	0.00112
6.2E-06	0.00237	0.0014	0.00163	0.00068	0.00113
6.3E-06	0.00313	0.00062	0.00174	0.00139	0.00062
6.3E-06	0.00211	0.00147	0.00078	0.0009	0.00094
6.3E-06	0.00327	0.0012	0.00122	0.00019	0.00146
6.4E-06	0.00212	0.00074	0.00112	0.00073	0.00161
6.4E-06	0.002	0.00073	0.00129	0.00045	0.00112
6.4E-06	0.00326	0.00174	0.00127	0.00051	0.00134
6.5E-06	0.00161	0.00136	0.00063	0.00103	0.00105
6.5E-06	0.00234	0.00158	0.00074	0.0009	0.00091
6.5E-06	0.00262	0.00095	0.00209	0.00036	0.00111
6.6E-06	0.00312	0.00094	0.00114	0.00115	0.00141
6.6E-06	0.0032	0.00157	0.00113	0.00066	0.00047
6.6E-06	0.00191	0.00065	0.00068	0.00086	0.00112
6.7E-06	0.00234	0.00081	0.00093	0.00079	0.00173
6.7E-06	0.00192	0.00082	0.00145	-1E-05	0.00178
6.7E-06	0.00244	0.00173	0.00069	0.00069	0.00121
6.8E-06	0.00209	0.00078	0.00134	0.00123	0.00199
6.8E-06	0.00161	0.00076	0.00055	0.00098	0.00147
6.8E-06	0.00219	0.00165	0.00068	0.00026	0.00142
6.8E-06	0.00109	0.00167	0.00119	0.00107	0.00133
6.9E-06	0.00203	0.00155	0.00131	0.00033	0.00124
6.9E-06	0.00187	0.00043	0.00071	9.9E-05	0.00095
6.9E-06	0.00134	0.00102	0.00151	0.00187	0.00103
7E-06	0.00124	0.00128	0.00123	0.00043	0.00119
7E-06	0.00098	0.00135	0.00065	0.0002	0.00154
7E-06	0.0017	0.0008	0.0003	0.00065	0.00136
7.1E-06	0.00096	0.00136	0.00075	0.00105	0.00054
7.1E-06	0.00178	0.00162	0.0016	0.00104	0.00178
7.1E-06	0.00125	0.00145	0.00019	0.00074	0.00187
7.2E-06	0.00149	0.00094	0.00056	0.00098	0.00139
7.2E-06	0.00262	0.00176	0.00074	0.00049	0.00122
7.2E-06	0.00183	0.00077	0.00084	0.0011	0.00142
7.3E-06	0.00176	0.0008	0.00119	0.00047	0.00135
7.3E-06	0.00131	0.00142	0.0002	0.00022	0.0004
7.3E-06	0.00116	0.0008	0.00096	0.00067	0.00077
7.4E-06	0.00236	0.00113	-0.0006	0.00073	0.00158
7.4E-06	0.00143	0.00155	0.00149	0.00037	0.00119
7.4E-06	0.00209	0.00044	0.00055	0.00054	0.00066
7.5E-06	0.00224	0.00112	0.00105	0.00063	0.00129

7.5E-06	0.00229	0.00143	0.00138	0.00135	0.00088
7.5E-06	0.00099	0.00116	0.00123	0.002	0.00129
7.6E-06	0.00215	0.00139	0.00145	0.00066	0.00113
7.6E-06	0.00183	0.00079	1.6E-05	-5E-05	0.0009
7.6E-06	0.00171	0.00136	0.00059	0.00064	0.00162
7.6E-06	0.00252	0.0009	0.00109	0.0005	0.00087
7.7E-06	0.00187	0.00092	0.00091	9.8E-05	0.00076
7.7E-06	0.00188	0.00045	0.00127	0.00067	0.001
7.7E-06	0.00195	0.00085	5.9E-05	0.00036	0.00092
7.8E-06	0.00154	0.00149	0.00142	0.00015	0.00111
7.8E-06	0.00103	0.00071	0.00146	0.00229	0.00141
7.8E-06	0.0021	0.0015	0.00052	3.3E-05	0.00112
7.9E-06	0.0024	0.00185	0.00104	0.00117	0.00082
7.9E-06	0.00148	0.0007	0.00039	0.00083	0.0015
7.9E-06	0.00183	0.00139	0.00088	0.00102	0.00055
8E-06	0.00228	0.00129	0.00033	0.00049	0.00099
8E-06	0.00198	0.00011	0.00015	0.00054	0.00133
8E-06	0.00146	0.00033	0.00062	0.00057	0.00058
8.1E-06	0.00194	0.00102	0.00126	0.0007	0.00086
8.1E-06	0.002	0.0013	0.00125	0.00032	0.00152
8.1E-06	0.00206	0.00157	0.00096	0.00099	0.00025
8.2E-06	0.0013	0.00058	0.0016	1.2E-05	0.00095
8.2E-06	0.00166	0.00091	0.00014	0.00152	0.00124
8.2E-06	0.00203	0.00082	0.00103	0.00084	0.00128
8.3E-06	0.00167	0.00108	0.00135	0.00085	0.00112
8.3E-06	0.00109	7E-05	0.0011	0.00058	0.00046
8.3E-06	0.00092	0.00021	0.00048	0.0007	0.00137
8.4E-06	0.0015	0.00178	0.00053	0.00096	0.00072
8.4E-06	0.00218	0.00131	0.00061	0.00115	0.00106
8.4E-06	0.00145	0.00131	0.00113	0.00089	0.00138
8.4E-06	0.00171	0.00112	0.00103	0.00086	0.00139
8.5E-06	0.00314	0.0011	0.0006	0.00095	0.00115
8.5E-06	0.00227	0.0009	0.00048	-5E-05	0.001
8.5E-06	0.00269	0.00084	0.00072	-9E-05	0.00105
8.6E-06	0.00108	0.00071	-0.0007	0.00071	0.00193
8.6E-06	0.00092	0.00086	0.00062	0.00052	0.00106
8.6E-06	0.00176	0.00113	0.0007	-0.0007	0.00066
8.7E-06	0.00224	0.00116	0.00058	0.00036	0.00094
8.7E-06	0.00203	0.00087	0.00095	0.00125	0.00093
8.7E-06	0.00125	0.00105	0.00052	7.2E-05	0.00059
8.8E-06	0.00188	0.00159	0.00067	0.00047	0.00132
8.8E-06	0.0016	0.00063	0.00016	0.00014	0.00036
8.8E-06	0.00129	0.00126	0.00036	-0.0005	0.00141
8.9E-06	0.00106	0.00055	0.00119	0.00091	0.00091
8.9E-06	0.00171	0.00139	7.5E-06	0.00116	0.00078
8.9E-06	0.00128	0.00152	0.0012	0.00055	0.0019
9E-06	0.00219	0.00071	0.00127	0.0006	0.0007
9E-06	0.00179	0.00121	0.00101	0.00105	0.00097
9E-06	0.00164	0.00083	0.00157	-0.0005	0.00076
9.1E-06	0.00118	0.00069	-4E-05	-3E-05	0.00088
9.1E-06	0.00182	0.00088	0.00065	0.00017	0.00128
9.1E-06	0.00288	0.00111	0.00011	0.00035	0.00105
9.2E-06	0.00178	0.00041	0.00047	0.00079	0.00162
9.2E-06	0.00077	0.00064	0.00025	0.00044	0.00159

9.2E-06	0.00202	0.00163	0.00098	0.00048	0.00164
9.2E-06	0.00216	0.00191	0.00049	0.00016	0.00071
9.3E-06	0.00216	0.00174	0.00092	0.00021	0.0002
9.3E-06	0.00165	0.00154	0.00055	0.00071	0.0009
9.3E-06	0.00099	0.00099	0.00064	0.00023	0.00146
9.4E-06	0.00171	0.00126	3.7E-05	-6E-05	0.00115
9.4E-06	0.0021	0.00079	0.00102	0.00074	0.00144
9.4E-06	0.00082	0.00058	0.00063	0.00072	0.00174
9.5E-06	0.00131	0.00029	0.00091	0.00075	0.0006
9.5E-06	0.00216	-0.0002	0.00024	0.00034	0.00079
9.5E-06	0.00133	8.5E-05	0.00071	0.00036	0.00119
9.6E-06	0.00124	0.00062	0.00093	0.00057	0.00063
9.6E-06	0.00205	0.00161	0.00032	0.00049	0.00155
9.6E-06	0.00219	0.00113	0.00085	0.00048	0.00093
9.7E-06	0.00204	0.00062	0.00011	0.00074	0.00136
9.7E-06	0.00233	0.00036	0.00045	0.00072	0.00101
9.7E-06	0.00177	0.0011	0.00024	0.00112	0.00024
9.8E-06	0.00166	0.00079	0.0009	0.00143	0.0017
9.8E-06	0.00187	7.7E-05	0.00107	0.00126	0.00096
9.8E-06	0.00162	0.00112	0.0001	0.00141	0.00061
9.9E-06	0.00123	0.0013	0.0007	0.00155	0.00054
9.9E-06	0.00166	0.00078	0.00084	0.00083	0.0011
9.9E-06	0.00231	0.00058	0.0002	0.00077	0.00154
1E-05	0.00214	0.00066	0.00052	0.00058	0.00136
1E-05	0.00209	0.00099	0.00086	0.00113	0.00162
1E-05	0.00245	0.00106	-0.0005	0.00082	0.00148
1E-05	0.0027	0.0012	0.00089	0.00102	0.00163
1E-05	0.00197	0.00059	0.0007	0.00065	0.00063
1E-05	0.00169	0.00053	0.00152	0.00161	0.00166
1E-05	0.00231	0.00061	0.00062	0.00076	0.00078
1E-05	0.00159	0.00019	0.00158	0.00075	0.00079
1E-05	0.00142	0.0011	0.00117	0.00122	0.00151
1E-05	0.00115	0.00091	0.00101	0.00068	0.00122
1E-05	0.00206	0.00082	0.00072	0.00063	0.00119
1E-05	0.00223	0.00135	0.00115	0.00076	0.00124
1E-05	0.00211	0.00101	0.00136	0.00031	0.00134
1E-05	0.00201	0.00096	0.00089	0.00104	0.00124
1E-05	0.00172	0.00097	0.00069	0.00085	0.00109
1E-05	0.00145	0.00032	0.00133	0.00052	0.00127
1E-05	0.00201	0.00058	0.00075	0.00073	0.00065
1.1E-05	0.00179	0.00063	0.00102	0.0014	0.0008
1.1E-05	0.00147	0.00055	0.00095	0.00096	0.00047
1.1E-05	0.00216	0.001	0.0007	0.0009	0.00165
1.1E-05	0.002	0.0008	0.00047	0.00026	0.00137
1.1E-05	0.00183	0.00091	0.00011	0.00082	0.00113
1.1E-05	0.00184	0.00067	0.00079	0.00029	0.00064
1.1E-05	0.0025	-0.0002	0.00036	0.0003	0.00122
1.1E-05	0.0018	0.00093	0.0008	0.00016	0.00162
1.1E-05	0.0016	8E-05	0.00045	0.00027	0.00171
1.1E-05	0.00165	0.00094	0.00057	0.00131	0.0014
1.1E-05	0.00162	0.00071	0.00083	0.00041	0.00148
1.1E-05	0.00204	0.00045	0.00025	0.00072	0.00091
1.1E-05	0.00086	0.00042	0.00059	0.00044	0.00219
1.1E-05	0.00296	0.00092	0.00068	0.00118	0.00096

1.1E-05	0.00167	0.00036	0.00104	-1E-04	0.00131
1.1E-05	0.00214	0.00107	0.00104	-0.0002	0.00111
1.1E-05	0.00218	0.00155	0.00107	0.00072	0.00083
1.1E-05	0.00111	0.00064	0.00059	0.0003	0.00144
1.1E-05	0.00127	0.00035	0.00106	0.00069	0.00124
1.1E-05	0.00231	0.00144	0.00109	-0.0007	0.00172
1.1E-05	0.0019	0.00113	0.00054	0.00037	0.00011
1.1E-05	0.00146	0.00066	0.00085	0.0003	0.00107
1.1E-05	0.00134	0.00049	0.00065	-0.0001	0.00065
1.1E-05	0.0017	0.00118	0.00109	0.0006	0.00024
1.1E-05	0.00233	0.00049	0.00072	0.00066	0.00015
1.1E-05	0.00205	0.00057	-0.0002	0.00159	0.00125
1.1E-05	0.00223	0.00077	0.00118	0.0008	0.00101
1.1E-05	0.0018	0.00114	0.00093	-0.0008	0.00036
1.1E-05	0.00207	0.00063	0.00084	0.00018	0.00044
1.1E-05	0.00164	0.00032	0.00104	0.00012	0.00084
1.1E-05	0.00216	0.00026	0.00071	0.00021	0.00097
1.1E-05	0.00139	0.00086	0.00038	-0.0005	0.00122
1.2E-05	0.00222	0.00083	0.0012	3E-05	0.00087
1.2E-05	0.00176	0.00064	0.00022	0.00086	0.00059
1.2E-05	0.00136	0.00065	0.00039	0.00076	0.00085
1.2E-05	0.00169	0.00113	0.00069	0.00014	0.00083
1.2E-05	0.00138	0.00071	0.00023	-0.0001	0.00162
1.2E-05	0.00183	0.00098	0.00067	0.00081	0.00106
1.2E-05	0.00228	0.00065	0.0003	0.00078	0.00098
1.2E-05	0.00177	0.00075	0.00022	-0.0002	0.00102
1.2E-05	0.00062	0.00022	0.00063	0.00012	0.00104
1.2E-05	0.00199	0.00112	0.00139	0.00019	0.0011
1.2E-05	0.00206	0.00142	-0.0002	0.00038	0.00129
1.2E-05	0.00164	0.00039	0.00044	0.0004	0.00132
1.2E-05	0.00201	0.00044	0.00105	0.00056	0.00144
1.2E-05	0.00164	0.00077	0.00046	0.00022	0.00087
1.2E-05	0.00203	0.00118	0.0005	0.0004	0.00208
1.2E-05	0.00134	0.00089	0.0012	0.00031	0.00182
1.2E-05	0.00258	2.4E-05	-0.0002	-0.0004	0.00094
1.2E-05	0.00087	0.00063	0.00041	0.00036	0.00086
1.2E-05	0.0018	0.00122	-8E-05	0.00011	0.00195
1.2E-05	0.00186	0.00045	0.00116	0.00088	0.0015
1.2E-05	0.00151	0.00097	0.00057	0.00055	0.00241
1.2E-05	0.00202	0.00089	0.00019	0.00129	0.00055
1.2E-05	0.0009	0.00051	-9E-05	0.0002	0.00115
1.2E-05	0.00183	8.5E-05	0.00013	0.00056	0.00096
1.2E-05	0.00198	0.00123	0.00061	0.0005	0.00095
1.2E-05	0.00133	0.00062	0.0003	0.00036	0.0015
1.2E-05	0.00143	0.00079	0.00066	0.00104	0.00161
1.2E-05	0.00092	0.00094	-0.0003	0.00057	0.00219
1.2E-05	0.00251	0.00121	0.00054	0.00112	0.0018
1.2E-05	0.00174	0.00166	0.00027	-0.0005	0.00024
1.2E-05	0.00154	0.00087	0.00044	0.00047	0.00035
1.3E-05	0.00137	0.00075	0.00064	0.0011	0.0015
1.3E-05	0.00146	0.00062	-0.0002	0.00032	0.00047
1.3E-05	0.00245	0.0015	0.00013	-0.0001	0.00149
1.3E-05	0.00161	0.00033	0.00052	0.00071	0.00087
1.3E-05	0.00196	0.00066	-0.0003	0.0001	0.00069

1.3E-05	0.0017	0.00087	-0.0002	0.00067	0.00146
1.3E-05	0.00194	0.00066	0.0002	0.00043	0.00103
1.3E-05	0.00112	0.00078	0.0004	0.00067	0.0012
1.3E-05	0.00209	0.00022	0.00045	-8E-05	0.00126
1.3E-05	0.00117	0.00125	0.00027	0.00088	-0.0001
1.3E-05	0.00178	0.00112	0.00014	0.0004	0.00081
1.3E-05	0.00143	0.00026	-0.0001	0.0004	0.00129
1.3E-05	0.00169	0.00079	-0.0001	0.00085	0.00113
1.3E-05	0.00191	0.00039	0.00083	0.00055	0.00125
1.3E-05	0.00089	2E-05	-0.0002	0.00144	0.00171
1.3E-05	0.00171	-2E-05	-0.0002	0.00121	0.0015
1.3E-05	0.00198	-0.0003	0.0006	0.00079	0.00197
1.3E-05	0.00037	0.00067	0.00072	0.00042	0.00173
1.3E-05	0.00206	0.00073	-0.0003	0.00044	0.00099
1.3E-05	0.00191	0.00089	0.00015	0.00066	0.00173
1.3E-05	0.00168	0.00152	0.00041	-6E-05	0.00082
1.3E-05	0.0014	0.00068	0.00038	0.00013	0.00024
1.3E-05	0.00158	0.00151	0.00089	0.00141	0.00072
1.3E-05	0.00192	0.00094	0.00016	0.00104	0.00139
1.3E-05	0.00164	0.00093	0.00082	0.0008	0.00098
1.3E-05	0.00164	0.00105	0.00045	0.00057	0.00152
1.3E-05	0.00097	0.00056	0.00044	0.00075	0.00101
1.3E-05	0.00136	0.00128	0.00012	0.00064	0.00147
1.3E-05	0.00183	0.00095	0.00027	0.00024	0.00138
1.3E-05	0.00126	0.00111	-5E-05	0.00034	0.00102
1.3E-05	0.00111	0.00102	0.00019	0.00066	0.00125
1.4E-05	0.00195	0.00073	-4E-05	0.00054	0.00206
1.4E-05	0.00124	0.00079	-0.0001	0.00032	0.001
1.4E-05	0.00243	0.00089	0.00035	0.00056	0.0016
1.4E-05	0.00183	0.00022	0.00046	0.00159	0.00168
1.4E-05	0.0021	0.00095	0.00072	0.00032	0.0016
1.4E-05	0.00199	0.00076	0.00065	-0.0001	0.0007
1.4E-05	0.00164	0.00103	8.2E-05	0.00093	0.00105
1.4E-05	0.00177	0.00047	0.00029	0.00022	0.00107
1.4E-05	0.00098	0.00108	7.2E-05	0.00079	0.00084
1.4E-05	0.00148	0.00031	-4E-05	0.00128	0.00091
1.4E-05	0.00169	0.00069	0.00031	0.00108	0.00053
1.4E-05	0.00116	0.00083	0.00133	-0.0001	0.00049
1.4E-05	0.00187	-0.0002	0.00063	-1E-04	0.00062
1.4E-05	0.0024	0.00118	-0.0003	0.00051	0.00128
1.4E-05	0.00139	0.00114	-0.0002	0.00078	0.00085
1.4E-05	0.0012	0.00027	0.00013	0.0008	0.00105
1.4E-05	0.00179	0.00061	6.4E-06	0.0003	0.0016
1.4E-05	0.00109	0.00104	0.00048	0.00058	0.00056
1.4E-05	0.00206	0.00018	0.00095	0.00018	0.00145
1.4E-05	0.00147	0.00042	0.00034	0.0004	0.00146
1.4E-05	0.00046	0.00084	0.00082	0.00068	0.00119
1.4E-05	0.00139	0.00055	0.00119	-0.0002	0.00038
1.4E-05	0.00129	0.00161	0.00012	0.0002	0.00122
1.4E-05	0.0018	0.00098	0.00013	0.00029	0.00065
1.4E-05	0.00068	0.0005	-5E-05	0.00045	0.00116
1.4E-05	0.00214	0.00101	0.00074	0.00069	0.00065
1.4E-05	0.00091	0.00138	0.00046	0.0006	0.00135
1.4E-05	0.00129	0.00162	-0.0003	0.00052	0.00117

1.4E-05	0.00085	6.9E-05	0.00047	0.00047	0.00173
1.4E-05	0.0014	0.00114	0.00024	0.00019	0.00059
1.4E-05	0.00176	-9E-05	0.00046	0.00123	0.00093
1.5E-05	0.00106	0.00032	0.00029	0.00077	0.00104
1.5E-05	0.00091	0.0006	0.00078	0.00043	0.00109
1.5E-05	0.00167	0.00103	0.00064	-0.0004	0.00089
1.5E-05	0.00086	0.00082	-0.0002	-0.0002	0.00174
1.5E-05	0.00094	0.00033	0.00025	0.00025	0.00119
1.5E-05	0.00175	0.00081	0.00033	-2E-05	0.00083
1.5E-05	0.00188	0.00132	0.00112	0.00113	0.0005
1.5E-05	0.00103	0.00082	0.00036	0.00039	0.001
1.5E-05	0.00132	0.00116	0.00019	0.00039	0.00037
1.5E-05	0.00152	0.00073	0.00065	0.00057	0.00162
1.5E-05	0.00158	0.00035	0.00043	0.00023	0.00097
1.5E-05	0.00084	0.00058	3.1E-05	9.1E-05	0.00218
1.5E-05	0.00105	0.00045	0.00074	0.0006	0.00144
1.5E-05	0.00113	0.00033	0.00027	0.00031	0.00144
1.5E-05	0.00104	-0.0001	0.0003	-0.0002	0.00066
1.5E-05	0.0017	0.00114	0.0008	-5E-05	0.0003

Table SI.3A: Primary kinetic data for the reaction of 1-methyl-2-pyrrolidone (**S3**) with CumO• generated by 355 nm LFP in MeCN (**Figure SI.3A**).

**t / s**	**ΔA 1-methyl-2-pyrrolidone (S3)**				
	**0.077 M**	**0.154M**	**0.229M**	**0.303M**	**0.400M**
-1E-06	0.00025	0.00025	0.0001	4.6E-05	-0.0006
-1E-06	0.00022	0.00066	-8E-05	-0.0004	-8E-05
-9E-07	0.00054	0.0005	-1E-04	0.00108	-0.0007
-9E-07	-0.0001	0.00016	8.3E-05	-1E-05	-0.0007
-9E-07	0.00025	-0.0003	6E-05	-0.0003	-0.0007
-8E-07	0.00017	0.00018	0.00036	0.00052	-0.0002
-8E-07	8.4E-05	0.00045	-9E-05	0.00035	-0.0003
-8E-07	-0.0003	0.00062	-2E-05	0.00051	-0.0005
-7E-07	-9E-05	0.00076	-0.0001	0.00072	-0.0005
-7E-07	0.00023	0.00089	-0.0005	-0.0003	-0.0004
-7E-07	0.00017	0.00042	0.00022	-6E-05	0.00019
-6E-07	-0.0002	0.00031	-0.0006	0.00055	-0.0003
-6E-07	0.00019	0.00049	-0.0002	0.00075	0.00018
-6E-07	-0.0002	0.00021	0.00016	0.00081	-1E-04
-5E-07	0.0002	0.00042	-0.0002	0.00023	0.00034
-5E-07	0.0006	0.00044	-0.0001	0.00042	-0.001
-5E-07	-0.0006	0.00021	0.0002	-0.0009	-0.0003
-4E-07	3.7E-05	0.00043	0.00053	0.00044	-0.0003
-4E-07	-0.0005	0.00024	0.00013	0.00037	-0.0004
-4E-07	0.00038	0.00034	-0.0004	0.00091	-0.0005
-4E-07	0.00036	0.00051	0.00039	0.0001	-0.0007
-3E-07	-0.0002	0.0004	-0.0002	0.00035	-0.0012
-3E-07	-0.0002	0.00047	-0.0001	0.00076	-0.0002
-3E-07	0.00039	0.00084	-0.0001	-0.0001	-0.0009
-2E-07	-6E-05	0.00021	-0.0004	0.00044	-0.0006
-2E-07	0.00017	0.00038	0.00029	0.00041	-0.0006
-2E-07	4E-05	-0.0003	-0.0003	0.0006	-0.0008
-1E-07	-0.0009	0.00045	-0.0004	0.00066	-0.0005
-1E-07	-0.0006	0.00018	-0.0002	0.00085	-0.0003
-6E-08	0.00022	0.00041	-0.0005	0.00074	-0.0003
-3E-08	-0.0002	0.00022	-0.0011	0.00038	-0.0001
-5E-10	-0.0031	-0.0022	-0.0037	-0.0023	-0.005
3.1E-08	-0.0124	-0.0096	-0.0148	-0.0125	-0.0202
6.3E-08	0.01087	0.01103	0.01045	0.01011	0.00864
9.5E-08	0.01335	0.01416	0.01271	0.01341	0.01094
1.3E-07	0.01275	0.01305	0.01299	0.01349	0.01131
1.6E-07	0.01298	0.01223	0.01188	0.0126	0.01005
1.9E-07	0.01248	0.01221	0.01212	0.01134	0.00943
2.2E-07	0.01238	0.01196	0.01118	0.01113	0.00928
2.6E-07	0.01176	0.01092	0.01065	0.00994	0.00828
2.9E-07	0.01083	0.01115	0.00963	0.00959	0.00722
3.2E-07	0.01108	0.01003	0.00951	0.00909	0.00653
3.5E-07	0.01038	0.00971	0.00836	0.00729	0.00576
3.8E-07	0.00978	0.00935	0.0082	0.00723	0.00541
4.2E-07	0.00946	0.00946	0.00882	0.007	0.00558
4.5E-07	0.00976	0.00882	0.00778	0.00705	0.00529
4.8E-07	0.00839	0.00878	0.00694	0.00682	0.00471
5.1E-07	0.00905	0.00802	0.00708	0.00632	0.00431
5.4E-07	0.00917	0.00816	0.00606	0.00575	0.0045

5.8E-07	0.00753	0.00776	0.00615	0.00464	0.0035
6.1E-07	0.00771	0.00712	0.00608	0.00496	0.00326
6.4E-07	0.00766	0.00668	0.00496	0.00545	0.0034
6.7E-07	0.00739	0.00634	0.00528	0.00492	0.00316
7E-07	0.0077	0.00648	0.0046	0.00447	0.00302
7.4E-07	0.00678	0.00619	0.00456	0.004	0.00266
7.7E-07	0.00709	0.00543	0.00472	0.00454	0.00224
8E-07	0.00702	0.0057	0.00453	0.00299	0.00219
8.3E-07	0.00695	0.00554	0.00415	0.00343	0.00179
8.6E-07	0.00645	0.00577	0.00356	0.00242	0.0022
9E-07	0.00609	0.00429	0.00279	0.0024	0.00193
9.3E-07	0.00513	0.00509	0.00294	0.00281	0.00124
9.6E-07	0.00533	0.00504	0.00354	0.00349	0.00125
9.9E-07	0.00511	0.00481	0.00246	0.00272	0.00123
1E-06	0.00506	0.00453	0.00277	0.00295	0.0014
1.1E-06	0.00528	0.00367	0.0017	0.00279	0.0018
1.1E-06	0.00562	0.00408	0.00298	0.00264	0.00115
1.1E-06	0.0048	0.00444	0.00283	0.00262	0.00121
1.2E-06	0.00458	0.0043	0.002	0.00242	0.00107
1.2E-06	0.00501	0.00369	0.00274	0.00181	0.0012
1.2E-06	0.00478	0.00346	0.0019	0.0018	0.00076
1.2E-06	0.00377	0.00346	0.00138	0.00193	0.00069
1.3E-06	0.00375	0.00356	0.00174	0.00139	0.00059
1.3E-06	0.00385	0.00309	0.00208	0.00163	0.00135
1.3E-06	0.00382	0.00316	0.00081	0.00141	0.00011
1.4E-06	0.00361	0.00353	0.00136	0.00195	0.00023
1.4E-06	0.00355	0.00317	0.00195	0.00144	0.001
1.4E-06	0.00331	0.00294	0.00219	0.00159	0.00099
1.5E-06	0.00362	0.00296	0.0009	0.00153	-0.0002
1.5E-06	0.00347	0.00255	0.00125	0.00139	0.00071
1.5E-06	0.0031	0.00273	0.00156	0.00202	0.0007
1.6E-06	0.00316	0.00287	0.00163	0.00121	0.00045
1.6E-06	0.00393	0.00304	0.00139	0.00151	0.00011
1.6E-06	0.00335	0.00237	0.00137	0.00175	0.0005
1.7E-06	0.00276	0.00284	0.00131	0.00138	0.00102
1.7E-06	0.00241	0.00248	0.00133	0.00107	0.00062
1.7E-06	0.00191	0.00187	0.0012	0.00114	0.00011
1.8E-06	0.00279	0.0024	0.00145	0.00141	0.00022
1.8E-06	0.00211	0.00228	0.00027	0.00085	-0.0002
1.8E-06	0.00267	0.00273	0.00109	0.00068	0.00038
1.9E-06	0.00177	0.00242	0.00061	0.0007	0.00024
1.9E-06	0.00257	0.00164	0.00075	0.00075	-0.0008
1.9E-06	0.00199	0.00258	0.00187	0.00176	-0.0003
2E-06	0.00163	0.00205	0.00069	0.00098	-0.0004
2E-06	0.00253	0.00235	0.00095	0.00074	0.00015
2E-06	0.00288	0.00228	0.00095	0.00113	-0.0002
2E-06	0.00212	0.00224	0.00132	0.00108	0.00057
2.1E-06	0.00135	0.00175	-0.0004	0.00131	0.00021
2.1E-06	0.00197	0.00188	0.00082	0.00046	0.00043
2.1E-06	0.00168	0.00153	0.00094	0.00105	-0.0005
2.2E-06	0.00102	0.00155	0.00092	0.00082	0.00037
2.2E-06	0.00177	0.00217	0.00068	0.00115	0.00012
2.2E-06	0.00217	0.00182	0.00036	0.00122	0.00021
2.3E-06	0.00159	0.00133	0.00068	0.00089	-0.0004

2.3E-06	0.00161	0.00168	0.00021	0.00052	0.00034
2.3E-06	0.00126	0.00201	0.00145	0.00058	0.00106
2.4E-06	0.00145	0.00214	-1E-04	0.00078	0.00089
2.4E-06	0.00219	0.0017	0.00084	0.00033	0.00042
2.4E-06	0.0014	0.00163	0.00058	0.00055	0.00044
2.5E-06	0.00248	0.00142	9E-05	0.00045	0.0006
2.5E-06	0.00164	0.00117	0.00047	0.00085	0.00038
2.5E-06	0.00146	0.00144	0.00038	0.00027	0.00057
2.6E-06	0.00172	0.00185	0.00127	-2E-05	0.00035
2.6E-06	0.00129	0.00167	0.00127	0.00098	-0.0003
2.6E-06	0.00118	0.00205	0.00034	0.00111	-0.0001
2.7E-06	0.00041	0.00148	0.00021	0.00138	0.00021
2.7E-06	0.00144	0.00186	0.00072	0.00073	0.00053
2.7E-06	-0.0003	0.0016	0.0009	0.00034	0.00072
2.8E-06	0.00105	0.00245	0.00105	-4E-05	0.00017
2.8E-06	0.00107	0.00152	0.00043	0.00038	-0.0003
2.8E-06	0.00118	0.00158	0.00016	0.00059	-0.0002
2.8E-06	0.00131	0.00148	-0.0005	0.00056	0.00038
2.9E-06	0.00112	0.00123	0.00027	0.00038	-0.0002
2.9E-06	0.00077	0.00116	0.00061	0.00113	0.00025
2.9E-06	0.00113	0.00168	8.1E-05	0.00101	0.00016
3E-06	0.00127	0.00223	0.00035	0.00127	4E-05
3E-06	0.00113	0.00131	0.00078	0.00067	0.00094
3E-06	0.00092	0.00189	0.0002	0.00034	0.00064
3.1E-06	0.00117	0.00178	0.00106	-0.0002	0.00049
3.1E-06	0.00141	0.00182	-0.0004	0.00024	0.00061
3.1E-06	0.00174	0.00148	0.00035	0.00133	0.00065
3.2E-06	0.00066	0.0014	-0.0004	0.00137	-0.0003
3.2E-06	0.00117	0.00086	0.00098	0.00067	0.0006
3.2E-06	0.00083	0.00196	4.3E-05	6.5E-05	0.00013
3.3E-06	0.0012	0.00195	0.00115	0.00125	0.00076
3.3E-06	0.00042	0.00144	0.00078	0.00089	0.00041
3.3E-06	0.00115	0.00114	-0.0008	0.00092	-0.0008
3.4E-06	0.00079	0.00193	-0.0005	0.00078	0.00012
3.4E-06	0.00096	0.00127	0.00098	0.00113	-0.0002
3.4E-06	0.00084	0.00117	0.00035	0.00071	0.00037
3.5E-06	0.00171	0.00144	0.00054	0.00099	-0.0002
3.5E-06	0.00061	0.00178	-0.0003	0.00112	0.00035
3.5E-06	-6E-05	0.00162	0.00034	0.00056	0.00066
3.6E-06	0.00068	0.00129	0.00046	0.00042	-0.0005
3.6E-06	0.00095	0.00182	0.00067	0.00058	0.00026
3.6E-06	0.00108	0.00086	9E-06	0.00011	0.00021
3.6E-06	0.00025	0.00109	-9E-05	0.00011	0.00021
3.7E-06	4.7E-05	0.00156	0.00087	0.00105	-0.0001
3.7E-06	0.00012	0.00164	0.00069	0.00081	-0.0001
3.7E-06	0.00033	0.00139	-0.0005	0.00059	0.00031
3.8E-06	0.00125	0.00142	0.00013	0.00022	-0.0009
3.8E-06	0.00046	0.00095	0.00031	0.00016	0.00027
3.8E-06	0.0011	0.00128	0.00069	0.00057	-2E-06
3.9E-06	-8E-05	0.00171	0.00071	0.00033	0.00064
3.9E-06	0.00084	0.00184	0.00057	0.00067	-0.0005
3.9E-06	0.00019	0.00129	0.00064	0.00134	0.00059
4E-06	0.00133	0.0013	-0.0002	0.00111	0.00071
4E-06	0.00072	0.00104	0.00112	0.00059	-0.0003

4E-06	-0.0002	0.00086	-0.0001	0.00077	-0.0002
4.1E-06	8E-05	0.00134	-0.0008	5.1E-05	0.00057
4.1E-06	0.0017	0.00167	8.5E-05	0.00046	4.8E-05
4.1E-06	0.00049	0.00094	-0.0003	0.00055	0.00021
4.2E-06	0.00024	0.00126	0.00033	0.00022	0.00016
4.2E-06	0.00106	0.00174	0.00068	-5E-05	0.00045
4.2E-06	0.00038	0.00197	1.8E-05	0.00055	-0.0009
4.3E-06	0.00117	0.00232	0.00078	-0.0004	-9E-05
4.3E-06	0.00068	0.00126	0.00063	0.00041	0.00021
4.3E-06	-0.0004	0.00162	0.00029	0.00056	0.00036
4.4E-06	0.00068	0.00165	0.00058	0.00049	-7E-06
4.4E-06	0.00057	0.00096	0.00015	-2E-05	0.0003
4.4E-06	7.1E-05	0.00107	0.0002	0.00012	0.00024
4.4E-06	0.00073	0.00144	0.00061	0.00044	0.00073
4.5E-06	-0.0001	0.00111	-0.0002	0.0006	-1E-05
4.5E-06	-0.0001	0.00208	-0.0001	0.00078	-0.0008
4.5E-06	0.00024	0.00103	0.00087	0.00073	-0.0003
4.6E-06	0.00125	0.00079	0.00053	0.00024	-0.0006
4.6E-06	0.00075	0.00076	3.5E-05	0.0012	0.00014
4.6E-06	0.0004	0.00111	0.00012	0.00071	-0.0001
4.7E-06	0.00097	0.00061	0.00054	0.00076	0.00075
4.7E-06	0.00062	0.00098	0.00011	-0.0003	0.00019
4.7E-06	0.00054	0.00177	-0.0008	-0.0002	0.00038
4.8E-06	0.00088	0.0016	0.0007	0.00042	0.0004
4.8E-06	0.00078	0.00169	0.00081	5.7E-05	1.8E-05
4.8E-06	-0.0004	0.00147	0.00026	0.00049	0.00027
4.9E-06	0.00031	0.00074	0.00047	0.00086	2E-05
4.9E-06	0.00119	0.00156	0.00044	0.0008	0.00052
4.9E-06	0.00031	0.00126	0.00048	0.00101	0.00026
5E-06	0.00016	0.00098	-0.0004	0.00043	0.00021
5E-06	0.00012	0.00113	-0.0001	0.00028	0.00053
5E-06	5E-05	0.00063	-0.0005	0.00073	0.00014
5.1E-06	-0.0003	0.0017	0.00029	0.00082	-0.0002
5.1E-06	-9E-06	0.00191	0.00051	0.00064	0.0001
5.1E-06	0.00043	0.00166	-0.0002	0.00037	0.00064
5.2E-06	0.00071	0.00169	0.00039	0.00078	0.00028
5.2E-06	-0.0001	0.00131	0.00017	-0.0003	0.00047
5.2E-06	0.00097	0.00079	0.00045	0.00037	0.0001
5.2E-06	0.00023	0.00135	0.00012	-0.0002	0.00056
5.3E-06	-0.0003	0.00135	-0.0002	0.00086	-4E-05
5.3E-06	0.00014	0.00149	0.00076	0.00024	-0.0009
5.3E-06	-7E-06	0.00142	0.0002	0.00065	0.00055
5.4E-06	0.00034	0.00076	0.00117	0.00038	0.00012
5.4E-06	0.00073	0.00037	0.00021	0.00079	0.00015
5.4E-06	0.00063	0.00111	0.00059	-0.0001	0.00062
5.5E-06	0.00075	0.00138	0.00051	0.00066	3E-05
5.5E-06	0.00075	0.00154	-0.0002	0.00096	0.00058
5.5E-06	0.0004	0.00116	0.00064	0.0006	0.00016
5.6E-06	0.00065	0.00094	0.00093	0.00112	-0.0001
5.6E-06	0.00097	0.00118	0.00027	0.00104	-0.0002
5.6E-06	0.00032	0.00104	0.00014	-0.0004	0.0008
5.7E-06	0.00047	0.00116	0.00064	7.9E-05	0.00071
5.7E-06	0.00095	0.00156	0.00017	0.00102	0.00049
5.7E-06	0.00102	0.00102	-5E-05	0.00096	0.00084

5.8E-06	0.00032	0.00126	0.00023	0.00061	0.00014
5.8E-06	0.00048	0.00099	-7E-05	0.00048	0.00062
5.8E-06	9.6E-05	0.00138	0.00068	0.00036	0.00015
5.9E-06	0.00045	0.00128	0.00035	0.00064	0.0003
5.9E-06	0.00068	0.00133	0.00107	0.00092	-0.0002
5.9E-06	0.00043	0.00155	0.00085	-0.0003	-0.0005
6E-06	0.00067	0.00073	0.00035	0.00026	0.00069
6E-06	0.00099	0.00102	0.00086	0.00017	-0.0002
6E-06	0.00022	0.00165	0.00123	0.0006	0.00018
6E-06	0.00047	0.00132	0.00041	-7E-07	0.00052
6.1E-06	-0.0003	0.0015	0.00023	0.00056	0.00018
6.1E-06	0.0005	0.00163	1.1E-05	0.00032	0.00018
6.1E-06	0.00094	0.00093	0.00017	-0.0005	-0.0003
6.2E-06	0.00036	0.00169	-0.0002	-1E-05	-0.0003
6.2E-06	0.00072	0.00103	-0.0001	0.00131	-0.0004
6.2E-06	0.00125	0.00149	0.00025	0.00097	0.00045
6.3E-06	0.00037	0.00103	0.00054	0.00046	0.00014
6.3E-06	0.00053	0.00076	0.00065	0.00091	-4E-05
6.3E-06	-0.0001	0.00113	0.00046	0.00077	-3E-05
6.4E-06	0.00025	0.00131	0.00069	0.00028	-6E-06
6.4E-06	2.3E-05	0.00138	-0.0004	0.00012	0.00038
6.4E-06	0.00029	0.00143	-0.0003	0.00054	-0.0003
6.5E-06	0.00083	0.00055	-0.0001	0.00082	0.00029
6.5E-06	0.00064	0.00138	-0.0003	0.0004	0.0001
6.5E-06	6.5E-05	0.00195	-0.0001	0.00024	0.00023
6.6E-06	0.00108	0.00131	2.3E-05	-0.0006	-0.0002
6.6E-06	0.00121	0.00121	0.00053	0.00045	0.00052
6.6E-06	0.00093	0.00101	0.00034	0.00018	0.00058
6.7E-06	0.00063	0.00125	-0.0002	0.00041	0.00066
6.7E-06	0.00076	0.00144	-0.0002	0.00047	-0.0005
6.7E-06	0.00057	0.00063	0.00038	-0.0003	-0.0003
6.8E-06	0.0002	0.0009	0.00071	0.00072	0.00051
6.8E-06	0.00062	0.00103	-0.0004	0.00046	0.00059
6.8E-06	0.0004	0.00091	-0.0005	0.00088	-1E-04
6.8E-06	0.00059	0.00153	0.00068	0.00012	1.1E-05
6.9E-06	0.00039	0.00156	0.00056	0.00042	0.00034
6.9E-06	-0.0008	0.0016	-0.0002	0.00075	7.4E-05
6.9E-06	0.00013	0.00114	0.0007	0.00026	0.00036
7E-06	-0.0003	0.00066	0.00036	0.00142	0.00049
7E-06	0.00063	0.00115	-1E-05	0.00073	0.00077
7E-06	-0.0006	0.00084	-0.001	0.00057	-0.0002
7.1E-06	0.00012	0.00054	5.6E-05	0.00105	-3E-06
7.1E-06	0.00031	0.00075	-0.0003	0.00149	0.0002
7.1E-06	0.00072	0.00094	7.1E-05	0.00024	0.00083
7.2E-06	0.00032	0.00087	0.00019	0.00065	-5E-05
7.2E-06	-0.0004	0.00131	0.00017	0.00078	0.00022
7.2E-06	6E-05	0.00155	0.00032	0.00049	4.6E-05
7.3E-06	0.00026	0.00091	0.00109	0.00063	0.00016
7.3E-06	0.00108	0.00045	-0.0003	0.00019	0.00018
7.3E-06	2.3E-05	0.00094	0.0002	0.00058	0.00077
7.4E-06	-5E-05	0.00106	0.00012	0.00118	0.00038
7.4E-06	0.00027	0.00119	6.9E-05	-5E-05	0.00092
7.4E-06	0.0006	0.00135	-0.0008	0.00071	0.00053
7.5E-06	0.00068	0.00156	5.7E-05	0.00012	-6E-05

7.5E-06	8.6E-05	0.00169	-0.0004	0.00046	0.00043
7.5E-06	-0.0002	0.00175	-0.0004	0.00126	0.00034
7.6E-06	3.9E-05	0.00065	-0.0003	0.00055	0.00042
7.6E-06	0.00059	0.0015	-0.0002	-3E-05	0.00021
7.6E-06	0.00039	0.00164	0.00059	0.00089	0.00064
7.6E-06	1.1E-05	0.00131	0.00072	0.00053	0.00081
7.7E-06	-0.0002	0.00091	-0.0007	0.00055	0.0007
7.7E-06	0.0005	0.00085	4.9E-05	2.8E-05	-0.0001
7.7E-06	0.00036	0.0014	0.00044	-0.0001	-0.0001
7.8E-06	0.00023	0.00104	0.00057	0.00098	0.00069
7.8E-06	0.00067	0.00135	0.00077	0.00062	0.0002
7.8E-06	-0.0002	0.001	-0.0002	0.00056	0.00039
7.9E-06	0.00012	0.00095	0.00058	0.00036	0.00061
7.9E-06	-0.0001	0.00067	-6E-05	0.00099	0.00043
7.9E-06	0.00071	0.00163	-0.0001	0.00082	0.00029
8E-06	-0.0005	0.00082	0.00047	0.00053	1.6E-05
8E-06	0.00018	0.00104	0.00046	-0.0003	4.2E-05
8E-06	0.00015	0.00119	-0.0003	0.00075	9.5E-05
8.1E-06	8.9E-05	0.00131	5E-05	-0.0003	0.00022
8.1E-06	0.00084	0.00135	-0.0006	0.00054	-3E-05
8.1E-06	0.00047	0.00057	0.00026	0.0003	0.00047
8.2E-06	-2E-05	0.00161	-0.0004	-0.0002	0.00088
8.2E-06	0.00088	0.00098	-0.0006	0.0004	0.00051
8.2E-06	-0.0006	0.00096	-0.0004	0.00074	0.0005
8.3E-06	0.00011	0.00167	0.00047	-0.0004	2.5E-05
8.3E-06	0.00078	0.00104	0.00075	0.00045	0.00056
8.3E-06	0.00063	0.00106	-0.0005	3.7E-05	-5E-06
8.4E-06	-0.0003	0.00106	0.00084	0.00074	0.0004
8.4E-06	5.6E-05	0.00113	0.00032	0.00037	-2E-05
8.4E-06	0.00038	0.00057	-0.0003	0.00059	0.00033
8.4E-06	4.3E-05	0.00072	-0.0001	-0.0001	0.00012
8.5E-06	0.00087	0.00089	-0.0001	0.00015	-0.0001
8.5E-06	0.0004	0.00114	-0.0005	0.0004	-0.0001
8.5E-06	0.00043	0.00109	-0.0001	-0.0002	-6E-05
8.6E-06	0.00028	0.00104	-4E-05	0.00051	2.5E-05
8.6E-06	0.00022	0.00103	-2E-05	0.00036	0.00029
8.6E-06	0.00013	0.00103	0.00048	0.00058	0.00061
8.7E-06	0.00078	0.00095	-0.0003	0.0013	0.00033
8.7E-06	-0.0006	0.00095	-0.0005	0.00122	0.00035
8.7E-06	0.00042	0.00107	0.00015	0.00062	1.4E-05
8.8E-06	0.00038	0.00051	-6E-05	0.0004	0.00023
8.8E-06	0.00072	0.0008	-0.0002	0.00043	0.0004
8.8E-06	4.9E-05	0.00054	-0.0004	0.00052	-0.0002
8.9E-06	6.6E-05	0.0009	-0.0009	0.0008	8.7E-05
8.9E-06	0.00012	0.00114	0.00049	0.00047	0.00028
8.9E-06	0.00089	0.00071	0.00042	0.00097	2.7E-05
9E-06	0.00018	0.00032	0.00019	0.00052	0.00024
9E-06	9.6E-05	0.00071	-0.0004	0.00076	0.00049
9E-06	0.00104	0.00055	-0.0003	0.00027	-0.0001
9.1E-06	0.00087	0.00073	5.6E-05	0.00017	0.00038
9.1E-06	-1E-05	0.00091	-0.0008	0.00022	2.9E-05
9.1E-06	0.00031	0.00063	-0.0002	0.0002	0.00042
9.2E-06	0.00057	0.00097	-2E-05	0.00078	0.00012
9.2E-06	-0.0002	0.00097	2E-05	-0.0003	0.00068

9.2E-06	0.00052	0.00032	-0.0002	0.00101	0.00028
9.2E-06	0.0004	0.00096	0.00019	4.1E-05	-9E-05
9.3E-06	1.7E-06	0.00075	2.8E-05	0.00106	0.0003
9.3E-06	-0.0004	0.00082	-5E-05	0.00055	0.0003
9.3E-06	0.00018	0.00094	-5E-05	0.00015	0.00018
9.4E-06	7.1E-06	0.00067	0.00032	-0.0002	0.00074
9.4E-06	-3E-05	0.00053	0.0001	0.001	0.00088
9.4E-06	0.00018	0.00065	0.00048	0.00024	0.00026
9.5E-06	-0.0003	0.00066	-0.0001	0.00086	0.00028
9.5E-06	0.00055	0.00084	-0.0013	0.00043	0.00021
9.5E-06	0.00055	0.0012	-0.0002	-4E-05	0.00091
9.6E-06	0.00079	0.00051	-0.0007	0.00042	0.00103
9.6E-06	-9E-05	0.00087	-0.0002	0.00083	0.00058
9.6E-06	0.00071	0.00163	0.00012	0.00018	0.00026
9.7E-06	-0.0003	0.00104	-8E-06	0.00023	0.00049
9.7E-06	-0.0002	0.00083	0.00081	0.00089	1.8E-05
9.7E-06	0.00048	0.00123	-0.0006	0.00068	-0.0003
9.8E-06	0.0009	0.00094	-0.0006	0.00097	-0.0003
9.8E-06	0.00033	0.00073	-0.0004	0.00095	0.00032
9.8E-06	0.00036	0.00085	0.00012	0.0006	0.00047
9.9E-06	0.00056	0.00057	0.00039	0.0007	0.0005
9.9E-06	-2E-05	0.00085	-0.0001	-1E-05	7.7E-05
9.9E-06	0.00017	0.00083	0.0003	0.00021	0.00048
1E-05	0.00095	0.00093	-1E-04	0.0008	-5E-05
1E-05	0.0006	0.00103	0.00025	-8E-05	0.00018
1E-05	0.00044	0.00018	8.6E-05	0.00072	0.00047
1E-05	-0.0001	0.00062	0.00035	0.00054	0.00053
1E-05	0.00013	0.00097	0.00076	0.0007	0.00037
1E-05	0.00034	0.00095	0.00012	0.00117	0.00015
1E-05	-0.0001	0.00086	0.00023	0.00081	-7E-05
1E-05	-0.0002	-9E-05	-0.0002	0.00033	-0.0001
1E-05	0.0002	0.00104	0.0005	0.00108	0.00069
1E-05	-4E-05	0.00103	-0.0002	0.00107	0.00021
1E-05	0.00031	0.00064	-0.0004	0.00038	0.00029
1E-05	-0.0001	0.00067	0.00012	0.00012	3E-05
1E-05	0.00021	0.00055	-4E-05	0.00062	0.00072
1E-05	-0.0004	0.00083	-6E-05	0.00058	0.00035
1E-05	0.00079	0.0009	-0.0003	2E-05	0.00021
1E-05	0.00067	0.00089	0.00016	0.00065	0.00025
1E-05	0.00036	0.0009	1.3E-05	0.00086	0.00018
1.1E-05	0.00054	0.00073	6.4E-05	-0.0002	0.00036
1.1E-05	0.00023	0.00091	0.00019	0.00018	0.00025
1.1E-05	-0.0005	0.00071	0.0001	0.0004	-0.0003
1.1E-05	0.00025	0.00086	-0.0002	0.00099	9.9E-05
1.1E-05	0.00012	0.00049	-0.0002	0.00045	0.00011
1.1E-05	4.3E-05	0.00042	0.00043	-0.0003	0.00044
1.1E-05	-0.0001	0.00074	-4E-05	6.7E-05	0.00023
1.1E-05	7.7E-05	0.0005	0.00024	0.0006	0.00047
1.1E-05	-0.0001	0.00073	-0.0001	0.00059	-0.0003
1.1E-05	-0.0001	0.00069	9.5E-05	0.00012	-1E-05
1.1E-05	-4E-05	0.00053	-0.0002	0.00103	-0.0005
1.1E-05	7.8E-05	0.00075	2.9E-05	0.00049	0.00014
1.1E-05	0.00038	0.00072	-7E-05	-0.0003	0.00022
1.1E-05	0.00016	0.00027	-0.0004	0.00095	-0.0003

1.1E-05	0.00017	0.00085	-4E-05	0.00038	0.0002
1.1E-05	-0.0003	0.0005	0.00028	0.00023	0.00025
1.1E-05	0.00061	0.00061	0.00072	0.00023	-0.0003
1.1E-05	0.00041	0.00068	0.00014	0.00029	0.00035
1.1E-05	0.00054	0.00083	-0.0003	0.0005	9.6E-05
1.1E-05	0.00039	0.00053	-0.0004	0.00026	0.00022
1.1E-05	-0.0002	0.00082	0.00019	0.00038	0.00108
1.1E-05	3.4E-05	0.00047	0.00017	0.00029	0.00013
1.1E-05	7.4E-06	0.00022	0.00042	0.00057	-0.0002
1.1E-05	0.00046	0.00077	-0.0007	0.00041	0.00048
1.1E-05	0.0003	0.00066	-0.0005	0.0004	-9E-05
1.1E-05	0.0002	0.00073	-7E-05	0.00076	0.00035
1.1E-05	-0.0005	0.00026	-8E-05	0.00022	-6E-05
1.1E-05	0.00069	0.00054	0.00011	0.00038	-0.0003
1.1E-05	0.00031	8.2E-05	-9E-05	7.7E-05	-8E-05
1.1E-05	-0.0002	0.00022	2.6E-05	0.00072	0.00044
1.1E-05	3.8E-05	0.00054	9.7E-05	-0.0006	-0.0003
1.1E-05	5.8E-05	0.00066	0.00061	-0.0001	0.00022
1.2E-05	0.00054	0.00079	-0.0001	0.00016	0.00045
1.2E-05	0.00073	0.0007	0.00033	0.00043	-0.0002
1.2E-05	1.9E-05	0.00052	-0.0006	-7E-05	-3E-05
1.2E-05	0.00053	0.00056	3.2E-05	0.00064	0.00028
1.2E-05	0.00018	0.00018	0.00015	-0.0002	0.00029
1.2E-05	0.0002	0.00026	0.0002	0.00044	0.00076
1.2E-05	0.00021	0.00041	0.00026	0.00041	0.00063
1.2E-05	0.00056	0.00074	0.00031	0.0003	-0.0001
1.2E-05	0.00038	0.00023	-0.0002	0.00122	0.00074
1.2E-05	0.00097	0.00068	0.00066	0.00086	9.9E-05
1.2E-05	-3E-05	0.0006	0.00011	0.00097	-0.0004
1.2E-05	-5E-05	0.00068	0.00047	-8E-05	0.00088
1.2E-05	9.9E-05	0.00052	-4E-05	8E-05	0.00034
1.2E-05	0.00043	0.00062	-7E-06	0.00099	-0.0002
1.2E-05	0.00017	0.00043	-0.0002	0.001	0.00011
1.2E-05	0.00065	0.00034	8.1E-05	-4E-05	0.00046
1.2E-05	0.00045	9.7E-05	-0.0001	0.00035	0.00025
1.2E-05	0.00053	0.00077	-0.0004	3.7E-05	0.00055
1.2E-05	0.00044	0.00021	-0.0004	0.00021	0.00115
1.2E-05	0.00022	0.00058	-0.0003	1.2E-05	0.00013
1.2E-05	5E-05	6.5E-05	-0.0005	0.00057	0.00023
1.2E-05	0.0006	0.00039	0.00017	0.00117	0.00034
1.2E-05	0.00037	0.00029	-0.0006	0.00086	-9E-05
1.2E-05	-0.0002	-0.0003	0.0003	0.00027	0.00028
1.2E-05	2.9E-05	0.00048	0.00022	0.00067	-0.0002
1.2E-05	0.00056	0.00027	-0.0005	-0.0001	0.00011
1.2E-05	0.00048	0.00027	3.5E-06	0.00032	0.00072
1.2E-05	-0.0003	0.00059	0.00079	0.00068	-0.0001
1.2E-05	0.00012	6.2E-05	-0.0004	-7E-05	-1E-05
1.2E-05	-0.0002	0.00048	0.00088	0.00092	0.00014
1.2E-05	0.00013	0.00056	-1E-04	7E-05	-4E-05
1.3E-05	0.00077	-0.0003	-0.0003	0.00033	0.00017
1.3E-05	0.00035	7.9E-05	-0.0002	0.00022	-0.0004
1.3E-05	0.00051	0.0005	0.0005	8.3E-05	1.3E-05
1.3E-05	0.00014	0.00064	0.00046	0.00051	0.0005
1.3E-05	0.00024	-0.0004	-0.0001	0.0005	-0.0004

1.3E-05	-0.0001	0.00018	-0.0002	9.8E-05	0.00012
1.3E-05	0.0003	0.0007	-0.0001	0.00032	-0.0008
1.3E-05	0.00035	0.00033	6E-05	0.00056	0.00012
1.3E-05	-0.0007	-0.0002	0.00037	-0.0002	0.00033
1.3E-05	4.8E-05	0.00025	0.00053	0.00018	0.00015
1.3E-05	-0.0001	0.00044	-0.0001	0.00026	0.00023
1.3E-05	0.00024	0.0004	0.00015	0.00092	0.0006
1.3E-05	0.0004	0.00036	0.00029	0.00034	-0.0003
1.3E-05	8.8E-05	-2E-05	-0.0005	0.00038	0.00055
1.3E-05	0.00038	-6E-05	-6E-05	0.00099	-6E-05
1.3E-05	0.0005	-0.0005	0.00046	-0.0002	0.00078
1.3E-05	-0.0002	0.00059	0.00017	2.7E-05	-0.0004
1.3E-05	-4E-05	0.00018	7.1E-05	0.00072	0.00018
1.3E-05	-0.0004	0.00052	5.1E-05	0.00135	0.00021
1.3E-05	-0.0003	0.00014	0.00022	0.00069	-6E-05
1.3E-05	0.00049	1E-05	-0.0005	0.00042	8.5E-06
1.3E-05	4.8E-05	2.5E-05	-0.0004	0.00026	-0.0006
1.3E-05	-0.0002	1.4E-05	-0.0008	0.00128	-0.0003
1.3E-05	0.00036	0.00039	5.3E-05	0.00047	0.00014
1.3E-05	0.00056	1E-04	-0.0003	1.4E-05	0.00043
1.3E-05	5E-05	-8E-05	1.5E-05	-1E-06	0.00024
1.3E-05	0.00023	5.6E-05	-0.0003	0.00092	-0.0005
1.3E-05	9.1E-05	0.00049	-0.0001	0.00018	0.00019
1.3E-05	0.00019	0.00021	-4E-05	0.00085	-0.0001
1.3E-05	0.00011	0.00046	-0.0007	0.00042	-0.0002
1.3E-05	0.00046	0.00027	0.00064	-0.0001	-0.0003
1.4E-05	1.3E-05	5.8E-05	0.00051	0.00025	0.00044
1.4E-05	-0.0001	0.00019	-0.0003	0.00093	-1E-04
1.4E-05	0.00011	0.00019	-2E-05	0.0007	0.00055
1.4E-05	0.0008	0.0004	6.2E-05	0.00021	-0.0007
1.4E-05	-4E-05	-0.0002	-0.0012	-0.0003	-4E-05
1.4E-05	0.00038	8.9E-05	0.00037	-0.0001	3.8E-05
1.4E-05	0.00017	0.00039	-5E-05	0.00032	9.3E-05
1.4E-05	0.00056	0.00068	0.00023	0.00072	-0.0004
1.4E-05	-0.0004	0.00028	-5E-05	-0.0003	-0.0002
1.4E-05	0.00049	-0.0002	-0.0002	0.00017	-9E-05
1.4E-05	1.2E-05	5E-05	-0.0007	-3E-05	-0.0002
1.4E-05	0.0002	3.8E-05	7E-06	0.00075	0.00087
1.4E-05	0.00013	0.00039	-0.0002	0.00029	4.7E-05
1.4E-05	8.2E-06	0.00045	-0.0002	-0.0003	0.00023
1.4E-05	9E-05	0.00034	0.00023	9.2E-05	-0.0002
1.4E-05	0.00048	0.0002	-0.0004	0.00036	5.1E-05
1.4E-05	0.00067	3.8E-05	0.00065	0.00016	0.00032
1.4E-05	0.00078	0.00019	-0.0004	0.00027	-0.0002
1.4E-05	0.00033	0.0001	-0.0004	1.5E-05	0.00043
1.4E-05	0.00033	0.00019	4.1E-05	0.0003	0.00015
1.4E-05	0.00048	-1E-05	0.00012	-9E-05	-0.0002
1.4E-05	0.00061	-0.0004	-0.0003	0.00033	3.8E-05
1.4E-05	0.00037	-0.0003	9.9E-05	0.00029	-5E-06
1.4E-05	0.00094	-8E-05	0.00051	-0.0004	0.0007
1.4E-05	5E-05	0.00015	-0.0005	0.00035	0.0005
1.4E-05	0.00019	0.00044	-0.0002	-0.0001	6E-05
1.4E-05	0.00084	-0.0005	0.00063	-8E-05	-0.0005
1.4E-05	-0.0003	0.00043	-0.0002	0.00082	0.00082

1.4E-05	0.00076	5.5E-05	-0.0001	0.00059	0.00037
1.4E-05	0.00069	-0.0001	-0.0004	3.4E-05	-8E-05
1.4E-05	0.00083	-8E-05	-7E-05	0.00014	7.4E-05
1.5E-05	-0.0002	8.9E-05	-0.0004	-0.0003	-0.0003
1.5E-05	0.00043	-0.0002	6E-05	-0.0003	-0.0002
1.5E-05	-0.0002	-0.0002	-3E-05	0.00114	7.4E-05
1.5E-05	0.0008	-0.0006	0.0004	0.00029	0.00019
1.5E-05	-0.0002	6.3E-06	-0.0006	0.00013	-4E-06
1.5E-05	8.1E-05	-0.0002	0.00029	0.00057	-0.0001
1.5E-05	-0.0004	5.7E-05	3.7E-05	-0.0002	0.00042
1.5E-05	0.00097	0.00013	-4E-05	0.00038	-0.0005
1.5E-05	0.00065	-0.0002	-0.0004	-0.0003	-6E-05
1.5E-05	0.00038	-0.0002	0.00055	0.00057	6.6E-05
1.5E-05	0.00028	0.00026	-0.0003	0.00061	0.00032
1.5E-05	3E-05	-0.0003	-0.0002	0.00017	-0.0001
1.5E-05	0.00022	0.00041	5.3E-05	0.00019	-0.0005
1.5E-05	-0.0006	-0.0001	-0.0004	0.00042	-0.0001
1.5E-05	0.00011	-7E-05	-0.0004	-0.0002	9.9E-05
1.5E-05	0.00023	0.00023	-0.0007	0.00017	-8E-05

Table SI.3B: Primary kinetic data for the reaction of 1-methyl-2-pyrrolidone (**S3**) with CumO$^{\bullet}$ generated by 355 nm LFP in DMSO (**Figure SI.3B**).

**t / s**	**ΔA 1-methyl-2-pyrrolidone (S3)**				
	**0.0517 M**	**0.103 M**	**0.154 M**	**0.204 M**	**0.253 M**
-9.92E-07	-1.22E-04	1.31E-04	-1.58E-04	-4.49E-04	8.33E-05
-9.60E-07	-6.90E-04	1.04E-03	-9.48E-05	-4.15E-04	1.55E-04
-9.28E-07	-2.25E-04	4.70E-04	-3.56E-05	-6.90E-04	2.45E-04
-8.96E-07	-7.82E-05	5.97E-04	-5.11E-04	-5.80E-04	6.67E-04
-8.64E-07	5.52E-05	6.09E-04	-4.68E-04	-2.31E-04	4.86E-04
-8.32E-07	4.92E-04	4.47E-04	4.15E-04	-4.99E-04	6.40E-04
-8.00E-07	5.82E-04	5.84E-04	-2.10E-04	-7.79E-04	7.43E-05
-7.68E-07	-9.57E-05	1.06E-03	-5.33E-05	-1.18E-03	2.48E-04
-7.36E-07	1.66E-04	6.24E-04	-1.13E-05	-2.37E-04	-5.37E-04
-7.04E-07	1.31E-04	4.70E-04	-2.24E-04	-1.31E-03	1.64E-04
-6.72E-07	3.14E-04	8.28E-04	7.02E-05	-1.24E-03	6.94E-04
-6.40E-07	5.59E-04	4.92E-04	-1.36E-04	-6.99E-04	6.22E-04
-6.08E-07	-2.51E-04	8.70E-04	-4.66E-04	-1.17E-03	-4.37E-05
-5.76E-07	4.94E-04	7.09E-04	-3.06E-04	-8.69E-04	1.50E-03
-5.44E-07	2.72E-04	5.47E-04	-1.91E-04	-9.04E-04	9.51E-04
-5.12E-07	1.10E-04	4.92E-04	-3.00E-04	-1.10E-03	3.65E-04
-4.80E-07	-6.05E-04	2.77E-04	-2.67E-04	-4.51E-04	-6.65E-05
-4.48E-07	-2.43E-04	5.20E-04	-5.30E-04	-1.61E-03	2.53E-04
-4.16E-07	-5.58E-04	6.99E-04	-2.78E-04	-5.53E-04	2.97E-04
-3.84E-07	2.06E-04	5.48E-04	-3.67E-04	-6.77E-04	7.67E-05
-3.52E-07	-3.16E-04	4.27E-04	-5.82E-05	-1.19E-03	6.91E-04
-3.20E-07	-1.62E-04	6.40E-04	-2.72E-04	-9.74E-04	7.98E-04
-2.88E-07	-6.91E-05	1.28E-04	-1.39E-04	-1.21E-03	7.49E-04
-2.56E-07	-1.41E-04	7.24E-04	-1.22E-04	-1.22E-03	-1.37E-05
-2.24E-07	-2.93E-04	4.37E-04	-3.28E-04	-1.17E-03	7.38E-04
-1.92E-07	-5.02E-05	4.20E-04	7.17E-04	-9.73E-04	6.94E-04
-1.60E-07	-3.74E-05	5.79E-04	-4.18E-04	-6.92E-04	5.98E-04
-1.28E-07	-2.09E-04	9.83E-04	-2.74E-04	-1.06E-03	2.76E-05
-9.62E-08	-1.37E-04	5.04E-04	9.24E-06	-5.89E-04	-1.54E-04
-6.42E-08	-4.07E-04	6.17E-04	-2.12E-04	-9.65E-04	6.49E-04
-3.22E-08	1.50E-04	3.74E-04	3.72E-04	-8.47E-04	7.19E-04
-1.78E-10	-3.22E-03	-2.13E-03	-2.75E-03	-3.43E-03	-2.54E-03
3.18E-08	-1.05E-02	-9.85E-03	-1.16E-02	-1.08E-02	-1.37E-02
6.38E-08	1.49E-02	1.53E-02	1.37E-02	1.26E-02	1.32E-02
9.58E-08	1.54E-02	1.61E-02	1.55E-02	1.45E-02	1.50E-02
1.28E-07	1.56E-02	1.52E-02	1.47E-02	1.41E-02	1.35E-02
1.60E-07	1.42E-02	1.48E-02	1.40E-02	1.25E-02	1.33E-02
1.92E-07	1.39E-02	1.42E-02	1.32E-02	1.13E-02	1.24E-02
2.24E-07	1.34E-02	1.37E-02	1.33E-02	1.12E-02	1.17E-02
2.56E-07	1.31E-02	1.38E-02	1.18E-02	1.04E-02	1.07E-02
2.88E-07	1.26E-02	1.26E-02	1.13E-02	9.54E-03	9.78E-03
3.20E-07	1.23E-02	1.26E-02	1.12E-02	9.37E-03	9.87E-03
3.52E-07	1.15E-02	1.19E-02	1.09E-02	8.07E-03	9.22E-03
3.84E-07	1.17E-02	1.10E-02	9.88E-03	7.96E-03	8.13E-03
4.16E-07	1.10E-02	1.06E-02	9.48E-03	7.13E-03	7.59E-03
4.48E-07	1.07E-02	1.08E-02	9.12E-03	6.98E-03	7.83E-03
4.80E-07	1.05E-02	1.02E-02	8.89E-03	6.66E-03	7.42E-03
5.12E-07	9.99E-03	9.22E-03	7.99E-03	5.69E-03	6.62E-03
5.44E-07	9.84E-03	9.25E-03	7.72E-03	5.92E-03	6.01E-03

5.76E-07	8.99E-03	9.29E-03	7.64E-03	5.43E-03	5.73E-03
6.08E-07	8.91E-03	8.91E-03	6.71E-03	4.91E-03	5.49E-03
6.40E-07	8.48E-03	8.46E-03	7.01E-03	4.40E-03	5.27E-03
6.72E-07	8.15E-03	7.83E-03	6.09E-03	4.63E-03	4.46E-03
7.04E-07	7.97E-03	7.51E-03	5.66E-03	3.46E-03	4.50E-03
7.36E-07	8.17E-03	7.07E-03	6.13E-03	3.28E-03	4.42E-03
7.68E-07	7.60E-03	6.64E-03	5.52E-03	3.13E-03	4.47E-03
8.00E-07	7.32E-03	6.73E-03	4.99E-03	3.60E-03	3.93E-03
8.32E-07	6.75E-03	6.23E-03	5.00E-03	2.92E-03	3.92E-03
8.64E-07	7.00E-03	5.93E-03	4.90E-03	3.21E-03	3.72E-03
8.96E-07	7.12E-03	5.87E-03	3.69E-03	2.79E-03	3.45E-03
9.28E-07	6.46E-03	4.93E-03	4.16E-03	2.44E-03	3.08E-03
9.60E-07	5.77E-03	5.77E-03	4.06E-03	2.32E-03	3.44E-03
9.92E-07	5.49E-03	5.34E-03	4.99E-03	2.01E-03	2.56E-03
1.02E-06	5.68E-03	4.87E-03	3.56E-03	1.28E-03	2.21E-03
1.06E-06	5.82E-03	4.73E-03	3.59E-03	1.67E-03	2.10E-03
1.09E-06	5.27E-03	4.84E-03	3.14E-03	1.64E-03	2.28E-03
1.12E-06	5.24E-03	4.96E-03	3.01E-03	1.63E-03	1.95E-03
1.15E-06	5.35E-03	4.71E-03	3.19E-03	1.51E-03	2.16E-03
1.18E-06	5.52E-03	4.60E-03	3.41E-03	1.37E-03	2.30E-03
1.22E-06	4.63E-03	4.70E-03	2.92E-03	1.16E-03	1.83E-03
1.25E-06	4.66E-03	4.21E-03	3.02E-03	1.45E-03	2.01E-03
1.28E-06	4.96E-03	4.02E-03	2.76E-03	1.21E-03	1.78E-03
1.31E-06	4.56E-03	4.28E-03	2.88E-03	8.27E-04	1.79E-03
1.34E-06	4.56E-03	3.89E-03	2.68E-03	1.31E-03	1.85E-03
1.38E-06	4.36E-03	3.87E-03	2.96E-03	7.59E-04	1.78E-03
1.41E-06	3.89E-03	3.47E-03	3.36E-03	5.79E-04	1.38E-03
1.44E-06	3.84E-03	3.23E-03	2.74E-03	1.06E-03	1.61E-03
1.47E-06	4.44E-03	2.67E-03	2.21E-03	8.13E-04	1.82E-03
1.50E-06	3.73E-03	3.20E-03	2.63E-03	8.66E-04	1.78E-03
1.54E-06	3.93E-03	2.45E-03	2.75E-03	5.52E-04	1.77E-03
1.57E-06	3.25E-03	2.96E-03	2.46E-03	6.29E-04	1.67E-03
1.60E-06	3.52E-03	3.19E-03	2.44E-03	1.06E-03	1.61E-03
1.63E-06	3.32E-03	2.92E-03	2.63E-03	1.93E-04	1.67E-03
1.66E-06	3.21E-03	2.80E-03	2.47E-03	5.64E-04	1.45E-03
1.70E-06	3.17E-03	2.72E-03	2.06E-03	2.36E-04	1.82E-03
1.73E-06	3.16E-03	2.74E-03	2.22E-03	2.81E-04	1.28E-03
1.76E-06	3.17E-03	2.84E-03	2.04E-03	6.36E-04	1.81E-03
1.79E-06	3.22E-03	2.75E-03	2.38E-03	-2.16E-04	1.57E-03
1.82E-06	3.18E-03	2.70E-03	2.02E-03	6.62E-05	1.51E-03
1.86E-06	3.26E-03	2.75E-03	1.71E-03	4.65E-04	1.18E-03
1.89E-06	3.20E-03	2.45E-03	1.54E-03	-1.91E-05	1.25E-03
1.92E-06	3.28E-03	2.44E-03	1.55E-03	5.80E-05	1.81E-03
1.95E-06	2.20E-03	2.70E-03	1.76E-03	-7.52E-05	1.42E-03
1.98E-06	2.69E-03	2.27E-03	2.05E-03	-5.09E-05	1.24E-03
2.02E-06	2.34E-03	2.41E-03	2.00E-03	4.78E-04	1.12E-03
2.05E-06	2.91E-03	2.40E-03	1.79E-03	2.42E-04	1.92E-03
2.08E-06	2.14E-03	2.14E-03	1.32E-03	4.26E-04	1.53E-03
2.11E-06	2.33E-03	2.17E-03	1.50E-03	2.51E-05	1.26E-03
2.14E-06	2.17E-03	2.61E-03	1.57E-03	4.07E-04	1.66E-03
2.18E-06	2.01E-03	2.21E-03	1.36E-03	-2.26E-04	9.29E-04
2.21E-06	2.29E-03	1.82E-03	1.60E-03	1.37E-05	1.61E-03
2.24E-06	2.15E-03	1.94E-03	1.19E-03	4.91E-04	1.25E-03
2.27E-06	2.22E-03	1.77E-03	1.46E-03	-3.59E-04	1.62E-03

2.30E-06	1.91E-03	2.06E-03	1.31E-03	1.71E-04	1.53E-03
2.34E-06	2.08E-03	2.26E-03	8.72E-04	1.03E-04	1.81E-03
2.37E-06	1.77E-03	1.73E-03	1.56E-03	5.07E-04	1.50E-03
2.40E-06	1.91E-03	2.37E-03	1.18E-03	3.03E-04	1.40E-03
2.43E-06	1.56E-03	2.00E-03	1.64E-03	2.81E-04	1.20E-03
2.46E-06	1.68E-03	1.40E-03	1.62E-03	3.71E-05	7.29E-04
2.50E-06	1.60E-03	2.40E-03	1.63E-03	-1.90E-04	1.62E-03
2.53E-06	1.56E-03	2.45E-03	1.32E-03	-1.75E-04	1.23E-03
2.56E-06	1.96E-03	1.46E-03	6.56E-04	-3.84E-04	1.28E-03
2.59E-06	1.34E-03	2.43E-03	1.24E-03	-4.05E-04	1.15E-03
2.62E-06	1.38E-03	2.17E-03	1.24E-03	6.35E-04	5.15E-04
2.66E-06	1.47E-03	2.08E-03	1.47E-03	-6.06E-04	1.40E-03
2.69E-06	1.56E-03	2.22E-03	1.25E-03	4.52E-04	1.30E-03
2.72E-06	1.56E-03	2.49E-03	3.79E-04	3.59E-04	1.43E-03
2.75E-06	1.43E-03	1.84E-03	1.65E-03	-2.63E-04	1.53E-03
2.78E-06	1.17E-03	2.19E-03	1.40E-03	-1.09E-03	1.23E-03
2.82E-06	1.18E-03	2.14E-03	1.04E-03	-1.81E-04	1.05E-03
2.85E-06	1.61E-03	2.05E-03	7.69E-04	-1.22E-04	1.22E-03
2.88E-06	9.42E-04	2.10E-03	9.02E-04	6.40E-05	7.91E-04
2.91E-06	1.41E-03	2.15E-03	2.65E-04	-2.60E-04	1.30E-03
2.94E-06	1.24E-03	1.51E-03	7.74E-04	-1.62E-04	1.58E-03
2.98E-06	1.55E-03	1.18E-03	4.44E-04	1.99E-04	1.56E-03
3.01E-06	1.27E-03	1.28E-03	8.31E-04	-1.31E-04	9.58E-04
3.04E-06	1.16E-03	1.13E-03	6.90E-04	2.12E-04	1.04E-03
3.07E-06	1.09E-03	2.37E-03	4.62E-04	-2.93E-04	1.38E-03
3.10E-06	1.14E-03	1.20E-03	2.46E-04	-4.52E-04	8.93E-04
3.14E-06	1.43E-03	1.48E-03	7.48E-04	9.07E-05	1.31E-03
3.17E-06	5.87E-04	1.49E-03	9.46E-04	1.37E-04	1.61E-03
3.20E-06	7.54E-04	1.68E-03	5.31E-04	1.69E-04	1.04E-03
3.23E-06	1.40E-03	1.46E-03	1.32E-03	3.60E-04	6.85E-04
3.26E-06	1.23E-03	7.52E-04	9.24E-04	2.26E-04	1.11E-03
3.30E-06	9.63E-04	1.14E-03	2.80E-04	-9.64E-06	1.12E-03
3.33E-06	1.00E-03	5.48E-04	4.17E-04	-7.15E-04	7.69E-04
3.36E-06	6.62E-04	8.97E-04	4.15E-04	1.07E-05	9.36E-04
3.39E-06	8.67E-04	9.11E-04	7.04E-04	1.85E-04	9.32E-04
3.42E-06	4.22E-04	1.38E-03	3.46E-04	2.49E-05	8.42E-04
3.46E-06	7.62E-04	1.44E-03	9.32E-04	-2.60E-04	1.22E-03
3.49E-06	1.22E-03	1.95E-03	4.36E-04	-1.32E-04	2.47E-04
3.52E-06	9.07E-04	2.13E-03	2.76E-04	-2.57E-04	1.02E-03
3.55E-06	8.55E-04	1.49E-03	1.17E-03	-2.89E-04	1.38E-03
3.58E-06	7.21E-04	2.29E-03	5.67E-04	4.83E-05	9.69E-04
3.62E-06	6.91E-04	1.77E-03	5.05E-04	-2.69E-04	9.95E-04
3.65E-06	9.98E-04	1.22E-03	4.62E-04	-3.51E-04	5.83E-04
3.68E-06	9.49E-04	1.62E-03	1.14E-03	-6.84E-04	1.05E-03
3.71E-06	6.57E-04	1.54E-03	1.86E-04	-1.66E-04	8.33E-04
3.74E-06	1.21E-03	1.54E-03	5.79E-04	-4.87E-04	1.05E-03
3.78E-06	1.11E-03	1.89E-03	7.87E-04	-8.31E-05	1.14E-03
3.81E-06	6.35E-04	1.51E-03	5.64E-04	-4.90E-04	1.62E-03
3.84E-06	1.48E-03	1.74E-03	2.36E-04	-3.40E-04	6.98E-04
3.87E-06	8.85E-04	1.18E-03	7.43E-04	-1.57E-04	8.29E-04
3.90E-06	8.20E-04	1.79E-03	2.09E-04	-3.12E-04	6.96E-04
3.94E-06	1.20E-03	2.09E-03	5.25E-04	2.68E-04	5.80E-04
3.97E-06	1.35E-03	1.62E-03	9.20E-04	2.04E-04	1.28E-03
4.00E-06	8.36E-04	8.50E-04	8.86E-04	-1.42E-04	7.71E-04

4.03E-06	9.03E-04	2.15E-03	7.80E-04	-7.77E-05	9.23E-04
4.06E-06	8.65E-04	1.33E-03	1.43E-03	-3.40E-05	-2.33E-04
4.10E-06	7.83E-04	1.83E-03	2.07E-04	-1.67E-04	1.28E-03
4.13E-06	1.30E-03	1.52E-03	6.80E-04	-4.42E-04	1.13E-03
4.16E-06	7.83E-04	1.68E-03	1.30E-03	-4.68E-04	8.37E-04
4.19E-06	8.50E-04	1.34E-03	1.17E-03	-1.40E-04	5.73E-04
4.22E-06	8.82E-04	1.77E-03	4.04E-04	-2.96E-04	1.28E-03
4.26E-06	9.48E-04	2.07E-03	8.52E-04	-7.39E-04	1.15E-03
4.29E-06	8.90E-04	1.51E-03	1.30E-03	-4.18E-04	1.24E-03
4.32E-06	6.14E-04	1.15E-03	1.11E-03	1.73E-04	1.06E-03
4.35E-06	9.37E-04	1.22E-03	9.24E-04	-6.11E-04	1.13E-03
4.38E-06	5.21E-04	1.48E-03	9.14E-04	-5.00E-04	1.23E-03
4.42E-06	7.73E-04	2.02E-03	4.94E-04	1.78E-04	1.03E-03
4.45E-06	1.01E-03	1.79E-03	1.30E-03	8.03E-05	8.53E-04
4.48E-06	8.25E-04	1.08E-03	5.70E-04	-7.38E-04	1.55E-03
4.51E-06	-1.29E-04	1.28E-03	8.50E-04	4.76E-04	1.20E-03
4.54E-06	8.56E-04	1.93E-03	1.02E-03	-4.36E-04	4.24E-04
4.58E-06	7.57E-04	1.30E-03	1.01E-03	-4.38E-04	7.25E-04
4.61E-06	3.02E-04	9.69E-04	1.24E-03	-3.60E-05	1.34E-03
4.64E-06	9.28E-04	9.44E-04	8.43E-04	5.26E-04	9.71E-04
4.67E-06	4.81E-06	1.59E-03	1.08E-03	-1.33E-04	5.28E-04
4.70E-06	3.13E-04	1.50E-03	7.71E-04	-5.16E-04	1.23E-03
4.74E-06	-2.92E-04	1.01E-03	1.11E-03	-4.81E-04	1.11E-03
4.77E-06	4.82E-04	1.62E-03	2.95E-04	-2.18E-04	1.48E-03
4.80E-06	5.62E-04	1.16E-03	8.19E-04	1.44E-04	1.19E-03
4.83E-06	6.26E-04	1.80E-03	6.13E-04	5.53E-05	9.62E-04
4.86E-06	2.76E-04	6.13E-04	7.53E-04	-1.11E-04	9.24E-04
4.90E-06	8.35E-04	1.02E-03	5.50E-04	-5.27E-04	1.43E-03
4.93E-06	2.83E-04	6.44E-04	8.91E-04	-1.01E-03	8.94E-04
4.96E-06	-1.06E-04	6.90E-04	1.24E-03	-1.42E-04	1.03E-03
4.99E-06	-1.13E-05	1.10E-03	6.19E-04	-5.22E-04	1.12E-03
5.02E-06	4.67E-04	1.07E-03	5.92E-04	-7.96E-04	1.05E-03
5.06E-06	4.35E-04	1.76E-03	5.74E-04	-7.19E-04	1.17E-03
5.09E-06	4.59E-04	8.24E-04	3.71E-04	-3.53E-04	1.24E-03
5.12E-06	6.14E-04	1.74E-03	7.17E-04	-1.87E-04	1.30E-03
5.15E-06	4.14E-04	1.39E-03	5.09E-04	1.06E-05	8.89E-04
5.18E-06	-7.71E-05	1.66E-03	1.07E-03	-1.67E-04	5.87E-04
5.22E-06	3.82E-04	1.28E-03	9.68E-04	-4.09E-04	1.33E-03
5.25E-06	3.47E-04	8.73E-04	2.53E-04	-3.56E-04	9.47E-04
5.28E-06	1.04E-04	9.33E-04	1.14E-03	-5.87E-05	1.34E-03
5.31E-06	9.06E-04	1.66E-03	4.29E-04	-6.12E-04	1.33E-03
5.34E-06	6.07E-04	5.16E-04	8.61E-04	-2.96E-05	8.24E-04
5.38E-06	5.37E-04	1.40E-03	3.39E-04	-6.59E-04	1.01E-03
5.41E-06	2.21E-04	1.55E-03	-1.01E-04	-2.76E-04	9.23E-04
5.44E-06	3.44E-04	1.57E-03	3.04E-04	-1.47E-04	3.42E-04
5.47E-06	7.80E-04	1.47E-03	1.16E-03	-5.70E-04	9.37E-04
5.50E-06	3.29E-04	1.41E-03	8.19E-05	-9.70E-04	5.40E-04
5.54E-06	2.00E-04	2.33E-03	5.82E-04	-7.28E-04	7.71E-04
5.57E-06	7.96E-04	2.14E-03	6.14E-04	-2.69E-04	4.40E-04
5.60E-06	1.08E-03	1.47E-03	9.65E-04	-3.99E-04	1.37E-03
5.63E-06	1.03E-03	2.19E-03	3.80E-04	-1.98E-04	7.64E-04
5.66E-06	5.82E-04	1.55E-03	6.25E-04	6.69E-05	1.03E-03
5.70E-06	3.31E-04	1.24E-03	6.70E-04	-1.22E-04	7.74E-04
5.73E-06	6.78E-04	1.63E-03	4.26E-05	-5.62E-04	1.06E-03

5.76E-06	4.39E-04	1.62E-03	7.42E-04	-1.90E-04	4.27E-04
5.79E-06	2.28E-04	2.26E-03	6.97E-04	5.93E-04	1.08E-03
5.82E-06	1.79E-04	1.79E-03	8.79E-04	-1.31E-05	1.27E-03
5.86E-06	1.07E-05	1.17E-03	7.35E-04	5.44E-04	8.96E-04
5.89E-06	-1.26E-04	2.23E-03	7.33E-04	-6.86E-04	1.02E-03
5.92E-06	5.75E-04	1.79E-03	7.81E-04	-5.82E-04	1.04E-03
5.95E-06	7.41E-04	1.92E-03	1.03E-03	-6.17E-04	1.45E-03
5.98E-06	1.95E-04	1.11E-03	4.26E-04	8.79E-05	1.06E-03
6.02E-06	2.88E-04	2.03E-03	-1.19E-04	1.44E-04	7.20E-04
6.05E-06	5.19E-04	1.56E-03	6.12E-04	-9.17E-04	1.00E-03
6.08E-06	6.73E-04	1.50E-03	6.56E-04	-4.23E-04	8.07E-04
6.11E-06	5.17E-04	1.38E-03	6.49E-04	3.02E-04	1.18E-03
6.14E-06	6.49E-04	1.98E-03	7.59E-04	2.49E-04	9.90E-04
6.18E-06	7.96E-04	1.88E-03	1.28E-03	-1.16E-03	7.76E-04
6.21E-06	5.25E-04	2.42E-03	1.00E-03	-5.66E-04	9.78E-04
6.24E-06	2.58E-04	9.42E-04	5.75E-04	-1.43E-04	1.20E-03
6.27E-06	2.48E-04	7.75E-04	1.01E-03	-6.46E-05	9.38E-04
6.30E-06	7.94E-04	1.53E-03	6.02E-04	1.84E-04	9.57E-04
6.34E-06	2.17E-04	1.26E-03	6.17E-04	-3.64E-04	5.78E-04
6.37E-06	5.18E-04	1.68E-03	-2.39E-04	-5.38E-04	7.46E-04
6.40E-06	6.16E-04	1.89E-03	-2.30E-04	-4.42E-04	8.72E-04
6.43E-06	9.03E-04	1.42E-03	2.06E-04	-8.20E-05	1.25E-03
6.46E-06	3.78E-04	1.92E-03	8.03E-04	-2.89E-04	1.25E-03
6.50E-06	5.81E-04	1.12E-03	4.82E-04	-4.91E-05	1.31E-03
6.53E-06	4.78E-04	2.04E-03	3.69E-04	2.83E-05	8.17E-04
6.56E-06	2.02E-04	1.21E-03	9.10E-04	-1.41E-04	6.29E-04
6.59E-06	3.13E-04	1.20E-03	2.67E-04	2.27E-04	1.15E-03
6.62E-06	5.94E-04	1.67E-03	4.34E-04	2.07E-05	8.60E-04
6.66E-06	6.67E-04	1.60E-03	1.03E-03	-4.23E-04	1.43E-03
6.69E-06	6.99E-04	2.11E-03	2.89E-04	2.75E-04	5.01E-04
6.72E-06	-9.04E-06	1.34E-03	3.32E-04	-8.24E-04	8.87E-04
6.75E-06	1.79E-04	1.12E-03	7.18E-04	-2.73E-04	8.35E-04
6.78E-06	3.84E-04	2.08E-03	5.10E-04	-2.03E-04	9.35E-04
6.82E-06	7.50E-04	1.64E-03	2.42E-04	-4.66E-04	1.13E-03
6.85E-06	6.23E-04	9.58E-04	1.12E-03	-6.25E-07	9.62E-04
6.88E-06	9.48E-04	1.14E-03	5.56E-04	-3.74E-04	8.30E-04
6.91E-06	6.99E-04	1.67E-03	5.16E-04	-4.89E-06	6.98E-04
6.94E-06	-1.73E-04	1.62E-03	2.09E-04	-7.02E-04	1.01E-03
6.98E-06	5.47E-04	2.24E-03	1.09E-03	-1.17E-04	1.07E-03
7.01E-06	5.19E-04	1.62E-03	7.50E-04	-1.59E-04	1.24E-03
7.04E-06	4.75E-04	1.56E-03	2.72E-04	1.96E-04	8.57E-04
7.07E-06	-1.63E-04	1.85E-03	4.58E-04	6.05E-05	8.25E-04
7.10E-06	6.65E-04	1.93E-03	3.51E-04	-1.08E-04	1.07E-03
7.14E-06	-9.70E-06	2.03E-03	5.76E-04	-1.87E-04	8.83E-04
7.17E-06	1.67E-04	2.22E-03	6.08E-04	6.01E-05	1.04E-03
7.20E-06	3.98E-04	1.76E-03	7.29E-05	-1.34E-04	6.54E-04
7.23E-06	-6.12E-05	1.03E-03	1.18E-04	-7.74E-04	1.09E-03
7.26E-06	4.20E-04	7.55E-04	9.09E-04	-1.25E-05	9.53E-04
7.30E-06	1.81E-04	1.42E-03	8.02E-04	-2.73E-04	4.47E-04
7.33E-06	8.16E-04	1.83E-03	5.48E-04	-3.43E-04	1.07E-03
7.36E-06	4.78E-04	1.11E-03	1.13E-03	-1.13E-04	1.08E-03
7.39E-06	5.27E-04	9.94E-04	4.56E-05	1.70E-04	7.31E-04
7.42E-06	1.73E-04	8.61E-04	6.18E-04	1.00E-04	7.76E-04
7.46E-06	7.67E-05	1.29E-03	1.09E-03	-5.75E-04	9.11E-04

7.49E-06	5.59E-04	1.34E-03	3.64E-04	-3.59E-04	2.65E-04
7.52E-06	8.64E-04	1.68E-03	6.20E-04	-5.55E-04	1.14E-03
7.55E-06	8.23E-04	1.33E-03	3.44E-04	-1.22E-04	7.19E-04
7.58E-06	4.84E-04	1.49E-03	1.49E-03	-5.12E-04	7.69E-04
7.62E-06	3.72E-04	9.74E-04	5.01E-04	-2.12E-04	9.94E-04
7.65E-06	5.35E-04	1.26E-03	8.21E-04	-2.52E-04	7.43E-04
7.68E-06	7.73E-04	1.53E-03	3.78E-04	-9.51E-05	9.48E-04
7.71E-06	3.48E-05	1.92E-03	-1.08E-04	-7.44E-05	6.21E-04
7.74E-06	4.18E-04	1.02E-03	6.15E-04	-1.22E-05	7.41E-04
7.78E-06	4.27E-04	1.33E-03	7.53E-04	2.74E-04	7.35E-04
7.81E-06	7.05E-04	9.14E-04	1.23E-03	-2.17E-04	1.13E-03
7.84E-06	6.08E-05	1.37E-03	9.10E-04	-5.51E-04	1.38E-03
7.87E-06	2.43E-04	1.80E-03	1.06E-03	-5.13E-04	8.58E-04
7.90E-06	6.52E-05	1.73E-03	5.45E-04	-2.08E-05	8.42E-04
7.94E-06	4.47E-04	1.18E-03	1.10E-03	-3.90E-04	2.30E-04
7.97E-06	7.63E-04	1.62E-03	5.18E-04	2.87E-05	2.88E-04
8.00E-06	4.92E-04	1.50E-03	5.57E-04	-1.88E-04	1.32E-03
8.03E-06	3.05E-04	1.04E-03	6.09E-04	-3.28E-04	7.20E-04
8.06E-06	1.33E-03	6.52E-04	6.92E-04	-4.00E-04	1.24E-04
8.10E-06	1.97E-04	1.02E-03	6.71E-04	-4.81E-04	4.80E-04
8.13E-06	2.80E-04	1.49E-03	7.31E-04	-3.22E-04	3.40E-04
8.16E-06	6.76E-04	1.45E-03	3.42E-04	-8.88E-06	7.17E-04
8.19E-06	7.00E-04	1.41E-03	6.86E-04	-4.33E-04	1.08E-03
8.22E-06	4.59E-04	1.38E-03	8.43E-04	-5.46E-04	1.04E-03
8.26E-06	5.37E-04	9.58E-04	4.83E-04	-2.21E-04	1.45E-03
8.29E-06	8.20E-04	1.45E-03	6.09E-04	-3.70E-04	1.04E-03
8.32E-06	4.49E-04	7.09E-04	6.55E-04	-4.84E-04	2.54E-04
8.35E-06	2.89E-04	1.21E-03	5.46E-04	-5.18E-04	8.77E-04
8.38E-06	2.49E-04	1.16E-03	3.17E-04	-3.46E-04	5.00E-04
8.42E-06	6.57E-04	1.53E-03	5.86E-04	-9.01E-04	8.19E-04
8.45E-06	8.58E-04	6.72E-04	4.52E-04	-3.59E-04	7.73E-04
8.48E-06	3.65E-04	1.02E-03	7.38E-04	-4.31E-04	8.57E-04
8.51E-06	5.09E-04	8.22E-04	5.83E-04	-4.44E-04	3.30E-04
8.54E-06	6.55E-04	1.09E-03	7.05E-04	-1.68E-04	1.28E-03
8.58E-06	9.06E-04	1.55E-03	9.12E-04	-6.53E-04	5.46E-04
8.61E-06	5.75E-04	1.07E-03	4.99E-04	-2.10E-04	1.87E-03
8.64E-06	6.82E-04	1.19E-03	8.40E-04	-6.42E-04	8.42E-04
8.67E-06	7.71E-04	1.47E-03	1.06E-03	-5.89E-04	3.00E-04
8.70E-06	6.44E-04	1.34E-03	1.08E-03	2.03E-04	-4.02E-04
8.74E-06	8.22E-04	1.36E-03	7.52E-04	-3.91E-04	6.98E-04
8.77E-06	8.64E-04	1.30E-03	7.94E-04	-2.55E-04	5.44E-04
8.80E-06	4.51E-04	1.09E-03	7.67E-04	-3.72E-04	3.52E-04
8.83E-06	9.05E-04	1.70E-03	9.82E-04	-1.10E-04	7.22E-04
8.86E-06	6.13E-04	1.52E-03	1.08E-03	-3.59E-04	1.24E-03
8.90E-06	8.19E-04	1.49E-03	1.15E-03	-5.82E-04	1.01E-03
8.93E-06	3.93E-04	1.22E-03	6.06E-04	-4.14E-05	1.12E-03
8.96E-06	1.41E-04	9.83E-04	1.05E-03	-6.36E-04	3.04E-04
8.99E-06	6.79E-04	1.41E-03	4.96E-04	-2.20E-04	2.39E-04
9.02E-06	7.54E-04	1.83E-03	2.14E-04	5.10E-05	5.82E-04
9.06E-06	5.68E-04	1.62E-03	9.09E-04	-1.93E-04	1.15E-05
9.09E-06	6.59E-04	1.05E-03	8.81E-04	-3.49E-04	5.10E-04
9.12E-06	1.03E-03	9.22E-04	8.46E-04	-2.41E-04	5.69E-04
9.15E-06	7.78E-04	1.44E-03	4.44E-04	-3.48E-04	1.28E-03
9.18E-06	6.95E-04	1.19E-03	3.01E-04	-2.90E-04	9.69E-04

9.22E-06	9.22E-04	1.68E-03	7.20E-04	-3.47E-04	5.90E-04
9.25E-06	1.04E-04	1.22E-03	5.61E-04	-6.22E-04	5.36E-04
9.28E-06	7.57E-04	1.67E-03	5.78E-04	-5.85E-04	9.80E-04
9.31E-06	7.74E-04	1.54E-03	1.08E-03	-3.30E-04	7.09E-04
9.34E-06	9.93E-04	1.47E-03	1.01E-03	-5.65E-04	4.71E-04
9.38E-06	4.53E-04	1.17E-03	5.98E-04	-2.97E-04	1.89E-04
9.41E-06	7.57E-04	9.39E-04	7.81E-04	-2.53E-04	7.30E-04
9.44E-06	8.41E-04	8.79E-04	6.81E-04	-8.10E-04	8.29E-04
9.47E-06	1.09E-03	8.00E-04	9.92E-04	-7.48E-04	1.41E-03
9.50E-06	5.71E-04	1.08E-03	1.01E-03	-4.84E-04	5.11E-04
9.54E-06	8.86E-04	1.15E-03	2.73E-04	-4.95E-04	6.44E-04
9.57E-06	2.30E-04	1.20E-03	8.30E-04	-1.49E-04	-8.21E-05
9.60E-06	4.58E-04	8.72E-04	9.63E-04	-2.62E-04	7.03E-04
9.63E-06	4.68E-04	9.51E-04	1.14E-03	-2.93E-04	6.54E-04
9.66E-06	6.40E-04	8.02E-04	4.07E-05	-4.78E-04	1.26E-03
9.70E-06	5.58E-04	9.28E-04	-1.23E-04	-7.13E-04	6.01E-04
9.73E-06	7.57E-04	9.40E-04	8.06E-04	-4.26E-04	5.84E-04
9.76E-06	7.86E-04	1.25E-03	3.11E-04	-7.63E-04	9.66E-04
9.79E-06	3.81E-04	8.83E-04	1.02E-03	-6.19E-04	8.80E-04
9.82E-06	-1.23E-04	8.12E-04	6.21E-04	-5.38E-04	8.63E-04
9.86E-06	8.41E-04	1.02E-03	8.54E-04	-5.45E-04	9.62E-04
9.89E-06	7.21E-04	1.23E-03	9.18E-04	-4.65E-04	6.33E-04
9.92E-06	2.30E-04	1.11E-03	7.80E-04	-6.97E-04	2.22E-04
9.95E-06	6.81E-04	8.80E-04	7.37E-04	-5.66E-04	1.32E-03
9.98E-06	3.26E-04	1.20E-03	1.07E-03	1.99E-05	1.45E-04
1.00E-05	2.82E-04	1.11E-03	7.99E-04	-6.50E-04	3.43E-04
1.01E-05	7.38E-04	1.18E-03	4.89E-04	-4.57E-04	4.83E-04
1.01E-05	9.96E-04	1.55E-03	6.66E-04	-8.64E-05	2.17E-04
1.01E-05	7.09E-04	1.42E-03	8.23E-04	-5.10E-04	-2.51E-05
1.01E-05	6.54E-04	1.22E-03	9.07E-04	-7.70E-05	5.59E-04
1.02E-05	5.06E-04	8.05E-04	5.07E-04	-3.28E-04	3.34E-04
1.02E-05	5.89E-04	8.84E-04	1.02E-03	-4.34E-04	5.68E-04
1.02E-05	6.84E-04	1.00E-03	7.86E-04	-3.18E-05	2.47E-04
1.03E-05	5.84E-04	1.48E-03	4.68E-04	-5.33E-04	4.76E-04
1.03E-05	6.70E-04	9.94E-04	6.41E-04	-6.45E-04	-2.75E-05
1.03E-05	1.01E-03	9.20E-04	8.81E-04	-5.78E-04	5.59E-04
1.04E-05	7.78E-04	1.20E-03	8.71E-04	-5.66E-04	4.62E-04
1.04E-05	5.26E-04	8.98E-04	5.80E-04	-7.07E-04	2.33E-04
1.04E-05	2.02E-04	1.04E-03	2.70E-04	-7.06E-04	5.71E-04
1.05E-05	3.29E-04	9.54E-04	7.44E-04	-5.67E-04	8.26E-05
1.05E-05	5.90E-04	1.19E-03	1.13E-03	-5.33E-04	1.25E-04
1.05E-05	6.52E-04	9.53E-04	1.96E-04	-5.70E-04	1.11E-03
1.06E-05	8.69E-04	9.37E-04	4.14E-04	-1.36E-04	9.41E-05
1.06E-05	9.57E-04	1.26E-03	8.11E-04	-5.21E-04	1.40E-03
1.06E-05	8.66E-04	1.25E-03	7.48E-04	-4.35E-04	3.23E-04
1.07E-05	5.49E-04	9.95E-04	1.00E-03	-5.88E-04	1.59E-04
1.07E-05	7.80E-04	9.35E-04	9.59E-04	-4.19E-04	4.95E-04
1.07E-05	2.09E-04	1.53E-03	5.05E-04	-2.34E-04	9.16E-04
1.08E-05	4.56E-04	1.00E-03	6.30E-04	-1.20E-04	9.15E-04
1.08E-05	6.13E-04	9.57E-04	5.79E-04	-2.73E-04	1.23E-03
1.08E-05	4.89E-04	1.23E-03	1.08E-03	-4.29E-04	6.74E-04
1.09E-05	4.81E-04	7.85E-04	9.31E-04	-5.25E-04	8.51E-04
1.09E-05	9.73E-05	1.10E-03	6.19E-04	-1.78E-04	5.82E-04
1.09E-05	9.11E-04	1.07E-03	4.63E-04	-6.29E-04	5.40E-04

1.09E-05	2.99E-04	1.03E-03	8.90E-04	-4.99E-04	4.61E-04
1.10E-05	5.26E-04	9.79E-04	1.03E-03	-3.29E-04	4.46E-04
1.10E-05	4.85E-04	1.15E-03	5.12E-04	-4.56E-04	6.07E-04
1.10E-05	5.11E-04	8.94E-04	6.82E-04	-2.48E-04	4.52E-04
1.11E-05	4.37E-04	7.46E-04	5.84E-04	-4.99E-04	4.75E-04
1.11E-05	1.06E-03	8.99E-04	1.12E-03	-7.29E-04	8.41E-04
1.11E-05	8.84E-04	1.15E-03	6.31E-04	-4.30E-04	1.02E-03
1.12E-05	4.81E-04	1.00E-03	8.26E-04	-3.14E-04	-1.68E-04
1.12E-05	4.95E-04	1.14E-03	3.14E-04	-3.10E-04	3.40E-04
1.12E-05	5.44E-04	1.58E-03	8.93E-04	-4.16E-04	1.87E-04
1.13E-05	6.73E-04	1.02E-03	1.32E-03	-4.99E-04	4.64E-04
1.13E-05	6.85E-04	1.08E-03	9.14E-04	1.97E-05	6.02E-04
1.13E-05	6.58E-04	1.06E-03	-2.50E-04	1.25E-05	4.85E-04
1.14E-05	3.51E-04	8.67E-04	4.40E-04	-7.04E-04	1.93E-04
1.14E-05	6.17E-04	1.09E-03	7.33E-04	-6.16E-04	-2.49E-04
1.14E-05	5.28E-04	1.00E-03	7.98E-04	-3.98E-06	6.43E-04
1.15E-05	6.32E-04	1.11E-03	1.05E-03	-2.11E-04	1.66E-05
1.15E-05	9.44E-04	8.23E-04	8.99E-04	-2.16E-04	1.34E-04
1.15E-05	7.64E-04	9.21E-04	6.63E-04	-7.42E-04	4.36E-04
1.16E-05	4.95E-04	1.00E-03	3.55E-04	-1.30E-05	5.89E-04
1.16E-05	1.68E-04	1.16E-03	4.34E-04	-3.16E-04	5.57E-04
1.16E-05	2.85E-04	8.31E-04	5.47E-04	-4.62E-04	2.52E-04
1.17E-05	6.73E-04	1.14E-03	4.61E-04	-7.00E-04	3.32E-04
1.17E-05	8.27E-04	1.72E-03	6.47E-04	1.96E-04	2.05E-04
1.17E-05	7.37E-04	1.01E-03	1.15E-03	4.09E-04	1.47E-04
1.17E-05	6.61E-04	1.16E-03	5.87E-04	-1.95E-04	3.94E-04
1.18E-05	1.35E-04	1.61E-03	9.75E-04	-3.26E-05	1.15E-04
1.18E-05	1.05E-03	7.76E-04	8.90E-04	-3.11E-04	7.86E-04
1.18E-05	8.51E-04	1.12E-03	1.04E-03	-6.86E-04	3.44E-08
1.19E-05	6.21E-04	9.37E-04	8.74E-04	-3.88E-04	-1.39E-04
1.19E-05	5.54E-04	1.28E-03	1.17E-03	-7.66E-04	6.48E-04
1.19E-05	4.74E-04	8.43E-04	1.24E-03	-6.81E-04	6.00E-04
1.20E-05	6.11E-04	9.14E-04	8.88E-04	-7.30E-04	2.42E-04
1.20E-05	1.13E-04	7.57E-04	1.07E-03	-4.23E-04	8.89E-04
1.20E-05	9.40E-04	1.08E-03	5.89E-04	-4.06E-04	6.22E-04
1.21E-05	4.49E-04	9.70E-04	7.92E-04	-1.18E-04	2.93E-04
1.21E-05	8.43E-04	9.63E-04	2.37E-04	-5.28E-04	5.10E-04
1.21E-05	2.75E-04	8.73E-04	6.43E-04	8.05E-05	7.56E-04
1.22E-05	6.85E-04	1.34E-03	6.65E-04	-2.54E-04	2.18E-04
1.22E-05	9.40E-04	1.12E-03	4.16E-04	-2.15E-04	2.68E-04
1.22E-05	6.82E-04	7.52E-04	7.77E-04	-3.35E-04	7.13E-04
1.23E-05	4.52E-04	6.07E-04	8.64E-04	-6.91E-04	7.09E-04
1.23E-05	9.00E-04	8.20E-04	1.14E-03	-7.87E-04	3.15E-04
1.23E-05	3.23E-04	1.08E-03	1.14E-03	-6.40E-04	7.39E-04
1.24E-05	3.76E-04	1.40E-03	9.67E-04	-4.44E-04	7.34E-04
1.24E-05	-1.38E-04	1.08E-03	8.10E-04	-6.14E-04	4.18E-04
1.24E-05	9.36E-04	1.03E-03	9.72E-04	-4.07E-05	5.50E-04
1.25E-05	5.86E-04	9.51E-04	9.47E-04	-3.21E-04	2.54E-04
1.25E-05	-1.93E-05	8.41E-04	6.92E-04	-6.22E-04	-7.42E-05
1.25E-05	-1.59E-05	1.17E-03	3.37E-04	-4.04E-04	6.61E-04
1.25E-05	3.89E-04	1.13E-03	8.07E-04	-1.99E-04	3.84E-04
1.26E-05	3.76E-06	4.95E-04	6.18E-04	-5.37E-04	7.47E-04
1.26E-05	2.96E-04	3.71E-04	8.73E-04	-3.74E-04	4.68E-04
1.26E-05	1.46E-04	9.31E-04	4.18E-04	-2.13E-04	4.14E-04

1.27E-05	7.51E-04	9.21E-04	6.16E-04	-7.95E-04	5.46E-04
1.27E-05	2.17E-04	1.21E-03	-1.45E-05	-4.61E-04	6.41E-04
1.27E-05	4.48E-04	1.33E-03	4.02E-04	-1.48E-04	3.97E-04
1.28E-05	6.07E-04	1.36E-03	1.29E-03	-3.20E-04	2.34E-06
1.28E-05	2.67E-04	1.02E-03	1.19E-03	-5.19E-04	7.38E-04
1.28E-05	3.59E-04	1.10E-03	-1.60E-04	-1.86E-04	4.73E-04
1.29E-05	6.29E-04	9.63E-04	9.54E-04	-5.52E-04	4.51E-04
1.29E-05	-7.18E-05	9.04E-04	1.06E-04	-1.17E-04	1.89E-04
1.29E-05	9.38E-05	1.06E-03	3.49E-04	-1.02E-04	5.94E-04
1.30E-05	7.41E-04	9.04E-04	3.25E-04	-1.15E-04	4.67E-04
1.30E-05	3.07E-04	6.94E-04	7.44E-04	-4.46E-05	7.81E-04
1.30E-05	9.02E-04	8.27E-04	6.00E-04	-4.00E-04	5.01E-04
1.31E-05	8.81E-04	9.58E-04	7.90E-04	-1.63E-04	8.05E-04
1.31E-05	4.56E-04	8.99E-04	7.14E-04	-4.60E-04	7.65E-04
1.31E-05	1.50E-04	6.62E-04	7.87E-04	-5.07E-04	-4.12E-05
1.32E-05	6.33E-04	1.10E-03	1.04E-03	-4.11E-04	4.32E-04
1.32E-05	4.91E-04	6.31E-04	-2.53E-04	-2.47E-05	2.33E-04
1.32E-05	-1.27E-04	1.48E-03	5.21E-04	-6.81E-04	7.67E-04
1.33E-05	6.60E-04	8.48E-04	8.39E-04	-2.81E-04	5.10E-04
1.33E-05	4.92E-04	9.50E-04	7.90E-04	-1.21E-04	2.82E-04
1.33E-05	1.16E-04	8.12E-04	4.10E-04	-5.51E-04	2.36E-04
1.33E-05	1.02E-03	1.12E-03	4.17E-04	-2.96E-04	5.17E-04
1.34E-05	4.87E-04	1.08E-03	4.61E-04	-4.44E-04	8.29E-04
1.34E-05	8.25E-04	1.22E-03	1.78E-04	-7.87E-04	3.49E-04
1.34E-05	3.15E-04	1.20E-03	4.64E-04	-5.34E-04	1.15E-03
1.35E-05	7.71E-04	1.09E-03	4.99E-04	-7.29E-04	6.08E-04
1.35E-05	4.42E-04	1.27E-03	-8.89E-05	-5.76E-04	3.98E-04
1.35E-05	5.26E-04	1.04E-03	8.92E-05	-1.41E-04	5.54E-04
1.36E-05	-2.67E-05	1.38E-03	2.56E-04	-2.59E-04	6.93E-04
1.36E-05	3.23E-04	1.03E-03	4.03E-04	-6.57E-04	2.57E-04
1.36E-05	-4.66E-05	1.20E-03	6.70E-04	-1.82E-04	1.05E-03
1.37E-05	3.67E-04	1.40E-03	1.72E-04	-3.74E-04	7.36E-04
1.37E-05	2.62E-05	1.39E-03	4.86E-04	7.79E-05	4.14E-04
1.37E-05	1.89E-04	8.19E-04	2.56E-04	-5.43E-04	-3.85E-05
1.38E-05	-1.74E-04	6.65E-04	1.51E-04	2.12E-04	4.51E-04
1.38E-05	4.74E-04	8.83E-04	6.11E-04	-5.02E-04	7.46E-04
1.38E-05	5.63E-04	1.09E-03	9.39E-04	-1.41E-06	6.33E-04
1.39E-05	3.89E-04	1.28E-03	4.39E-04	-9.23E-05	4.17E-04
1.39E-05	4.72E-04	1.15E-03	1.46E-04	-5.83E-04	9.55E-04
1.39E-05	1.11E-04	1.28E-03	2.84E-04	-7.66E-04	4.79E-04
1.40E-05	5.93E-04	1.21E-03	-2.78E-04	-5.18E-04	1.16E-03
1.40E-05	-1.63E-05	1.13E-03	4.57E-04	-6.64E-04	3.30E-04
1.40E-05	3.72E-04	1.41E-03	6.82E-04	-1.88E-04	7.71E-04
1.41E-05	2.25E-04	8.06E-04	7.95E-04	-8.30E-05	1.04E-03
1.41E-05	1.02E-03	9.15E-04	8.05E-04	-4.20E-04	8.11E-04
1.41E-05	4.64E-05	7.24E-04	2.03E-05	-9.36E-04	7.24E-04
1.41E-05	-3.11E-04	1.44E-03	4.60E-05	-5.32E-04	4.99E-04
1.42E-05	3.09E-04	1.10E-03	2.80E-04	-8.34E-04	6.00E-04
1.42E-05	1.99E-04	1.00E-03	6.61E-05	1.23E-04	2.14E-04
1.42E-05	3.37E-04	9.15E-04	7.50E-04	2.33E-04	3.12E-04
1.43E-05	2.21E-04	9.25E-04	4.60E-04	3.13E-05	4.96E-04
1.43E-05	3.89E-04	1.03E-03	1.58E-04	-2.64E-04	1.41E-03
1.43E-05	1.90E-04	4.77E-04	4.72E-04	-5.72E-04	1.10E-03
1.44E-05	2.57E-04	1.05E-03	2.00E-04	-6.66E-04	7.59E-04

1.44E-05	-4.16E-04	1.17E-03	7.24E-04	3.15E-04	7.14E-04
1.44E-05	9.46E-04	1.24E-03	4.62E-04	-2.02E-04	2.98E-04
1.45E-05	3.63E-04	1.12E-03	1.21E-04	-2.74E-04	8.47E-04
1.45E-05	6.48E-04	1.05E-03	1.46E-04	-2.76E-04	1.09E-03
1.45E-05	1.91E-05	9.48E-04	-4.09E-05	2.07E-04	4.87E-04
1.46E-05	2.47E-04	1.22E-03	6.79E-04	-6.57E-04	9.28E-04
1.46E-05	5.31E-04	1.50E-03	7.46E-04	1.94E-05	6.65E-04
1.46E-05	-5.91E-05	1.19E-03	3.89E-04	-6.83E-04	2.26E-04
1.47E-05	4.76E-04	1.41E-03	4.84E-04	1.35E-04	3.82E-04
1.47E-05	3.61E-04	1.26E-03	1.22E-04	-3.22E-04	4.06E-04
1.47E-05	3.32E-04	7.02E-04	3.32E-04	6.87E-06	8.36E-04
1.48E-05	-2.80E-04	1.33E-03	1.43E-04	-3.89E-04	4.17E-04
1.48E-05	-1.80E-04	1.10E-03	5.08E-04	-4.70E-04	6.68E-04
1.48E-05	2.90E-04	9.17E-04	-1.49E-04	-5.07E-04	8.64E-04
1.49E-05	4.31E-04	1.08E-03	-4.70E-06	-9.84E-05	8.40E-04
1.49E-05	1.78E-04	1.27E-03	3.74E-05	1.39E-04	8.90E-04
1.49E-05	4.82E-04	1.03E-03	1.08E-03	-5.56E-04	5.13E-04
1.49E-05	1.53E-04	7.77E-04	4.36E-04	4.61E-04	6.52E-04
1.50E-05	-2.96E-04	1.07E-03	9.26E-04	-7.85E-04	9.87E-04

Table SI.4: Primary kinetic data for the reaction of 1-ethyl-2-pyrrolidone (**S4**) with CumO• generated by 355 nm LFP in MeCN (**Figure SI.4**).

**t / s**	**ΔA 1-ethyl-2-pyrrolidone (S4)**				
	**0.044M**	**0.109M**	**0.194M**	**0.278M**	**0.381M**
-1E-06	0.00109	0.00047	-0.0005	0.00023	-0.0013
-1E-06	0.00089	0.00124	0.00017	0.00011	-0.0021
-9E-07	0.0011	0.00083	0.00056	-0.0008	-0.0029
-9E-07	0.00147	0.00033	-0.0007	-0.0006	-0.0021
-9E-07	3.1E-05	0.00094	0.00015	-0.0006	-0.0016
-8E-07	0.00048	0.00073	0.00012	-0.0004	-9E-05
-8E-07	0.00046	0.00024	4.8E-05	-0.0006	-0.0019
-8E-07	0.00068	0.00152	0.00015	0.00022	-0.0011
-7E-07	0.00095	0.0008	-6E-05	-0.0005	-0.0021
-7E-07	0.00125	0.00024	0.00033	-0.0006	-0.0021
-7E-07	0.00081	0.00106	0.00066	-0.0005	-0.0013
-6E-07	0.00141	0.00086	0.0003	-0.0005	-0.0018
-6E-07	0.00143	0.00053	-0.0002	-0.0002	-0.0022
-6E-07	0.00051	0.00077	-0.0007	-0.0002	-0.0024
-5E-07	0.00089	0.0002	-0.0006	-0.0006	-0.0014
-5E-07	0.00046	0.00157	-0.0006	0.00031	-0.0025
-5E-07	0.00064	0.00029	0.0005	0.00011	-0.0018
-4E-07	0.00075	0.00086	-0.0005	-0.0008	-0.0017
-4E-07	0.00171	0.00059	0.00016	-0.0007	-0.0018
-4E-07	0.00092	0.00115	0.00024	0.00012	-0.0019
-4E-07	0.00025	0.0006	0.00017	-0.0012	-0.0021
-3E-07	0.00084	0.00037	-0.0007	-0.0004	-0.0019
-3E-07	0.00044	0.0005	-9E-05	-3E-05	-0.0019
-3E-07	0.00072	0.00057	-0.0002	-0.0008	-0.0018
-2E-07	0.00075	5.4E-05	0.00021	-0.0012	-0.0009
-2E-07	0.00019	0.00091	-8E-05	-0.0006	-0.0016
-2E-07	0.00014	0.00046	-0.0005	0.00018	-0.0019
-1E-07	0.00045	0.00086	-0.0006	-0.0001	-0.002
-1E-07	0.00088	0.00101	-0.0006	-0.0003	-0.0022
-6E-08	5.4E-05	0.00044	-1E-05	-0.0007	-0.0017
-3E-08	0.00066	0.00093	-0.0005	-0.0002	-0.0022
-2E-10	-0.0016	-0.003	-0.0028	-0.0037	-0.0058
3.2E-08	-0.0125	-0.0176	-0.0163	-0.0179	-0.0199
6.4E-08	0.01063	0.00757	0.00292	-0.0007	-0.006
9.6E-08	0.01448	0.01425	0.01302	0.01235	0.00909
1.3E-07	0.01545	0.01503	0.01293	0.01229	0.00979
1.6E-07	0.0141	0.01333	0.01291	0.01242	0.00893
1.9E-07	0.01402	0.0141	0.01214	0.01098	0.00882
2.2E-07	0.01476	0.0134	0.01139	0.01089	0.00828
2.6E-07	0.01431	0.0131	0.01055	0.01036	0.00695
2.9E-07	0.01292	0.01249	0.0108	0.00971	0.00669
3.2E-07	0.01293	0.01188	0.01053	0.00938	0.00569
3.5E-07	0.01234	0.0105	0.00943	0.00861	0.00557
3.8E-07	0.01154	0.01115	0.009	0.00831	0.00448
4.2E-07	0.0124	0.01071	0.00828	0.00774	0.00436
4.5E-07	0.01102	0.00981	0.00775	0.00704	0.00367
4.8E-07	0.011	0.00937	0.00717	0.00709	0.00389
5.1E-07	0.01074	0.00952	0.00757	0.00619	0.00298
5.4E-07	0.01053	0.00901	0.00784	0.00538	0.00319

5.8E-07	0.00996	0.00947	0.00671	0.00508	0.00309
6.1E-07	0.01016	0.00931	0.00647	0.00522	0.00199
6.4E-07	0.00986	0.00801	0.00602	0.0051	0.00241
6.7E-07	0.00983	0.00738	0.00623	0.00426	0.00169
7E-07	0.0093	0.00759	0.00511	0.00462	0.00146
7.4E-07	0.00899	0.00696	0.005	0.00519	0.00144
7.7E-07	0.00827	0.00691	0.00493	0.00355	0.00053
8E-07	0.00868	0.00698	0.0047	0.00331	0.00084
8.3E-07	0.00774	0.00693	0.00499	0.00304	0.0007
8.6E-07	0.00712	0.00639	0.00406	0.00302	0.00051
9E-07	0.00802	0.00631	0.00445	0.00221	-0.0002
9.3E-07	0.0078	0.00608	0.00345	0.00211	0.00021
9.6E-07	0.00834	0.00547	0.00442	0.00326	0.00011
9.9E-07	0.0064	0.0052	0.00337	0.00202	-2E-05
1E-06	0.00685	0.00568	0.00336	0.00174	-0.0004
1.1E-06	0.00693	0.00499	0.00315	0.00166	-0.0006
1.1E-06	0.00613	0.00472	0.00315	0.0016	-9E-05
1.1E-06	0.00633	0.0048	0.00297	0.00215	0.00021
1.2E-06	0.0058	0.00497	0.00285	0.00134	-0.001
1.2E-06	0.00622	0.0046	0.0028	0.00177	-0.0009
1.2E-06	0.00584	0.00453	0.0026	0.00147	-0.0008
1.2E-06	0.00563	0.00405	0.00234	0.00194	-0.0007
1.3E-06	0.00555	0.00412	0.00206	0.00169	-0.0007
1.3E-06	0.00535	0.00439	0.00164	0.00123	-0.0007
1.3E-06	0.00509	0.00433	0.00209	0.00169	-0.0013
1.4E-06	0.00512	0.00352	0.00169	0.00018	-0.0009
1.4E-06	0.0062	0.0034	0.00126	0.00142	-0.0007
1.4E-06	0.00538	0.00439	0.00139	0.00145	-0.0004
1.5E-06	0.00476	0.00409	0.00209	0.0013	-0.0007
1.5E-06	0.00457	0.00317	0.00223	0.0013	-0.0012
1.5E-06	0.0052	0.00356	0.00161	0.00085	-0.0015
1.6E-06	0.00473	0.00374	0.00189	0.00073	-0.0014
1.6E-06	0.00446	0.0035	0.00168	0.00077	-0.0008
1.6E-06	0.00429	0.00396	0.00232	0.00142	-0.0014
1.7E-06	0.00449	0.00333	0.00155	0.00057	-0.0005
1.7E-06	0.00413	0.00332	0.00116	0.00115	-0.0007
1.7E-06	0.00479	0.00327	0.0015	0.00034	-0.0009
1.8E-06	0.00516	0.0024	0.00063	0.00023	-0.0012
1.8E-06	0.00382	0.00291	0.00173	0.00078	-0.0014
1.8E-06	0.0036	0.00259	0.00139	0.00081	-0.0017
1.9E-06	0.00369	0.00243	0.00074	0.00094	-0.0011
1.9E-06	0.0036	0.00236	0.00078	0.00033	-0.0013
1.9E-06	0.00402	0.00211	0.00095	-0.0002	-0.0011
2E-06	0.00385	0.00168	0.00135	0.00042	-0.0013
2E-06	0.00383	0.00247	0.00079	0.00098	-0.0011
2E-06	0.00357	0.00263	0.00156	0.00073	-0.0012
2E-06	0.00367	0.00121	0.00045	0.00018	-0.0017
2.1E-06	0.00288	0.00258	0.00068	0.00068	-0.0014
2.1E-06	0.00299	0.0024	0.00112	0.00047	-0.001
2.1E-06	0.00312	0.00268	0.0009	-7E-05	-0.0008
2.2E-06	0.00302	0.00203	0.0008	0.00036	-0.0012
2.2E-06	0.00291	0.00173	0.0015	0.00078	-0.0006
2.2E-06	0.00276	0.00214	0.00104	0.00047	-0.0007
2.3E-06	0.0031	0.00223	0.0012	0.00061	-0.0008

2.3E-06	0.00244	0.00196	0.00083	0.0007	-0.001
2.3E-06	0.00264	0.00262	0.00098	0.00031	-0.0009
2.4E-06	0.00189	0.00222	0.00071	0.00032	-0.0012
2.4E-06	0.00248	0.00233	0.00097	0.00079	-0.0014
2.4E-06	0.00205	0.00155	0.0011	0.00072	-0.0014
2.5E-06	0.00232	0.00208	0.00097	0.00096	-0.0015
2.5E-06	0.00229	0.00136	0.00093	-5E-05	-0.0017
2.5E-06	0.00204	0.00188	0.0011	0.00099	-0.0012
2.6E-06	0.00208	0.00239	0.00131	7.6E-05	-0.0016
2.6E-06	0.00256	0.00211	0.00076	0.00064	-0.0011
2.6E-06	0.0018	0.00121	0.00101	0.00074	-0.0011
2.7E-06	0.00192	0.00175	0.00085	0.00068	-0.0015
2.7E-06	0.00203	0.00261	0.0004	0.001	-0.0012
2.7E-06	0.0024	0.00176	0.00095	0.00022	-0.0014
2.8E-06	0.00127	0.00057	0.00031	-0.0005	-0.0013
2.8E-06	0.00159	0.00226	0.00076	0.00036	-0.0012
2.8E-06	0.00227	0.00182	3.3E-05	-0.0004	-0.0019
2.8E-06	0.00268	0.00148	0.00084	0.00142	-0.0016
2.9E-06	0.00156	0.00188	0.00072	1.3E-05	-0.0013
2.9E-06	0.00263	0.00154	0.0013	0.00066	-0.0012
2.9E-06	0.00175	0.00158	0.001	0.00032	-0.0011
3E-06	0.00176	0.00212	0.00125	0.00049	-0.0016
3E-06	0.0028	0.00167	0.00049	-8E-06	-0.0024
3E-06	0.0019	0.00137	0.00036	-0.0002	-0.002
3.1E-06	0.00171	0.00151	0.00081	-0.0006	-0.0016
3.1E-06	0.0019	0.00217	0.00048	0.0008	-0.0017
3.1E-06	0.00141	0.00221	0.00057	0.00041	-0.0009
3.2E-06	0.00237	0.00213	0.00085	0.00034	-0.0011
3.2E-06	0.00203	0.00146	0.00062	0.00043	-0.0018
3.2E-06	0.00209	0.00131	0.00105	0.00097	-0.0016
3.3E-06	0.00203	0.00122	-0.0001	8.9E-05	-0.001
3.3E-06	0.0016	0.00134	0.00123	0.00012	-0.0012
3.3E-06	0.00161	0.00152	0.0003	0.00079	-0.0018
3.4E-06	0.00163	0.00182	0.00069	0.00021	-0.0005
3.4E-06	0.00208	0.00132	0.00129	0.00066	-0.001
3.4E-06	0.00237	0.00151	0.00045	0.0003	-0.0013
3.5E-06	0.00208	0.00137	0.00082	0.0005	-0.0021
3.5E-06	0.00209	0.00101	-0.0004	-0.0002	-0.0021
3.5E-06	0.00189	0.00123	0.00037	0.00043	-0.0014
3.6E-06	0.00247	0.0012	0.00076	0.00055	-0.0015
3.6E-06	0.0021	0.00097	0.00091	0.00055	-0.0017
3.6E-06	0.00137	0.00112	0.00076	4.9E-05	-0.001
3.6E-06	0.00146	0.00124	0.00065	0.00028	-0.002
3.7E-06	0.0016	0.00137	0.00058	0.00014	-0.001
3.7E-06	0.00129	0.00141	0.00042	0.00056	-0.0005
3.7E-06	0.00182	0.00138	0.0004	0.00078	-0.0013
3.8E-06	0.00146	0.00048	0.0006	0.00042	-0.0023
3.8E-06	0.00118	0.00106	0.00074	0.0003	-0.0021
3.8E-06	0.00123	0.00143	0.00065	-0.0003	-0.0018
3.9E-06	0.00081	0.00187	0.00109	8.3E-05	-0.0013
3.9E-06	0.00137	0.00119	0.00058	-6E-05	-0.0014
3.9E-06	0.00208	0.00118	0.0005	0.00067	-0.0018
4E-06	0.00206	0.00151	0.00032	0.00068	-0.0015
4E-06	0.00211	0.00159	-0.0004	3.7E-05	-0.0015

4E-06	0.0013	0.00146	0.0003	0.00026	-0.0011
4.1E-06	0.0014	0.0011	0.00103	0.00053	-0.0014
4.1E-06	0.00081	0.00106	0.00056	-0.0003	-0.0018
4.1E-06	0.00146	0.00083	0.00078	0.00056	-0.0023
4.2E-06	0.00092	0.00116	0.00046	0.00046	-0.0018
4.2E-06	0.00158	0.00146	0.00012	0.00091	-0.0012
4.2E-06	0.00126	0.00147	0.00055	0.00159	-0.0016
4.3E-06	0.00143	0.0013	0.00022	0.00037	-0.0012
4.3E-06	0.00145	0.00118	0.00035	-0.0002	-0.0014
4.3E-06	0.00118	0.00144	0.00093	0.00045	-0.001
4.4E-06	0.00137	0.00056	5.7E-05	0.00088	-0.0018
4.4E-06	0.00165	0.00089	0.00072	0.00047	-0.0014
4.4E-06	0.00074	0.00146	0.00043	-2E-05	-0.0014
4.4E-06	0.00071	0.00119	0.00056	0.00072	-0.0017
4.5E-06	0.002	0.00108	0.00058	0.00041	-0.0027
4.5E-06	0.0012	0.00169	2.5E-05	4.6E-05	-0.0022
4.5E-06	0.00156	0.00119	0.00083	-0.0003	-0.0016
4.6E-06	0.00095	0.00096	0.00051	0.00057	-0.0012
4.6E-06	0.00131	0.00175	0.00036	-0.0002	-0.0014
4.6E-06	0.0012	0.00115	0.00065	0.00052	-0.0021
4.7E-06	0.00071	0.00085	0.00078	0.00022	-0.002
4.7E-06	0.00129	0.00162	0.00033	0.00053	-0.0017
4.7E-06	0.00072	0.00081	0.00075	-0.0001	-0.001
4.8E-06	0.00134	0.00118	0.00034	0.00056	-0.0011
4.8E-06	0.00064	0.00102	0.00055	0.00053	-0.0009
4.8E-06	0.00052	0.00079	0.0003	0.00036	-0.0021
4.9E-06	0.00118	0.00064	0.0002	0.00052	-0.0009
4.9E-06	0.00158	0.00134	0.00013	-2E-05	-0.0008
4.9E-06	0.00146	0.00082	-6E-05	-8E-06	-0.0015
5E-06	0.00128	0.00164	0.00072	5.8E-05	-0.002
5E-06	0.00067	0.00121	0.00076	0.0002	-0.001
5E-06	0.00149	0.0012	-3E-05	0.00073	-0.0014
5.1E-06	0.0017	0.0012	0.00042	7E-05	-0.0019
5.1E-06	0.00132	0.00145	0.00049	-6E-06	-0.0017
5.1E-06	0.00105	0.00067	0.00051	0.00038	-0.0016
5.2E-06	0.00103	0.00042	0.00033	4E-05	-0.0017
5.2E-06	0.00132	0.00122	0.00065	0.0007	-0.0024
5.2E-06	0.00194	0.00101	7.6E-05	9E-05	-0.0023
5.2E-06	0.00141	0.00088	0.00019	-0.0002	-0.0017
5.3E-06	0.00139	0.00069	8E-05	-7E-05	-0.0022
5.3E-06	0.00173	0.00112	0.00055	-1E-04	-0.0015
5.3E-06	0.0011	0.00104	0.00094	0.0008	-0.0015
5.4E-06	0.00146	0.00128	0.00033	0.0007	-0.0018
5.4E-06	0.00149	0.00036	0.00089	0.00018	-0.0016
5.4E-06	0.00112	0.00129	0.00032	-0.0002	-0.0015
5.5E-06	0.00086	0.00192	0.00055	0.00017	-0.0016
5.5E-06	0.0016	0.00161	0.00034	3.3E-05	-0.0008
5.5E-06	0.00096	0.00113	7.1E-05	0.00024	-0.0018
5.6E-06	0.00137	0.00118	0.00066	0.0007	-0.0017
5.6E-06	0.00096	0.00087	0.00028	0.00057	-0.0015
5.6E-06	0.00039	0.00093	0.00073	-4E-05	-0.0014
5.7E-06	0.0008	0.00078	0.00076	0.00014	-0.0017
5.7E-06	0.00121	0.00115	0.00114	0.00064	-0.0013
5.7E-06	0.00086	0.00139	0.00058	0.00026	-0.0014

5.8E-06	0.00112	0.00095	0.00089	-0.0003	-0.0016
5.8E-06	0.00107	0.0013	0.00105	0.00028	-0.0018
5.8E-06	0.00063	0.00095	0.0002	0.00058	-0.0012
5.9E-06	0.00099	0.00135	0.00035	0.00039	-0.0018
5.9E-06	0.00131	0.00115	0.00098	0.00037	-0.0011
5.9E-06	0.0009	0.00104	0.00078	0.0004	-0.002
6E-06	0.00151	0.00114	0.00046	0.00056	-0.0014
6E-06	0.00086	0.00112	7E-05	0.00026	-0.0026
6E-06	0.00228	0.00137	0.00062	0.00036	-0.0022
6E-06	0.00098	0.00035	0.00078	-4E-05	-0.0015
6.1E-06	0.0005	0.00104	0.00053	-0.0003	-0.0017
6.1E-06	0.00099	0.00106	0.0004	0.00042	-0.0016
6.1E-06	0.0015	0.00196	0.00011	0.00059	-0.0019
6.2E-06	0.00086	0.00114	0.00019	0.00044	-0.0017
6.2E-06	0.00164	0.00083	0.0003	0.00013	-0.0021
6.2E-06	0.00076	0.00096	0.00056	0.00031	-0.0015
6.3E-06	0.00137	0.00087	-1E-05	0.00026	-0.0013
6.3E-06	0.00075	0.00068	0.00035	0.00035	-0.0012
6.3E-06	0.002	0.00127	8.8E-05	0.00079	-0.0015
6.4E-06	0.00042	0.00149	0.00049	-0.0004	-0.0014
6.4E-06	0.00124	0.00125	7.2E-05	0.00045	-0.0018
6.4E-06	0.001	0.00066	0.00042	9E-05	-0.0009
6.5E-06	0.00058	0.00134	0.00039	0.00083	-0.0019
6.5E-06	0.0013	0.00189	0.00058	0.00099	-0.0012
6.5E-06	0.00111	0.00161	0.0011	-7E-05	-0.0014
6.6E-06	0.00093	0.00082	0.00084	0.00027	-0.0014
6.6E-06	0.00075	0.00111	0.00069	0.00012	-0.0007
6.6E-06	0.00074	0.00151	0.00091	0.00032	-0.0018
6.7E-06	0.00111	0.00153	0.00126	0.00054	-0.0016
6.7E-06	0.00089	0.00084	0.00069	0.00021	-0.0015
6.7E-06	0.00092	0.00117	0.00016	0.00015	-0.0012
6.8E-06	0.00147	0.00094	0.00045	5.4E-05	-0.0007
6.8E-06	0.00119	0.00102	0.00025	0.00084	-0.0012
6.8E-06	0.00134	0.00095	0.00107	0.00017	-0.0017
6.8E-06	0.00164	0.00137	0.00098	0.00022	-0.0016
6.9E-06	0.00092	0.00147	0.00031	0.00065	-0.0012
6.9E-06	0.00093	0.00159	0.0003	0.00061	-0.0017
6.9E-06	0.00098	0.00109	0.00111	-0.0002	-0.0005
7E-06	0.00075	0.0015	0.0004	0.00062	-0.0007
7E-06	0.00121	0.00046	0.00054	0.00037	-0.0011
7E-06	0.00146	0.00094	0.00078	0.00018	-0.0012
7.1E-06	0.00133	0.00152	0.00055	0.00023	-0.0009
7.1E-06	0.00179	0.00156	0.00045	0.00038	-0.001
7.1E-06	0.00155	0.00118	0.00048	-4E-05	-0.0017
7.2E-06	0.00143	0.00095	0.0005	2.5E-05	-0.001
7.2E-06	0.0008	0.00106	-0.0004	0.00044	-0.0017
7.2E-06	0.00176	0.00095	-6E-05	-0.0002	-0.0018
7.3E-06	0.00135	0.00076	0.00057	0.00019	-0.0012
7.3E-06	0.00143	0.00126	0.00062	0.00029	-0.0018
7.3E-06	0.00045	0.00126	0.00044	-2E-05	-0.0013
7.4E-06	0.00082	0.00063	5E-05	-0.0003	-0.0009
7.4E-06	0.00083	0.00131	9.6E-05	0.00103	-0.0017
7.4E-06	0.00133	0.00067	0.00079	-0.0002	-0.002
7.5E-06	0.00084	0.00102	0.00035	0.0003	-0.0019

7.5E-06	0.00084	0.0006	7.9E-06	0.00048	-0.0014
7.5E-06	0.00193	0.00063	0.00019	0.00052	-0.0018
7.6E-06	0.00119	0.00115	0.00107	-0.0006	-0.0014
7.6E-06	0.00157	0.00098	0.00057	0.00051	-0.0016
7.6E-06	0.00082	0.00045	0.00086	-0.0003	-0.001
7.6E-06	0.00128	0.00032	0.00068	0.00033	-0.0022
7.7E-06	0.00084	0.0009	0.00078	7.3E-05	-0.0027
7.7E-06	0.00134	0.0005	2.2E-05	0.00083	-0.0021
7.7E-06	0.00087	0.00128	2.8E-05	0.00044	-0.0019
7.8E-06	0.00151	0.00149	-0.0001	0.0003	-0.0014
7.8E-06	0.00082	0.00156	9.3E-05	6.9E-05	-0.0013
7.8E-06	0.00102	0.00098	0.00019	0.00027	-0.0014
7.9E-06	0.00121	0.00116	0.0009	-9E-05	-0.0013
7.9E-06	0.00066	0.00152	0.00019	0.0004	-0.0018
7.9E-06	0.00114	0.00053	0.00095	0.00061	-0.0019
8E-06	0.0012	0.00074	0.00028	0.00024	-0.0015
8E-06	0.00098	0.00091	0.00032	0.00045	-0.0009
8E-06	0.00112	0.00132	7.3E-05	0.0006	-0.0014
8.1E-06	0.00055	0.00121	-8E-06	-0.0005	-0.0026
8.1E-06	0.00073	0.00144	-4E-05	-4E-05	-0.0014
8.1E-06	0.00126	0.00144	0.00036	0.00078	-0.0009
8.2E-06	0.00133	0.00188	0.00046	0.00061	-0.0015
8.2E-06	0.00105	0.00101	-0.0002	-9E-05	-0.0011
8.2E-06	0.00119	0.00109	0.00103	-0.0002	-0.0027
8.3E-06	0.00123	0.00161	0.00075	0.00049	-0.0014
8.3E-06	0.00152	0.00132	0.00066	0.00014	-0.0013
8.3E-06	0.00134	0.00105	0.00018	0.00015	-0.0018
8.4E-06	0.0009	0.00143	8.3E-05	0.00031	-0.0022
8.4E-06	0.00207	0.00082	9.9E-05	0.00042	-0.0011
8.4E-06	0.00185	0.00122	9.5E-05	0.00019	-0.0019
8.4E-06	0.00112	0.00064	0.00072	9.6E-05	-0.0018
8.5E-06	0.00045	0.00132	0.00069	0.00074	-0.0021
8.5E-06	0.0011	0.00113	-5E-05	-4E-05	-0.003
8.5E-06	0.00063	0.00094	5.3E-05	0.0003	-0.0009
8.6E-06	0.00068	0.00039	-0.0002	-0.0002	-0.002
8.6E-06	0.00113	0.001	0.00077	0.00067	-0.0013
8.6E-06	0.00069	0.00111	0.00026	0.00014	-0.0016
8.7E-06	0.00063	0.00077	-0.0002	9.1E-05	-0.0017
8.7E-06	0.00062	0.00128	-0.0002	0.00032	-0.0018
8.7E-06	0.00032	0.00113	0.00023	-0.0002	-0.001
8.8E-06	0.00115	0.00129	0.00014	0.0001	-0.0015
8.8E-06	0.00095	0.00121	0.00019	0.00049	-0.0008
8.8E-06	0.0011	0.00089	0.00033	0.00125	-0.0023
8.9E-06	0.00094	0.00112	0.00053	0.00071	-0.0019
8.9E-06	0.00113	0.00112	0.00018	0.00038	-0.0009
8.9E-06	0.0008	0.00129	-0.0001	0.00026	-0.001
9E-06	0.00127	0.00111	0.00031	0.00034	-0.0018
9E-06	0.00099	0.00117	1.2E-05	0.00033	-0.0011
9E-06	0.00072	0.0012	9.5E-05	-0.0006	-0.0015
9.1E-06	0.00094	0.00096	0.00055	-7E-05	-0.0007
9.1E-06	0.00125	0.00164	0.00018	-0.0003	-0.0019
9.1E-06	0.00127	0.0014	0.00027	-3E-05	-0.0012
9.2E-06	0.00147	0.00054	0.0006	-0.0007	-0.0011
9.2E-06	0.00137	0.00028	0.00066	-0.0004	-0.0013

9.2E-06	0.00083	0.00144	0.00092	-9E-05	-0.0016
9.2E-06	0.00103	0.00081	-0.0002	4.2E-06	-0.0024
9.3E-06	0.00191	0.00094	0.00038	-0.0001	-0.002
9.3E-06	0.00108	0.00119	0.00074	-0.0004	-0.0018
9.3E-06	0.00172	0.0014	-0.0003	-0.0003	-0.0013
9.4E-06	0.00089	0.00133	0.00045	-0.0004	-0.0018
9.4E-06	0.00197	0.0007	7E-05	-6E-05	-0.0016
9.4E-06	0.0017	0.00115	0.00062	-0.0001	-0.0019
9.5E-06	0.00143	0.00132	0.00071	-0.0002	-0.0012
9.5E-06	0.00141	0.00074	0.00017	0.00026	-0.0016
9.5E-06	0.00125	0.00117	-0.0002	0.0005	-0.0004
9.6E-06	0.00173	0.00084	4.6E-05	0.00055	-0.0012
9.6E-06	0.00122	0.00096	-0.0001	0.00071	-0.002
9.6E-06	0.00096	0.00132	-1E-04	0.00114	-0.0016
9.7E-06	0.00097	0.0015	0.00028	0.00046	-0.001
9.7E-06	0.00204	0.00065	0.00015	-0.0002	-0.0019
9.7E-06	0.00122	0.00115	0.00036	0.0007	-0.0016
9.8E-06	0.00134	0.00136	9.5E-05	0.00085	-0.002
9.8E-06	0.00058	0.00066	0.0004	0.00051	-0.0024
9.8E-06	0.00147	0.0015	-6E-05	-0.0002	-0.002
9.9E-06	0.00098	0.00049	0.00051	-0.0003	-0.0015
9.9E-06	0.00112	0.00073	0.00085	-0.0003	-0.0016
9.9E-06	0.00121	0.00126	-0.0001	-3E-07	-0.0013
1E-05	0.00099	0.00117	0.00034	-0.0002	-0.002
1E-05	0.00116	0.00027	0.00033	0.00034	-0.0021
1E-05	0.00203	0.00113	0.0006	-0.0002	-0.0015
1E-05	0.0013	0.00154	0.00093	-1E-04	-0.0019
1E-05	0.00136	0.00096	0.00048	0.00049	-0.0013
1E-05	0.0016	0.00103	0.00091	-0.0001	-0.0014
1E-05	0.00103	0.00025	0.0001	0.00026	-0.0018
1E-05	0.00077	0.00121	-6E-05	0.00012	-0.0016
1E-05	0.00111	0.00114	0.00026	0.00037	-0.0014
1E-05	0.00161	0.00115	0.00051	-8E-05	-0.0018
1E-05	0.00199	0.00102	0.00019	-0.0006	-0.0011
1E-05	0.001	0.00066	0.0001	-0.0001	-0.0017
1E-05	0.00178	0.00109	0.00028	0.00036	-0.0022
1E-05	0.00102	0.00045	0.00059	0.00045	-0.0016
1E-05	0.00112	0.00095	0.00091	-0.0002	-0.0014
1E-05	0.00077	0.00157	0.00064	0.0001	-0.0027
1E-05	0.00193	0.00203	0.00015	0.00044	-0.0013
1.1E-05	0.00121	0.00168	0.00045	-0.0002	-0.0015
1.1E-05	0.00066	0.00091	0.00015	6.1E-05	-0.0011
1.1E-05	0.0022	0.00072	6.8E-05	-0.0003	-0.0011
1.1E-05	0.00126	0.00164	0.00015	-0.0004	-0.0022
1.1E-05	0.0005	0.00069	5.2E-06	-0.0002	-0.0025
1.1E-05	0.00124	0.00114	0.00088	0.00041	-0.0016
1.1E-05	0.00141	0.00071	0.0004	-0.0003	-0.0012
1.1E-05	0.00155	0.00157	0.00034	0.00033	-0.0014
1.1E-05	0.0013	0.0016	-0.0003	-0.0003	-0.0017
1.1E-05	0.00165	0.00052	0.00077	0.00085	-0.0019
1.1E-05	0.00123	0.00123	0.00094	-0.0007	-0.0011
1.1E-05	0.00191	0.0011	-0.0002	0.00045	-0.002
1.1E-05	0.00144	0.00137	0.00027	-0.0001	-0.0016
1.1E-05	0.00169	0.00092	-3E-05	-0.0002	-0.0016

1.1E-05	0.00126	0.00094	-0.0005	-5E-05	-0.0017
1.1E-05	0.00142	0.00045	9.3E-05	-0.0008	-0.0024
1.1E-05	0.00183	0.0009	0.00028	7.8E-05	-0.0015
1.1E-05	0.00158	0.00145	0.00012	-0.0002	-0.0011
1.1E-05	0.00176	0.00033	0.00036	0.00053	-0.0013
1.1E-05	0.00064	-3E-05	-0.0006	0.00118	-0.0019
1.1E-05	0.00151	0.00075	-0.0002	-3E-05	-0.0015
1.1E-05	0.00091	0.00128	0.00052	-4E-05	-0.0012
1.1E-05	0.00149	0.00099	0.00036	0.00014	-0.0016
1.1E-05	0.00193	0.00048	0.00057	0.00047	-0.0017
1.1E-05	0.00139	0.00105	0.00098	0.00021	-0.0019
1.1E-05	0.00193	0.00084	0.00009	-0.0004	-0.0023
1.1E-05	0.00089	0.00127	0.00022	0.00026	-0.0016
1.1E-05	0.00177	0.00019	0.0006	0.00076	-0.0016
1.1E-05	0.00166	0.00191	0.00108	-0.0004	-0.0015
1.1E-05	0.00109	0.00125	0.00075	0.00048	-0.0016
1.1E-05	0.00117	0.00103	-3E-05	0.00081	-0.0021
1.1E-05	0.00079	0.00148	0.00037	-0.0004	-0.0017
1.2E-05	0.00189	0.0014	0.00056	4.8E-05	-0.0017
1.2E-05	0.00156	0.00112	8E-05	0.00043	-0.0012
1.2E-05	0.00176	0.00139	0.00016	-0.0004	-0.0017
1.2E-05	0.00121	0.0005	-0.0005	-7E-06	-0.0018
1.2E-05	0.00122	0.00145	-8E-05	0.00025	-0.0018
1.2E-05	0.00119	0.00114	0.00028	-0.0003	-0.0019
1.2E-05	0.00163	0.00185	0.00024	0.00059	-0.0018
1.2E-05	0.00142	0.00127	-0.0006	-0.0004	-0.0021
1.2E-05	0.00196	0.00131	-0.0005	0.0002	-0.0014
1.2E-05	0.00154	0.00049	-3E-05	-0.0002	-0.0016
1.2E-05	0.0016	0.00051	0.00103	0.00016	-0.0012
1.2E-05	0.0019	0.00022	0.00036	-0.0006	-0.0017
1.2E-05	0.00178	0.00124	0.00046	-0.0003	-0.0019
1.2E-05	0.00086	0.00223	0.00035	-0.0003	-0.0016
1.2E-05	0.00198	0.00154	0.00075	-6E-05	-0.0019
1.2E-05	0.00097	0.00069	0.00072	-5E-05	-0.0014
1.2E-05	0.00079	0.00065	7.8E-05	0.00027	-0.0023
1.2E-05	0.0016	0.0014	0.00105	-0.0001	-0.0011
1.2E-05	0.00141	0.00077	0.00064	0.00023	-0.0019
1.2E-05	0.00148	0.00078	0.0006	0.00036	-0.0017
1.2E-05	0.00128	0.00056	0.00081	-0.0003	-0.0014
1.2E-05	0.00206	0.00161	0.00045	0.00048	-0.0016
1.2E-05	0.00145	0.00172	0.00114	-0.0002	-0.0016
1.2E-05	0.00139	0.0006	0.00035	0.0001	-0.0027
1.2E-05	0.00115	0.00059	0.00042	0.0005	-0.0015
1.2E-05	0.00105	-6E-05	0.00077	0.00043	-0.001
1.2E-05	0.00163	0.0005	0.0004	0.00076	-0.0019
1.2E-05	0.00145	0.00086	0.00056	0.00066	-0.0019
1.2E-05	0.00118	0.00116	-0.0004	0.0002	-0.0012
1.2E-05	0.002	0.00122	-0.0002	0.00036	-0.0016
1.2E-05	0.00129	0.00114	0.00099	0.00059	-0.0018
1.3E-05	0.00174	0.00189	0.00067	0.00074	-0.0019
1.3E-05	0.00124	0.00083	0.00017	-0.0004	-0.0016
1.3E-05	0.00118	0.00088	0.00074	0.00027	-0.0024
1.3E-05	0.0014	0.00096	0.00081	0.00092	-0.0017
1.3E-05	0.00119	0.00181	0.00078	0.00038	-0.0008

1.3E-05	0.00186	0.00098	0.00045	0.00013	-0.0013
1.3E-05	0.00106	0.00123	3.9E-05	0.00032	-0.0016
1.3E-05	0.00099	0.0013	0.00028	0.0004	-0.0019
1.3E-05	0.00138	0.00121	0.00037	-0.0002	-0.0016
1.3E-05	0.00162	0.00102	0.0005	0.00012	-0.0016
1.3E-05	0.00186	0.00083	0.00041	0.00035	-0.0018
1.3E-05	0.00199	0.0018	3.7E-05	0.00012	-0.0014
1.3E-05	0.00141	0.00114	0.00088	6.8E-05	-0.0014
1.3E-05	0.00148	0.00074	0.00041	4.4E-05	-0.0015
1.3E-05	0.00134	0.00085	0.0004	-0.0003	-0.0015
1.3E-05	0.00101	0.00103	0.00094	-0.0005	-0.0017
1.3E-05	0.00165	0.0007	0.00018	1E-05	-0.0013
1.3E-05	0.00172	0.00091	-0.0003	1E-05	-0.0012
1.3E-05	0.0014	0.00134	0.00029	7.9E-05	-0.0016
1.3E-05	0.00181	0.00058	0.00059	0.00036	-0.0019
1.3E-05	0.0016	0.00138	0.00091	0.00012	-0.0011
1.3E-05	0.00253	0.00082	0.00034	0.00014	-0.0019
1.3E-05	0.00173	0.00049	-0.0007	-0.0004	-0.0019
1.3E-05	0.00139	0.00057	0.00039	3.6E-05	-0.0011
1.3E-05	0.00155	0.00015	0.00077	0.00035	-0.0017
1.3E-05	0.00096	0.00079	0.00082	-0.0002	-0.0018
1.3E-05	0.00103	0.00068	0.00042	9.9E-05	-0.0017
1.3E-05	0.00135	0.00085	0.0002	8.2E-05	-0.002
1.3E-05	0.00212	0.00038	0.00048	-0.0007	-0.001
1.3E-05	0.00137	0.00113	0.00087	-0.0012	-0.001
1.3E-05	0.00148	0.00033	0.00033	-4E-05	-0.0015
1.4E-05	0.00099	0.00045	-0.0005	-0.0001	-0.0012
1.4E-05	0.00209	0.00063	0.00059	3.1E-05	-0.0012
1.4E-05	0.00166	0.00061	-0.0006	0.00025	-0.0016
1.4E-05	0.00163	0.00058	0.00058	8.5E-05	-0.0013
1.4E-05	0.00095	0.00127	0.00045	0.00017	-0.0019
1.4E-05	0.00155	0.00056	8.4E-05	-0.0004	-0.0015
1.4E-05	0.00089	0.00024	0.00053	-0.0008	-0.0014
1.4E-05	0.00099	0.00093	0.00011	-0.0002	-0.0018
1.4E-05	0.00123	-4E-05	0.00052	8.7E-06	-0.0014
1.4E-05	0.00169	0.00112	0.00022	-0.0004	-0.0011
1.4E-05	0.00209	0.00085	0.00015	-3E-05	-0.0012
1.4E-05	0.00156	0.00157	0.00064	-0.0001	-0.0015
1.4E-05	0.0005	0.00098	0.00021	0.00073	-0.0016
1.4E-05	0.00165	0.0008	0.00112	-0.0003	-0.0016
1.4E-05	0.00106	0.0014	0.0012	-0.0006	-0.002
1.4E-05	0.00166	0.00088	0.00048	-0.0004	-0.0013
1.4E-05	0.00115	0.00049	0.00078	0.00046	-0.002
1.4E-05	0.00156	0.00063	0.00147	0.00031	-0.0017
1.4E-05	0.00152	0.001	0.00012	-0.0002	-0.0015
1.4E-05	0.00057	0.00057	0.00081	-2E-05	-0.0009
1.4E-05	0.00083	0.00148	0.00068	0.00015	-0.0019
1.4E-05	0.00174	0.00069	0.001	-0.0002	-0.0014
1.4E-05	0.00131	0.00052	-0.0003	-0.0001	-0.0012
1.4E-05	0.00129	0.00056	0.00063	0.00013	-0.001
1.4E-05	0.00066	0.00078	0.00061	0.00031	-0.0011
1.4E-05	0.00168	0.00117	0.00076	0.00021	-0.0016
1.4E-05	0.00076	0.00096	0.00036	0.00018	-0.0018
1.4E-05	0.00118	0.00133	0.00099	0.00053	-0.0014

1.4E-05	0.00088	0.00101	0.00155	-0.0002	-0.0017
1.4E-05	0.00142	0.00027	0.00074	-0.0002	-0.0017
1.4E-05	0.0013	0.00075	0.00018	-0.0005	-0.0013
1.5E-05	0.00095	0.0016	0.00051	4.8E-05	-0.0021
1.5E-05	0.00095	0.00071	0.00013	7.4E-05	-0.0015
1.5E-05	0.00098	0.00072	-0.0007	3.6E-05	-0.0007
1.5E-05	0.00108	0.00065	0.00067	-0.0001	-0.0017
1.5E-05	0.00185	0.00127	0.00046	0.00075	-0.0026
1.5E-05	0.00128	0.00113	0.00079	-1E-05	-0.0017
1.5E-05	0.00128	0.00104	-0.0003	0.00054	-0.002
1.5E-05	0.00118	0.00077	0.00036	-2E-05	-0.0025
1.5E-05	0.00138	0.001	0.00053	0.00078	-0.0019
1.5E-05	0.00208	0.00122	0.00031	-0.0004	-0.0012
1.5E-05	0.00095	0.00116	0.00023	0.00024	-0.0015
1.5E-05	0.00141	0.00069	0.00061	-0.0003	-0.0023
1.5E-05	0.00108	0.00053	0.00098	0.00054	-0.0018
1.5E-05	0.00098	0.00092	0.00046	0.00015	-0.0017
1.5E-05	0.00146	0.00107	0.00068	0.00024	-0.0013
1.5E-05	0.00163	0.00074	-5E-05	0.00028	-0.0015

Table SI.5: Primary kinetic data for the reaction of 1-benzyl-2-pyrrolidone (**S5**) with CumO• generated by 355 nm LFP in MeCN (**Figure SI.5**).

**t / s**	**ΔA 1-benzyl-2-pyrrolidone (S5)**				
	**0.0771M**	**0.152M**	**0.240M**	**0.381M**	**0.568M**
-1E-06	-8E-05	9.4E-05	-0.0003	-0.0004	0.00028
-1E-06	4.5E-05	4E-05	-0.0007	-0.0002	0.0005
-9E-07	-0.0001	-0.0001	-0.0009	-8E-05	0.0005
-9E-07	0.00047	-0.0006	-0.0009	-0.001	0.00092
-9E-07	0.00021	6.9E-05	-0.0004	-0.0002	0.00095
-8E-07	-0.0007	-0.0005	-0.0009	-4E-05	0.0008
-8E-07	3.6E-05	0.00021	-0.001	-0.0012	0.00052
-8E-07	-0.0004	0.00015	-0.001	-0.0012	0.00062
-7E-07	1.8E-05	-0.0005	2.2E-06	-0.0006	0.00091
-7E-07	-0.0003	0.00011	-0.0013	-0.0006	5.7E-05
-7E-07	-0.0001	-0.0009	-0.0003	0.00028	0.00069
-6E-07	-4E-05	7.9E-05	-0.0005	0.00014	0.00094
-6E-07	-0.0004	-9E-05	-0.0011	-0.0003	0.00064
-6E-07	-0.0003	-0.0003	-0.0007	-0.001	0.00081
-5E-07	-0.0002	-0.0005	-0.0007	-0.0008	0.00102
-5E-07	-0.0004	-5E-05	0.00054	-0.0001	0.00131
-5E-07	-0.0002	-0.0001	6.3E-05	0.00011	0.00134
-4E-07	0.00017	0.00039	0.0004	0.00036	0.00146
-4E-07	-0.0001	-8E-05	-0.0012	8.7E-05	-0.0004
-4E-07	0.00022	-0.0001	0.00016	-0.0005	-0.0002
-4E-07	-1E-04	-0.0003	-0.001	-0.0006	0.00052
-3E-07	-7E-05	0.00022	-0.0005	-0.0004	0.00074
-3E-07	-0.0001	-0.0002	-0.001	-0.0007	0.00051
-3E-07	0.0005	7.1E-05	-0.0008	-0.0011	0.00147
-2E-07	3.2E-05	-9E-05	-0.0002	-0.0008	0.0003
-2E-07	1.6E-05	-0.0001	-0.0004	-1E-04	0.00107
-2E-07	-0.0007	0.00024	-0.001	-0.0001	0.00109
-1E-07	-1E-04	-0.0004	-0.0002	-0.0007	0.00107
-1E-07	0.00018	0.00031	9.7E-05	-0.0007	-0.0003
-6E-08	-0.0005	0.00012	-0.0004	-0.0008	0.00129
-3E-08	6.2E-05	-0.0002	-0.0005	-0.0008	0.00102
-4E-10	-0.0033	-0.0024	-0.005	-0.004	-0.0025
3.2E-08	-0.0128	-0.0155	-0.0212	-0.0166	-0.0162
6.4E-08	0.01248	0.00763	0.00243	-0.0034	-0.0087
9.6E-08	0.01841	0.01836	0.01599	0.01467	0.01318
1.3E-07	0.01786	0.0178	0.01725	0.0156	0.01446
1.6E-07	0.01666	0.01725	0.01535	0.0145	0.01449
1.9E-07	0.01652	0.01595	0.01618	0.01468	0.0138
2.2E-07	0.01624	0.01547	0.01541	0.01454	0.01372
2.6E-07	0.01636	0.016	0.01437	0.01294	0.01271
2.9E-07	0.01613	0.0145	0.01377	0.01251	0.01137
3.2E-07	0.01488	0.01438	0.0138	0.01181	0.01057
3.5E-07	0.01384	0.01456	0.01351	0.01174	0.01084
3.8E-07	0.01378	0.01316	0.01302	0.01098	0.0104
4.2E-07	0.0129	0.01248	0.01194	0.01035	0.0095
4.5E-07	0.01287	0.01258	0.01048	0.00958	0.00931
4.8E-07	0.01292	0.01182	0.01064	0.00978	0.0097
5.1E-07	0.01209	0.01112	0.01043	0.00891	0.00912
5.4E-07	0.01206	0.0105	0.01017	0.00918	0.00824

5.8E-07	0.01142	0.01023	0.00991	0.00829	0.0077
6.1E-07	0.01077	0.00988	0.00938	0.00767	0.00694
6.4E-07	0.01088	0.00965	0.00904	0.00691	0.0074
6.7E-07	0.01049	0.00974	0.00883	0.00726	0.00682
7E-07	0.01011	0.00935	0.00798	0.00665	0.00653
7.4E-07	0.00966	0.00909	0.00747	0.00613	0.00653
7.7E-07	0.00967	0.00905	0.00785	0.00591	0.00624
8E-07	0.00939	0.00813	0.00743	0.00508	0.0061
8.3E-07	0.00949	0.008	0.00612	0.00578	0.00586
8.6E-07	0.00905	0.00777	0.0066	0.00468	0.00518
9E-07	0.0086	0.00825	0.0061	0.00438	0.00521
9.3E-07	0.00866	0.00712	0.00646	0.00434	0.00441
9.6E-07	0.00811	0.00633	0.00689	0.00485	0.00473
9.9E-07	0.00767	0.00662	0.0057	0.00406	0.00515
1E-06	0.00784	0.00536	0.00667	0.00368	0.00371
1.1E-06	0.00725	0.00662	0.00551	0.00407	0.00375
1.1E-06	0.00732	0.00645	0.00534	0.00353	0.00443
1.1E-06	0.00665	0.00541	0.00512	0.00373	0.00381
1.2E-06	0.00655	0.00586	0.00412	0.00383	0.00478
1.2E-06	0.00575	0.00511	0.00454	0.00396	0.00454
1.2E-06	0.00668	0.00541	0.00434	0.0026	0.0041
1.2E-06	0.00586	0.0052	0.00393	0.003	0.00403
1.3E-06	0.00622	0.00528	0.00364	0.003	0.00373
1.3E-06	0.00574	0.00502	0.00468	0.0028	0.00312
1.3E-06	0.00551	0.00464	0.00416	0.00406	0.00326
1.4E-06	0.00551	0.0049	0.00417	0.00243	0.00274
1.4E-06	0.00514	0.00461	0.00404	0.00228	0.00343
1.4E-06	0.00498	0.00545	0.00359	0.00193	0.00349
1.5E-06	0.00457	0.00402	0.00315	0.00281	0.00292
1.5E-06	0.00487	0.00425	0.0027	0.00229	0.00262
1.5E-06	0.00555	0.00405	0.00292	0.00259	0.00201
1.6E-06	0.00472	0.00415	0.00306	0.00214	0.00326
1.6E-06	0.00433	0.00462	0.00285	0.00135	0.0032
1.6E-06	0.0042	0.00484	0.0032	0.00181	0.00307
1.7E-06	0.00409	0.00423	0.00246	0.00208	0.00385
1.7E-06	0.00369	0.00437	0.00262	0.00229	0.00303
1.7E-06	0.00388	0.00311	0.00293	0.00239	0.00292
1.8E-06	0.00398	0.00365	0.00258	0.00209	0.00322
1.8E-06	0.00496	0.00388	0.00367	0.00142	0.00378
1.8E-06	0.00373	0.00286	0.00228	0.00186	0.00323
1.9E-06	0.0034	0.00348	0.00235	0.00122	0.003
1.9E-06	0.00377	0.00305	0.0022	0.00164	0.00242
1.9E-06	0.00431	0.00283	0.00238	0.00152	0.00185
2E-06	0.00328	0.00261	0.00192	0.00095	0.00241
2E-06	0.00362	0.00238	0.00188	0.00176	0.0026
2E-06	0.00337	0.00306	0.00291	0.00095	0.00258
2E-06	0.00377	0.00266	0.00188	0.00057	0.00281
2.1E-06	0.00362	0.00283	0.00218	0.00131	0.00228
2.1E-06	0.00337	0.00223	0.00212	0.00161	0.00248
2.1E-06	0.00299	0.00278	0.00184	0.00141	0.00254
2.2E-06	0.00271	0.00309	0.00195	0.00112	0.0019
2.2E-06	0.00336	0.00242	0.00287	0.00046	0.00235
2.2E-06	0.00269	0.00299	0.00158	0.00088	0.00228
2.3E-06	0.00276	0.00257	0.00141	0.00142	0.00221

2.3E-06	0.00299	0.00229	0.00245	0.00086	0.00252
2.3E-06	0.00227	0.00249	0.00211	0.00133	0.00221
2.4E-06	0.00235	0.00242	0.00085	0.00144	0.00309
2.4E-06	0.0026	0.00223	0.00161	0.00094	0.00255
2.4E-06	0.00245	0.0029	0.00177	0.00101	0.00243
2.5E-06	0.00272	0.00196	0.00187	0.00081	0.00286
2.5E-06	0.00244	0.00238	0.00174	0.00085	0.00242
2.5E-06	0.00197	0.00225	0.00189	0.00122	0.00253
2.6E-06	0.00178	0.00233	0.00079	0.00125	0.00275
2.6E-06	0.00236	0.00197	0.00182	0.00134	0.00226
2.6E-06	0.00197	0.00179	0.00207	0.00078	0.00268
2.7E-06	0.00233	0.00234	0.00058	0.00081	0.00206
2.7E-06	0.00167	0.00165	0.00112	0.00086	0.00266
2.7E-06	0.00195	0.00208	0.00115	0.00138	0.00148
2.8E-06	0.00238	0.00176	0.00108	0.00144	0.00223
2.8E-06	0.00259	0.00185	0.00134	0.00112	0.00266
2.8E-06	0.00211	0.00203	0.00158	0.00056	0.00267
2.8E-06	0.00161	0.00216	8.7E-05	0.00104	0.00208
2.9E-06	0.00189	0.00211	0.00088	0.00048	0.00231
2.9E-06	0.00209	0.00209	0.00064	0.00113	0.00234
2.9E-06	0.00133	0.00292	0.00047	0.00091	0.00202
3E-06	0.00156	0.0019	0.00026	0.00039	0.00249
3E-06	0.0011	0.00141	0.00066	6.9E-05	0.00201
3E-06	0.00127	0.00209	0.00098	0.00051	0.00229
3.1E-06	0.00167	0.00191	0.00086	0.00088	0.0023
3.1E-06	0.00108	0.00151	0.00104	0.0009	0.00299
3.1E-06	0.00177	0.00079	0.00118	0.00134	0.00232
3.2E-06	0.0011	0.001	0.00099	0.00055	0.00251
3.2E-06	0.00091	0.00211	0.00161	0.00118	0.00202
3.2E-06	0.00133	0.00121	0.00083	0.00035	0.00268
3.3E-06	0.00023	0.00103	0.00098	0.0012	0.00177
3.3E-06	0.00164	0.00098	0.00033	0.00109	0.00124
3.3E-06	0.00184	0.00159	0.00096	0.00071	0.00193
3.4E-06	0.0016	0.00169	0.0014	0.00059	0.00138
3.4E-06	0.00091	0.00104	0.0009	0.00071	0.00179
3.4E-06	0.00114	0.00099	0.00127	0.00136	0.00244
3.5E-06	0.00052	0.00096	0.00092	0.00104	0.00264
3.5E-06	0.00135	0.00133	0.0008	0.0004	0.00298
3.5E-06	0.00128	0.00116	0.0009	0.00076	0.00208
3.6E-06	0.00154	0.00102	0.00098	0.00102	0.0018
3.6E-06	0.00186	0.00096	0.00105	0.00113	0.00205
3.6E-06	0.00159	0.00048	0.00118	0.00143	0.00188
3.6E-06	0.00166	0.00107	0.00045	0.0007	0.0019
3.7E-06	0.00182	0.00072	-0.0001	0.00062	0.00188
3.7E-06	0.00142	0.00098	0.00067	0.00059	0.00224
3.7E-06	0.00131	0.00138	0.00075	0.00123	0.00248
3.8E-06	0.00079	0.00127	0.00014	0.00085	0.00178
3.8E-06	0.00154	0.00104	0.00138	0.00076	0.00181
3.8E-06	0.00069	0.0016	0.00105	0.00059	0.00223
3.9E-06	0.0017	0.00062	0.00081	0.00082	0.0019
3.9E-06	0.00145	0.00089	0.00117	0.00047	0.00233
3.9E-06	0.00094	0.00129	0.00054	0.00057	0.00169
4E-06	0.00099	0.00059	0.00132	0.00107	0.00207
4E-06	0.00067	0.00075	0.00057	0.00091	0.00258

4E-06	0.00086	0.00064	0.00074	0.00068	0.00151
4.1E-06	0.00074	0.00096	0.00143	0.0008	0.00132
4.1E-06	0.00181	0.00053	0.00088	0.00077	0.00186
4.1E-06	0.00106	0.0018	0.00069	0.00074	0.00218
4.2E-06	0.00069	0.00104	0.00107	0.00081	0.00191
4.2E-06	0.00112	0.00153	0.00014	0.0008	0.00122
4.2E-06	0.00108	0.0006	0.00115	0.00057	0.00219
4.3E-06	0.00049	0.00077	0.00098	0.00084	0.00161
4.3E-06	0.00093	0.00125	0.00079	0.00099	0.00146
4.3E-06	0.00099	0.00049	0.00099	0.00079	0.00138
4.4E-06	0.00069	0.00132	0.00075	0.00039	0.00146
4.4E-06	0.00034	0.00068	0.00057	0.00095	0.00152
4.4E-06	6.5E-05	0.00141	0.00069	0.00094	0.00205
4.4E-06	0.00091	0.00102	0.0003	0.00053	0.00222
4.5E-06	0.00039	0.00029	0.0011	0.0003	0.00224
4.5E-06	0.00065	0.00113	0.00063	0.00064	0.00246
4.5E-06	0.00041	0.00077	0.00056	0.00035	0.00264
4.6E-06	0.00113	0.00123	0.00036	0.00067	0.00169
4.6E-06	0.00081	0.0013	0.001	0.00107	0.00142
4.6E-06	0.00074	0.00106	0.00118	0.00093	0.00195
4.7E-06	0.00063	0.00134	0.00049	0.00102	0.00172
4.7E-06	0.00032	0.00066	0.00052	0.00094	0.00124
4.7E-06	0.00039	0.00213	0.00084	0.00114	0.00186
4.8E-06	0.00026	0.00098	5.2E-05	0.00023	0.00145
4.8E-06	0.00119	0.0006	0.00035	0.00094	0.00155
4.8E-06	0.00073	0.00068	0.00051	0.00105	0.00227
4.9E-06	0.00061	0.00112	0.00085	0.00067	0.00238
4.9E-06	0.00111	0.00124	0.00078	0.00052	0.00174
4.9E-06	0.001	0.00083	0.00083	0.00052	0.00176
5E-06	0.00045	0.00079	0.0008	0.00091	0.00202
5E-06	-4E-05	0.00035	0.00095	0.00107	0.002
5E-06	0.00039	0.00045	0.00063	0.00033	0.00193
5.1E-06	0.00045	0.00102	0.00086	0.00071	0.00122
5.1E-06	0.00071	0.00082	0.00061	0.00073	0.00155
5.1E-06	0.00025	0.00134	0.00044	0.00042	0.00187
5.2E-06	0.00088	0.00105	0.00046	0.00027	0.00245
5.2E-06	0.00103	0.00027	0.0007	0.00059	0.00189
5.2E-06	6.5E-05	0.00077	0.00068	0.00039	0.00235
5.2E-06	0.00114	0.00067	0.0014	0.00088	0.00231
5.3E-06	0.00071	0.00126	0.00058	0.00137	0.00188
5.3E-06	0.00065	0.0015	0.00031	0.00035	0.00215
5.3E-06	0.00136	0.00115	0.00039	0.00075	0.00259
5.4E-06	0.00067	0.00127	0.0009	0.00082	0.00138
5.4E-06	0.00059	0.00041	0.00099	0.00054	0.00144
5.4E-06	0.00099	0.0004	0.00044	0.00098	0.00173
5.5E-06	0.00018	-0.0002	0.00098	0.00084	0.00244
5.5E-06	0.00127	0.00121	0.00036	0.00033	0.00168
5.5E-06	0.0008	0.00123	0.00207	0.00037	0.00148
5.6E-06	0.00042	0.00173	0.0007	0.00021	0.00195
5.6E-06	7.6E-05	0.00047	0.00015	0.00017	0.00168
5.6E-06	0.00033	0.00116	0.00067	0.0006	0.00259
5.7E-06	0.00018	0.00143	0.00016	0.00074	0.00204
5.7E-06	0.00025	0.00056	0.00095	0.00055	0.00185
5.7E-06	0.00025	0.00061	0.00069	0.0011	0.002

5.8E-06	0.00049	0.0013	0.00075	0.00087	0.00208
5.8E-06	0.00065	0.00076	0.00063	0.00113	0.00187
5.8E-06	2.2E-05	0.00042	0.00037	0.00022	0.00288
5.9E-06	-0.0003	0.00102	0.00094	0.00135	0.00165
5.9E-06	0.00123	0.00094	0.00098	0.0005	0.00144
5.9E-06	6.9E-05	0.00103	0.00081	0.00103	0.00133
6E-06	0.00018	0.00095	0.0007	0.0011	0.00179
6E-06	-0.0004	0.00078	-0.0003	0.00046	0.00154
6E-06	0.00045	0.00065	0.00015	0.0007	0.00178
6E-06	0.00063	0.00082	0.00092	0.00035	0.00229
6.1E-06	-0.0002	0.0011	0.00117	0.00042	0.00163
6.1E-06	0.00117	0.00098	0.00099	0.00059	0.00223
6.1E-06	0.00071	0.00103	0.00061	-0.0004	0.00256
6.2E-06	-0.0005	0.00082	4.9E-05	0.00023	0.00102
6.2E-06	0.00016	0.0008	0.00015	0.00052	0.00156
6.2E-06	-0.0003	0.00025	0.00067	0.00042	0.00192
6.3E-06	-0.0003	0.00026	0.00058	7.4E-05	0.00162
6.3E-06	0.00025	0.00129	3.8E-05	0.00022	0.00222
6.3E-06	0.00019	0.00133	0.00052	0.00032	0.0015
6.4E-06	-0.0004	0.00114	0.00088	0.00038	0.00171
6.4E-06	1.9E-05	0.00066	0.00041	-0.0002	0.00259
6.4E-06	0.0003	0.0006	0.00116	0.00026	0.00228
6.5E-06	0.0007	0.00115	0.00097	0.00097	0.00152
6.5E-06	0.00036	0.00036	0.00074	0.00037	0.00226
6.5E-06	-0.0004	0.00138	5.4E-05	-7E-05	0.00196
6.6E-06	0.00085	0.0003	0.00013	6.6E-05	0.00173
6.6E-06	0.00025	0.00187	0.00049	-0.0009	0.00132
6.6E-06	0.00086	0.00043	0.0008	0.00059	0.00164
6.7E-06	0.00063	0.00052	0.00115	0.00106	0.00146
6.7E-06	0.00036	0.00121	0.00049	1E-05	0.0025
6.7E-06	0.00015	0.00096	0.00139	-0.0005	0.0014
6.8E-06	0.00043	0.00025	-2E-05	0.00092	0.00101
6.8E-06	0.00031	0.00095	0.00095	-7E-05	0.00179
6.8E-06	0.00038	0.00031	0.00057	0.00067	0.00109
6.8E-06	0.00047	0.00079	0.00095	0.00045	0.00126
6.9E-06	0.00017	0.00104	0.00068	0.00089	0.00182
6.9E-06	7.5E-05	0.0021	0.00039	0.00097	0.00227
6.9E-06	-0.0003	0.00119	0.00033	0.00027	0.00175
7E-06	0.00069	0.00107	0.00046	-0.0002	0.0013
7E-06	0.00046	0.0016	0.0009	0.00044	0.00199
7E-06	0.00077	0.00107	-0.0003	0.0007	0.00168
7.1E-06	0.00109	0.00087	-0.0002	0.0011	0.00207
7.1E-06	-1E-04	0.00068	1E-05	0.00115	0.00161
7.1E-06	0.00057	1.7E-05	-0.0002	0.00026	0.00216
7.2E-06	0.00023	0.0008	0.0006	0.00046	0.00082
7.2E-06	-0.0004	0.00076	0.00081	0.00063	0.00187
7.2E-06	0.00082	0.00048	0.00057	0.00134	0.00201
7.3E-06	0.00035	0.00097	0.00072	0.00044	0.00203
7.3E-06	6.9E-05	0.00153	0.00033	0.00047	0.00231
7.3E-06	-2E-06	0.00113	0.00074	0.00039	0.00169
7.4E-06	-0.0001	0.00037	-7E-05	0.00034	0.0013
7.4E-06	0.0007	-4E-06	0.00079	0.00081	0.00154
7.4E-06	0.00017	0.00019	0.00069	0.00076	0.00165
7.5E-06	3.1E-05	0.00103	0.00102	-0.0002	0.00156

7.5E-06	-0.0001	0.00154	0.00064	0.00061	0.00197
7.5E-06	0.00034	0.00068	0.0011	-0.0001	0.00135
7.6E-06	0.00049	0.00122	0.00088	0.00041	0.00138
7.6E-06	0.00041	0.00133	0.00062	0.00079	0.00152
7.6E-06	2.6E-05	-0.0003	0.00112	-3E-05	0.00138
7.6E-06	-0.0003	0.00151	0.00029	0.00028	0.0023
7.7E-06	0.00034	0.00162	0.00048	0.00012	0.0012
7.7E-06	0.00045	0.00113	0.00107	0.00054	0.00201
7.7E-06	-0.0006	0.00108	0.00107	0.00017	0.00158
7.8E-06	0.00026	0.00056	-3E-05	-0.0003	0.00197
7.8E-06	-0.0006	0.00098	0.00126	0.00044	0.0016
7.8E-06	0.00017	0.00063	0.00053	0.00013	0.00191
7.9E-06	-0.0004	-0.0002	0.00071	0.0006	0.00172
7.9E-06	0.00031	0.00103	0.00102	0.00102	0.00151
7.9E-06	-0.0004	0.00024	0.00093	-0.0002	0.00178
8E-06	-0.0007	0.00089	0.0008	0.00021	0.00245
8E-06	-8E-05	0.00065	0.00092	-0.0002	0.00154
8E-06	0.00044	-0.0004	3.4E-05	0.00075	0.00244
8.1E-06	-0.0002	0.00034	-0.0004	0.00067	0.00242
8.1E-06	-0.0002	0.00068	0.00048	0.00012	0.00123
8.1E-06	0.00056	0.00148	0.00073	0.00035	0.00234
8.2E-06	0.00116	0.00147	0.00094	-9E-05	0.00119
8.2E-06	0.00089	-0.0003	0.00139	0.00038	0.00189
8.2E-06	0.00062	0.00037	0.00123	0.00081	0.00206
8.3E-06	-0.0008	0.00034	0.00097	0.00029	0.00158
8.3E-06	0.00062	0.00104	0.00051	0.00122	0.00258
8.3E-06	0.00031	0.00108	0.0004	0.00046	0.00204
8.4E-06	-0.0002	0.00114	0.00095	0.00053	0.00168
8.4E-06	-0.0003	0.00128	0.00013	0.00056	0.00198
8.4E-06	0.00021	0.00076	0.00078	-0.0004	0.0012
8.4E-06	-2E-06	-0.0002	0.00108	-4E-05	0.00173
8.5E-06	-0.0003	0.00048	1.3E-05	-3E-05	0.00154
8.5E-06	0.00023	0.00106	0.00063	3.8E-05	0.0014
8.5E-06	0.00019	0.00091	0.0004	-7E-05	0.00185
8.6E-06	-0.0005	0.00099	0.00053	0.00041	0.00117
8.6E-06	0.00013	0.00145	0.00128	0.00017	0.00192
8.6E-06	0.0002	0.00081	0.00053	0.00045	0.00151
8.7E-06	0.00063	0.00113	0.00098	-0.0006	0.00207
8.7E-06	0.00018	0.00014	0.00026	0.00044	0.0018
8.7E-06	0.00022	0.00055	0.00039	0.00051	0.0014
8.8E-06	0.00017	0.00127	-0.0002	-0.0002	0.00189
8.8E-06	-6E-05	0.00096	0.00038	0.00011	0.00141
8.8E-06	-0.0002	0.00045	0.00021	-0.0003	0.00142
8.9E-06	0.00046	-0.0003	0.00054	-0.0002	0.00182
8.9E-06	-0.0002	0.00031	0.00063	-0.0002	0.00201
8.9E-06	0.0008	0.00171	0.00049	0.00027	0.00218
9E-06	0.00087	0.00076	0.00039	0.00012	0.00209
9E-06	0.00015	-3E-05	0.00011	0.00035	0.00162
9E-06	0.00036	0.0009	0.00029	4.1E-05	0.00147
9.1E-06	0.00041	0.00081	-0.0001	0.00057	0.00097
9.1E-06	0.00031	0.00032	0.00095	0.00015	0.00088
9.1E-06	0.00035	0.00051	0.00024	-0.0003	0.00164
9.2E-06	0.00037	0.00148	0.00075	0.00023	0.00157
9.2E-06	0.00086	0.00073	0.00042	0.00109	0.0021

9.2E-06	0.00011	0.00051	0.00048	0.00053	0.00145
9.2E-06	0.0002	0.001	0.0006	2.1E-05	0.00172
9.3E-06	-0.0003	0.00052	0.00117	-2E-06	0.00191
9.3E-06	9E-05	0.00034	0.00064	0.00055	0.00176
9.3E-06	0.00078	0.00076	0.00044	-8E-05	0.00156
9.4E-06	3E-05	0.00071	0.00134	1.1E-05	0.00212
9.4E-06	0.00068	-0.0004	0.00042	0.00053	0.00163
9.4E-06	-0.0003	0.00022	0.00073	-0.0005	0.00171
9.5E-06	-0.0003	0.00144	0.00066	0.0009	0.00243
9.5E-06	-0.0003	0.00013	0.00102	0.00025	0.00239
9.5E-06	0.00089	0.00097	-0.0002	-0.0002	0.00148
9.6E-06	-5E-05	0.00083	0.00065	-0.0002	0.00183
9.6E-06	2.5E-05	0.00072	0.00023	-0.0001	0.00171
9.6E-06	3E-05	0.00126	0.00062	0.00032	0.00152
9.7E-06	0.00049	0.00049	0.00035	-0.0003	0.00184
9.7E-06	0.00042	0.00128	-5E-06	0.00039	0.00226
9.7E-06	0.00032	0.00083	-0.0003	0.00114	0.00265
9.8E-06	-0.0001	5.5E-05	0.00065	0.00073	0.00147
9.8E-06	-0.0002	0.00078	0.00079	0.00058	0.00186
9.8E-06	-0.0001	0.00106	0.00141	0.00076	0.00125
9.9E-06	0.00054	-0.0004	5.7E-05	0.00025	0.00181
9.9E-06	0.00024	0.00032	0.00067	0.00099	0.00112
9.9E-06	0.00031	0.00112	-0.0002	-0.0002	0.00194
1E-05	-0.0006	0.00052	0.00053	0.00035	0.00097
1E-05	0.00039	0.00054	0.0009	0.0005	0.00197
1E-05	0.0008	0.00147	0.00108	0.00028	0.00162
1E-05	-7E-05	0.00109	0.0004	4.2E-05	0.00165
1E-05	0.00041	0.00081	0.00093	0.00012	0.00123
1E-05	0.00091	0.00123	-0.0001	0.00078	0.00253
1E-05	0.00066	0.00063	0.00013	0.00077	0.00123
1E-05	0.00027	0.00011	0.00023	0.00086	0.00178
1E-05	-0.0002	0.00088	0.00033	1.7E-05	0.00155
1E-05	-0.0002	0.00064	0.00096	-0.0004	0.00124
1E-05	8.5E-05	7.1E-05	0.00062	-0.0003	0.00216
1E-05	-0.0002	0.0003	-3E-05	0.00043	0.00132
1E-05	0.00051	0.00061	0.0009	-9E-05	0.00138
1E-05	0.0003	3.3E-05	0.00035	0.00051	0.00169
1E-05	0.00026	0.00069	-6E-05	-8E-05	0.00213
1E-05	-0.0005	0.00029	0.00081	0.00036	0.00177
1E-05	0.00024	-8E-05	0.00061	0.001	0.00105
1.1E-05	-0.0001	0.00034	0.00037	-0.0005	0.00139
1.1E-05	0.00036	0.001	0.00042	0.00096	0.00151
1.1E-05	0.00026	0.00038	0.00105	0.00036	0.00129
1.1E-05	0.00047	0.00074	-0.0002	0.00031	0.00152
1.1E-05	0.00038	0.00032	0.0005	-9E-05	0.001
1.1E-05	-0.0002	0.00037	0.00093	-0.0004	0.00228
1.1E-05	5.6E-05	0.00135	0.001	0.00044	0.00128
1.1E-05	1.3E-05	0.00086	0.00033	-5E-05	0.00143
1.1E-05	0.00074	0.00079	0.00055	5.4E-05	0.00115
1.1E-05	0.00032	0.00096	0.00024	0.00064	0.00207
1.1E-05	7E-05	0.00175	-0.0001	1.6E-05	0.00179
1.1E-05	0.00061	0.00097	0.00029	0.00011	0.00152
1.1E-05	0.00078	0.0012	0.00033	-0.0004	0.00181
1.1E-05	0.00018	0.00086	0.00108	-0.0002	0.00155

1.1E-05	7.1E-05	0.001	0.00045	0.00042	0.00126
1.1E-05	0.00026	0.0012	0.0004	0.00033	0.00209
1.1E-05	0.00017	0.00114	0.00032	0.00021	0.00154
1.1E-05	-0.0003	0.00127	0.00059	3.9E-05	0.0026
1.1E-05	-0.0001	0.00017	0.00043	0.0002	0.00217
1.1E-05	-0.0005	-0.0005	0.00033	0.00018	0.00171
1.1E-05	-0.0005	0.00051	0.00042	-0.0004	0.00177
1.1E-05	0.00043	0.00046	0.00012	-1E-04	0.00236
1.1E-05	-0.0003	-0.0003	1.7E-05	-0.0001	0.00178
1.1E-05	-0.0004	-0.0002	5E-05	0.0007	0.00231
1.1E-05	0.00077	0.00047	9.5E-05	6.9E-05	0.00173
1.1E-05	0.00024	0.00091	0.00024	0.00064	0.0019
1.1E-05	1.5E-05	0.0013	0.00112	0.00041	0.00126
1.1E-05	0.0003	0.00069	6.2E-05	-7E-05	0.00247
1.1E-05	0.0003	0.00022	-0.0002	0.00051	0.00087
1.1E-05	-5E-05	0.00062	0.00049	3.6E-05	0.002
1.1E-05	-0.0006	0.0003	0.0003	0.00017	0.00199
1.1E-05	0.00037	0.00046	0.0003	1.3E-05	0.0022
1.2E-05	0.00065	7.6E-05	0.00083	0.00012	0.00241
1.2E-05	0.00037	0.00014	-0.0006	-0.0003	0.00229
1.2E-05	0.0002	0.00054	0.00053	0.00046	0.00236
1.2E-05	-7E-05	-0.0001	0.00082	0.00066	0.00205
1.2E-05	0.00111	0.00087	0.00064	0.00053	0.00192
1.2E-05	0.00026	-0.0003	0.00077	0.00047	0.00224
1.2E-05	0.00021	0.00114	0.00041	-2E-06	0.0021
1.2E-05	-0.0005	0.00025	0.0003	-2E-05	0.00097
1.2E-05	0.00011	0.0003	0.00076	0.00062	0.00224
1.2E-05	-0.0003	0.00089	0.00083	0.00021	0.00192
1.2E-05	0.00039	0.00019	0.00018	-8E-05	0.00097
1.2E-05	-1E-05	0.0004	0.00052	-5E-05	0.00179
1.2E-05	-0.0002	-0.0002	8.4E-05	-0.0003	0.00241
1.2E-05	-0.0002	-6E-05	0.00103	7.5E-05	0.00164
1.2E-05	0.0007	0.00045	7.5E-06	-0.0008	0.00168
1.2E-05	0.00035	4.9E-05	4.2E-05	0.00044	0.00145
1.2E-05	0.00017	0.00065	0.0005	8.8E-07	0.00192
1.2E-05	-0.0005	0.00103	0.00046	0.00053	0.00189
1.2E-05	0.00049	0.00087	0.00056	0.00044	0.00166
1.2E-05	-0.0001	0.0008	0.00103	-0.0002	0.00159
1.2E-05	0.0008	0.00048	0.00015	0.00025	0.0012
1.2E-05	0.00114	0.00106	0.00028	0.0006	0.00171
1.2E-05	8.7E-05	0.00138	0.00077	2.8E-06	0.00214
1.2E-05	2.7E-05	0.00077	0.00043	0.00043	0.00185
1.2E-05	-0.0005	0.00081	0.00023	0.0004	0.00189
1.2E-05	0.00122	0.00108	-1E-05	0.00016	0.0019
1.2E-05	0.00022	0.00054	0.00016	0.0004	0.00125
1.2E-05	-0.0003	0.00122	-0.0002	-0.0001	0.0012
1.2E-05	0.00013	0.00098	-1E-05	0.00033	0.00175
1.2E-05	0.00056	0.00124	0.00054	0.00095	0.00131
1.2E-05	0.00022	0.00094	0.00027	0.00043	0.0018
1.3E-05	0.00109	0.00082	0.0002	0.00014	0.00125
1.3E-05	0.00082	0.00091	0.0007	-0.0003	0.0014
1.3E-05	0.00024	-0.0001	0.00083	-0.0001	0.00259
1.3E-05	-0.0001	0.00052	0.00016	-4E-05	0.0026
1.3E-05	0.00068	0.00072	0.00048	0.00025	0.00168

1.3E-05	-5E-05	0.00193	0.00082	0.00027	0.0018
1.3E-05	0.00062	0.0004	-0.0003	0.00024	0.00165
1.3E-05	0.00083	0.00045	0.0003	-0.0003	0.00126
1.3E-05	0.00044	0.00042	0.00015	0.00043	0.00237
1.3E-05	-0.0001	0.00049	0.00012	-0.0005	0.00169
1.3E-05	-0.0002	0.00041	3.8E-05	9E-05	0.00189
1.3E-05	0.00096	0.0002	0.00073	0.00065	0.00098
1.3E-05	0.00098	0.00089	0.00061	-0.0001	0.00225
1.3E-05	1.5E-05	0.00163	0.00013	9.1E-05	0.00137
1.3E-05	0.00046	0.00072	0.00034	0.00026	0.00197
1.3E-05	0.00137	0.00011	0.00058	0.00082	0.00159
1.3E-05	-0.0001	0.00049	0.0006	0.00035	0.00203
1.3E-05	0.00028	8.5E-05	-4E-05	3.1E-05	0.00207
1.3E-05	0.00014	0.00118	0.0004	0.00052	0.00197
1.3E-05	6.4E-05	0.00033	0.00082	0.00018	0.00213
1.3E-05	0.00017	0.00018	0.00021	0.00027	0.00196
1.3E-05	-0.0007	0.00112	0.00023	0.00023	0.00191
1.3E-05	0.0005	0.00136	-4E-05	0.00011	0.00192
1.3E-05	0.00034	0.0012	0.00071	0.00049	0.00166
1.3E-05	0.00025	0.00166	0.00023	-0.0002	0.00132
1.3E-05	0.00022	0.00079	0.00058	0.00077	0.00214
1.3E-05	0.00031	-6E-05	0.00106	0.00042	0.00198
1.3E-05	0.00085	0.00054	-0.0005	-0.0009	0.00197
1.3E-05	0.00032	0.00024	0.00063	-0.0004	0.00169
1.3E-05	3.9E-05	0.00035	0.00076	3.1E-05	0.00173
1.3E-05	0.00019	0.00026	0.00036	-0.0002	0.00167
1.4E-05	0.00027	0.00098	0.00036	-0.0009	0.00192
1.4E-05	-1E-04	0.00066	0.00026	-0.0002	0.0023
1.4E-05	-0.0005	0.00035	0.00052	0.00033	0.00113
1.4E-05	0.00069	0.00081	0.00032	1.7E-05	0.00109
1.4E-05	0.00084	9.7E-06	0.00051	-5E-05	0.00206
1.4E-05	0.00023	0.0008	0.00067	-0.0002	0.00159
1.4E-05	0.00086	0.00036	0.00014	0.00029	0.00198
1.4E-05	-4E-05	-0.0004	-0.0004	-0.0002	0.00117
1.4E-05	0.00033	-0.0004	9.6E-05	-1E-04	0.00234
1.4E-05	-0.0001	-0.0004	5.7E-05	0.00048	0.00261
1.4E-05	0.00015	0.00083	9.3E-05	-2E-06	0.00188
1.4E-05	0.00036	0.00012	0.00027	0.00019	0.00198
1.4E-05	0.00051	-0.0004	-8E-05	0.00023	0.00174
1.4E-05	0.00028	-5E-05	-4E-05	0.0006	0.00155
1.4E-05	0.00043	0.0008	0.00021	0.00017	0.00226
1.4E-05	-2E-05	0.00036	0.00075	0.00013	0.00113
1.4E-05	4.7E-05	0.00074	3.4E-05	-0.0002	0.00205
1.4E-05	0.00091	0.00048	0.00068	0.00013	0.00202
1.4E-05	0.00073	0.00093	0.00023	0.00076	0.00159
1.4E-05	0.00024	0.00116	0.00071	-5E-05	0.0011
1.4E-05	0.00011	0.00113	0.00063	0.00017	0.00181
1.4E-05	0.00047	0.00019	0.00066	9.3E-05	0.00163
1.4E-05	0.00032	1.2E-06	0.00051	0.0002	0.00129
1.4E-05	0.00031	0.00091	-9E-05	-0.0008	0.00148
1.4E-05	0.00074	0.00079	0.00013	3.9E-06	0.00224
1.4E-05	0.00073	0.00042	0.00036	0.00105	0.00268
1.4E-05	-0.0006	0.00109	0.00101	-0.0005	0.00191
1.4E-05	0.00062	0.00063	0.00029	-0.0007	0.00216

1.4E-05	0.00065	0.00059	0.00035	-0.0002	0.00163
1.4E-05	6.1E-05	0.00063	1.2E-05	-0.0003	0.00209
1.4E-05	9.1E-05	0.0007	-9E-07	-0.0004	0.00236
1.5E-05	-0.0001	0.00018	0.00074	-0.0005	0.0019
1.5E-05	0.00057	0.00034	0.00018	-0.0004	0.00204
1.5E-05	0.00093	0.00097	1.1E-05	0.00014	0.00254
1.5E-05	0.00044	-7E-05	-0.0001	-0.0003	0.00176
1.5E-05	0.00069	0.00071	0.00133	-0.0002	0.00184
1.5E-05	0.00097	0.00013	0.00029	-0.0007	0.00167
1.5E-05	0.00044	0.00029	0.0006	-0.0004	0.00184
1.5E-05	0.00029	0.00045	0.00079	-0.0004	0.00191
1.5E-05	0.00026	0.00058	0.00042	5.5E-05	0.002
1.5E-05	0.00014	0.00051	2.1E-05	-4E-05	0.00138
1.5E-05	0.00062	5.2E-05	0.00081	0.00031	0.00177
1.5E-05	0.00076	-2E-05	0.0005	-0.0001	0.00123
1.5E-05	0.0005	0.00022	0.00019	0.00014	0.0015
1.5E-05	-0.0001	0.00068	0.00061	0.00013	0.00166
1.5E-05	0.00068	0.0009	0.0002	0.00011	0.00247
1.5E-05	0.00033	0.00016	-0.0003	-0.0002	0.00174

Table SI.6: Primary kinetic data for the reaction of 1-cyclohexyl-2-pyrrolidone (**S6**) with CumO$^{\bullet}$ generated by 355 nm LFP in MeCN (**Figure SI.6**).

t / s	ΔA 1-cyclohexyl-2-pyrrolidone (S6)				
	**0.0610M**	**0.136M**	**0.223M**	**0.321M**	**0.430M**
-1E-06	0.00052	-0.0002	-0.0006	0.00084	0.00012
-1E-06	0.0003	3.7E-06	-0.0008	0.00142	-0.0011
-9E-07	-0.0006	-7E-05	-0.0008	0.00118	-0.0008
-9E-07	-0.0002	0.00036	-0.001	0.00153	-0.0003
-9E-07	0.00075	7.9E-05	-0.0009	0.00101	0.00015
-8E-07	-0.0002	0.00016	-0.0008	0.00189	-0.0005
-8E-07	9.3E-05	-0.0002	-0.001	0.00172	-0.0003
-8E-07	-0.0006	-0.0002	-0.0008	0.00059	-0.0004
-7E-07	-0.0006	7.6E-05	-0.0009	0.00161	-0.001
-7E-07	0.00028	-0.0001	0.00015	0.00104	-0.0002
-7E-07	-0.0002	3.8E-05	-0.0005	0.00126	-0.0005
-6E-07	0.00019	0.00023	-0.0007	0.00076	-8E-05
-6E-07	1.2E-05	6E-05	-0.0003	0.00125	-0.0002
-6E-07	-0.0006	0.00035	-0.0008	0.00175	-0.0004
-5E-07	-0.0007	0.00014	-0.0001	0.00112	-0.0005
-5E-07	-0.0005	0.00017	-0.0007	0.00132	-0.0006
-5E-07	-0.0005	0.00033	-0.0003	0.00056	0.00018
-4E-07	-1E-05	-0.0004	-0.0006	0.00093	-0.0005
-4E-07	0.0003	0.00018	-0.0005	0.00109	-0.0008
-4E-07	0.00028	-0.0003	-0.0006	0.00114	-0.0001
-4E-07	7.4E-05	0.00013	-0.0005	0.00062	-0.0002
-3E-07	0.00039	-0.0005	-0.0011	0.00126	0.00072
-3E-07	-7E-05	0.00024	-0.0007	0.00149	0.00034
-3E-07	-0.0003	0.00026	-8E-05	0.00101	-0.0007
-2E-07	-6E-07	-0.0002	-0.0004	0.00126	0.0003
-2E-07	0.00038	-0.0001	-0.0007	0.00123	0.00014
-2E-07	0.00021	-3E-05	-0.0004	0.00093	0.00014
-1E-07	0.00034	0.00029	-0.0003	0.00145	6.6E-05
-1E-07	-0.0006	1.8E-05	0.00041	0.00088	-0.0005
-6E-08	0.00022	-0.0002	-0.0009	0.00116	4.3E-05
-3E-08	-0.0004	0.00032	-0.0002	0.00083	-0.0005
-4E-10	-0.0035	-0.0029	-0.0036	-0.0029	-0.0038
3.2E-08	-0.0196	-0.0169	-0.0183	-0.0184	-0.0183
6.4E-08	0.00931	0.00269	-0.0032	-0.0065	-0.0117
9.6E-08	0.0163	0.01452	0.01379	0.01391	0.01077
1.3E-07	0.01621	0.01473	0.01497	0.01593	0.01309
1.6E-07	0.0149	0.01474	0.01411	0.01486	0.01228
1.9E-07	0.015	0.01427	0.01399	0.01406	0.01233
2.2E-07	0.01432	0.01376	0.01314	0.01405	0.01103
2.6E-07	0.01421	0.01289	0.01302	0.01233	0.01036
2.9E-07	0.01291	0.01276	0.01108	0.012	0.00968
3.2E-07	0.01274	0.01184	0.0113	0.01213	0.00886
3.5E-07	0.01265	0.01132	0.01084	0.01127	0.00879
3.8E-07	0.01281	0.01092	0.00899	0.01112	0.00835
4.2E-07	0.01262	0.01093	0.0095	0.01042	0.00741
4.5E-07	0.01137	0.00953	0.00888	0.00931	0.00786
4.8E-07	0.01124	0.00998	0.00848	0.00853	0.00645
5.1E-07	0.01071	0.00969	0.00822	0.0085	0.00593
5.4E-07	0.01022	0.00931	0.00771	0.00874	0.00664

5.8E-07	0.00933	0.00863	0.00652	0.00835	0.00552
6.1E-07	0.0105	0.00836	0.007	0.00853	0.005
6.4E-07	0.01	0.00796	0.00717	0.00749	0.00461
6.7E-07	0.00974	0.00694	0.00638	0.00723	0.00437
7E-07	0.00861	0.00688	0.00541	0.00647	0.00482
7.4E-07	0.00872	0.00779	0.00625	0.00588	0.00334
7.7E-07	0.0082	0.0067	0.00561	0.0054	0.00354
8E-07	0.00811	0.00622	0.00528	0.00584	0.00347
8.3E-07	0.00874	0.00622	0.00497	0.00578	0.00329
8.6E-07	0.00771	0.00632	0.00415	0.00546	0.00224
9E-07	0.00706	0.00639	0.00517	0.00562	0.00246
9.3E-07	0.00838	0.00595	0.00457	0.00553	0.0034
9.6E-07	0.00635	0.00554	0.00378	0.00435	0.00222
9.9E-07	0.00634	0.00534	0.00359	0.00405	0.00256
1E-06	0.00719	0.00443	0.00378	0.00473	0.00158
1.1E-06	0.00665	0.00492	0.0036	0.00436	0.00177
1.1E-06	0.00655	0.00466	0.00362	0.00406	0.00257
1.1E-06	0.0065	0.00431	0.00288	0.00394	0.00178
1.2E-06	0.00649	0.00515	0.00338	0.00391	0.00119
1.2E-06	0.00538	0.00509	0.00339	0.00325	0.00193
1.2E-06	0.00536	0.00411	0.00379	0.00362	0.00225
1.2E-06	0.00563	0.00431	0.0033	0.00408	0.00083
1.3E-06	0.00609	0.00399	0.00299	0.0028	0.00159
1.3E-06	0.00605	0.00384	0.00242	0.00283	0.00112
1.3E-06	0.00524	0.00344	0.00245	0.003	0.0009
1.4E-06	0.00545	0.00306	0.00211	0.00332	0.00188
1.4E-06	0.00509	0.00309	0.0025	0.00342	0.00126
1.4E-06	0.00503	0.00329	0.00217	0.00285	0.0014
1.5E-06	0.00492	0.00329	0.00233	0.00373	0.00114
1.5E-06	0.00453	0.00332	0.00229	0.00277	0.00114
1.5E-06	0.00464	0.00375	0.00185	0.00284	0.00117
1.6E-06	0.00367	0.00302	0.00128	0.00294	0.00043
1.6E-06	0.00414	0.0031	0.00171	0.00332	0.00156
1.6E-06	0.00349	0.00343	0.00176	0.00312	0.00153
1.7E-06	0.00418	0.00296	0.00156	0.00283	0.0008
1.7E-06	0.00387	0.00252	0.00113	0.00312	0.00166
1.7E-06	0.00375	0.00228	0.00104	0.00279	0.00164
1.8E-06	0.00348	0.0017	0.00155	0.00284	0.00163
1.8E-06	0.0035	0.00148	0.00082	0.00273	0.00128
1.8E-06	0.00336	0.00205	0.00113	0.00284	0.0016
1.9E-06	0.00314	0.002	0.00126	0.00235	0.0005
1.9E-06	0.00404	0.00251	0.00154	0.0023	0.00066
1.9E-06	0.00296	0.00224	0.00093	0.00221	0.00043
2E-06	0.00262	0.00163	0.0008	0.00207	-0.0002
2E-06	0.00308	0.00139	0.00146	0.00172	0.00136
2E-06	0.00296	0.0015	0.00113	0.00256	0.00173
2E-06	0.00282	0.00144	0.00094	0.00215	0.00136
2.1E-06	0.00306	0.00097	0.00088	0.00222	0.00121
2.1E-06	0.00311	0.00121	0.00056	0.00183	0.00031
2.1E-06	0.00317	0.00171	0.00143	0.00242	0.00078
2.2E-06	0.00192	0.00091	0.00115	0.0018	0.0007
2.2E-06	0.00248	0.00118	0.00068	0.00249	0.00085
2.2E-06	0.00257	0.0015	0.00072	0.00165	0.00038
2.3E-06	0.00253	0.00092	0.00104	0.00182	0.00026

2.3E-06	0.00208	0.00154	0.00136	0.00278	0.00065
2.3E-06	0.00259	0.00135	0.00055	0.00262	0.00095
2.4E-06	0.00147	0.00154	0.00017	0.002	0.00031
2.4E-06	0.0018	0.00056	0.0012	0.00216	0.00023
2.4E-06	0.0025	0.00099	0.00025	0.00149	0.00092
2.5E-06	0.00234	0.00118	0.00058	0.00179	0.00038
2.5E-06	0.00199	0.00105	0.00111	0.00265	0.00118
2.5E-06	0.00089	0.00118	-0.0005	0.00237	0.00079
2.6E-06	0.00165	0.00094	0.00024	0.00184	0.00081
2.6E-06	0.00152	0.00203	0.00121	0.00217	0.00098
2.6E-06	0.0018	0.00169	0.00092	0.00158	0.0009
2.7E-06	0.00152	0.00127	-0.0002	0.00193	0.00054
2.7E-06	0.00132	0.00184	0.00028	0.0017	-3E-05
2.7E-06	0.00124	0.0011	0.00066	0.00176	0.00038
2.8E-06	0.00114	0.00085	0.00069	0.00107	0.00093
2.8E-06	0.00153	0.00125	0.00065	0.00315	0.00106
2.8E-06	0.00246	0.00094	0.00056	0.00255	0.0007
2.8E-06	0.00154	0.00053	0.00089	0.00259	0.00098
2.9E-06	0.00177	0.00107	-0.0005	0.00221	0.00055
2.9E-06	0.00161	0.0013	0.0007	0.00252	0.00074
2.9E-06	0.00207	0.00111	0.00024	0.00228	0.00053
3E-06	0.00164	0.00045	0.00071	0.00293	0.00061
3E-06	0.00198	0.00116	0.00043	0.00194	0.00021
3E-06	0.00083	0.00049	0.00058	0.00194	0.00028
3.1E-06	0.0006	0.00097	0.00081	0.00165	0.00014
3.1E-06	0.00114	0.00026	0.00052	0.00263	-0.0003
3.1E-06	0.00126	0.00144	0.00038	0.0028	0.00046
3.2E-06	0.00104	0.00055	0.00037	0.00248	0.00062
3.2E-06	0.00151	0.00113	0.00074	0.00148	0.00079
3.2E-06	0.00092	0.0006	0.00028	0.0025	0.00096
3.3E-06	0.0008	0.0006	-1E-05	0.00193	0.00086
3.3E-06	0.00073	-7E-06	-0.0005	0.002	0.00104
3.3E-06	0.00045	0.00032	-0.0003	0.00289	0.00118
3.4E-06	0.00144	0.00018	0.00026	0.00273	0.00078
3.4E-06	0.00074	0.00085	0.00087	0.00232	0.00161
3.4E-06	0.00126	0.00083	0.0004	0.0016	0.00014
3.5E-06	0.00056	0.00049	-1E-05	0.00229	0.00068
3.5E-06	0.00125	0.00014	0.00038	0.00141	0.00141
3.5E-06	0.00159	0.00094	0.00066	0.00157	0.0011
3.6E-06	0.00054	0.00011	0.00042	0.00184	0.0007
3.6E-06	0.00032	0.00101	0.0005	0.00147	0.00012
3.6E-06	0.00091	0.00115	0.00098	0.0026	0.0012
3.6E-06	0.00133	0.00047	0.00118	0.00255	0.00088
3.7E-06	0.00037	0.00016	0.00065	0.00195	0.00129
3.7E-06	0.00084	0.00061	0.00077	0.00165	0.00074
3.7E-06	0.00134	0.00093	-0.0002	0.00093	0.0005
3.8E-06	0.00077	0.00095	0.00041	0.00183	0.00016
3.8E-06	0.00089	0.00082	-0.0001	0.00248	0.00055
3.8E-06	0.00139	0.00061	-7E-05	0.0017	0.00064
3.9E-06	0.00196	5.1E-06	9.3E-05	0.00285	7.8E-05
3.9E-06	0.00068	-3E-05	0.00072	0.00236	0.00053
3.9E-06	0.00061	0.00083	5.6E-05	0.00256	0.00066
4E-06	0.0015	0.00048	0.0008	0.00181	0.00075
4E-06	0.00035	0.00027	0.0007	0.00209	0.00051

4E-06	-8E-05	-0.0003	0.00076	0.00118	0.00061
4.1E-06	0.00036	0.00026	0.00014	0.00314	0.00015
4.1E-06	0.00064	0.00079	0.00033	0.0028	0.00083
4.1E-06	0.00056	0.00088	-0.0013	0.00158	0.00028
4.2E-06	0.00065	0.00016	0.00011	0.00173	0.00103
4.2E-06	0.00074	0.00014	7E-05	0.00175	0.00066
4.2E-06	0.00064	0.0002	0.00091	0.00186	0.00065
4.3E-06	9.1E-06	0.00126	0.00026	0.00127	0.00075
4.3E-06	0.00098	0.00016	0.0005	0.00193	0.00033
4.3E-06	0.00032	-0.0004	0.00093	0.00235	0.00102
4.4E-06	0.00048	-0.0001	-0.0003	0.00099	0.00021
4.4E-06	0.00096	0.00029	0.00021	0.00108	0.00061
4.4E-06	0.00098	8.6E-05	-0.0004	0.00209	0.00037
4.4E-06	0.00074	0.00016	-5E-05	0.00198	-0.0005
4.5E-06	0.00051	-0.0002	-0.0009	0.0017	-2E-05
4.5E-06	0.00114	-0.0003	0.00065	0.00231	0.00117
4.5E-06	0.00075	0.00013	-0.0008	0.00192	0.00017
4.6E-06	0.00074	0.00018	-7E-05	0.00225	0.00046
4.6E-06	0.00073	-0.0004	0.00048	0.00175	0.00112
4.6E-06	0.00103	-3E-05	0.00029	0.0017	0.00058
4.7E-06	0.00052	-0.0003	5.7E-05	0.00228	-0.0002
4.7E-06	0.00027	0.00074	0.00023	0.00191	0.00021
4.7E-06	0.00044	-0.0003	-0.0007	0.00149	0.00012
4.8E-06	0.00077	0.0008	0.00073	0.00226	0.00031
4.8E-06	0.00021	0.00049	0.00095	0.00186	0.00057
4.8E-06	0.00014	0.00055	-0.0005	0.00199	0.00091
4.9E-06	0.00044	0.0012	0.00037	0.0019	0.00137
4.9E-06	0.00044	0.00111	0.00026	0.00195	0.00093
4.9E-06	0.00023	0.00086	9.6E-05	0.00153	0.00042
5E-06	0.00074	0.00029	0.00031	0.00121	-0.0003
5E-06	-1E-04	0.00014	0.00031	0.00203	0.00096
5E-06	0.00092	7.1E-06	-0.0002	0.00292	0.00023
5.1E-06	0.00139	0.00019	-0.0005	0.00127	0.00071
5.1E-06	-0.0001	0.00058	0.00023	0.00184	-0.0001
5.1E-06	2.9E-05	0.00043	0.00072	0.00238	0.00033
5.2E-06	0.00074	-0.0002	0.00075	0.00277	0.00075
5.2E-06	0.00039	-0.0001	0.00031	0.00213	0.00098
5.2E-06	-0.0003	0.00024	-8E-05	0.00176	0.00115
5.2E-06	0.00064	0.00039	-0.0002	0.00209	-0.0005
5.3E-06	0.00082	0.00048	-6E-06	0.00198	-0.0001
5.3E-06	0.00096	0.00038	0.00024	0.00259	0.00017
5.3E-06	0.00063	0.00054	0.00016	0.00105	-0.0003
5.4E-06	6.7E-05	-0.0004	-0.0005	0.00159	0.00151
5.4E-06	0.00019	0.00044	-0.0005	0.0011	-0.0001
5.4E-06	0.0008	-0.0003	-0.0004	0.00262	0.00036
5.5E-06	0.00058	0.00041	-0.0003	0.00188	0.00115
5.5E-06	0.0009	-8E-05	7E-05	0.00258	0.00073
5.5E-06	0.00079	-0.0008	0.00033	0.00174	0.00065
5.6E-06	0.0011	-6E-05	0.00011	0.00185	0.00098
5.6E-06	0.00052	-0.0002	3.4E-05	0.00145	0.00043
5.6E-06	0.00012	-5E-05	-8E-05	0.00175	0.00059
5.7E-06	-0.0003	0.00078	-0.0002	0.00146	-0.0005
5.7E-06	0.00048	0.00105	0.00055	0.00114	-7E-05
5.7E-06	0.00042	0.00055	0.00017	0.00194	0.00032

5.8E-06	-0.0003	0.00025	-8E-05	0.00241	0.00017
5.8E-06	0.00036	0.00028	-0.0002	0.00162	0.00061
5.8E-06	0.00016	-0.0002	0.00043	0.00232	0.00067
5.9E-06	0.00044	0.00011	-0.0003	0.0023	0.00079
5.9E-06	0.00015	0.00024	0.00031	0.00179	0.00087
5.9E-06	0.00036	0.0006	3.2E-05	0.00111	0.00055
6E-06	0.00079	3.5E-06	0.00014	0.00173	0.00029
6E-06	-3E-05	-0.0005	0.00066	0.00173	0.00039
6E-06	0.00017	-1E-04	4.2E-05	0.00186	0.00097
6E-06	0.00044	0.00046	7.1E-05	0.00108	-0.0001
6.1E-06	0.00035	0.00018	0.00045	0.00171	0.00074
6.1E-06	0.00077	0.00043	0.00035	0.00103	0.00118
6.1E-06	0.00123	-1E-04	0.00064	0.00147	0.00121
6.2E-06	0.00016	0.00092	3.1E-06	0.00179	0.00029
6.2E-06	0.00017	0.00041	-7E-05	0.00142	0.00049
6.2E-06	0.00019	0.00038	-7E-05	0.00208	0.0004
6.3E-06	-0.0001	0.0004	-4E-05	0.00122	0.00074
6.3E-06	0.0003	0.00026	-0.0005	0.00174	-0.0001
6.3E-06	0.00067	0.00072	-0.0002	0.002	0.00036
6.4E-06	0.00045	0.0004	-0.0004	0.00128	0.00042
6.4E-06	0.00074	0.00019	0.00048	0.00121	0.00129
6.4E-06	-0.0005	0.0006	-0.0004	0.00161	0.00061
6.5E-06	0.00064	0.00027	-0.0004	0.00209	0.00074
6.5E-06	-3E-05	0.00056	-0.0003	0.00195	0.00063
6.5E-06	0.00085	0.00073	0.00093	0.00155	0.00078
6.6E-06	0.00028	0.00053	-0.0001	0.00092	0.00058
6.6E-06	0.0004	-4E-05	-0.0006	0.00243	0.00098
6.6E-06	0.00034	0.00069	-0.0003	0.00208	-3E-05
6.7E-06	0.00021	0.00052	-9E-05	0.00202	-0.0004
6.7E-06	0.00018	-0.0003	9.8E-05	0.00188	0.00022
6.7E-06	0.00098	-0.0003	-3E-05	0.00198	0.0008
6.8E-06	0.00107	0.00013	-0.0002	0.0019	0.00096
6.8E-06	-6E-05	-0.0001	-0.0002	0.00174	0.00127
6.8E-06	0.00019	0.00053	0.00035	0.00146	-4E-05
6.8E-06	0.00017	0.0002	0.0006	0.00162	0.00099
6.9E-06	0.00031	-1E-04	-0.0003	0.00276	0.00141
6.9E-06	-0.0001	-0.0003	0.00027	0.00151	0.00027
6.9E-06	0.00097	0.00017	-4E-05	0.002	0.00077
7E-06	0.00045	0.00014	-0.0002	0.00215	0.00011
7E-06	0.00047	0.00029	-9E-05	0.00203	0.00039
7E-06	0.00023	0.00033	-2E-05	0.0021	0.00101
7.1E-06	0.00042	-0.0002	-4E-05	0.00182	0.00048
7.1E-06	0.00072	-0.0003	-0.0002	0.00186	0.00066
7.1E-06	0.00028	-0.0004	-0.0005	0.00124	0.00046
7.2E-06	0.00012	-4E-05	-0.0005	0.00155	0.00033
7.2E-06	0.00067	-9E-06	5.2E-05	0.00194	-0.0003
7.2E-06	0.00065	-0.0002	0.00037	0.00185	0.00066
7.3E-06	0.00025	0.00032	-0.0003	0.00198	0.00079
7.3E-06	0.00058	2.9E-05	0.00017	0.00157	9.1E-05
7.3E-06	0.00024	-0.0005	-0.0001	0.00137	0.00131
7.4E-06	0.00057	-0.0001	0.00026	0.00228	0.00066
7.4E-06	0.00093	-7E-05	-0.0006	0.00176	0.00051
7.4E-06	0.00016	-8E-05	-6E-05	0.00128	0.00057
7.5E-06	0.00104	-0.0001	-0.0006	0.00237	0.00021

7.5E-06	0.0008	7.5E-05	-0.0003	0.00274	0.00042
7.5E-06	0.00039	-0.0003	-0.0005	0.00254	0.00073
7.6E-06	0.00057	0.0001	-0.0002	0.00184	0.00028
7.6E-06	0.00089	-0.0002	-0.0005	0.00128	0.00096
7.6E-06	0.00052	0.00033	-0.0005	0.00196	0.00093
7.6E-06	0.00058	-0.0003	-0.0002	0.00108	0.00037
7.7E-06	-0.0004	3.5E-05	-0.0003	0.00186	0.00061
7.7E-06	0.00038	0.00056	-0.0002	0.00108	0.00049
7.7E-06	0.00016	-0.0002	-0.0002	0.00201	0.00032
7.8E-06	0.00106	-2E-05	0.00076	0.00186	0.00049
7.8E-06	0.00035	0.00066	5.5E-05	0.00161	0.00035
7.8E-06	-0.0002	-0.0002	2.2E-05	0.00127	0.00035
7.9E-06	-0.0002	5.9E-05	-0.0002	0.00168	0.00058
7.9E-06	0.00048	-0.0004	-0.0003	0.00108	0.00046
7.9E-06	0.00063	-4E-05	0.00016	0.00186	0.00083
8E-06	0.00065	-0.0006	-2E-05	0.00223	0.00049
8E-06	0.00058	0.00029	-2E-05	0.00178	0.00052
8E-06	0.00013	-6E-05	-0.001	0.00217	3.8E-06
8.1E-06	0.00023	-1E-05	-0.0001	0.00131	0.0005
8.1E-06	0.00059	-0.0002	0.00038	0.0018	0.00022
8.1E-06	7E-05	0.00053	-0.0006	0.00193	0.00039
8.2E-06	-0.0001	0.00015	-0.0003	0.00213	0.00086
8.2E-06	1.5E-05	-0.0005	2.2E-05	0.00159	5.9E-05
8.2E-06	-2E-06	-0.0002	0.00017	0.00173	0.0004
8.3E-06	0.00047	-0.0004	-0.0002	0.00155	3.7E-05
8.3E-06	-0.0008	3.7E-05	7.4E-05	0.00162	0.00046
8.3E-06	0.00052	6.5E-05	-0.0004	0.00105	0.00031
8.4E-06	0.0002	-0.0004	0.00052	0.00151	0.00019
8.4E-06	-2E-05	-0.0002	-0.0005	0.00151	0.00025
8.4E-06	0.00028	-8E-05	-0.0003	0.00137	0.00039
8.4E-06	0.00018	-0.0002	0.0002	0.00214	0.00063
8.5E-06	0.0011	0.00029	0.00011	0.00181	0.00099
8.5E-06	-0.0003	0.00012	-0.0001	0.00171	0.00028
8.5E-06	6.3E-06	-3E-05	-0.0001	0.00143	0.00094
8.6E-06	0.00033	0.00026	-0.0004	0.00192	0.00039
8.6E-06	0.0003	-6E-05	-0.0003	0.00174	0.00073
8.6E-06	0.00035	0.00012	-0.0002	0.00135	0.00026
8.7E-06	0.00018	0.00038	-0.0003	0.00207	0.00049
8.7E-06	-9E-05	0.00026	-0.0005	0.00204	0.00069
8.7E-06	0.00023	0.00027	-8E-05	0.00128	0.00058
8.8E-06	0.00019	7.4E-05	-0.0005	0.00181	0.00048
8.8E-06	0.00028	-0.0002	3.1E-05	0.00137	5.2E-05
8.8E-06	0.00091	0.00055	-0.0001	0.00213	-6E-05
8.9E-06	0.00015	-0.0003	-0.0003	0.0021	0.00014
8.9E-06	0.00098	0.0008	-0.0004	0.00141	0.00102
8.9E-06	0.00105	0.00055	-0.0001	0.00222	-6E-05
9E-06	0.00159	0.00062	-2E-05	0.00204	0.00055
9E-06	6.1E-05	5.3E-06	0.00042	0.00165	0.00022
9E-06	0.00037	0.00045	-0.0004	0.00177	0.00024
9.1E-06	0.00049	-0.0001	-0.0001	0.00259	0.00048
9.1E-06	0.00067	2.7E-05	-0.0002	0.0019	0.00019
9.1E-06	0.00036	-0.0006	-0.0002	0.00126	0.0003
9.2E-06	0.0002	0.00036	0.00033	0.00106	0.00013
9.2E-06	0.00023	0.00022	0.00016	0.00255	0.00081

9.2E-06	0.00069	0.00098	-0.0007	0.00212	0.0004
9.2E-06	0.00087	0.00063	-0.0005	0.00175	0.00067
9.3E-06	0.00054	0.00067	-0.0001	0.00158	0.00019
9.3E-06	0.00021	0.00041	-8E-05	0.00171	0.00043
9.3E-06	1.1E-05	2.2E-05	6.8E-06	0.00236	6.5E-05
9.4E-06	0.00033	0.00016	-0.0006	0.00134	0.0003
9.4E-06	0.0003	0.00035	-0.0009	0.00221	0.00019
9.4E-06	0.0004	-0.0006	-0.0003	0.00149	0.00019
9.5E-06	0.0007	0.00086	-0.0002	0.00184	0.00053
9.5E-06	0.00081	0.00044	0.00034	0.00169	0.00073
9.5E-06	0.00071	2.5E-06	-0.0006	0.00201	-0.0005
9.6E-06	0.0001	9.6E-05	-8E-05	0.002	0.00041
9.6E-06	0.00063	-0.0004	-9E-05	0.00164	0.00067
9.6E-06	0.00019	0.00086	-7E-05	0.00119	0.00012
9.7E-06	0.00029	5.6E-05	-0.0004	0.00164	0.00044
9.7E-06	0.00036	-0.0002	-0.0008	0.00207	-0.0003
9.7E-06	0.0006	0.00073	-0.0006	0.00164	0.00062
9.8E-06	0.00036	0.00035	-0.0001	0.00144	0.00044
9.8E-06	0.00016	0.00051	-0.0003	0.00199	0.00082
9.8E-06	0.00043	0.00016	0.0003	0.00167	0.00059
9.9E-06	0.00108	-0.0003	-0.0003	0.00115	0.00031
9.9E-06	0.00047	-0.0003	-0.0008	0.00156	-0.0002
9.9E-06	0.00024	0.00024	-0.0004	0.00162	-4E-05
1E-05	0.00041	-4E-06	-0.0001	0.00166	0.00073
1E-05	0.00041	6.9E-05	-0.0002	0.00128	0.00055
1E-05	0.00087	-0.0003	-0.0005	0.00189	0.00035
1E-05	0.0009	0.00017	-0.0002	0.00167	0.00045
1E-05	0.00131	-0.0001	-0.0002	0.0023	0.00026
1E-05	0.00017	0.00034	0.0004	0.00197	0.00011
1E-05	0.00078	0.00032	-1E-05	0.00126	-0.0002
1E-05	0.00019	0.00018	-0.0002	0.00114	0.00025
1E-05	0.00072	0.00062	-0.0004	0.00185	0.00039
1E-05	-1E-05	0.00072	-0.0004	0.0016	0.00064
1E-05	0.00034	0.00039	0.00036	0.00162	-0.0003
1E-05	-8E-05	-2E-05	-0.0003	0.00205	-0.0001
1E-05	0.00114	0.00061	0.0003	0.00164	0.00048
1E-05	0.00093	-4E-05	-0.0007	0.00207	0.00018
1E-05	0.00053	-8E-05	-0.0001	0.00227	7E-05
1E-05	-0.0003	0.00018	-0.0003	0.00105	0.00019
1E-05	0.00095	0.00055	-0.0002	0.00167	0.0004
1.1E-05	0.00045	0.00015	0.00043	0.00127	0.00027
1.1E-05	-0.0001	0.00088	-0.0004	0.00147	0.00051
1.1E-05	0.0005	0.00029	-0.0004	0.00167	-0.0001
1.1E-05	0.00049	-7E-05	0.00043	0.00132	0.00021
1.1E-05	0.00038	-0.0002	-1E-04	0.00156	-0.0002
1.1E-05	0.0007	-0.0001	-0.0003	0.00196	0.00024
1.1E-05	0.00104	0.00041	-0.0004	0.00127	0.00013
1.1E-05	0.00023	0.00024	-0.0003	0.00162	0.00048
1.1E-05	0.00037	-0.0008	0.00056	0.0016	0.00045
1.1E-05	0.00031	-0.0003	-0.0002	0.00128	0.00088
1.1E-05	0.0004	2.7E-05	0.00013	0.00164	0.00052
1.1E-05	0.00063	0.00014	-6E-05	0.00196	0.00062
1.1E-05	0.00059	0.00035	0.0004	0.00185	0.00064
1.1E-05	-0.0003	0.00045	-0.0002	0.00149	0.00079

1.1E-05	0.00074	0.00026	0.00021	0.00147	0.00059
1.1E-05	0.00075	-0.0002	1.3E-05	0.00207	0.00023
1.1E-05	-0.0004	0.00076	-0.0006	0.00227	0.00024
1.1E-05	0.00021	0.00064	-0.0005	0.00116	-3E-05
1.1E-05	0.00029	0.00077	-0.0005	0.00178	0.00048
1.1E-05	0.0002	0.00056	-0.0002	0.0021	-4E-05
1.1E-05	-0.0003	0.00034	-0.0006	0.00221	0.00067
1.1E-05	-0.0002	0.00043	-0.0004	0.00201	0.00056
1.1E-05	-4E-05	0.00036	0.00011	0.00175	0.00017
1.1E-05	0.00011	0.00037	0.00044	0.00183	-0.0001
1.1E-05	1.9E-05	0.00047	-0.0003	0.00154	0.0004
1.1E-05	0.00027	-0.0002	5.1E-05	0.00159	0.00025
1.1E-05	8.1E-05	0.00024	-0.0005	0.0014	0.00012
1.1E-05	0.00045	7.8E-05	-0.0001	0.00133	1E-04
1.1E-05	0.00012	0.00053	-0.0006	0.00095	0.00052
1.1E-05	-0.0003	-0.0008	-0.0002	0.00135	0.00011
1.1E-05	-1E-05	0.00015	7.8E-05	0.00133	5.8E-05
1.1E-05	0.0002	0.00011	-0.0006	0.00173	-7E-05
1.2E-05	0.00046	0.0012	-0.0006	0.00205	0.0002
1.2E-05	3.5E-05	-2E-05	-0.0004	0.00121	0.00019
1.2E-05	0.00036	-1E-05	-0.0002	0.00184	0.00058
1.2E-05	0.00018	0.00062	-0.0004	0.00152	0.00014
1.2E-05	-0.0008	9.4E-05	-0.0002	0.00157	-0.0003
1.2E-05	0.00066	-0.0003	0.0003	0.00169	0.00052
1.2E-05	3.6E-05	0.00055	-0.0003	0.00191	0.0006
1.2E-05	0.00035	0.00037	-0.0004	0.00168	0.00013
1.2E-05	0.0005	0.00018	-0.0002	0.00217	0.00043
1.2E-05	0.00076	0.00046	-0.0002	0.00203	5.2E-05
1.2E-05	-0.0005	0.00054	-0.0007	0.00161	0.00047
1.2E-05	0.00049	0.00105	-0.0004	0.00156	-0.0003
1.2E-05	0.00053	0.00034	-0.0004	0.00135	0.00032
1.2E-05	0.00032	-6E-06	-0.0005	0.00144	0.00017
1.2E-05	1.8E-05	5.8E-05	-0.0005	0.00134	0.00044
1.2E-05	-0.0002	1.6E-05	-0.0002	0.0008	0.00017
1.2E-05	-0.0001	-0.0004	-0.0004	0.00159	0.00015
1.2E-05	0.00108	-0.0004	-0.0003	0.00182	-5E-05
1.2E-05	-0.0002	-2E-06	-2E-05	0.0015	0.00033
1.2E-05	0.00073	-0.0003	-3E-05	0.00194	0.00042
1.2E-05	0.00031	-0.0002	0.00011	0.0022	5.5E-05
1.2E-05	0.0001	0.00056	-0.0006	0.00159	0.00046
1.2E-05	0.00024	0.00034	-7E-06	0.00175	0.00027
1.2E-05	0.00043	0.00086	-0.0008	0.00183	0.00044
1.2E-05	0.00029	9.5E-06	-0.0004	0.00087	0.00018
1.2E-05	8.3E-05	0.0009	-0.0006	0.00094	0.00032
1.2E-05	0.00062	0.00016	0.00028	0.00162	0.00036
1.2E-05	0.00065	0.00063	-0.0006	0.00191	-3E-05
1.2E-05	0.00028	0.00014	-0.0002	0.00157	-0.0001
1.2E-05	0.00042	-0.0006	-0.0003	0.00175	0.00032
1.2E-05	0.0003	0.00086	-0.0005	0.00174	4.2E-05
1.3E-05	0.00062	-0.001	-0.0005	0.00224	0.00017
1.3E-05	0.00084	-7E-05	0.00019	0.00114	5.5E-05
1.3E-05	0.00038	0.0002	-0.0009	0.00273	0.00037
1.3E-05	-4E-05	0.00032	-0.0005	0.00146	0.00031
1.3E-05	0.00068	0.0005	-0.0006	0.00157	-0.0004

1.3E-05	-2E-05	9.6E-05	-0.0007	0.00175	-0.0003
1.3E-05	0.00033	0.0009	-0.0005	0.0018	0.00048
1.3E-05	0.00054	-0.0002	1.9E-05	0.00183	0.0002
1.3E-05	0.00042	-0.0002	-0.0012	0.00177	0.00038
1.3E-05	0.00041	0.00015	-0.0008	0.00128	0.00072
1.3E-05	-0.0001	0.00051	-0.0004	0.00166	0.00047
1.3E-05	0.00081	0.00028	-0.0002	0.00153	0.00021
1.3E-05	-0.0002	0.00073	-0.0003	0.00183	8.4E-05
1.3E-05	-0.0004	-9E-05	-0.0003	0.00121	0.00068
1.3E-05	0.00023	0.0005	-7E-05	0.00161	7.5E-05
1.3E-05	5.2E-05	-8E-05	-1E-05	0.0017	0.00012
1.3E-05	1.9E-05	0.00071	0.00041	0.00153	-0.0007
1.3E-05	-7E-05	0.00066	-0.0005	0.00129	0.00022
1.3E-05	0.00034	0.00063	7.2E-06	0.00151	0.0006
1.3E-05	0.00061	0.00012	-0.0003	0.00179	0.00045
1.3E-05	-8E-05	0.00017	-6E-05	0.00142	0.00019
1.3E-05	6.1E-05	-2E-05	-3E-05	0.00085	-7E-05
1.3E-05	-0.0002	0.00016	-0.0005	0.00173	-3E-05
1.3E-05	0.00017	0.00085	-0.0004	0.00164	0.0003
1.3E-05	-0.0003	0.00094	-0.0003	0.00156	-0.0003
1.3E-05	-4E-05	0.0004	-0.0002	0.00155	-0.0003
1.3E-05	-7E-05	0.00108	-0.0005	0.00134	0.00064
1.3E-05	-0.0001	5.4E-05	-0.0004	0.00128	0.00024
1.3E-05	0.00015	0.00017	0.00019	0.00164	0.00037
1.3E-05	-2E-05	2.8E-05	-2E-05	0.00137	0.00112
1.3E-05	0.00019	0.00027	-0.0003	0.00121	0.0007
1.4E-05	-0.0001	-0.0004	-0.0002	0.002	-9E-05
1.4E-05	0.00019	0.00071	0.00039	0.00175	4.3E-05
1.4E-05	0.00013	0.00023	-0.0003	0.00198	0.00054
1.4E-05	-0.0001	0.00065	-0.0007	0.0016	0.00062
1.4E-05	7.3E-05	0.00069	-0.0006	0.00115	0.00078
1.4E-05	-0.0001	0.00019	-0.0004	0.0023	0.00105
1.4E-05	0.00091	-8E-06	9.4E-06	0.00145	0.00044
1.4E-05	0.00048	0.00094	-0.0005	0.00134	0.00042
1.4E-05	0.00063	0.00016	-0.0002	0.00179	0.00017
1.4E-05	0.00038	0.00011	-0.0008	0.00156	0.00012
1.4E-05	0.00037	0.00049	-0.0009	0.00107	-0.0002
1.4E-05	-4E-05	0.00065	-0.0002	0.00122	-0.0002
1.4E-05	0.001	0.00079	-0.0011	0.00099	0.0004
1.4E-05	0.00033	0.00014	0.00013	0.00157	0.00012
1.4E-05	-0.0001	0.0004	0.00024	0.00161	0.00028
1.4E-05	-0.0002	0.00067	1.4E-05	0.00173	6.1E-05
1.4E-05	0.00022	0.00034	-0.0008	0.00173	-5E-05
1.4E-05	0.00016	0.00101	-5E-05	0.00124	0.00028
1.4E-05	0.00012	7.5E-05	-8E-05	0.00193	-3E-05
1.4E-05	0.0007	0.00071	4.9E-05	0.00148	0.00036
1.4E-05	0.0005	0.00036	-0.0004	0.00133	0.00014
1.4E-05	-0.0003	0.00057	-0.0004	0.00129	0.00025
1.4E-05	-0.0002	0.00011	-0.0005	0.00116	-0.0005
1.4E-05	6.8E-05	-0.0001	-8E-05	0.00172	-0.0001
1.4E-05	0.00034	0.00022	-0.0005	0.00138	0.00028
1.4E-05	0.00023	0.00041	-0.0003	0.00195	-0.0003
1.4E-05	0.00036	0.00031	-0.0004	0.00191	-3E-05
1.4E-05	-0.0001	0.00095	-0.0001	0.00133	0.00017

1.4E-05	0.00041	0.00049	-0.0008	0.00186	-0.0004
1.4E-05	-8E-05	-3E-05	-0.0004	0.0013	0.00036
1.4E-05	-0.0003	0.0008	0.00069	0.00117	-8E-05
1.5E-05	0.00016	0.00093	-0.0003	0.00207	0.00052
1.5E-05	-0.0004	-9E-05	-5E-05	0.00212	0.00127
1.5E-05	-0.0002	0.00104	0.00076	0.00203	-0.0009
1.5E-05	0.00039	0.00122	-0.0002	0.00149	0.00023
1.5E-05	-0.0002	-5E-05	-0.0004	0.00201	-0.0007
1.5E-05	-1E-05	-0.0003	-0.0006	0.00188	-0.0003
1.5E-05	0.00068	-0.0003	-3E-05	0.00203	-0.0001
1.5E-05	0.00017	0.00089	2.3E-05	0.00232	-0.0004
1.5E-05	-0.0002	0.0004	-0.0003	0.00149	0.00039
1.5E-05	-0.0004	0.00018	-0.0003	0.00186	0.0009
1.5E-05	0.0003	0.00056	-0.0007	0.00189	-3E-05
1.5E-05	-4E-05	-2E-05	-5E-05	0.00133	0.00048
1.5E-05	-0.0001	-0.0001	-0.0004	0.00173	0.00035
1.5E-05	0.00033	0.00033	-0.0005	0.00142	-0.0004
1.5E-05	3.9E-05	-0.0002	0.00071	0.00141	-2E-05
1.5E-05	0.00017	4.2E-05	0.0007	0.00109	1.1E-05

Table SI.7: Primary kinetic data for the reaction of 1-phenyl-2-pyrrolidone (**S7**) with CumO• generated by 355 nm LFP in MeCN (**Figure SI.7**).

**t / s**	**ΔA 1-phenyl-2-pyrrolidone (S7)**				
	**0.080M**	**0.235M**	**0.400M**	**0.592M**	**0.799M**
-1E-06	-0.0007	0.00085	0.00018	-0.0003	-0.0004
-1E-06	-0.0009	0.00166	-0.0007	0.00131	-0.0008
-9E-07	-0.0006	0.00053	-0.0007	0.00052	1.6E-05
-9E-07	-0.0005	0.0014	-0.0004	0.0008	0.00052
-9E-07	9.5E-05	0.00049	-0.0005	0.00023	-0.0004
-8E-07	-0.0004	-0.0001	-0.0011	0.0008	0.0006
-8E-07	-0.0002	0.00017	-0.0009	0.00033	-0.0003
-8E-07	-0.0007	0.00049	-0.0005	0.00092	-0.0002
-7E-07	-0.0005	4.2E-05	-0.0004	0.00084	-0.0005
-7E-07	-0.0002	0.00045	-0.0001	0.00019	-0.0003
-7E-07	0.00086	0.00039	-0.0003	0.00102	-0.0006
-6E-07	7E-05	0.00106	-0.0008	0.0008	-0.0004
-6E-07	0.00019	0.0007	-0.0006	0.00073	-0.0003
-6E-07	-0.0004	0.00109	-0.0005	0.00039	-0.0004
-5E-07	0.00033	0.00048	-0.0012	0.00061	-0.0004
-5E-07	-0.0002	0.00206	-0.0001	0.00063	-0.0016
-5E-07	0.00033	-0.0004	-0.0004	0.00053	-0.0008
-4E-07	-0.0001	0.0015	-0.0003	0.00079	-0.0004
-4E-07	-0.0005	0.00061	-0.0006	0.00094	-0.0005
-4E-07	-0.0012	0.0013	-0.0007	0.00085	-0.0008
-4E-07	-0.0003	0.00089	-0.0006	0.00078	-0.0006
-3E-07	-0.0005	0.00029	-0.001	0.00038	-0.0003
-3E-07	0.00022	0.00059	-0.0007	0.00058	-0.0005
-3E-07	-0.0006	0.00078	-0.0001	0.00154	-0.0009
-2E-07	-0.0003	0.00089	0.00063	0.00086	-0.0002
-2E-07	3.8E-05	0.00139	-0.0011	0.00063	-0.0006
-2E-07	-2E-05	0.00151	-0.0004	0.00113	-0.0005
-1E-07	-0.0006	0.00072	0.00025	0.00051	-0.0007
-1E-07	0.00013	0.00085	-0.0006	0.00109	-5E-05
-6E-08	0.00061	0.00057	-0.0002	0.00064	-0.0005
-3E-08	0.00038	0.00073	-0.0003	0.00104	0.00015
-4E-10	-0.0033	-0.0029	-0.0036	-0.0022	-0.0039
3.2E-08	-0.0084	-0.0097	-0.0143	-0.0119	-0.0139
6.4E-08	0.01511	0.01743	0.01654	0.01691	0.016
9.6E-08	0.01523	0.01783	0.01685	0.01884	0.01753
1.3E-07	0.0144	0.01741	0.01558	0.01671	0.0154
1.6E-07	0.01345	0.01628	0.01432	0.01519	0.01486
1.9E-07	0.01276	0.01521	0.0136	0.01487	0.01359
2.2E-07	0.01219	0.01462	0.01245	0.01441	0.01298
2.6E-07	0.01269	0.01506	0.01217	0.01391	0.01229
2.9E-07	0.01247	0.01379	0.01195	0.01247	0.01126
3.2E-07	0.01265	0.0136	0.0102	0.01225	0.01029
3.5E-07	0.01131	0.01313	0.01104	0.01075	0.00955
3.8E-07	0.01125	0.01182	0.00986	0.0108	0.0093
4.2E-07	0.01056	0.01181	0.00918	0.0104	0.00881
4.5E-07	0.01058	0.01175	0.00941	0.01007	0.00822
4.8E-07	0.0101	0.01094	0.00892	0.00843	0.00717
5.1E-07	0.01029	0.00923	0.00797	0.00857	0.00644
5.4E-07	0.00972	0.01035	0.00779	0.00835	0.00662

5.8E-07	0.00906	0.00941	0.00682	0.00769	0.00619
6.1E-07	0.00832	0.00972	0.00697	0.00736	0.00519
6.4E-07	0.00845	0.0086	0.00702	0.00771	0.00496
6.7E-07	0.00893	0.00854	0.00654	0.00634	0.00517
7E-07	0.00908	0.00944	0.00569	0.00725	0.00455
7.4E-07	0.00863	0.00825	0.00514	0.00633	0.00395
7.7E-07	0.00716	0.00785	0.00543	0.00591	0.00415
8E-07	0.00662	0.00807	0.00538	0.00556	0.00412
8.3E-07	0.00726	0.00761	0.00495	0.00511	0.00361
8.6E-07	0.00663	0.00712	0.00401	0.00502	0.00315
9E-07	0.00625	0.00766	0.00465	0.00452	0.0031
9.3E-07	0.00626	0.00693	0.00483	0.00455	0.00247
9.6E-07	0.00591	0.00681	0.00414	0.0042	0.00257
9.9E-07	0.00665	0.00594	0.00351	0.00416	0.0027
1E-06	0.00481	0.00591	0.00301	0.00389	0.00271
1.1E-06	0.00598	0.00637	0.00283	0.00382	0.00201
1.1E-06	0.0056	0.00674	0.00341	0.00394	0.00175
1.1E-06	0.00501	0.00603	0.00276	0.00361	0.00169
1.2E-06	0.00546	0.00585	0.00242	0.00378	0.00186
1.2E-06	0.00511	0.00511	0.00258	0.00281	0.00119
1.2E-06	0.00522	0.00563	0.00315	0.00287	0.00107
1.2E-06	0.00497	0.00527	0.00216	0.00261	0.00127
1.3E-06	0.00597	0.00498	0.00277	0.00296	0.00186
1.3E-06	0.00464	0.00529	0.00265	0.00287	0.00105
1.3E-06	0.00433	0.00426	0.00256	0.00279	0.00114
1.4E-06	0.00441	0.00578	0.00148	0.0028	0.00118
1.4E-06	0.00463	0.00484	0.00144	0.00273	0.00149
1.4E-06	0.00485	0.00467	0.00164	0.00303	0.00112
1.5E-06	0.00356	0.00476	0.00136	0.00166	0.00173
1.5E-06	0.00364	0.00505	0.00239	0.00239	0.00188
1.5E-06	0.00387	0.00426	0.00157	0.00246	0.00157
1.6E-06	0.00322	0.00372	0.00233	0.00239	0.00114
1.6E-06	0.00351	0.00397	0.00155	0.00245	0.00111
1.6E-06	0.00338	0.00372	0.00167	0.00224	0.00075
1.7E-06	0.00362	0.00378	0.00094	0.0022	0.00087
1.7E-06	0.00239	0.00379	0.00144	0.00205	0.00055
1.7E-06	0.00307	0.00361	0.00158	0.00161	0.00046
1.8E-06	0.00279	0.00342	0.00067	0.00206	0.00073
1.8E-06	0.00283	0.00366	0.00203	0.00197	0.00141
1.8E-06	0.00227	0.00297	0.00084	0.00215	0.00085
1.9E-06	0.00257	0.00323	0.0012	0.00144	0.00092
1.9E-06	0.00233	0.00318	0.00109	0.00196	0.00021
1.9E-06	0.00242	0.00227	0.00013	0.0025	0.00125
2E-06	0.00218	0.00312	0.00114	0.00121	0.00101
2E-06	0.00212	0.00359	0.00063	0.00171	0.00095
2E-06	0.00254	0.00332	0.00081	0.002	0.00123
2E-06	0.00224	0.0025	0.00065	0.0011	0.00078
2.1E-06	0.00266	0.00326	0.00083	0.00163	0.00091
2.1E-06	0.00183	0.00309	0.00142	0.00176	-2E-05
2.1E-06	0.00229	0.00313	0.00074	0.00241	0.00178
2.2E-06	0.00272	0.00343	0.00051	0.00167	-0.0002
2.2E-06	0.00228	0.00306	0.00065	0.00203	0.00049
2.2E-06	0.00299	0.00321	0.00122	0.00138	0.00139
2.3E-06	0.00211	0.00219	0.00077	0.00194	-5E-05

2.3E-06	0.00232	0.00295	0.00066	0.0014	0.00094
2.3E-06	0.00245	0.00193	0.00148	0.00171	0.00103
2.4E-06	0.00249	0.00277	0.00109	0.0018	-0.0003
2.4E-06	0.00158	0.00284	0.0012	0.00212	0.00056
2.4E-06	0.00194	0.0036	0.00083	0.00169	0.00166
2.5E-06	0.00199	0.00243	0.00069	0.00229	0.00063
2.5E-06	0.00179	0.00206	0.00037	0.00229	0.00082
2.5E-06	0.00111	0.00276	0.00022	0.00155	0.00115
2.6E-06	0.00222	0.00251	0.00085	0.00203	0.00123
2.6E-06	0.00182	0.002	0.0004	0.00159	-0.0001
2.6E-06	0.00133	0.00267	0.00051	0.00091	0.00051
2.7E-06	0.00229	0.00258	0.00037	0.00179	0.00153
2.7E-06	0.0021	0.00245	0.00069	0.00089	-0.0002
2.7E-06	0.0016	0.00198	0.00037	0.00059	0.00066
2.8E-06	0.00092	0.00204	0.00057	0.00116	0.00093
2.8E-06	0.00191	0.00209	0.00064	0.00127	-0.0001
2.8E-06	0.0016	0.00204	0.00099	0.0011	0.00072
2.8E-06	0.00129	0.00215	0.00068	0.00161	0.00058
2.9E-06	0.00203	0.00259	-0.0002	0.00112	0.00059
2.9E-06	0.00176	0.00225	0.00091	0.00112	0.00035
2.9E-06	0.00099	0.00217	4.4E-05	0.00119	0.00058
3E-06	0.00128	0.00203	0.00073	0.00116	2.1E-05
3E-06	0.00213	0.00205	1.1E-05	0.00174	0.0003
3E-06	0.00141	0.00142	0.00053	0.00062	0.00067
3.1E-06	0.00192	0.00219	0.001	0.00133	-8E-05
3.1E-06	0.00172	0.00215	0.00037	0.00144	0.00055
3.1E-06	0.00086	0.00239	-0.0002	0.0014	8.2E-05
3.2E-06	0.00115	0.00271	0.00014	0.0007	0.00035
3.2E-06	0.0005	0.00289	-0.0008	0.00134	-0.0004
3.2E-06	0.00066	0.00215	0.00033	0.00175	0.00077
3.3E-06	0.00131	0.00227	-0.0005	0.00126	0.00062
3.3E-06	0.00126	0.00184	-0.0002	0.00128	0.0003
3.3E-06	0.0013	0.00151	0.00027	0.00122	0.00031
3.4E-06	0.00214	0.00169	-0.0003	0.00129	7.5E-05
3.4E-06	0.00043	0.00177	0.00082	0.00135	0.00039
3.4E-06	0.00096	0.0013	-0.0005	0.00153	0.00085
3.5E-06	0.00159	0.00215	0.00069	0.00186	0.00054
3.5E-06	0.00054	0.00176	0.00021	0.00152	0.00133
3.5E-06	0.00063	0.00137	0.00015	0.00152	-0.0002
3.6E-06	0.00135	0.00226	2.1E-05	0.0012	0.00027
3.6E-06	0.00038	0.00179	-0.0004	0.00099	0.00071
3.6E-06	0.00149	0.00135	-2E-05	0.00142	0.00148
3.6E-06	0.00037	0.00139	-6E-06	0.00165	0.00033
3.7E-06	0.0006	0.00134	8.6E-05	0.00195	0.0001
3.7E-06	0.00051	0.00137	0.00071	0.00101	0.00041
3.7E-06	0.00065	0.00122	0.00074	0.00052	-0.0001
3.8E-06	-0.0002	0.00108	0.00043	0.00123	0.00025
3.8E-06	0.00043	0.00115	-6E-05	0.00099	-9E-05
3.8E-06	0.00052	0.00183	0.00048	0.00168	0.00011
3.9E-06	0.00071	0.00121	-0.0004	0.00139	0.00103
3.9E-06	7.1E-05	0.00126	-4E-05	0.00187	0.00066
3.9E-06	0.00061	0.00141	0.00057	0.00138	-0.0001
4E-06	0.0009	0.00169	-0.0004	0.00096	-0.0003
4E-06	9.7E-05	0.002	0.00021	0.00192	-0.0002

4E-06	0.00073	0.00196	-0.0002	0.00161	0.00011
4.1E-06	0.0011	0.00118	4.4E-05	0.00206	0.00036
4.1E-06	0.00063	0.00205	-6E-06	0.00134	0.00054
4.1E-06	0.00038	0.00153	0.00024	0.00141	0.00051
4.2E-06	0.00101	0.00137	0.00064	0.00206	0.00059
4.2E-06	0.00109	0.00105	8.8E-05	0.00089	0.00119
4.2E-06	8.4E-05	0.00251	0.00051	0.00208	0.00016
4.3E-06	0.00023	0.0019	-0.0005	0.00131	0.0005
4.3E-06	0.00079	0.00208	-7E-05	0.00107	-4E-06
4.3E-06	-0.0001	0.00117	0.00012	0.0014	0.00072
4.4E-06	0.00111	0.00143	-0.0011	0.00114	0.00035
4.4E-06	0.00074	0.00136	9.7E-05	0.00111	0.00059
4.4E-06	0.00039	0.00169	3.1E-05	0.00157	0.00063
4.4E-06	0.0003	0.00222	-0.0002	0.00154	-0.0005
4.5E-06	0.00062	0.00148	-5E-05	0.00125	0.00082
4.5E-06	0.00054	0.00192	-0.0001	0.00087	0.00086
4.5E-06	-5E-06	0.00159	-1E-04	0.00109	0.00059
4.6E-06	7.1E-05	0.00144	2E-05	0.00178	0.00055
4.6E-06	-7E-05	0.00159	-7E-05	0.00187	0.00067
4.6E-06	0.00081	0.00101	0.0005	0.00113	0.00041
4.7E-06	-0.0002	0.00161	-0.0001	0.00119	0.00071
4.7E-06	0.00044	0.00139	0.00033	0.00101	0.00071
4.7E-06	0.00042	0.0017	-0.0004	0.00049	0.00071
4.8E-06	-9E-05	0.0013	-0.0005	0.00139	0.00067
4.8E-06	0.00028	0.00093	-0.0002	0.00085	0.0013
4.8E-06	0.00041	0.00132	-0.0001	0.0012	0.00034
4.9E-06	0.00072	0.0016	-0.0004	0.00189	0.00051
4.9E-06	-0.0002	0.0011	-0.0004	0.00137	0.00016
4.9E-06	0.00043	0.00065	-0.0005	0.00167	0.00028
5E-06	0.00102	0.00054	0.00013	0.00172	0.0008
5E-06	0.00048	0.00087	0.00027	0.00168	0.00013
5E-06	0.00026	0.00165	-8E-05	0.00129	0.00045
5.1E-06	0.00022	0.00115	-0.0002	0.00034	-0.0001
5.1E-06	-0.0002	0.00183	0.00033	0.00095	0.00111
5.1E-06	0.00025	0.00189	-0.0005	0.00146	3E-06
5.2E-06	-3E-06	0.00097	-1E-04	0.00149	0.00041
5.2E-06	0.00029	0.00145	-0.0005	0.00111	0.00081
5.2E-06	0.00017	0.00176	0.00047	0.00097	-8E-05
5.2E-06	-0.0003	0.00232	0.00056	0.00151	0.00015
5.3E-06	6.4E-05	0.0017	-0.0003	0.00056	0.00036
5.3E-06	0.00049	0.00151	-0.0002	0.00028	0.00028
5.3E-06	0.0004	0.00125	-0.0003	0.00094	0.00097
5.4E-06	0.00065	0.00185	-3E-05	0.00098	0.00038
5.4E-06	5.3E-05	0.00195	0.00022	0.00071	0.00049
5.4E-06	-0.0002	0.00157	-0.0004	0.0011	0.0011
5.5E-06	0.00016	0.00185	5.9E-05	0.00109	0.00082
5.5E-06	0.00048	0.00129	0.00035	0.00125	0.0008
5.5E-06	-0.0003	0.00188	-0.0002	0.00137	0.0007
5.6E-06	0.00026	0.00166	-2E-05	0.00198	0.00087
5.6E-06	0.0003	0.00212	0.00058	0.00158	0.00065
5.6E-06	0.00055	0.00108	0.00014	0.00117	0.00024
5.7E-06	0.00012	0.00133	-0.0001	0.00147	0.00061
5.7E-06	0.00051	0.00112	-0.0004	0.00197	0.00036
5.7E-06	-0.0001	0.0012	0.00011	0.00104	0.00067

5.8E-06	0.00057	0.00051	-0.0004	0.0012	0.00038
5.8E-06	0.0008	0.00208	0.00038	0.00166	0.00061
5.8E-06	-0.0002	0.00183	-0.0007	0.00113	0.00044
5.9E-06	-0.0001	0.00167	3.1E-06	0.00076	0.00057
5.9E-06	-0.0005	0.00132	-0.0002	0.00152	0.00045
5.9E-06	0.00034	0.00158	0.00029	0.00109	0.00024
6E-06	0.00052	0.0011	-0.0004	0.00132	0.00072
6E-06	2.4E-06	0.00106	-9E-05	0.00188	0.00113
6E-06	-0.0002	0.00024	0.0003	0.00085	0.00016
6E-06	-4E-05	0.00151	6.7E-05	0.00175	0.00013
6.1E-06	0.00032	0.00205	-0.0004	0.00147	0.0007
6.1E-06	0.00018	0.00164	7.7E-05	0.00152	0.00035
6.1E-06	0.00028	0.00115	-0.0001	0.00088	0.00093
6.2E-06	0.00052	0.0014	4.1E-05	0.00167	0.00114
6.2E-06	-0.0001	0.00199	0.00039	0.00155	0.00035
6.2E-06	2.2E-05	0.00084	0.00016	0.00106	0.00118
6.3E-06	0.00026	0.00102	0.00017	0.00136	0.00099
6.3E-06	0.00062	0.00142	-0.0004	0.0011	8.9E-05
6.3E-06	-0.0003	0.00084	0.00043	0.00072	0.00083
6.4E-06	-0.0001	0.00127	-0.0006	0.00105	0.00062
6.4E-06	-0.0001	0.00165	-0.0005	0.00133	0.00056
6.4E-06	0.00017	0.00197	0.00056	0.0014	0.00056
6.5E-06	0.00014	0.00066	-0.0001	0.00089	0.00057
6.5E-06	0.00039	0.0013	-0.0007	0.00111	0.00081
6.5E-06	0.00026	0.00103	-0.0005	0.00106	0.00033
6.6E-06	0.00022	0.0011	0.00026	0.00075	0.00072
6.6E-06	0.00038	0.00077	-0.0004	0.0012	0.00069
6.6E-06	0.0006	0.00113	0.00023	0.00106	0.00093
6.7E-06	5E-05	0.00142	-0.0004	0.00054	0.00092
6.7E-06	-0.0005	0.00144	0.00026	0.00122	0.00056
6.7E-06	0.00043	0.00187	-0.0005	0.00188	-0.0002
6.8E-06	0.00054	0.00152	0.00054	0.00115	0.0006
6.8E-06	0.00062	0.00101	0.00044	0.00107	0.00042
6.8E-06	0.00036	0.00114	0.00026	0.00088	7.3E-05
6.8E-06	0.00077	0.00076	-0.0004	0.001	0.00046
6.9E-06	0.00012	0.00129	-7E-05	0.00095	0.00083
6.9E-06	0.0008	0.00069	-0.0009	0.00125	0.00059
6.9E-06	-3E-05	0.00117	0.00016	0.00094	0.0004
7E-06	0.00026	0.00093	-0.0004	0.00155	0.0003
7E-06	0.0002	0.00091	-0.0002	0.00157	0.00013
7E-06	0.00119	0.0008	-0.0002	0.00143	0.00023
7.1E-06	0.00051	0.00142	2.4E-05	0.00178	0.00029
7.1E-06	0.00045	0.00097	-7E-05	0.0017	0.00011
7.1E-06	0.0003	0.00071	0.00011	0.00125	0.00035
7.2E-06	8.6E-05	0.00112	0.00018	0.00122	0.00047
7.2E-06	6.3E-06	0.00025	0.00018	0.00114	0.00048
7.2E-06	-0.0004	0.00117	-0.0001	0.00135	0.00029
7.3E-06	0.00044	0.00165	-5E-05	0.00117	0.00057
7.3E-06	0.00053	0.00094	-0.0008	0.00084	0.00073
7.3E-06	0.00048	0.00118	-0.0002	0.00187	0.00053
7.4E-06	-1E-04	0.00075	-0.0003	0.00152	0.00028
7.4E-06	-0.0005	0.00146	-0.0002	0.00069	0.00033
7.4E-06	0.0002	0.00135	-0.0001	0.00125	0.00039
7.5E-06	0.00113	0.00086	-0.0002	0.00038	0.00044

7.5E-06	0.00028	0.00132	-0.0003	0.00169	0.00062
7.5E-06	-0.0002	0.00102	0.00068	0.00152	0.00054
7.6E-06	1.2E-05	0.00169	-0.0005	0.00079	0.00077
7.6E-06	0.00115	0.00125	-0.0005	0.00128	-0.0002
7.6E-06	0.00053	0.00113	-0.0009	0.00157	0.00059
7.6E-06	0.00021	0.0009	0.00042	0.00087	-0.0002
7.7E-06	0.00027	0.00174	-0.0005	0.00102	-9E-05
7.7E-06	0.00086	0.0011	0.00011	0.00123	0.00049
7.7E-06	0.00017	0.00152	-3E-05	0.00114	0.00045
7.8E-06	0.00018	0.0003	-0.0007	0.00111	0.00081
7.8E-06	5.1E-05	0.00104	-0.0007	0.00109	0.00061
7.8E-06	-0.0004	0.00102	-0.0003	0.00136	0.00107
7.9E-06	-0.0003	0.00101	-0.0004	0.00167	0.00059
7.9E-06	-0.0001	0.00161	-0.0002	0.00078	8.6E-05
7.9E-06	0.001	0.00112	-0.0006	0.00089	0.00042
8E-06	2.1E-05	0.0008	-0.0001	0.00087	0.00057
8E-06	0.0006	0.00077	0.00022	0.00117	0.00017
8E-06	-1E-04	0.00133	1.6E-05	0.00201	0.00047
8.1E-06	0.00023	0.00134	-0.0003	0.0013	0.00018
8.1E-06	-0.0002	0.00112	0.00022	0.00131	0.00011
8.1E-06	0.00044	0.00145	-0.0005	0.00121	0.00044
8.2E-06	0.00041	0.0008	0.00031	0.00151	0.00033
8.2E-06	0.00041	0.00063	-0.0003	0.00043	0.00051
8.2E-06	-0.0002	0.00127	-0.0004	0.00199	0.00082
8.3E-06	-0.0008	0.00093	0.00016	0.00178	0.00024
8.3E-06	-3E-05	0.00018	-0.0006	0.0006	0.0002
8.3E-06	0.00044	0.00086	2.5E-05	0.00077	0.00045
8.4E-06	-0.0011	0.00061	4E-05	0.001	0.00043
8.4E-06	-0.0006	0.001	-0.0004	0.0021	0.00037
8.4E-06	-0.0002	0.00111	0.00011	0.00081	-0.0001
8.4E-06	0.00027	0.00132	-0.0002	0.00075	-0.0003
8.5E-06	-0.0006	0.00113	-0.0002	0.00096	0.00034
8.5E-06	-0.0008	0.00103	2.9E-05	0.0014	0.0006
8.5E-06	-7E-05	0.00143	-0.0002	0.00073	0.00045
8.6E-06	0.00043	0.00132	-0.0009	0.00082	7.5E-05
8.6E-06	0.00025	0.00092	-8E-05	0.00094	0.00046
8.6E-06	9.3E-05	0.00104	0.00017	0.00035	0.00017
8.7E-06	-0.0003	0.00032	-0.0006	0.00107	0.00024
8.7E-06	9E-05	0.00117	0.00027	0.00136	-0.0002
8.7E-06	0.0003	0.0009	8E-05	0.00146	2.2E-05
8.8E-06	-0.0005	0.0011	-1E-04	0.00138	0.00092
8.8E-06	-0.0002	0.00047	-5E-05	0.00127	0.00066
8.8E-06	-0.0003	0.00051	-3E-05	0.00144	0.00067
8.9E-06	-0.0003	0.00153	-0.0003	0.00087	0.00031
8.9E-06	0.00034	0.00086	-0.0009	0.0016	3.1E-05
8.9E-06	0.00057	0.00127	-0.0003	0.00069	0.00027
9E-06	0.00065	0.00137	-0.0005	0.00141	9.3E-05
9E-06	-0.0005	0.00178	0.00037	0.00065	0.00021
9E-06	-0.0003	0.0007	-0.0002	0.0013	0.00039
9.1E-06	4.9E-05	0.0009	-0.0003	0.00114	0.00046
9.1E-06	-0.0005	0.00072	-0.0005	0.00044	-1E-05
9.1E-06	0.00036	0.00012	0.00029	0.00118	0.00096
9.2E-06	0.00012	0.00126	0.00023	0.00154	5.7E-05
9.2E-06	-0.0002	0.00137	0.00029	0.0008	0.00022

9.2E-06	0.00027	0.00067	-0.0009	0.00126	0.00032
9.2E-06	0.00032	0.00191	6.7E-05	0.00101	-6E-05
9.3E-06	-0.0005	0.00121	0.00018	0.00055	0.00042
9.3E-06	0.00018	0.00138	0.00033	0.0008	0.0001
9.3E-06	0.00051	0.00042	0.00013	0.00081	0.00051
9.4E-06	-6E-05	0.00164	0.00044	0.00041	-0.0003
9.4E-06	0.00016	0.00088	0.00059	0.00133	0.00067
9.4E-06	-3E-05	0.00102	-0.0005	0.00091	-0.0004
9.5E-06	0.00013	0.00081	-0.0004	0.00099	-0.0003
9.5E-06	-0.0005	0.00113	-0.0003	0.00145	-9E-05
9.5E-06	0.00026	0.00124	-0.0005	0.00117	0.00043
9.6E-06	-0.0002	0.0007	-1E-05	0.00134	-6E-05
9.6E-06	-0.0004	0.00099	0.00031	0.00097	0.00052
9.6E-06	0.00023	0.00117	-0.0007	0.00036	0.00029
9.7E-06	0.00028	0.00065	-0.0006	0.00096	0.00022
9.7E-06	0.00033	0.0009	-0.0005	0.00038	0.00089
9.7E-06	0.00014	0.00089	-8E-05	0.00092	0.00052
9.8E-06	0.00029	0.0006	0.00037	0.00101	0.00081
9.8E-06	0.00065	0.00035	-0.0001	0.00066	0.00119
9.8E-06	-0.0002	0.00064	-0.0001	0.00092	0.00073
9.9E-06	-0.0003	0.00098	0.00011	0.00018	0.00032
9.9E-06	0.00042	0.00102	0.00068	0.0013	4.4E-05
9.9E-06	0.00012	0.00149	0.00072	0.00101	-0.0004
1E-05	0.00062	0.00157	-0.0005	0.0012	7.8E-05
1E-05	0.00031	0.00112	0.00071	0.0014	0.00047
1E-05	-0.0003	0.00088	-0.0002	0.00149	0.00055
1E-05	0.00038	0.00132	0.00055	0.00106	0.00015
1E-05	-0.0004	0.00106	-0.0005	0.00166	7.7E-05
1E-05	-1E-05	0.00056	0.00015	0.00143	-0.0002
1E-05	9.5E-05	0.00096	-0.0001	0.00199	0.00015
1E-05	-1E-05	0.00072	-0.0002	0.00244	-5E-05
1E-05	1.6E-05	0.00038	-9E-05	0.00073	0.0009
1E-05	0.00024	0.00138	0.00013	0.00089	0.00069
1E-05	6.3E-05	0.00126	0.00045	0.00144	0.00042
1E-05	0.0004	0.00119	-0.0007	0.00063	0.00032
1E-05	0.00027	0.00117	-0.0002	0.00105	2.8E-06
1E-05	0.00037	0.00148	-0.0007	0.00027	0.00058
1E-05	0.00026	0.00158	0.00017	0.00053	0.00037
1E-05	0.00021	0.00114	-0.0006	0.00134	-0.0001
1E-05	0.0004	0.00133	-0.0004	0.00113	0.00026
1.1E-05	-0.0003	0.00065	0.00051	0.00089	-0.0002
1.1E-05	1.9E-05	0.00148	0.00048	0.00128	0.00048
1.1E-05	0.00079	0.0009	-0.0003	0.0011	-0.0001
1.1E-05	0.00058	0.00058	-0.0001	0.00153	0.00032
1.1E-05	3.2E-05	0.00071	0.00034	0.00025	0.00031
1.1E-05	-0.0006	0.00041	-0.0004	0.00039	-8E-05
1.1E-05	0.00036	0.0005	-0.0012	0.00076	5E-05
1.1E-05	6.8E-05	0.0016	0.00042	0.00232	0.00068
1.1E-05	0.00085	0.00085	0.00016	0.00188	-0.0001
1.1E-05	0.00024	0.00091	2.2E-05	0.00152	-6E-05
1.1E-05	-0.0005	0.00052	0.00022	0.00109	-0.0002
1.1E-05	4.5E-05	0.00106	-0.0003	0.00071	0.00064
1.1E-05	0.00014	0.00106	0.00016	0.00073	0.00037
1.1E-05	-0.0003	0.00061	-0.0004	0.00041	0.00046

1.1E-05	-0.0004	0.00122	-0.0004	0.001	0.00037
1.1E-05	6.9E-05	0.00098	-0.0001	0.00091	8.7E-05
1.1E-05	-0.0008	0.00094	0.00015	0.00082	0.00036
1.1E-05	-6E-06	0.00117	-0.0004	0.00016	0.00073
1.1E-05	-0.0007	0.0009	-8E-05	0.00133	0.00092
1.1E-05	0.00034	0.00019	-9E-05	0.0013	0.00013
1.1E-05	0.00087	0.00061	-0.0002	0.00165	-0.0001
1.1E-05	1E-05	0.00073	0.0008	0.0007	-0.0002
1.1E-05	-0.0004	4.7E-05	-0.0002	0.00078	0.00053
1.1E-05	8.2E-05	0.00049	2E-05	0.00074	0.00036
1.1E-05	8.9E-05	0.00102	-0.0002	0.00085	0.00017
1.1E-05	0.00044	7E-05	-0.0009	0.00161	0.00018
1.1E-05	0.00018	0.0002	0.00039	0.00129	0.00012
1.1E-05	0.00035	0.00066	0.00084	0.0013	0.00013
1.1E-05	0.0002	0.00124	-0.0009	0.00095	0.00025
1.1E-05	0.00024	0.00091	-3E-05	0.00132	-2E-05
1.1E-05	0.00026	0.00054	-0.0006	0.00155	0.00029
1.1E-05	0.00069	0.00033	-0.0005	0.00237	-9E-05
1.2E-05	-0.0002	0.00079	-0.0002	0.00163	0.00041
1.2E-05	-0.0001	0.00103	0.00043	0.00131	1.7E-05
1.2E-05	0.00064	0.00104	0.00091	0.00101	0.00073
1.2E-05	0.00042	4.9E-05	0.00046	0.00191	-0.0004
1.2E-05	7.2E-05	0.00073	-0.0003	0.0012	0.00025
1.2E-05	-0.0003	0.00121	-0.0006	0.0018	0.00013
1.2E-05	0.00018	0.00101	3.1E-06	0.00184	-8E-05
1.2E-05	-0.0001	0.00101	0.00098	0.0012	0.00061
1.2E-05	7.7E-05	0.00087	-0.0006	0.00092	-3E-05
1.2E-05	0.0003	0.00144	-0.0005	0.00153	0.00093
1.2E-05	-0.0003	0.00113	-2E-05	0.00071	0.00032
1.2E-05	5.5E-05	0.00098	0.00011	0.00166	0.00091
1.2E-05	5.9E-05	0.00152	0.00016	0.00078	-0.0002
1.2E-05	-0.0002	0.00159	0.00033	0.00045	5.6E-05
1.2E-05	-0.0002	0.00047	-0.0001	0.00094	0.00066
1.2E-05	0.00064	0.00065	4.5E-05	0.00117	0.00024
1.2E-05	0.00021	0.00135	-0.0003	0.00102	7.9E-05
1.2E-05	-6E-05	0.0006	0.00022	0.00064	9.2E-05
1.2E-05	0.00024	0.00122	0.00031	0.00089	-4E-05
1.2E-05	0.00058	0.00082	0.00036	0.00102	0.00052
1.2E-05	-5E-05	0.00173	-0.0003	0.00118	-0.0001
1.2E-05	-0.0003	0.00091	0.00043	0.0004	-0.0004
1.2E-05	-0.0004	0.00028	-0.001	0.00064	0.00058
1.2E-05	0.00054	0.00108	-0.0009	0.00061	0.00028
1.2E-05	-0.0002	0.00057	-0.0002	0.00123	-0.0002
1.2E-05	0.00076	0.00099	-0.0003	0.00162	-0.0003
1.2E-05	-8E-05	0.0014	0.00015	0.00049	0.00084
1.2E-05	-0.0004	0.00138	-0.0003	0.00115	0.00076
1.2E-05	0.00043	0.001	-0.0005	0.0013	0.00028
1.2E-05	-0.0005	0.00149	0.0002	0.00092	-9E-05
1.2E-05	-0.0004	0.00149	-0.0006	0.00094	-0.0007
1.3E-05	-9E-05	0.00183	-0.0004	0.00105	0.00057
1.3E-05	-0.0003	0.00148	-0.0005	0.00148	0.00053
1.3E-05	0.00012	0.00108	-0.0006	0.00193	-0.0004
1.3E-05	0.00034	0.00073	-0.0011	0.00069	0.00035
1.3E-05	0.00077	0.00157	-0.0009	0.00138	-0.0003

1.3E-05	-0.0001	-9E-05	0.00033	0.00125	0.0001
1.3E-05	0.00022	0.00019	-0.0002	0.00153	0.00058
1.3E-05	0.00096	0.0011	-0.0008	0.00098	0.00012
1.3E-05	0.00057	0.00055	8.8E-05	0.00094	0.00018
1.3E-05	-0.0002	0.00059	-0.0002	0.0011	0.00022
1.3E-05	-0.0009	0.00124	9.4E-05	0.00114	0.00058
1.3E-05	-0.0001	0.00143	-0.0002	0.00033	0.00073
1.3E-05	-1E-05	0.00169	-8E-05	0.0012	2E-05
1.3E-05	-0.0003	0.00155	-0.0004	0.00127	-0.0002
1.3E-05	-0.0002	0.00111	-0.0003	0.0008	0.00033
1.3E-05	0.00024	0.00121	0.00025	0.00081	9.5E-05
1.3E-05	0.00113	0.00084	2.2E-05	0.0008	-5E-05
1.3E-05	-6E-05	0.00046	0.00041	0.00163	0.00031
1.3E-05	-0.0002	0.0003	0.00048	0.00138	0.00013
1.3E-05	0.0008	0.00155	-0.0002	0.00142	0.00015
1.3E-05	0.00067	0.00125	-0.0004	0.00102	0.00022
1.3E-05	0.0002	0.00019	-3E-05	0.00042	0.00043
1.3E-05	0.00037	0.00091	0.00083	0.00127	0.00085
1.3E-05	0.00046	0.00135	-0.0004	0.00151	0.00073
1.3E-05	0.00034	0.00059	-0.0006	0.00063	-0.0004
1.3E-05	-3E-05	0.00171	-0.0005	0.00101	0.00025
1.3E-05	0.00065	0.00075	-0.0001	0.00135	0.00079
1.3E-05	0.00052	0.00033	-0.0005	0.00107	0.00032
1.3E-05	0.00021	0.00067	-0.0004	0.00124	0.00048
1.3E-05	0.00048	0.00067	-0.0007	0.00126	0.0007
1.3E-05	0.00054	0.00094	0.00079	0.00122	0.0002
1.4E-05	0.00089	0.00125	-0.0003	0.00138	-0.0001
1.4E-05	0.00136	0.00118	-0.0005	0.00116	0.00013
1.4E-05	0.00073	0.0009	-0.0007	0.00084	-0.0002
1.4E-05	-0.0002	-0.0001	-0.0013	0.00111	0.00019
1.4E-05	-8E-05	0.00046	0.00027	0.00097	0.00029
1.4E-05	0.00038	0.00063	0.0004	0.00126	0.00056
1.4E-05	0.00036	0.00069	-0.0004	0.00107	5.5E-05
1.4E-05	0.00019	0.00078	0.00019	0.00062	0.0003
1.4E-05	0.00039	0.00084	-0.0003	0.00065	0.00106
1.4E-05	-8E-05	0.00064	-0.0003	0.00115	0.00024
1.4E-05	0.00047	0.00016	3E-05	0.00066	-7E-05
1.4E-05	0.00014	0.002	-0.0006	0.00101	-4E-05
1.4E-05	3.1E-05	0.00044	-0.0007	0.00087	-0.0002
1.4E-05	0.00031	0.00094	0.00047	0.00068	0.00075
1.4E-05	3.9E-05	0.00035	-7E-05	0.00028	0.00076
1.4E-05	0.00095	9.8E-06	-0.0007	0.00066	0.00062
1.4E-05	0.00116	0.00015	0.00019	0.00183	0.00015
1.4E-05	0.00041	0.00087	0.00035	0.00125	5.1E-05
1.4E-05	0.00053	0.00094	-0.0008	0.00085	0.00017
1.4E-05	0.00013	0.00064	0.0002	0.00112	-0.0003
1.4E-05	0.00033	0.00077	-0.0005	0.00081	0.00032
1.4E-05	0.00063	0.00098	6E-05	0.00049	0.00017
1.4E-05	0.00022	0.00141	-0.0004	0.00133	0.00064
1.4E-05	-1E-05	0.00092	-0.0002	0.00023	-8E-05
1.4E-05	-0.0004	0.00089	-4E-06	0.00077	0.00033
1.4E-05	0.00069	0.0019	-0.0004	0.00102	0.00072
1.4E-05	6.2E-05	0.00121	1.6E-05	0.00127	8.2E-05
1.4E-05	-0.0005	0.00084	-0.0003	0.00037	0.00045

1.4E-05	1.1E-05	0.00058	-0.0006	0.00115	0.00028
1.4E-05	0.00078	0.00145	-0.0004	0.00097	0.00027
1.4E-05	0.00095	0.00168	-0.0006	0.00131	0.00021
1.5E-05	0.00048	-1E-05	-0.0009	0.00101	7.7E-05
1.5E-05	0.00041	0.00099	0.00082	0.00053	4.1E-05
1.5E-05	0.00013	0.00157	-0.0008	0.00086	-0.0002
1.5E-05	-0.0007	0.00059	-0.0002	0.00118	-4E-05
1.5E-05	-5E-06	0.00076	-0.0005	0.00107	0.00014
1.5E-05	1.4E-05	0.00068	-0.0004	0.00088	0.00023
1.5E-05	0.00064	0.0007	-0.0004	0.0013	0.00033
1.5E-05	-7E-05	0.00128	0.00059	0.00071	2.1E-05
1.5E-05	-2E-05	0.00166	0.00038	0.0005	-0.0003
1.5E-05	0.00028	0.0017	0.00013	0.00088	-0.0002
1.5E-05	0.0003	0.00057	-9E-05	0.00046	0.00023
1.5E-05	0.00029	0.00074	0.00104	0.00104	6.4E-05
1.5E-05	0.0011	0.00095	0.00021	0.00055	0.00015
1.5E-05	0.00018	0.00082	-0.0004	0.00109	-0.0003
1.5E-05	-0.0004	0.0012	-0.0004	0.00111	-3E-05
1.5E-05	0.00023	0.00133	-0.0006	0.00108	0.00051

Table SI.8: Primary kinetic data for the reaction of 2-piperidinone (**S8**) with CumO$^{\bullet}$ generated by 355 nm LFP in MeCN (**Figure SI.8**).

**t / s**	**ΔA 2-piperidinone (S8)**				
	**0.0807M**	**0.245M**	**0.404M**	**0.598M**	**0.807M**
-1E-06	-5E-06	0.0007	-3E-06	0.00044	0.00068
-1E-06	0.00103	0.00076	-0.0006	9.8E-06	0.00067
-9E-07	0.0014	0.00187	-0.0003	0.00023	0.0007
-9E-07	0.00114	0.00122	0.00032	0.00016	0.00058
-9E-07	0.0011	0.00085	-0.0004	0.00018	0.0007
-8E-07	0.00154	0.00041	-8E-05	0.00037	0.00072
-8E-07	0.00132	0.00045	-0.0009	-0.001	0.00048
-8E-07	0.00079	0.00029	0.00017	0.00078	0.00019
-7E-07	0.00094	0.00103	0.00015	-0.0002	0.00085
-7E-07	0.00035	0.00033	-0.0002	5.8E-05	0.00056
-7E-07	0.00128	0.00024	9.6E-05	0.0005	0.00046
-6E-07	0.00085	-0.0001	-0.0006	4.3E-06	0.00086
-6E-07	0.0019	0.00023	0.00042	-3E-05	0.0005
-6E-07	0.00093	0.00012	-0.0003	-7E-05	9.4E-05
-5E-07	0.00138	0.00096	-9E-05	6.1E-05	0.00025
-5E-07	0.00077	0.00174	-0.0001	0.0006	9.7E-05
-5E-07	0.00092	0.00029	-0.0003	0.0004	0.00068
-4E-07	0.00078	0.00068	-0.0005	0.00043	0.00075
-4E-07	0.00163	0.00057	-0.0007	-0.0002	0.00073
-4E-07	0.00091	0.00071	0.00019	-0.0003	0.00054
-4E-07	0.00133	0.00174	1.1E-05	0.00076	0.00064
-3E-07	0.00076	0.00105	-0.0007	-1E-06	0.00075
-3E-07	0.00056	0.00105	-0.0004	8.7E-05	0.00093
-3E-07	0.00088	0.00064	-0.0009	-0.0003	0.00053
-2E-07	0.00162	0.00123	-0.0002	0.00062	0.00071
-2E-07	0.00142	0.00177	0.00049	0.00066	-6E-05
-2E-07	0.00148	0.00087	-0.0006	-0.0003	-6E-06
-1E-07	0.00077	0.00134	-0.0006	-0.0007	-8E-05
-1E-07	0.00056	5E-05	-0.0003	-0.0002	0.0009
-6E-08	0.00084	8.3E-05	-0.0008	-0.0002	0.00032
-3E-08	0.00108	0.00056	-0.0007	0.00028	1.4E-05
-4E-10	-0.0026	-0.0031	-0.003	-0.0023	-0.0038
3.2E-08	-0.0154	-0.0139	-0.0164	-0.0132	-0.0164
6.4E-08	0.00871	0.0082	0.00814	0.00772	0.00911
9.6E-08	0.01558	0.01728	0.01505	0.01625	0.01839
1.3E-07	0.01569	0.01575	0.0142	0.01433	0.0162
1.6E-07	0.0151	0.01499	0.01243	0.01337	0.01425
1.9E-07	0.01461	0.014	0.01177	0.01222	0.01309
2.2E-07	0.01497	0.01423	0.0117	0.01095	0.01302
2.6E-07	0.01379	0.01275	0.01124	0.01064	0.01121
2.9E-07	0.01426	0.01217	0.01081	0.0089	0.01092
3.2E-07	0.01357	0.01234	0.00957	0.00906	0.00996
3.5E-07	0.01339	0.0119	0.00807	0.00889	0.00859
3.8E-07	0.0123	0.01098	0.00787	0.00816	0.00785
4.2E-07	0.01228	0.01052	0.00783	0.00774	0.00817
4.5E-07	0.01248	0.01019	0.00777	0.00735	0.00709
4.8E-07	0.01105	0.00966	0.0072	0.00608	0.00679
5.1E-07	0.0115	0.00906	0.00698	0.00593	0.00613
5.4E-07	0.01053	0.00911	0.00614	0.00576	0.00645

5.8E-07	0.01088	0.00921	0.00616	0.00514	0.00574
6.1E-07	0.01054	0.00845	0.00581	0.00432	0.00512
6.4E-07	0.01067	0.00743	0.00616	0.00571	0.00514
6.7E-07	0.00993	0.00703	0.00513	0.00432	0.00521
7E-07	0.00963	0.00725	0.00448	0.00475	0.0042
7.4E-07	0.00927	0.00725	0.00461	0.00354	0.00424
7.7E-07	0.00948	0.00685	0.00407	0.00382	0.00446
8E-07	0.0089	0.00673	0.00421	0.00349	0.00421
8.3E-07	0.00867	0.00652	0.00407	0.00369	0.00336
8.6E-07	0.00838	0.00623	0.00347	0.00316	0.00317
9E-07	0.00796	0.00649	0.00248	0.00277	0.00328
9.3E-07	0.00837	0.00503	0.00283	0.00247	0.00331
9.6E-07	0.00735	0.00583	0.00252	0.00223	0.00309
9.9E-07	0.00773	0.00503	0.00229	0.00235	0.0029
1E-06	0.00729	0.00422	0.00201	0.00252	0.00289
1.1E-06	0.00724	0.00484	0.00289	0.00224	0.00336
1.1E-06	0.00735	0.00422	0.00371	0.00303	0.00236
1.1E-06	0.00707	0.00482	0.00219	0.00233	0.0026
1.2E-06	0.00697	0.00401	0.00267	0.00229	0.00226
1.2E-06	0.00675	0.0047	0.00157	0.00149	0.00213
1.2E-06	0.00642	0.00412	0.00178	0.00179	0.00241
1.2E-06	0.00598	0.00448	0.00172	0.00207	0.00212
1.3E-06	0.00573	0.00422	0.00175	0.00186	0.00175
1.3E-06	0.00675	0.00495	0.00194	0.00227	0.00264
1.3E-06	0.00598	0.00358	0.00047	0.00201	0.00167
1.4E-06	0.00626	0.00437	0.00216	0.00166	0.00245
1.4E-06	0.00601	0.00389	0.00044	0.00154	0.00251
1.4E-06	0.00567	0.00379	0.00145	0.00135	0.00242
1.5E-06	0.00691	0.00383	0.00169	0.00091	0.00127
1.5E-06	0.0061	0.00305	0.0009	0.00107	0.00134
1.5E-06	0.0051	0.0033	0.00113	0.00105	0.00217
1.6E-06	0.00511	0.00284	0.00075	0.00106	0.00232
1.6E-06	0.00483	0.00287	0.00076	0.00114	0.00198
1.6E-06	0.00471	0.00274	0.00126	0.00079	0.00181
1.7E-06	0.00466	0.00234	0.00103	0.001	0.00157
1.7E-06	0.00374	0.00313	0.00027	0.00036	0.00224
1.7E-06	0.00485	0.00353	0.00065	0.00075	0.00259
1.8E-06	0.00427	0.00245	0.00076	0.00132	0.00248
1.8E-06	0.00395	0.00297	0.00069	0.00089	0.00172
1.8E-06	0.004	0.00248	0.00048	0.00127	0.00159
1.9E-06	0.00439	0.00271	0.00049	0.00129	0.00157
1.9E-06	0.00371	0.00252	0.0008	0.00149	0.00154
1.9E-06	0.00451	0.00209	0.00104	0.00106	0.0025
2E-06	0.00435	0.00285	0.00034	0.0014	0.00253
2E-06	0.00328	0.00318	0.00056	0.00146	0.00254
2E-06	0.00425	0.00309	0.00112	0.00106	0.00146
2E-06	0.00286	0.00221	0.00112	0.00092	0.00189
2.1E-06	0.00365	0.00233	0.00069	0.00146	0.00177
2.1E-06	0.00266	0.00279	0.0004	0.00072	0.00158
2.1E-06	0.00314	0.00198	0.00089	0.00127	0.00157
2.2E-06	0.00309	0.00143	0.00081	0.00112	0.00168
2.2E-06	0.00377	0.00249	0.00082	0.00189	0.00228
2.2E-06	0.00302	0.00261	0.00031	0.00127	0.00163
2.3E-06	0.00215	0.00207	1.3E-05	0.00126	0.00156

2.3E-06	0.00255	0.002	0.00052	0.00096	0.00298
2.3E-06	0.00299	0.00257	0.00054	0.00112	0.00225
2.4E-06	0.00304	0.00186	0.0004	0.00127	0.00192
2.4E-06	0.00308	0.00239	0.00054	0.00124	0.0019
2.4E-06	0.00275	0.00205	-6E-05	0.00141	0.00236
2.5E-06	0.00332	0.00286	0.00108	0.00104	0.00183
2.5E-06	0.0025	0.00253	-6E-05	0.00168	0.00181
2.5E-06	0.00267	0.0017	0.00066	0.00088	0.0022
2.6E-06	0.00308	0.00183	-1E-05	0.00067	0.00219
2.6E-06	0.0031	0.00181	0.00043	0.00034	0.00163
2.6E-06	0.00243	0.00213	0.00066	0.00156	0.00191
2.7E-06	0.00241	0.00199	0.00083	0.00117	0.00111
2.7E-06	0.00318	0.00196	0.00011	0.00098	0.00204
2.7E-06	0.00245	0.00149	0.00041	0.00126	0.00243
2.8E-06	0.00271	0.00198	-7E-05	0.00177	0.00138
2.8E-06	0.00228	0.0014	5.8E-06	0.00164	0.00169
2.8E-06	0.00223	0.00166	9.4E-05	0.00143	0.00204
2.8E-06	0.00228	0.00194	0.00054	0.00132	0.00153
2.9E-06	0.00293	0.00184	0.0006	0.00149	0.00184
2.9E-06	0.00237	0.0018	0.00016	0.0014	0.00179
2.9E-06	0.00212	0.00144	-0.0004	0.00098	0.00159
3E-06	0.00264	0.00185	-0.0001	0.00095	0.00064
3E-06	0.00215	0.00166	-3E-05	0.00078	0.00193
3E-06	0.00235	0.00144	0.00017	0.00065	0.00197
3.1E-06	0.00258	0.00216	-0.0003	0.00078	0.00125
3.1E-06	0.00248	0.00141	0.00052	0.0007	0.00152
3.1E-06	0.0024	0.0019	-0.0004	0.00123	0.00134
3.2E-06	0.00234	0.00204	0.00018	0.0006	0.00049
3.2E-06	0.00196	0.00131	0.00058	0.00123	0.00092
3.2E-06	0.00233	0.00155	2.7E-05	0.00097	0.00147
3.3E-06	0.002	0.00166	-0.0004	0.00102	0.00085
3.3E-06	0.00277	0.00087	-0.0002	0.00073	0.00104
3.3E-06	0.00257	0.00161	-1E-04	0.00045	0.00123
3.4E-06	0.00273	0.00125	-0.0001	0.00133	0.00203
3.4E-06	0.00204	0.00122	0.00025	0.00096	0.00141
3.4E-06	0.0024	0.00194	-0.0004	0.00028	0.00156
3.5E-06	0.00161	0.00245	0.00081	0.00127	0.00155
3.5E-06	0.00191	0.00069	-0.0003	0.00127	0.00099
3.5E-06	0.00208	0.00154	-0.0005	0.00064	0.00114
3.6E-06	0.00175	0.00142	-4E-05	-9E-05	0.00128
3.6E-06	0.00217	0.00152	-0.0003	0.00041	0.00062
3.6E-06	0.00177	0.00155	-0.0002	0.00067	0.00158
3.6E-06	0.00185	0.002	0.00023	0.00093	0.00085
3.7E-06	0.0017	0.00103	7.4E-05	0.00069	0.00085
3.7E-06	0.00217	0.00181	-0.0004	0.00086	0.00235
3.7E-06	0.0018	0.00192	0.00096	0.00101	0.00205
3.8E-06	0.00267	0.00141	-9E-05	0.00072	0.0014
3.8E-06	0.00168	0.00161	-0.0001	0.00092	0.00222
3.8E-06	0.00182	0.0012	1.5E-05	0.00125	0.00192
3.9E-06	0.0019	0.00098	0.00081	0.0009	0.00141
3.9E-06	0.00158	0.00082	0.00038	0.00048	0.00088
3.9E-06	0.0022	0.00113	0.00019	0.00113	0.00119
4E-06	0.00191	0.00116	0.00064	0.00139	0.00249
4E-06	0.00155	0.00111	0.00018	0.00058	0.00069

4E-06	0.00222	0.0012	7.5E-05	0.00178	0.00157
4.1E-06	0.00215	0.00047	-0.0003	0.00135	0.00138
4.1E-06	0.00178	0.00146	0.00016	0.00119	0.00152
4.1E-06	0.00181	0.00221	0.00031	0.00095	0.00124
4.2E-06	0.00186	0.00195	-0.0002	0.00069	0.00173
4.2E-06	0.00203	0.00163	0.00053	-2E-06	0.00133
4.2E-06	0.00047	0.00138	1E-05	0.00104	0.00097
4.3E-06	0.0019	0.00119	0.00053	0.00149	0.00122
4.3E-06	0.00195	0.00205	-0.0006	0.00108	0.00109
4.3E-06	0.0022	0.00078	0.00097	0.00064	0.00184
4.4E-06	0.00243	0.00137	4.1E-05	0.00062	0.00139
4.4E-06	0.00055	0.00128	0.00025	0.00102	0.00222
4.4E-06	0.0014	0.00121	0.00048	0.00029	0.0017
4.4E-06	0.00201	0.0017	0.00031	0.00099	0.00177
4.5E-06	0.00232	0.00123	-9E-05	0.00054	0.00128
4.5E-06	0.00201	0.00174	6.9E-06	0.00149	0.00175
4.5E-06	0.00208	0.00174	-0.0002	0.00123	0.00161
4.6E-06	0.00194	0.00102	-0.0007	0.00076	0.00162
4.6E-06	0.00184	0.00146	-0.0002	0.00067	0.00061
4.6E-06	0.00152	0.00128	0.00024	0.00179	0.00151
4.7E-06	0.00144	0.00153	-0.0002	0.00121	0.00127
4.7E-06	0.00216	0.00156	0.00039	0.00052	0.00142
4.7E-06	0.0013	0.0016	5.7E-05	0.00099	0.00165
4.8E-06	0.00214	0.00143	0.00097	0.00095	0.00109
4.8E-06	0.00087	0.00172	0.00089	0.00112	0.00027
4.8E-06	0.00149	0.00153	0.00037	0.00055	0.00051
4.9E-06	0.00125	0.00137	-0.0003	0.00078	0.00118
4.9E-06	0.00103	0.00174	6.9E-05	0.0009	0.0012
4.9E-06	0.00035	0.00191	-2E-05	0.00117	0.0011
5E-06	0.00116	0.00183	0.00024	0.00155	-2E-05
5E-06	0.00135	0.00134	0.00066	0.00115	0.00087
5E-06	0.00083	0.00174	4.2E-05	0.00054	0.00112
5.1E-06	0.00143	0.00164	-0.0004	0.00096	0.0007
5.1E-06	0.00251	0.00225	-0.0003	0.00125	0.00129
5.1E-06	0.00177	0.001	-0.0003	0.00082	0.00169
5.2E-06	0.00159	0.00165	8.7E-05	0.00093	0.00122
5.2E-06	0.00156	0.00136	-5E-05	0.00097	0.00181
5.2E-06	0.00086	0.00195	0.00042	0.00121	0.00115
5.2E-06	0.00157	0.00185	0.00017	0.00168	0.00076
5.3E-06	0.001	0.00171	0.00057	0.001	0.00128
5.3E-06	0.00153	0.00124	-0.0007	0.00068	0.00059
5.3E-06	0.00175	0.00134	0.0008	0.00159	0.00163
5.4E-06	0.00137	0.0019	5.4E-05	0.00105	0.00095
5.4E-06	0.00109	0.00088	-0.0001	0.00036	0.00095
5.4E-06	0.00182	0.00146	-0.0004	0.00107	0.00097
5.5E-06	0.00102	0.00132	-0.0005	0.00137	0.00138
5.5E-06	0.00116	0.00142	-0.0005	0.00081	0.00187
5.5E-06	0.00149	0.00125	-6E-05	0.00086	0.00144
5.6E-06	0.00184	0.00154	0.00051	0.00029	0.00134
5.6E-06	0.00152	0.00117	0.00076	0.00076	0.00095
5.6E-06	0.00211	0.00146	0.00013	0.00143	0.00154
5.7E-06	0.00133	0.0019	-0.0002	0.00155	0.00024
5.7E-06	0.00185	0.00136	0.00034	0.00072	0.00082
5.7E-06	0.00118	0.00147	-0.0001	0.00164	0.00131

5.8E-06	0.00199	0.00201	-0.0001	0.00147	0.00081
5.8E-06	0.00227	0.00167	4.8E-05	0.00121	0.00095
5.8E-06	0.00132	0.00187	0.00066	0.00193	0.00099
5.9E-06	0.00167	0.00043	0.00061	-0.0001	0.00075
5.9E-06	0.00123	0.0019	-0.0002	0.00103	0.00093
5.9E-06	0.00179	0.00131	-0.0002	0.00077	0.00125
6E-06	0.00117	0.00174	-0.0004	0.00128	0.00078
6E-06	0.00212	0.00119	-0.0004	0.00106	0.0005
6E-06	0.00157	0.00141	0.00022	0.00129	0.00076
6E-06	0.002	0.00226	-6E-06	0.00109	0.00085
6.1E-06	0.00131	0.00169	0.00033	0.00114	0.00131
6.1E-06	0.00133	0.00157	-0.0005	4.9E-05	0.00145
6.1E-06	0.00199	0.00127	0.00013	0.00025	0.00146
6.2E-06	0.00054	0.00162	-0.0001	0.0013	0.00117
6.2E-06	0.00217	0.00166	0.00024	0.00132	0.00176
6.2E-06	0.00147	0.00203	0.00039	0.00022	0.00151
6.3E-06	0.00166	0.00171	-0.0006	0.00133	0.00097
6.3E-06	0.00073	0.00093	-0.0008	0.00092	5.7E-05
6.3E-06	0.0018	0.00081	-0.0006	0.00139	0.00113
6.4E-06	0.0018	0.00213	0.00022	0.00125	0.00147
6.4E-06	0.00173	0.001	0.00027	0.00103	0.00133
6.4E-06	0.0012	0.00133	-0.0001	0.00105	0.00144
6.5E-06	0.00208	0.00142	0.00035	0.00081	0.00094
6.5E-06	0.00193	0.00148	0.00097	0.0011	0.00176
6.5E-06	0.00157	0.00144	0.00084	0.00111	0.00101
6.6E-06	0.00202	0.00132	-0.0002	0.00119	0.0015
6.6E-06	0.00115	0.00187	-0.0006	0.0014	0.001
6.6E-06	0.00212	0.00161	0.00024	0.00168	0.00076
6.7E-06	0.00115	0.00124	0.00023	0.00151	0.00173
6.7E-06	0.00162	0.00035	0.00066	0.00059	0.00117
6.7E-06	0.00162	0.00135	0.00041	0.00107	0.00085
6.8E-06	0.00215	0.00133	0.00037	0.00116	0.00104
6.8E-06	0.00173	0.00118	-5E-05	0.00099	0.00099
6.8E-06	0.00124	0.00196	-0.0002	0.00128	0.00063
6.8E-06	0.00099	0.00196	0.00021	0.001	0.00118
6.9E-06	0.00129	0.00071	-0.0001	0.00131	0.00152
6.9E-06	0.00114	0.00152	0.00078	0.0009	0.00098
6.9E-06	0.00149	0.00168	0.00058	0.00153	0.0004
7E-06	0.00153	0.00141	1.7E-05	0.00137	0.00058
7E-06	0.00097	0.00147	-2E-06	0.00137	0.00011
7E-06	0.00224	0.0018	-0.0003	0.00108	0.00146
7.1E-06	0.00109	0.00148	-1E-04	0.0011	0.00154
7.1E-06	0.00209	0.00108	-1E-04	0.00113	0.00088
7.1E-06	0.00145	0.00159	0.00029	0.00032	0.00106
7.2E-06	0.00154	0.00158	-0.0002	0.0008	0.00228
7.2E-06	0.00184	0.00154	0.00019	0.00114	0.00013
7.2E-06	0.00159	0.00114	0.00084	0.0019	0.00089
7.3E-06	0.00121	0.00202	9.1E-05	0.00192	0.00044
7.3E-06	0.00198	0.00138	-0.0005	0.00108	0.00089
7.3E-06	0.00148	0.00136	-0.0007	0.00177	0.00042
7.4E-06	0.0021	0.00085	-0.0009	0.00165	0.00098
7.4E-06	0.00199	0.00198	-0.0002	0.00136	0.0013
7.4E-06	0.00182	0.00108	-0.0002	0.00026	0.00112
7.5E-06	0.00152	0.0014	-0.0001	0.00075	0.0014

7.5E-06	0.00097	0.0018	-0.0002	0.00182	0.00138
7.5E-06	0.00126	0.00188	-0.0006	0.00141	0.00014
7.6E-06	0.001	0.00139	6E-05	0.00163	0.00015
7.6E-06	0.0007	0.00152	5.2E-05	0.00106	0.00059
7.6E-06	0.00157	0.00302	9.2E-05	0.00064	0.00129
7.6E-06	0.00151	0.00105	0.00018	0.00122	0.00102
7.7E-06	0.00193	0.00143	-0.0004	0.00133	0.00025
7.7E-06	0.00192	0.00162	0.00022	0.00083	0.001
7.7E-06	0.00161	0.00176	0.00024	0.00142	8.9E-05
7.8E-06	0.00169	0.00223	-0.0002	0.00153	0.00089
7.8E-06	0.00099	0.00204	0.00017	0.00126	0.00097
7.8E-06	0.0011	0.00178	-0.0003	0.0019	0.00105
7.9E-06	0.00092	0.00175	9.3E-06	0.00138	0.00031
7.9E-06	0.00142	0.00167	0.00036	0.00155	0.00101
7.9E-06	0.0017	0.00248	6.8E-05	0.00144	0.00036
8E-06	0.00164	0.00134	-0.0006	0.00142	0.0012
8E-06	0.00154	0.00172	3E-05	0.00155	0.00061
8E-06	0.00136	0.0012	0.0004	0.00139	0.00131
8.1E-06	0.00114	0.0014	6.5E-05	0.0015	0.00107
8.1E-06	0.00216	0.0012	-5E-05	0.00077	0.00087
8.1E-06	0.00165	0.00179	-0.0004	0.00135	0.00113
8.2E-06	0.0012	0.00106	0.00037	0.0014	0.00127
8.2E-06	0.00104	0.00214	1.2E-05	0.00082	0.00055
8.2E-06	0.00141	0.00179	7.8E-05	0.00128	0.00126
8.3E-06	0.00113	0.00122	-0.0002	0.00073	0.00148
8.3E-06	0.00148	0.00181	-0.0001	0.00155	0.0006
8.3E-06	0.00088	0.00179	-0.0001	0.00058	0.00099
8.4E-06	0.00152	0.00154	-7E-05	0.00085	0.00079
8.4E-06	0.00163	0.00169	-0.0007	0.00034	0.00056
8.4E-06	0.00194	0.00147	3.8E-05	0.00093	0.0007
8.4E-06	0.00119	0.00129	0.00047	0.001	0.00102
8.5E-06	0.00168	0.00197	-7E-05	0.00147	0.00067
8.5E-06	0.00154	0.0014	0.00022	0.00104	0.00108
8.5E-06	0.00134	0.00164	-0.0004	0.00147	0.00089
8.6E-06	0.00166	0.00186	-0.0002	0.00097	0.00104
8.6E-06	0.00132	0.0019	-6E-05	0.00093	0.0008
8.6E-06	0.00176	0.00133	0.00051	0.0012	0.00091
8.7E-06	0.00177	0.00174	2.5E-05	0.00121	0.00039
8.7E-06	0.00168	0.00162	8.7E-05	0.0013	0.00135
8.7E-06	0.00143	0.00153	-2E-05	0.00097	0.00086
8.8E-06	0.0012	0.00167	-0.0004	0.00105	0.00145
8.8E-06	0.0015	0.0011	-5E-05	0.00087	0.00073
8.8E-06	0.0017	0.00162	-8E-05	0.00083	0.00051
8.9E-06	0.0017	0.00104	7.7E-05	0.00124	0.00112
8.9E-06	0.00091	0.00087	-0.0002	0.00119	0.0011
8.9E-06	0.00148	0.002	-0.0002	0.00103	3.1E-05
9E-06	0.00069	0.00117	-0.0007	0.00062	-0.0002
9E-06	0.00175	0.00176	0.00038	0.00108	0.00086
9E-06	0.00197	0.002	0.00015	0.00075	0.00118
9.1E-06	0.00264	0.00186	-0.0003	0.00141	0.0013
9.1E-06	0.00167	0.00184	-0.0002	0.00124	0.00071
9.1E-06	0.00066	0.0017	-0.0004	0.00159	0.00066
9.2E-06	0.00132	0.00201	-0.0004	0.00142	0.00053
9.2E-06	0.00133	0.00186	-0.0004	0.00142	0.0013

9.2E-06	0.00077	0.00154	-0.0002	0.00086	0.00096
9.2E-06	0.00144	0.00179	-0.0002	0.00136	0.00048
9.3E-06	0.00099	0.00165	-0.0006	0.00104	0.00063
9.3E-06	0.0013	0.00183	-0.0002	0.00145	0.00122
9.3E-06	0.00221	0.00127	-0.0005	0.001	0.00023
9.4E-06	0.00192	0.00126	0.0006	0.00101	0.00016
9.4E-06	0.0017	0.00126	4.1E-06	0.00153	0.00058
9.4E-06	0.0014	0.00156	0.00075	0.00065	-0.0001
9.5E-06	0.00118	0.00212	8.3E-05	0.00072	0.00132
9.5E-06	0.00156	0.00102	-0.0006	0.00097	0.00106
9.5E-06	0.00157	0.00251	0.00011	0.00132	0.00154
9.6E-06	0.00112	0.00179	-0.0005	0.00097	0.00132
9.6E-06	0.00147	0.00135	-7E-05	0.00097	0.00106
9.6E-06	0.00182	0.0014	0.00029	0.00019	0.00059
9.7E-06	0.00212	0.00163	-0.0003	0.00051	0.00088
9.7E-06	0.00138	0.00158	-0.0007	0.00059	0.00098
9.7E-06	0.00182	0.00183	0.00013	0.0004	0.00048
9.8E-06	0.00205	0.00191	-9E-06	0.00081	0.00045
9.8E-06	0.00116	0.00124	0.00031	0.00025	0.00092
9.8E-06	0.00159	0.00133	-0.0003	0.00133	0.00099
9.9E-06	0.00171	0.00186	-0.0007	0.00114	0.00041
9.9E-06	0.0015	0.0016	-0.0007	0.00094	0.00085
9.9E-06	0.002	0.0018	-0.0002	0.00085	0.00131
1E-05	0.00173	0.00108	-0.0002	0.00093	0.00092
1E-05	0.00097	0.0017	-0.0002	0.00125	0.00029
1E-05	0.00123	0.00235	0.00014	0.00216	0.00041
1E-05	0.00128	0.00183	0.00023	0.00088	0.00161
1E-05	0.00054	0.00118	-0.0006	0.00032	-7E-06
1E-05	0.00177	0.00168	-0.0006	0.00177	0.00091
1E-05	0.00152	0.00184	0.00027	0.00124	0.00086
1E-05	0.00138	0.00109	-0.0002	0.00139	0.00041
1E-05	0.00041	0.00236	-0.0003	0.00151	0.00117
1E-05	0.00163	0.00103	-0.0009	0.00061	0.00084
1E-05	0.001	0.00093	-0.0003	0.00135	0.0008
1E-05	0.00107	0.00268	-0.0005	0.00135	0.00049
1E-05	0.00147	0.00218	-9E-05	0.00162	0.0007
1E-05	0.002	0.00092	-0.0001	0.00189	0.0009
1E-05	0.00081	0.0015	-2E-05	0.00122	0.00118
1E-05	0.00117	0.002	0.00016	0.0013	0.00053
1E-05	0.00053	0.00158	-0.0005	0.00124	0.00114
1.1E-05	0.00126	0.00097	-0.0003	0.00055	0.0013
1.1E-05	0.00049	0.00127	-0.0004	0.00124	0.00099
1.1E-05	0.00163	0.00136	-0.0007	0.00117	0.00137
1.1E-05	0.00069	0.00121	-0.0006	0.00136	0.00123
1.1E-05	0.0012	0.00129	-0.0008	0.0012	0.00112
1.1E-05	0.00178	0.00128	-0.0003	0.00114	0.00111
1.1E-05	0.00125	0.00142	-0.0008	0.00101	0.00059
1.1E-05	0.00161	0.00204	-0.0005	0.00207	9E-05
1.1E-05	0.00222	0.00127	-0.0002	0.00057	0.00075
1.1E-05	0.00255	0.00085	0.00054	0.00132	0.00025
1.1E-05	0.00089	0.00108	0.00011	0.00024	0.00044
1.1E-05	0.00136	0.00137	4.4E-05	0.00044	9.5E-05
1.1E-05	0.00172	0.00107	-0.001	0.00183	0.00105
1.1E-05	0.00155	0.00196	0.00027	0.00143	0.00107

1.1E-05	0.00164	0.00116	-0.0006	0.00147	0.00065
1.1E-05	0.00131	0.00166	-0.0002	0.00165	0.00119
1.1E-05	0.00192	0.00149	-0.0002	0.00082	0.0008
1.1E-05	0.00202	0.0011	-0.0003	0.00159	0.00073
1.1E-05	0.00182	0.00174	-0.0006	0.00209	0.00098
1.1E-05	0.00143	0.00092	-0.0006	0.0017	0.00086
1.1E-05	0.00164	0.00107	-0.001	0.00126	0.00061
1.1E-05	0.00134	0.00109	-0.0005	0.00155	0.00131
1.1E-05	0.00137	0.00147	-0.0003	0.00077	0.00063
1.1E-05	0.00164	0.00102	0.00064	0.00022	0.00056
1.1E-05	0.00116	0.00155	-0.0009	0.00072	-2E-05
1.1E-05	0.00145	0.00096	-0.0002	0.00151	0.00083
1.1E-05	0.00096	0.00136	-0.0002	0.00106	-0.0001
1.1E-05	0.00128	0.00188	-3E-05	0.00189	0.00056
1.1E-05	0.00091	0.00191	-0.0004	0.00032	0.00067
1.1E-05	0.00091	0.00129	-0.0004	0.00112	0.00046
1.1E-05	0.00135	0.00109	-0.0003	0.00147	0.00038
1.1E-05	0.00179	0.00166	-0.0006	0.0013	-5E-05
1.2E-05	0.00103	0.00194	-0.0005	0.00112	0.00049
1.2E-05	0.00117	0.00182	-0.0008	0.0012	0.0005
1.2E-05	0.00177	0.00108	-0.0001	0.00161	-5E-06
1.2E-05	0.00165	0.00149	-3E-05	0.00108	3.1E-05
1.2E-05	0.00101	0.00173	-0.0005	0.00087	0.00033
1.2E-05	0.00079	0.00164	0.00048	0.00116	0.00056
1.2E-05	0.00125	0.00154	0.00048	0.00249	0.00078
1.2E-05	0.00216	0.00085	0.0003	0.0011	-0.0004
1.2E-05	0.00154	0.00184	-0.0004	0.00091	-0.0003
1.2E-05	0.00125	0.00248	-0.0001	0.00042	0.00043
1.2E-05	0.0011	0.00164	-0.0006	0.00137	0.00017
1.2E-05	0.00143	0.00154	-0.0005	0.00071	0.00032
1.2E-05	0.00097	0.00174	-0.0011	0.00127	0.00096
1.2E-05	0.00166	0.00135	-0.0003	0.00035	0.00077
1.2E-05	0.00163	0.00144	-7E-05	0.00114	0.0009
1.2E-05	0.00158	0.00116	-0.0009	0.00193	0.00111
1.2E-05	0.00123	0.00123	9.7E-05	0.00208	0.00092
1.2E-05	0.00056	0.00202	-0.0008	0.00132	0.00109
1.2E-05	0.00132	0.00163	0.00023	0.00139	0.00047
1.2E-05	0.00116	0.0011	-0.0004	0.00058	0.00084
1.2E-05	0.00075	0.00235	-0.0002	0.00128	0.00084
1.2E-05	0.00095	0.00247	0.00027	0.00122	0.00034
1.2E-05	0.00019	0.00212	0.00044	0.00081	0.00077
1.2E-05	9.3E-05	0.00172	7.6E-05	0.00214	0.00051
1.2E-05	0.00133	0.0011	2.4E-05	0.00151	0.00067
1.2E-05	0.0008	0.00128	2.2E-05	0.00064	0.00038
1.2E-05	0.00134	0.00231	-0.0008	0.00125	0.0004
1.2E-05	0.00081	0.00199	-0.0003	0.00165	0.00068
1.2E-05	0.00117	0.00148	-0.0003	0.00091	0.00016
1.2E-05	0.00181	0.00135	-0.0002	0.00136	0.00111
1.2E-05	0.00072	0.00171	-0.0011	0.00101	0.00043
1.3E-05	0.00103	0.00163	3E-05	0.00118	0.00012
1.3E-05	0.00129	0.00109	0.0005	0.00116	0.0004
1.3E-05	0.00068	0.00201	5.5E-05	0.00188	0.00099
1.3E-05	0.00105	0.00133	-0.0003	0.00135	0.00043
1.3E-05	0.00153	0.00109	-0.0003	0.00143	0.00024

1.3E-05	0.00146	0.00109	-0.0007	0.00143	0.00071
1.3E-05	0.00135	0.00185	-0.0008	0.00051	0.00027
1.3E-05	0.00061	0.0012	-0.0009	0.00193	0.00073
1.3E-05	0.00127	0.0023	3E-05	0.00173	0.00098
1.3E-05	0.00175	0.00171	-0.0004	0.00136	-0.0002
1.3E-05	0.00117	0.00222	-0.0003	0.00145	0.00021
1.3E-05	0.00119	0.001	0.00056	0.00101	0.00071
1.3E-05	0.00132	0.00155	-0.0002	0.00185	0.00058
1.3E-05	0.00183	0.00127	3.6E-05	0.00124	0.00118
1.3E-05	0.00149	0.00134	-0.0003	0.00156	0.00047
1.3E-05	0.00121	0.00164	-0.0001	0.00132	0.00092
1.3E-05	0.00163	0.00161	0.00062	0.00109	0.00175
1.3E-05	0.00218	0.00128	-0.0003	0.00071	0.00098
1.3E-05	0.00114	0.00163	-0.0007	0.00117	0.00065
1.3E-05	0.00118	0.00146	0.00016	0.00145	0.00032
1.3E-05	0.0012	0.00129	3.7E-05	0.00018	0.00087
1.3E-05	0.00107	0.00128	-0.0004	0.00125	-6E-05
1.3E-05	0.00115	0.00171	-0.0007	0.00144	0.00051
1.3E-05	0.00187	0.00176	2.5E-05	0.00184	0.00087
1.3E-05	0.00152	0.0011	-0.0002	0.00124	0.00058
1.3E-05	0.00113	0.00202	-0.0002	0.00115	0.00093
1.3E-05	0.00073	0.00115	-0.001	0.00095	0.00083
1.3E-05	0.00132	0.00085	-0.0009	0.00144	-0.0004
1.3E-05	0.00118	0.00185	0.0003	0.00101	0.00029
1.3E-05	0.00131	0.00176	5.9E-05	0.00059	0.00031
1.3E-05	0.00112	0.00166	0.00024	0.00133	6.3E-05
1.4E-05	0.00094	0.00135	-0.0006	0.00162	0.00045
1.4E-05	0.00048	0.00168	-0.0002	0.00102	0.00045
1.4E-05	0.0012	0.00203	-0.0002	0.0016	0.00126
1.4E-05	0.00187	0.00244	-0.0002	0.00127	0.00035
1.4E-05	0.00161	0.00154	-0.0004	0.00099	0.00081
1.4E-05	0.00161	0.00149	2.4E-07	0.00196	0.00063
1.4E-05	0.00214	0.00154	3.1E-05	0.00088	0.00077
1.4E-05	0.00049	0.00197	-0.0005	0.00103	0.00086
1.4E-05	0.00103	0.0007	-0.0004	0.00063	0.001
1.4E-05	0.00117	0.00194	-0.0008	0.00087	0.00021
1.4E-05	0.00119	0.00205	-0.0007	0.00047	0.00036
1.4E-05	0.0016	0.00158	-0.0003	0.00114	0.0009
1.4E-05	0.00137	0.00163	-4E-05	0.00158	0.00029
1.4E-05	0.00091	0.0016	0.00066	0.00135	0.00022
1.4E-05	0.00141	0.00157	0.00055	0.0009	0.00106
1.4E-05	0.00144	0.00121	-0.0002	0.00095	2.1E-05
1.4E-05	0.00077	0.0011	-0.0006	0.0015	0.00048
1.4E-05	0.00068	0.00084	0.0005	0.00094	0.001
1.4E-05	0.00168	0.00132	-0.0006	0.00129	0.00088
1.4E-05	0.00071	0.00091	0.00016	0.00085	0.00131
1.4E-05	0.00172	0.00149	0.00062	0.00088	0.00055
1.4E-05	0.00202	0.00205	0.00089	0.0009	0.00107
1.4E-05	0.00189	0.00189	-0.0007	0.00105	0.00072
1.4E-05	0.00146	0.00106	-0.0007	0.0015	0.00114
1.4E-05	0.00176	0.0015	-0.0007	0.00153	0.0006
1.4E-05	0.00162	0.00129	4.9E-06	0.00075	0.00063
1.4E-05	0.00051	0.00216	-0.0004	0.00071	0.00033
1.4E-05	0.00135	0.00086	0.00025	0.00197	2.8E-05

1.4E-05	0.00113	0.00116	-0.0005	0.00144	0.00064
1.4E-05	0.00162	0.00095	-0.0005	0.00184	0.00075
1.4E-05	0.00159	0.00205	0.00016	0.00114	0.00142
1.5E-05	0.00052	0.00115	-0.0003	0.00063	0.00139
1.5E-05	0.00223	0.00119	-0.0005	0.0012	0.00109
1.5E-05	0.00134	0.00112	-0.0004	0.00155	0.00041
1.5E-05	0.002	0.00179	0.00049	0.00096	0.00099
1.5E-05	0.00151	0.00121	-0.0003	0.00093	0.00101
1.5E-05	0.00148	0.0014	-0.001	0.00131	0.00126
1.5E-05	0.00097	0.0015	8.6E-05	0.00157	-0.0007
1.5E-05	0.00174	0.00134	-3E-05	0.001	0.00046
1.5E-05	0.00143	0.00097	0.00032	0.00103	-0.0006
1.5E-05	0.00059	0.00187	-0.001	0.00068	0.00068
1.5E-05	0.00137	0.00135	-0.0004	0.00139	0.00091
1.5E-05	0.00127	0.00177	-0.0002	0.00068	0.00095
1.5E-05	0.00122	0.00134	0.00015	0.00129	-0.0005
1.5E-05	0.00112	0.00153	-0.0008	0.00108	0.0008
1.5E-05	0.00109	0.00183	-0.001	0.00116	0.00026
1.5E-05	0.00151	0.002	-0.0001	0.00067	0.00012

Table SI.9: Primary kinetic data for the reaction of 1-methyl-2-piperidinone (**S9**) with CumO• generated by 355 nm LFP in MeCN (**Figure SI.9**).

**t / s**	**ΔA 1-methyl-2-piperidinone (S9)**				
	**0.044M**	**0.109M**	**0.215M**	**0.319M**	**0.421M**
-1E-06	-0.0014	-0.0002	-0.0002	-0.0005	0.00059
-1E-06	-0.0008	-0.0005	0.00112	-0.0003	0.00012
-9E-07	-0.0005	-0.0004	0.00011	-0.0006	0.00035
-9E-07	-0.0005	0.00053	0.00076	-0.0008	0.00035
-9E-07	-0.0011	-0.0005	0.00051	-0.0014	-0.0002
-8E-07	-0.0007	3.9E-05	0.00067	-0.0006	-0.0003
-8E-07	-0.0011	0.00071	0.00012	-0.0009	-0.0001
-8E-07	-0.0007	-0.0008	0.00091	-0.0002	0.00049
-7E-07	-0.0009	0.0006	0.00104	-0.0009	1.1E-05
-7E-07	-0.0012	0.00031	0.00084	-0.0004	-0.0003
-7E-07	-0.0009	-0.0002	0.00082	-0.001	6E-06
-6E-07	-0.0012	0.0007	0.00091	-0.0014	0.0015
-6E-07	-0.0008	-0.0002	0.00163	-0.0002	-2E-05
-6E-07	-0.001	0.0007	0.0005	-0.0008	-3E-05
-5E-07	-0.0007	0.00013	0.00036	-0.001	0.00038
-5E-07	-0.0018	6E-05	0.00129	-0.0009	0.00011
-5E-07	-0.0012	-0.0003	0.00093	-0.0007	0.00061
-4E-07	-0.0009	0.00094	0.00076	-0.0004	-0.0008
-4E-07	-0.0011	-0.0001	0.00022	-0.0006	-0.0004
-4E-07	-0.0006	-0.0002	-0.0001	-0.0008	0.00056
-4E-07	-0.0013	-0.0003	0.00028	-0.0015	1.9E-05
-3E-07	-0.0011	0.00028	0.00081	-0.0012	0.00046
-3E-07	-0.0013	0.00014	0.0002	-0.0009	-0.0005
-3E-07	-0.0013	-0.0006	0.00075	-0.0014	0.00039
-2E-07	-0.0011	0.00016	0.00082	-0.0011	0.00037
-2E-07	-0.0007	0.00011	0.00059	0.00022	0.00114
-2E-07	-0.0002	-0.0006	0.0011	-0.0009	0.00038
-1E-07	-0.0009	7.7E-05	0.00023	-5E-05	0.00037
-1E-07	-0.0009	-0.0006	0.00028	0.00019	3E-05
-6E-08	-0.0004	0.00028	0.00111	-0.0009	0.00042
-3E-08	-0.001	0.00077	0.00056	-0.0013	0.00043
-2E-10	-0.0035	-0.0034	-0.0029	-0.0038	-0.0041
3.2E-08	-0.0092	-0.011	-0.0183	-0.0171	-0.0193
6.4E-08	0.01844	0.01818	0.01435	0.01174	0.00856
9.6E-08	0.02018	0.02386	0.02304	0.02239	0.02148
1.3E-07	0.01962	0.0221	0.02287	0.02121	0.02208
1.6E-07	0.01897	0.02114	0.02108	0.02039	0.02003
1.9E-07	0.01816	0.02059	0.02015	0.01873	0.01875
2.2E-07	0.01795	0.021	0.02016	0.01846	0.01749
2.6E-07	0.01662	0.01851	0.01832	0.01667	0.01628
2.9E-07	0.01637	0.01796	0.01842	0.01557	0.01527
3.2E-07	0.01573	0.01857	0.01709	0.01426	0.01367
3.5E-07	0.01546	0.01748	0.01598	0.01303	0.01358
3.8E-07	0.01444	0.01574	0.01469	0.01292	0.01269
4.2E-07	0.0141	0.01573	0.01425	0.01299	0.0112
4.5E-07	0.01379	0.01507	0.01387	0.01107	0.01096
4.8E-07	0.01391	0.01445	0.01255	0.01085	0.01011
5.1E-07	0.01251	0.01326	0.01304	0.01079	0.00885
5.4E-07	0.01291	0.01387	0.01112	0.01006	0.00913

5.8E-07	0.01232	0.01277	0.01104	0.0089	0.00822
6.1E-07	0.01128	0.01169	0.01016	0.00772	0.00785
6.4E-07	0.01131	0.0114	0.00993	0.00801	0.00778
6.7E-07	0.01103	0.01126	0.00985	0.00708	0.00697
7E-07	0.01085	0.01068	0.00858	0.00691	0.00852
7.4E-07	0.00984	0.01001	0.00838	0.00649	0.00708
7.7E-07	0.00996	0.00994	0.00844	0.0065	0.00726
8E-07	0.00978	0.01072	0.0086	0.00598	0.00636
8.3E-07	0.00879	0.01028	0.00701	0.00542	0.00565
8.6E-07	0.0097	0.00958	0.00757	0.00521	0.00583
9E-07	0.00797	0.00898	0.00711	0.00501	0.00587
9.3E-07	0.00832	0.00845	0.00718	0.00429	0.00524
9.6E-07	0.00878	0.00731	0.00624	0.00496	0.00543
9.9E-07	0.00814	0.00854	0.0064	0.0047	0.00558
1E-06	0.00722	0.00668	0.00623	0.00374	0.00497
1.1E-06	0.00709	0.00794	0.00511	0.00301	0.00402
1.1E-06	0.00733	0.00713	0.00631	0.00378	0.004
1.1E-06	0.00634	0.00756	0.00536	0.00359	0.00412
1.2E-06	0.00719	0.00722	0.00545	0.00258	0.00412
1.2E-06	0.00563	0.00595	0.00557	0.00337	0.00448
1.2E-06	0.00589	0.00629	0.00525	0.00382	0.00386
1.2E-06	0.00626	0.00631	0.00485	0.00213	0.00443
1.3E-06	0.00604	0.00508	0.0045	0.00242	0.00474
1.3E-06	0.00529	0.00535	0.00542	0.00258	0.00385
1.3E-06	0.00572	0.00541	0.00399	0.00205	0.00496
1.4E-06	0.00557	0.00533	0.00454	0.0016	0.00382
1.4E-06	0.00452	0.00451	0.00363	0.00268	0.00424
1.4E-06	0.00494	0.00453	0.00443	0.00163	0.00364
1.5E-06	0.00408	0.00338	0.00391	0.00183	0.00376
1.5E-06	0.00467	0.00381	0.00474	0.00257	0.00328
1.5E-06	0.00495	0.00485	0.00362	0.00156	0.00397
1.6E-06	0.00454	0.00453	0.0048	0.00176	0.0032
1.6E-06	0.00464	0.00488	0.00308	0.00239	0.0032
1.6E-06	0.00405	0.00426	0.00409	0.00109	0.00327
1.7E-06	0.00399	0.00386	0.00289	0.00204	0.00352
1.7E-06	0.00467	0.0041	0.00361	0.00203	0.00376
1.7E-06	0.00349	0.0029	0.00372	0.00135	0.00224
1.8E-06	0.00337	0.00353	0.00301	0.00217	0.00271
1.8E-06	0.00447	0.00308	0.00282	0.00227	0.00289
1.8E-06	0.00328	0.00325	0.003	0.00149	0.00283
1.9E-06	0.00306	0.00316	0.00288	0.00241	0.0023
1.9E-06	0.00295	0.00327	0.0036	0.0012	0.00327
1.9E-06	0.00419	0.00297	0.00226	0.0007	0.00373
2E-06	0.00353	0.00373	0.00311	0.00153	0.00325
2E-06	0.00313	0.00228	0.00271	0.00165	0.00301
2E-06	0.00348	0.00254	0.00347	0.00098	0.00304
2E-06	0.00365	0.0026	0.0032	0.00121	0.00222
2.1E-06	0.00265	0.00211	0.00335	0.00141	0.00262
2.1E-06	0.00181	0.00239	0.00298	0.00172	0.00279
2.1E-06	0.00287	0.00291	0.0028	0.0004	0.00216
2.2E-06	0.00303	0.00183	0.00272	0.0014	0.00284
2.2E-06	0.00242	0.00248	0.0029	0.00064	0.00231
2.2E-06	0.00183	0.00257	0.00222	0.00084	0.0023
2.3E-06	0.00178	0.00231	0.00298	0.0012	0.00354

2.3E-06	0.00193	0.00227	0.00276	0.00109	0.00308
2.3E-06	0.00173	0.00264	0.00308	0.00066	0.00166
2.4E-06	0.00168	0.00355	0.00218	0.00143	0.00347
2.4E-06	0.00164	0.00252	0.00231	0.00151	0.00295
2.4E-06	0.00147	0.00219	0.0022	0.00176	0.00253
2.5E-06	0.00201	0.0017	0.00224	0.00172	0.00404
2.5E-06	0.00201	0.00219	0.00258	0.00137	0.0031
2.5E-06	0.00187	0.00349	0.00192	0.0008	0.00285
2.6E-06	0.00201	0.00251	0.00268	0.00214	0.00235
2.6E-06	0.00133	0.00116	0.00289	0.00084	0.00264
2.6E-06	0.0004	0.00254	0.00196	0.00085	0.00217
2.7E-06	0.00148	0.00241	0.00201	0.00142	0.00283
2.7E-06	0.00141	0.00203	0.00295	0.0013	0.00265
2.7E-06	0.00101	0.00219	0.00189	0.00142	0.00237
2.8E-06	7.6E-06	0.00236	0.00256	0.00114	0.00284
2.8E-06	0.00119	0.00229	0.00178	0.00197	0.00259
2.8E-06	0.00099	0.00251	0.00165	0.00098	0.00236
2.8E-06	0.00245	0.00201	0.00272	1.5E-05	0.00284
2.9E-06	0.00059	0.00134	0.00291	0.00088	0.00205
2.9E-06	0.00122	0.00242	0.00207	0.00092	0.00243
2.9E-06	0.001	0.00132	0.00292	0.00078	0.00259
3E-06	0.00052	0.00282	0.00154	0.00039	0.00277
3E-06	0.00183	0.00235	0.00136	0.00037	0.00213
3E-06	0.00111	0.00135	0.00238	0.00092	0.00277
3.1E-06	2.2E-05	0.00181	0.00123	0.00081	0.00271
3.1E-06	0.00051	0.00227	0.00236	0.00032	0.00304
3.1E-06	0.00047	0.00155	0.00222	0.00035	0.00234
3.2E-06	0.00047	0.00176	0.00177	0.00111	0.0013
3.2E-06	0.00116	0.00134	0.00136	0.00056	0.00331
3.2E-06	0.00026	0.00153	0.00217	0.00178	0.00233
3.3E-06	0.00081	0.00207	0.00311	0.001	0.00229
3.3E-06	0.00027	0.00177	0.00148	0.00069	0.00292
3.3E-06	0.00102	0.00065	0.00211	7E-05	0.00198
3.4E-06	-0.0002	0.00129	0.00242	5.7E-05	0.0024
3.4E-06	0.00013	0.0017	0.0026	0.00105	0.00296
3.4E-06	-0.0003	0.00183	0.00239	0.00097	0.00296
3.5E-06	-0.0003	0.00128	0.00131	0.00031	0.00205
3.5E-06	3.5E-05	0.00173	0.00158	0.00116	0.00156
3.5E-06	0.00065	0.00282	0.00191	0.00065	0.00268
3.6E-06	0.00024	0.00157	0.00163	0.00073	0.00242
3.6E-06	0.0003	0.00153	0.00218	0.00114	0.00144
3.6E-06	0.00018	0.00115	0.00172	0.00059	0.0023
3.6E-06	-0.0002	0.00165	0.00191	0.00052	0.00184
3.7E-06	-0.0003	0.00109	0.00275	0.00037	0.00278
3.7E-06	-0.0002	0.00191	0.00189	0.00094	0.00224
3.7E-06	-0.0003	0.00161	0.00254	0.0004	0.00272
3.8E-06	1E-05	0.00147	0.0013	0.00116	0.00189
3.8E-06	-0.0004	0.00129	0.00223	0.00057	0.0023
3.8E-06	0.00043	0.00185	0.00225	0.00077	0.00203
3.9E-06	0.00056	0.00125	0.00123	0.00113	0.00197
3.9E-06	0.00114	0.00177	0.00157	0.00147	0.00217
3.9E-06	0.00027	0.0017	0.00208	0.00051	0.00345
4E-06	-2E-06	0.00135	0.00216	0.00087	0.00317
4E-06	-5E-05	0.00087	0.0033	0.00109	0.00258

4E-06	0.00029	0.0017	0.00163	0.00028	0.00208
4.1E-06	2.1E-05	0.00065	0.00223	0.0013	0.00209
4.1E-06	0.00113	0.00175	0.00224	0.00099	0.00187
4.1E-06	0.00057	0.0011	0.00147	0.00103	0.00237
4.2E-06	-0.0003	0.00033	0.00192	0.00019	0.00258
4.2E-06	5.2E-05	0.00133	0.00166	0.00054	0.0024
4.2E-06	3.8E-05	0.00103	0.00226	0.00076	0.00241
4.3E-06	0.00071	0.00171	0.00237	0.00081	0.00189
4.3E-06	0.00056	0.00194	0.00133	0.00059	0.00239
4.3E-06	0.00017	0.00118	0.00137	0.00147	0.00206
4.4E-06	0.00012	0.00165	0.0023	0.00072	0.00157
4.4E-06	-0.0011	0.00049	0.00239	0.00058	0.00235
4.4E-06	9.3E-06	0.00139	0.00134	0.00033	0.0026
4.4E-06	0.00019	0.00078	0.0012	0.00105	0.00218
4.5E-06	-0.0003	0.00093	0.00254	0.00115	0.00182
4.5E-06	-0.0002	0.00166	0.00222	0.00153	0.00198
4.5E-06	7.1E-05	0.00168	0.00236	0.00053	0.00209
4.6E-06	-0.0006	0.00092	0.0023	0.00108	0.00129
4.6E-06	0.00024	0.00211	0.00227	0.00038	0.0019
4.6E-06	-5E-05	0.00179	0.00173	0.00113	0.00161
4.7E-06	0.0002	0.00151	0.0016	0.00098	0.00132
4.7E-06	4.3E-05	0.001	0.00196	0.00109	0.00114
4.7E-06	0.00033	0.00128	0.00168	0.00107	0.00202
4.8E-06	0.00027	0.00159	0.00179	0.00114	0.00159
4.8E-06	0.00038	0.00094	0.002	0.0007	0.00211
4.8E-06	0.00066	0.00162	0.00206	0.00087	0.00162
4.9E-06	-0.0006	0.00191	0.00234	0.0011	0.00238
4.9E-06	-0.0003	0.00177	0.00275	0.00014	0.00257
4.9E-06	-5E-05	0.00209	0.00241	0.00025	0.00279
5E-06	-0.0009	0.0014	0.00189	0.00098	0.00202
5E-06	-0.0003	0.00113	0.00136	0.00058	0.00223
5E-06	0.00032	0.00152	0.00156	0.00122	0.00211
5.1E-06	-0.0006	0.00076	0.00179	0.00064	0.00252
5.1E-06	0.00039	0.00156	0.00194	0.00082	0.00242
5.1E-06	-0.0003	0.00129	0.00117	0.00128	0.00265
5.2E-06	-0.0005	0.00128	0.00104	0.00084	0.0017
5.2E-06	-0.0011	0.00091	0.0022	0.00064	0.0023
5.2E-06	2.1E-05	0.00184	0.00195	0.00063	0.00217
5.2E-06	0.00036	0.00107	0.00139	0.00066	0.00236
5.3E-06	-0.0009	0.00221	0.00184	0.00069	0.00202
5.3E-06	6.2E-06	0.00166	0.00125	0.0013	0.00153
5.3E-06	0.0006	0.0018	0.00206	0.00085	0.00222
5.4E-06	-0.0009	0.00164	0.00214	0.00145	0.00156
5.4E-06	-0.0008	0.00111	0.00182	0.00164	0.00147
5.4E-06	-0.0003	0.00108	0.00246	0.00109	0.00231
5.5E-06	-9E-05	0.00141	0.00207	0.00159	0.00231
5.5E-06	-0.0009	0.00017	0.00167	0.00111	0.00242
5.5E-06	-0.0003	0.00106	0.0018	0.00109	0.00363
5.6E-06	0.00087	0.00057	0.00273	0.00068	0.00202
5.6E-06	-0.0004	0.00122	0.0009	0.00067	0.00155
5.6E-06	-0.0007	0.00092	0.00237	0.00074	0.00232
5.7E-06	-0.0003	0.00057	0.00095	0.00077	0.00205
5.7E-06	-0.0002	0.00115	0.00135	0.00112	0.00249
5.7E-06	-0.0005	0.0013	0.00116	0.00106	0.002

5.8E-06	-0.0006	0.00024	0.00194	0.00074	0.00215
5.8E-06	-0.0008	0.0008	0.00222	0.00044	0.00196
5.8E-06	-0.0002	0.00051	0.00141	0.00113	0.00259
5.9E-06	0.00019	0.00156	0.00169	0.0004	0.002
5.9E-06	0.00038	0.00152	0.00247	0.00074	0.00217
5.9E-06	-1E-04	0.001	0.00207	0.00029	0.0013
6E-06	-0.0003	0.00103	0.00191	0.00052	0.00231
6E-06	-0.0009	0.00048	0.00124	0.00061	0.00206
6E-06	-0.0005	0.001	0.00226	0.00055	0.00245
6E-06	0.00019	0.00066	0.0014	0.00089	0.00214
6.1E-06	-0.0006	0.00081	0.00134	0.00041	0.00141
6.1E-06	-7E-05	0.00022	0.00186	0.00035	0.0023
6.1E-06	-0.0004	0.00191	0.00255	0.00021	0.00164
6.2E-06	0.00065	0.00073	0.00182	0.00115	0.00226
6.2E-06	0.00054	0.00121	0.00182	0.0009	0.00159
6.2E-06	-0.0001	0.00062	0.00168	0.00115	0.00263
6.3E-06	0.00016	0.00117	0.0027	0.00093	0.00209
6.3E-06	0.00023	0.00155	0.00223	0.00079	0.00154
6.3E-06	-0.0002	0.00125	0.00172	0.0007	0.00237
6.4E-06	-0.0003	0.00038	0.00223	0.00127	0.00184
6.4E-06	-0.0002	0.00106	0.0017	0.00089	0.00202
6.4E-06	6.6E-05	0.00112	0.00206	0.0004	0.00197
6.5E-06	0.00039	0.00094	0.00194	0.00056	0.00199
6.5E-06	-0.0003	0.00074	0.00217	0.00109	0.00213
6.5E-06	0.00082	0.00102	0.00137	0.00044	0.00178
6.6E-06	-0.0008	0.00081	0.00158	0.00161	0.00179
6.6E-06	-0.0007	0.00124	0.00189	0.00047	0.00213
6.6E-06	-0.0009	0.00044	0.00192	0.00073	0.00221
6.7E-06	-0.0004	0.0012	0.00141	0.00124	0.00198
6.7E-06	0.0004	0.00131	0.00261	0.00091	0.00222
6.7E-06	-0.0004	0.00043	0.00232	0.00106	0.00124
6.8E-06	-0.0005	-8E-05	0.00113	0.00154	0.00235
6.8E-06	0.00042	0.00044	0.0018	0.00039	0.00207
6.8E-06	-0.0004	0.00073	0.00244	0.00191	0.00237
6.8E-06	0.00031	0.00011	0.00215	0.00164	0.00195
6.9E-06	-4E-05	0.00121	0.00138	1.4E-05	0.00173
6.9E-06	-3E-05	0.00112	0.00157	0.00092	0.00229
6.9E-06	-0.0013	0.0006	0.00172	0.00146	0.00195
7E-06	-0.0007	0.0003	0.00199	0.00099	0.00246
7E-06	6.9E-05	0.00099	0.00106	0.001	0.00231
7E-06	-0.0006	0.00101	0.0017	0.00065	0.00203
7.1E-06	-0.0003	0.00099	0.00171	0.00082	0.00242
7.1E-06	-0.0004	0.00114	0.0013	0.00155	0.00226
7.1E-06	-0.0002	0.00085	0.00215	0.00061	0.00223
7.2E-06	-0.001	0.00156	0.00197	0.0005	0.00242
7.2E-06	0.00019	0.00147	0.00168	0.00065	0.00222
7.2E-06	-0.0002	0.00061	0.00168	0.00047	0.00203
7.3E-06	-7E-05	0.0012	0.00136	0.00086	0.00174
7.3E-06	8.5E-05	0.00068	0.00177	0.00155	0.0019
7.3E-06	-0.0004	0.00033	0.00247	0.00085	0.00256
7.4E-06	-0.0013	0.00084	0.00244	0.00035	0.00228
7.4E-06	6.4E-05	0.00172	0.00182	0.00044	0.00212
7.4E-06	0.00021	0.00106	0.00177	0.00056	0.00219
7.5E-06	-0.0004	0.00095	0.00184	0.00082	0.00105

7.5E-06	-0.0008	0.00145	0.00165	0.00109	0.00196
7.5E-06	-0.0003	0.00091	0.00214	0.00081	0.00229
7.6E-06	-0.0002	0.00149	0.0018	0.00122	0.00148
7.6E-06	-0.0013	0.00124	0.00216	0.00124	0.00133
7.6E-06	-0.0003	0.00049	0.0017	0.00063	0.0024
7.6E-06	0.00028	0.00071	0.0012	0.00118	0.00149
7.7E-06	-0.0002	0.00125	0.00183	0.0009	0.00206
7.7E-06	0.0006	0.00154	0.00204	0.00087	0.00197
7.7E-06	-0.0006	0.00162	0.00207	0.00129	0.00158
7.8E-06	-0.0009	0.00186	0.00191	0.00117	0.00219
7.8E-06	-3E-05	0.0006	0.00075	0.00136	0.00251
7.8E-06	-0.0003	0.00147	0.00148	0.00045	0.00163
7.9E-06	-0.0008	0.00073	0.0025	0.00189	0.00191
7.9E-06	-0.0006	0.00177	0.00249	0.00064	0.00147
7.9E-06	-0.0013	0.00088	0.00234	0.00083	0.0022
8E-06	-0.0003	0.00095	0.00205	0.00163	0.00196
8E-06	-0.0006	0.00093	0.00115	0.00117	0.00186
8E-06	-0.001	0.0006	0.00165	0.00044	0.0022
8.1E-06	-0.0005	0.0004	0.00205	0.00083	0.0026
8.1E-06	-0.0001	0.00038	0.00229	0.00026	0.00209
8.1E-06	-0.0021	0.00061	0.00245	0.00024	0.00226
8.2E-06	-0.0012	5.2E-05	0.00121	0.00046	0.00251
8.2E-06	-0.001	0.0008	0.00262	0.00127	0.00202
8.2E-06	-0.0007	0.00139	0.0018	0.0011	0.00222
8.3E-06	-0.0003	0.00056	0.00241	0.00022	0.00222
8.3E-06	0.00043	0.00102	0.00215	0.00032	0.00192
8.3E-06	0.00012	0.00044	0.00215	0.00108	0.00205
8.4E-06	-0.0001	0.00081	0.00191	0.00083	0.0015
8.4E-06	-0.0008	0.00082	0.00185	0.00089	0.00275
8.4E-06	-0.0001	0.00153	0.00157	0.00114	0.00198
8.4E-06	-0.0006	0.00128	0.00171	0.00043	0.00148
8.5E-06	-0.0008	0.00099	0.002	0.00064	0.00227
8.5E-06	-0.0007	0.0004	0.0025	0.00057	0.00193
8.5E-06	-0.0006	0.00065	0.00199	0.0003	0.00195
8.6E-06	-0.0001	0.00151	0.00124	0.00065	0.00235
8.6E-06	-0.0004	0.00045	0.00195	0.00101	0.00227
8.6E-06	-0.0007	0.0014	0.0014	0.00085	0.00146
8.7E-06	0.00034	0.0018	0.00166	0.0003	0.00226
8.7E-06	-0.0009	0.0014	0.00147	-0.0002	0.00148
8.7E-06	-0.001	0.00141	0.00226	0.00082	0.00256
8.8E-06	-6E-05	0.00086	0.00195	0.00091	0.00236
8.8E-06	-7E-05	0.00125	0.00142	0.00049	0.00208
8.8E-06	-0.0004	0.00097	0.00182	0.00053	0.00153
8.9E-06	1.2E-05	0.00085	0.00239	0.00034	0.00173
8.9E-06	-0.0003	0.00169	0.00186	0.00026	0.00176
8.9E-06	-0.0003	0.00062	0.00235	0.0011	0.00266
9E-06	-0.0012	0.00096	0.00238	0.00109	0.00215
9E-06	-0.0007	0.00171	0.00259	0.0017	0.00205
9E-06	-0.0015	0.0014	0.00235	0.0005	0.00215
9.1E-06	-0.0008	0.00131	0.00186	0.00117	0.00224
9.1E-06	-0.0005	0.00116	0.00225	0.00086	0.00184
9.1E-06	5.1E-05	0.00148	0.00166	0.00143	0.00204
9.2E-06	-0.0005	0.00134	0.001	0.00102	0.00157
9.2E-06	-0.0018	0.00107	0.00242	0.00079	0.00177

9.2E-06	-0.0012	0.00048	0.00181	0.00114	0.00066
9.2E-06	6.8E-05	0.00062	0.00195	0.00165	0.00233
9.3E-06	-0.0002	0.00086	0.00147	0.0011	0.00225
9.3E-06	-0.0005	0.00138	0.00187	0.00065	0.00158
9.3E-06	-0.0004	0.00104	0.0027	0.00018	0.0022
9.4E-06	8.2E-06	0.00103	0.00226	0.00048	0.00244
9.4E-06	-0.0001	0.00086	0.00224	0.00096	0.00171
9.4E-06	-0.0012	0.00056	0.00239	-6E-05	0.00199
9.5E-06	0.00035	0.00096	0.00162	0.00111	0.0023
9.5E-06	-0.0006	0.00105	0.00187	0.00142	0.0024
9.5E-06	-0.0002	0.00151	0.00202	0.00071	0.00156
9.6E-06	-0.0003	0.00095	0.00217	0.00211	0.00182
9.6E-06	0.00036	0.00138	0.00181	0.00117	0.0019
9.6E-06	-9E-05	0.00029	0.00162	0.00013	0.00217
9.7E-06	0.00022	0.00188	0.00169	0.00067	0.00162
9.7E-06	0.00031	0.00186	0.0012	0.00164	0.00226
9.7E-06	-0.0012	0.00111	0.00184	0.00082	0.00165
9.8E-06	-0.0013	0.0005	0.0018	0.00062	0.00208
9.8E-06	-0.0012	0.00153	0.0028	0.00019	0.0021
9.8E-06	-0.0002	0.00042	0.00233	0.0006	0.00185
9.9E-06	-0.0007	0.00043	0.00208	0.00118	0.00232
9.9E-06	0.00022	0.00098	0.00231	0.0005	0.00227
9.9E-06	-0.0006	0.00015	0.00173	0.00084	0.0015
1E-05	-0.001	0.00163	0.00146	0.00024	0.00161
1E-05	-0.0014	0.00067	0.00175	0.00081	0.00092
1E-05	-0.0011	0.00079	0.00196	4.5E-05	0.00256
1E-05	-0.0011	0.00107	0.00252	0.00022	0.00246
1E-05	-0.0001	0.00125	0.00234	0.00166	0.00209
1E-05	-0.0001	0.00015	0.00206	0.0017	0.00176
1E-05	-0.0004	0.00094	0.00102	0.00026	0.00216
1E-05	-0.0006	0.0011	0.00155	0.00059	0.00181
1E-05	-0.0003	0.00068	0.00221	0.0003	0.00189
1E-05	0.00029	0.00112	0.00229	0.00108	0.00157
1E-05	-0.0008	0.00053	0.00158	0.00084	0.00178
1E-05	-0.0005	0.00049	0.00238	0.00108	0.00211
1E-05	-0.0002	0.0009	0.00274	0.00133	0.0023
1E-05	-0.0006	0.00079	0.0012	0.001	0.00169
1E-05	-0.0011	0.0003	0.00178	0.00046	0.00226
1E-05	-0.0002	0.00095	0.00211	0.00126	0.00171
1E-05	-6E-05	0.00045	0.00178	0.00119	0.00186
1.1E-05	-0.0007	-6E-05	0.00197	0.00065	0.00156
1.1E-05	-2E-05	0.00086	0.00175	0.0005	0.00156
1.1E-05	8.6E-06	0.00047	0.00209	0.00041	0.00197
1.1E-05	-0.0002	0.00125	0.00211	0.00025	0.00214
1.1E-05	-0.0006	0.00058	0.00166	0.00056	0.00208
1.1E-05	-0.0007	0.00158	0.00159	0.00085	0.00191
1.1E-05	-0.0012	0.00041	0.00182	0.00089	0.00234
1.1E-05	0.00021	0.00116	0.0023	0.00085	0.0024
1.1E-05	0.00023	0.0008	0.00245	0.00109	0.00164
1.1E-05	2.6E-05	0.00094	0.00112	0.00079	0.00215
1.1E-05	-0.0012	0.00084	0.00176	0.00107	0.00181
1.1E-05	-0.0006	0.00076	0.002	0.0011	0.00169
1.1E-05	-0.0009	0.00124	0.00211	0.00085	0.00235
1.1E-05	-0.0002	0.00037	0.00183	0.00171	0.00226

1.1E-05	-0.0007	0.0006	0.00234	0.0005	0.00227
1.1E-05	-0.0003	0.00171	0.00174	0.00136	0.00177
1.1E-05	6.2E-05	0.00102	0.00042	0.00034	0.00137
1.1E-05	-6E-05	0.00093	0.00222	0.00047	0.00256
1.1E-05	-0.0012	0.0003	0.00164	0.00083	0.00329
1.1E-05	-0.0004	0.00121	0.00161	0.00125	0.00137
1.1E-05	-4E-05	0.0002	0.00179	0.00099	0.00268
1.1E-05	-0.0003	-0.0005	0.00131	0.00107	0.00163
1.1E-05	-0.0013	0.00136	0.00142	0.00071	0.00186
1.1E-05	-0.001	0.00077	0.00145	0.0004	0.00153
1.1E-05	-9E-05	0.00043	0.00131	0.00146	0.00214
1.1E-05	-0.0006	0.0011	0.00186	0.00032	0.00217
1.1E-05	-0.0003	0.00098	0.00099	0.00086	0.00217
1.1E-05	-0.001	0.00102	0.00132	0.00072	0.00191
1.1E-05	-0.0008	0.00076	0.00192	0.00164	0.00223
1.1E-05	-0.0005	0.00034	0.00243	0.0015	0.00188
1.1E-05	8.7E-05	0.00035	0.00183	0.00105	0.00176
1.1E-05	-0.0005	0.00029	0.00245	0.0015	0.00249
1.2E-05	-0.0005	0.00092	0.002	0.00076	0.00231
1.2E-05	-0.0012	0.00104	0.00252	0.00127	0.00175
1.2E-05	-0.0012	0.00066	0.00264	0.00078	0.00185
1.2E-05	-0.0007	0.00152	0.00165	0.00078	0.00263
1.2E-05	-0.0015	0.00074	0.00205	0.00106	0.00168
1.2E-05	-0.0003	0.00079	0.0012	8.3E-05	0.0018
1.2E-05	-0.001	0.00153	0.00159	0.00087	0.00194
1.2E-05	0.00024	0.00108	0.00112	0.00135	0.00171
1.2E-05	-0.0006	0.00121	0.00199	0.00053	0.00146
1.2E-05	-0.0005	0.00127	0.00191	0.00082	0.00087
1.2E-05	-1E-05	0.00057	0.0019	0.00101	0.00297
1.2E-05	-0.0007	0.00052	0.00119	0.00166	0.00188
1.2E-05	-0.0002	0.00071	0.00136	0.00047	0.0022
1.2E-05	-0.0012	0.00116	0.00113	0.00149	0.0018
1.2E-05	-0.0004	0.00125	0.00193	0.00149	0.00218
1.2E-05	-0.0009	0.00039	0.00169	0.00019	0.00187
1.2E-05	-0.0004	0.00092	0.00149	0.0008	0.00253
1.2E-05	-0.001	0.00122	0.00119	0.0005	0.00152
1.2E-05	-0.0002	0.00037	0.00204	0.00051	0.00176
1.2E-05	-0.0005	0.00056	0.00196	0.00064	0.00219
1.2E-05	-0.0007	0.00016	0.00173	0.00043	0.00128
1.2E-05	-0.0008	0.00096	0.00298	0.00018	0.00305
1.2E-05	-0.0009	0.0006	0.0019	0.00082	0.00221
1.2E-05	-0.0002	0.00093	0.0025	0.00068	0.00191
1.2E-05	-0.0008	0.00042	0.00151	0.00064	0.00191
1.2E-05	-0.0012	0.0009	0.00182	0.00073	0.00208
1.2E-05	-0.0006	0.00092	0.00165	0.0012	0.00287
1.2E-05	-0.0009	0.00064	0.00214	0.00115	0.00214
1.2E-05	-0.0018	0.00023	0.00241	0.00092	0.00239
1.2E-05	-0.0009	0.00068	0.00273	0.00126	0.00222
1.2E-05	-0.0009	5.3E-05	0.00199	0.00112	0.00178
1.3E-05	-0.0007	0.0007	0.00162	0.00062	0.00191
1.3E-05	0.00014	0.00121	0.00156	0.00023	0.00214
1.3E-05	-8E-05	0.00125	0.0024	0.00075	0.00161
1.3E-05	-0.0003	0.00117	0.00069	0.00067	0.00149
1.3E-05	-0.0007	0.00062	0.00131	0.00109	0.00174

1.3E-05	-0.0007	0.00032	0.00201	0.00089	0.00227
1.3E-05	-0.0002	0.00099	0.00249	0.00044	0.00205
1.3E-05	-0.001	0.00092	0.00185	0.00067	0.00144
1.3E-05	-0.0009	0.00072	0.00193	0.00111	0.0016
1.3E-05	-0.0014	0.00012	0.00238	9.8E-05	0.00208
1.3E-05	-0.0008	0.00036	0.0023	0.00088	0.00207
1.3E-05	-0.0007	8E-05	0.00153	0.00087	0.00239
1.3E-05	-0.0012	0.00037	0.00108	0.00068	0.00233
1.3E-05	-0.0011	0.00014	0.00182	0.00131	0.00225
1.3E-05	-0.0006	0.00131	0.00204	-3E-05	0.00195
1.3E-05	-0.0003	0.00038	0.00207	0.0002	0.0014
1.3E-05	-0.0007	9.8E-05	0.0021	0.00077	0.00274
1.3E-05	-0.0005	-0.0001	0.00122	0.00109	0.00197
1.3E-05	0.00049	0.00145	0.00212	0.00059	0.00221
1.3E-05	-0.0008	0.00161	0.00255	0.00084	0.00149
1.3E-05	-0.0012	0.00033	0.00231	0.00027	0.00237
1.3E-05	-0.0012	0.00048	0.00179	0.00094	0.00187
1.3E-05	-0.0005	-0.0003	0.00249	0.00118	0.0021
1.3E-05	-0.0005	0.00073	0.00182	0.00091	0.00239
1.3E-05	-0.0002	0.0005	0.00112	0.00107	0.00246
1.3E-05	-2E-05	0.00054	0.00225	0.00058	0.00193
1.3E-05	-0.0007	0.0007	0.00156	0.00062	0.00135
1.3E-05	-0.0005	0.00026	0.00184	0.00129	0.00178
1.3E-05	0.00018	0.00021	0.00227	0.00019	0.0017
1.3E-05	-0.0004	0.00063	0.00274	0.00054	0.00197
1.3E-05	-0.0001	0.00025	0.00228	0.00094	0.0008
1.4E-05	-0.0002	0.00037	0.00209	0.00109	0.00188
1.4E-05	3.4E-05	0.00097	0.002	0.00041	0.00187
1.4E-05	-0.0007	0.00146	0.00181	0.00065	0.00155
1.4E-05	-0.0008	0.0015	0.0022	0.00052	0.00246
1.4E-05	-0.0004	0.00095	0.00236	0.00172	0.00074
1.4E-05	-0.0007	0.00096	0.00178	0.00065	0.00177
1.4E-05	-0.0007	0.00076	0.00251	0.00109	0.00237
1.4E-05	-0.0003	0.00069	0.00216	0.00047	0.00137
1.4E-05	-0.0019	0.00013	0.00189	0.00127	0.00294
1.4E-05	0.00013	0.00049	0.00197	0.00074	0.00184
1.4E-05	0.00019	0.00116	0.00213	0.00135	0.00157
1.4E-05	-0.0008	0.00067	0.00154	0.00082	0.00189
1.4E-05	-0.0011	0.00055	0.00215	0.00016	0.00167
1.4E-05	-0.0002	0.00062	0.00192	0.00029	0.00168
1.4E-05	-0.0002	0.00088	0.0017	0.00084	0.00215
1.4E-05	0.00025	0.00092	0.00146	0.00093	0.0018
1.4E-05	0.00017	0.00101	0.00136	0.00072	0.00168
1.4E-05	-0.0004	0.00065	0.00223	0.00105	0.00225
1.4E-05	-0.0005	0.00145	0.00221	0.0013	0.00185
1.4E-05	-0.0009	-0.0002	0.00208	0.00077	0.00159
1.4E-05	-4E-05	0.00078	0.00287	0.00118	0.0012
1.4E-05	-0.0005	0.00117	0.00174	0.00055	0.00156
1.4E-05	0.00013	0.00071	0.00288	0.00105	0.00174
1.4E-05	-0.0005	0.00014	0.00173	0.0006	0.00163
1.4E-05	-0.0001	0.00088	0.00184	0.00091	0.0025
1.4E-05	-0.0008	-0.0002	0.00203	0.00062	0.00199
1.4E-05	-0.0003	0.0004	0.00233	0.00114	0.00221
1.4E-05	-0.0009	0.00013	0.00131	0.00129	0.00253

1.4E-05	-0.001	0.00076	0.00219	0.00106	0.00242
1.4E-05	0.0004	0.00052	0.00206	0.00032	0.00261
1.4E-05	0.00036	0.001	0.00276	0.00094	0.00222
1.5E-05	-0.0008	0.00046	0.00246	0.00061	0.0021
1.5E-05	-0.0004	0.0007	0.00195	0.00064	0.00189
1.5E-05	-5E-05	0.00066	0.00195	0.00066	0.00196
1.5E-05	-0.0003	0.00104	0.0014	0.00069	0.00165
1.5E-05	-0.0014	0.00074	0.00203	0.0004	0.00127
1.5E-05	-0.0008	0.00146	0.00181	0.00109	0.00209
1.5E-05	8.9E-05	0.00094	0.00168	0.00127	0.00246
1.5E-05	-0.0007	0.00057	0.00098	0.00116	0.00239
1.5E-05	-0.0009	0.00025	0.00135	0.00116	0.00234
1.5E-05	-0.0016	0.0007	0.00278	0.00196	0.00293
1.5E-05	-0.001	0.00141	0.00223	0.00131	0.002
1.5E-05	0.0006	0.0007	0.00181	0.00126	0.00195
1.5E-05	0.00016	0.00059	0.00179	0.00113	0.00192
1.5E-05	-0.0009	0.00062	0.00143	0.00043	0.002
1.5E-05	0.00038	0.00013	0.00148	0.00125	0.00227
1.5E-05	-0.0005	0.00092	0.00179	0.00053	0.00189

Table SI.10: Primary kinetic data for the reaction of ε-caprolactam (**S10**) with CumO• generated by 355 nm LFP in MeCN (**Figure SI.10**).

t / s	ΔA ε-caprolactam (S10)				
	0.1M	0.303M	0.5M	0.74M	1.0M
-1E-06	-0.0002	1.3E-05	-0.0006	-0.0002	0.00026
-1E-06	-0.0006	0.00018	-1E-05	-8E-05	1.4E-05
-9E-07	-0.0013	0.00016	-0.0008	0.0013	-0.0001
-9E-07	-0.0007	0.00052	-0.0006	0.00028	0.00011
-9E-07	-0.0012	0.00029	-4E-05	0.00039	-0.0001
-8E-07	-0.0004	0.0008	-0.0004	-0.0002	0.00015
-8E-07	-0.0006	0.00027	-0.0002	0.00063	0.00029
-8E-07	-0.0006	0.0009	-0.0006	7.4E-05	-0.0002
-7E-07	-0.0004	0.00029	-0.0004	0.00063	0.00059
-7E-07	-0.0012	0.00031	-0.0011	8.1E-05	-3E-05
-7E-07	-0.0009	0.00027	-4E-05	0.00051	0.00059
-6E-07	-0.0007	0.0005	-0.0001	0.00015	0.00051
-6E-07	-0.0007	0.00012	-0.0009	0.0007	-2E-05
-6E-07	-0.0011	0.00075	-0.0002	0.00027	0.00016
-5E-07	-0.0009	0.00039	-0.0004	0.00029	-0.0002
-5E-07	-0.0005	0.00043	-0.0006	0.00085	-0.0009
-5E-07	-0.0009	0.00036	-0.0007	-0.0004	-7E-05
-4E-07	-0.0011	0.00041	-0.0002	0.00016	0.00041
-4E-07	-0.0009	0.00056	-2E-06	0.00044	0.00014
-4E-07	-0.0002	0.00042	-0.0007	0.00015	-0.0002
-4E-07	-0.0005	0.00043	-0.0004	0.00061	0.00029
-3E-07	-0.0012	-0.0001	1.6E-05	0.00075	0.00042
-3E-07	-0.0009	-0.0003	-9E-05	0.00064	0.00043
-3E-07	-0.0009	0.00037	-0.0006	0.00021	0.00033
-2E-07	-0.0006	0.00041	-0.0005	-8E-05	0.00068
-2E-07	-0.0015	0.00047	0.00021	0.00014	0.00034
-2E-07	-0.0018	0.0002	-0.0005	0.00051	0.00108
-1E-07	-0.0011	0.00041	-0.0012	0.00063	-0.0015
-1E-07	-0.0017	0.00042	-0.001	0.00012	0.00036
-6E-08	-0.0006	0.00028	-0.0007	1.9E-05	-0.0002
-3E-08	-0.0008	0.00071	-0.0009	-0.0003	0.00012
-4E-10	-0.0042	-0.0037	-0.0036	-0.0022	-0.0031
3.2E-08	-0.0064	-0.0082	-0.0087	-0.0079	-0.0103
6.4E-08	0.01455	0.01639	0.01337	0.01329	0.01365
9.6E-08	0.01584	0.01684	0.01469	0.01441	0.01445
1.3E-07	0.01476	0.01654	0.01403	0.01389	0.0135
1.6E-07	0.01461	0.01618	0.01416	0.01392	0.01235
1.9E-07	0.01416	0.01458	0.01349	0.01272	0.0119
2.2E-07	0.01431	0.01507	0.0126	0.01239	0.01177
2.6E-07	0.01368	0.01373	0.01265	0.0114	0.01095
2.9E-07	0.0134	0.01351	0.01217	0.01172	0.01069
3.2E-07	0.01351	0.01369	0.01197	0.01037	0.01006
3.5E-07	0.01266	0.01382	0.01113	0.0105	0.00994
3.8E-07	0.0116	0.01204	0.01081	0.01018	0.00907
4.2E-07	0.01193	0.01246	0.01213	0.01038	0.00854
4.5E-07	0.01142	0.01235	0.00975	0.00955	0.00792
4.8E-07	0.01046	0.01122	0.01062	0.00834	0.00815
5.1E-07	0.01115	0.01142	0.01021	0.00838	0.0083
5.4E-07	0.01024	0.01041	0.00944	0.00814	0.00668

5.8E-07	0.01064	0.01125	0.00888	0.00817	0.00692
6.1E-07	0.00987	0.01075	0.00792	0.00737	0.00743
6.4E-07	0.01026	0.00997	0.00799	0.00715	0.00595
6.7E-07	0.0097	0.0102	0.00783	0.00693	0.00642
7E-07	0.00904	0.00977	0.00712	0.0069	0.00547
7.4E-07	0.00843	0.00926	0.00682	0.00694	0.00582
7.7E-07	0.00862	0.00867	0.00622	0.00703	0.00549
8E-07	0.00883	0.00882	0.00677	0.00604	0.00524
8.3E-07	0.00812	0.00801	0.00656	0.00532	0.00478
8.6E-07	0.00881	0.00779	0.00625	0.00536	0.00447
9E-07	0.00695	0.00799	0.00561	0.00523	0.00506
9.3E-07	0.00712	0.00774	0.00578	0.0046	0.00487
9.6E-07	0.00738	0.00793	0.00592	0.00475	0.00306
9.9E-07	0.00748	0.00691	0.00517	0.00451	0.00322
1E-06	0.00714	0.00743	0.00539	0.0043	0.00359
1.1E-06	0.00722	0.00691	0.00422	0.00468	0.00384
1.1E-06	0.00614	0.00674	0.00442	0.00429	0.00278
1.1E-06	0.00693	0.00699	0.00398	0.00412	0.00346
1.2E-06	0.00566	0.00608	0.00448	0.00406	0.00365
1.2E-06	0.00566	0.00602	0.00406	0.00342	0.00311
1.2E-06	0.00575	0.00679	0.00355	0.00319	0.00292
1.2E-06	0.00523	0.00574	0.00409	0.0034	0.00275
1.3E-06	0.00562	0.00567	0.0037	0.00352	0.00252
1.3E-06	0.00437	0.00579	0.00368	0.00292	0.00187
1.3E-06	0.005	0.0056	0.00309	0.00342	0.00258
1.4E-06	0.00539	0.0056	0.00331	0.00338	0.00223
1.4E-06	0.00425	0.00579	0.00324	0.00309	0.00169
1.4E-06	0.00419	0.00538	0.00306	0.00266	0.00299
1.5E-06	0.00472	0.00511	0.0031	0.00292	0.00234
1.5E-06	0.00408	0.00529	0.0031	0.00322	0.0022
1.5E-06	0.00404	0.005	0.00273	0.00257	0.00161
1.6E-06	0.00405	0.00449	0.00208	0.0021	0.00143
1.6E-06	0.00401	0.00451	0.00209	0.00239	0.00267
1.6E-06	0.00419	0.00462	0.00207	0.00212	0.00092
1.7E-06	0.00312	0.00405	0.00205	0.00257	0.0016
1.7E-06	0.00395	0.00432	0.00209	0.00223	0.00178
1.7E-06	0.00324	0.00418	0.00326	0.00238	0.00196
1.8E-06	0.00346	0.00373	0.00154	0.00208	0.00171
1.8E-06	0.00368	0.00363	0.00204	0.00185	0.00068
1.8E-06	0.00335	0.00429	0.00128	0.00189	0.00154
1.9E-06	0.00314	0.0034	0.00214	0.00182	0.00156
1.9E-06	0.00238	0.00386	0.0012	0.00184	0.00134
1.9E-06	0.00319	0.00379	0.00171	0.00144	0.00198
2E-06	0.00264	0.00267	0.0018	0.00258	0.00191
2E-06	0.00238	0.00335	0.0006	0.00204	0.00093
2E-06	0.0028	0.00306	0.00044	0.00267	0.00149
2E-06	0.0024	0.00298	0.00085	0.00157	0.00113
2.1E-06	0.00253	0.00303	0.00113	0.00157	0.0011
2.1E-06	0.00227	0.00299	0.0015	0.00101	0.00039
2.1E-06	0.00177	0.00334	0.00086	0.00183	0.00102
2.2E-06	0.00215	0.00347	0.00122	0.00178	0.00101
2.2E-06	0.00175	0.00315	0.00138	0.00096	0.00169
2.2E-06	0.00212	0.00279	0.00129	0.00085	0.00175
2.3E-06	0.00257	0.0025	0.00096	0.00126	0.00158

2.3E-06	0.0018	0.00275	0.00058	0.00164	0.00037
2.3E-06	0.00158	0.00247	0.00154	0.00145	0.00101
2.4E-06	0.00194	0.00261	0.00106	0.00127	0.00132
2.4E-06	0.00156	0.0027	0.00078	0.00137	0.00105
2.4E-06	0.00186	0.0032	0.0011	0.00142	0.00097
2.5E-06	0.00114	0.00266	0.00068	0.00133	0.00058
2.5E-06	0.00186	0.00273	0.00123	0.00168	0.00044
2.5E-06	0.00092	0.00233	0.00045	0.00073	0.00091
2.6E-06	0.00126	0.00184	0.00114	0.00135	0.00069
2.6E-06	0.00097	0.00248	0.00043	0.00124	0.00166
2.6E-06	0.00069	0.00204	0.00047	0.00062	0.00104
2.7E-06	0.00145	0.0022	0.00059	0.00072	0.0007
2.7E-06	0.00085	0.00236	0.00022	0.00123	0.00023
2.7E-06	0.00167	0.00198	0.00045	0.00083	0.00031
2.8E-06	0.00105	0.0021	0.00051	0.00085	0.00057
2.8E-06	0.00108	0.00193	0.00056	0.00096	0.00124
2.8E-06	0.00082	0.00213	0.00046	0.00078	0.00087
2.8E-06	0.0007	0.00203	0.00016	0.00077	0.00093
2.9E-06	0.00024	0.0025	-1E-05	0.00054	0.00036
2.9E-06	0.001	0.00219	-0.0003	0.00119	0.00011
2.9E-06	0.00077	0.00184	-0.0005	0.00089	0.00072
3E-06	0.00099	0.0019	0.00026	0.00148	0.00056
3E-06	0.00037	0.00205	-3E-05	0.00077	0.00079
3E-06	0.00032	0.00227	0.00028	0.00073	0.00089
3.1E-06	-0.0003	0.00101	0.00046	0.00099	0.00116
3.1E-06	0.00042	0.00207	6.4E-05	0.00133	0.00046
3.1E-06	0.00046	0.00193	0.00038	0.00107	0.00104
3.2E-06	0.00049	0.00153	0.00129	0.00078	0.00041
3.2E-06	0.0005	0.00162	-0.0005	0.00092	0.00056
3.2E-06	0.00048	0.00187	-0.0007	0.0006	0.00088
3.3E-06	0.00043	0.00234	0.00013	0.00111	-0.0005
3.3E-06	0.0005	0.00163	-9E-05	0.00064	-3E-05
3.3E-06	0.00046	0.00148	0.00017	0.00093	-0.0001
3.4E-06	0.00023	0.00106	-6E-05	0.00083	0.00026
3.4E-06	0.00066	0.00146	-0.0007	0.00129	0.00045
3.4E-06	4.2E-05	0.00134	-0.0004	0.00073	-0.0002
3.5E-06	3.5E-05	0.00224	-0.0004	0.00108	0.00111
3.5E-06	0.00025	0.00187	0.00012	0.00108	0.00082
3.5E-06	0.00027	0.00131	-2E-05	0.00091	0.00024
3.6E-06	0.00022	0.0015	-5E-06	0.00058	0.00072
3.6E-06	-0.0002	0.00172	0.0001	0.00053	0.00133
3.6E-06	-0.0006	0.00194	1E-05	0.00019	0.00082
3.6E-06	-0.0003	0.00107	0.00017	0.00063	0.0007
3.7E-06	-4E-05	0.00102	-0.0002	0.00094	0.00052
3.7E-06	-0.0006	0.00108	-0.0001	0.00061	0.00075
3.7E-06	0.00022	0.00077	0.00013	0.00085	0.0001
3.8E-06	-0.0006	0.00147	-0.0001	-0.0002	0.00099
3.8E-06	0.00028	0.00202	-4E-06	0.00014	0.00061
3.8E-06	-0.0005	0.0021	-8E-05	-0.0002	0.00026
3.9E-06	0.00015	0.00145	0.00035	0.00013	0.00059
3.9E-06	-0.0006	0.00121	-0.0008	0.00025	-0.0004
3.9E-06	-0.0009	0.00143	-0.0003	0.00077	0.00029
4E-06	5.4E-05	0.00134	-0.0002	0.00058	0.00063
4E-06	-0.0002	0.0013	-4E-05	0.00075	0.00078

4E-06	-0.0008	0.0011	-0.0007	0.00042	0.00115
4.1E-06	0.00047	0.00133	-0.0004	0.00055	-3E-05
4.1E-06	-0.0009	0.00107	0.0001	0.00034	0.00101
4.1E-06	-0.0005	0.00152	-3E-05	0.00046	0.00037
4.2E-06	0.00027	0.0016	-6E-05	0.00069	6E-08
4.2E-06	0.00028	0.00143	-0.0002	0.00108	0.00026
4.2E-06	-0.0004	0.00093	-0.0003	0.00047	0.00056
4.3E-06	0.00017	0.00106	-0.001	0.00118	0.00034
4.3E-06	-0.0001	0.00059	-0.0003	0.00116	0.00048
4.3E-06	-0.0008	0.00121	-0.0001	0.00061	5E-05
4.4E-06	0.00012	0.00124	-0.0004	0.00017	0.00048
4.4E-06	-6E-05	0.00078	-3E-05	0.00074	-0.0004
4.4E-06	-0.0008	0.00063	-0.0004	0.00076	0.00037
4.4E-06	-0.0002	0.00065	-0.0004	-0.0003	-2E-05
4.5E-06	-0.0007	0.00157	-0.0002	0.00052	0.00045
4.5E-06	-0.0006	0.00084	-0.0004	0.00074	0.00093
4.5E-06	-0.0008	0.00078	-0.0002	0.00132	0.00051
4.6E-06	0.00051	0.00048	-0.0003	0.001	0.00036
4.6E-06	-0.0003	0.00122	-5E-05	0.00142	-0.0001
4.6E-06	-0.0008	0.00078	-0.0003	0.00037	0.00048
4.7E-06	-0.0008	0.00025	1E-04	0.00082	0.00045
4.7E-06	-0.0009	0.00027	-7E-05	0.00071	-0.0003
4.7E-06	0.00048	0.00072	-0.0006	0.00093	0.00072
4.8E-06	-0.0006	0.00106	2E-05	0.00105	0.00011
4.8E-06	-0.0002	0.00111	-0.0004	5.7E-05	-0.0003
4.8E-06	-0.0002	0.00118	-0.0005	0.0008	3E-05
4.9E-06	0.00044	0.00139	-0.0004	-0.0004	0.00054
4.9E-06	-0.0009	0.00036	-0.0003	9.1E-05	-1E-05
4.9E-06	-0.0006	0.00181	-3E-05	0.00064	0.00046
5E-06	-0.0002	0.00093	-0.0003	0.00068	-0.0003
5E-06	-0.0008	0.00057	3.5E-05	0.00107	0.00067
5E-06	-0.0014	0.00016	-0.0005	0.00012	8.3E-05
5.1E-06	-0.0015	0.00044	-0.0006	0.00071	0.0006
5.1E-06	-0.0012	0.00178	-0.0011	0.0009	0.00031
5.1E-06	-0.0006	0.00026	0.00011	0.0008	-2E-05
5.2E-06	-0.0008	0.00046	-0.0009	-4E-05	-0.0001
5.2E-06	-0.0006	0.00071	-0.0012	0.00041	-0.0006
5.2E-06	-0.0001	0.00153	-0.0005	7.4E-05	9.5E-05
5.2E-06	-0.0008	0.00069	-0.0004	0.00077	0.00055
5.3E-06	-0.001	0.00117	-4E-05	0.00014	-2E-06
5.3E-06	-0.0005	0.00055	-0.0009	0.00014	-0.0003
5.3E-06	-0.0014	0.00115	-0.0009	-0.0004	0.00089
5.4E-06	-0.0008	0.00123	-0.001	0.00044	0.0002
5.4E-06	-9E-05	0.00137	-0.0008	0.00022	-0.0002
5.4E-06	-0.0008	0.00101	-0.0007	0.00057	0.00029
5.5E-06	-0.0008	0.00089	-0.0003	0.00026	0.00128
5.5E-06	-0.002	0.00182	-0.0004	0.00035	0.0013
5.5E-06	-0.0009	0.00075	0.00074	0.00107	0.00014
5.6E-06	-9E-05	0.0007	-2E-05	-0.0003	0.0005
5.6E-06	0.00016	0.00076	-0.0008	0.00033	0.00076
5.6E-06	-0.0011	0.00126	-0.0004	0.00049	0.00064
5.7E-06	-0.0001	3.8E-05	-8E-05	-0.0003	0.00106
5.7E-06	-0.0005	0.00091	-0.0007	0.0006	0.00068
5.7E-06	-0.0013	0.00171	-7E-05	0.00064	8.9E-05

5.8E-06	-0.0012	0.00104	-0.0006	0.00045	0.00059
5.8E-06	-0.0003	-4E-06	-0.0003	-0.0002	0.00084
5.8E-06	-0.0003	0.00052	1E-05	0.00021	0.00056
5.9E-06	-0.0006	0.00056	0.00014	0.00028	0.00017
5.9E-06	-0.0005	0.00103	-0.0005	0.00072	-0.0006
5.9E-06	0.00012	0.00068	-0.0009	0.00075	0.00047
6E-06	-0.0002	0.00022	-0.0012	0.00091	4.7E-05
6E-06	-0.0008	0.00088	-0.0008	0.0004	0.00025
6E-06	-0.0009	0.00125	-0.0003	0.00076	0.00054
6E-06	-0.0002	0.00058	0.00015	0.00055	0.00038
6.1E-06	-0.0008	0.00116	-0.0008	0.00074	2.2E-05
6.1E-06	-0.0015	0.00082	0.00011	0.00032	0.00063
6.1E-06	-0.001	0.00084	-0.0002	0.00098	0.00061
6.2E-06	-0.0006	0.00118	-0.0016	0.00016	0.00064
6.2E-06	-0.001	0.00077	-0.0009	0.0004	-0.0001
6.2E-06	-0.0014	-0.0003	-0.001	0.00031	0.00026
6.3E-06	-0.0004	0.00062	-0.0003	0.00042	0.00033
6.3E-06	-0.0006	0.00102	-0.0012	0.0008	0.00042
6.3E-06	-0.0006	0.0017	8.5E-05	0.00102	0.00022
6.4E-06	-0.0006	0.00059	-0.0003	0.00059	0.00063
6.4E-06	-0.001	0.0017	-0.0009	0.00029	1.6E-05
6.4E-06	-0.001	0.00072	-4E-05	0.00064	0.00016
6.5E-06	-0.0007	-0.0004	-0.0004	0.00091	0.00059
6.5E-06	-0.0013	0.00112	-0.0011	0.00045	0.00054
6.5E-06	-0.0006	0.0005	-0.0007	0.00032	-0.0002
6.6E-06	-0.0009	0.00041	0.00022	2.9E-05	0.00054
6.6E-06	-0.0005	0.00078	9.9E-05	3.7E-05	0.00041
6.6E-06	-0.0006	0.00027	-0.0003	0.00034	0.0005
6.7E-06	-0.0013	0.00012	-0.0003	0.00044	0.00023
6.7E-06	-0.0004	-2E-05	-0.0008	0.00097	0.00018
6.7E-06	-0.0009	0.00038	-0.0007	-0.0002	0.0005
6.8E-06	-0.0011	0.00092	-0.0004	0.00058	0.00102
6.8E-06	-0.001	0.0004	-0.0006	0.00049	0.0006
6.8E-06	-0.0006	0.00094	-0.0011	0.00056	0.00093
6.8E-06	-0.0006	0.00123	-0.001	0.00101	0.00094
6.9E-06	-0.0016	0.00048	0.00019	-6E-05	0.0006
6.9E-06	-0.0006	0.00081	-0.0004	0.00127	8.8E-05
6.9E-06	-0.001	0.00034	0.00018	0.0012	0.00068
7E-06	-0.0003	0.00152	-0.0007	0.00043	0.00087
7E-06	-0.0009	0.0003	-0.0006	-9E-05	0.00057
7E-06	-0.0014	-0.0007	-0.0004	-0.0008	0.00044
7.1E-06	-0.0009	0.00095	-0.0015	4.4E-05	0.00047
7.1E-06	-0.0005	0.00076	-0.0002	0.00059	-0.0004
7.1E-06	-0.0002	0.00093	-0.001	0.0008	0.00055
7.2E-06	-0.0004	0.00148	-0.0004	-0.0004	0.00071
7.2E-06	-0.0007	0.00085	-0.0011	0.00035	0.00079
7.2E-06	-0.0011	0.00038	-0.0003	0.00046	0.00066
7.3E-06	-0.0007	0.00059	-0.0005	0.00127	0.00044
7.3E-06	-0.0009	0.00127	-0.0004	-0.0002	0.00047
7.3E-06	-0.0014	0.0014	-0.0003	0.00072	-0.0002
7.4E-06	-0.001	0.00076	0.00016	0.00013	0.00021
7.4E-06	-0.0014	0.00103	0.00044	0.00051	0.00116
7.4E-06	-0.001	0.00096	-0.0001	0.00061	0.00087
7.5E-06	-0.0011	0.00038	-5E-06	0.00061	0.00054

7.5E-06	-0.0008	0.00109	-0.0005	0.0012	-0.0004
7.5E-06	-0.0006	0.0006	-0.0007	8.3E-05	-3E-05
7.6E-06	-0.0014	0.00118	-0.0009	-0.0005	0.00046
7.6E-06	-0.0006	0.00076	-0.0008	0.00038	-0.0002
7.6E-06	-0.0003	0.00026	-9E-05	0.00068	0.00031
7.6E-06	-0.0009	0.00071	-1E-05	-1E-04	0.00053
7.7E-06	-0.0007	0.00105	-0.0008	-0.0004	-0.0003
7.7E-06	-0.0009	0.00085	-0.0002	-0.0005	0.00031
7.7E-06	-0.0009	0.00137	3.2E-05	0.00079	-0.0004
7.8E-06	-0.001	0.001	-0.0003	0.00108	0.00064
7.8E-06	-0.001	0.00081	0.00021	0.00074	0.00024
7.8E-06	-0.0007	0.00107	-0.0006	0.00025	0.0003
7.9E-06	-0.0017	0.00088	6.4E-05	0.00043	0.00026
7.9E-06	-0.0008	0.00057	-0.0005	0.00056	0.00015
7.9E-06	-0.0011	0.00076	-0.0006	0.0005	-0.0003
8E-06	-0.0009	0.00108	-4E-05	0.0007	0.00024
8E-06	-0.0008	0.00021	-2E-05	-0.0002	0.00022
8E-06	-0.001	0.00027	-0.0003	2.6E-05	-8E-05
8.1E-06	-0.0006	0.00079	0.00064	0.00068	0.00029
8.1E-06	-0.0013	0.00167	-0.0004	0.00099	0.0002
8.1E-06	-0.0016	0.0004	-0.0003	0.00033	-0.0003
8.2E-06	-0.001	0.0003	-0.0004	-0.0002	0.00012
8.2E-06	-0.0005	0.00109	0.0001	-0.0003	-0.0001
8.2E-06	-0.0013	0.00056	0.00031	-0.0003	0.00048
8.3E-06	-0.0006	8.1E-05	-0.0002	9.5E-05	0.00035
8.3E-06	-0.0008	0.00029	-0.0006	0.00108	0.00103
8.3E-06	-0.0013	0.00037	-0.0008	0.00016	0.00065
8.4E-06	-0.0007	0.00023	2E-05	0.00043	0.00022
8.4E-06	-0.0013	0.00059	-0.0004	0.00031	4.7E-06
8.4E-06	-0.0004	0.00064	-0.0003	0.0015	0.00011
8.4E-06	-0.001	0.00066	-0.0002	-0.0003	7.3E-05
8.5E-06	-0.0004	0.00082	-0.0007	0.00101	0.00036
8.5E-06	-0.0003	0.00071	-0.0005	0.0005	0.00033
8.5E-06	-0.0002	0.00105	-0.0002	0.00111	0.00077
8.6E-06	-0.0006	0.00045	9.1E-05	0.00096	3.9E-05
8.6E-06	-0.0007	0.00062	-0.0002	0.00041	-0.0007
8.6E-06	-0.0002	0.00101	-0.0003	0.0002	0.00053
8.7E-06	-0.001	0.00077	-0.0002	0.00017	0.00034
8.7E-06	-0.0013	-7E-05	-0.001	0.00122	0.00096
8.7E-06	-0.0002	0.00045	-0.001	0.00099	0.00015
8.8E-06	-0.0005	1.6E-05	-0.0002	0.00045	0.00021
8.8E-06	-0.0007	-0.0001	-0.0001	0.00074	8.2E-05
8.8E-06	-0.0012	0.00117	-0.0009	-0.0002	-0.0004
8.9E-06	-0.0008	0.00025	-0.0009	0.00051	-9E-05
8.9E-06	-0.0014	0.00061	-0.0004	8.8E-05	5.6E-05
8.9E-06	-5E-05	7.9E-05	-0.0006	0.0001	8E-05
9E-06	-0.001	0.00073	-3E-06	0.00106	0.00052
9E-06	-0.001	0.00062	-0.0009	0.0007	0.00093
9E-06	-0.0004	0.00111	-0.0017	0.00088	0.00031
9.1E-06	-0.0014	0.00057	-0.0003	0.0009	0.00046
9.1E-06	-0.0008	0.00049	-0.0009	-0.0001	0.00032
9.1E-06	-0.0016	0.00143	-0.0004	0.00035	-0.0003
9.2E-06	-0.0014	-8E-05	-0.0006	0.00063	-8E-05
9.2E-06	-0.0007	9.6E-05	-0.0012	0.00019	0.00024

9.2E-06	0.00016	0.00056	-0.0001	0.00044	0.00058
9.2E-06	-0.0008	0.00083	0.00016	0.00052	-0.0003
9.3E-06	-0.0008	0.00051	-0.0003	0.00072	-0.0005
9.3E-06	-0.0011	0.00105	-0.001	0.00107	0.00063
9.3E-06	-0.0003	0.00098	-0.0009	0.00026	-0.0002
9.4E-06	-0.001	0.00125	-0.0008	0.00019	-8E-05
9.4E-06	-0.0005	0.00049	-0.0005	0.00106	0.00085
9.4E-06	-0.0007	0.0004	6.3E-05	0.00089	-0.0003
9.5E-06	-0.0012	0.00056	-0.0005	0.00046	-0.0004
9.5E-06	-0.0004	0.00061	-0.0013	-0.0002	0.00079
9.5E-06	-0.0005	0.0005	-9E-05	0.00065	-0.0007
9.6E-06	-0.0005	-0.0001	-0.0004	0.00134	5.5E-05
9.6E-06	-0.0009	0.00071	-0.0004	0.00064	-2E-05
9.6E-06	-0.001	0.00093	-0.0007	0.00046	0.00066
9.7E-06	-0.0005	0.0011	-0.001	0.00064	0.00049
9.7E-06	-0.001	-3E-06	-0.0008	0.00158	0.00066
9.7E-06	-0.0012	0.00113	0.00014	-5E-06	8.8E-05
9.8E-06	-0.0003	0.00085	-0.0005	0.00075	-0.0004
9.8E-06	-0.0013	0.00079	0.00032	0.00022	-0.0002
9.8E-06	-0.001	0.00083	-0.0008	0.00056	0.00045
9.9E-06	-0.0008	0.00027	0.0001	0.00031	-0.0001
9.9E-06	-0.0016	0.00033	0.00012	0.0015	0.00079
9.9E-06	-0.0014	0.00034	0.00036	0.00095	0.00039
1E-05	-0.0015	0.001	-0.0012	0.00099	0.00042
1E-05	-0.0007	4.3E-05	-0.0007	0.00074	-7E-05
1E-05	-0.0007	0.00108	-0.001	0.00048	-0.0005
1E-05	-0.0013	0.00088	-0.0005	0.00035	0.00074
1E-05	-0.0011	0.00129	-0.0003	0.00111	0.0009
1E-05	-0.0003	2.5E-05	8.6E-05	0.00074	0.00059
1E-05	-0.001	0.00039	6.5E-05	0.00059	0.00043
1E-05	-0.0007	0.00037	-0.0017	0.00028	0.00074
1E-05	-0.0004	0.00143	-0.0003	0.00048	0.00105
1E-05	-0.0005	0.00105	-0.0005	0.00053	0.00053
1E-05	-0.0011	0.00112	-0.0011	-8E-05	8E-05
1E-05	-0.0005	0.00152	4E-05	0.00076	-4E-05
1E-05	-0.0006	0.00066	-0.0015	0.00084	6.2E-05
1E-05	-0.0008	0.00055	-0.0012	0.00082	-9E-05
1E-05	-0.0008	0.0001	-0.0005	0.00053	1.6E-05
1E-05	-0.0007	0.00057	-0.0013	0.00095	0.00014
1E-05	-0.0017	0.00032	-1E-04	-0.0005	-0.0002
1.1E-05	-0.001	0.0006	-5E-05	0.0012	0.00047
1.1E-05	-0.0003	0.00071	-0.0013	-0.0001	0.00087
1.1E-05	-0.0007	0.00053	-0.0006	9.9E-05	0.00034
1.1E-05	-0.0007	0.00059	-0.0014	0.00137	0.00053
1.1E-05	-0.0008	0.00113	-0.0011	0.00072	0.0003
1.1E-05	-0.0009	0.00071	-0.0004	0.00086	0.00073
1.1E-05	-0.0008	0.00102	-0.0007	-0.0002	0.00043
1.1E-05	-0.0015	0.0009	-0.0013	0.00036	-0.0004
1.1E-05	-0.001	0.00031	-0.0006	0.0001	0.00056
1.1E-05	-0.0005	0.00103	-0.0004	0.00039	-0.0001
1.1E-05	-0.0007	0.00054	-0.0004	0.00218	0.00146
1.1E-05	4.3E-05	0.00064	0.00019	0.00034	7.1E-05
1.1E-05	-0.0006	0.00074	-0.0002	0.00068	0.00012
1.1E-05	-0.0006	0.00144	-3E-05	0.001	0.0007

1.1E-05	0.00016	0.00084	-0.0004	0.00065	-0.0011
1.1E-05	-0.001	0.00018	-0.0002	0.00119	-0.0004
1.1E-05	-0.001	-5E-05	-0.0005	0.00157	-9E-05
1.1E-05	-0.0005	-0.0003	0.00051	0.00111	-0.0009
1.1E-05	-0.0004	0.00034	0.00105	0.00062	0.00052
1.1E-05	-0.0014	0.00063	0.00032	0.00074	0.00054
1.1E-05	-0.001	0.00035	-0.0005	0.00086	-0.0003
1.1E-05	-0.0006	0.00049	0.00047	0.00105	0.00054
1.1E-05	-0.0012	0.00051	-0.0003	0.00072	0.00047
1.1E-05	-0.0007	0.00089	0.0005	0.00143	0.00071
1.1E-05	-0.0015	0.00086	0.00025	0.00084	0.00026
1.1E-05	-0.0004	-6E-05	0.00071	0.00131	-0.0001
1.1E-05	-0.0011	0.00048	-8E-05	-0.0005	0.00019
1.1E-05	-0.0007	0.00018	0.00087	0.00071	0.00043
1.1E-05	-0.0001	0.00058	6.3E-05	0.00061	-8E-05
1.1E-05	-0.0009	0.00094	0.00011	0.00089	0.00042
1.1E-05	-0.0005	0.0004	-5E-05	0.0012	-0.0005
1.1E-05	-0.0004	0.00093	-0.0007	0.00088	0.0009
1.2E-05	-0.0005	0.00055	-0.0007	0.0007	-0.0003
1.2E-05	-0.0006	0.00047	7.1E-05	0.00061	0.00021
1.2E-05	-0.0004	0.00113	-0.0007	0.00047	0.00035
1.2E-05	-0.001	0.00145	2E-05	-0.0002	5.1E-05
1.2E-05	-0.0004	0.00099	-0.0006	0.00086	0.0002
1.2E-05	-0.0006	0.00039	-0.0004	0.00046	0.00013
1.2E-05	-0.0011	0.0005	0.00074	0.00059	0.00023
1.2E-05	-0.0011	3.2E-05	-0.0002	2.2E-05	0.00041
1.2E-05	-0.0002	0.00034	-0.0009	0.00076	-0.0009
1.2E-05	-0.0009	0.00125	-0.0017	0.00098	0.00073
1.2E-05	-0.0003	0.00098	0.00067	-2E-05	-0.0008
1.2E-05	-0.0002	0.0004	-0.0008	0.0009	0.00023
1.2E-05	-0.0004	-1E-04	-0.0005	0.0011	-0.0005
1.2E-05	-0.0004	0.00018	-0.0002	0.0014	0.00041
1.2E-05	-0.0004	6.2E-05	-1E-04	0.00109	-0.0004
1.2E-05	-0.0008	0.00083	0.00029	0.0007	0.00033
1.2E-05	-0.0006	0.00042	-0.0006	0.00098	-0.0001
1.2E-05	-0.0009	0.00077	-0.0008	0.00142	0.00046
1.2E-05	-0.0004	0.00068	-0.0013	0.00111	-0.0002
1.2E-05	-0.0006	0.00028	-0.0017	0.00067	-0.0007
1.2E-05	-0.0004	0.00084	-0.0002	0.00088	-0.0002
1.2E-05	-0.0006	0.00048	-0.0002	0.00068	4.9E-06
1.2E-05	-0.0007	0.00011	-0.0008	0.00051	-0.0003
1.2E-05	-0.0006	0.0012	0.00035	0.00028	-0.0003
1.2E-05	-0.0009	0.00047	-0.0008	0.00151	0.00013
1.2E-05	-0.0006	0.00146	-0.0001	0.0009	0.00028
1.2E-05	-0.0003	0.0007	-0.0006	0.00147	0.00038
1.2E-05	-0.0008	0.00057	-0.0005	0.0007	0.00044
1.2E-05	-9E-05	0.00011	-0.0003	0.00073	-0.001
1.2E-05	-0.0004	0.00025	0.00012	0.00141	-0.0003
1.2E-05	-0.0007	7.7E-05	-0.0002	0.00062	-0.0005
1.3E-05	-0.0002	0.00027	-0.0001	0.00056	-3E-05
1.3E-05	-0.0008	0.00145	0.00012	0.00053	0.00047
1.3E-05	-0.0002	0.0004	-0.0009	0.0013	0.00045
1.3E-05	-0.0008	0.00064	-2E-05	0.00085	0.00029
1.3E-05	-0.0007	0.00037	-0.0008	0.0018	2.3E-05

1.3E-05	-0.0005	0.00051	-0.0013	0.00136	1.4E-05
1.3E-05	-0.0006	0.00083	3.3E-05	0.00051	-0.0005
1.3E-05	-0.0008	0.00035	-0.0005	0.00089	-0.0003
1.3E-05	-0.0002	0.00038	-0.0003	0.00156	-0.0007
1.3E-05	-0.0004	0.0006	0.00012	0.00082	-9E-05
1.3E-05	-0.0003	0.00076	0.00028	0.00134	-0.0006
1.3E-05	-0.0011	0.00096	0.00032	0.00066	-0.0001
1.3E-05	-0.0008	0.0009	0.00028	0.00092	-9E-05
1.3E-05	-0.0009	0.00094	6E-05	0.00091	-0.0006
1.3E-05	-0.001	0.00065	-0.0007	0.00109	0.00078
1.3E-05	-0.0006	0.00139	-0.0006	0.00074	0.0004
1.3E-05	-0.0006	0.00072	-0.0005	-1E-05	0.00023
1.3E-05	-0.0006	0.00027	-0.0006	0.00041	9.2E-05
1.3E-05	-0.0006	0.00099	-0.0012	0.00052	5.7E-06
1.3E-05	-0.0003	0.00148	-6E-05	0.00097	-9E-05
1.3E-05	-0.0005	0.00125	-0.0009	0.00093	0.00019
1.3E-05	-0.0004	0.00044	0.00023	0.0013	-0.0004
1.3E-05	-0.0001	0.00052	-0.0004	0.001	0.00033
1.3E-05	-0.001	0.00043	-0.0004	0.00098	-0.0002
1.3E-05	-0.0008	0.00158	-0.0013	0.0009	-0.0004
1.3E-05	-0.0006	0.00107	-0.0002	0.00026	0.00047
1.3E-05	-0.0014	0.00078	-0.0003	0.00086	4.8E-05
1.3E-05	-0.0007	0.00065	-0.0001	0.00021	-0.0008
1.3E-05	-0.0006	0.0014	-0.0007	0.00029	0.00095
1.3E-05	-0.0009	0.00058	-0.0002	0.00103	0.00017
1.3E-05	-0.0003	0.0003	0.00029	0.0008	0.00019
1.4E-05	-8E-05	0.0006	-0.0004	0.00079	0.00035
1.4E-05	-0.0003	0.00079	-0.0005	0.00067	0.00094
1.4E-05	-0.0005	0.00113	-0.0004	0.00056	-0.0005
1.4E-05	-0.0007	0.00069	-0.0002	0.00013	0.00096
1.4E-05	-0.0015	0.00112	-0.001	0.00118	-0.0002
1.4E-05	0.0003	0.00106	3E-05	0.00137	0.00015
1.4E-05	-0.0008	0.0005	0.00045	0.00083	0.00043
1.4E-05	-0.0005	0.00053	-0.0005	0.00083	9.2E-05
1.4E-05	-0.0006	0.00123	-0.0005	0.00105	0.00048
1.4E-05	-0.0004	0.00185	0.00017	0.00094	2.6E-05
1.4E-05	-0.0003	0.00166	-0.0005	0.00049	-9E-05
1.4E-05	0.00023	0.00076	-0.0004	0.00141	0.00059
1.4E-05	-0.0007	0.00094	-0.0002	0.001	0.00037
1.4E-05	-0.0013	0.00095	-0.0002	0.00034	-2E-05
1.4E-05	-0.0012	0.00123	-0.0007	0.00118	0.00076
1.4E-05	-0.0009	0.00038	-0.0007	0.00123	0.00021
1.4E-05	-0.0008	-0.0001	-0.0006	0.00089	0.00115
1.4E-05	-0.0006	0.00016	0.00048	0.00089	-0.0004
1.4E-05	-0.0008	0.00042	-0.0007	0.0011	0.00056
1.4E-05	-0.0002	0.00147	0.00031	0.00067	9.2E-05
1.4E-05	1.2E-06	0.00105	-0.0004	0.00196	1.9E-05
1.4E-05	-0.0008	0.00083	-0.0005	0.00107	-8E-05
1.4E-05	-0.0008	9.6E-05	-0.0003	0.00136	0.00048
1.4E-05	-0.0006	0.00148	-0.0005	1.7E-05	0.00016
1.4E-05	-0.0007	0.00085	-0.0005	-4E-05	0.00028
1.4E-05	7.2E-05	0.00084	0.00036	1.2E-05	-0.0003
1.4E-05	-0.0005	0.00108	0.00041	0.00141	0.00058
1.4E-05	-0.0006	0.00094	-0.0008	0.00129	0.00014

1.4E-05	4.5E-05	0.00112	-0.0003	0.00145	0.00013
1.4E-05	-0.0007	0.00101	-0.0006	0.00142	0.00053
1.4E-05	-0.0005	-0.0003	-0.0008	0.00066	-0.0003
1.5E-05	-0.001	-3E-05	-0.0001	0.00076	7.9E-05
1.5E-05	-0.001	0.00115	-0.0009	0.00138	-0.0003
1.5E-05	-0.0006	0.00078	-0.0005	0.00075	3.8E-05
1.5E-05	8.8E-05	0.0008	-0.0008	0.00074	-0.0006
1.5E-05	-0.0008	0.00133	-0.0004	0.00106	-0.0005
1.5E-05	-0.001	0.00065	-0.0005	0.00068	-9E-05
1.5E-05	-0.0009	0.00066	-0.0007	0.00084	0.00019
1.5E-05	-0.0004	0.001	8.9E-05	0.00039	0.00114
1.5E-05	-0.0008	0.00105	-0.0004	0.00125	-3E-05
1.5E-05	-0.001	0.00078	-0.0007	0.00071	0.00059
1.5E-05	-0.0009	-8E-06	-0.0005	0.00105	0.00017
1.5E-05	-0.0006	0.00111	-0.0008	0.00111	0.00097
1.5E-05	-0.0006	0.00145	-0.0003	0.00095	-0.0002
1.5E-05	-0.0005	0.00086	-0.0002	0.00107	-0.0001
1.5E-05	-0.0001	0.00096	-0.0007	0.00153	-0.0004
1.5E-05	-0.0005	-0.0004	-0.0003	0.00094	0.00035

Table SI.11: Primary kinetic data for the reaction of 1-methyl-ε-caprolactam (**S11**) with CumO• generated by 355 nm LFP in MeCN (**Figure SI.11**).

t / s	ΔA 1-methyl-ε-caprolactam (S11)				
	**0.060M**	**0.157M**	**0.271M**	**0.436M**	**0.645M**
-1E-06	-0.0002	0.00021	-0.0007	0.00037	0.00048
-1E-06	-0.001	0.00045	-0.0007	0.00083	0.0006
-9E-07	-0.0001	0.00036	-0.001	0.00011	0.00135
-9E-07	-0.0001	6E-05	2.2E-05	0.00105	0.00085
-9E-07	0.0001	0.00029	-0.0001	-4E-05	0.00026
-8E-07	-0.0008	0.0007	-0.0006	0.00076	0.00094
-8E-07	0.00014	0.0005	-0.0005	0.00055	0.00069
-8E-07	0.00044	0.00088	-0.0003	0.00013	0.00051
-7E-07	-0.0005	0.00017	-0.0001	0.00011	0.00058
-7E-07	0.00021	0.00037	-0.0001	-3E-05	0.00082
-7E-07	4.1E-05	3.4E-05	-0.0008	0.00051	0.00113
-6E-07	-0.0002	-4E-05	-0.0002	-8E-05	0.00065
-6E-07	0.00015	-0.0002	-0.0007	0.00039	0.00094
-6E-07	-7E-05	0.00021	-0.0005	-0.0004	0.00048
-5E-07	-0.0002	5.9E-05	-0.0007	0.00029	0.00097
-5E-07	-7E-05	0.00041	-0.0005	0.0008	0.00066
-5E-07	-3E-05	6.4E-05	-0.0003	-1E-05	0.00031
-4E-07	0.00019	0.00045	3.7E-06	0.00069	0.00084
-4E-07	0.00032	0.00056	0.00053	0.00055	0.0005
-4E-07	0.00017	0.00069	-0.0002	0.00098	0.00095
-4E-07	-0.0005	0.00082	-0.0007	-0.0002	0.00132
-3E-07	0.00027	0.0007	-0.0004	-2E-05	0.00037
-3E-07	0.00011	-0.0003	-0.0009	0.00053	0.00097
-3E-07	-0.0007	0.0008	-0.0009	0.00067	0.00084
-2E-07	-9E-05	0.00087	-0.0005	0.00015	5.8E-05
-2E-07	-0.0008	0.00114	-0.0003	0.00053	0.00077
-2E-07	0.00025	0.00044	-0.0005	0.00014	0.00125
-1E-07	0.00047	0.00073	-0.0009	0.00012	0.00126
-1E-07	0.00033	0.00062	-0.0006	0.00081	0.00081
-6E-08	0.00013	0.00069	-0.0003	0.00081	0.00066
-3E-08	-0.0008	0.00118	-0.0005	0.00026	0.00023
-3E-10	-0.0032	-0.0016	-0.0038	-0.0026	-0.0031
3.2E-08	-0.0031	-0.0032	-0.0058	-0.0043	-0.0055
6.4E-08	0.02008	0.01979	0.01873	0.01876	0.01921
9.6E-08	0.01998	0.02008	0.01911	0.01842	0.0182
1.3E-07	0.01859	0.02	0.01762	0.01755	0.01676
1.6E-07	0.0182	0.01927	0.01673	0.01601	0.01563
1.9E-07	0.01758	0.01846	0.01653	0.01543	0.01493
2.2E-07	0.01719	0.01752	0.01606	0.01481	0.01349
2.6E-07	0.01698	0.01637	0.01579	0.01396	0.01313
2.9E-07	0.01631	0.01675	0.0145	0.01353	0.01224
3.2E-07	0.01603	0.01548	0.01294	0.01312	0.01059
3.5E-07	0.01542	0.01516	0.01224	0.01294	0.01113
3.8E-07	0.01485	0.0144	0.01253	0.01149	0.00957
4.2E-07	0.01474	0.01408	0.01135	0.01139	0.00992
4.5E-07	0.01401	0.01315	0.01134	0.01072	0.00862
4.8E-07	0.01285	0.01296	0.01058	0.01012	0.00814
5.1E-07	0.01262	0.01312	0.01044	0.00889	0.00728
5.4E-07	0.01234	0.01265	0.01011	0.00912	0.00769

5.8E-07	0.01287	0.01188	0.00914	0.00847	0.00659
6.1E-07	0.01287	0.01171	0.00905	0.00739	0.00742
6.4E-07	0.01205	0.01151	0.00772	0.00757	0.00683
6.7E-07	0.01226	0.01058	0.00815	0.007	0.00677
7E-07	0.01089	0.00999	0.00759	0.00642	0.0052
7.4E-07	0.01092	0.00915	0.00743	0.00715	0.00559
7.7E-07	0.01079	0.0094	0.00678	0.00568	0.00533
8E-07	0.01011	0.00959	0.00678	0.0057	0.00502
8.3E-07	0.00957	0.00928	0.00661	0.00609	0.00458
8.6E-07	0.00903	0.00915	0.00654	0.00544	0.00506
9E-07	0.00914	0.00796	0.00545	0.00491	0.00423
9.3E-07	0.00884	0.0082	0.00541	0.00473	0.00372
9.6E-07	0.0087	0.00814	0.00523	0.00537	0.00374
9.9E-07	0.0091	0.00691	0.00518	0.00502	0.00403
1E-06	0.00875	0.00678	0.00461	0.00419	0.0034
1.1E-06	0.00823	0.00676	0.00496	0.00376	0.00316
1.1E-06	0.00788	0.00743	0.00416	0.00371	0.0028
1.1E-06	0.00774	0.00657	0.00499	0.00338	0.00307
1.2E-06	0.00796	0.00704	0.00373	0.00306	0.00285
1.2E-06	0.0071	0.00703	0.0034	0.0035	0.00359
1.2E-06	0.00729	0.00593	0.00422	0.00324	0.00276
1.2E-06	0.00676	0.00659	0.00378	0.00358	0.00254
1.3E-06	0.00621	0.00555	0.00391	0.00283	0.00287
1.3E-06	0.00609	0.00636	0.00406	0.00284	0.00347
1.3E-06	0.00626	0.0061	0.00397	0.00262	0.00303
1.4E-06	0.00647	0.00556	0.00351	0.00229	0.00258
1.4E-06	0.00574	0.00493	0.00315	0.00317	0.00225
1.4E-06	0.00573	0.00537	0.00325	0.0021	0.00199
1.5E-06	0.00564	0.00528	0.00338	0.00262	0.00162
1.5E-06	0.00533	0.00561	0.003	0.00225	0.00307
1.5E-06	0.00631	0.00451	0.00333	0.00274	0.00249
1.6E-06	0.00504	0.00465	0.00272	0.00249	0.00289
1.6E-06	0.00568	0.00519	0.00305	0.00215	0.00241
1.6E-06	0.00505	0.00393	0.00232	0.00189	0.00299
1.7E-06	0.0052	0.00412	0.00177	0.00221	0.00326
1.7E-06	0.00515	0.00428	0.0026	0.00274	0.00217
1.7E-06	0.00506	0.00492	0.00181	0.00161	0.00245
1.8E-06	0.00469	0.0038	0.00256	0.00169	0.00183
1.8E-06	0.00473	0.00392	0.00223	0.00211	0.0021
1.8E-06	0.00466	0.00363	0.00226	0.00202	0.00236
1.9E-06	0.0046	0.00372	0.00206	0.00088	0.00187
1.9E-06	0.00361	0.00472	0.00201	0.00202	0.00128
1.9E-06	0.00392	0.00309	0.00264	0.00166	0.00189
2E-06	0.00432	0.00438	0.00191	0.00186	0.00241
2E-06	0.00408	0.00401	0.0019	0.00128	0.0029
2E-06	0.00405	0.00372	0.00149	0.00128	0.00177
2E-06	0.00416	0.00246	0.00197	0.00111	0.00164
2.1E-06	0.00342	0.00247	0.00158	0.00136	0.00111
2.1E-06	0.00322	0.00312	0.00211	0.00207	0.00238
2.1E-06	0.00352	0.0031	0.00226	0.00175	0.00161
2.2E-06	0.00377	0.00392	0.00208	0.00093	0.00174
2.2E-06	0.0029	0.00301	0.00144	0.00197	0.00151
2.2E-06	0.00298	0.00343	0.00216	0.00085	0.00149
2.3E-06	0.00293	0.00321	0.00205	0.00106	0.0013

2.3E-06	0.00305	0.00327	0.00193	0.00098	0.00133
2.3E-06	0.00338	0.00229	0.00115	0.00176	0.00172
2.4E-06	0.00286	0.00255	0.00048	0.00154	0.00193
2.4E-06	0.00311	0.00251	0.00117	0.00094	0.00144
2.4E-06	0.00281	0.00234	0.00167	0.00095	0.00172
2.5E-06	0.00215	0.00253	0.00173	0.00128	0.00137
2.5E-06	0.00323	0.00253	0.00118	0.00103	0.00196
2.5E-06	0.00243	0.00262	0.00079	0.00091	0.00183
2.6E-06	0.00249	0.00312	0.00154	0.00107	0.00291
2.6E-06	0.00297	0.00289	0.00172	0.00159	0.00192
2.6E-06	0.00258	0.00252	0.00128	0.00098	0.00194
2.7E-06	0.00224	0.00276	0.00147	0.00181	0.00176
2.7E-06	0.00227	0.0029	0.00114	0.00108	0.00161
2.7E-06	0.00254	0.0025	0.00147	0.00096	0.00167
2.8E-06	0.00201	0.00206	0.00216	0.00161	0.00266
2.8E-06	0.00208	0.00244	0.00161	0.00159	0.00213
2.8E-06	0.0025	0.00239	0.0008	0.00173	0.00208
2.8E-06	0.00225	0.00188	0.00153	0.00099	0.00137
2.9E-06	0.0021	0.0022	0.00099	0.00074	0.00162
2.9E-06	0.00252	0.00197	0.00065	0.00093	0.00149
2.9E-06	0.00168	0.0017	0.00195	0.00041	0.00171
3E-06	0.00248	0.00271	0.00156	0.00092	0.0019
3E-06	0.00221	0.00184	0.0012	0.00135	0.00146
3E-06	0.00182	0.00162	7.1E-05	0.0008	0.00198
3.1E-06	0.0019	0.00252	0.00094	0.00115	0.00124
3.1E-06	0.00123	0.00187	0.00198	0.00106	0.00272
3.1E-06	0.00221	0.00207	0.00055	0.00091	0.00163
3.2E-06	0.0014	0.00258	0.00063	0.00055	0.00178
3.2E-06	0.00262	0.00168	0.00027	0.0007	0.00246
3.2E-06	0.00176	0.00224	0.00116	0.00132	0.0022
3.3E-06	0.00155	0.00229	0.00096	0.00093	0.00115
3.3E-06	0.00167	0.00187	0.001	0.001	0.00125
3.3E-06	0.00187	0.00133	0.00112	0.00067	0.00135
3.4E-06	0.0025	0.00223	0.00144	0.00098	0.00109
3.4E-06	0.00168	0.00213	0.00069	0.00042	0.00162
3.4E-06	0.0012	0.00108	0.00099	0.00137	0.00131
3.5E-06	0.00101	0.00212	0.00015	0.00123	0.00181
3.5E-06	0.00109	0.00229	0.00079	0.00137	0.00148
3.5E-06	0.0019	0.00157	0.00121	0.00109	0.00177
3.6E-06	0.00169	0.00265	0.00044	0.00146	0.00282
3.6E-06	0.00114	0.00227	0.00039	0.00115	0.00152
3.6E-06	0.00158	0.00229	0.0001	0.00046	0.00126
3.6E-06	0.0017	0.00108	-0.0004	0.001	0.00188
3.7E-06	0.00104	0.00066	0.00019	0.00032	0.00145
3.7E-06	0.00111	0.00203	0.00085	0.00036	0.00125
3.7E-06	0.00098	0.00126	0.0003	0.00081	0.00268
3.8E-06	0.00017	0.00229	0.00057	0.00078	0.00116
3.8E-06	0.00067	0.0012	0.00105	0.00098	0.00198
3.8E-06	0.00114	0.00198	0.00081	0.00074	0.002
3.9E-06	0.00116	0.00143	0.00058	0.00094	0.00112
3.9E-06	0.00099	0.00084	8.9E-05	0.00092	0.00209
3.9E-06	0.00063	0.00171	0.0009	0.0007	0.00118
4E-06	0.00094	0.0015	0.00033	0.00125	0.00123
4E-06	0.00091	0.00108	-5E-05	0.00054	0.00182

4E-06	0.00115	0.00148	0.00033	0.00067	0.0011
4.1E-06	0.00076	0.00212	0.00038	0.0017	0.00202
4.1E-06	0.00034	0.00162	0.00036	0.00085	0.00173
4.1E-06	0.00127	0.00215	0.00032	0.00135	0.00097
4.2E-06	0.00122	0.00208	0.00036	0.00089	0.00127
4.2E-06	0.0018	0.00156	0.00013	0.00098	0.00145
4.2E-06	0.00153	0.00106	8.3E-05	0.00088	0.00183
4.3E-06	0.00092	0.00147	8E-05	0.00109	0.00185
4.3E-06	0.00078	0.00079	0.00126	0.00068	0.00164
4.3E-06	0.00084	0.0018	0.00044	0.0003	0.00109
4.4E-06	0.00092	0.00136	0.00037	0.0007	0.00169
4.4E-06	0.00051	0.00191	0.0005	0.00085	0.00103
4.4E-06	0.00078	0.00146	0.00104	0.00055	0.00162
4.4E-06	0.00127	0.00217	0.00101	0.00115	0.00107
4.5E-06	0.0004	0.00238	0.00096	0.00103	0.00178
4.5E-06	0.00073	0.00224	0.00036	0.00064	0.00237
4.5E-06	0.00073	0.00128	-0.0003	0.001	0.00114
4.6E-06	0.00103	0.00113	-0.0001	0.00093	0.00136
4.6E-06	0.00068	0.00171	0.00051	0.00111	0.00257
4.6E-06	0.00057	0.00154	0.00037	0.00075	0.00182
4.7E-06	0.00094	0.0018	0.00073	0.0004	0.00184
4.7E-06	0.00136	0.00153	0.00018	0.00071	0.00069
4.7E-06	0.00036	0.00164	0.00016	0.00063	0.00156
4.8E-06	0.00055	0.00071	-0.0002	0.00061	0.00126
4.8E-06	0.00046	0.00077	-7E-05	0.00076	0.00118
4.8E-06	0.00023	0.00118	0.0003	0.00033	0.00157
4.9E-06	0.0008	0.00156	0.00073	0.00113	0.00164
4.9E-06	0.00034	0.00164	9E-05	0.00097	0.00195
4.9E-06	0.00049	0.00117	0.00044	0.00094	0.00156
5E-06	0.00046	0.00123	0.00072	0.0009	0.0022
5E-06	0.0013	0.00139	0.00056	0.00157	0.00135
5E-06	0.0006	0.00082	0.00105	0.00104	0.0013
5.1E-06	0.00103	0.0018	-5E-05	0.00032	0.00119
5.1E-06	0.0006	0.00144	0.00057	0.00122	0.00183
5.1E-06	0.00043	0.00123	0.00058	0.00161	0.00185
5.2E-06	0.00043	0.00092	0.00059	0.00109	0.00213
5.2E-06	0.00111	0.0012	0.00113	0.00117	0.00193
5.2E-06	0.00082	0.00185	0.0004	0.00165	0.00164
5.2E-06	0.00032	0.00153	-6E-05	0.0007	0.00149
5.3E-06	0.00059	0.00101	1.1E-05	0.00141	0.00187
5.3E-06	0.00128	0.00144	-0.0002	0.00115	0.0019
5.3E-06	0.00133	0.00128	0.00065	0.00072	0.00117
5.4E-06	0.00055	0.0016	0.00071	0.00124	0.00171
5.4E-06	0.00068	0.00143	-0.0008	0.00127	0.0021
5.4E-06	0.00098	0.00119	-1E-05	0.00045	0.00256
5.5E-06	0.00058	0.00145	0.0014	0.00075	0.00199
5.5E-06	0.00065	0.00137	0.00044	0.00074	0.00146
5.5E-06	0.00073	0.0011	0.00019	0.001	0.0016
5.6E-06	0.00067	0.00142	0.00089	0.00135	0.00186
5.6E-06	0.00038	0.0009	0.0006	0.00089	0.00148
5.6E-06	0.00119	0.00058	0.00028	0.00069	0.00164
5.7E-06	0.00078	0.0016	-3E-05	0.00054	0.00165
5.7E-06	0.00057	0.00127	0.00099	0.00091	0.00087
5.7E-06	0.00038	0.00162	4.1E-05	-8E-05	0.00144

5.8E-06	0.00046	0.00149	0.00106	0.00069	0.00195
5.8E-06	0.00033	0.00148	0.00046	0.00092	0.00135
5.8E-06	0.00068	0.00113	0.0011	0.00088	0.00118
5.9E-06	0.00047	0.00145	0.00079	0.00039	0.00176
5.9E-06	0.00057	0.00155	0.0009	0.00105	0.00215
5.9E-06	2.1E-05	0.00118	8E-05	0.00129	0.00181
6E-06	0.0005	0.00087	-7E-05	0.00105	0.00167
6E-06	0.0006	0.00067	0.00077	0.00104	0.00089
6E-06	0.00054	0.00183	-3E-05	2.7E-05	0.00171
6E-06	0.00034	0.00114	9.6E-05	0.00078	0.00158
6.1E-06	0.00052	0.00196	0.0004	0.00133	0.00168
6.1E-06	0.00095	0.0016	0.00014	0.0004	0.00101
6.1E-06	0.00087	0.00087	0.00093	0.00108	0.00147
6.2E-06	0.0007	0.00103	0.00021	0.00011	0.00055
6.2E-06	0.00051	0.00089	-0.0004	0.0005	0.00116
6.2E-06	0.00085	0.00117	0.00072	-0.0007	0.00151
6.3E-06	0.00056	0.00089	0.00026	0.00059	0.00147
6.3E-06	0.00031	0.00118	0.00045	0.00174	0.00182
6.3E-06	-5E-07	0.00207	4.2E-05	0.0012	0.0018
6.4E-06	-5E-05	0.00104	0.00056	0.0009	0.00113
6.4E-06	0.00018	0.00121	0.00066	0.00061	0.00074
6.4E-06	-3E-05	0.00119	0.00078	0.00127	0.00149
6.5E-06	-0.0002	0.00099	0.00099	0.00139	0.0017
6.5E-06	0.00018	0.0011	0.0005	0.00162	0.00149
6.5E-06	0.00013	0.00081	0.00042	0.001	0.00169
6.6E-06	0.0004	0.00115	0.0005	0.0011	0.00163
6.6E-06	0.00059	0.00106	-0.0005	0.00062	0.00126
6.6E-06	0.00027	0.00057	9.7E-05	0.00097	0.00199
6.7E-06	-0.0001	0.00073	0.00084	0.0009	0.00168
6.7E-06	-0.0007	0.00092	0.00058	0.00065	0.00155
6.7E-06	0.00035	0.00095	5.2E-06	0.00136	0.00144
6.8E-06	-0.0006	0.0006	0.00138	0.00129	0.00178
6.8E-06	0.0004	0.00152	-0.0001	0.00054	0.00137
6.8E-06	0.00024	0.00118	0.00026	0.00033	0.00135
6.8E-06	-2E-05	0.00117	3.4E-05	0.00056	0.00089
6.9E-06	-8E-05	0.00097	0.00049	0.00152	0.00122
6.9E-06	0.00041	0.00102	0.00068	0.00023	0.00102
6.9E-06	0.00028	0.00129	0.00084	-0.0003	0.00107
7E-06	0.00052	0.00097	0.00032	0.00064	0.00096
7E-06	0.00013	0.00106	0.0009	0.00095	0.00137
7E-06	0.00022	0.00132	0.00052	0.00192	0.00208
7.1E-06	0.00059	0.00087	-4E-06	0.00057	0.00083
7.1E-06	-0.0001	0.00141	0.00075	0.00091	0.00102
7.1E-06	0.00044	0.00085	0.00055	0.00102	0.00169
7.2E-06	0.00028	0.00106	0.00029	0.00037	0.00135
7.2E-06	0.00068	0.0015	0.00047	0.00105	0.00165
7.2E-06	8.5E-05	0.00105	0.00093	0.00099	0.00092
7.3E-06	0.00046	0.00113	0.00011	0.00133	0.0014
7.3E-06	0.00129	0.00201	0.00135	0.00025	0.0019
7.3E-06	0.00079	0.00157	0.00056	0.00069	0.00146
7.4E-06	-0.0004	0.00109	0.00038	0.00052	0.00159
7.4E-06	-0.0005	0.00104	0.0008	0.00102	0.00138
7.4E-06	1.9E-05	0.00122	0.00065	0.00114	0.00205
7.5E-06	0.00062	0.00078	0.001	0.00137	0.00088

7.5E-06	0.00077	0.0007	0.00048	0.00189	0.0014
7.5E-06	0.00028	0.00018	9.7E-05	0.0016	0.00153
7.6E-06	0.00022	0.00158	-0.0003	0.0011	0.00211
7.6E-06	9.1E-05	0.00131	0.00047	0.0007	0.00162
7.6E-06	0.00015	0.00144	0.00039	0.0016	0.00149
7.6E-06	0.00125	0.00106	0.00056	0.00175	0.00153
7.7E-06	-0.0009	0.00096	0.0009	0.00062	0.00134
7.7E-06	-0.0004	0.00123	0.00117	-8E-05	0.00237
7.7E-06	0.00044	0.00126	0.0004	0.00202	0.00121
7.8E-06	-0.0005	0.00156	0.00112	0.0004	0.00176
7.8E-06	0.00037	0.00133	0.00074	0.00144	0.00154
7.8E-06	3E-05	0.00123	0.00104	0.0005	0.0019
7.9E-06	-0.0002	0.00175	0.00068	0.00043	0.00163
7.9E-06	0.00011	0.00145	0.00057	0.00132	0.00113
7.9E-06	-1E-05	0.00117	0.0002	0.00117	0.00176
8E-06	0.00013	0.00079	-0.0002	0.00138	0.00134
8E-06	0.00018	0.00132	0.00082	0.0018	0.00101
8E-06	-0.0004	0.00073	0.00056	0.00162	0.00136
8.1E-06	0.00036	0.00067	0.00046	0.00088	0.00145
8.1E-06	0.0005	0.00117	0.00026	0.00096	0.00058
8.1E-06	-0.0005	0.00116	-4E-05	0.00084	0.00073
8.2E-06	0.00061	0.00119	0.00023	0.00221	0.00111
8.2E-06	0.00066	0.00135	0.00087	0.00039	0.00055
8.2E-06	0.00057	0.00101	0.00029	0.00094	0.00121
8.3E-06	0.00052	0.001	-9E-05	0.00107	0.00138
8.3E-06	6.7E-05	0.00061	0.00028	0.00023	0.00142
8.3E-06	-0.0001	0.00151	0.00025	0.00046	0.00162
8.4E-06	-0.0004	0.00121	0.00086	0.00167	0.00111
8.4E-06	-0.0003	0.00103	0.00038	0.00129	0.0017
8.4E-06	0.00103	0.0015	0.00085	0.00093	0.00113
8.4E-06	0.00035	0.00127	0.00062	0.0006	0.0013
8.5E-06	0.00036	0.00119	0.00034	0.0005	0.00159
8.5E-06	-0.0003	0.00061	0.00071	0.00149	0.00097
8.5E-06	9.2E-05	0.00122	0.0004	0.00014	0.00101
8.6E-06	-0.0004	0.00117	0.00041	0.00052	0.0013
8.6E-06	2.2E-05	0.00069	0.00061	0.00025	0.00098
8.6E-06	-0.0001	0.00086	-0.0005	0.0008	0.00166
8.7E-06	0.00061	0.00085	0.00059	-9E-06	0.00183
8.7E-06	0.00033	3.8E-05	0.00052	0.00099	0.00075
8.7E-06	0.00018	0.00136	0.00052	0.00027	0.00148
8.8E-06	0.00011	0.00117	0.00038	0.00047	0.00134
8.8E-06	0.00012	0.00121	0.00075	0.00033	0.00136
8.8E-06	-0.0001	0.00104	0.00029	0.00029	0.00184
8.9E-06	-9E-05	0.00098	0.00024	0.00071	0.00086
8.9E-06	0.00056	0.00086	-0.0002	0.00031	0.00067
8.9E-06	0.00092	0.00102	-0.0003	0.00099	0.00176
9E-06	0.00078	0.00096	-0.0002	0.00047	0.00118
9E-06	0.00065	0.00096	-0.0007	0.00085	0.0015
9E-06	0.00065	0.00088	9.8E-05	0.00087	0.00189
9.1E-06	0.00048	0.00129	0.00063	0.00102	0.00152
9.1E-06	0.00062	0.00125	0.00054	-0.0009	0.00169
9.1E-06	0.00049	0.00104	6.1E-05	1.4E-05	0.00108
9.2E-06	-0.0002	0.00136	-0.0002	-0.0002	0.001
9.2E-06	-5E-05	0.00067	2.9E-05	0.00014	0.00066

9.2E-06	0.00037	0.00084	0.00045	0.00122	-0.0001
9.2E-06	-0.0004	0.00187	0.00073	0.00022	0.0005
9.3E-06	-0.0002	0.00124	0.00039	0.00043	0.00052
9.3E-06	-0.0007	0.00117	0.00039	-0.0002	0.00133
9.3E-06	-0.0005	0.00031	0.00065	0.00155	0.0013
9.4E-06	0.0008	0.00095	0.00032	0.0006	0.00142
9.4E-06	0.00055	0.00058	0.00029	0.00014	0.00154
9.4E-06	-0.0003	0.00127	0.0004	0.001	0.00144
9.5E-06	-0.0004	0.00108	0.00026	3.7E-05	0.00022
9.5E-06	-8E-05	0.00152	-0.0002	0.00013	0.00088
9.5E-06	-0.0009	0.00112	0.00073	0.00051	0.00107
9.6E-06	-0.0002	0.00101	0.00044	0.00016	0.0015
9.6E-06	-0.0002	0.00052	-0.0003	0.00044	0.00166
9.6E-06	-6E-05	0.00056	-0.0003	6.2E-05	0.00137
9.7E-06	-3E-05	0.00056	0.0006	0.00023	0.00158
9.7E-06	0.00027	0.00079	0.00044	0.00121	0.00224
9.7E-06	0.00029	0.00154	3.8E-05	0.00097	0.00106
9.8E-06	9.2E-05	0.00058	5.5E-05	0.00046	0.00189
9.8E-06	-0.0004	0.00094	0.00029	-7E-05	0.00156
9.8E-06	0.00016	0.00147	0.00033	0.00081	0.00108
9.9E-06	-1E-04	0.00078	0.00021	0.00132	0.00111
9.9E-06	-0.0004	0.00048	0.00047	0.00132	0.00108
9.9E-06	-0.0002	0.00047	-0.0006	0.00126	0.00115
1E-05	-0.0003	0.00058	0.00087	0.00091	0.00098
1E-05	-0.0007	0.00087	0.00012	0.00084	0.00185
1E-05	-0.0004	0.001	-0.0002	-0.0002	6.7E-05
1E-05	0.00013	0.00123	3.2E-05	-0.0002	0.00131
1E-05	-0.0006	0.0004	0.00057	0.00091	0.00158
1E-05	4.1E-06	0.00092	0.00045	0.00128	0.00164
1E-05	-0.0003	0.00095	-0.0001	0.00131	0.00166
1E-05	-0.0003	0.00098	-9E-05	0.00108	0.00212
1E-05	8.2E-05	0.00027	-0.0006	0.00142	0.0011
1E-05	-1E-05	0.00046	-0.0005	0.00137	0.00069
1E-05	-0.0004	0.00156	0.00047	0.00117	0.00133
1E-05	-0.0009	0.00104	-6E-05	0.00061	0.00189
1E-05	-0.0001	0.00122	7.7E-05	0.00089	0.00151
1E-05	-0.0006	0.00127	0.00013	0.00089	0.00103
1E-05	0.00054	0.00107	0.00036	0.00165	0.00156
1E-05	0.00106	0.0009	6.3E-05	0.00067	0.00113
1E-05	8.8E-05	0.00103	-0.0004	0.00084	0.00062
1.1E-05	4.5E-05	0.00103	-0.0005	0.00046	0.00164
1.1E-05	-4E-06	0.00133	0.00049	0.00078	0.00135
1.1E-05	-0.0002	0.00088	-0.0001	-0.0003	0.00094
1.1E-05	0.00012	0.00122	7E-05	0.00101	0.00136
1.1E-05	1.2E-05	0.00113	6.2E-05	0.00025	0.0009
1.1E-05	-0.0004	0.00087	0.00019	0.00035	0.00082
1.1E-05	-0.0004	0.00127	-0.0002	0.00031	0.00141
1.1E-05	-0.0005	0.00128	-0.0005	0.00103	0.00155
1.1E-05	0.00016	0.0014	-0.0004	0.00038	0.0013
1.1E-05	0.00021	0.00098	-9E-05	0.00116	0.00105
1.1E-05	-4E-05	0.00091	0.00093	0.00143	0.00082
1.1E-05	0.00047	0.00088	0.00023	0.00058	0.00059
1.1E-05	0.00042	0.00089	0.00013	0.00117	0.0019
1.1E-05	0.00052	0.00092	0.0009	0.00126	0.00092

1.1E-05	3.7E-05	0.0007	0.00018	0.00118	0.00226
1.1E-05	0.00047	0.00149	0.00014	0.00055	0.00039
1.1E-05	0.00038	0.00016	1.1E-05	0.00102	0.0002
1.1E-05	0.00022	0.00051	-0.0005	0.00058	0.00084
1.1E-05	7.5E-05	0.00109	9.9E-07	0.00042	0.00119
1.1E-05	-0.0004	0.00103	0.0008	0.00041	0.00115
1.1E-05	0.00026	0.00079	-0.0002	0.00116	0.00117
1.1E-05	0.00015	0.00084	0.00034	0.00084	0.00074
1.1E-05	0.0004	0.00065	0.00022	0.0008	0.00226
1.1E-05	-0.0003	0.00114	0.00024	0.00019	0.00132
1.1E-05	-7E-05	0.00066	0.00046	0.00144	0.00128
1.1E-05	-3E-05	0.00145	-0.0002	0.00059	0.00091
1.1E-05	0.00018	0.00108	0.00045	0.00131	0.00101
1.1E-05	-0.0003	0.00075	0.00087	0.00155	0.00175
1.1E-05	0.00025	0.00095	0.00047	0.00125	0.0012
1.1E-05	-0.0002	0.00121	0.00015	0.00096	0.00153
1.1E-05	-2E-05	0.00087	-0.0002	0.00043	0.00136
1.1E-05	-0.0001	0.00071	0.00095	0.00058	0.00078
1.2E-05	-0.0005	0.00124	0.00079	0.00172	0.00108
1.2E-05	-0.0002	0.00157	6.5E-05	0.00061	0.0013
1.2E-05	-0.0001	0.00052	0.00022	0.00102	0.00122
1.2E-05	-0.0004	0.00097	-8E-05	0.0009	0.00113
1.2E-05	0.00031	0.00128	4E-05	0.00067	0.00142
1.2E-05	0.00013	0.00088	0.00089	0.00011	0.00179
1.2E-05	-0.0006	0.00071	0.0003	0.00062	0.00162
1.2E-05	-0.0006	0.00142	-0.0003	0.00138	0.00075
1.2E-05	2.7E-05	0.00092	-0.0007	0.00151	0.0007
1.2E-05	0.0003	0.0017	0.00059	0.00159	0.00132
1.2E-05	-0.0002	0.00113	0.0002	0.00055	0.00114
1.2E-05	-0.0001	0.00105	0.00067	0.00149	0.00143
1.2E-05	-6E-05	0.00146	-2E-05	0.00149	0.00086
1.2E-05	9.7E-05	0.00171	0.00038	0.00014	0.00106
1.2E-05	0.00018	0.00082	-0.0006	0.00139	0.00097
1.2E-05	-0.0003	0.00106	0.00012	0.00049	0.00113
1.2E-05	0.0001	0.00136	0.0005	0.0012	0.00163
1.2E-05	-0.0007	0.00076	0.00115	0.00103	0.00158
1.2E-05	0.00017	0.00166	0.00025	0.00074	0.00096
1.2E-05	-0.0002	0.00121	0.00024	0.00085	0.00055
1.2E-05	-0.0002	0.00105	0.00016	0.00141	0.00116
1.2E-05	-0.0003	0.00068	0.00073	0.00116	0.00206
1.2E-05	0.00055	0.00089	-6E-06	0.00106	0.00173
1.2E-05	0.00031	0.00092	-0.0002	0.00089	0.00173
1.2E-05	0.00047	0.00143	-0.0001	0.00085	0.00078
1.2E-05	0.00023	0.00093	0.00054	0.00148	0.00162
1.2E-05	-0.0002	0.00089	0.00072	0.00088	0.00163
1.2E-05	-0.0004	0.00058	0.00043	0.00134	0.00108
1.2E-05	0.00023	0.00074	0.00031	0.00063	0.00109
1.2E-05	0.00018	0.00079	3.3E-05	0.00016	0.00151
1.2E-05	0.00093	0.00085	-0.0008	0.00065	0.00114
1.3E-05	0.00067	0.00134	0.00039	0.00126	0.00077
1.3E-05	-0.0003	0.00036	0.00039	0.00104	0.00138
1.3E-05	0.00035	0.00151	0.00025	0.00048	0.0005
1.3E-05	-0.0004	0.00086	7.4E-05	0.00105	0.00115
1.3E-05	0.00057	0.00055	-0.0005	0.00149	0.00166

1.3E-05	-0.0001	0.00113	-0.0002	0.00108	0.00153
1.3E-05	-0.0002	0.00093	0.00036	0.00111	0.00088
1.3E-05	0.0007	0.00091	0.00024	0.00097	0.00169
1.3E-05	0.00011	0.00161	-0.0003	0.00122	0.00069
1.3E-05	0.00017	0.00108	-0.0004	0.00109	0.0016
1.3E-05	0.00044	0.00042	0.00018	0.00047	0.0017
1.3E-05	-0.0004	0.00111	0.00076	0.00135	0.00143
1.3E-05	0.00016	0.00152	0.00029	0.0008	0.00122
1.3E-05	-0.0003	0.00126	0.00068	0.00012	0.00125
1.3E-05	0.00053	0.00087	0.00038	0.00047	0.00112
1.3E-05	8.1E-05	0.00091	0.00054	0.00097	0.00134
1.3E-05	-7E-05	0.00096	-0.0002	0.00114	0.00056
1.3E-05	-7E-05	0.00089	-0.0003	0.00105	0.00126
1.3E-05	-0.0003	0.00104	0.00055	0.00133	0.00105
1.3E-05	0.0002	0.00141	0.0003	0.0011	0.00252
1.3E-05	0.00014	0.00124	-0.0004	0.00105	0.00167
1.3E-05	0.00056	0.00116	0.00013	0.00107	0.00137
1.3E-05	-0.0005	0.00119	0.00022	0.00065	0.00113
1.3E-05	-6E-05	0.00087	0.00011	3.8E-05	0.00095
1.3E-05	-0.0003	0.00165	0.00047	0.00108	0.00154
1.3E-05	0.00036	0.0011	-0.0003	0.00093	0.00166
1.3E-05	-0.0001	0.00126	-0.0004	0.00088	0.00106
1.3E-05	-0.0001	0.00101	0.00018	0.00095	0.00152
1.3E-05	0.00034	0.00095	0.0005	0.00115	0.00131
1.3E-05	-0.0006	0.0012	-0.0002	-1E-04	0.00086
1.3E-05	-0.0006	0.00116	0.00011	0.00058	0.00108
1.4E-05	-7E-05	0.00065	0.00045	0.00176	0.0015
1.4E-05	-2E-06	0.00134	5.3E-05	0.00123	0.00175
1.4E-05	-0.0002	0.00118	-0.0002	0.00158	0.00189
1.4E-05	-0.0001	0.00112	-0.0002	-3E-05	0.00171
1.4E-05	0.00022	0.00134	1.9E-05	0.00021	0.00163
1.4E-05	0.00014	0.00104	0.00023	0.00104	0.00176
1.4E-05	-0.0001	0.00142	0.00043	-0.0002	0.00087
1.4E-05	0.00122	0.00117	-0.0002	0.00083	0.00151
1.4E-05	0.00053	0.00142	0.00015	0.00064	0.0005
1.4E-05	-5E-05	0.00113	-0.0003	0.0007	0.00162
1.4E-05	0.0003	0.00112	-0.0001	0.00124	0.00165
1.4E-05	-0.0007	0.00154	5.4E-05	0.00169	0.00242
1.4E-05	1E-05	0.00097	-0.0004	0.00125	0.00136
1.4E-05	-7E-06	0.00092	0.00044	-4E-05	0.00143
1.4E-05	-0.0004	0.00106	0.00037	0.00039	0.00092
1.4E-05	-0.0005	0.00107	-0.0009	0.00023	0.00163
1.4E-05	-0.0007	0.00089	0.00055	0.00148	0.0012
1.4E-05	-0.0005	0.0011	0.00019	0.00079	0.00124
1.4E-05	-0.0009	0.00076	0.00055	0.0005	0.00214
1.4E-05	0.00031	0.00126	0.00028	0.00084	0.00181
1.4E-05	-0.0007	0.00117	0.00052	0.00042	0.00151
1.4E-05	-0.0007	0.00146	-3E-05	0.00049	0.00129
1.4E-05	-0.0004	0.00076	0.00027	0.00074	0.00211
1.4E-05	-0.0006	0.00026	0.00087	0.00118	0.00111
1.4E-05	-0.0001	0.00096	0.00043	0.0012	0.00092
1.4E-05	-6E-05	0.0012	0.00022	0.00157	0.00157
1.4E-05	-0.0002	0.00116	0.00064	0.00084	0.00079
1.4E-05	-0.0009	0.00071	-0.0003	0.00149	0.00089

1.4E-05	-0.0005	0.00125	0.00014	0.00152	0.00083
1.4E-05	-8E-05	0.00093	0.00064	-0.0004	0.00135
1.4E-05	-8E-05	0.00149	-5E-05	0.00095	0.0016
1.5E-05	0.00057	0.00108	0.00069	0.0011	0.00172
1.5E-05	0.00065	0.00114	-0.0005	0.00101	0.00089
1.5E-05	-0.0004	0.00076	0.00025	0.00094	0.00183
1.5E-05	-0.0005	0.00089	-0.0001	0.00107	0.00153
1.5E-05	-0.0004	0.00053	0.00057	0.00164	0.00132
1.5E-05	-0.0003	0.0002	-0.0004	0.00097	0.00117
1.5E-05	-0.0011	0.00085	7.3E-05	0.00038	0.00152
1.5E-05	-0.0005	0.00086	0.00025	0.00102	0.00117
1.5E-05	-0.0003	0.0006	-0.0003	0.00055	0.00114
1.5E-05	-0.0002	0.00059	-0.0003	0.00111	0.00163
1.5E-05	-0.0004	0.00076	0.00026	0.00122	0.0022
1.5E-05	-0.0002	0.00075	0.00037	0.00081	0.00102
1.5E-05	-0.0002	0.00145	0.00063	0.00138	0.00187
1.5E-05	-0.0002	0.00067	0.00084	0.00059	0.00133
1.5E-05	1.1E-05	0.00072	0.00057	0.00132	0.00118
1.5E-05	-0.0008	0.00141	0.00064	0.00021	0.0015

Table SI.12: Primary kinetic data for the reaction of ω-heptalactam (**S12**) with CumO$^{\bullet}$ generated by 355 nm LFP in MeCN (**Figure SI.12**).

**t / s**	**ΔA ω-heptalactam (S12)**				
	**0.270M**	**0.70M**	**1.39M**	**1.925M**	**2.695M**
-1E-06	0.00041	-0.0006	-0.0005	0.00038	0.00075
-1E-06	-6E-05	-0.0006	-0.0009	-0.0002	0.00091
-9E-07	-4E-05	-0.0004	-0.0012	-0.0002	0.00111
-9E-07	0.00085	0.00052	-0.0004	-0.0004	0.00126
-9E-07	0.00062	-0.0001	-0.0011	7.3E-06	0.00109
-8E-07	0.00068	-0.0005	-0.0004	-0.0001	0.00118
-8E-07	0.00024	-0.0006	-0.001	0.0006	0.00153
-8E-07	0.00023	-0.0012	-0.0019	-0.0007	0.00138
-7E-07	0.00055	-0.0013	-0.0011	-0.0004	0.00099
-7E-07	0.00036	-0.0004	-0.002	-0.0004	0.00219
-7E-07	-0.0003	-0.0005	-0.0006	0.0002	0.00129
-6E-07	6.8E-05	-0.0003	-0.0009	-0.0003	0.00135
-6E-07	-0.0006	-0.0008	-0.0018	0.0008	0.00133
-6E-07	-5E-05	-0.0011	-0.0007	0.00051	0.0014
-5E-07	-0.0007	-0.0006	-0.0016	0.00048	0.00058
-5E-07	0.00027	-0.0005	-0.0013	0.00014	0.00135
-5E-07	8.7E-05	-0.0009	-0.0011	0.00016	0.00283
-4E-07	0.00014	0.00039	-0.0016	-0.0005	0.00159
-4E-07	-0.001	-0.0009	-0.0017	-0.0007	0.00049
-4E-07	-0.0001	-0.0005	-0.001	0.00017	0.00126
-4E-07	-0.0002	-0.0003	-0.0006	-0.0003	0.00138
-3E-07	-0.0002	-0.0008	-0.0009	-0.0006	0.00183
-3E-07	-0.0002	-0.0001	-0.0003	0.00011	0.00141
-3E-07	7E-05	-0.0008	-0.0011	-0.0002	0.00099
-2E-07	-0.0003	-0.0002	-0.0013	-0.0002	0.00068
-2E-07	0.00033	-5E-05	-0.0014	-1E-05	0.00111
-2E-07	0.00028	-0.0005	-0.0015	0.00012	0.00173
-1E-07	0.00045	0.00073	-0.0019	-0.0006	0.0017
-1E-07	-1E-05	-8E-05	-0.0015	-0.0004	0.00062
-6E-08	-0.0005	-8E-05	-0.0008	0.00082	0.00141
-3E-08	-0.0004	-0.0006	-0.0021	0.00045	0.00104
-4E-10	-0.0034	-0.0042	-0.0049	-0.0029	-0.0023
3.2E-08	-0.0071	-0.0143	-0.0153	-0.0146	-0.017
6.4E-08	0.02508	0.02354	0.0189	0.01873	0.01711
9.6E-08	0.0274	0.02633	0.02412	0.0251	0.02417
1.3E-07	0.02698	0.02604	0.02336	0.02407	0.02352
1.6E-07	0.02693	0.0241	0.02203	0.02189	0.02121
1.9E-07	0.02565	0.02356	0.02058	0.02201	0.02047
2.2E-07	0.02446	0.02328	0.01859	0.01952	0.01927
2.6E-07	0.02405	0.02178	0.01847	0.01889	0.01769
2.9E-07	0.02288	0.02051	0.01715	0.01864	0.01688
3.2E-07	0.02238	0.01953	0.01618	0.01686	0.01523
3.5E-07	0.02159	0.0186	0.01517	0.01525	0.01448
3.8E-07	0.02176	0.01802	0.01493	0.01474	0.01411
4.2E-07	0.02026	0.01772	0.01402	0.0132	0.01296
4.5E-07	0.02002	0.01685	0.01383	0.01225	0.01221
4.8E-07	0.01838	0.01639	0.01163	0.01183	0.01142
5.1E-07	0.01704	0.01527	0.01175	0.011	0.01116
5.4E-07	0.01727	0.0146	0.01042	0.01069	0.01034

5.8E-07	0.01785	0.01349	0.01052	0.00989	0.00997
6.1E-07	0.01677	0.01365	0.01023	0.00965	0.00905
6.4E-07	0.01646	0.01291	0.00988	0.00802	0.00849
6.7E-07	0.01615	0.01219	0.0095	0.00826	0.00794
7E-07	0.01499	0.01223	0.00883	0.00786	0.00793
7.4E-07	0.01601	0.01109	0.00759	0.00709	0.00737
7.7E-07	0.01474	0.01057	0.00783	0.00667	0.00695
8E-07	0.01364	0.01098	0.00754	0.00635	0.00584
8.3E-07	0.01314	0.01009	0.00718	0.00567	0.00579
8.6E-07	0.01309	0.00928	0.00661	0.00555	0.0053
9E-07	0.01271	0.0091	0.00513	0.00473	0.00558
9.3E-07	0.013	0.00869	0.0047	0.0047	0.0048
9.6E-07	0.01169	0.00879	0.00495	0.00446	0.00461
9.9E-07	0.01163	0.0085	0.00481	0.00365	0.00425
1E-06	0.01145	0.00779	0.00448	0.00495	0.00423
1.1E-06	0.01039	0.0076	0.00486	0.00341	0.00403
1.1E-06	0.0115	0.00698	0.00412	0.00353	0.00409
1.1E-06	0.01023	0.00624	0.00389	0.00302	0.00425
1.2E-06	0.00988	0.0066	0.0036	0.00312	0.00429
1.2E-06	0.01	0.00646	0.00309	0.00319	0.00409
1.2E-06	0.00975	0.00613	0.00298	0.00296	0.00407
1.2E-06	0.00922	0.00569	0.00232	0.00322	0.00365
1.3E-06	0.00889	0.00614	0.00286	0.0025	0.0035
1.3E-06	0.00877	0.00533	0.00284	0.00272	0.00283
1.3E-06	0.00868	0.00507	0.00247	0.00152	0.00317
1.4E-06	0.00892	0.0054	0.00242	0.00143	0.00338
1.4E-06	0.0077	0.00549	0.00214	0.00214	0.00261
1.4E-06	0.00838	0.00475	0.00134	0.0017	0.00275
1.5E-06	0.00702	0.00453	0.00143	0.00182	0.00323
1.5E-06	0.00817	0.00479	0.00181	0.00253	0.00346
1.5E-06	0.0072	0.0035	0.00168	0.00256	0.00205
1.6E-06	0.00721	0.00421	0.00077	0.00112	0.00267
1.6E-06	0.0075	0.0044	0.00126	0.00171	0.00303
1.6E-06	0.00774	0.00399	0.00161	0.00136	0.00267
1.7E-06	0.00674	0.00347	0.00127	0.0017	0.00321
1.7E-06	0.00649	0.00371	0.00099	0.00132	0.00314
1.7E-06	0.0056	0.00314	0.00128	0.00115	0.0025
1.8E-06	0.00562	0.00267	0.00065	0.00121	0.0028
1.8E-06	0.00631	0.00287	0.00046	0.00119	0.00266
1.8E-06	0.00592	0.00318	0.00097	0.00107	0.00273
1.9E-06	0.00571	0.002	0.00084	0.00122	0.00178
1.9E-06	0.00488	0.00264	0.0009	0.00109	0.00211
1.9E-06	0.00498	0.00279	0.00068	0.00122	0.00295
2E-06	0.00461	0.00175	0.00043	0.00164	0.00226
2E-06	0.00526	0.00218	0.00048	0.00121	0.00254
2E-06	0.00507	0.00151	0.00082	0.00089	0.00237
2E-06	0.00426	0.0019	0.00026	0.00083	0.00102
2.1E-06	0.00466	0.0011	0.00038	0.00059	0.00255
2.1E-06	0.00423	0.00172	3.2E-05	0.0005	0.00216
2.1E-06	0.00478	0.00153	0.00084	0.00081	0.00215
2.2E-06	0.00553	0.00186	0.00069	0.0005	0.00154
2.2E-06	0.00411	0.00216	8.1E-05	0.00067	0.00231
2.2E-06	0.0049	0.00133	0.00038	0.00089	0.0023
2.3E-06	0.00315	0.00119	-8E-05	0.00041	0.00342

2.3E-06	0.00349	0.00172	0.00047	0.00015	0.00195
2.3E-06	0.00443	0.00111	0.00016	0.00069	0.00191
2.4E-06	0.00415	0.0015	0.00017	0.00103	0.00177
2.4E-06	0.00376	0.0012	-0.0001	0.00067	0.00198
2.4E-06	0.0037	0.0008	0.00033	0.00062	0.00159
2.5E-06	0.00388	0.00066	-0.0002	0.00067	0.00215
2.5E-06	0.00312	0.00033	-0.0003	0.00028	0.00272
2.5E-06	0.00356	0.00103	-0.0011	0.00054	0.00172
2.6E-06	0.00331	0.00071	-0.0002	0.00109	0.00274
2.6E-06	0.00307	0.00169	-9E-05	5.9E-05	0.00242
2.6E-06	0.00224	0.0014	-0.0005	0.00035	0.00209
2.7E-06	0.00306	0.00097	-0.0009	0.00042	0.00245
2.7E-06	0.00302	0.00076	-0.0004	0.00064	0.00201
2.7E-06	0.00349	0.00101	-0.0006	0.00065	0.00266
2.8E-06	0.00285	0.0009	-0.001	0.00061	0.00224
2.8E-06	0.00298	0.00135	-0.0002	0.00085	0.00157
2.8E-06	0.00268	0.00115	-0.0008	-0.0001	0.00133
2.8E-06	0.00188	0.00017	-0.0006	-0.0002	0.00062
2.9E-06	0.00254	0.00102	-7E-05	0.00051	0.00161
2.9E-06	0.00321	0.00069	-0.0003	0.0003	0.00251
2.9E-06	0.00279	0.00059	0.00012	-0.0002	0.00256
3E-06	0.00222	0.00027	-0.0013	0.00047	0.00205
3E-06	0.00207	0.0008	-0.0017	5.9E-05	0.00231
3E-06	0.00249	0.00093	-0.0003	0.00079	0.00243
3.1E-06	0.00138	0.00098	-0.0006	0.0003	0.00142
3.1E-06	0.00141	0.00037	-0.0006	0.00031	0.00264
3.1E-06	0.00251	0.00107	-0.0009	0.00087	0.00232
3.2E-06	0.00243	0.00026	-0.0013	0.0004	0.00221
3.2E-06	0.00179	0.0008	-0.0008	0.00022	0.00177
3.2E-06	0.00168	0.00057	-0.001	0.00098	0.00249
3.3E-06	0.00283	0.00146	-0.0011	0.00029	0.00231
3.3E-06	0.00165	0.0004	-0.0007	0.00057	0.00235
3.3E-06	0.0015	-0.0004	-0.0003	0.00109	0.00275
3.4E-06	0.00158	0.00044	-0.0009	0.00035	0.00204
3.4E-06	0.00199	0.00046	-0.0013	0.00038	0.00128
3.4E-06	0.00132	1.9E-05	-0.0015	0.00064	0.00145
3.5E-06	0.00114	0.00051	-0.0012	0.00019	0.00138
3.5E-06	0.00089	-4E-05	-0.0015	0.00027	0.00262
3.5E-06	0.00148	5.2E-05	-0.0014	0.00022	0.00298
3.6E-06	0.00082	8.9E-06	-0.0005	0.00031	0.00223
3.6E-06	0.00161	0.00027	-0.0015	0.00109	0.00218
3.6E-06	0.00162	0.00019	-0.0009	0.00042	0.00183
3.6E-06	0.00111	0.00042	-0.0011	0.00051	0.00239
3.7E-06	0.00198	0.00011	-0.0012	0.00086	0.00195
3.7E-06	0.00174	0.00034	-0.0014	0.00064	0.00185
3.7E-06	0.001	-0.0007	-0.0011	0.00036	0.0016
3.8E-06	0.00186	0.00091	-0.0009	0.00011	0.00184
3.8E-06	0.00113	-1E-05	-0.0008	0.00058	0.00215
3.8E-06	0.00121	-1E-06	3.9E-05	0.00134	0.00273
3.9E-06	0.00185	0.00046	-0.0008	0.0003	0.00273
3.9E-06	0.00115	-0.0008	-0.0011	0.00083	0.00187
3.9E-06	0.00073	0.00103	-8E-05	0.00074	0.00133
4E-06	0.00189	9.9E-05	-0.0016	-0.0001	0.00182
4E-06	0.00101	-1E-04	-0.0014	1.6E-05	0.0022

4E-06	0.00104	0.00048	-0.001	-0.0001	0.0015
4.1E-06	0.001	7.9E-05	-0.0015	-1E-04	0.00193
4.1E-06	0.00045	7.8E-05	-0.0016	0.00033	0.00186
4.1E-06	0.00096	2E-05	-0.0011	0.00041	0.00223
4.2E-06	0.00124	-0.0002	-0.0013	0.0008	0.00137
4.2E-06	0.00093	0.00051	-0.0007	0.00081	0.00202
4.2E-06	0.00139	0.0008	-0.0008	0.00023	0.00223
4.3E-06	0.00073	-0.0003	-0.0009	0.0009	0.00095
4.3E-06	0.00047	0.00037	-0.001	0.00018	0.0018
4.3E-06	0.00091	0.00041	-0.0009	0.00062	0.00299
4.4E-06	0.001	-0.0009	-0.0021	0.00031	0.00224
4.4E-06	0.00107	-0.0001	-0.0012	-0.0002	0.00219
4.4E-06	0.00111	-0.0005	-0.0006	0.00097	0.00278
4.4E-06	0.00152	-0.0006	-0.0003	0.00075	0.00166
4.5E-06	0.00093	0.00017	-0.0016	0.00065	0.00178
4.5E-06	0.00118	-0.0003	-0.0007	0.00121	0.00182
4.5E-06	0.00061	-0.0002	-0.0011	0.00058	0.00248
4.6E-06	0.00065	-0.0005	-0.0019	0.00014	0.00218
4.6E-06	0.0011	-0.0012	-0.0014	0.00084	0.00211
4.6E-06	0.00083	-0.0008	-0.0013	0.00076	0.00304
4.7E-06	0.00087	0.00029	-0.0012	0.00029	0.00163
4.7E-06	0.00133	1.4E-05	-0.0018	-0.0001	0.00124
4.7E-06	0.0006	-0.0007	-0.001	0.00024	0.00145
4.8E-06	0.00127	0.00064	-0.0014	0.0003	0.00203
4.8E-06	0.00064	-3E-05	-0.0017	0.00042	0.00161
4.8E-06	0.00023	0.00019	-0.0011	0.00026	0.0017
4.9E-06	0.00069	-0.0006	-0.0002	0.00052	0.00147
4.9E-06	0.00066	-0.0004	-0.0015	0.00116	0.00248
4.9E-06	0.0005	0.00031	-0.0008	5.9E-05	0.00229
5E-06	0.0006	5.8E-05	-4E-05	0.00013	0.00216
5E-06	0.0002	-0.0005	-0.0004	0.00043	0.00261
5E-06	0.00043	-0.0009	-0.0008	3.6E-05	0.00203
5.1E-06	0.00034	0.00028	-0.0006	0.00024	0.00206
5.1E-06	0.00062	-0.0003	-0.0009	-0.0002	0.00201
5.1E-06	0.00076	-0.0003	-0.0013	-3E-05	0.00298
5.2E-06	0.00012	0.00049	-0.0009	-2E-05	0.00252
5.2E-06	0.0012	-0.0001	-0.0012	-0.0003	0.00191
5.2E-06	0.00053	-0.0007	-0.0009	-0.0004	0.00234
5.2E-06	0.00058	-0.0003	-0.0017	-5E-05	0.00208
5.3E-06	0.00073	-0.0011	-0.0008	0.00053	0.00271
5.3E-06	0.00079	-0.0009	-0.0009	0.00015	0.00264
5.3E-06	0.00045	-0.0011	-0.0006	-5E-05	0.0022
5.4E-06	0.00051	-0.0011	-0.0006	0.0006	0.00188
5.4E-06	0.00013	-0.0008	-0.0009	-0.0004	0.00339
5.4E-06	0.0004	-0.0002	-0.0015	0.00053	0.00175
5.5E-06	0.00064	-0.0006	-0.0008	0.00013	0.00176
5.5E-06	0.00065	0.00038	-0.0007	0.00018	0.00235
5.5E-06	0.00097	-0.0007	-0.0005	0.00026	0.00244
5.6E-06	0.00053	-0.0003	-0.0013	-1E-05	0.00173
5.6E-06	0.00044	-0.0008	-0.0004	0.00056	0.00282
5.6E-06	0.00061	-0.0004	-0.001	0.00048	0.00222
5.7E-06	0.00013	-0.0002	-0.001	-0.0002	0.00249
5.7E-06	0.00037	0.00025	-0.0006	6.2E-05	0.00203
5.7E-06	-0.0003	-0.0006	-0.0011	-7E-05	0.00244

5.8E-06	0.00072	-0.0005	-0.0012	4.1E-05	0.00286
5.8E-06	0.00056	-0.0003	-0.0011	0.00101	0.00214
5.8E-06	0.00045	-0.0009	-7E-05	0.00063	0.00267
5.9E-06	0.00019	0.00023	-0.0009	0.0006	0.00233
5.9E-06	0.00065	-0.0005	-0.0012	0.00023	0.00113
5.9E-06	0.00055	0.00023	-0.001	0.00061	0.00181
6E-06	0.00064	-0.0005	-0.0005	0.00062	0.00192
6E-06	0.00022	-0.0008	-0.0011	0.00087	0.00266
6E-06	7E-05	-0.0001	-0.0007	0.00055	0.00176
6E-06	0.00038	-0.0002	-0.0013	0.0004	0.00189
6.1E-06	0.00021	-0.0004	-0.0014	0.00073	0.00247
6.1E-06	6.6E-05	-0.0003	-0.0005	0.0011	0.00245
6.1E-06	0.0015	0.00064	-0.0015	0.00059	0.0028
6.2E-06	0.0004	0.00035	-0.001	0.00062	0.00217
6.2E-06	1.1E-05	-0.0003	-0.0011	0.00129	0.00339
6.2E-06	-0.0001	-7E-05	-0.0017	0.00064	0.00211
6.3E-06	0.00114	-0.0002	-0.0013	0.00061	0.00149
6.3E-06	0.00057	-0.0009	-0.0007	-5E-05	0.00138
6.3E-06	0.00099	-0.0003	-0.0015	0.00022	0.00261
6.4E-06	-0.0003	-0.0008	-0.0011	0.00032	0.00257
6.4E-06	0.0005	-4E-06	-0.001	0.00039	0.00235
6.4E-06	-0.0002	-0.0006	-0.001	0.00018	0.00269
6.5E-06	0.00049	0.00014	-0.0015	0.00027	0.002
6.5E-06	0.00021	-0.0004	-0.0002	-0.0002	0.00231
6.5E-06	-6E-06	-0.0006	-0.0006	-1E-05	0.00198
6.6E-06	0.00079	-0.0006	-0.0007	-4E-05	0.00163
6.6E-06	0.0004	-0.0008	-0.0004	0.00093	0.00118
6.6E-06	-3E-05	-0.0004	-0.001	0.00114	0.00254
6.7E-06	0.00048	-0.0003	-0.0005	0.00048	0.00257
6.7E-06	0.00038	-0.001	-0.0007	0.00093	0.00187
6.7E-06	0.00057	-0.0012	-0.001	-2E-05	0.00233
6.8E-06	0.00048	-7E-05	-0.0014	0.00059	0.00191
6.8E-06	0.00081	0.00021	-0.0014	0.00069	0.00191
6.8E-06	-4E-05	-0.0003	-0.0013	0.00014	0.00255
6.8E-06	-0.0003	-0.0008	-0.0008	0.00061	0.00158
6.9E-06	0.00028	-0.0006	-0.0014	6.6E-05	0.00157
6.9E-06	0.00039	-0.001	-0.0008	0.00056	0.00244
6.9E-06	0.00023	-0.0004	-0.0013	-0.0005	0.0021
7E-06	-4E-05	-0.0004	-0.0008	0.00056	0.00237
7E-06	0.00048	-0.0012	-0.0014	0.00083	0.00201
7E-06	-0.0004	-0.0002	-0.0012	0.00063	0.00202
7.1E-06	0.0003	-0.0009	-0.0016	0.00027	0.00238
7.1E-06	3.5E-05	-0.0001	-0.0014	0.00052	0.00263
7.1E-06	0.00058	-0.0002	-0.0009	0.0004	0.00201
7.2E-06	6.2E-05	-0.0007	-0.0008	0.00038	0.00191
7.2E-06	-0.0004	-0.0008	-0.0001	0.00032	0.00189
7.2E-06	0.0004	-0.0008	-0.0002	0.00029	0.00204
7.3E-06	-0.0003	-0.0007	-0.0012	0.00012	0.00259
7.3E-06	0.00024	-0.0009	-0.0003	0.001	0.00257
7.3E-06	-0.0007	-5E-05	-0.0013	0.00144	0.00194
7.4E-06	2.6E-05	-0.0008	-0.0014	0.00076	0.00269
7.4E-06	0.00089	-1E-04	-0.0009	0.00067	0.00212
7.4E-06	-0.0003	-0.0008	-0.0015	0.00015	0.00255
7.5E-06	-0.0007	-0.0003	-0.0012	0.00037	0.00264

7.5E-06	-0.0006	-0.0004	-0.0012	0.00072	0.00235
7.5E-06	0.00032	-0.0008	-0.001	0.00011	0.00207
7.6E-06	-0.0004	0.00059	-0.0008	0.00068	0.00151
7.6E-06	-1E-04	-0.0005	-0.0013	0.00037	0.00234
7.6E-06	-0.0002	-0.0014	-0.0008	-0.0002	0.00265
7.6E-06	8.4E-05	-0.0013	-0.0007	-0.0001	0.00263
7.7E-06	-6E-05	-0.0008	-0.0017	-4E-05	0.00171
7.7E-06	0.00069	-0.0002	-0.0008	0.00046	0.00214
7.7E-06	-0.0003	-0.0006	-0.0011	0.00065	0.00169
7.8E-06	0.00027	-0.0012	-0.001	0.00041	0.00171
7.8E-06	5.6E-07	-0.0004	-0.0017	0.00027	0.00209
7.8E-06	0.00043	-0.0006	-0.0004	0.00118	0.00192
7.9E-06	0.00048	-0.0016	-0.0015	0.00057	0.00247
7.9E-06	-0.0006	-0.001	-0.0015	-0.0002	0.0018
7.9E-06	-0.0003	-0.0006	-0.0003	-0.0001	0.00213
8E-06	0.0004	-0.0006	-0.0009	0.0007	0.00293
8E-06	0.00085	-0.0005	-0.0008	0.0003	0.0012
8E-06	5.6E-06	-0.0009	-0.0013	1.3E-05	0.00263
8.1E-06	0.00057	-0.0007	-0.0004	0.00044	0.00297
8.1E-06	-3E-05	-0.0008	-0.0015	0.00016	0.00252
8.1E-06	0.00012	-0.0009	-0.0022	5.5E-05	0.00166
8.2E-06	0.00014	-0.0008	-0.0021	0.00048	0.00214
8.2E-06	2.8E-05	-0.0009	-0.0013	8.4E-05	0.00124
8.2E-06	0.00018	-0.0011	-0.0007	0.00082	0.00199
8.3E-06	0.00016	-0.0004	-0.0021	3E-05	0.00231
8.3E-06	-0.0002	-0.0008	-0.0013	-0.0004	0.00181
8.3E-06	0.00026	-0.0009	-0.0007	0.00015	0.00228
8.4E-06	-0.0002	-0.0008	-0.0016	0.00037	0.00269
8.4E-06	0.00046	-0.0004	-0.001	-0.0007	0.00233
8.4E-06	-2E-06	-0.0009	-0.0002	8.4E-05	0.00241
8.4E-06	0.00065	-0.0003	-0.0013	0.00051	0.00186
8.5E-06	-0.0003	-0.0009	-0.0014	0.00066	0.00147
8.5E-06	-7E-05	-0.0011	-0.0009	0.00043	0.00249
8.5E-06	1.2E-05	-0.0012	-0.0015	0.00073	0.0031
8.6E-06	9.4E-05	-0.0005	-0.0012	0.00032	0.00317
8.6E-06	1.8E-05	-0.0008	-0.0009	0.00055	0.00259
8.6E-06	-0.0004	-0.0006	-0.0017	-0.0001	0.00255
8.7E-06	-2E-05	-0.0009	-5E-05	0.00078	0.00174
8.7E-06	-0.0011	-0.0007	-0.002	0.00063	0.00234
8.7E-06	-0.0005	-0.001	-0.0015	0.00036	0.0025
8.8E-06	0.00059	-0.0008	-0.0018	0.00065	0.00289
8.8E-06	-0.0002	-0.0011	-0.0005	-0.0001	0.00219
8.8E-06	-0.0004	-0.0008	-0.0018	0.00081	0.00235
8.9E-06	-3E-05	-0.0004	-0.0008	0.00065	0.00108
8.9E-06	-0.0002	-0.0008	-0.0015	7.8E-05	0.00193
8.9E-06	3.2E-05	-0.0007	-0.0009	0.00067	0.0022
9E-06	-3E-05	-0.001	-0.0018	-0.0001	0.0022
9E-06	0.00045	-0.0007	-0.0017	0.00018	0.00192
9E-06	-0.0005	-0.0008	-0.0017	0.00083	0.00124
9.1E-06	0.00025	0.00015	-0.0019	0.00025	0.00191
9.1E-06	0.00021	-0.0005	-0.0017	0.00054	0.00208
9.1E-06	-8E-05	-0.0003	-0.0015	0.00101	0.0023
9.2E-06	7.2E-06	-0.0012	-0.0014	0.00015	0.00202
9.2E-06	0.00056	-0.0003	-0.001	0.00039	0.0029

9.2E-06	0.00084	-0.0014	-0.0019	0.00026	0.00308
9.2E-06	3.3E-05	-0.0007	-0.0015	-2E-05	0.00241
9.3E-06	4.2E-05	-0.0006	-0.0014	0.0011	0.00287
9.3E-06	0.0002	-0.0014	-0.0007	2.1E-05	0.00193
9.3E-06	-0.0006	-0.0006	-0.0013	0.0005	0.00228
9.4E-06	-0.0008	-0.0003	-0.0017	9.7E-05	0.00306
9.4E-06	-0.0004	-0.0002	-0.0013	0.00104	0.00163
9.4E-06	6.4E-05	-0.0001	-0.0011	0.00152	0.0016
9.5E-06	0.00047	-5E-05	-0.0016	0.0006	0.00178
9.5E-06	-0.0005	0.00022	-0.0002	0.00011	0.00175
9.5E-06	-2E-05	-0.001	-0.0008	0.00051	0.00208
9.6E-06	-4E-05	-0.0007	-0.0007	0.00044	0.00152
9.6E-06	-0.0004	-0.0009	-0.0005	0.00046	0.0017
9.6E-06	-0.0005	-0.0005	-0.0016	0.00029	0.0017
9.7E-06	0.00031	-0.0011	-0.0009	-7E-05	0.00229
9.7E-06	-0.0007	-0.0008	-0.0012	0.0001	0.00229
9.7E-06	-9E-05	-0.0009	-0.001	0.00048	0.0024
9.8E-06	0.00017	-0.0003	-0.001	0.00043	0.00224
9.8E-06	0.00044	-0.0011	-0.0016	0.00076	0.00161
9.8E-06	-7E-05	-0.001	-0.0014	0.00062	0.00214
9.9E-06	-0.0008	0.00024	-0.0009	0.00081	0.00234
9.9E-06	-0.0008	-0.0009	-0.0011	0.00074	0.00101
9.9E-06	-0.0002	-0.0006	-0.0013	0.00037	0.00237
1E-05	0.00035	-0.0014	-0.0016	0.00057	0.00208
1E-05	-3E-05	-0.0008	-0.0015	0.0011	0.00214
1E-05	-0.0003	0.00018	-0.0016	0.00061	0.00187
1E-05	-0.0002	-0.001	-0.0003	0.00079	0.00162
1E-05	0.00031	0.00019	-0.0012	0.00051	0.00149
1E-05	0.00047	-0.0001	-0.0004	0.00065	0.00214
1E-05	0.00014	-0.002	-0.0016	4.7E-05	0.00172
1E-05	0.0003	-0.0014	-0.0015	0.00029	0.00172
1E-05	-0.0007	-0.0004	-0.001	0.00088	0.00219
1E-05	-0.0001	-0.0004	-0.001	-0.0003	0.00105
1E-05	0.00021	0.00021	-0.0012	0.001	0.00201
1E-05	2.7E-05	-0.0002	-0.0013	0.0006	0.00231
1E-05	-0.0002	-0.0013	-0.0018	0.00033	0.00178
1E-05	0.00014	-0.0004	-0.0006	0.00064	0.00138
1E-05	-7E-05	-0.0007	-0.0013	0.00051	0.00245
1E-05	0.00053	-0.0013	-0.0007	0.00018	0.00209
1E-05	-0.0002	-7E-05	-0.0017	0.00054	0.00278
1.1E-05	-0.0002	-0.0005	-0.0015	0.00031	0.00249
1.1E-05	-0.0002	-0.0003	-0.0014	0.00043	0.00213
1.1E-05	0.00011	0.00056	-0.001	0.00057	0.00227
1.1E-05	-1E-04	-0.0004	-0.0011	0.0009	0.00232
1.1E-05	0.0002	-0.0018	-0.0005	0.00095	0.00249
1.1E-05	-0.0005	0.0002	-0.0017	0.00059	0.00218
1.1E-05	0.00044	-0.0005	-0.0015	0.00042	0.00189
1.1E-05	0.00042	-0.0015	-0.001	0.00068	0.00232
1.1E-05	-0.0008	-0.0005	-0.0019	0.00058	0.0025
1.1E-05	-0.0009	-0.0004	-0.0017	0.00031	0.0027
1.1E-05	9E-05	-0.0004	-0.0011	-0.0001	0.00304
1.1E-05	0.00071	-0.0007	-0.0015	0.0009	0.00214
1.1E-05	-0.0005	-0.0003	-0.0012	0.00075	0.00231
1.1E-05	0.00032	-0.0005	-0.0013	0.00045	0.00206

1.1E-05	-0.0003	-0.001	-0.0006	0.00075	0.00172
1.1E-05	0.00047	-0.0007	-0.0008	0.00069	0.00242
1.1E-05	8.7E-06	3.2E-05	-0.0009	0.00042	0.00254
1.1E-05	0.00027	-0.0002	-0.0009	8.1E-05	0.0021
1.1E-05	0.00052	-0.0012	-0.0022	0.00101	0.00276
1.1E-05	-0.0004	-0.0004	-0.001	0.00011	0.00192
1.1E-05	-0.0003	0.00048	-0.0014	-9E-05	0.00197
1.1E-05	-0.0003	-0.0009	-0.0016	-0.0001	0.00198
1.1E-05	0.00072	-0.0004	-0.0009	0.00045	0.00227
1.1E-05	-0.0006	-0.0002	-0.0015	0.00097	0.00212
1.1E-05	-7E-05	-0.0008	-0.0018	0.0007	0.00215
1.1E-05	-0.0007	-0.0011	-0.0019	0.00039	0.00253
1.1E-05	-0.0005	-0.0014	-0.0016	0.00014	0.00194
1.1E-05	1.2E-05	-8E-05	-0.001	0.00039	0.00218
1.1E-05	0.0002	-0.0004	-0.0011	0.00118	0.00163
1.1E-05	-4E-06	0.00013	-0.0015	0.00063	0.00209
1.1E-05	0.00028	-0.0008	-0.0016	0.00079	0.00182
1.1E-05	0.00034	-0.0006	-0.0012	0.00026	0.00144
1.2E-05	0.00023	-0.0009	-0.0014	0.00074	0.00236
1.2E-05	2.3E-05	-0.0016	-0.0019	0.00027	0.00154
1.2E-05	-0.0005	-0.0007	-0.0006	0.0005	0.00306
1.2E-05	-0.0002	-0.0008	-0.0015	0.00132	0.00173
1.2E-05	-0.0002	-0.001	-0.0018	0.00084	0.0021
1.2E-05	2E-05	-5E-05	-0.0015	-0.0002	0.00281
1.2E-05	-0.0006	-0.001	-0.0008	-1E-05	0.00316
1.2E-05	2.4E-06	-0.0013	-0.0012	0.00031	0.00249
1.2E-05	-0.0005	4E-05	-0.0012	-0.0003	0.00249
1.2E-05	-0.0004	0.00017	-0.0012	0.00036	0.00228
1.2E-05	-0.0002	0.00051	-0.0006	0.00121	0.00248
1.2E-05	0.00055	-0.0007	-0.0014	0.00091	0.00178
1.2E-05	0.00042	0.00023	-0.0012	0.00026	0.00255
1.2E-05	-0.0004	-0.0006	-0.0003	-0.0001	0.00202
1.2E-05	5.5E-05	-0.0008	-0.001	-0.0007	0.00201
1.2E-05	-0.0005	0.00012	-0.0005	0.00033	0.00175
1.2E-05	-0.0006	-0.0003	-0.0012	0.00066	0.00186
1.2E-05	-0.0003	1E-05	-0.0011	0.00126	0.00238
1.2E-05	-0.0003	-0.0004	-0.0019	-1E-05	0.00238
1.2E-05	-0.0007	-8E-05	-0.0014	0.00053	0.00187
1.2E-05	0.00067	-0.0003	-0.0022	0.00013	0.00229
1.2E-05	2.7E-05	-0.0009	-0.0013	0.0011	0.00248
1.2E-05	-0.0011	-0.0005	-0.0012	0.00038	0.00258
1.2E-05	-9E-05	-0.0002	-0.001	0.00044	0.00201
1.2E-05	-0.0001	-8E-05	-0.0009	0.00057	0.00176
1.2E-05	-0.0007	1.4E-05	-0.0015	0.00127	0.00197
1.2E-05	-3E-05	-1E-05	-0.0007	0.00013	0.00215
1.2E-05	-0.0004	-0.0007	-0.0014	0.00107	0.00245
1.2E-05	-0.0001	-0.0015	-0.0012	0.00094	0.00174
1.2E-05	-0.0004	0.00043	-0.0018	0.00026	0.00136
1.2E-05	-0.0007	3.4E-06	-0.0002	-0.0003	0.00161
1.3E-05	-0.0002	-0.0001	-0.001	0.0006	0.00159
1.3E-05	-0.0001	0.00036	-0.0011	0.00016	0.00236
1.3E-05	6.4E-05	-0.0006	-0.001	-3E-05	0.00205
1.3E-05	-0.0006	-0.0004	-0.001	-2E-06	0.00275
1.3E-05	-1E-04	-0.0008	-0.0008	0.00014	0.00136

1.3E-05	0.00014	0.00021	-0.0023	0.00018	0.00175
1.3E-05	-0.0004	-0.0005	-0.0013	-0.0007	0.00185
1.3E-05	0.00025	-0.0003	-0.001	-0.0001	0.00239
1.3E-05	6.5E-05	4.4E-05	-0.0018	0.00088	0.00171
1.3E-05	-0.0002	-0.0002	-0.0012	0.00026	0.00194
1.3E-05	5.7E-05	-0.0011	-0.001	-0.0002	0.00224
1.3E-05	0.00021	0.0005	-0.0014	0.00077	0.00184
1.3E-05	0.00037	-0.0003	-0.0012	0.00125	0.00251
1.3E-05	0.00045	-0.0006	-0.0012	0.00182	0.00243
1.3E-05	-7E-05	0.00052	-0.0011	0.0003	0.00208
1.3E-05	-0.0001	0.00039	-0.0023	0.00027	0.00194
1.3E-05	0.00044	-0.0005	-0.0014	-0.0004	0.00161
1.3E-05	-0.0005	0.00029	-0.0012	9.4E-05	0.00284
1.3E-05	-2E-05	-4E-05	-0.0008	0.0005	0.0021
1.3E-05	0.00012	-0.0003	-0.0011	-0.0006	0.00247
1.3E-05	-7E-05	-0.0008	-0.0012	0.00026	0.00166
1.3E-05	-1E-05	-0.0002	-0.0012	0.00088	0.00186
1.3E-05	-0.001	-0.0004	-0.0012	-0.0007	0.0018
1.3E-05	0.0004	-0.0009	-0.0009	0.00092	0.00155
1.3E-05	-0.0003	-0.0007	-0.0015	0.00062	0.0017
1.3E-05	0.00031	-8E-05	-0.0013	0.0002	0.00181
1.3E-05	0.00014	-0.001	-0.0015	-0.0001	0.0019
1.3E-05	0.00041	-0.0008	-0.0008	-0.0003	0.00248
1.3E-05	-0.0002	-0.001	-0.001	0.00012	0.00267
1.3E-05	-3E-05	-0.0004	-0.001	0.00105	0.00268
1.3E-05	0.00013	-0.0004	-0.0006	0.00031	0.00208
1.4E-05	-5E-05	-0.0013	-0.001	-0.0003	0.00294
1.4E-05	0.00076	-5E-05	-0.0015	0.00061	0.00239
1.4E-05	-5E-07	-0.0005	-0.001	0.00025	0.00237
1.4E-05	0.00054	0.00018	-0.0015	9.1E-05	0.00147
1.4E-05	0.00026	7.3E-05	-0.0017	0.00043	0.00254
1.4E-05	-0.0007	-0.0006	-0.0007	0.00017	0.0021
1.4E-05	0.00024	-0.0005	-0.0017	0.00101	0.00223
1.4E-05	0.0003	-5E-05	-0.001	0.00074	0.00262
1.4E-05	-0.0001	0.00057	-0.0012	0.00123	0.00191
1.4E-05	0.00019	0.00028	-0.0004	1.9E-05	0.00181
1.4E-05	0.00048	-0.0006	-0.0006	0.00035	0.00227
1.4E-05	-6E-05	-0.0015	-0.0011	0.00084	0.00151
1.4E-05	0.00013	0.00028	-0.0014	0.00017	0.00294
1.4E-05	0.0003	-0.0002	-0.0009	0.00121	0.0011
1.4E-05	0.00048	-0.0005	-0.0011	-6E-05	0.0009
1.4E-05	-0.0001	-0.0005	-0.001	0.00031	0.00221
1.4E-05	-6E-07	-0.0002	-0.0014	0.00031	0.00232
1.4E-05	-0.0003	-0.0009	-0.0014	-0.0003	0.00193
1.4E-05	-0.0002	-0.0014	-0.0014	0.0004	0.00191
1.4E-05	3.4E-05	-0.0008	-0.0011	0.00039	0.00212
1.4E-05	-0.0003	-0.0012	-0.0008	0.00027	0.00164
1.4E-05	-4E-05	-0.0006	-0.0005	0.00093	0.00162
1.4E-05	-0.0001	-0.0006	-0.0005	-1E-04	0.00128
1.4E-05	5.8E-05	-0.0004	-0.0018	0.00015	0.00215
1.4E-05	-0.0004	-0.0007	-0.0006	0.0005	0.00177
1.4E-05	0.00039	-0.001	-0.0014	0.0009	0.00187
1.4E-05	-2E-05	-0.0002	-0.0012	0.00047	0.00178
1.4E-05	0.00127	-0.0007	-0.0013	0.00029	0.00161

1.4E-05	-0.0003	-0.0004	-0.0011	9.9E-05	0.00221
1.4E-05	-0.0003	1.3E-06	-0.0012	-0.0002	0.00223
1.4E-05	0.00049	-0.0003	-0.0011	0.00036	0.00206
1.5E-05	0.00045	-2E-05	-0.0007	-0.0002	0.0021
1.5E-05	-0.0002	-0.0006	-0.001	0.00089	0.00208
1.5E-05	0.00055	-1E-06	-0.0008	-0.0004	0.0021
1.5E-05	-0.0001	-0.0004	-0.0008	3.4E-05	0.00302
1.5E-05	-0.0004	-0.0007	-0.0014	0.00093	0.00225
1.5E-05	-0.0005	-0.0006	-0.0019	0.00074	0.00195
1.5E-05	-0.0002	-0.0011	-0.001	-0.0003	0.00233
1.5E-05	-0.0003	-0.0009	-0.0012	-0.0004	0.002
1.5E-05	0.00045	-0.0013	-0.0016	-9E-05	0.0025
1.5E-05	-0.0003	-0.0001	-0.0012	3.6E-05	0.00226
1.5E-05	0.00046	-0.0008	-0.0013	0.00017	0.00234
1.5E-05	9.1E-05	0.00011	-0.0011	0.00028	0.00216
1.5E-05	0.00096	-0.0009	-0.0014	-2E-05	0.00217
1.5E-05	0.00018	-0.0004	-0.0003	0.00049	0.00198
1.5E-05	0.00011	-0.0003	-0.0015	-0.0004	0.00193
1.5E-05	-6E-05	0.00037	-0.0013	-0.0008	0.00097

Table SI.13: Primary kinetic data for the reaction of 1-methyl-ω-heptalactam (**S13**) with CumO$^{\bullet}$ generated by 355 nm LFP in MeCN (**Figure SI.13**).

t / s	ΔA 1-methyl-ω-heptalactam (S13)				
	0.173M	0.337M	0.674M	0.990M	1.30M
-1E-06	-0.0005	0.00015	0.00018	-5E-05	-0.0002
-1E-06	-0.0007	0.00018	0.00021	0.00036	-0.0004
-9E-07	-0.0006	0.00093	0.00085	-0.0004	-0.0002
-9E-07	-0.0011	0.00078	0.0006	-0.0004	-0.0005
-9E-07	-0.0006	0.00028	6.6E-05	-1E-04	-0.0009
-8E-07	-0.0009	0.00089	0.0002	0.00016	-0.0009
-8E-07	-0.0008	-0.0001	0.00065	0.00011	-0.0003
-8E-07	-0.0008	0.00082	0.00043	-0.0005	-0.0006
-7E-07	-0.0005	0.00078	0.00069	-0.0002	-0.0009
-7E-07	-0.0003	0.00066	0.00077	-0.0003	-0.0008
-7E-07	3E-05	0.00045	0.0005	-0.0002	-0.0006
-6E-07	-0.0009	0.00052	0.00046	0.00022	-0.001
-6E-07	-0.0007	0.00073	1E-05	0.00015	-0.0007
-6E-07	-0.0007	0.00063	0.00024	0.00073	-0.0007
-5E-07	-0.0009	0.0003	-8E-07	0.00041	-0.0005
-5E-07	-0.0004	0.00087	0.00018	-4E-05	-0.0005
-5E-07	-0.0004	0.00079	-0.0001	-0.0002	-0.0002
-4E-07	-0.0011	0.00052	0.00046	-0.0001	-0.0009
-4E-07	-0.0009	0.00035	0.0007	-0.0004	-0.0009
-4E-07	-0.0008	0.00098	0.0004	3.1E-05	-0.0005
-4E-07	-0.0004	0.00129	-6E-05	0.00013	-0.0006
-3E-07	-0.0009	0.00083	0.00046	-8E-05	-0.0006
-3E-07	-0.0004	0.0008	-8E-05	0.00024	-0.0003
-3E-07	-0.0006	0.00109	0.00014	0.00038	-0.0002
-2E-07	-0.0008	0.00078	0.00083	0.00042	-0.0008
-2E-07	-0.0006	0.00089	0.00053	-0.0006	-0.0002
-2E-07	-0.0011	0.00104	-0.0001	0.00021	-0.0004
-1E-07	-0.0011	0.00142	0.00025	3.8E-05	-0.0003
-1E-07	-0.0008	0.00146	0.00056	0.00041	-0.0002
-6E-08	-0.0006	0.00081	0.00069	3.5E-05	-0.0005
-3E-08	-0.0006	0.00036	5.9E-05	-0.0002	-0.0006
-4E-10	-0.0028	-0.0028	-0.0017	-0.0036	-0.0032
3.2E-08	-0.0052	-0.0059	-0.0073	-0.0103	-0.0121
6.4E-08	0.02154	0.0225	0.02083	0.01987	0.01846
9.6E-08	0.02178	0.02292	0.02127	0.02098	0.01971
1.3E-07	0.02142	0.02201	0.02072	0.01966	0.01785
1.6E-07	0.02039	0.02052	0.01939	0.01895	0.01662
1.9E-07	0.01944	0.01862	0.01812	0.0172	0.01534
2.2E-07	0.01813	0.01817	0.01723	0.01627	0.01482
2.6E-07	0.01796	0.01786	0.0167	0.01502	0.01326
2.9E-07	0.01703	0.01797	0.01614	0.01443	0.01292
3.2E-07	0.01719	0.01695	0.01453	0.01352	0.01131
3.5E-07	0.01635	0.01525	0.01451	0.01294	0.01126
3.8E-07	0.01599	0.01508	0.01383	0.0123	0.01061
4.2E-07	0.01577	0.01508	0.01445	0.01181	0.01018
4.5E-07	0.01425	0.0148	0.01285	0.01139	0.0094
4.8E-07	0.01442	0.0138	0.01259	0.01026	0.00891
5.1E-07	0.01415	0.01394	0.01192	0.0105	0.00906
5.4E-07	0.01365	0.01277	0.0115	0.00987	0.00773

5.8E-07	0.01373	0.01271	0.01091	0.00906	0.00777
6.1E-07	0.01211	0.01216	0.01037	0.00872	0.00742
6.4E-07	0.01209	0.01219	0.01031	0.00857	0.007
6.7E-07	0.01132	0.0106	0.01006	0.00868	0.00604
7E-07	0.0117	0.01176	0.00943	0.00678	0.00561
7.4E-07	0.01156	0.01082	0.01004	0.00669	0.00596
7.7E-07	0.01146	0.0105	0.00892	0.00738	0.00507
8E-07	0.01141	0.01056	0.00902	0.00686	0.00497
8.3E-07	0.01046	0.00974	0.00818	0.00688	0.0055
8.6E-07	0.01042	0.0104	0.00832	0.00629	0.00481
9E-07	0.01041	0.00984	0.00832	0.00597	0.00434
9.3E-07	0.01009	0.00923	0.00807	0.00578	0.0043
9.6E-07	0.0099	0.00873	0.00738	0.00534	0.00478
9.9E-07	0.0078	0.00946	0.00726	0.00524	0.004
1E-06	0.00948	0.00793	0.00613	0.00512	0.00395
1.1E-06	0.00834	0.00726	0.00682	0.00518	0.00402
1.1E-06	0.00801	0.00715	0.00673	0.00526	0.00367
1.1E-06	0.00827	0.00819	0.00618	0.00476	0.00394
1.2E-06	0.0081	0.00765	0.0056	0.00491	0.00354
1.2E-06	0.00801	0.00686	0.00635	0.0043	0.00342
1.2E-06	0.00802	0.00717	0.0059	0.00445	0.00352
1.2E-06	0.00781	0.00648	0.006	0.00417	0.00341
1.3E-06	0.00716	0.00636	0.00557	0.004	0.00276
1.3E-06	0.0069	0.00689	0.00534	0.00376	0.00293
1.3E-06	0.0066	0.00642	0.00592	0.00367	0.00305
1.4E-06	0.00712	0.0062	0.0053	0.00387	0.00283
1.4E-06	0.00589	0.0062	0.00556	0.0037	0.0024
1.4E-06	0.006	0.00588	0.00445	0.00287	0.00286
1.5E-06	0.00579	0.00632	0.00483	0.00321	0.00267
1.5E-06	0.00607	0.00545	0.00466	0.00322	0.00275
1.5E-06	0.00638	0.00515	0.0051	0.00366	0.00268
1.6E-06	0.00566	0.00549	0.00448	0.00218	0.00253
1.6E-06	0.00573	0.00487	0.00486	0.00318	0.00232
1.6E-06	0.00555	0.00468	0.00441	0.00322	0.00316
1.7E-06	0.00615	0.00522	0.00471	0.00281	0.00248
1.7E-06	0.00467	0.00458	0.00427	0.00202	0.0022
1.7E-06	0.0048	0.00447	0.00428	0.00233	0.00277
1.8E-06	0.00465	0.00436	0.00387	0.0021	0.00243
1.8E-06	0.00469	0.00539	0.00369	0.00285	0.00153
1.8E-06	0.00473	0.00484	0.00318	0.00229	0.00154
1.9E-06	0.00442	0.0049	0.00358	0.00193	0.00252
1.9E-06	0.00499	0.00527	0.00342	0.0025	0.00219
1.9E-06	0.00477	0.0043	0.00375	0.00183	0.00158
2E-06	0.00439	0.00392	0.00434	0.00256	0.00217
2E-06	0.00438	0.0044	0.004	0.00242	0.00259
2E-06	0.00352	0.0053	0.00382	0.00274	0.00196
2E-06	0.00377	0.00396	0.00384	0.00263	0.00125
2.1E-06	0.0037	0.00388	0.0035	0.00241	0.00165
2.1E-06	0.00345	0.00395	0.00334	0.00202	0.00165
2.1E-06	0.00389	0.00382	0.00334	0.00202	0.00241
2.2E-06	0.0042	0.00371	0.00332	0.00242	0.00205
2.2E-06	0.00414	0.00418	0.00405	0.00156	0.00203
2.2E-06	0.00351	0.00334	0.00313	0.00218	0.00157
2.3E-06	0.00325	0.00281	0.00315	0.00205	0.00194

2.3E-06	0.00373	0.0022	0.00287	0.00177	0.00217
2.3E-06	0.00325	0.00382	0.00362	0.00149	0.00161
2.4E-06	0.00353	0.00385	0.00356	0.00221	0.0023
2.4E-06	0.00214	0.00304	0.00353	0.0025	0.00181
2.4E-06	0.00365	0.00214	0.00304	0.00227	0.00231
2.5E-06	0.00375	0.0029	0.00306	0.00181	0.00233
2.5E-06	0.00252	0.00337	0.00323	0.00178	0.00223
2.5E-06	0.00268	0.00305	0.00283	0.00159	0.00189
2.6E-06	0.00296	0.00315	0.00331	0.0026	0.00214
2.6E-06	0.0032	0.00342	0.00329	0.00188	0.00221
2.6E-06	0.00265	0.00314	0.00288	0.00219	0.00186
2.7E-06	0.00261	0.00264	0.00331	0.00165	0.00151
2.7E-06	0.00298	0.00303	0.00316	0.00241	0.00224
2.7E-06	0.00287	0.00376	0.00315	0.00192	0.00189
2.8E-06	0.00179	0.00337	0.00299	0.00176	0.00204
2.8E-06	0.00287	0.00358	0.00298	0.0017	0.00196
2.8E-06	0.00259	0.00239	0.00261	0.00171	0.00205
2.8E-06	0.00246	0.00247	0.00278	0.0018	0.00226
2.9E-06	0.00208	0.00267	0.00315	0.00186	0.00267
2.9E-06	0.00291	0.0033	0.00315	0.00182	0.00251
2.9E-06	0.00246	0.00326	0.0033	0.00186	0.00228
3E-06	0.00226	0.00276	0.00374	0.00174	0.00199
3E-06	0.00252	0.00309	0.00318	0.00219	0.00159
3E-06	0.00266	0.00322	0.00266	0.00136	0.00166
3.1E-06	0.00265	0.00283	0.00285	0.00178	0.00185
3.1E-06	0.00219	0.00235	0.00274	0.00193	0.00247
3.1E-06	0.00237	0.00262	0.00275	0.00141	0.00242
3.2E-06	0.00241	0.00241	0.00264	0.0022	0.00253
3.2E-06	0.00219	0.00219	0.0027	0.00219	0.00134
3.2E-06	0.0023	0.00299	0.00276	0.00197	0.00144
3.3E-06	0.0011	0.00212	0.0027	0.00208	0.00128
3.3E-06	0.00205	0.00282	0.00288	0.00196	0.00187
3.3E-06	0.00198	0.0029	0.0025	0.00131	0.00142
3.4E-06	0.00158	0.0029	0.00244	0.00078	0.00178
3.4E-06	0.00232	0.00228	0.00264	0.00108	0.00146
3.4E-06	0.00202	0.00252	0.00269	0.00221	0.00196
3.5E-06	0.00163	0.00211	0.00238	0.00109	0.00194
3.5E-06	0.00182	0.0025	0.00222	0.00135	0.00175
3.5E-06	0.00228	0.00225	0.00218	0.00135	0.00171
3.6E-06	0.00248	0.00215	0.00311	0.00172	0.00211
3.6E-06	0.00215	0.00246	0.00284	0.00128	0.00189
3.6E-06	0.00234	0.00219	0.00186	0.00134	0.00158
3.6E-06	0.00166	0.00292	0.00213	0.00151	0.00146
3.7E-06	0.00198	0.00202	0.00177	0.00148	0.00133
3.7E-06	0.00116	0.00209	0.00206	0.00036	0.00182
3.7E-06	0.0021	0.0029	0.00203	0.00112	0.00163
3.8E-06	0.0019	0.00146	0.00214	0.00123	0.00176
3.8E-06	0.00162	0.00219	0.00207	0.00094	0.00189
3.8E-06	0.00127	0.00225	0.00203	0.00089	0.00183
3.9E-06	0.0011	0.00198	0.00176	0.00138	0.00194
3.9E-06	0.00131	0.00214	0.00224	0.00188	0.00184
3.9E-06	0.00134	0.00279	0.00273	0.00109	0.0021
4E-06	0.00155	0.00252	0.00199	0.00116	0.00145
4E-06	0.00159	0.00198	0.00279	0.00137	0.00119

4E-06	0.00175	0.00154	0.00181	0.00122	0.00193
4.1E-06	0.00145	0.00289	0.00166	0.00082	0.00121
4.1E-06	0.00086	0.00234	0.00297	0.00125	0.00131
4.1E-06	0.00184	0.00236	0.00179	0.00104	0.00179
4.2E-06	0.00195	0.00126	0.00277	0.0009	0.0022
4.2E-06	0.00183	0.00155	0.00173	0.00102	0.00112
4.2E-06	0.00097	0.00149	0.00237	0.0013	0.00174
4.3E-06	0.00158	0.00203	0.00226	0.00109	0.00204
4.3E-06	0.00161	0.00119	0.00167	0.00132	0.00141
4.3E-06	0.00058	0.00114	0.00215	0.00123	0.00109
4.4E-06	0.00125	0.00119	0.00244	0.00176	0.00166
4.4E-06	0.0009	0.00106	0.00165	0.00125	0.00152
4.4E-06	0.00058	0.00146	0.00158	0.00135	0.00141
4.4E-06	0.00081	0.00211	0.00197	0.00063	0.00155
4.5E-06	0.00146	0.00244	0.00168	0.00096	0.0014
4.5E-06	0.00141	0.0017	0.00138	0.00074	0.00129
4.5E-06	0.00114	0.0021	0.00207	0.00098	0.00085
4.6E-06	0.0013	0.00208	0.00201	0.00073	0.00136
4.6E-06	0.00141	0.00232	0.00199	0.0011	0.00126
4.6E-06	0.00125	0.00252	0.00238	0.0015	0.00168
4.7E-06	0.00172	0.00152	0.00256	0.00091	0.00131
4.7E-06	0.00099	0.00162	0.00102	0.00108	0.00175
4.7E-06	0.00089	0.00165	0.0018	0.0012	0.00147
4.8E-06	0.00115	0.0021	0.00247	0.00102	0.00204
4.8E-06	0.00117	0.00261	0.00234	0.00093	0.00058
4.8E-06	0.00089	0.00087	0.00283	0.00117	0.00222
4.9E-06	0.00096	0.00121	0.002	0.00092	0.00186
4.9E-06	0.00087	0.00173	0.00129	0.00114	0.00088
4.9E-06	0.00106	0.00191	0.00142	0.00051	0.00137
5E-06	0.00129	0.00141	0.00169	0.00115	0.0011
5E-06	0.00074	0.00184	0.00153	0.00148	0.00164
5E-06	0.00072	0.00197	0.00143	0.00126	0.00121
5.1E-06	0.00119	0.00123	0.00148	0.00104	0.0014
5.1E-06	0.00082	0.00217	0.00184	0.00121	0.00105
5.1E-06	0.00107	0.00181	0.00124	0.00106	0.0012
5.2E-06	0.00113	0.00155	0.00129	0.00111	0.00081
5.2E-06	0.00102	0.00093	0.00172	0.00165	0.0015
5.2E-06	0.00096	0.00189	0.00128	0.00098	0.00116
5.2E-06	0.00088	0.00244	0.0012	0.0002	0.00079
5.3E-06	0.00149	0.00131	0.00152	0.00091	0.00145
5.3E-06	0.00091	0.00199	0.00212	0.00125	0.00175
5.3E-06	0.00092	0.00188	0.00177	0.00095	0.00129
5.4E-06	0.00088	0.00183	0.00153	0.00089	0.00182
5.4E-06	0.00081	0.00201	0.00105	0.00051	0.00107
5.4E-06	0.00069	0.00181	0.00107	0.00043	0.00141
5.5E-06	0.00056	0.00185	0.00142	0.00094	0.00141
5.5E-06	0.00056	0.00057	0.00116	0.00063	0.00181
5.5E-06	0.00088	0.00116	0.00185	0.00092	0.00107
5.6E-06	0.00127	0.0013	0.00145	0.00119	0.00169
5.6E-06	0.00055	0.0007	0.00142	0.00152	0.00135
5.6E-06	0.00125	0.00089	0.00129	0.00067	0.00115
5.7E-06	0.00074	0.00089	0.00113	0.00131	0.00077
5.7E-06	0.00107	0.00112	0.00223	0.00132	0.00118
5.7E-06	0.00122	0.00107	0.00193	0.00119	0.00129

5.8E-06	0.00101	0.00163	0.00105	0.00108	0.00138
5.8E-06	9.9E-05	0.00197	0.00161	0.00125	0.00095
5.8E-06	0.00054	0.00148	0.0009	0.00087	0.00054
5.9E-06	0.00074	0.00153	0.00093	0.00116	0.00112
5.9E-06	0.00084	0.00133	0.00143	0.00084	0.00148
5.9E-06	0.00024	0.0016	0.00116	0.00174	0.00072
6E-06	0.00063	0.00245	0.0012	0.00153	0.00069
6E-06	0.00097	0.00175	0.00094	0.00101	0.00075
6E-06	0.00114	0.00152	0.00182	0.00181	0.00137
6E-06	0.00068	0.00155	0.001	0.00095	0.00101
6.1E-06	0.0004	0.00132	0.00138	0.00093	0.00082
6.1E-06	0.00129	0.00191	0.00159	0.00032	0.00146
6.1E-06	0.00173	0.00161	0.00168	0.00094	0.00125
6.2E-06	0.00103	0.00228	0.00135	0.0011	0.00129
6.2E-06	0.00104	0.00132	0.00148	0.00118	0.001
6.2E-06	0.00053	0.00129	0.00157	0.00075	0.00083
6.3E-06	0.0001	0.00159	0.00164	0.00067	0.00084
6.3E-06	0.00103	0.00117	0.00126	0.00097	0.0015
6.3E-06	0.00074	0.00141	0.00142	0.00064	0.00149
6.4E-06	0.00021	0.00195	0.00145	0.00097	0.00055
6.4E-06	0.00151	0.00151	0.00176	0.00084	0.00086
6.4E-06	0.00138	0.00189	0.00121	0.00131	0.00057
6.5E-06	-4E-05	0.00138	0.00132	0.00124	0.00115
6.5E-06	0.0001	0.00223	0.00177	0.00087	0.00129
6.5E-06	0.00029	0.00157	0.00096	0.00152	0.00073
6.6E-06	0.00073	0.00193	0.00109	0.00074	0.00054
6.6E-06	0.00137	0.00137	0.00079	0.00091	0.0012
6.6E-06	0.00023	0.00204	0.00186	0.00058	0.00066
6.7E-06	0.00063	0.00165	0.00186	0.00065	0.00102
6.7E-06	0.00077	0.00246	0.00115	0.00144	0.00093
6.7E-06	0.0009	0.00116	0.00049	0.00113	0.00068
6.8E-06	0.00083	0.00165	0.00174	0.00128	0.00121
6.8E-06	0.00061	0.00225	0.00162	0.00056	0.00064
6.8E-06	0.00066	0.00239	0.00133	0.00037	0.00074
6.8E-06	0.00069	0.00218	0.00165	0.00093	0.00117
6.9E-06	0.00024	0.00186	0.00142	0.0012	0.00072
6.9E-06	0.00031	0.00129	0.00155	0.00047	0.00132
6.9E-06	0.00068	0.00094	0.00186	0.00079	0.00097
7E-06	0.00101	0.00175	0.0016	0.00127	0.0006
7E-06	0.00104	0.00155	0.001	0.00087	0.00067
7E-06	-2E-06	0.00164	0.00113	0.00106	0.00089
7.1E-06	0.00012	0.00126	0.00116	0.00109	0.00054
7.1E-06	5.1E-05	0.00135	0.00212	0.001	0.00073
7.1E-06	-0.0003	0.00153	0.00154	0.00104	0.00116
7.2E-06	-0.0003	0.00182	0.00037	0.00094	0.00053
7.2E-06	0.00046	0.00148	0.00154	0.00076	0.00138
7.2E-06	-0.0001	0.00163	0.00233	0.00121	0.00078
7.3E-06	0.00071	0.00188	0.00203	0.00157	0.00073
7.3E-06	0.00068	0.00184	0.00141	0.00141	0.00069
7.3E-06	0.00046	0.0018	0.0012	0.00147	0.00109
7.4E-06	0.00093	0.00135	0.0021	0.00088	0.0009
7.4E-06	0.00124	0.0017	0.00134	0.00095	0.00083
7.4E-06	-0.0002	0.00031	0.00132	0.00193	-0.0001
7.5E-06	0.0008	0.00127	0.00195	0.00083	0.00088

7.5E-06	0.00038	0.00182	0.00091	0.00085	0.00083
7.5E-06	0.00038	0.00163	0.00145	0.00098	0.00055
7.6E-06	0.00027	0.00136	0.00178	0.00113	0.00075
7.6E-06	0.00054	0.00163	0.00144	0.00069	0.001
7.6E-06	0.00027	0.00189	0.00144	0.0013	0.00041
7.6E-06	3.1E-05	0.00183	0.00123	0.00079	0.00152
7.7E-06	0.00058	0.00166	0.00138	0.0009	0.0005
7.7E-06	0.00038	0.00169	0.0013	0.00097	0.00077
7.7E-06	0.00031	0.00175	0.00133	0.00131	0.0014
7.8E-06	0.00012	0.00149	0.00131	0.00125	0.00149
7.8E-06	0.00062	0.00118	0.00086	0.0006	0.00065
7.8E-06	0.00016	0.00143	0.00148	0.00093	0.00158
7.9E-06	5E-06	0.00104	0.00137	0.00081	0.00103
7.9E-06	0.00034	0.00131	0.00137	0.00101	0.00092
7.9E-06	0.00063	0.00039	0.00107	0.00051	0.00111
8E-06	0.00057	0.00153	0.00113	0.00179	0.00085
8E-06	0.0005	0.00145	0.00145	0.00158	0.00115
8E-06	-0.0003	0.00093	0.00108	0.00149	0.00036
8.1E-06	0.00054	0.00074	0.00223	0.00133	0.00118
8.1E-06	0.00069	0.00192	0.00186	0.00167	0.00053
8.1E-06	0.00026	0.00131	0.00145	0.00048	0.00195
8.2E-06	0.0002	0.00186	0.00201	0.00081	0.0016
8.2E-06	0.00059	0.00196	0.00147	0.00134	0.00102
8.2E-06	-0.0002	0.00108	0.0021	0.00183	0.00135
8.3E-06	-0.0001	0.00116	0.00163	0.00133	0.00122
8.3E-06	-0.0004	0.00042	0.00182	0.00058	0.00117
8.3E-06	5.2E-05	0.00052	0.00209	0.00117	0.00052
8.4E-06	0.00067	0.00125	0.00175	0.00041	0.00165
8.4E-06	0.00056	0.00167	0.00087	0.00119	0.00065
8.4E-06	0.00091	0.00237	0.00108	0.00093	0.00042
8.4E-06	0.0006	0.00112	0.00208	0.00092	0.00095
8.5E-06	-0.0001	0.00204	0.0016	0.00057	0.00092
8.5E-06	-0.0003	0.00124	0.00207	0.00159	0.0011
8.5E-06	-4E-05	0.0011	0.00185	0.00139	0.00101
8.6E-06	0.00052	0.00201	0.00177	0.001	0.00069
8.6E-06	0.00065	0.00103	0.0023	0.00123	0.0006
8.6E-06	0.00056	0.00156	0.00163	0.00104	0.00086
8.7E-06	0.00074	0.00187	0.00171	0.00036	0.00195
8.7E-06	0.00012	0.00126	0.00176	0.00127	0.00066
8.7E-06	0.00072	0.0012	0.00186	0.00169	0.00107
8.8E-06	-4E-06	0.00156	0.00139	0.0009	0.00106
8.8E-06	0.0003	0.00164	0.00231	0.00116	0.00049
8.8E-06	6.2E-05	0.00148	0.00198	0.00039	0.00148
8.9E-06	0.00022	0.0004	0.00112	0.0016	0.00044
8.9E-06	0.00035	0.00189	0.00179	0.00103	0.0009
8.9E-06	0.00067	0.00188	0.00114	0.00138	0.00073
9E-06	0.00066	0.00188	0.00176	0.00109	0.00034
9E-06	0.00045	0.00195	0.00015	0.00155	0.00062
9E-06	0.00022	0.00133	0.00116	0.00095	0.00071
9.1E-06	-9E-05	0.00177	0.00158	0.00029	0.00081
9.1E-06	-0.0002	0.00038	0.00073	0.00091	0.00122
9.1E-06	0.0006	0.00206	0.00098	0.00134	0.00105
9.2E-06	0.0003	0.00143	0.0018	0.00085	0.00092
9.2E-06	0.00024	0.00072	0.00152	0.00073	0.00073

9.2E-06	0.00085	0.00108	0.00211	0.0015	0.00045
9.2E-06	0.00037	0.00113	0.00201	0.0006	0.00063
9.3E-06	6.1E-05	0.00084	0.00172	0.00012	0.0015
9.3E-06	0.00033	0.00189	0.00123	0.00151	0.00022
9.3E-06	-0.0003	0.00151	0.00134	0.0007	0.0004
9.4E-06	-0.0002	0.00152	0.00199	0.00115	0.00057
9.4E-06	-0.0001	0.00164	0.00189	0.00091	0.00136
9.4E-06	0.00042	0.00098	0.00195	0.00128	0.00032
9.5E-06	0.00029	0.00147	0.00144	0.00123	0.00109
9.5E-06	-0.0007	0.00164	0.00181	0.00039	0.00169
9.5E-06	0.00035	0.00141	0.0019	0.00108	0.00092
9.6E-06	3.4E-05	0.00098	0.00132	0.00098	0.00062
9.6E-06	-0.0003	0.00141	0.00121	0.00149	0.00044
9.6E-06	-0.0003	0.0012	0.00093	0.00058	0.00081
9.7E-06	0.00075	0.0015	0.00113	0.00104	0.00057
9.7E-06	0.00015	0.00153	0.00157	0.0007	0.00066
9.7E-06	0.00083	0.00098	0.00136	0.00111	0.00113
9.8E-06	0.00048	0.00133	0.00098	0.0013	0.00063
9.8E-06	-0.0002	0.00155	0.00103	0.00123	0.00069
9.8E-06	0.00042	0.00206	0.00181	0.00087	0.00091
9.9E-06	-8E-05	0.00113	0.00075	0.00116	0.0014
9.9E-06	-1E-05	0.00253	0.00168	0.00112	0.00126
9.9E-06	0.0002	0.00122	0.00124	0.00165	0.00146
1E-05	-0.0005	0.00159	0.00154	0.00103	0.00065
1E-05	0.00014	0.00127	0.00182	0.00044	0.00017
1E-05	0.00023	0.00089	0.00205	0.00062	0.0011
1E-05	0.00063	0.00145	0.00162	0.00098	0.00095
1E-05	-0.0002	0.00164	0.00181	0.00107	0.00048
1E-05	-0.0001	0.0012	0.00083	0.0005	0.00069
1E-05	-0.0004	0.00159	0.00131	0.00131	0.00094
1E-05	-2E-05	0.00114	0.00124	0.00146	0.00071
1E-05	-4E-05	0.00122	0.00161	0.00113	0.00089
1E-05	6.4E-05	0.00173	0.00183	0.0008	0.0005
1E-05	0.00106	0.00159	0.00215	0.00081	0.00066
1E-05	0.00014	0.00224	0.00094	0.00038	0.00072
1E-05	-9E-06	0.00098	0.00144	0.00122	0.00123
1E-05	9.6E-05	0.00103	0.00151	0.00121	0.00119
1E-05	-0.0006	0.00069	0.00219	0.00104	0.00086
1E-05	0.00059	0.00064	0.00185	0.00069	-4E-05
1E-05	-0.0002	0.00111	0.00215	0.0012	0.00043
1.1E-05	9.7E-05	0.00038	0.00202	0.00071	0.00083
1.1E-05	0.00027	0.00107	0.00135	0.00108	0.00142
1.1E-05	0.00026	0.00098	0.00099	0.0009	0.00039
1.1E-05	9.6E-05	0.00074	0.00206	0.00145	0.00086
1.1E-05	0.00086	0.00182	0.00147	0.00119	0.00052
1.1E-05	0.00104	0.00129	0.00164	0.00079	0.00073
1.1E-05	0.00033	0.00076	0.00133	0.00145	0.00025
1.1E-05	0.00031	0.00048	0.00171	0.00174	0.00087
1.1E-05	0.00023	0.00213	0.00095	0.00166	0.00082
1.1E-05	-8E-05	0.00171	0.00113	0.00135	0.00064
1.1E-05	0.00041	0.00156	0.00158	0.00056	0.00075
1.1E-05	0.00051	0.00156	0.00085	0.00103	0.0013
1.1E-05	-0.0005	0.00185	0.0022	0.00106	0.00079
1.1E-05	0.00082	0.00197	0.00131	0.0004	0.00072

1.1E-05	-0.0003	0.00137	0.00176	0.00117	0.00096
1.1E-05	-5E-06	0.00086	0.00205	0.00081	0.00125
1.1E-05	-0.0002	0.00071	0.00123	0.00098	0.00085
1.1E-05	8.6E-05	0.00074	0.00125	0.00131	0.00111
1.1E-05	0.00062	0.00116	0.00175	0.00074	0.00098
1.1E-05	0.00036	0.0014	0.00094	0.00133	0.00084
1.1E-05	5.4E-05	0.0016	0.00113	0.00157	0.00042
1.1E-05	0.00065	0.00251	0.00168	0.00108	0.00125
1.1E-05	0.00048	0.00113	0.00214	0.00152	0.00078
1.1E-05	2E-05	0.00089	0.00119	0.00099	0.00082
1.1E-05	0.00041	0.00148	0.00179	0.00121	0.00128
1.1E-05	0.00013	0.00117	0.00149	0.00071	0.00113
1.1E-05	0.00058	0.00075	0.00115	0.00095	0.00061
1.1E-05	0.00028	0.00097	0.00158	0.00112	0.00056
1.1E-05	-0.0001	0.00088	0.00133	0.00087	0.00085
1.1E-05	3.1E-05	0.00129	0.00116	0.00145	0.00064
1.1E-05	-3E-05	0.00147	0.00204	0.00118	0.00105
1.1E-05	0.00043	0.00238	0.0021	0.0013	0.00126
1.2E-05	0.00031	0.00159	0.00146	0.00096	0.00083
1.2E-05	-0.0007	0.0016	0.002	0.00134	0.00108
1.2E-05	0.00012	0.00189	0.00161	0.0012	0.00043
1.2E-05	-6E-06	0.0005	0.00212	0.00073	0.00078
1.2E-05	0.00019	0.00059	0.00142	0.00108	0.00158
1.2E-05	-2E-05	0.0011	0.00155	0.00109	0.00104
1.2E-05	0.00035	0.00161	0.00237	0.00107	0.00101
1.2E-05	0.00018	0.00086	0.00145	0.00056	0.00084
1.2E-05	0.00024	0.00174	0.00238	0.00079	0.00133
1.2E-05	-0.0002	0.00085	0.00177	0.00108	0.00012
1.2E-05	0.00012	0.00169	0.00163	0.0016	0.00134
1.2E-05	-0.0004	0.00191	0.00186	0.00138	0.00102
1.2E-05	0.0002	0.00102	0.00183	0.00114	0.00103
1.2E-05	0.00011	0.00132	0.00168	0.00121	0.00093
1.2E-05	-0.0005	0.00059	0.00087	0.00142	0.00082
1.2E-05	0.00044	0.00116	0.00173	0.00125	0.00091
1.2E-05	-7E-05	0.00106	0.00109	0.00197	0.00098
1.2E-05	7.5E-06	0.00079	0.00146	0.00176	0.00088
1.2E-05	7.7E-05	0.00063	0.00208	0.0017	0.00056
1.2E-05	4.9E-06	0.00053	0.00159	0.00092	0.00072
1.2E-05	0.0001	0.00068	0.00155	0.00103	0.00033
1.2E-05	0.0005	0.00077	0.00113	0.0011	0.0006
1.2E-05	0.00036	0.0007	0.00108	0.00085	0.00089
1.2E-05	0.00089	0.00092	0.00194	0.00135	0.00044
1.2E-05	0.00036	0.00096	0.0016	0.0011	0.00065
1.2E-05	4.3E-05	0.0018	0.00224	0.00158	0.00061
1.2E-05	0.00035	0.00158	0.00144	0.00125	0.00061
1.2E-05	0.00047	0.00182	0.00187	0.00027	0.00054
1.2E-05	0.00036	0.00163	0.0012	0.00107	0.00144
1.2E-05	2E-05	0.002	0.00187	0.00156	0.00077
1.2E-05	0.00038	0.00127	0.00121	0.00075	0.00101
1.3E-05	0.00053	0.0021	0.00141	0.00169	0.00042
1.3E-05	0.00105	0.00172	0.00187	0.00147	0.00103
1.3E-05	0.0009	0.00114	0.00149	0.00111	0.00111
1.3E-05	0.00028	0.00169	0.00229	0.00099	0.00105
1.3E-05	0.00067	0.00142	0.00188	0.00049	0.00075

1.3E-05	0.00061	0.00084	0.00172	0.00043	0.0013
1.3E-05	0.00022	0.00155	0.00181	0.0008	0.00066
1.3E-05	0.00015	0.00166	0.00149	0.00041	0.00062
1.3E-05	0.00052	0.00104	0.00215	0.00086	0.00054
1.3E-05	0.0005	0.00239	0.00199	0.00132	0.00077
1.3E-05	0.00053	0.00153	0.00154	0.00122	0.00075
1.3E-05	0.00057	0.00034	0.0019	0.00108	0.001
1.3E-05	-1E-05	0.00174	0.00137	0.00119	0.00076
1.3E-05	0.00014	0.0011	0.0016	0.00022	0.00077
1.3E-05	0.00021	0.00135	0.00147	0.00125	0.00054
1.3E-05	-0.0001	0.00182	0.00162	0.00125	0.00047
1.3E-05	0.00034	0.00178	0.00129	0.0006	0.00016
1.3E-05	0.00016	0.0017	0.00119	0.00064	-0.0003
1.3E-05	0.00083	0.00126	0.00145	0.00066	0.00077
1.3E-05	0.00056	0.0017	0.00078	0.00061	7.8E-05
1.3E-05	-0.0002	0.00074	0.00181	0.00159	-0.0001
1.3E-05	0.00054	0.00045	0.00148	0.001	0.00015
1.3E-05	0.00038	0.00136	0.00117	-4E-06	0.00037
1.3E-05	0.00013	0.00219	0.00142	0.00033	0.00028
1.3E-05	-2E-05	0.0011	0.0013	0.00103	0.0011
1.3E-05	-3E-05	0.00176	0.00074	0.00125	0.00102
1.3E-05	0.00024	0.00042	0.00172	0.00075	0.00017
1.3E-05	-0.0004	0.00215	0.00067	0.00042	0.00034
1.3E-05	-2E-05	0.00091	0.00206	0.00081	0.00056
1.3E-05	0.00042	0.00071	0.00126	0.00113	0.00106
1.3E-05	0.00014	0.0012	0.00179	0.00112	0.0007
1.4E-05	-0.0006	0.0011	0.00101	0.00101	0.0007
1.4E-05	-1E-05	0.00171	0.00227	0.00081	0.0011
1.4E-05	-8E-05	0.00111	0.00092	0.00069	0.00065
1.4E-05	-7E-05	0.00152	0.00105	0.00115	0.00057
1.4E-05	-0.0004	0.00185	0.00053	0.00145	0.00141
1.4E-05	0.00036	0.00018	0.00121	0.0009	0.00104
1.4E-05	-5E-05	0.00205	0.00205	0.00089	0.00078
1.4E-05	0.00039	0.00235	0.0022	0.00045	0.00063
1.4E-05	-0.0001	0.00072	0.00177	0.00049	0.00059
1.4E-05	0.00029	0.00062	0.0019	0.0008	0.00048
1.4E-05	0.00039	0.00014	0.00117	0.00087	0.00022
1.4E-05	0.00114	0.00179	0.00092	0.00112	0.0008
1.4E-05	-0.0002	0.00083	0.00084	0.00098	0.00073
1.4E-05	-0.001	0.00186	0.00182	0.00092	0.00019
1.4E-05	0.00063	0.00095	0.00096	0.00013	0.0006
1.4E-05	0.00028	0.00086	0.00125	0.00066	0.00112
1.4E-05	-4E-05	0.00187	0.00162	0.00155	0.00042
1.4E-05	0.00075	0.00121	0.00094	0.0012	0.00142
1.4E-05	-0.0002	0.00166	0.00152	0.00089	0.00023
1.4E-05	0.00012	0.00121	0.00197	0.0008	0.00108
1.4E-05	0.00047	0.00143	0.00116	0.00086	-4E-05
1.4E-05	0.00023	0.00133	0.00087	0.00089	0.0006
1.4E-05	0.0006	0.00091	0.00126	0.00083	0.00043
1.4E-05	-0.0004	0.00098	0.0019	0.00092	0.00053
1.4E-05	0.00042	0.00132	0.0005	0.00094	0.00087
1.4E-05	7.9E-05	0.00103	0.00171	0.00137	0.00024
1.4E-05	0.00019	0.00104	0.00137	0.00086	0.00046
1.4E-05	-1E-04	0.00129	0.00121	0.00106	0.00057

1.4E-05	0.00038	0.00055	0.00149	0.00103	0.00088
1.4E-05	0.00017	0.0015	0.00146	0.00069	0.00107
1.4E-05	3E-07	0.00074	0.00148	0.00098	0.00077
1.5E-05	0.00045	0.00217	0.00183	0.00092	0.00087
1.5E-05	0.00046	0.00185	0.00102	0.00121	0.00092
1.5E-05	0.00029	0.00091	0.00125	0.00089	0.00121
1.5E-05	0.00067	0.00104	0.00163	0.00095	0.00085
1.5E-05	0.00028	0.0009	0.00132	0.00068	0.00123
1.5E-05	1.7E-05	0.00048	0.00169	0.00083	0.00127
1.5E-05	-0.0006	0.00154	0.00158	0.00103	0.00135
1.5E-05	-9E-05	0.00176	0.0018	0.00129	9.2E-05
1.5E-05	0.00056	0.00131	0.002	0.00123	0.00017
1.5E-05	0.00044	0.0013	0.00126	0.00158	0.00089
1.5E-05	0.00031	0.00063	0.00183	0.00067	0.00103
1.5E-05	0.00138	0.00155	0.00145	0.00052	0.00136
1.5E-05	0.0003	0.00149	0.00128	0.00097	0.00056
1.5E-05	0.00049	0.00133	0.00202	0.0011	0.00076
1.5E-05	0.00013	0.00148	0.001	0.00084	0.00027
1.5E-05	0.00021	0.00148	0.00158	0.0014	-0.0002

Table SI.14A: Primary kinetic data for the reaction of 2-imidazolidinone (**S14**) with CumO• generated by 355 nm LFP in MeCN (**Figure SI.14A**).

t / s	ΔA 2-imidazolidinone (S14)				
	0.0196M	0.049M	0.098M	0.145M	0.196M
-1E-06	-4E-05	-0.0003	0.00023	0.00063	0.00028
-1E-06	0.00045	-0.0019	8.5E-05	0.00162	0.00038
-9E-07	0.00022	-0.0018	-0.0009	7.7E-05	0.00012
-9E-07	7.7E-05	-0.0014	-0.0003	0.00076	7.7E-05
-9E-07	0.0006	-0.0008	0.00014	0.0003	0.00098
-8E-07	0.00025	-0.0003	-0.0006	0.00045	-0.0001
-8E-07	0.00022	-0.0018	-0.0003	0.00077	-0.0003
-8E-07	0.00093	-0.0016	-0.0005	0.0021	-0.0005
-7E-07	0.00014	-0.0014	-0.0004	0.00151	0.00093
-7E-07	-2E-05	-0.0012	-0.0009	0.00136	0.00034
-7E-07	0.00025	-0.0018	0.00067	0.00145	0.00083
-6E-07	-0.0001	-0.0013	0.00019	0.00112	0.00118
-6E-07	-8E-05	-0.0016	-0.0007	0.00106	0.00082
-6E-07	0.00015	-0.0009	-0.0006	0.00211	0.00056
-5E-07	5.5E-05	-0.001	-3E-05	0.0012	0.00077
-5E-07	0.00049	-0.0008	-4E-05	0.00088	0.00067
-5E-07	-0.0007	-0.001	-0.0008	0.00044	-3E-06
-4E-07	0.00045	-0.0019	-0.0003	0.00065	0.00077
-4E-07	0.00079	-0.0016	-0.0004	0.00107	0.00077
-4E-07	0.0002	-0.0011	-0.0009	0.00161	0.0003
-4E-07	-0.0004	-0.0015	3.5E-05	0.00131	0.00121
-3E-07	0.00048	-0.001	-0.0005	0.00032	0.00072
-3E-07	-0.0003	-0.0004	-0.0003	0.00092	0.00062
-3E-07	-0.0009	-0.0013	6.3E-05	0.00136	-0.0003
-2E-07	-5E-05	-0.0009	-0.0001	-5E-05	0.00116
-2E-07	-0.0005	-0.0009	6.3E-05	0.00049	0.00113
-2E-07	-0.0002	-0.0003	-0.0007	0.00076	0.00061
-1E-07	-0.0005	-0.0015	6.7E-05	0.00133	0.00011
-1E-07	-0.0002	-0.0014	-0.0004	0.00092	0.00011
-6E-08	0.0003	-0.001	8.7E-05	0.0006	-0.0004
-3E-08	0.00059	-0.0009	-0.0005	0.00053	0.00062
-3E-10	-0.0027	-0.0035	-0.0036	-0.0035	-0.0025
3.2E-08	-0.0016	-0.0023	-0.0053	-0.009	-0.0058
6.4E-08	0.02587	0.02491	0.02273	0.02473	0.02192
9.6E-08	0.02612	0.02657	0.02454	0.02485	0.02231
1.3E-07	0.02499	0.02481	0.02308	0.02313	0.02055
1.6E-07	0.02444	0.0244	0.02166	0.02113	0.0194
1.9E-07	0.02391	0.02315	0.02088	0.02054	0.01766
2.2E-07	0.02333	0.02272	0.0203	0.01973	0.01755
2.6E-07	0.02313	0.02227	0.01827	0.01755	0.01609
2.9E-07	0.02198	0.02129	0.0167	0.01731	0.01401
3.2E-07	0.02101	0.02054	0.0163	0.01605	0.01309
3.5E-07	0.0198	0.01923	0.01606	0.01515	0.01198
3.8E-07	0.01951	0.01808	0.01427	0.01354	0.011
4.2E-07	0.01982	0.01851	0.01419	0.01348	0.01061
4.5E-07	0.01839	0.01759	0.01404	0.01287	0.01048
4.8E-07	0.01832	0.01673	0.01245	0.01152	0.00941
5.1E-07	0.01806	0.01562	0.01231	0.01132	0.00852
5.4E-07	0.01814	0.01564	0.012	0.01044	0.00843

5.8E-07	0.01686	0.01494	0.01105	0.00931	0.00749
6.1E-07	0.01605	0.01406	0.01042	0.00914	0.00818
6.4E-07	0.01595	0.01413	0.01002	0.00882	0.00708
6.7E-07	0.01562	0.01256	0.00964	0.00859	0.00618
7E-07	0.01493	0.01312	0.00895	0.00843	0.00514
7.4E-07	0.01427	0.0121	0.00891	0.00805	0.00495
7.7E-07	0.01423	0.01141	0.00784	0.00715	0.00456
8E-07	0.01369	0.01099	0.00794	0.00675	0.00514
8.3E-07	0.01316	0.01059	0.00726	0.00678	0.00482
8.6E-07	0.01315	0.01024	0.00665	0.00639	0.00335
9E-07	0.01204	0.00993	0.00671	0.00552	0.00327
9.3E-07	0.01192	0.00923	0.00622	0.00586	0.00346
9.6E-07	0.01157	0.00887	0.00555	0.00504	0.00345
9.9E-07	0.01125	0.00843	0.00578	0.00539	0.00291
1E-06	0.01035	0.00906	0.00479	0.00573	0.00279
1.1E-06	0.01125	0.0083	0.00535	0.00512	0.00255
1.1E-06	0.01046	0.00821	0.00555	0.0043	0.0025
1.1E-06	0.00991	0.00734	0.00488	0.00462	0.00147
1.2E-06	0.0095	0.00723	0.00452	0.00441	0.00158
1.2E-06	0.00927	0.00778	0.00411	0.00397	0.0018
1.2E-06	0.00967	0.00692	0.00383	0.00512	0.00214
1.2E-06	0.00924	0.00655	0.00436	0.00466	0.00166
1.3E-06	0.00833	0.00642	0.0031	0.004	0.00197
1.3E-06	0.0082	0.00596	0.00433	0.00371	0.0022
1.3E-06	0.00783	0.0053	0.00405	0.00307	0.00163
1.4E-06	0.00845	0.00553	0.00274	0.00284	0.00155
1.4E-06	0.00793	0.00491	0.00311	0.00267	0.00169
1.4E-06	0.00753	0.0041	0.00283	0.00351	0.00087
1.5E-06	0.008	0.00479	0.00333	0.0037	0.00183
1.5E-06	0.0067	0.00442	0.00301	0.00309	0.00125
1.5E-06	0.00796	0.00455	0.00286	0.00347	0.00162
1.6E-06	0.00698	0.00477	0.00221	0.00304	0.00063
1.6E-06	0.00757	0.00422	0.00313	0.00258	0.00115
1.6E-06	0.00644	0.0037	0.00173	0.0034	0.00109
1.7E-06	0.00703	0.00431	0.00237	0.00355	0.00147
1.7E-06	0.00624	0.00426	0.00198	0.00212	0.00092
1.7E-06	0.00561	0.00423	0.00188	0.00225	0.00097
1.8E-06	0.00598	0.00357	0.00286	0.00211	0.00096
1.8E-06	0.00645	0.00398	0.00179	0.00233	0.00171
1.8E-06	0.00621	0.00283	0.00237	0.00212	0.00066
1.9E-06	0.00499	0.00294	0.00158	0.00252	0.00164
1.9E-06	0.00542	0.00277	0.00107	0.00204	0.00118
1.9E-06	0.00537	0.00257	0.00217	0.00292	0.00131
2E-06	0.00491	0.00272	0.00133	0.00165	0.00057
2E-06	0.00493	0.00297	0.00145	0.00196	0.00152
2E-06	0.00488	0.00289	0.00073	0.00182	0.0008
2E-06	0.00492	0.00241	0.00146	0.00215	0.00106
2.1E-06	0.00467	0.00271	0.0021	0.00254	0.00123
2.1E-06	0.004	0.00123	0.00077	0.00276	0.0006
2.1E-06	0.0044	0.00236	0.00056	0.00141	0.00126
2.2E-06	0.00385	0.00212	0.00091	0.00204	0.00087
2.2E-06	0.00402	0.00197	0.0006	0.00135	0.00085
2.2E-06	0.00467	0.00125	0.00062	0.00181	0.00032
2.3E-06	0.0048	0.00163	0.00072	0.00141	0.00115

2.3E-06	0.00461	0.00211	0.0008	0.00219	0.00145
2.3E-06	0.00398	0.00179	0.001	0.00168	0.00109
2.4E-06	0.00374	0.00135	0.00061	0.00241	0.0016
2.4E-06	0.00341	0.001	8.2E-05	0.00228	0.00094
2.4E-06	0.00362	0.00065	0.00025	0.00164	0.00111
2.5E-06	0.00351	0.00106	0.00071	0.0015	0.00073
2.5E-06	0.00388	0.00147	0.00092	0.00249	0.00053
2.5E-06	0.00379	0.00126	0.00072	0.0013	0.001
2.6E-06	0.00321	0.00155	0.0007	0.00153	0.00043
2.6E-06	0.00329	0.00093	0.00085	0.00155	0.00051
2.6E-06	0.00305	0.00095	0.00112	0.00112	-0.0003
2.7E-06	0.00307	0.00123	0.00062	0.0017	0.00077
2.7E-06	0.00392	-0.0002	-0.0001	0.00164	0.00046
2.7E-06	0.00346	-2E-06	0.00117	0.00172	0.00081
2.8E-06	0.00275	0.00082	0.00061	0.00137	0.00091
2.8E-06	0.00297	0.00026	0.00056	0.0022	0.00198
2.8E-06	0.00403	-3E-05	-0.0001	0.00118	0.00122
2.8E-06	0.00263	0.00019	0.00025	0.00118	0.00104
2.9E-06	0.00213	-1E-05	0.00016	0.00101	0.00064
2.9E-06	0.00193	0.00036	0.00103	0.00159	0.00142
2.9E-06	0.00337	0.00036	0.00112	0.00079	0.00087
3E-06	0.00207	0.00014	0.00024	0.00143	0.00085
3E-06	0.00286	0.00078	0.00041	0.00198	0.0012
3E-06	0.00216	0.0003	2.6E-05	0.00191	0.0005
3.1E-06	0.00234	4.6E-05	0.00127	0.00054	0.00056
3.1E-06	0.00253	-8E-05	0.00088	0.00093	0.00066
3.1E-06	0.0016	2.4E-05	-1E-06	0.00113	0.00113
3.2E-06	0.0019	0.00043	-0.0002	0.0014	0.00048
3.2E-06	0.00243	0.00024	0.00013	0.00126	0.00066
3.2E-06	0.00145	0.00078	0.00087	0.00123	0.0011
3.3E-06	0.00179	-9E-05	0.00058	0.00142	0.00082
3.3E-06	0.00096	0.00031	0.00025	0.00183	0.00041
3.3E-06	0.00206	0.00053	0.00041	0.00168	-0.0001
3.4E-06	0.00239	0.00027	0.00068	0.00041	0.00046
3.4E-06	0.00146	-0.0006	2.8E-05	0.00182	0.00134
3.4E-06	0.0022	0.00013	0.00045	0.00119	0.00045
3.5E-06	0.0017	0.00025	0.00066	0.00027	0.00051
3.5E-06	0.00224	-0.0001	5E-05	0.00083	0.00077
3.5E-06	0.00135	6.3E-05	0.00036	0.00225	0.00122
3.6E-06	0.00161	0.00035	0.00059	0.00146	0.00021
3.6E-06	0.00141	-0.0002	0.00052	0.00136	0.00128
3.6E-06	0.00222	4.3E-05	-0.0003	0.00213	0.00043
3.6E-06	0.00236	0.00039	-1E-04	0.00172	0.00154
3.7E-06	0.00166	-0.0006	-0.0002	0.00151	0.00063
3.7E-06	0.00148	-0.0008	-0.0005	0.00093	0.00031
3.7E-06	0.00082	-2E-05	-0.0002	0.00112	0.00043
3.8E-06	0.00118	-0.0008	0.00067	0.00178	0.00051
3.8E-06	0.00146	-0.0008	5.3E-05	0.00191	0.00073
3.8E-06	0.0015	8.4E-05	-0.0001	0.00203	0.00151
3.9E-06	0.00168	-0.0007	-0.0004	0.00183	0.00049
3.9E-06	0.00106	-0.0002	0.00048	0.00165	0.00091
3.9E-06	0.00097	-0.0003	0.00048	0.00159	0.00104
4E-06	0.00127	-0.0012	0.00058	0.00161	0.00171
4E-06	0.00139	0.0004	0.00011	0.00105	0.00068

4E-06	0.00129	-0.0002	0.00066	0.00186	0.00037
4.1E-06	0.00176	-0.0003	0.00054	0.00097	0.0013
4.1E-06	0.00106	-0.0002	0.0002	0.00194	0.00072
4.1E-06	0.00093	0.00022	0.00086	0.0011	0.00078
4.2E-06	0.00096	1.3E-05	-0.0002	0.00138	0.00033
4.2E-06	3.8E-05	-0.0004	0.00031	0.00203	0.00041
4.2E-06	0.00132	-0.0003	-0.0002	0.00166	0.00127
4.3E-06	0.00154	0.00042	-0.0003	0.00203	0.00102
4.3E-06	0.00133	-0.0005	0.00046	0.00181	0.00094
4.3E-06	0.00082	-0.0003	-0.0001	0.00226	0.00038
4.4E-06	0.00102	-0.0014	4.3E-05	0.00059	-5E-05
4.4E-06	0.00123	-0.0008	0.00013	0.00113	0.00098
4.4E-06	0.00099	-0.0015	0.00102	0.00103	2E-05
4.4E-06	0.00115	-0.0011	0.00051	0.00152	0.00057
4.5E-06	0.00135	-8E-05	-0.0002	0.00144	0.00025
4.5E-06	0.00105	0.00048	0.00013	0.0019	0.00068
4.5E-06	0.00121	-0.0004	0.0005	0.00127	0.00107
4.6E-06	0.00078	-0.0003	0.00048	0.00108	0.00065
4.6E-06	0.00166	0.00022	0.00019	0.00117	0.00158
4.6E-06	0.00074	0.00021	2.3E-05	0.00114	0.00084
4.7E-06	0.00084	1.1E-05	-0.0004	0.00172	0.00075
4.7E-06	0.00079	-0.0012	0.0003	0.00095	0.00042
4.7E-06	0.00127	-0.0005	3.6E-05	0.00121	0.00126
4.8E-06	0.00092	-0.0005	0.00032	0.00203	0.00048
4.8E-06	0.00136	-0.0004	8.9E-05	0.00104	0.00049
4.8E-06	0.00112	-0.0006	0.00043	0.00117	0.00114
4.9E-06	-9E-05	-0.0006	1.5E-05	0.00199	0.0013
4.9E-06	0.00128	-0.0004	0.00027	0.00168	0.00026
4.9E-06	0.00072	5.1E-05	1.7E-05	0.00119	0.00065
5E-06	0.00189	-0.0004	0.00017	0.00145	0.00071
5E-06	0.00167	-0.0003	0.00051	0.00133	0.001
5E-06	0.00046	-0.0005	-0.0004	0.00143	0.00122
5.1E-06	0.00039	-0.0003	-8E-05	0.00082	0.00022
5.1E-06	0.00053	-0.0007	0.00082	0.00055	0.00062
5.1E-06	0.00145	-0.0002	0.00021	0.00127	0.00057
5.2E-06	0.00073	0.00067	0.00095	0.00073	0.00027
5.2E-06	0.00048	-0.0008	-3E-05	0.00191	0.00096
5.2E-06	0.00085	-0.0006	4.8E-05	0.00143	0.0009
5.2E-06	0.00136	-0.001	-0.0003	0.00121	0.00116
5.3E-06	0.00063	-0.0003	0.00011	0.00131	0.00065
5.3E-06	0.00043	-0.0003	1.2E-05	0.00086	0.00083
5.3E-06	0.00055	-0.001	-0.0003	0.00105	0.00018
5.4E-06	0.00114	-0.0012	0.00024	0.00049	0.00028
5.4E-06	0.00093	-0.0011	0.00047	0.00068	0.00091
5.4E-06	0.00062	-0.0004	0.00034	0.00273	0.00117
5.5E-06	0.00112	-0.0005	-0.0003	0.00181	0.00074
5.5E-06	0.0005	-0.0012	0.001	0.00097	0.00041
5.5E-06	0.00085	-0.0014	0.00039	0.00158	0.0011
5.6E-06	0.00067	-0.001	0.00016	0.00183	0.00036
5.6E-06	0.00029	-0.0007	7E-05	0.00207	0.00035
5.6E-06	0.00023	-0.0007	-0.0004	0.00212	0.00064
5.7E-06	0.00028	0.0006	0.00037	0.00223	0.00035
5.7E-06	0.00084	-0.0006	0.00036	0.00113	0.00024
5.7E-06	0.00044	-0.0004	0.0005	0.00119	0.00057

5.8E-06	0.00087	-0.0009	0.0001	0.00228	0.00048
5.8E-06	0.00028	1.7E-05	-2E-05	0.00177	0.00041
5.8E-06	0.001	-0.0005	0.00051	0.00152	0.00055
5.9E-06	0.00052	-0.0008	0.0006	0.00142	0.00094
5.9E-06	0.00061	-0.0008	0.00032	0.00122	0.00093
5.9E-06	0.00062	-0.0005	-0.0005	0.0016	0.00112
6E-06	0.00096	-0.0018	0.00033	0.00184	0.00129
6E-06	0.00111	-0.0007	0.00012	0.00143	0.00073
6E-06	-0.0001	-0.0008	0.00058	0.00097	0.00075
6E-06	0.00076	-0.0007	0.00032	0.00082	0.0005
6.1E-06	0.00084	-0.001	-0.0006	0.00071	5.7E-05
6.1E-06	0.00059	-0.0004	0.00016	0.00237	0.00033
6.1E-06	0.00048	-0.0008	7.6E-05	0.00143	0.00082
6.2E-06	0.00107	-0.0012	0.00039	0.00108	0.00085
6.2E-06	0.00079	-0.0013	-0.0004	0.00191	0.00058
6.2E-06	0.00124	-0.0001	-0.0001	0.00139	0.00053
6.3E-06	0.00119	-0.001	-9E-06	0.00094	0.00118
6.3E-06	0.00077	-0.0007	0.00057	0.0015	0.00069
6.3E-06	0.00127	-1E-05	0.00035	0.00058	0.00145
6.4E-06	-0.0003	8.9E-05	0.00023	0.00139	0.00126
6.4E-06	0.00135	-0.0006	-0.0005	0.00148	8.4E-05
6.4E-06	0.00053	-0.0008	0.00039	0.00168	0.00088
6.5E-06	0.00075	-0.0009	0.00037	0.00096	0.00109
6.5E-06	0.00082	-0.0004	-0.0003	0.00071	0.00113
6.5E-06	0.00074	-0.0006	0.00025	0.00114	0.0009
6.6E-06	0.00078	-9E-05	4.4E-05	0.00103	0.00116
6.6E-06	0.00059	-0.0006	5.2E-05	0.00141	0.00095
6.6E-06	0.00052	-0.0009	-8E-05	0.00162	0.00018
6.7E-06	0.00117	-0.0003	0.00013	0.00095	0.0006
6.7E-06	0.00071	-0.0008	0.00038	0.00108	0.00086
6.7E-06	0.0008	-0.0013	-0.0002	0.0013	0.00132
6.8E-06	0.00092	-0.001	0.00027	0.00148	0.00108
6.8E-06	0.00042	-0.0003	0.00039	0.00156	0.00035
6.8E-06	0.00063	-0.0014	0.00019	0.00191	0.00094
6.8E-06	0.00109	-0.0008	0.0005	0.00162	0.00132
6.9E-06	0.00069	-0.0007	0.00018	0.00149	0.00142
6.9E-06	0.00074	-0.0006	0.00026	0.00241	8E-05
6.9E-06	0.00075	-0.0008	0.001	0.00118	0.00076
7E-06	0.00022	-0.0002	0.00018	0.00128	0.00088
7E-06	0.00045	-0.0011	2.9E-05	0.00163	0.00112
7E-06	0.00051	-0.0007	0.00042	0.00166	0.0005
7.1E-06	0.00069	8E-05	0.00054	0.00128	0.00031
7.1E-06	0.00063	1.9E-05	0.00037	0.00091	0.0008
7.1E-06	0.00031	-0.0012	6.8E-05	0.00049	0.00131
7.2E-06	0.00054	-0.0015	5.7E-05	0.00062	0.00051
7.2E-06	0.00042	-0.0014	0.00012	0.00083	0.00082
7.2E-06	0.00043	-0.0005	0.00062	0.00116	0.00064
7.3E-06	0.00076	-0.0009	0.00028	0.00173	0.00108
7.3E-06	0.00037	-0.0011	1.6E-05	0.00156	0.00103
7.3E-06	0.00017	-0.0003	0.00034	0.00213	0.00018
7.4E-06	-0.0003	-0.0007	0.00043	0.00049	-9E-05
7.4E-06	0.00151	-0.0012	0.00032	0.00156	0.0008
7.4E-06	0.00097	-0.0003	0.0006	0.00088	0.00103
7.5E-06	0.00022	-0.0013	0.00073	0.00105	0.00076

7.5E-06	0.00112	-0.0009	-1E-05	0.00205	0.00068
7.5E-06	7.4E-05	-0.0001	-6E-05	0.00104	0.00059
7.6E-06	0.00042	-0.0006	0.00027	0.00103	0.00117
7.6E-06	0.00108	-0.0009	0.00018	0.00105	0.00069
7.6E-06	0.00133	-0.0008	-0.0001	0.00246	0.00107
7.6E-06	-0.0002	-0.0015	0.0012	0.0017	0.00036
7.7E-06	0.00069	-0.0004	0.00013	0.00093	0.00148
7.7E-06	0.00018	-0.0009	0.00053	0.00135	0.00145
7.7E-06	0.00032	-0.0011	-0.0002	0.00148	0.00045
7.8E-06	0.00115	-0.0008	0.00068	0.00124	0.00038
7.8E-06	0.00064	-0.0011	-0.0002	0.00132	0.00045
7.8E-06	0.00151	-0.0014	5.2E-05	0.00118	0.00098
7.9E-06	0.00082	-0.0011	-0.0003	0.00054	0.00094
7.9E-06	-0.0001	-0.0006	0.00102	0.00167	0.00052
7.9E-06	-4E-05	-0.0005	0.00099	0.00072	0.00077
8E-06	-0.0003	-0.0011	-0.0002	0.00169	0.00082
8E-06	0.0007	-0.001	0.00067	0.00201	0.00028
8E-06	0.00051	-0.0007	0.00041	0.00051	0.00084
8.1E-06	0.00045	-0.0008	0.00034	0.00065	0.00052
8.1E-06	0.00049	-0.0004	0.00059	0.00052	0.00116
8.1E-06	0.00018	-0.0004	0.00049	0.00076	0.00093
8.2E-06	0.00129	-7E-05	0.00081	0.00088	0.0005
8.2E-06	0.00064	-0.0007	0.00014	0.00031	0.00111
8.2E-06	0.00069	-0.0013	-0.0002	0.00153	0.00068
8.3E-06	0.00047	-0.0012	0.00039	0.00136	0.00093
8.3E-06	0.00092	-0.0009	0.00037	0.00061	0.00029
8.3E-06	0.00014	-0.0012	0.00011	0.00065	0.00066
8.4E-06	0.00108	-0.0006	-5E-05	0.00146	0.00053
8.4E-06	0.00115	-0.0007	-0.0001	0.00049	0.0003
8.4E-06	-0.0002	-0.0015	0.00066	0.00145	0.00021
8.4E-06	0.00085	-0.0004	0.00012	0.00067	1E-05
8.5E-06	-0.0001	-0.0007	-0.0005	0.00124	0.00091
8.5E-06	-0.0001	-0.0007	-0.0008	0.00098	0.00037
8.5E-06	0.00055	-0.0004	0.00018	0.00121	0.00117
8.6E-06	0.00062	-0.0009	-3E-05	0.00147	0.0011
8.6E-06	-0.0001	-0.0012	-0.0002	0.00129	0.00101
8.6E-06	0.00044	-0.0013	0.00065	0.00058	0.00086
8.7E-06	0.00033	-0.0009	0.0002	0.00031	0.00096
8.7E-06	0.00073	-0.0013	-6E-06	0.00173	0.00061
8.7E-06	0.00074	-0.0011	-0.0002	0.00085	0.00015
8.8E-06	-0.0003	-0.0007	-0.0008	0.00167	0.00074
8.8E-06	0.0004	-0.0011	0.0005	0.00186	0.00058
8.8E-06	0.00024	-0.0007	0.00052	0.00174	0.00095
8.9E-06	-7E-05	-0.0012	6.6E-05	0.00133	0.00092
8.9E-06	0.0006	-0.0009	0.0001	0.00117	0.00082
8.9E-06	0.00094	-0.0006	0.00059	0.00088	0.0016
9E-06	0.00029	-0.0009	0.00042	0.00193	0.00109
9E-06	0.00081	-0.0003	0.00012	0.00169	0.00138
9E-06	0.0005	-0.0003	0.00014	0.00089	0.0009
9.1E-06	0.0009	-0.0005	0.00075	0.00066	0.00108
9.1E-06	0.00059	-0.0012	0.00018	0.00064	0.00117
9.1E-06	0.00025	0.00029	0.00035	0.00086	0.00093
9.2E-06	0.00039	-0.0003	0.00012	0.00087	0.00054
9.2E-06	0.00046	-0.0008	7.8E-05	0.0007	0.00106

9.2E-06	0.00039	-0.0013	0.00082	0.0002	0.00165
9.2E-06	0.00061	-0.0012	0.00054	0.00172	0.00159
9.3E-06	0.00036	-0.0002	-0.0005	0.00018	0.00138
9.3E-06	0.0004	-0.0007	0.00103	0.00127	0.00029
9.3E-06	0.00063	-0.0002	0.00081	0.00128	0.00105
9.4E-06	0.00111	5.4E-05	0.00025	0.00125	0.00116
9.4E-06	0.00031	-0.0006	0.00044	0.0015	0.00077
9.4E-06	0.00036	-0.0013	0.00015	0.001	0.0007
9.5E-06	4.7E-05	-0.0001	0.00043	0.00132	0.00043
9.5E-06	0.00014	-0.001	-4E-05	0.00145	0.0003
9.5E-06	0.00107	-0.001	-0.001	0.00105	0.00056
9.6E-06	0.00025	-0.0006	0.0004	0.00115	0.00114
9.6E-06	0.0004	-0.0007	0.00075	0.00156	0.00087
9.6E-06	0.0009	-0.0011	0.00015	0.00217	0.00103
9.7E-06	-0.0001	-0.0007	-0.0005	0.00189	0.00137
9.7E-06	-0.0001	-0.001	0.0004	0.00213	0.00091
9.7E-06	0.00068	-0.0009	-0.0002	0.00197	0.00036
9.8E-06	0.00011	-0.0007	0.00021	0.00112	0.00091
9.8E-06	0.00047	-0.0011	0.00041	0.00167	0.00038
9.8E-06	0.00055	-0.0007	0.00072	0.00142	0.00088
9.9E-06	0.00038	-0.0003	0.00096	0.00063	0.00074
9.9E-06	0.00019	-0.0004	0.00045	0.00171	0.00061
9.9E-06	0.00032	-0.0007	-7E-05	0.00191	0.00102
1E-05	0.00044	-0.001	0.00131	0.00137	0.0012
1E-05	0.00101	-0.001	0.00034	0.00174	0.00088
1E-05	0.00089	-0.0007	0.00029	0.00111	0.00053
1E-05	0.00074	0.00047	0.00028	0.00035	0.0012
1E-05	0.00046	-0.0011	0.00042	0.00109	0.00118
1E-05	0.00031	-0.0015	0.00126	0.00163	0.00155
1E-05	0.00078	-0.0006	0.00071	0.00168	0.00052
1E-05	0.00089	-0.0014	-0.0005	0.00153	0.00087
1E-05	0.00084	-0.001	0.00107	0.00171	0.00141
1E-05	0.00058	-0.0015	0.0008	0.00132	0.00091
1E-05	0.00052	-0.0014	-0.0001	0.00139	-0.0001
1E-05	0.00078	-0.0007	-9E-05	0.00078	0.00141
1E-05	0.00017	-0.0011	0.00077	0.0013	0.00156
1E-05	0.00085	-0.0006	0.00013	0.00045	0.00074
1E-05	0.00104	-0.0015	0.00064	0.00123	5.8E-05
1E-05	-0.0002	-0.0005	0.00012	0.0011	0.00074
1E-05	0.00055	-0.0005	0.00042	0.00049	0.00111
1.1E-05	0.00095	-0.001	0.00015	0.00083	0.00122
1.1E-05	0.00068	-0.0006	0.0003	0.00041	0.00126
1.1E-05	0.001	-0.0013	0.00064	0.00096	-0.0004
1.1E-05	0.00061	-0.0011	0.0009	0.00096	0.00099
1.1E-05	0.00052	-0.0001	0.00047	5E-05	-0.0002
1.1E-05	0.00074	-0.0015	0.0012	0.00117	0.0001
1.1E-05	0.00101	-0.0016	-7E-05	0.00131	0.00086
1.1E-05	9.1E-06	-0.0003	0.00071	0.00169	0.00096
1.1E-05	-9E-05	-0.0011	0.00052	0.00151	0.00093
1.1E-05	0.00121	-0.0005	0.00063	0.00137	0.00066
1.1E-05	0.00148	-0.0005	0.00079	0.00095	0.00143
1.1E-05	0.00083	-0.001	-0.0002	0.00137	0.00056
1.1E-05	0.00099	-0.0003	0.00106	0.00107	0.00112
1.1E-05	0.00025	0.00013	0.00101	0.00078	0.00072

1.1E-05	0.00059	0.00058	0.00067	0.00109	0.00056
1.1E-05	0.00142	-0.0006	0.00067	0.00159	0.0009
1.1E-05	0.0007	-0.0004	-6E-05	0.00196	0.00064
1.1E-05	0.00064	-0.0009	0.00096	0.00184	0.00128
1.1E-05	0.00094	-0.0012	0.00132	0.00074	0.00167
1.1E-05	0.00073	-0.0005	0.00121	0.0013	0.00115
1.1E-05	0.00121	-0.0009	0.00034	0.00138	0.0005
1.1E-05	0.00126	-0.002	-0.0003	0.00118	0.00186
1.1E-05	0.00096	-0.0012	0.00039	0.00153	0.00105
1.1E-05	0.0008	-0.0008	0.00102	0.00109	0.0012
1.1E-05	0.00144	-0.0013	3.1E-05	0.00179	0.00114
1.1E-05	0.00083	-0.0016	0.00079	0.00108	0.00137
1.1E-05	0.00104	-0.001	0.00094	0.00107	0.00111
1.1E-05	-2E-05	-0.0007	0.00027	0.00122	0.00033
1.1E-05	0.00041	-0.0006	0.0005	0.00143	0.0006
1.1E-05	0.0003	-0.0007	0.00109	0.00173	0.00082
1.1E-05	0.00104	-0.0013	0.00102	0.00116	0.0008
1.1E-05	0.00018	-0.0005	-0.0001	0.0015	0.00072
1.2E-05	-0.0001	-0.0008	0.0001	0.00153	0.00063
1.2E-05	0.00043	-0.0003	-5E-05	0.00125	0.00028
1.2E-05	0.0011	-0.0008	0.00046	0.00104	0.00096
1.2E-05	0.00049	-0.001	0.00063	0.0013	0.00102
1.2E-05	0.00105	-0.0006	0.00015	0.00214	0.00116
1.2E-05	0.0006	-0.0007	0.0004	0.00186	0.00063
1.2E-05	0.00044	-0.0007	6.6E-06	0.00142	0.0009
1.2E-05	0.00059	-0.0011	7.9E-05	0.00077	0.00094
1.2E-05	0.00064	-0.0012	0.00095	0.00109	0.00013
1.2E-05	0.00105	-0.0006	4.5E-05	0.00089	0.00145
1.2E-05	4.1E-05	-0.0005	0.00063	0.00073	0.00174
1.2E-05	0.00056	-0.0007	0.00072	0.00113	0.00045
1.2E-05	0.00041	-0.0008	0.00032	0.0005	0.00046
1.2E-05	0.00031	-0.0009	0.00092	0.00011	0.0003
1.2E-05	0.00067	-8E-06	2E-05	0.001	0.00045
1.2E-05	0.00025	-0.0012	0.00078	0.00113	0.00073
1.2E-05	0.0011	-0.0009	0.00043	0.00111	0.00115
1.2E-05	0.00096	-0.0011	0.00051	0.00133	0.00087
1.2E-05	0.00013	-0.0015	0.00047	0.00094	0.00085
1.2E-05	0.00037	-0.0011	0.00137	0.0011	0.00073
1.2E-05	0.00119	-0.0007	0.00077	0.00121	0.00062
1.2E-05	0.00032	-0.0009	-0.0004	0.001	0.00061
1.2E-05	0.00085	-0.0018	0.00056	0.00134	0.0014
1.2E-05	-0.0003	-0.0008	0.00034	0.00038	0.00024
1.2E-05	-5E-05	-0.0001	0.00093	0.00116	0.00107
1.2E-05	0.00023	-0.0009	0.00062	0.00149	0.00089
1.2E-05	0.00033	-0.0009	9.9E-05	0.00103	0.00137
1.2E-05	0.00057	-0.0007	9E-05	0.00168	0.0007
1.2E-05	0.00102	-0.0014	0.00071	0.0016	0.00196
1.2E-05	0.00026	-0.0016	0.00101	0.00161	0.00027
1.2E-05	0.00042	-0.001	0.00109	0.00149	0.00047
1.3E-05	0.00024	-0.0013	0.00094	0.0008	0.0013
1.3E-05	5.7E-06	-0.0007	0.0004	0.00086	0.00108
1.3E-05	6.3E-05	-0.0003	0.00036	0.00105	0.00181
1.3E-05	0.00022	-0.001	0.00041	0.00089	0.0011
1.3E-05	0.00032	-0.0013	0.00079	0.00137	0.00126

1.3E-05	5.2E-05	-0.0011	0.00069	0.00133	-3E-05
1.3E-05	9.2E-05	-0.001	0.00124	0.00084	0.00108
1.3E-05	0.00111	-0.0002	0.00114	0.00103	0.00066
1.3E-05	0.00042	-0.0003	0.0012	0.00163	0.00078
1.3E-05	0.00068	-0.0014	0.00086	0.00145	0.00083
1.3E-05	0.00104	-0.0013	0.00055	0.00164	0.00017
1.3E-05	0.00123	-0.0013	0.00095	0.00165	0.00091
1.3E-05	0.00026	-0.0007	8.1E-05	0.00152	0.00123
1.3E-05	0.00043	-0.0012	0.00038	0.0009	0.00076
1.3E-05	0.00011	-0.0006	-0.0006	0.00068	0.00111
1.3E-05	0.00026	-0.0008	0.00021	0.00096	0.00074
1.3E-05	0.00112	-0.0007	0.00032	0.00136	-0.0002
1.3E-05	0.00088	-0.0016	0.00013	0.00125	0.00015
1.3E-05	0.00024	-0.001	0.00041	0.00156	0.00066
1.3E-05	0.00125	-0.001	4.9E-06	0.00078	0.00111
1.3E-05	0.00108	-0.0013	0.00027	0.00118	0.00143
1.3E-05	0.0004	-0.0011	0.00056	0.00132	0.0002
1.3E-05	0.00037	-0.0009	0.00059	0.0008	0.00082
1.3E-05	0.00081	-0.0002	0.00011	0.00119	0.00094
1.3E-05	0.00029	-0.0012	1.6E-05	0.0011	0.00075
1.3E-05	0.00093	-0.0005	0.00032	0.00177	0.00038
1.3E-05	0.00084	-0.0011	0.00052	0.00151	0.00149
1.3E-05	0.00117	-0.0013	0.00068	0.00147	0.00159
1.3E-05	0.00031	-0.0008	0.00038	0.00088	0.00093
1.3E-05	0.00011	-0.0007	-0.0006	0.00086	0.00057
1.3E-05	0.00014	-0.0007	-0.0005	0.00147	0.00072
1.4E-05	0.00097	-0.0008	-0.0002	0.00107	0.00057
1.4E-05	0.00077	-0.0006	-8E-06	0.00207	0.00045
1.4E-05	0.0006	-0.0013	0.00046	0.00153	0.00063
1.4E-05	0.00055	-0.0008	0.00038	0.00093	0.0001
1.4E-05	0.00059	-0.0005	6.6E-05	0.00122	0.00046
1.4E-05	0.00089	-0.0003	-0.0005	0.00086	0.00096
1.4E-05	0.00076	-0.0015	0.00024	0.00071	0.00067
1.4E-05	0.0003	-0.0009	0.00065	0.0014	0.00097
1.4E-05	0.0003	-0.0008	0.00017	0.00105	0.00071
1.4E-05	0.00094	-0.0009	0.00026	0.00181	5.6E-05
1.4E-05	0.00017	-0.0009	-0.0002	0.00116	0.00116
1.4E-05	-8E-05	-1E-04	0.0002	0.00105	0.00089
1.4E-05	0.00032	-0.0006	0.00049	0.00154	0.00161
1.4E-05	0.001	-0.0009	0.00067	0.00104	0.00054
1.4E-05	0.00078	-0.0005	0.00071	0.00089	0.00088
1.4E-05	0.00049	-0.0013	0.00093	0.00118	0.00098
1.4E-05	0.00061	-0.0011	0.00069	0.00142	0.00052
1.4E-05	-0.0002	-0.001	0.00049	0.0016	0.00072
1.4E-05	0.00087	-0.0005	0.00013	0.00093	0.00068
1.4E-05	0.0007	-0.0009	0.00031	0.00119	0.00047
1.4E-05	0.00079	-0.0003	0.00041	0.0009	0.00055
1.4E-05	0.00106	-0.0007	0.00075	0.00101	0.00097
1.4E-05	0.00055	-0.0011	0.0001	0.00153	0.0007
1.4E-05	0.00061	-0.0013	0.00027	0.00148	0.0006
1.4E-05	0.00084	-0.0009	0.00041	0.00082	0.00124
1.4E-05	0.00128	-0.0005	7.7E-07	0.00076	0.00045
1.4E-05	0.00058	-0.0004	0.00049	0.00105	0.00147
1.4E-05	0.00097	-0.0006	0.0003	0.00142	0.00035

1.4E-05	0.00104	-0.0001	-0.0004	0.00118	0.00067
1.4E-05	0.00032	-0.001	3.6E-05	0.00133	0.00153
1.4E-05	0.00034	-0.0011	-0.0008	0.0002	0.00093
1.5E-05	0.00042	-0.0011	0.00067	0.00115	0.00024
1.5E-05	0.00011	-0.0018	0.00026	0.00129	0.00047
1.5E-05	0.00026	-0.0005	-8E-06	0.00165	0.00038
1.5E-05	0.00168	7.2E-05	0.00049	0.00136	0.00063
1.5E-05	0.00046	-0.0009	0.00037	0.00127	0.00094
1.5E-05	0.00027	-0.0009	-1E-05	0.00113	0.00082
1.5E-05	0.00051	-0.0009	0.0004	0.00117	0.00099
1.5E-05	0.00132	-0.0012	0.00023	0.00104	0.00011
1.5E-05	0.00069	-0.0006	0.00076	0.00083	0.00115
1.5E-05	0.001	-0.0003	0.00049	0.00103	0.00101
1.5E-05	0.00118	-0.0008	0.00048	0.00137	0.00118
1.5E-05	-5E-05	-0.0011	0.00069	0.00068	1.7E-05
1.5E-05	-0.0003	-0.0012	0.00081	0.00139	0.00079
1.5E-05	0.00028	-0.0008	0.00103	0.00075	0.00103
1.5E-05	0.00021	-0.0012	-0.0008	0.0014	0.00041
1.5E-05	0.00096	-0.0009	-0.0003	0.00132	0.00072

Table SI.14B: Primary kinetic data for the reaction of 2-imidazolidinone (**S14**) with CumO$^{\bullet}$ generated by 355 nm LFP in DMSO (**Figure SI.14B**).

**t / s**	**ΔA 2-imidazolidinone (S14)**				
	**0.075 M**	**0.056 M**	**0.0375 M**	**0.025 M**	**0.0075 M**
-9.92E-07	-5.46E-04	-2.95E-04	1.09E-04	-2.99E-06	-4.48E-05
-9.60E-07	-2.28E-04	-5.05E-04	-1.35E-04	3.60E-04	-3.66E-04
-9.28E-07	4.98E-05	-4.62E-04	-5.24E-05	2.89E-04	1.20E-04
-8.96E-07	-6.07E-04	-2.98E-04	5.57E-04	1.49E-04	-7.88E-04
-8.64E-07	-4.82E-04	-3.67E-04	2.59E-04	5.58E-04	-5.51E-04
-8.32E-07	1.30E-04	-3.14E-04	3.04E-04	2.26E-04	-1.84E-04
-8.00E-07	2.60E-04	-3.27E-04	-7.37E-04	6.20E-04	4.35E-05
-7.68E-07	2.47E-04	-6.04E-04	2.99E-04	6.79E-04	-3.86E-04
-7.36E-07	-4.29E-04	-3.75E-04	-5.59E-05	5.15E-05	-3.28E-04
-7.04E-07	-3.64E-05	-3.87E-04	2.61E-04	5.55E-05	-1.94E-04
-6.72E-07	-2.46E-04	-3.72E-04	4.93E-04	2.17E-04	-3.35E-04
-6.40E-07	-6.04E-04	-5.58E-04	9.11E-04	5.05E-04	-2.82E-04
-6.08E-07	-1.93E-04	-4.57E-04	4.74E-04	9.55E-04	-3.23E-04
-5.76E-07	-5.19E-04	-5.65E-04	9.45E-05	5.62E-04	-6.55E-05
-5.44E-07	-5.38E-04	-3.25E-04	4.81E-04	2.79E-04	-1.95E-04
-5.12E-07	-2.19E-04	-3.72E-04	1.82E-04	5.27E-04	-2.22E-04
-4.80E-07	-3.08E-04	-4.70E-04	-2.42E-04	7.09E-04	-1.09E-04
-4.48E-07	-3.52E-04	-3.98E-04	-3.31E-04	5.53E-04	7.53E-05
-4.16E-07	2.36E-04	-3.22E-04	-6.90E-05	2.42E-04	3.17E-04
-3.84E-07	-2.87E-04	-2.38E-05	1.13E-04	3.88E-04	-8.08E-04
-3.52E-07	-3.98E-04	-2.37E-04	4.35E-04	-2.30E-04	-4.19E-04
-3.20E-07	-6.27E-04	-5.05E-05	-3.07E-04	-4.22E-04	-4.09E-04
-2.88E-07	-5.22E-04	-2.77E-04	-1.17E-04	4.81E-04	-1.79E-04
-2.56E-07	-6.46E-04	-3.77E-04	5.53E-04	4.62E-04	-6.02E-04
-2.24E-07	-1.24E-03	-3.28E-04	-4.91E-05	4.38E-04	-8.26E-04
-1.92E-07	-6.14E-04	-8.19E-04	1.49E-04	1.45E-04	-6.07E-04
-1.60E-07	-8.95E-04	-7.01E-04	4.22E-04	2.14E-04	-7.33E-04
-1.28E-07	1.25E-04	-6.77E-04	4.70E-04	1.63E-04	-5.51E-04
-9.64E-08	-3.85E-04	-8.34E-04	2.67E-04	4.51E-04	-2.40E-04
-6.44E-08	-6.62E-04	-5.42E-04	2.35E-04	-1.09E-04	-1.06E-04
-3.24E-08	-4.96E-04	-6.29E-04	4.37E-05	9.80E-05	-1.11E-04
-3.75E-10	-3.75E-03	-2.91E-03	-2.34E-03	-2.69E-03	-3.13E-03
3.16E-08	-1.53E-02	-1.03E-02	-9.54E-03	-1.19E-02	-8.93E-03
6.36E-08	1.07E-02	1.13E-02	1.33E-02	1.40E-02	1.44E-02
9.56E-08	1.20E-02	1.30E-02	1.45E-02	1.51E-02	1.44E-02
1.28E-07	1.15E-02	1.24E-02	1.45E-02	1.37E-02	1.48E-02
1.60E-07	1.10E-02	1.14E-02	1.28E-02	1.35E-02	1.44E-02
1.92E-07	1.03E-02	1.06E-02	1.24E-02	1.35E-02	1.40E-02
2.24E-07	9.30E-03	1.02E-02	1.17E-02	1.26E-02	1.38E-02
2.56E-07	8.87E-03	9.57E-03	1.20E-02	1.19E-02	1.33E-02
2.88E-07	8.18E-03	8.49E-03	1.15E-02	1.17E-02	1.28E-02
3.20E-07	7.77E-03	8.04E-03	1.07E-02	1.18E-02	1.28E-02
3.52E-07	7.40E-03	7.58E-03	1.01E-02	1.15E-02	1.19E-02
3.84E-07	6.53E-03	7.53E-03	9.53E-03	1.06E-02	1.18E-02
4.16E-07	5.84E-03	7.07E-03	8.85E-03	9.75E-03	1.12E-02
4.48E-07	5.39E-03	6.39E-03	9.22E-03	1.00E-02	1.08E-02
4.80E-07	4.98E-03	5.91E-03	8.07E-03	8.89E-03	1.12E-02
5.12E-07	5.26E-03	5.65E-03	8.23E-03	8.79E-03	1.02E-02
5.44E-07	4.99E-03	5.76E-03	7.37E-03	8.93E-03	1.09E-02

5.76E-07	3.44E-03	5.45E-03	6.56E-03	7.98E-03	9.44E-03
6.08E-07	3.45E-03	5.15E-03	6.28E-03	7.69E-03	9.05E-03
6.40E-07	3.46E-03	4.76E-03	6.16E-03	7.83E-03	9.29E-03
6.72E-07	3.36E-03	4.97E-03	6.50E-03	7.52E-03	9.18E-03
7.04E-07	2.68E-03	4.02E-03	6.12E-03	7.12E-03	8.49E-03
7.36E-07	2.93E-03	3.76E-03	5.95E-03	6.31E-03	8.20E-03
7.68E-07	2.64E-03	3.16E-03	5.42E-03	6.97E-03	7.92E-03
8.00E-07	2.72E-03	2.90E-03	5.41E-03	6.05E-03	8.38E-03
8.32E-07	2.26E-03	2.56E-03	4.73E-03	6.15E-03	7.44E-03
8.64E-07	2.01E-03	2.61E-03	4.77E-03	5.90E-03	7.19E-03
8.96E-07	1.62E-03	2.65E-03	4.31E-03	5.61E-03	7.22E-03
9.28E-07	1.34E-03	2.08E-03	3.93E-03	5.44E-03	7.17E-03
9.60E-07	1.22E-03	2.61E-03	4.18E-03	5.25E-03	7.08E-03
9.92E-07	1.10E-03	2.21E-03	4.25E-03	5.11E-03	6.76E-03
1.02E-06	1.20E-03	1.79E-03	3.83E-03	5.34E-03	6.45E-03
1.06E-06	1.77E-03	1.80E-03	3.51E-03	4.44E-03	6.81E-03
1.09E-06	1.26E-03	1.66E-03	3.73E-03	4.39E-03	6.01E-03
1.12E-06	1.25E-03	1.74E-03	4.13E-03	4.20E-03	6.28E-03
1.15E-06	1.38E-03	1.35E-03	2.95E-03	4.10E-03	6.05E-03
1.18E-06	1.25E-03	1.02E-03	2.92E-03	3.85E-03	6.07E-03
1.22E-06	6.19E-04	1.36E-03	2.97E-03	4.37E-03	5.63E-03
1.25E-06	1.45E-03	1.54E-03	2.14E-03	3.35E-03	4.79E-03
1.28E-06	1.08E-03	1.39E-03	2.05E-03	4.15E-03	5.48E-03
1.31E-06	7.87E-04	1.19E-03	2.48E-03	3.40E-03	5.36E-03
1.34E-06	9.52E-04	1.29E-03	2.38E-03	3.37E-03	5.14E-03
1.38E-06	8.56E-04	1.05E-03	2.51E-03	3.74E-03	5.02E-03
1.41E-06	7.74E-04	6.13E-04	2.39E-03	3.49E-03	4.86E-03
1.44E-06	8.86E-04	6.29E-04	2.74E-03	2.70E-03	4.64E-03
1.47E-06	1.06E-03	9.43E-04	2.18E-03	3.25E-03	4.84E-03
1.50E-06	3.53E-04	8.79E-04	2.09E-03	3.18E-03	4.87E-03
1.54E-06	7.72E-05	1.08E-03	1.49E-03	3.30E-03	4.10E-03
1.57E-06	6.21E-04	7.93E-04	1.72E-03	2.69E-03	3.88E-03
1.60E-06	9.81E-04	4.34E-04	1.82E-03	3.01E-03	3.69E-03
1.63E-06	1.87E-04	5.41E-04	1.23E-03	2.88E-03	3.92E-03
1.66E-06	7.60E-04	3.34E-04	1.75E-03	2.02E-03	3.89E-03
1.70E-06	5.76E-04	1.55E-04	1.75E-03	2.42E-03	3.25E-03
1.73E-06	8.84E-04	6.93E-04	1.61E-03	1.97E-03	3.47E-03
1.76E-06	3.64E-04	-5.59E-05	1.80E-03	2.03E-03	3.64E-03
1.79E-06	5.35E-04	1.87E-04	1.58E-03	1.44E-03	3.62E-03
1.82E-06	-3.78E-05	6.31E-04	1.36E-03	1.57E-03	3.76E-03
1.86E-06	-5.73E-05	2.46E-04	1.53E-03	2.11E-03	3.45E-03
1.89E-06	5.42E-04	2.42E-04	1.81E-03	1.69E-03	3.65E-03
1.92E-06	-3.79E-04	9.67E-05	1.13E-03	1.86E-03	3.30E-03
1.95E-06	2.65E-04	5.61E-04	1.27E-03	1.53E-03	3.10E-03
1.98E-06	1.11E-03	5.79E-04	1.45E-03	1.65E-03	3.35E-03
2.02E-06	4.88E-04	2.57E-04	1.37E-03	1.53E-03	3.25E-03
2.05E-06	4.60E-04	8.21E-04	1.40E-03	1.84E-03	3.20E-03
2.08E-06	1.37E-04	4.55E-04	1.65E-03	1.38E-03	3.25E-03
2.11E-06	-5.15E-05	7.00E-05	1.41E-03	1.55E-03	3.03E-03
2.14E-06	-1.19E-05	3.68E-05	1.91E-03	1.44E-03	2.81E-03
2.18E-06	-5.35E-05	2.88E-04	1.62E-03	1.78E-03	2.89E-03
2.21E-06	1.02E-04	1.89E-05	1.33E-03	1.34E-03	3.35E-03
2.24E-06	5.54E-05	6.88E-05	1.06E-03	1.17E-03	2.61E-03
2.27E-06	-4.51E-05	-2.41E-04	1.26E-03	1.25E-03	2.39E-03

2.30E-06	-5.56E-04	3.16E-04	1.40E-03	1.04E-03	2.56E-03
2.34E-06	3.55E-04	-2.55E-04	1.11E-03	1.16E-03	2.80E-03
2.37E-06	-1.29E-04	1.90E-04	1.36E-03	1.33E-03	2.75E-03
2.40E-06	-4.78E-04	1.82E-04	9.34E-04	1.72E-03	2.93E-03
2.43E-06	-1.36E-04	3.32E-04	1.50E-03	1.49E-03	2.41E-03
2.46E-06	4.49E-04	3.19E-04	1.99E-03	8.77E-04	2.62E-03
2.50E-06	-1.17E-06	4.08E-04	9.23E-04	1.46E-03	2.26E-03
2.53E-06	2.88E-04	2.89E-04	1.30E-03	8.01E-04	2.54E-03
2.56E-06	1.28E-04	-4.07E-05	3.98E-04	1.17E-03	1.72E-03
2.59E-06	1.56E-04	5.80E-04	1.10E-03	9.70E-04	2.41E-03
2.62E-06	-2.34E-04	6.46E-05	9.52E-04	9.34E-04	2.80E-03
2.66E-06	3.24E-04	-1.11E-04	8.69E-04	1.48E-03	2.30E-03
2.69E-06	-7.46E-04	-1.59E-06	9.76E-04	8.58E-04	2.14E-03
2.72E-06	-6.16E-04	-4.94E-05	7.87E-04	9.63E-04	2.46E-03
2.75E-06	-2.15E-04	1.11E-04	7.25E-04	7.31E-04	2.26E-03
2.78E-06	-1.51E-04	8.93E-05	7.11E-04	1.14E-03	1.97E-03
2.82E-06	-1.75E-04	-1.58E-05	1.35E-03	1.75E-03	1.86E-03
2.85E-06	1.06E-04	3.71E-05	3.82E-04	1.03E-03	2.28E-03
2.88E-06	-2.74E-04	5.40E-04	4.21E-04	8.78E-04	1.72E-03
2.91E-06	2.44E-04	7.65E-05	4.31E-04	7.43E-04	1.89E-03
2.94E-06	-4.15E-05	-3.31E-04	6.75E-04	8.81E-04	1.55E-03
2.98E-06	5.65E-05	-5.80E-05	7.53E-04	8.35E-04	1.71E-03
3.01E-06	-5.66E-04	-1.19E-04	1.52E-03	7.72E-04	1.98E-03
3.04E-06	-1.26E-04	-8.15E-05	6.92E-04	8.31E-04	1.84E-03
3.07E-06	-5.36E-04	2.64E-05	5.02E-04	5.16E-04	8.99E-04
3.10E-06	4.16E-04	1.11E-04	8.14E-04	4.23E-04	1.98E-03
3.14E-06	-3.89E-04	-5.82E-05	7.77E-04	1.05E-03	2.00E-03
3.17E-06	1.55E-04	-2.76E-04	7.25E-04	9.31E-04	1.94E-03
3.20E-06	-3.82E-04	2.45E-05	8.69E-04	1.27E-03	1.56E-03
3.23E-06	-2.30E-04	-3.65E-04	6.57E-04	8.28E-04	1.46E-03
3.26E-06	4.24E-05	1.49E-04	6.64E-04	7.08E-04	1.95E-03
3.30E-06	-5.62E-04	3.16E-04	1.15E-03	8.91E-04	1.04E-03
3.33E-06	-2.07E-05	-7.42E-04	5.65E-04	8.06E-04	1.75E-03
3.36E-06	-2.64E-04	-5.21E-04	1.05E-03	3.38E-04	1.53E-03
3.39E-06	-4.61E-04	-2.48E-05	8.27E-04	9.65E-04	1.39E-03
3.42E-06	-4.10E-04	-3.39E-04	9.97E-04	6.87E-04	9.97E-04
3.46E-06	1.72E-04	-4.89E-04	6.09E-04	8.37E-04	1.53E-03
3.49E-06	-7.11E-04	5.54E-06	7.29E-04	9.46E-04	1.04E-03
3.52E-06	4.00E-04	7.46E-05	5.17E-04	9.76E-04	1.23E-03
3.55E-06	1.10E-05	1.38E-04	3.36E-04	1.08E-03	1.14E-03
3.58E-06	-5.89E-05	-4.64E-04	4.88E-04	3.58E-04	9.87E-04
3.62E-06	-2.62E-04	-6.10E-05	4.03E-04	4.64E-04	1.25E-03
3.65E-06	-6.17E-05	1.23E-04	3.00E-04	8.57E-04	1.26E-03
3.68E-06	6.14E-05	-2.91E-04	1.36E-03	2.45E-04	1.11E-03
3.71E-06	-3.26E-04	-1.31E-04	6.47E-04	6.90E-04	1.72E-03
3.74E-06	2.51E-04	-5.13E-04	9.49E-04	2.46E-04	5.00E-04
3.78E-06	-3.48E-04	-1.09E-04	9.78E-04	2.81E-04	1.21E-03
3.81E-06	-4.74E-04	-2.54E-04	1.43E-04	3.34E-04	1.42E-03
3.84E-06	-2.62E-04	6.88E-05	5.59E-04	8.04E-04	9.56E-04
3.87E-06	-3.89E-04	-3.18E-04	7.53E-04	9.63E-04	4.24E-04
3.90E-06	-4.44E-05	-5.13E-04	8.34E-04	7.43E-04	1.11E-03
3.94E-06	-3.78E-04	-1.71E-04	9.21E-04	6.78E-04	9.52E-04
3.97E-06	-1.82E-04	-1.29E-04	9.77E-04	1.04E-03	1.08E-03
4.00E-06	-6.94E-05	-3.02E-04	9.00E-04	8.12E-04	1.21E-03

4.03E-06	4.06E-05	-5.74E-04	1.07E-03	6.35E-04	6.00E-04
4.06E-06	-6.35E-04	-5.98E-04	4.85E-04	7.63E-04	1.23E-03
4.10E-06	-4.59E-04	-2.70E-04	5.54E-04	6.94E-04	1.04E-03
4.13E-06	-6.02E-04	-2.17E-04	4.64E-04	9.86E-04	1.14E-03
4.16E-06	-1.76E-04	3.61E-05	4.20E-04	5.92E-04	9.78E-04
4.19E-06	-7.19E-04	1.39E-04	1.16E-04	3.43E-05	1.32E-03
4.22E-06	-2.03E-04	-3.80E-04	4.83E-04	7.26E-04	1.03E-03
4.26E-06	-7.11E-04	-2.99E-04	3.53E-04	6.22E-04	1.08E-03
4.29E-06	-3.07E-04	-3.27E-04	4.54E-04	1.87E-04	9.20E-04
4.32E-06	-4.49E-04	-5.15E-04	2.75E-04	5.13E-04	1.43E-03
4.35E-06	-2.18E-04	5.54E-06	4.25E-04	2.39E-04	1.03E-03
4.38E-06	-5.92E-04	-2.84E-04	6.30E-04	2.24E-04	9.77E-04
4.42E-06	-9.14E-05	-4.39E-04	8.72E-04	9.39E-04	1.88E-03
4.45E-06	-4.20E-04	-2.65E-04	7.81E-04	9.32E-04	1.08E-03
4.48E-06	-9.29E-05	-4.96E-04	2.00E-04	1.06E-03	1.26E-03
4.51E-06	-1.25E-04	7.02E-05	5.49E-04	1.26E-03	1.24E-03
4.54E-06	2.61E-04	-9.92E-05	7.71E-04	5.46E-04	1.07E-03
4.58E-06	4.29E-05	-4.47E-04	1.42E-04	6.21E-04	1.14E-03
4.61E-06	-2.71E-04	-7.21E-05	7.09E-04	3.69E-04	6.91E-04
4.64E-06	3.23E-05	-2.07E-04	8.98E-04	8.98E-04	9.19E-04
4.67E-06	-8.96E-05	-3.96E-04	5.80E-04	5.10E-04	1.08E-03
4.70E-06	-3.49E-04	-5.31E-04	4.41E-04	7.73E-05	9.73E-04
4.74E-06	1.79E-04	-8.84E-05	4.30E-04	6.97E-04	1.26E-03
4.77E-06	5.40E-04	-5.84E-04	1.14E-04	2.74E-04	7.49E-04
4.80E-06	1.84E-05	-3.63E-04	7.51E-04	4.00E-05	9.08E-04
4.83E-06	-7.66E-05	-2.42E-04	9.35E-04	2.21E-04	8.16E-04
4.86E-06	3.09E-04	-1.88E-04	6.01E-04	9.45E-04	8.54E-04
4.90E-06	-1.87E-04	-2.62E-05	9.43E-04	7.76E-04	9.16E-04
4.93E-06	-2.18E-04	-1.04E-04	2.59E-04	4.87E-04	1.26E-03
4.96E-06	2.36E-04	-2.31E-04	8.14E-04	6.75E-05	9.14E-04
4.99E-06	-6.31E-04	6.27E-05	1.06E-03	2.17E-04	1.04E-03
5.02E-06	-7.49E-04	-1.40E-04	7.15E-04	3.17E-04	7.51E-04
5.06E-06	-4.09E-04	-2.45E-04	6.09E-05	7.08E-04	1.01E-03
5.09E-06	-2.39E-04	-6.23E-05	3.07E-04	6.43E-04	1.02E-03
5.12E-06	-3.19E-04	5.15E-05	3.56E-04	3.75E-04	5.08E-04
5.15E-06	-4.48E-04	-1.16E-04	3.38E-04	2.97E-04	7.63E-04
5.18E-06	1.54E-04	-2.52E-04	1.81E-04	7.36E-04	1.37E-03
5.22E-06	-3.11E-04	-8.02E-05	4.56E-04	4.35E-04	7.36E-04
5.25E-06	5.37E-04	1.33E-04	1.35E-04	-1.08E-04	1.11E-03
5.28E-06	8.20E-05	-1.93E-04	8.88E-05	3.87E-04	1.29E-03
5.31E-06	9.22E-05	-1.03E-04	5.91E-04	1.02E-03	1.18E-03
5.34E-06	-4.18E-04	-1.99E-04	6.96E-04	6.06E-04	1.17E-03
5.38E-06	-3.87E-04	-5.00E-05	4.12E-04	4.70E-06	1.07E-03
5.41E-06	-1.26E-04	-1.40E-04	7.45E-04	3.98E-04	7.03E-04
5.44E-06	-3.61E-04	-2.47E-04	5.02E-04	4.21E-04	5.35E-04
5.47E-06	-3.33E-05	-1.18E-04	3.00E-04	1.88E-04	8.01E-04
5.50E-06	-1.52E-04	1.33E-04	7.67E-04	-8.87E-05	1.16E-03
5.54E-06	-2.45E-04	-2.56E-04	8.48E-04	4.46E-04	8.09E-04
5.57E-06	-3.92E-06	-3.98E-04	3.69E-04	3.59E-04	8.51E-04
5.60E-06	-5.64E-04	-1.70E-04	5.00E-04	-3.01E-04	7.33E-04
5.63E-06	1.18E-04	-5.75E-06	5.55E-04	4.37E-04	1.11E-03
5.66E-06	-7.66E-04	-1.34E-04	8.42E-04	3.67E-04	9.08E-04
5.70E-06	-5.33E-04	-1.73E-04	3.80E-04	5.90E-04	1.36E-03
5.73E-06	-2.42E-04	-3.43E-04	8.88E-04	-1.76E-04	7.12E-04

5.76E-06	-8.35E-04	-1.66E-05	1.26E-03	-1.35E-04	3.74E-04
5.79E-06	-4.58E-05	-2.14E-04	9.91E-04	-6.68E-05	-1.19E-04
5.82E-06	-3.27E-04	-1.86E-04	1.32E-03	2.03E-04	2.85E-04
5.86E-06	-3.62E-04	-4.62E-04	1.07E-04	-6.69E-04	5.64E-04
5.89E-06	-2.45E-04	-1.25E-04	1.55E-04	3.76E-06	2.66E-04
5.92E-06	-5.52E-04	-3.41E-04	2.84E-04	4.56E-04	4.21E-04
5.95E-06	-2.65E-04	-3.31E-04	7.32E-04	8.73E-05	6.63E-04
5.98E-06	1.21E-04	3.70E-04	1.73E-04	6.67E-04	8.19E-04
6.02E-06	-3.43E-04	-8.71E-05	1.43E-04	-6.30E-05	1.19E-03
6.05E-06	-2.01E-04	-9.32E-05	4.97E-04	5.66E-04	1.14E-03
6.08E-06	-7.93E-04	-2.72E-04	2.87E-04	4.37E-04	4.02E-04
6.11E-06	-8.05E-04	-2.32E-04	1.10E-04	4.76E-04	1.27E-03
6.14E-06	-6.31E-04	-2.94E-04	6.33E-04	-1.37E-04	1.09E-03
6.18E-06	-4.84E-04	-6.06E-05	5.08E-04	1.75E-04	1.32E-03
6.21E-06	-7.17E-04	-9.15E-05	5.93E-04	6.90E-04	3.84E-04
6.24E-06	-2.72E-04	-1.11E-04	1.08E-04	-5.35E-06	1.08E-03
6.27E-06	-8.65E-04	-3.19E-04	9.85E-04	-3.61E-04	1.07E-03
6.30E-06	-2.46E-04	3.01E-05	1.77E-04	1.54E-04	6.16E-04
6.34E-06	-6.03E-05	-3.83E-04	4.48E-04	8.41E-05	8.55E-04
6.37E-06	-5.59E-04	-5.56E-04	6.57E-04	5.85E-04	7.51E-04
6.40E-06	-3.16E-04	5.26E-06	5.35E-04	1.07E-04	8.10E-04
6.43E-06	-6.25E-04	-4.42E-04	-1.01E-05	-1.31E-05	5.76E-04
6.46E-06	-2.60E-04	-2.60E-04	-3.44E-04	-2.36E-05	5.25E-04
6.50E-06	8.47E-05	-3.55E-04	3.26E-04	3.38E-04	7.23E-04
6.53E-06	-6.36E-04	-2.22E-04	4.85E-06	1.60E-04	1.13E-03
6.56E-06	-4.71E-04	-2.09E-04	-1.05E-04	3.87E-05	7.32E-04
6.59E-06	-4.43E-04	-1.82E-04	2.82E-04	5.49E-04	8.31E-04
6.62E-06	-2.18E-04	-5.88E-04	6.87E-04	7.67E-04	8.92E-04
6.66E-06	-9.15E-04	-8.16E-05	1.22E-03	3.31E-04	8.45E-04
6.69E-06	-4.65E-04	-1.45E-04	-1.73E-04	-3.95E-04	1.16E-03
6.72E-06	-1.06E-03	-1.18E-04	6.35E-05	-1.44E-04	6.65E-04
6.75E-06	-9.31E-05	-6.17E-05	1.78E-06	-2.28E-04	6.37E-04
6.78E-06	3.59E-04	-5.55E-04	3.51E-04	4.35E-04	9.16E-04
6.82E-06	-8.77E-04	-4.25E-04	2.41E-04	5.46E-04	9.84E-04
6.85E-06	-1.71E-04	-2.01E-04	9.06E-04	-2.15E-04	1.45E-03
6.88E-06	-7.93E-04	-2.23E-04	7.29E-04	5.54E-04	1.37E-03
6.91E-06	2.76E-04	-1.30E-04	3.31E-04	3.74E-04	9.11E-04
6.94E-06	-5.77E-04	-4.52E-04	-1.97E-04	4.77E-04	5.55E-04
6.98E-06	-5.08E-04	1.34E-04	3.65E-04	-3.55E-04	1.22E-03
7.01E-06	1.33E-04	-2.14E-04	3.31E-04	1.78E-04	1.09E-03
7.04E-06	-1.30E-05	-2.88E-04	3.99E-04	3.64E-04	1.06E-03
7.07E-06	-5.61E-04	3.51E-04	2.35E-04	2.31E-04	8.72E-04
7.10E-06	-2.57E-04	-2.53E-04	-1.76E-04	-7.31E-05	6.94E-04
7.14E-06	-5.98E-04	3.89E-04	4.82E-04	1.98E-04	1.20E-03
7.17E-06	-2.80E-04	-1.56E-04	6.37E-04	-2.10E-04	1.18E-03
7.20E-06	-2.37E-04	-1.61E-05	1.01E-03	-7.22E-05	-3.36E-04
7.23E-06	-7.31E-05	-1.56E-04	4.00E-04	2.68E-04	8.86E-04
7.26E-06	-2.76E-04	-1.05E-04	1.99E-04	4.16E-05	7.10E-04
7.30E-06	-2.28E-04	3.23E-04	2.47E-04	2.06E-04	4.40E-04
7.33E-06	-9.85E-04	-1.33E-04	4.28E-04	-1.48E-04	8.83E-04
7.36E-06	-6.31E-04	-4.49E-04	9.29E-04	-2.40E-04	1.04E-03
7.39E-06	-8.03E-04	-5.44E-04	8.65E-04	-1.65E-04	6.41E-04
7.42E-06	-7.42E-04	-3.20E-05	7.62E-04	-3.80E-04	8.59E-05
7.46E-06	-6.96E-04	-6.25E-05	2.55E-04	3.48E-04	2.11E-04

7.49E-06	-7.45E-04	-3.32E-04	4.10E-04	-4.10E-05	3.05E-04
7.52E-06	-3.35E-04	-3.34E-04	-2.22E-04	1.85E-04	3.64E-04
7.55E-06	6.70E-04	-4.48E-04	-5.49E-05	-2.09E-04	9.11E-04
7.58E-06	-7.75E-04	-4.06E-05	1.82E-04	7.67E-05	1.11E-03
7.62E-06	-4.04E-04	-9.35E-04	8.01E-04	-8.51E-05	6.82E-04
7.65E-06	-7.13E-04	-9.07E-05	8.89E-04	1.02E-04	1.21E-03
7.68E-06	-4.24E-04	7.81E-05	3.44E-04	8.79E-04	2.98E-04
7.71E-06	-1.27E-04	-3.81E-05	3.46E-04	6.29E-04	1.22E-03
7.74E-06	-8.17E-04	-4.93E-04	9.25E-04	-9.61E-05	1.05E-03
7.78E-06	-5.39E-04	-2.39E-04	5.29E-04	-5.94E-04	1.10E-03
7.81E-06	-4.80E-04	-4.62E-04	4.11E-04	8.15E-06	3.98E-04
7.84E-06	-1.58E-04	-1.09E-04	9.20E-05	2.09E-04	6.18E-04
7.87E-06	-1.01E-03	1.21E-04	3.49E-04	4.77E-04	4.57E-04
7.90E-06	-8.17E-04	-4.96E-04	3.98E-04	-9.19E-05	5.94E-04
7.94E-06	-3.58E-04	-9.80E-05	3.45E-04	-2.30E-04	5.20E-04
7.97E-06	6.30E-05	-1.84E-04	1.15E-04	-9.14E-05	1.17E-03
8.00E-06	-8.45E-04	-2.56E-04	5.70E-04	-4.85E-04	7.02E-05
8.03E-06	-7.99E-04	-6.51E-04	5.72E-04	-2.03E-04	6.03E-04
8.06E-06	-2.83E-04	-7.09E-04	4.48E-04	-3.95E-04	3.22E-04
8.10E-06	-7.78E-04	-5.35E-04	5.59E-04	1.09E-04	8.39E-04
8.13E-06	-3.39E-04	-3.25E-04	1.62E-04	-4.83E-04	5.82E-04
8.16E-06	-7.77E-04	2.70E-04	4.46E-04	-4.23E-04	5.89E-04
8.19E-06	-4.80E-04	-6.85E-04	3.26E-04	-1.02E-04	1.13E-04
8.22E-06	-4.55E-04	-4.50E-04	6.59E-04	-5.95E-04	3.98E-04
8.26E-06	-7.69E-04	-4.47E-04	5.48E-04	-2.71E-04	2.94E-05
8.29E-06	-3.81E-04	-1.79E-04	1.06E-04	-3.71E-05	8.75E-04
8.32E-06	-5.85E-04	-1.43E-04	3.00E-04	2.50E-04	8.56E-04
8.35E-06	-6.50E-05	-1.25E-04	4.80E-04	-3.88E-04	3.85E-05
8.38E-06	-4.50E-04	-5.96E-05	5.76E-04	9.45E-05	8.03E-04
8.42E-06	-3.42E-04	-7.56E-04	1.31E-04	-4.12E-04	9.49E-04
8.45E-06	-1.13E-04	-3.71E-04	1.37E-03	-5.47E-04	9.06E-04
8.48E-06	-2.94E-04	-1.68E-04	2.55E-04	2.49E-04	7.91E-04
8.51E-06	-4.40E-04	-7.92E-04	9.72E-05	-2.18E-04	1.02E-03
8.54E-06	-1.40E-04	-3.12E-04	5.44E-04	-2.67E-04	8.54E-04
8.58E-06	-2.43E-04	-3.23E-04	8.59E-04	-2.38E-04	4.40E-04
8.61E-06	-4.78E-04	-1.94E-04	4.27E-04	-2.38E-04	4.94E-04
8.64E-06	-7.17E-04	-1.58E-04	5.97E-04	-8.41E-05	2.48E-04
8.67E-06	-2.41E-04	-5.78E-05	4.70E-04	-2.60E-04	7.47E-04
8.70E-06	-2.65E-04	-7.30E-05	1.77E-04	1.81E-06	9.59E-04
8.74E-06	-7.38E-04	9.10E-06	8.97E-05	5.67E-05	3.10E-04
8.77E-06	1.55E-04	7.11E-05	1.88E-04	1.36E-04	4.46E-04
8.80E-06	-4.30E-04	-2.90E-04	6.72E-04	-2.43E-04	4.16E-04
8.83E-06	-3.03E-04	-2.31E-04	6.66E-04	-4.26E-04	1.64E-04
8.86E-06	-2.65E-04	-3.41E-04	2.12E-04	-1.71E-04	8.60E-04
8.90E-06	-1.09E-04	2.40E-05	6.93E-04	-1.43E-04	4.30E-04
8.93E-06	-2.54E-04	-8.85E-04	-5.96E-07	-2.16E-04	7.10E-04
8.96E-06	-1.64E-04	-6.57E-04	6.87E-04	-1.67E-04	4.79E-04
8.99E-06	-7.26E-04	1.83E-05	2.60E-04	2.51E-04	3.51E-04
9.02E-06	-1.83E-04	6.16E-04	8.63E-04	2.12E-05	3.71E-04
9.06E-06	-3.00E-04	-3.77E-04	5.02E-04	-2.85E-04	8.10E-04
9.09E-06	-4.82E-04	-1.02E-04	1.50E-05	2.34E-04	5.38E-04
9.12E-06	7.08E-05	2.63E-04	5.08E-04	6.96E-04	5.71E-04
9.15E-06	3.70E-05	-6.02E-04	8.18E-04	1.30E-04	8.42E-04
9.18E-06	-7.03E-04	3.53E-05	8.11E-05	-1.11E-04	5.83E-04

9.22E-06	2.68E-04	-1.04E-03	9.71E-04	3.46E-04	4.15E-04
9.25E-06	-1.62E-05	-1.06E-03	3.42E-04	1.23E-04	3.38E-04
9.28E-06	-1.17E-04	-4.31E-04	7.07E-04	-5.31E-05	4.35E-04
9.31E-06	3.07E-04	1.28E-04	-1.04E-04	3.36E-04	-2.68E-04
9.34E-06	-4.64E-04	-2.51E-04	1.81E-04	4.28E-04	-5.90E-05
9.38E-06	-5.70E-05	-8.12E-04	4.51E-04	-1.44E-04	5.51E-04
9.41E-06	-4.99E-04	4.26E-04	1.61E-04	-1.99E-04	2.16E-04
9.44E-06	-2.47E-04	-6.37E-04	6.39E-04	1.77E-04	6.45E-04
9.47E-06	5.98E-05	2.45E-04	7.26E-05	-6.01E-05	2.82E-04
9.50E-06	-1.51E-04	-4.59E-04	2.42E-04	-3.99E-04	2.38E-04
9.54E-06	-2.81E-04	4.06E-05	3.43E-04	-3.61E-04	8.91E-04
9.57E-06	3.18E-05	-3.50E-04	7.86E-04	-9.07E-06	4.05E-04
9.60E-06	-3.62E-04	1.64E-04	7.19E-04	3.25E-05	3.70E-04
9.63E-06	-5.53E-04	3.03E-04	3.15E-04	-1.26E-05	6.28E-04
9.66E-06	-1.69E-05	6.95E-04	8.37E-04	3.93E-04	6.38E-04
9.70E-06	-1.02E-04	4.99E-04	6.97E-04	1.47E-04	5.03E-04
9.73E-06	6.85E-07	-4.96E-04	4.52E-04	1.43E-04	-1.07E-04
9.76E-06	4.04E-05	-3.54E-04	5.13E-04	-1.57E-04	-3.55E-04
9.79E-06	5.18E-05	-9.11E-05	5.69E-04	-2.21E-04	2.24E-04
9.82E-06	-1.89E-04	2.39E-04	-1.14E-04	9.03E-06	6.39E-04
9.86E-06	-2.15E-04	-4.09E-04	4.38E-04	1.90E-04	2.81E-04
9.89E-06	-5.59E-04	-2.76E-04	4.51E-04	2.75E-04	4.85E-04
9.92E-06	-3.01E-04	-5.24E-04	3.83E-04	-2.62E-04	1.08E-04
9.95E-06	-1.70E-05	-6.92E-04	1.01E-03	-3.42E-04	3.21E-04
9.98E-06	-2.27E-04	-5.16E-08	3.76E-04	-7.09E-04	-5.54E-05
1.00E-05	-3.62E-04	1.29E-04	4.85E-04	6.28E-05	-8.26E-05
1.01E-05	-2.90E-04	5.55E-05	5.59E-04	9.87E-05	7.67E-07
1.01E-05	-6.28E-04	-6.68E-04	8.34E-04	1.16E-04	5.61E-04
1.01E-05	-5.12E-04	-4.03E-04	3.93E-04	1.21E-05	5.38E-04
1.01E-05	-2.69E-04	-3.66E-04	7.33E-04	5.90E-04	-1.83E-04
1.02E-05	-1.90E-04	-7.10E-05	7.27E-04	8.87E-05	2.76E-04
1.02E-05	-1.08E-04	-4.70E-04	4.96E-04	-1.72E-04	3.54E-04
1.02E-05	-3.38E-04	-4.33E-04	5.75E-04	2.15E-04	4.09E-04
1.03E-05	1.99E-04	-1.44E-04	6.19E-04	2.19E-04	1.60E-04
1.03E-05	-2.10E-04	-2.54E-04	6.04E-04	5.07E-04	1.89E-04
1.03E-05	-4.22E-05	-6.68E-04	5.71E-04	5.25E-05	-2.75E-04
1.04E-05	-2.21E-04	-2.49E-04	8.33E-04	1.87E-04	1.62E-04
1.04E-05	-3.44E-04	-1.09E-04	7.95E-04	-1.35E-04	4.16E-04
1.04E-05	2.45E-04	-5.35E-04	9.38E-04	-5.19E-07	2.49E-04
1.05E-05	5.67E-05	-1.54E-04	2.29E-04	-2.71E-04	-8.46E-05
1.05E-05	6.04E-04	-3.03E-04	6.75E-04	1.10E-04	2.13E-04
1.05E-05	-1.46E-04	-7.34E-04	6.30E-04	-3.31E-04	4.44E-04
1.06E-05	4.93E-04	-2.94E-04	6.39E-04	-5.50E-05	1.04E-04
1.06E-05	2.81E-04	-3.01E-04	9.58E-04	6.17E-06	-2.96E-04
1.06E-05	9.55E-05	-6.87E-04	5.29E-04	3.71E-04	-1.46E-05
1.07E-05	4.28E-05	-8.01E-04	6.00E-04	1.51E-04	1.72E-04
1.07E-05	-4.51E-04	-1.87E-04	8.67E-04	6.76E-04	6.07E-04
1.07E-05	-4.27E-04	-7.85E-05	8.86E-04	4.66E-04	1.08E-04
1.08E-05	2.61E-04	9.14E-05	6.74E-04	-1.59E-04	2.31E-04
1.08E-05	-2.44E-05	-7.28E-04	1.07E-03	6.55E-04	4.50E-04
1.08E-05	-3.31E-04	-1.01E-04	8.61E-04	4.56E-04	3.11E-04
1.09E-05	7.78E-05	2.55E-04	9.81E-04	5.48E-04	1.01E-04
1.09E-05	6.20E-04	-5.41E-04	4.94E-04	4.28E-04	2.83E-05
1.09E-05	5.30E-04	-1.82E-04	1.03E-04	5.91E-05	-9.49E-05

1.09E-05	2.02E-04	-6.86E-04	1.21E-03	2.79E-04	2.38E-04
1.10E-05	2.79E-04	-1.75E-04	8.53E-04	-1.88E-04	2.90E-04
1.10E-05	4.01E-04	-8.09E-05	2.19E-04	6.32E-04	-4.58E-07
1.10E-05	2.39E-04	-4.45E-04	9.20E-04	1.50E-04	4.44E-04
1.11E-05	8.37E-05	-2.72E-04	7.28E-04	1.71E-04	2.98E-04
1.11E-05	2.52E-04	-4.53E-04	7.45E-04	-8.76E-06	1.14E-04
1.11E-05	9.88E-05	-7.84E-04	7.57E-04	2.16E-04	2.43E-04
1.12E-05	4.80E-04	-4.83E-04	2.00E-04	3.44E-04	4.28E-04
1.12E-05	4.37E-04	-6.44E-04	1.08E-03	-6.03E-04	1.40E-04
1.12E-05	1.44E-04	-5.41E-05	6.84E-04	-3.03E-04	5.25E-04
1.13E-05	3.06E-04	-6.72E-04	5.70E-05	4.05E-04	2.33E-04
1.13E-05	4.14E-04	-1.89E-04	6.56E-04	4.13E-04	-1.67E-05
1.13E-05	2.91E-04	-2.11E-04	3.30E-04	2.27E-04	1.30E-04
1.14E-05	-3.37E-04	-8.19E-04	7.69E-04	4.25E-04	2.99E-04
1.14E-05	-1.87E-04	-6.29E-04	6.32E-04	-7.42E-06	3.43E-04
1.14E-05	5.98E-04	-3.24E-04	7.98E-04	5.07E-04	5.13E-04
1.15E-05	-9.61E-05	-5.06E-04	2.81E-04	7.12E-04	-4.86E-05
1.15E-05	9.58E-05	-1.35E-04	7.58E-04	2.65E-04	5.90E-04
1.15E-05	3.72E-04	-3.09E-04	3.48E-04	7.15E-04	2.28E-05
1.16E-05	2.46E-04	-7.88E-04	1.12E-04	1.70E-04	1.37E-04
1.16E-05	-2.60E-04	-6.97E-04	8.41E-04	4.51E-05	4.07E-04
1.16E-05	1.12E-04	-6.08E-05	3.76E-04	2.27E-04	3.63E-04
1.17E-05	-7.53E-05	-3.43E-04	8.24E-04	-8.48E-05	5.39E-04
1.17E-05	-2.83E-05	-4.45E-04	4.38E-04	5.97E-05	7.61E-04
1.17E-05	4.90E-05	-4.15E-04	6.98E-04	2.38E-04	2.72E-04
1.17E-05	4.01E-04	-4.09E-04	6.46E-04	1.77E-04	3.02E-04
1.18E-05	3.35E-04	-4.90E-04	1.14E-03	2.01E-04	3.05E-04
1.18E-05	7.43E-05	-2.90E-04	5.64E-04	2.08E-04	4.93E-04
1.18E-05	3.39E-04	-1.83E-04	1.15E-03	1.04E-04	-1.04E-04
1.19E-05	-1.49E-04	-6.53E-04	5.83E-04	9.92E-05	-3.13E-05
1.19E-05	-1.59E-04	-1.96E-04	5.71E-04	1.36E-04	3.60E-04
1.19E-05	3.66E-04	2.67E-05	1.10E-03	6.47E-04	2.92E-04
1.20E-05	-1.86E-05	-2.19E-04	5.85E-04	4.63E-04	3.24E-04
1.20E-05	2.96E-04	-6.35E-04	9.68E-04	4.39E-04	3.93E-04
1.20E-05	5.73E-04	-1.81E-04	8.57E-04	3.97E-04	1.75E-04
1.21E-05	3.36E-04	-3.94E-04	1.41E-04	-4.92E-05	-6.09E-06
1.21E-05	-4.22E-04	-5.70E-04	2.77E-04	7.38E-04	1.34E-04
1.21E-05	9.99E-05	-8.40E-04	6.63E-04	3.31E-04	-2.38E-04
1.22E-05	-2.98E-04	-2.30E-04	2.52E-04	5.32E-04	-1.75E-04
1.22E-05	2.65E-04	1.48E-05	1.35E-04	5.90E-04	-4.26E-07
1.22E-05	3.08E-04	3.00E-06	1.16E-03	4.43E-04	-1.80E-04
1.23E-05	3.95E-04	-5.17E-04	1.38E-03	8.29E-05	4.34E-04
1.23E-05	2.27E-04	-6.10E-04	8.40E-04	1.49E-04	2.75E-04
1.23E-05	2.03E-04	-5.01E-04	6.78E-04	2.51E-04	4.04E-04
1.24E-05	2.55E-04	-3.40E-04	3.72E-04	-2.29E-04	5.49E-04
1.24E-05	2.80E-04	-6.75E-04	6.99E-04	7.86E-05	4.40E-04
1.24E-05	7.31E-04	-4.17E-04	6.70E-04	4.96E-04	1.89E-04
1.25E-05	5.96E-04	-7.82E-04	6.65E-04	3.44E-04	5.59E-04
1.25E-05	2.32E-04	-8.17E-04	3.89E-04	1.63E-04	6.10E-04
1.25E-05	2.78E-04	-2.74E-04	5.84E-04	5.84E-05	2.14E-05
1.25E-05	-7.09E-05	-2.73E-04	1.17E-04	3.63E-04	4.36E-04
1.26E-05	1.83E-04	-9.03E-05	6.93E-04	-7.21E-05	2.67E-04
1.26E-05	1.60E-04	-2.95E-04	1.39E-04	2.44E-04	5.49E-05
1.26E-05	3.91E-04	-2.03E-04	8.75E-04	1.62E-04	-2.68E-04

1.27E-05	3.36E-04	2.46E-05	6.42E-04	6.73E-04	-4.72E-05
1.27E-05	-4.31E-04	-2.65E-04	7.20E-04	1.74E-04	3.44E-04
1.27E-05	-6.95E-04	-1.69E-04	7.99E-04	7.42E-04	4.30E-04
1.28E-05	1.06E-06	-4.84E-04	7.00E-04	4.05E-04	3.21E-04
1.28E-05	4.18E-04	-7.70E-04	3.57E-04	2.92E-04	2.70E-04
1.28E-05	-9.96E-05	-4.73E-04	4.01E-04	2.50E-04	3.77E-05
1.29E-05	-3.23E-04	-3.37E-04	3.42E-04	-2.46E-04	6.44E-04
1.29E-05	1.12E-04	4.19E-05	5.62E-04	1.81E-04	4.90E-04
1.29E-05	-2.99E-04	1.75E-04	1.27E-04	5.68E-04	-5.71E-04
1.30E-05	3.06E-04	-2.75E-04	1.71E-04	3.61E-04	2.79E-04
1.30E-05	-2.87E-05	-1.22E-04	1.03E-03	1.46E-04	2.27E-05
1.30E-05	-5.57E-05	-1.95E-04	7.38E-04	3.09E-04	-5.45E-05
1.31E-05	2.37E-05	-4.04E-04	4.09E-04	-2.80E-04	2.71E-04
1.31E-05	1.82E-04	1.85E-04	6.41E-04	4.59E-04	2.63E-04
1.31E-05	-8.20E-05	3.83E-06	7.71E-04	3.97E-05	2.79E-04
1.32E-05	5.66E-06	-1.92E-05	1.67E-04	3.68E-05	1.77E-04
1.32E-05	3.06E-04	-2.10E-04	8.98E-04	-8.57E-04	2.34E-04
1.32E-05	-1.64E-04	-2.25E-04	2.15E-04	1.24E-04	4.00E-04
1.33E-05	-6.85E-05	1.37E-04	3.80E-04	-5.55E-05	1.44E-04
1.33E-05	-2.28E-04	-3.01E-04	3.55E-04	3.13E-04	4.74E-04
1.33E-05	3.67E-04	-6.14E-04	8.37E-04	3.75E-04	5.66E-04
1.33E-05	3.46E-04	-2.08E-04	5.71E-04	5.20E-05	5.64E-04
1.34E-05	5.48E-04	-3.39E-04	6.10E-04	5.91E-04	2.00E-04
1.34E-05	-1.84E-04	-2.80E-04	2.94E-04	-1.78E-04	6.47E-04
1.34E-05	-1.19E-04	-4.40E-04	6.93E-04	6.25E-04	4.38E-04
1.35E-05	-1.88E-04	-2.38E-05	3.97E-04	3.46E-04	3.25E-04
1.35E-05	3.78E-04	-6.21E-04	9.72E-04	-1.95E-05	6.17E-04
1.35E-05	2.83E-04	-5.41E-04	8.85E-04	3.30E-04	2.09E-04
1.36E-05	-2.55E-04	-3.78E-04	6.30E-04	9.13E-04	3.21E-04
1.36E-05	-4.55E-04	-7.57E-04	6.52E-04	2.63E-04	6.93E-04
1.36E-05	-4.29E-04	-4.91E-04	-7.00E-05	2.49E-04	1.35E-04
1.37E-05	-2.15E-04	-2.33E-04	1.38E-04	5.87E-04	2.93E-04
1.37E-05	2.66E-04	-1.81E-04	9.55E-04	5.42E-04	4.33E-04
1.37E-05	3.74E-04	-4.98E-04	3.17E-04	-8.92E-05	2.78E-04
1.38E-05	4.13E-04	-6.70E-04	5.00E-04	2.05E-04	9.49E-05
1.38E-05	8.20E-05	2.43E-04	3.71E-04	3.05E-04	2.84E-04
1.38E-05	-9.78E-05	-1.60E-04	5.95E-04	3.50E-04	8.67E-04
1.39E-05	2.18E-04	-7.50E-04	6.30E-04	-1.56E-04	4.28E-04
1.39E-05	2.07E-04	-8.25E-04	5.15E-04	3.50E-04	2.60E-04
1.39E-05	1.92E-04	-2.76E-04	4.15E-05	1.38E-04	2.17E-04
1.40E-05	-2.90E-04	-2.49E-04	5.29E-04	9.26E-05	7.98E-04
1.40E-05	1.57E-04	-5.55E-04	2.11E-04	-2.23E-04	3.09E-04
1.40E-05	3.50E-04	-3.68E-04	6.08E-04	6.01E-05	4.42E-04
1.41E-05	-3.04E-04	-3.19E-04	6.56E-04	5.92E-04	4.65E-04
1.41E-05	-3.91E-04	-4.23E-04	6.73E-04	3.63E-04	7.81E-04
1.41E-05	-1.85E-04	-4.09E-04	4.00E-04	1.52E-04	5.96E-04
1.41E-05	-5.40E-04	-5.83E-04	1.13E-04	-4.42E-04	7.21E-04
1.42E-05	1.34E-04	4.95E-05	1.03E-03	-9.13E-04	3.32E-04
1.42E-05	-5.79E-05	-5.72E-05	6.37E-05	3.17E-04	4.38E-04
1.42E-05	-1.29E-04	-1.62E-04	3.76E-04	-2.06E-04	7.00E-04
1.43E-05	2.00E-04	-3.24E-04	5.59E-04	-9.32E-05	5.01E-04
1.43E-05	-6.85E-04	-1.97E-04	7.71E-04	1.80E-04	5.02E-04
1.43E-05	-1.76E-04	-7.24E-05	1.97E-04	8.74E-04	8.16E-04
1.44E-05	2.15E-04	-1.38E-04	2.90E-04	-1.11E-05	1.02E-04

1.44E-05	2.19E-04	-1.54E-04	3.78E-05	5.60E-04	6.18E-04
1.44E-05	-3.60E-04	-1.81E-04	3.93E-04	-1.67E-04	3.31E-04
1.45E-05	-4.97E-04	-3.86E-05	3.24E-04	-8.23E-05	9.35E-05
1.45E-05	3.16E-05	-3.17E-05	-1.10E-04	-7.09E-04	3.58E-04
1.45E-05	1.53E-04	-1.92E-04	6.44E-05	1.17E-04	5.34E-04
1.46E-05	-2.40E-05	-8.01E-04	4.70E-04	6.81E-04	4.44E-04
1.46E-05	-4.67E-04	-4.01E-04	3.65E-04	3.38E-04	5.60E-04
1.46E-05	1.51E-05	-5.88E-04	6.32E-04	-4.46E-04	3.83E-04
1.47E-05	7.59E-04	-2.57E-04	6.58E-04	-4.72E-05	1.23E-04
1.47E-05	1.38E-05	-2.32E-04	2.09E-04	3.94E-04	2.36E-04
1.47E-05	-5.45E-04	-2.34E-04	7.87E-04	-3.96E-05	3.19E-04
1.48E-05	3.53E-04	-1.25E-04	6.58E-05	7.48E-04	1.03E-04
1.48E-05	1.55E-04	-1.09E-04	5.26E-05	4.24E-04	1.23E-03
1.48E-05	-1.56E-04	-3.47E-04	1.05E-04	2.17E-04	6.29E-04
1.49E-05	-2.72E-04	-4.84E-04	3.26E-04	6.91E-04	4.71E-04
1.49E-05	2.70E-05	-8.14E-04	1.30E-04	4.86E-04	9.99E-04
1.49E-05	-7.91E-05	-1.09E-04	3.31E-04	1.50E-05	6.03E-04
1.49E-05	-5.27E-05	-5.66E-04	2.54E-04	-1.35E-04	4.02E-04
1.50E-05	4.92E-04	-2.59E-04	2.22E-04	-1.41E-04	1.65E-04

Table SI.15A: Primary kinetic data for the reaction of 1,3-dimethyl-2-imidazolidinone (**S15**) with CumO• generated by 355 nm LFP in MeCN (**Figure SI.15A**).

t / s	ΔA 1,3-dimethyl-2-imidazolidinone (S15)				
	**5.83mM**	**14.8mM**	**26.1mM**	**36.7mM**	**49.1mM**
-1E-06	-0.0007	0.00015	0.00018	-0.0009	1.2E-05
-1E-06	-0.0012	-0.0001	-0.0008	-0.0014	-5E-05
-9E-07	-0.0018	0.00057	0.00043	-0.0022	0.00012
-9E-07	-0.0019	0.00028	-0.0008	-0.0019	0.00035
-9E-07	-0.0014	0.00062	-0.0002	-0.0021	0.00036
-8E-07	-0.0014	0.00088	-0.0007	-0.0016	0.00046
-8E-07	-0.0011	0.00035	-0.0008	-0.0021	0.00035
-8E-07	-0.0015	5.8E-05	-0.0006	-0.0021	-3E-05
-7E-07	-0.0013	-1E-04	0.00021	-0.0014	0.0008
-7E-07	-0.0013	-0.0005	-0.0002	-0.0015	0.00067
-7E-07	-0.0017	1.2E-06	-0.0007	-0.0011	0.00059
-6E-07	-0.0008	0.00078	-0.0009	-0.0009	0.00011
-6E-07	-0.0013	0.00063	0.00016	-0.0012	0.00035
-6E-07	-0.0016	0.00075	-0.0002	-0.0026	0.00038
-5E-07	-0.0014	0.00076	-0.0004	-0.0023	0.0004
-5E-07	-0.0012	0.00032	-0.0003	-0.002	0.00044
-5E-07	-0.0018	0.00013	-0.0002	-0.0015	0.0006
-4E-07	-0.0018	-6E-05	-0.0011	-0.0011	-0.0008
-4E-07	-0.0012	0.00031	-9E-05	-0.0013	-0.0002
-4E-07	-0.0014	0.00095	-0.0006	-0.0016	-9E-05
-4E-07	-0.0007	0.00101	-0.0005	-0.0018	-9E-05
-3E-07	-0.0013	0.0011	-0.0009	-0.0018	7.5E-05
-3E-07	-0.0014	-0.0012	-0.0007	-0.0015	0.00022
-3E-07	-0.0012	-0.0002	-0.0009	-0.0013	-0.0008
-2E-07	-0.0017	-7E-05	-0.0012	-0.0008	-0.0012
-2E-07	-0.001	0.00089	-0.0005	-0.0007	-0.0004
-2E-07	-0.0013	4.6E-05	-0.001	-0.001	-0.0003
-1E-07	-0.0016	0.00055	-6E-05	-0.0013	2.7E-05
-1E-07	-0.0008	0.00055	-0.0002	-0.0008	0.00047
-6E-08	-0.0008	0.00086	-0.0006	-0.0022	-0.0005
-3E-08	-0.0011	-0.0001	7.9E-05	-0.0012	-5E-05
-2E-10	-0.0045	-0.0031	-0.0043	-0.0045	-0.0038
3.2E-08	-0.0025	-0.0015	-0.0036	-0.0052	-0.0054
6.4E-08	0.0274	0.02595	0.02596	0.02156	0.02167
9.6E-08	0.0281	0.02705	0.02581	0.02119	0.0208
1.3E-07	0.02705	0.02616	0.02481	0.01945	0.01956
1.6E-07	0.02616	0.02358	0.02223	0.01822	0.0181
1.9E-07	0.0244	0.02359	0.0215	0.01698	0.01719
2.2E-07	0.02422	0.02256	0.02116	0.01576	0.01531
2.6E-07	0.02276	0.02164	0.01963	0.01449	0.01499
2.9E-07	0.02209	0.02151	0.01878	0.01405	0.01398
3.2E-07	0.02207	0.02003	0.01768	0.01241	0.01268
3.5E-07	0.02128	0.01962	0.01591	0.01159	0.0115
3.8E-07	0.01953	0.01804	0.01615	0.01045	0.01145
4.2E-07	0.0193	0.01725	0.01446	0.01021	0.01068
4.5E-07	0.01835	0.01671	0.01391	0.00998	0.01031
4.8E-07	0.01855	0.01669	0.01269	0.00847	0.00896
5.1E-07	0.01753	0.01607	0.01287	0.00867	0.0074
5.4E-07	0.01697	0.01553	0.01137	0.00786	0.00749

5.8E-07	0.01553	0.01431	0.01109	0.00678	0.00741
6.1E-07	0.01532	0.0139	0.01056	0.00637	0.00672
6.4E-07	0.01448	0.01339	0.01016	0.00653	0.00652
6.7E-07	0.01475	0.01266	0.00944	0.00574	0.00586
7E-07	0.01399	0.01249	0.00844	0.00563	0.00539
7.4E-07	0.01424	0.01199	0.00832	0.00478	0.00463
7.7E-07	0.01331	0.01176	0.00836	0.00492	0.00508
8E-07	0.01274	0.01096	0.00736	0.004	0.00501
8.3E-07	0.01308	0.00984	0.00705	0.00305	0.00441
8.6E-07	0.01272	0.01025	0.00738	0.0037	0.00378
9E-07	0.01117	0.01015	0.00657	0.00314	0.00378
9.3E-07	0.01168	0.01005	0.00624	0.00262	0.00327
9.6E-07	0.01055	0.00944	0.00647	0.00314	0.00366
9.9E-07	0.01035	0.00861	0.00605	0.00276	0.00369
1E-06	0.01051	0.00825	0.0051	0.00277	0.00344
1.1E-06	0.00988	0.00877	0.00512	0.00283	0.00329
1.1E-06	0.00987	0.00769	0.0053	0.0021	0.0027
1.1E-06	0.00912	0.00778	0.00466	0.00178	0.00277
1.2E-06	0.00906	0.00741	0.00442	0.00155	0.00265
1.2E-06	0.00902	0.00756	0.00314	0.00151	0.00211
1.2E-06	0.00853	0.00571	0.00375	0.00128	0.00244
1.2E-06	0.0085	0.00647	0.0035	0.00184	0.00311
1.3E-06	0.00783	0.00586	0.00304	0.00067	0.00235
1.3E-06	0.00809	0.00654	0.00353	0.00062	0.00172
1.3E-06	0.00726	0.00576	0.00326	0.00069	0.00251
1.4E-06	0.00696	0.00605	0.00366	0.00095	0.00202
1.4E-06	0.00687	0.00546	0.00269	-6E-05	0.00131
1.4E-06	0.00679	0.00542	0.00246	0.00086	0.00182
1.5E-06	0.00675	0.00489	0.00259	0.0008	0.0022
1.5E-06	0.00722	0.00556	0.00278	0.00069	0.00225
1.5E-06	0.00682	0.00476	0.00289	0.00113	0.00209
1.6E-06	0.00574	0.00526	0.00272	0.00093	0.00098
1.6E-06	0.00614	0.00433	0.00223	0.00028	0.00147
1.6E-06	0.00584	0.00477	0.00161	-1E-04	0.00226
1.7E-06	0.00541	0.00447	0.00198	-0.0004	0.00218
1.7E-06	0.00546	0.00455	0.0015	0.00042	0.0009
1.7E-06	0.00466	0.00393	0.002	-0.0001	0.00167
1.8E-06	0.00533	0.00381	0.00113	-0.0002	0.00155
1.8E-06	0.00458	0.00439	0.00117	-9E-05	0.00148
1.8E-06	0.00476	0.00442	0.00183	-0.001	0.00099
1.9E-06	0.00493	0.00359	0.00076	0.00034	0.00074
1.9E-06	0.00505	0.00376	0.00172	-0.0003	0.00101
1.9E-06	0.00385	0.00421	0.001	6.2E-05	0.00082
2E-06	0.00448	0.00358	0.00172	-0.001	0.0006
2E-06	0.00418	0.0034	0.00204	-0.001	0.00048
2E-06	0.00494	0.00401	0.00227	-0.0005	0.00044
2E-06	0.00326	0.00292	0.00109	-6E-05	0.00117
2.1E-06	0.00387	0.00383	0.00158	-0.0007	0.00165
2.1E-06	0.00394	0.00272	0.00152	-0.0006	0.00101
2.1E-06	0.00338	0.00298	0.00055	-0.0004	0.00075
2.2E-06	0.00329	0.00295	0.00085	-0.0003	0.00089
2.2E-06	0.00335	0.00312	0.00087	-0.001	0.00108
2.2E-06	0.00323	0.00284	0.00054	-0.0005	0.00136
2.3E-06	0.00283	0.00269	0.00057	-0.0008	0.00129

2.3E-06	0.00194	0.003	0.00032	-0.0012	0.00125
2.3E-06	0.00241	0.00247	0.00102	-0.0008	0.00106
2.4E-06	0.00294	0.00171	0.00098	-0.0004	0.00156
2.4E-06	0.00286	0.00259	0.00029	-0.0005	0.00086
2.4E-06	0.00223	0.00239	0.00064	-0.0016	0.00115
2.5E-06	0.00166	0.00225	0.00081	-0.0013	0.00085
2.5E-06	0.00144	0.00212	0.00109	-0.0006	0.00095
2.5E-06	0.00292	0.00163	0.00084	-0.0006	0.00087
2.6E-06	0.00168	0.00294	0.001	0.00027	0.00082
2.6E-06	0.00196	0.00197	0.00089	-0.0016	0.00142
2.6E-06	0.00221	0.00181	0.00098	-0.0005	0.00076
2.7E-06	0.00204	0.00158	0.00048	-0.0012	0.00158
2.7E-06	0.00199	0.00216	0.00046	-0.0015	0.00133
2.7E-06	0.00189	0.00194	0.00055	-0.0017	0.00108
2.8E-06	0.00168	0.00151	0.00049	-0.001	0.00132
2.8E-06	0.00173	0.00201	0.00043	-0.0004	0.00096
2.8E-06	0.00174	0.00185	0.00064	-0.0006	0.00123
2.8E-06	0.00156	0.00145	-3E-06	-0.0006	0.00137
2.9E-06	0.00134	0.00181	0.00053	-0.0009	0.00178
2.9E-06	0.00185	0.00161	5.4E-05	-0.0007	0.00108
2.9E-06	0.00181	0.00172	1.9E-05	-0.0012	0.00214
3E-06	0.00095	0.00226	0.00017	-0.0003	0.00088
3E-06	0.0011	0.0018	0.00062	-0.001	0.00099
3E-06	0.00073	0.00183	0.00026	-0.0008	0.00131
3.1E-06	0.0006	0.00206	0.00036	-0.001	0.00131
3.1E-06	0.001	0.00116	8.3E-05	-0.0012	0.00116
3.1E-06	0.00101	0.00151	0.00014	-0.0008	0.00066
3.2E-06	0.00125	0.00113	-0.0007	-0.0013	0.00069
3.2E-06	0.00138	0.00144	-0.0002	-0.0011	0.00092
3.2E-06	0.00011	0.00119	0.00014	-0.0015	0.0006
3.3E-06	0.00062	0.00225	0.00024	-0.0012	0.00084
3.3E-06	0.00238	0.00153	0.00051	-0.0009	0.00046
3.3E-06	0.00099	0.00157	1.4E-05	-0.0013	0.00132
3.4E-06	0.00039	0.00157	0.00033	-0.0011	0.00137
3.4E-06	0.00123	0.0019	0.00043	-0.0017	0.00141
3.4E-06	0.00079	0.00156	0.0004	-0.0006	0.00112
3.5E-06	0.0008	0.00128	0.0006	-0.0009	0.00066
3.5E-06	0.00066	0.00147	9.2E-06	-0.0014	0.00076
3.5E-06	0.00034	0.00167	3.3E-05	-0.0006	0.00113
3.6E-06	0.00126	0.00177	0.00075	-0.0007	0.00084
3.6E-06	0.0006	0.0019	0.00045	-0.0006	0.00062
3.6E-06	0.00037	0.00143	-7E-05	-0.0008	0.00167
3.6E-06	8.8E-05	0.00108	0.00013	-0.0017	0.00089
3.7E-06	0.00018	0.00116	5.3E-05	-0.0015	0.00055
3.7E-06	0.00088	0.00114	0.0001	-0.0012	0.00079
3.7E-06	0.00016	0.00095	5.8E-05	-0.0013	0.00119
3.8E-06	0.00087	0.00124	-0.0002	-0.0019	0.0008
3.8E-06	-0.0002	0.00093	-0.0001	-0.0012	0.00136
3.8E-06	-0.0002	0.00119	-0.0003	-0.0014	0.00093
3.9E-06	0.00051	0.00128	-3E-05	-0.001	0.00056
3.9E-06	-0.0003	0.00102	-0.0003	-0.0013	0.00132
3.9E-06	-9E-05	0.00091	0.00023	-0.0015	0.00049
4E-06	3.8E-05	0.00027	0.00048	-0.0016	0.00118
4E-06	-0.0004	0.00112	2.8E-05	-0.0011	0.0007

4E-06	-0.0003	0.00167	6.9E-05	-0.0012	0.00102
4.1E-06	-0.0007	0.0012	-0.0001	-0.0011	0.00128
4.1E-06	-0.0001	0.0018	0.00036	-0.0012	0.0011
4.1E-06	9.1E-05	0.0008	-0.0003	-0.0013	0.00099
4.2E-06	-0.0003	0.00135	-0.0005	-0.0016	0.00105
4.2E-06	-8E-05	0.00156	-0.0003	-0.0009	0.00052
4.2E-06	0.00078	0.00067	-0.0007	-0.0014	0.00074
4.3E-06	-5E-05	0.00095	0.00021	-0.0016	0.00121
4.3E-06	-0.0003	0.00077	0.00018	-0.0008	0.00055
4.3E-06	-0.0008	0.001	9.5E-05	-0.0012	0.00089
4.4E-06	-0.0004	0.00103	0.00056	-0.0009	0.00111
4.4E-06	-8E-05	0.00155	7.5E-06	-0.0012	0.00137
4.4E-06	-0.0002	0.00094	3.6E-05	-0.0014	0.00134
4.4E-06	-1E-04	0.001	-5E-06	-0.0003	0.00095
4.5E-06	-7E-06	0.00049	0.00013	-0.0007	0.00057
4.5E-06	-3E-05	0.00089	-0.0003	-0.0004	0.00172
4.5E-06	-3E-05	0.00097	0.00039	-0.0021	0.00154
4.6E-06	-3E-05	0.00115	-0.0004	-0.0012	0.00125
4.6E-06	-0.0001	0.00178	-0.0003	-0.0014	0.00045
4.6E-06	0.00022	0.00161	-0.0002	-0.0013	0.00126
4.7E-06	-3E-05	0.00129	4.1E-05	-0.0014	0.00042
4.7E-06	-0.0006	0.00173	-0.0004	-0.0012	0.00106
4.7E-06	-0.0008	0.00125	-0.0004	-0.0003	0.0009
4.8E-06	-5E-05	0.00141	-0.0006	-0.0002	0.00046
4.8E-06	0.00011	0.00148	0.00018	-0.0005	0.00112
4.8E-06	-0.0003	0.00054	-3E-05	-0.0006	0.00127
4.9E-06	-0.0006	0.00113	-0.0005	-0.0006	0.00157
4.9E-06	-0.0005	0.00068	-0.0007	-0.0006	0.00033
4.9E-06	-0.0012	0.00163	-0.0001	-0.0009	0.00026
5E-06	-0.0006	0.00093	8.2E-05	3.5E-05	0.001
5E-06	-0.0004	0.00061	4.5E-05	-0.0008	0.00077
5E-06	-0.0005	0.0013	-0.0001	-0.0005	0.00165
5.1E-06	-0.0003	0.00152	0.0002	-0.0006	0.00046
5.1E-06	-0.0006	0.00134	-0.0001	-0.0006	0.00056
5.1E-06	-0.0011	0.00074	-0.0002	-0.0011	0.00046
5.2E-06	-0.0008	0.00133	0.00013	-0.0009	0.00058
5.2E-06	-0.0012	0.0018	-0.0002	-0.0016	0.00054
5.2E-06	0.00013	0.00223	-0.0001	-0.0009	0.00044
5.2E-06	-0.0003	0.00109	0.00054	-0.0012	0.00053
5.3E-06	0.00013	0.00061	0.00037	-0.0016	0.00089
5.3E-06	-7E-05	0.00065	0.00041	-0.0011	0.0006
5.3E-06	-0.0004	0.00122	1.6E-05	-0.0018	0.00128
5.4E-06	-0.0012	-0.0001	-0.0001	-0.0012	0.00046
5.4E-06	-0.001	0.00101	-7E-05	-0.0015	0.00082
5.4E-06	-0.0005	0.00103	0.00013	-0.0008	0.00195
5.5E-06	-0.0009	0.00036	-0.0007	-0.0008	0.0015
5.5E-06	-0.0008	0.00097	-0.0003	-0.0015	0.00072
5.5E-06	-0.0009	0.00118	0.00054	-0.0005	0.00088
5.6E-06	-0.0005	0.00148	0.00027	-0.0008	0.00054
5.6E-06	-0.0004	0.00162	0.0007	-0.0009	0.00057
5.6E-06	-0.0007	0.00125	0.00011	-0.0018	0.00193
5.7E-06	0.00014	0.00035	-3E-05	-0.0011	0.00208
5.7E-06	-0.0003	0.00082	0.00056	-0.001	0.00046
5.7E-06	2E-05	0.00119	0.00023	-0.0012	0.00118

5.8E-06	-0.0004	0.00056	-0.0004	-0.0007	1E-04
5.8E-06	-0.0012	0.00083	0.00048	-0.001	0.00074
5.8E-06	-0.0008	0.0014	-0.0002	-0.0004	0.00148
5.9E-06	-0.0011	0.0012	-6E-05	-0.0005	0.00032
5.9E-06	-0.0004	0.00112	0.00058	-0.0007	0.00094
5.9E-06	-0.0011	0.00093	-0.0002	-0.0012	0.001
6E-06	-0.0007	0.00091	-0.0002	-0.0013	0.00069
6E-06	-0.0003	0.00131	-0.0003	-0.0006	0.00025
6E-06	-0.0009	0.00079	0.00039	-0.0009	0.00164
6E-06	-6E-06	0.00116	-0.0002	-0.0005	-0.0002
6.1E-06	-0.0005	0.00057	0.00013	-0.0016	0.00109
6.1E-06	-0.0004	0.0013	0.00039	-0.0016	0.00078
6.1E-06	-0.0006	0.00118	-2E-05	-0.001	0.0019
6.2E-06	-0.0008	0.00085	2.5E-05	-0.0015	0.00125
6.2E-06	-0.0004	0.00088	0.00048	-0.0012	0.00025
6.2E-06	-0.0007	0.00113	-0.0008	-0.0007	0.00103
6.3E-06	-0.0008	0.00042	-0.0003	-0.0011	0.00038
6.3E-06	-0.0002	0.00055	-0.0002	-0.001	0.00063
6.3E-06	-0.0009	0.00056	-6E-06	-0.0012	0.00162
6.4E-06	-0.0003	0.00032	-0.0001	-0.0008	0.0002
6.4E-06	-1E-04	0.0013	-0.0003	-0.0005	0.00028
6.4E-06	-0.0006	0.00086	-0.0001	-0.0009	0.00045
6.5E-06	-0.0001	0.00121	2.5E-06	-0.0005	0.00096
6.5E-06	-0.0003	0.00157	0.00045	-0.0001	0.00062
6.5E-06	-0.0004	0.00163	-0.0001	-0.0012	0.00101
6.6E-06	-0.0013	0.00063	2E-05	-0.0017	0.00235
6.6E-06	-0.0014	0.00071	0.00025	-0.0003	-0.0002
6.6E-06	-0.0011	0.00088	-0.0005	-0.0016	0.0008
6.7E-06	-0.0007	0.00084	-1E-04	-0.0009	0.00073
6.7E-06	-0.0005	0.00109	0.00053	-0.0013	0.00065
6.7E-06	-0.001	0.00078	-0.0004	-0.001	0.00178
6.8E-06	-0.0007	0.00093	-0.0005	-0.0013	0.00103
6.8E-06	-3E-05	0.00078	0.00035	-0.0011	0.00167
6.8E-06	-0.0004	0.00155	-0.0001	-0.0004	0.00067
6.8E-06	-0.0006	0.00143	-0.0001	-0.001	0.00061
6.9E-06	-0.0006	0.00119	5.5E-06	-0.0009	0.00067
6.9E-06	-0.001	0.00101	-0.001	-0.0018	0.00168
6.9E-06	-0.0009	0.00098	-0.0002	-0.0011	0.00085
7E-06	-0.0007	0.00083	0.00028	-0.001	0.00144
7E-06	-0.0003	0.00029	9.5E-05	-0.0011	0.00029
7E-06	-0.0004	0.00087	-0.0005	-0.0007	-0.0002
7.1E-06	-0.0005	0.00089	-0.0007	-0.0005	0.00052
7.1E-06	-0.0006	0.00103	-0.0005	-0.0012	0.00089
7.1E-06	-0.0006	0.00083	0.00017	-0.0007	0.00024
7.2E-06	-0.001	0.00143	-4E-05	-0.0007	0.00049
7.2E-06	-0.0006	0.00154	0.00051	-0.0007	0.00044
7.2E-06	-0.0004	0.00151	0.00013	-0.0012	0.00068
7.3E-06	-0.0009	0.00088	8.2E-05	-0.0013	0.00033
7.3E-06	-0.001	0.00108	-0.0002	-0.0005	0.00061
7.3E-06	-0.0008	0.00108	-7E-05	-0.0008	0.0006
7.4E-06	-0.0012	0.00084	0.00024	-0.0013	0.00078
7.4E-06	-0.0007	0.00087	0.00012	-0.0015	0.00082
7.4E-06	-0.0006	0.00108	0.00016	-0.0013	0.00104
7.5E-06	-0.0005	0.00148	0.00053	-0.0013	0.00144

7.5E-06	-0.0006	0.00095	-4E-05	-0.001	0.00051
7.5E-06	-0.0002	0.00118	-0.0005	-0.0015	0.00083
7.6E-06	-0.0006	0.00122	-0.0002	-0.001	0.00041
7.6E-06	-0.0012	0.00086	-3E-05	-0.0015	0.00048
7.6E-06	-0.0007	0.00149	-0.0003	-0.0007	0.00037
7.6E-06	-0.0014	0.00105	5.8E-05	-0.0016	0.00045
7.7E-06	-0.0008	0.00153	-0.0001	-0.0013	5.7E-05
7.7E-06	-0.0004	0.00115	-0.0002	-0.001	0.00161
7.7E-06	-0.0002	0.00087	-0.0002	-0.0012	0.00045
7.8E-06	-0.0011	0.00091	-0.0004	-0.0012	0.00035
7.8E-06	-0.0004	0.00076	-0.0003	-0.0007	0.00083
7.8E-06	-0.0006	0.00051	-4E-05	7.3E-05	0.00081
7.9E-06	-0.0009	0.00068	-0.0001	-0.001	0.00102
7.9E-06	-0.0007	0.00112	-0.001	-0.0012	0.00076
7.9E-06	-0.0006	0.0016	-0.0003	-0.0012	0.00075
8E-06	-0.0008	0.00156	0.00074	-0.0013	0.00155
8E-06	-0.0004	0.00123	-0.0004	-0.0013	0.00074
8E-06	-0.0006	0.00051	4.2E-05	-0.0005	0.00144
8.1E-06	-0.0011	0.00079	0.00045	-0.0008	0.00151
8.1E-06	-0.0008	0.00092	-0.0008	-0.0007	0.00132
8.1E-06	-0.0017	0.00141	-0.0008	-0.0015	0.00106
8.2E-06	-0.0008	0.00136	-0.0003	-0.0007	0.00129
8.2E-06	-0.0004	0.00076	0.0003	-0.0006	0.00076
8.2E-06	-0.0008	0.00106	0.00021	-0.0002	0.00176
8.3E-06	-0.0008	0.00118	-0.0003	-0.0009	0.00118
8.3E-06	-0.0005	0.00078	-0.0003	-0.0008	0.00092
8.3E-06	-0.0003	0.00093	-0.001	-0.0014	0.00117
8.4E-06	-0.0007	0.00093	0.0008	-0.001	0.00094
8.4E-06	-0.0008	0.00043	1.9E-05	-0.0016	0.00083
8.4E-06	-0.0013	0.00107	0.00028	-0.0016	0.00074
8.4E-06	-0.0004	0.00104	-0.0001	-0.0013	0.00041
8.5E-06	-0.0012	0.00122	-0.0004	-0.0018	0.00099
8.5E-06	-0.0012	0.00164	-0.0002	-0.0011	0.0012
8.5E-06	-0.0013	0.0004	-0.0004	-0.0003	0.00119
8.6E-06	-0.0014	0.00103	-0.0005	-0.001	0.00179
8.6E-06	-0.0013	0.00067	2.1E-07	-0.0011	0.00118
8.6E-06	-0.0004	0.00023	-0.0004	-0.0013	0.0008
8.7E-06	-0.0015	0.00117	-0.0009	-0.0008	0.00053
8.7E-06	-0.0015	0.00027	-0.0004	-0.0016	0.00039
8.7E-06	-0.0001	0.00056	-0.0003	-0.0002	0.00084
8.8E-06	-0.0013	0.00044	9.2E-06	-0.0018	0.00097
8.8E-06	-0.0017	-0.0006	-0.0002	-0.001	0.00033
8.8E-06	-0.0011	0.0007	6E-05	-0.001	0.0013
8.9E-06	-0.0012	0.00147	-0.0007	-0.0015	0.00133
8.9E-06	-0.0014	0.00128	-0.0003	-0.0016	0.0003
8.9E-06	-0.0006	0.00069	-0.0004	-0.0011	0.00165
9E-06	-0.0011	0.00099	-0.0008	-0.0001	0.00148
9E-06	-7E-05	0.00072	-0.0001	-0.0008	0.00118
9E-06	-0.001	0.00129	-0.0004	-0.0013	0.0012
9.1E-06	-0.0006	0.00124	-0.0001	-0.0009	0.00044
9.1E-06	-0.0007	0.00089	-0.0004	-0.0011	0.00113
9.1E-06	-0.0005	0.00084	-0.0004	-0.0002	0.00094
9.2E-06	-0.0006	0.00048	-0.0005	-0.0003	0.00116
9.2E-06	-0.0011	0.00025	-0.0004	-0.0002	0.00093

9.2E-06	-0.0011	0.0003	-0.0003	-0.0009	0.00102
9.2E-06	-0.0008	0.00106	-0.0004	-0.001	0.00053
9.3E-06	-0.001	0.00082	-0.0007	-0.0004	0.00128
9.3E-06	4.6E-05	0.00114	-0.0002	-0.0012	0.00095
9.3E-06	-0.001	0.00133	0.00024	-0.0019	0.00096
9.4E-06	-0.0018	0.00082	-0.0004	-0.0009	0.00139
9.4E-06	-0.0014	0.00066	-0.0002	-0.0017	0.00074
9.4E-06	-0.0015	0.00053	-0.001	-0.0006	0.00073
9.5E-06	-0.0016	0.00083	0.00013	-0.0008	0.00022
9.5E-06	-0.0011	0.00121	0.00022	-0.0001	0.00136
9.5E-06	-0.0006	0.00093	0.00039	-0.0013	0.00081
9.6E-06	-0.0011	0.0006	0.00021	-0.0017	0.00113
9.6E-06	0.00011	0.00087	0.00028	-0.0013	0.00091
9.6E-06	-0.0011	0.00116	-0.0001	-0.0012	0.00037
9.7E-06	-0.0012	0.00087	0.00014	-0.0013	-2E-05
9.7E-06	-0.0015	0.00075	-0.0006	-0.0006	0.00074
9.7E-06	-0.0009	0.00104	-0.0012	-0.0003	0.00112
9.8E-06	-0.0013	0.00047	0.00016	-0.0015	0.00034
9.8E-06	-0.0015	0.00064	-0.0005	-0.0004	0.00052
9.8E-06	-0.0012	0.00045	-0.0006	-0.0015	0.00041
9.9E-06	-0.0004	0.00054	-0.0004	-0.0001	0.00071
9.9E-06	-0.0015	0.00153	3.3E-05	-0.0013	0.00055
9.9E-06	-0.0006	0.00071	-0.0005	-0.0012	0.0006
1E-05	-0.0015	0.00085	2.3E-05	-0.0013	0.00099
1E-05	-0.0003	0.00047	6.2E-05	-0.0005	0.00051
1E-05	-0.0019	0.00095	0.00011	-0.0006	0.00063
1E-05	-0.0011	0.0011	0.00021	-0.0001	0.00144
1E-05	-0.0012	0.00081	0.00019	-0.0011	0.00059
1E-05	-0.0005	0.00095	0.00031	-0.001	0.0007
1E-05	-0.0007	0.00144	-0.0004	-0.0012	0.00124
1E-05	-0.0004	0.00127	-0.0004	-0.0011	0.00082
1E-05	-0.0013	0.00088	-0.0004	-0.0014	0.00043
1E-05	-0.0011	0.00056	0.00063	-0.0014	0.00102
1E-05	-0.0018	0.00075	8.7E-05	-0.0015	0.00097
1E-05	-0.0009	0.00139	-0.0002	-0.0011	0.0014
1E-05	-0.0017	0.00076	0.00044	-0.0012	0.0006
1E-05	-0.0011	0.00039	0.00088	-0.0009	0.00135
1E-05	-0.0019	0.00021	7E-06	-0.0003	0.00018
1E-05	-0.002	3.9E-05	-0.0003	-0.0012	0.00093
1E-05	-0.0005	0.0011	0.00076	-0.0004	0.00081
1.1E-05	-0.0009	0.00102	0.00024	-0.0014	0.00052
1.1E-05	-0.0011	0.00052	7.1E-05	-0.0018	0.00028
1.1E-05	-0.0014	0.00078	-0.0004	-0.0014	0.00078
1.1E-05	-0.0002	0.00124	-1E-04	8.7E-05	0.00088
1.1E-05	-0.0013	0.00075	-0.0004	-0.0012	0.00089
1.1E-05	-0.0015	0.00074	0.00019	-0.0006	0.00046
1.1E-05	-0.0006	0.00039	3.5E-05	-0.0013	0.00085
1.1E-05	-0.0019	0.00015	6.6E-05	-0.0014	0.00038
1.1E-05	-0.0008	0.00135	8.6E-05	-0.0005	0.00016
1.1E-05	-0.0007	0.00064	0.00038	0.00039	-0.0002
1.1E-05	-0.001	0.00046	0.00027	-0.0009	0.0012
1.1E-05	-0.0009	0.00094	-0.0002	-0.0008	0.00035
1.1E-05	-0.0008	0.00061	-0.0006	-0.0008	0.00101
1.1E-05	-0.0007	0.00106	-0.0002	-0.0008	0.00077

1.1E-05	-0.0013	0.00093	-0.0001	-0.0006	0.00058
1.1E-05	-0.0012	0.00012	-1E-05	-0.0005	0.00084
1.1E-05	-0.0008	0.00038	-0.0003	-0.0002	0.00123
1.1E-05	-0.0007	0.00069	-0.0013	-0.0008	0.00066
1.1E-05	-0.0004	0.00085	-0.0005	-0.0002	0.00104
1.1E-05	-0.0014	0.00069	-0.0003	-0.0013	0.00079
1.1E-05	-0.0013	0.00113	-5E-05	-0.0013	-2E-05
1.1E-05	-0.0011	0.00103	1.9E-05	-0.001	0.0005
1.1E-05	-0.0012	0.00064	7.2E-05	-0.0009	0.00043
1.1E-05	-0.0007	0.00116	-0.0008	-0.0016	0.00017
1.1E-05	-0.001	0.00089	0.00054	-0.0012	0.00068
1.1E-05	-0.0008	3.2E-05	-0.0009	-0.0014	0.00054
1.1E-05	-0.0016	0.00089	-0.0006	-0.0022	0.00025
1.1E-05	-0.001	0.00104	-0.0011	-0.0013	0.00106
1.1E-05	-0.0011	0.0006	0.00041	-0.0014	0.0008
1.1E-05	-0.0005	0.00032	-0.0002	-0.0002	0.00056
1.1E-05	-0.0011	0.00145	-0.0004	-0.0003	0.00071
1.1E-05	-0.0008	0.00054	-5E-05	-0.0003	0.0009
1.2E-05	-0.0012	0.00057	-0.0002	-0.0016	0.00108
1.2E-05	-0.0013	0.00126	0.00034	-0.0007	0.00074
1.2E-05	-0.0005	0.00051	0.00034	-0.0007	0.00036
1.2E-05	-0.0007	9.1E-05	-0.0008	-0.0009	0.0003
1.2E-05	-0.0004	0.00034	0.00029	-0.0012	0.00048
1.2E-05	-0.0011	0.00044	-0.0009	-0.001	0.00015
1.2E-05	-0.0009	0.00084	-4E-05	-0.0008	0.00042
1.2E-05	-0.0009	9.6E-05	0.00049	-0.0026	0.00131
1.2E-05	-0.0014	0.00094	0.00017	-0.0013	0.00083
1.2E-05	-0.0004	0.00132	-0.0003	-0.0015	0.00101
1.2E-05	-0.0016	0.00084	0.0001	-0.0014	0.00062
1.2E-05	-0.0011	0.00095	0.00041	-0.0008	0.00065
1.2E-05	-0.001	0.00122	-0.0016	-0.0004	0.00096
1.2E-05	-0.0015	0.00095	8.4E-05	-0.0008	0.00046
1.2E-05	-0.0012	0.00122	-0.001	-0.002	4.8E-05
1.2E-05	-0.0011	0.00027	-0.0004	-0.0013	0.00064
1.2E-05	-0.0009	0.00095	-2E-05	-0.0007	0.00082
1.2E-05	-0.0011	0.00054	-1E-05	-0.0006	0.00024
1.2E-05	-0.001	0.00108	-0.0001	-0.0007	0.00106
1.2E-05	-0.0015	0.00077	0.00051	-0.001	0.00076
1.2E-05	-0.0015	0.00126	0.00013	-0.0009	0.00022
1.2E-05	-0.0007	0.00087	0.00021	-0.0011	0.00071
1.2E-05	-0.0015	0.0009	-0.0009	-0.0007	0.0009
1.2E-05	-0.0007	0.00104	-0.0008	-0.0011	0.00151
1.2E-05	-0.0004	0.00149	-0.0002	-0.0017	0.00048
1.2E-05	-0.0012	0.00056	0.00012	-0.0011	0.00055
1.2E-05	-0.0008	0.00146	0.00031	-0.0017	-0.0001
1.2E-05	-0.0013	0.00109	0.00051	-0.0015	0.00102
1.2E-05	-0.0005	0.00047	0.00013	-0.0012	0.00011
1.2E-05	-0.0008	0.00074	-0.0006	-0.0012	0.00027
1.2E-05	-0.0007	0.00151	-0.0004	-0.001	0.00081
1.3E-05	-0.0009	0.00118	-1E-04	-0.0009	0.00055
1.3E-05	-0.0011	0.00114	-0.0002	-0.0013	0.00181
1.3E-05	-0.0003	0.00054	-0.0008	-0.0014	0.00059
1.3E-05	-0.0015	0.00066	-0.0005	-0.0009	0.00096
1.3E-05	-0.0012	0.001	0.00031	-0.0016	5.2E-05

1.3E-05	-0.0007	0.00031	-0.0005	-0.0014	0.0013
1.3E-05	-0.0011	0.0002	0.00028	-0.0002	0.00058
1.3E-05	-0.0002	0.00035	0.00072	-0.0013	0.00017
1.3E-05	-0.0007	0.00119	9.3E-05	-0.0011	0.00063
1.3E-05	-0.0009	7E-05	0.00036	-0.0007	0.00016
1.3E-05	-0.0005	0.00035	-0.0001	-0.0019	0.00034
1.3E-05	-0.0008	0.00109	0.00017	-0.0013	0.00117
1.3E-05	-0.0008	0.00057	-0.0005	-0.001	0.00124
1.3E-05	-0.0007	0.0013	-0.0006	-0.0016	0.00083
1.3E-05	-0.0008	0.00098	-0.0003	-0.0014	0.00028
1.3E-05	-0.0006	0.00071	-0.0002	-0.0011	-9E-05
1.3E-05	-0.0004	0.00107	-0.0006	-0.0013	0.00108
1.3E-05	-0.0002	0.001	-0.0009	-8E-07	0.00052
1.3E-05	-0.0007	-6E-05	-0.0004	-0.0001	-2E-05
1.3E-05	-0.0013	0.00043	-0.0008	-0.0014	0.00033
1.3E-05	-0.001	-0.0003	-0.0006	-0.0019	-0.0001
1.3E-05	-0.0004	0.00046	-0.0008	-0.0014	0.00084
1.3E-05	-0.0009	0.00094	0.00087	-0.0013	0.00071
1.3E-05	-0.0013	0.00093	-0.0006	-0.0008	0.00049
1.3E-05	-0.0012	0.00073	0.0006	-0.001	0.00116
1.3E-05	-0.0012	0.00117	-0.0002	-0.0009	0.00076
1.3E-05	-0.0012	0.0002	-0.0002	-0.0013	0.00067
1.3E-05	-0.0017	-0.0004	-0.0006	-0.0008	-4E-05
1.3E-05	-0.0005	0.00137	0.00024	-0.0009	-0.0002
1.3E-05	-0.0005	0.00069	-0.0003	-0.0014	2E-05
1.3E-05	-0.0004	0.00055	-0.0001	-0.0008	0.00053
1.4E-05	-0.0006	-0.0002	-0.0008	7.2E-05	0.00057
1.4E-05	-0.001	0.00073	8.5E-05	-0.0013	5.6E-05
1.4E-05	-0.0008	0.00036	0.0004	5.5E-05	0.00017
1.4E-05	-0.0004	0.00048	0.00037	-0.001	0.00099
1.4E-05	-0.0007	0.00065	-0.0002	-0.001	0.00075
1.4E-05	-0.0007	0.00084	0.00028	-0.001	0.00084
1.4E-05	-0.0014	0.00016	3.2E-05	-0.0009	0.001
1.4E-05	-0.0008	0.00174	0.00019	-0.0017	0.00085
1.4E-05	-0.0002	0.00063	-4E-05	-0.0018	0.00095
1.4E-05	-0.0013	0.00108	-0.0004	-0.001	0.00112
1.4E-05	-0.0008	0.00097	-0.0005	-0.0008	0.00052
1.4E-05	-0.0009	0.00083	-0.0004	-0.0017	0.00093
1.4E-05	-0.0008	0.00019	0.00088	-0.0014	0.00093
1.4E-05	-0.0009	9.3E-05	0.00066	-0.0016	0.00056
1.4E-05	-0.0008	0.0002	-0.0008	-0.0006	0.00057
1.4E-05	-0.0007	0.00011	-0.0011	-0.0005	0.00022
1.4E-05	-0.0009	0.00043	-0.0009	-0.0016	0.00025
1.4E-05	-0.001	0.0008	-0.0007	-0.0014	0.00034
1.4E-05	-0.0005	0.0004	-0.0001	-0.0011	0.00117
1.4E-05	-0.0009	0.00113	-0.0003	-0.0009	0.00131
1.4E-05	-0.0007	0.00083	-0.0001	-0.0004	0.00074
1.4E-05	-0.0011	0.00074	0.00029	-0.0009	0.0009
1.4E-05	-0.0011	0.00067	-0.0005	-0.0011	0.00044
1.4E-05	-0.0006	0.00073	0.00029	-0.0007	-1E-04
1.4E-05	-0.0007	0.00054	-0.0004	-0.0009	0.00096
1.4E-05	-0.0003	0.00038	-0.0005	-0.0015	0.00067
1.4E-05	-0.0002	0.0012	-0.0009	-0.0008	0.00096
1.4E-05	-0.0008	0.00069	0.00037	-0.0016	0.00069

1.4E-05	-0.0014	0.00073	-0.0002	-0.001	0.00084
1.4E-05	-0.0015	0.00156	-0.0004	-0.0009	0.00073
1.4E-05	-0.0011	0.0012	-0.0004	-0.0015	0.00086
1.5E-05	-0.0009	0.00062	-0.0012	-6E-05	0.00056
1.5E-05	-0.0008	5.6E-06	-0.0006	-0.0009	0.00065
1.5E-05	-0.0005	0.00074	-0.0011	-0.0011	0.00094
1.5E-05	-0.0005	0.00062	-0.0003	-0.0011	0.00085
1.5E-05	-0.0009	0.0006	-7E-05	-0.0017	0.00109
1.5E-05	-0.0007	0.00043	-0.0005	-0.0014	1.9E-05
1.5E-05	-0.0005	0.00061	7.7E-05	-0.0017	0.00097
1.5E-05	-0.0007	0.0006	-0.0007	-0.0011	-0.0002
1.5E-05	-0.0009	0.00091	-0.0011	-0.0013	0.00049
1.5E-05	-0.0004	0.00081	0.00026	-0.0013	-4E-05
1.5E-05	-0.0005	0.00049	-0.0003	-0.0014	0.00031
1.5E-05	-0.0013	0.00025	-0.0009	-0.0009	0.00144
1.5E-05	-0.001	0.00043	-0.0004	-0.0009	0.00094
1.5E-05	-0.0004	0.00023	-1E-04	-0.0013	0.00074
1.5E-05	-0.0006	0.00105	3.3E-05	-0.0014	0.0005
1.5E-05	-0.0006	0.0008	-0.0006	-0.0017	0.00081

Table SI.15B: Primary kinetic data for the reaction of 1,3-dimethyl-2-imidazolidinone (**S15**) with CumO• generated by 355 nm LFP in DMSO (**Figure SI.15B**).

t / s	ΔA 1,3-dimethyl-2-imidazolidinone (S15)				
	3.92 mM	8.6 mM	13.9 mM	19.8 mM	26.1 mM
-9.92E-07	-9.94E-05	2.95E-04	-3.41E-04	-4.27E-04	-1.44E-04
-9.60E-07	-1.98E-04	3.18E-04	-1.02E-04	-2.35E-04	-5.42E-04
-9.28E-07	-4.57E-04	5.57E-04	-3.95E-04	-2.31E-04	-1.99E-04
-8.96E-07	-2.40E-04	2.62E-04	-3.10E-04	-6.66E-04	-7.29E-04
-8.64E-07	2.64E-04	3.24E-04	1.49E-04	-3.54E-04	-9.14E-04
-8.32E-07	-2.74E-04	-7.53E-05	4.24E-05	-8.97E-04	-4.87E-04
-8.00E-07	2.01E-04	2.20E-04	1.15E-04	-3.88E-04	-4.34E-04
-7.68E-07	3.00E-04	2.93E-04	-1.10E-04	-6.68E-04	-4.34E-04
-7.36E-07	-1.52E-04	1.22E-04	-4.83E-04	-2.71E-04	-2.37E-04
-7.04E-07	-2.98E-04	-2.63E-04	-3.44E-04	-4.99E-04	-4.05E-04
-6.72E-07	1.13E-04	8.68E-05	1.34E-04	-3.48E-04	-1.32E-04
-6.40E-07	2.24E-06	-7.75E-05	4.72E-05	-2.52E-04	-6.14E-04
-6.08E-07	1.78E-04	1.17E-04	4.30E-04	-6.27E-04	1.15E-04
-5.76E-07	-1.47E-04	1.21E-04	-3.56E-05	-4.92E-04	-3.20E-04
-5.44E-07	-4.64E-06	5.49E-04	-5.51E-04	-2.35E-04	-5.85E-04
-5.12E-07	-6.18E-04	3.77E-04	-2.29E-04	2.70E-05	-3.13E-04
-4.80E-07	-8.30E-04	3.81E-04	-1.14E-03	-2.83E-04	-1.01E-03
-4.48E-07	-6.51E-05	5.41E-05	1.27E-04	-4.84E-04	-5.93E-04
-4.16E-07	-5.27E-04	1.18E-04	-5.42E-04	-5.32E-04	-2.13E-04
-3.84E-07	-2.73E-04	2.71E-04	-4.81E-04	-4.86E-05	-1.54E-04
-3.52E-07	-8.30E-05	-1.14E-04	-6.34E-04	-6.29E-04	-8.26E-04
-3.20E-07	-5.32E-04	-1.13E-04	-4.15E-06	-4.98E-04	-2.10E-04
-2.88E-07	-8.42E-05	8.85E-05	-1.76E-04	-2.66E-04	-9.43E-04
-2.56E-07	-7.77E-04	2.95E-05	1.29E-04	-2.59E-04	-5.60E-04
-2.24E-07	-2.34E-04	3.80E-04	2.14E-05	-4.63E-04	-2.22E-04
-1.92E-07	-3.08E-04	8.41E-05	1.36E-04	-1.66E-04	-3.36E-04
-1.60E-07	-4.63E-04	3.64E-05	-4.08E-04	3.32E-04	8.04E-05
-1.28E-07	-2.31E-04	-2.26E-05	-1.99E-04	-6.05E-04	-7.77E-05
-9.63E-08	-8.39E-05	5.24E-05	-2.47E-04	-3.06E-04	1.05E-04
-6.43E-08	-2.02E-06	9.77E-05	-1.84E-04	-4.66E-04	7.08E-04
-3.23E-08	5.29E-05	-1.84E-04	7.75E-05	-3.74E-05	-3.65E-04
-3.16E-10	-2.77E-03	-2.88E-03	-3.12E-03	-2.73E-03	-2.87E-03
3.17E-08	-8.93E-03	-9.15E-03	-1.12E-02	-9.95E-03	-9.26E-03
6.37E-08	1.51E-02	1.51E-02	1.46E-02	1.48E-02	1.32E-02
9.57E-08	1.58E-02	1.56E-02	1.51E-02	1.49E-02	1.34E-02
1.28E-07	1.54E-02	1.51E-02	1.44E-02	1.41E-02	1.29E-02
1.60E-07	1.48E-02	1.50E-02	1.35E-02	1.32E-02	1.13E-02
1.92E-07	1.46E-02	1.43E-02	1.27E-02	1.32E-02	1.09E-02
2.24E-07	1.40E-02	1.38E-02	1.28E-02	1.22E-02	1.05E-02
2.56E-07	1.34E-02	1.34E-02	1.18E-02	1.18E-02	9.63E-03
2.88E-07	1.38E-02	1.30E-02	1.10E-02	1.03E-02	9.27E-03
3.20E-07	1.31E-02	1.26E-02	1.10E-02	1.07E-02	8.78E-03
3.52E-07	1.24E-02	1.20E-02	1.03E-02	9.77E-03	8.10E-03
3.84E-07	1.19E-02	1.20E-02	1.03E-02	8.84E-03	7.84E-03
4.16E-07	1.16E-02	1.12E-02	9.00E-03	9.23E-03	7.04E-03
4.48E-07	1.11E-02	1.08E-02	9.64E-03	8.83E-03	6.65E-03
4.80E-07	1.11E-02	1.11E-02	8.26E-03	7.73E-03	6.55E-03
5.12E-07	1.14E-02	1.03E-02	8.36E-03	7.17E-03	6.42E-03
5.44E-07	1.04E-02	1.02E-02	7.74E-03	7.04E-03	5.79E-03

5.76E-07	1.06E-02	9.53E-03	6.88E-03	6.96E-03	5.11E-03
6.08E-07	9.69E-03	9.00E-03	6.64E-03	6.40E-03	4.52E-03
6.40E-07	9.24E-03	8.92E-03	7.11E-03	6.36E-03	5.06E-03
6.72E-07	9.29E-03	8.85E-03	6.30E-03	6.40E-03	4.73E-03
7.04E-07	9.09E-03	7.78E-03	6.41E-03	5.82E-03	4.37E-03
7.36E-07	9.00E-03	7.80E-03	6.71E-03	4.70E-03	3.51E-03
7.68E-07	7.88E-03	7.67E-03	6.42E-03	5.12E-03	3.61E-03
8.00E-07	7.85E-03	7.80E-03	5.25E-03	4.81E-03	3.94E-03
8.32E-07	7.57E-03	7.82E-03	5.10E-03	4.25E-03	3.13E-03
8.64E-07	7.92E-03	7.31E-03	4.91E-03	3.93E-03	3.46E-03
8.96E-07	7.77E-03	7.32E-03	5.17E-03	3.86E-03	3.21E-03
9.28E-07	6.50E-03	6.96E-03	4.36E-03	3.86E-03	2.70E-03
9.60E-07	6.27E-03	6.61E-03	4.22E-03	4.00E-03	2.63E-03
9.92E-07	6.22E-03	5.84E-03	4.20E-03	3.81E-03	2.44E-03
1.02E-06	6.17E-03	5.91E-03	3.81E-03	3.31E-03	2.54E-03
1.06E-06	6.18E-03	5.57E-03	4.23E-03	2.96E-03	2.79E-03
1.09E-06	5.68E-03	5.53E-03	4.03E-03	2.87E-03	2.44E-03
1.12E-06	5.17E-03	5.64E-03	3.78E-03	2.92E-03	2.06E-03
1.15E-06	5.38E-03	5.77E-03	3.60E-03	2.49E-03	1.66E-03
1.18E-06	5.36E-03	5.54E-03	3.34E-03	2.43E-03	1.35E-03
1.22E-06	5.97E-03	5.39E-03	3.54E-03	2.52E-03	1.91E-03
1.25E-06	5.41E-03	5.29E-03	3.07E-03	2.50E-03	1.74E-03
1.28E-06	4.65E-03	5.09E-03	3.05E-03	2.09E-03	1.52E-03
1.31E-06	5.21E-03	4.33E-03	2.47E-03	2.33E-03	1.75E-03
1.34E-06	4.72E-03	5.03E-03	3.45E-03	2.32E-03	1.11E-03
1.38E-06	4.93E-03	4.68E-03	2.86E-03	1.40E-03	2.02E-03
1.41E-06	4.83E-03	4.02E-03	2.95E-03	3.03E-03	2.22E-03
1.44E-06	4.33E-03	4.51E-03	2.02E-03	2.49E-03	1.42E-03
1.47E-06	4.76E-03	5.01E-03	2.41E-03	1.63E-03	1.34E-03
1.50E-06	4.27E-03	4.11E-03	2.75E-03	1.36E-03	1.02E-03
1.54E-06	3.94E-03	4.49E-03	2.55E-03	1.38E-03	6.72E-04
1.57E-06	4.39E-03	3.64E-03	2.07E-03	1.06E-03	1.02E-03
1.60E-06	3.42E-03	3.43E-03	3.31E-03	1.21E-03	8.66E-04
1.63E-06	4.27E-03	3.92E-03	2.44E-03	1.21E-03	6.32E-04
1.66E-06	3.64E-03	3.87E-03	1.93E-03	1.45E-03	1.10E-03
1.70E-06	3.47E-03	3.47E-03	1.62E-03	1.96E-03	8.11E-04
1.73E-06	3.30E-03	3.77E-03	1.76E-03	1.74E-03	7.09E-04
1.76E-06	3.61E-03	3.43E-03	2.51E-03	1.37E-03	7.61E-04
1.79E-06	3.28E-03	3.41E-03	1.74E-03	1.03E-03	5.98E-04
1.82E-06	3.23E-03	2.99E-03	1.51E-03	1.11E-03	1.02E-03
1.86E-06	3.13E-03	2.81E-03	1.97E-03	8.16E-04	1.01E-03
1.89E-06	2.73E-03	3.30E-03	1.07E-03	9.36E-04	7.47E-04
1.92E-06	2.55E-03	3.35E-03	1.45E-03	1.16E-03	7.42E-04
1.95E-06	3.39E-03	3.31E-03	6.20E-04	5.83E-04	6.84E-04
1.98E-06	2.47E-03	2.57E-03	1.35E-03	1.30E-03	9.47E-04
2.02E-06	2.84E-03	3.25E-03	1.71E-03	1.26E-03	6.96E-04
2.05E-06	2.60E-03	3.19E-03	9.92E-04	1.05E-03	4.81E-04
2.08E-06	2.67E-03	2.89E-03	1.14E-03	8.09E-04	1.39E-04
2.11E-06	2.47E-03	2.74E-03	1.14E-03	8.62E-04	2.43E-04
2.14E-06	2.28E-03	2.41E-03	1.05E-03	7.89E-04	1.10E-03
2.18E-06	1.71E-03	1.97E-03	9.19E-04	1.05E-03	5.77E-04
2.21E-06	2.09E-03	2.55E-03	1.24E-03	1.26E-03	2.73E-04
2.24E-06	2.70E-03	2.72E-03	1.39E-03	-1.01E-04	7.42E-04
2.27E-06	2.41E-03	2.56E-03	1.15E-03	8.03E-04	1.34E-04

2.30E-06	2.52E-03	2.29E-03	7.28E-04	6.91E-04	5.99E-04
2.34E-06	2.37E-03	2.90E-03	7.81E-04	7.25E-04	5.74E-04
2.37E-06	3.12E-03	2.90E-03	6.27E-04	7.66E-04	5.39E-04
2.40E-06	1.94E-03	2.62E-03	9.44E-04	9.74E-04	2.80E-04
2.43E-06	2.03E-03	2.32E-03	1.02E-03	9.38E-04	3.35E-04
2.46E-06	1.69E-03	2.19E-03	6.65E-04	6.63E-04	1.49E-04
2.50E-06	2.82E-03	2.01E-03	1.24E-03	8.55E-04	9.95E-05
2.53E-06	2.21E-03	2.28E-03	8.03E-04	8.00E-04	9.93E-04
2.56E-06	2.02E-03	2.63E-03	5.60E-04	7.95E-04	4.52E-04
2.59E-06	1.32E-03	2.00E-03	7.34E-04	1.05E-03	7.68E-04
2.62E-06	1.47E-03	3.05E-03	6.68E-04	1.18E-03	-6.06E-05
2.66E-06	2.13E-03	2.42E-03	2.92E-04	6.75E-04	3.35E-04
2.69E-06	2.04E-03	2.67E-03	6.62E-04	3.33E-04	2.71E-04
2.72E-06	2.28E-03	2.45E-03	8.29E-04	7.63E-04	3.41E-05
2.75E-06	1.75E-03	2.19E-03	5.70E-04	1.06E-03	6.50E-04
2.78E-06	1.40E-03	2.44E-03	9.03E-04	8.75E-04	6.53E-04
2.82E-06	1.30E-03	1.80E-03	1.16E-03	4.75E-04	4.98E-04
2.85E-06	1.48E-03	2.56E-03	5.78E-04	5.26E-04	5.35E-04
2.88E-06	1.85E-03	2.48E-03	6.05E-04	6.71E-04	6.01E-04
2.91E-06	1.47E-03	1.56E-03	7.98E-04	5.05E-04	6.91E-04
2.94E-06	1.46E-03	2.20E-03	9.02E-05	6.55E-04	4.36E-04
2.98E-06	1.11E-03	1.87E-03	7.95E-04	2.31E-04	2.50E-04
3.01E-06	1.08E-03	1.83E-03	6.40E-04	4.31E-04	8.07E-04
3.04E-06	9.83E-04	1.57E-03	5.41E-04	4.26E-04	1.18E-04
3.07E-06	8.43E-04	1.31E-03	7.28E-04	4.26E-04	4.83E-04
3.10E-06	1.02E-03	2.12E-03	3.22E-04	5.13E-04	5.10E-04
3.14E-06	7.84E-04	1.57E-03	4.13E-04	3.20E-04	6.84E-04
3.17E-06	8.29E-04	1.24E-03	7.35E-04	2.31E-04	3.51E-04
3.20E-06	1.34E-03	1.79E-03	5.67E-04	4.52E-04	4.64E-04
3.23E-06	1.48E-03	1.73E-03	1.18E-03	2.47E-04	5.44E-04
3.26E-06	1.38E-03	1.85E-03	7.84E-04	7.02E-04	5.20E-04
3.30E-06	1.05E-03	1.91E-03	5.76E-04	1.34E-04	4.49E-04
3.33E-06	1.33E-03	1.86E-03	4.36E-04	1.84E-04	9.53E-04
3.36E-06	1.00E-03	1.37E-03	4.39E-04	6.87E-04	3.42E-04
3.39E-06	7.61E-04	1.29E-03	5.57E-05	2.68E-04	6.28E-04
3.42E-06	1.08E-03	1.99E-03	3.57E-04	5.10E-04	6.54E-04
3.46E-06	7.34E-04	1.27E-03	2.36E-04	2.06E-04	3.93E-04
3.49E-06	1.29E-03	1.25E-03	4.19E-04	2.71E-04	6.48E-04
3.52E-06	8.93E-04	8.34E-04	4.87E-04	1.31E-03	3.20E-04
3.55E-06	4.99E-04	1.38E-03	5.05E-04	2.21E-04	6.33E-04
3.58E-06	5.88E-04	1.08E-03	6.22E-04	-1.76E-04	5.35E-04
3.62E-06	8.05E-04	1.28E-03	2.09E-04	2.64E-04	4.41E-04
3.65E-06	3.57E-04	1.07E-03	4.34E-04	2.64E-04	4.23E-04
3.68E-06	4.41E-04	1.11E-03	3.11E-04	5.74E-04	5.77E-04
3.71E-06	7.76E-04	1.30E-03	4.10E-04	-4.23E-05	-8.77E-05
3.74E-06	9.33E-04	1.52E-03	3.86E-04	1.95E-04	4.76E-04
3.78E-06	6.78E-04	1.52E-03	5.79E-04	4.71E-04	1.14E-04
3.81E-06	5.28E-04	2.26E-03	5.60E-04	6.72E-04	6.04E-04
3.84E-06	8.05E-04	1.94E-03	1.80E-04	3.76E-04	1.91E-04
3.87E-06	9.40E-04	2.18E-03	7.06E-04	4.83E-04	5.18E-04
3.90E-06	8.37E-04	1.01E-03	3.21E-04	2.05E-04	6.39E-04
3.94E-06	1.11E-03	1.28E-03	5.88E-04	1.45E-04	7.13E-04
3.97E-06	5.76E-04	1.40E-03	6.54E-04	3.06E-04	3.23E-04
4.00E-06	5.11E-04	1.32E-03	2.98E-04	1.04E-03	6.62E-04

4.03E-06	4.92E-04	1.55E-03	1.84E-04	4.45E-04	5.88E-04
4.06E-06	4.25E-04	1.62E-03	5.89E-04	7.07E-05	9.34E-05
4.10E-06	1.01E-03	1.37E-03	3.58E-04	5.34E-04	2.32E-04
4.13E-06	8.53E-04	1.62E-03	5.45E-04	7.46E-05	6.37E-04
4.16E-06	5.74E-04	1.65E-03	4.23E-04	4.73E-04	2.56E-04
4.19E-06	9.06E-04	1.63E-03	3.86E-04	5.32E-04	4.53E-04
4.22E-06	5.23E-04	1.75E-03	3.55E-04	7.11E-04	6.65E-04
4.26E-06	1.22E-03	1.53E-03	3.62E-04	8.92E-04	1.46E-04
4.29E-06	1.07E-03	1.99E-03	5.87E-04	6.69E-04	6.52E-04
4.32E-06	3.58E-04	1.81E-03	3.35E-04	7.56E-04	-2.13E-04
4.35E-06	6.85E-04	1.27E-03	4.08E-04	2.06E-04	6.65E-05
4.38E-06	5.56E-04	1.86E-03	3.94E-04	1.94E-04	6.68E-04
4.42E-06	5.37E-04	1.15E-03	8.24E-04	-8.22E-05	2.82E-04
4.45E-06	8.86E-04	1.31E-03	4.42E-04	7.43E-04	2.92E-04
4.48E-06	3.54E-04	1.37E-03	4.79E-04	3.66E-04	5.93E-04
4.51E-06	3.85E-04	1.76E-03	1.70E-04	1.06E-04	7.01E-04
4.54E-06	1.08E-03	1.36E-03	2.70E-04	3.43E-04	2.72E-04
4.58E-06	6.08E-04	1.61E-03	4.01E-04	5.88E-04	7.16E-04
4.61E-06	4.74E-04	1.49E-03	3.40E-04	6.85E-04	4.46E-04
4.64E-06	1.28E-03	1.35E-03	-1.44E-04	7.32E-05	6.42E-04
4.67E-06	3.62E-04	1.90E-03	4.28E-04	6.10E-04	1.11E-04
4.70E-06	3.03E-04	1.81E-03	4.84E-04	4.47E-04	5.42E-04
4.74E-06	4.07E-04	1.85E-03	8.41E-04	-1.37E-04	7.37E-05
4.77E-06	6.74E-04	1.82E-03	5.19E-04	2.60E-04	2.31E-04
4.80E-06	6.41E-04	1.71E-03	6.04E-04	3.24E-04	2.22E-04
4.83E-06	4.21E-04	1.76E-03	2.95E-04	5.16E-04	1.85E-04
4.86E-06	3.95E-04	1.40E-03	3.23E-04	5.85E-04	5.49E-04
4.90E-06	1.12E-03	1.45E-03	5.53E-04	1.74E-04	3.85E-04
4.93E-06	4.52E-04	1.66E-03	4.35E-04	3.26E-04	4.84E-04
4.96E-06	5.72E-04	1.39E-03	2.92E-04	6.23E-04	8.65E-04
4.99E-06	7.01E-04	1.95E-03	3.43E-04	-2.82E-05	3.39E-04
5.02E-06	4.60E-04	1.48E-03	2.62E-04	4.61E-04	9.54E-05
5.06E-06	1.14E-03	1.85E-03	2.32E-04	7.73E-04	-7.07E-05
5.09E-06	7.23E-04	1.44E-03	2.39E-04	3.72E-04	8.02E-04
5.12E-06	6.07E-04	1.92E-03	1.46E-04	3.74E-04	-5.80E-05
5.15E-06	1.38E-03	1.37E-03	5.44E-04	5.76E-04	5.75E-04
5.18E-06	6.09E-04	1.80E-03	4.28E-04	5.91E-04	6.66E-04
5.22E-06	6.59E-04	1.80E-03	5.50E-04	3.86E-04	8.19E-04
5.25E-06	9.72E-04	1.57E-03	-2.03E-04	7.08E-04	3.62E-04
5.28E-06	8.29E-04	1.33E-03	7.67E-04	6.44E-04	2.23E-04
5.31E-06	1.14E-03	1.38E-03	3.32E-04	6.43E-04	6.08E-04
5.34E-06	9.48E-04	9.50E-04	2.60E-04	8.85E-06	2.77E-04
5.38E-06	6.53E-04	1.29E-03	4.75E-04	2.39E-04	5.95E-04
5.41E-06	1.10E-03	1.36E-03	1.66E-04	5.37E-04	5.51E-04
5.44E-06	8.94E-04	1.96E-03	1.82E-04	5.44E-04	3.21E-04
5.47E-06	7.24E-04	1.84E-03	2.28E-04	5.22E-04	2.61E-04
5.50E-06	4.27E-04	1.24E-03	3.15E-04	4.64E-04	3.20E-04
5.54E-06	2.40E-04	1.43E-03	5.18E-04	4.36E-04	5.50E-04
5.57E-06	5.31E-04	1.46E-03	1.65E-04	5.81E-04	5.31E-04
5.60E-06	5.31E-04	1.49E-03	6.08E-04	7.44E-04	1.78E-04
5.63E-06	4.45E-04	1.82E-03	7.11E-04	3.96E-04	5.31E-04
5.66E-06	2.98E-04	2.04E-03	4.07E-04	1.39E-04	4.97E-04
5.70E-06	4.59E-04	1.93E-03	4.07E-04	5.72E-04	2.50E-04
5.73E-06	1.05E-03	1.52E-03	3.52E-04	1.01E-04	2.81E-04

5.76E-06	1.05E-03	9.94E-04	3.80E-04	3.23E-04	4.32E-04
5.79E-06	7.94E-04	1.50E-03	5.77E-05	6.01E-04	4.59E-04
5.82E-06	1.15E-03	1.71E-03	4.52E-04	1.61E-04	4.21E-04
5.86E-06	6.10E-04	1.44E-03	5.20E-04	-4.23E-05	4.25E-04
5.89E-06	6.73E-04	1.61E-03	4.77E-04	3.19E-04	6.03E-04
5.92E-06	7.38E-04	1.62E-03	2.49E-04	5.05E-06	2.18E-04
5.95E-06	7.61E-04	1.70E-03	5.86E-04	3.80E-05	6.36E-04
5.98E-06	3.31E-04	1.54E-03	3.98E-04	2.70E-04	1.48E-04
6.02E-06	3.10E-04	1.62E-03	-2.67E-05	3.59E-04	3.62E-04
6.05E-06	3.91E-04	1.62E-03	5.21E-04	5.16E-04	8.72E-04
6.08E-06	5.22E-04	1.88E-03	-2.93E-06	2.78E-04	4.49E-04
6.11E-06	3.32E-04	1.66E-03	3.13E-04	2.53E-04	4.20E-04
6.14E-06	3.46E-04	1.40E-03	-1.62E-04	1.09E-04	6.68E-04
6.18E-06	2.93E-04	1.53E-03	2.23E-04	9.10E-04	5.53E-04
6.21E-06	8.14E-04	1.59E-03	3.59E-04	7.04E-04	4.97E-04
6.24E-06	1.04E-03	1.01E-03	2.59E-04	-7.12E-05	6.00E-04
6.27E-06	3.27E-04	1.65E-03	2.70E-04	1.25E-04	3.93E-04
6.30E-06	6.98E-04	1.93E-03	-1.69E-05	2.51E-04	4.43E-04
6.34E-06	4.55E-04	1.49E-03	2.47E-04	3.81E-04	4.34E-04
6.37E-06	-1.31E-04	2.06E-03	3.44E-04	6.08E-04	4.49E-04
6.40E-06	5.04E-04	1.16E-03	4.88E-04	2.22E-04	6.18E-04
6.43E-06	6.19E-04	1.64E-03	1.37E-04	-1.33E-05	4.44E-04
6.46E-06	-1.66E-04	1.41E-03	3.71E-04	4.63E-04	4.49E-04
6.50E-06	6.26E-04	1.67E-03	5.23E-04	2.25E-04	2.40E-04
6.53E-06	1.30E-03	1.35E-03	4.49E-04	-1.34E-04	6.12E-04
6.56E-06	6.68E-05	1.43E-03	7.76E-04	1.27E-04	9.31E-05
6.59E-06	3.20E-04	1.89E-03	7.51E-04	8.08E-04	3.92E-04
6.62E-06	1.05E-04	1.62E-03	3.29E-04	4.12E-04	6.97E-04
6.66E-06	8.50E-04	1.66E-03	3.19E-04	5.72E-04	5.24E-04
6.69E-06	9.04E-04	1.45E-03	3.80E-04	3.42E-04	3.05E-04
6.72E-06	1.14E-03	1.46E-03	1.05E-04	7.26E-04	5.49E-04
6.75E-06	7.23E-04	1.60E-03	3.46E-04	1.60E-04	4.37E-04
6.78E-06	9.00E-04	1.94E-03	3.66E-05	-1.02E-04	6.68E-04
6.82E-06	7.34E-04	1.54E-03	6.16E-04	2.24E-04	5.59E-04
6.85E-06	7.81E-04	1.42E-03	7.59E-04	6.17E-04	4.68E-04
6.88E-06	7.17E-04	1.77E-03	3.61E-04	1.00E-04	5.13E-04
6.91E-06	1.23E-03	1.70E-03	2.52E-04	4.21E-04	9.36E-04
6.94E-06	7.81E-04	1.66E-03	3.20E-06	-1.36E-04	5.25E-04
6.98E-06	4.17E-04	1.45E-03	2.45E-04	5.92E-04	6.87E-04
7.01E-06	1.05E-03	1.45E-03	4.30E-04	4.79E-04	5.38E-04
7.04E-06	5.79E-04	1.40E-03	4.43E-04	3.40E-04	2.83E-04
7.07E-06	7.15E-04	1.56E-03	6.32E-04	1.00E-04	6.75E-04
7.10E-06	8.39E-04	1.54E-03	5.03E-04	4.81E-04	5.27E-04
7.14E-06	1.53E-03	1.75E-03	1.52E-04	4.35E-04	5.71E-04
7.17E-06	8.10E-04	1.72E-03	4.56E-04	-1.92E-04	2.52E-04
7.20E-06	4.01E-04	1.82E-03	2.58E-05	4.61E-04	4.10E-04
7.23E-06	1.46E-03	1.81E-03	5.28E-04	-8.50E-05	2.58E-04
7.26E-06	1.22E-03	1.81E-03	2.93E-04	-5.12E-05	4.28E-04
7.30E-06	4.49E-04	1.36E-03	4.64E-04	8.95E-05	2.51E-04
7.33E-06	1.21E-03	1.93E-03	6.26E-04	1.59E-04	-3.82E-05
7.36E-06	6.48E-04	1.88E-03	3.21E-04	8.92E-05	6.55E-04
7.39E-06	6.82E-04	1.34E-03	-8.46E-05	2.97E-04	5.23E-04
7.42E-06	8.84E-04	1.61E-03	7.61E-04	3.41E-04	5.51E-04
7.46E-06	8.43E-04	1.46E-03	3.94E-04	-5.28E-05	5.80E-04

7.49E-06	9.78E-04	1.49E-03	6.60E-04	7.40E-04	2.48E-04
7.52E-06	8.03E-04	1.89E-03	5.25E-04	6.63E-04	8.48E-04
7.55E-06	9.72E-04	1.30E-03	5.13E-04	-4.49E-05	3.13E-04
7.58E-06	5.52E-04	1.71E-03	2.80E-04	2.76E-04	5.98E-04
7.62E-06	4.36E-04	1.61E-03	5.73E-04	5.83E-04	8.72E-04
7.65E-06	7.45E-04	1.57E-03	5.25E-04	4.74E-04	6.53E-04
7.68E-06	6.16E-04	1.77E-03	1.59E-04	6.52E-05	5.19E-04
7.71E-06	4.16E-04	1.75E-03	-4.37E-05	5.71E-04	7.54E-04
7.74E-06	1.24E-04	1.68E-03	4.66E-04	4.04E-04	7.07E-04
7.78E-06	1.79E-04	2.10E-03	-3.55E-04	3.77E-04	-6.81E-05
7.81E-06	1.03E-03	1.87E-03	2.62E-04	1.33E-04	6.87E-04
7.84E-06	1.31E-03	1.29E-03	7.43E-05	2.24E-04	7.79E-04
7.87E-06	-3.79E-04	1.55E-03	1.07E-03	1.59E-04	4.78E-04
7.90E-06	-2.00E-04	1.30E-03	2.98E-04	3.53E-04	4.36E-04
7.94E-06	1.17E-04	1.52E-03	2.02E-04	5.75E-04	9.23E-04
7.97E-06	5.74E-05	1.70E-03	1.69E-04	3.94E-04	2.91E-04
8.00E-06	1.03E-04	1.57E-03	1.21E-04	3.75E-04	2.17E-04
8.03E-06	4.34E-04	1.35E-03	3.31E-04	5.69E-04	8.01E-04
8.06E-06	1.24E-03	1.44E-03	-3.57E-04	6.05E-04	8.56E-04
8.10E-06	9.08E-04	1.55E-03	-3.30E-05	6.29E-04	5.05E-04
8.13E-06	9.88E-04	1.67E-03	9.86E-04	5.56E-04	1.14E-03
8.16E-06	4.33E-04	1.25E-03	1.74E-04	2.76E-04	4.26E-04
8.19E-06	4.59E-04	1.39E-03	8.44E-04	1.38E-04	2.16E-04
8.22E-06	4.24E-04	1.41E-03	5.89E-04	3.56E-04	8.22E-04
8.26E-06	1.26E-03	1.49E-03	2.57E-04	4.42E-04	1.71E-04
8.29E-06	1.19E-03	1.62E-03	-4.10E-05	5.06E-04	3.73E-04
8.32E-06	5.73E-04	1.66E-03	6.17E-05	-4.00E-05	5.33E-04
8.35E-06	1.30E-03	2.00E-03	6.66E-04	8.47E-04	1.66E-04
8.38E-06	1.21E-03	1.49E-03	7.39E-04	9.66E-04	1.38E-04
8.42E-06	1.38E-03	1.54E-03	2.30E-04	9.16E-04	5.54E-04
8.45E-06	1.71E-04	1.48E-03	5.76E-04	1.25E-04	6.59E-04
8.48E-06	1.03E-03	1.45E-03	6.89E-04	4.14E-04	6.02E-04
8.51E-06	1.34E-03	9.46E-04	3.91E-04	2.93E-04	4.74E-04
8.54E-06	7.65E-04	1.74E-03	1.82E-04	5.21E-04	4.35E-04
8.58E-06	6.72E-04	9.90E-04	7.91E-04	4.64E-04	2.66E-04
8.61E-06	7.26E-04	1.29E-03	2.91E-04	6.60E-06	3.44E-04
8.64E-06	1.24E-03	1.20E-03	-2.48E-04	3.70E-04	2.63E-04
8.67E-06	2.39E-04	1.66E-03	6.79E-04	1.52E-05	8.78E-04
8.70E-06	1.56E-04	1.59E-03	5.19E-05	6.24E-04	6.94E-04
8.74E-06	7.95E-04	1.37E-03	5.32E-04	2.74E-04	3.94E-04
8.77E-06	6.24E-04	1.55E-03	1.44E-04	6.47E-04	9.82E-04
8.80E-06	1.92E-04	1.46E-03	3.79E-05	2.13E-04	3.73E-04
8.83E-06	1.05E-03	1.82E-03	1.14E-03	-1.52E-04	1.84E-04
8.86E-06	5.28E-04	1.48E-03	1.05E-03	1.16E-04	-4.82E-05
8.90E-06	4.57E-04	1.30E-03	1.30E-04	9.86E-04	2.41E-04
8.93E-06	-1.27E-04	1.11E-03	1.40E-04	1.91E-05	1.27E-03
8.96E-06	-2.28E-04	1.58E-03	1.02E-03	4.42E-04	8.47E-04
8.99E-06	6.26E-04	1.44E-03	8.15E-04	2.50E-04	9.76E-04
9.02E-06	7.18E-04	1.80E-03	6.30E-04	-1.54E-05	4.71E-04
9.06E-06	1.87E-04	1.51E-03	7.36E-04	2.44E-04	4.34E-04
9.09E-06	9.62E-04	9.17E-04	5.79E-04	5.44E-04	6.86E-04
9.12E-06	8.10E-04	1.52E-03	5.80E-04	-2.46E-04	1.38E-04
9.15E-06	4.11E-04	1.44E-03	5.86E-04	-9.77E-05	2.00E-04
9.18E-06	3.24E-04	1.44E-03	-2.37E-04	5.79E-04	1.12E-03

9.22E-06	9.27E-04	1.50E-03	3.76E-04	3.77E-04	4.50E-04
9.25E-06	1.10E-03	1.22E-03	5.88E-04	5.63E-04	2.40E-04
9.28E-06	6.17E-04	1.30E-03	7.45E-04	3.48E-04	6.38E-04
9.31E-06	5.76E-04	1.23E-03	2.09E-04	2.39E-04	2.02E-04
9.34E-06	7.20E-05	1.46E-03	8.17E-04	-3.44E-05	7.36E-04
9.38E-06	4.49E-04	1.33E-03	6.42E-04	5.76E-05	1.17E-03
9.41E-06	7.38E-04	1.36E-03	4.33E-04	5.65E-04	3.01E-04
9.44E-06	6.16E-04	1.52E-03	6.28E-05	1.84E-04	2.44E-04
9.47E-06	1.12E-03	1.46E-03	1.38E-03	-1.72E-04	8.06E-05
9.50E-06	9.47E-04	1.68E-03	1.32E-03	-3.63E-04	3.75E-04
9.54E-06	4.45E-04	1.73E-03	4.34E-04	2.81E-04	2.34E-04
9.57E-06	-9.46E-05	1.23E-03	1.17E-04	8.74E-05	4.59E-05
9.60E-06	6.93E-04	1.83E-03	4.06E-04	6.31E-04	1.49E-04
9.63E-06	8.26E-04	9.90E-04	6.84E-04	-4.65E-04	7.28E-04
9.66E-06	1.25E-03	1.41E-03	8.85E-04	1.01E-04	2.67E-04
9.70E-06	1.16E-03	1.44E-03	6.89E-04	3.26E-04	3.82E-04
9.73E-06	3.75E-04	1.21E-03	-2.51E-04	1.99E-04	3.32E-04
9.76E-06	1.09E-03	1.74E-03	5.91E-04	3.66E-04	1.79E-03
9.79E-06	5.41E-04	1.52E-03	8.23E-04	8.67E-05	9.82E-04
9.82E-06	4.08E-04	1.86E-03	9.36E-04	-4.64E-05	3.77E-05
9.86E-06	1.09E-03	1.74E-03	3.85E-04	-1.03E-05	6.19E-04
9.89E-06	1.61E-04	1.34E-03	9.18E-05	8.28E-04	6.84E-04
9.92E-06	1.24E-03	1.21E-03	-3.87E-04	6.44E-05	3.92E-05
9.95E-06	4.39E-04	1.98E-03	5.01E-04	2.10E-04	1.24E-03
9.98E-06	4.79E-04	4.50E-04	6.50E-04	5.97E-04	2.73E-04
1.00E-05	7.41E-04	9.25E-04	2.16E-04	-4.35E-04	8.57E-05
1.01E-05	1.06E-03	6.14E-04	5.00E-04	1.73E-04	7.38E-04
1.01E-05	4.50E-04	8.24E-04	2.01E-04	5.34E-04	1.04E-03
1.01E-05	5.14E-04	1.70E-03	3.83E-04	6.08E-04	3.21E-04
1.01E-05	5.06E-04	8.22E-04	4.61E-05	8.15E-04	-2.93E-04
1.02E-05	5.56E-04	1.45E-03	2.02E-04	-9.77E-05	-1.05E-04
1.02E-05	3.44E-04	1.30E-03	2.76E-04	2.47E-04	8.32E-04
1.02E-05	6.51E-04	9.79E-04	3.57E-04	-1.16E-04	-1.62E-04
1.03E-05	4.46E-04	1.15E-03	4.43E-04	6.51E-04	7.86E-04
1.03E-05	6.33E-04	1.47E-03	1.25E-03	1.19E-03	6.77E-04
1.03E-05	7.65E-04	1.55E-03	4.82E-04	3.03E-04	4.78E-04
1.04E-05	5.96E-04	9.60E-04	9.75E-05	8.25E-04	2.59E-04
1.04E-05	6.13E-04	1.57E-03	1.14E-03	5.45E-04	6.24E-04
1.04E-05	1.89E-04	1.37E-03	-1.16E-04	9.38E-06	1.11E-04
1.05E-05	8.23E-04	1.71E-03	-2.54E-04	9.41E-04	-6.07E-05
1.05E-05	9.80E-04	1.29E-03	6.78E-04	-2.42E-06	9.55E-04
1.05E-05	5.24E-04	9.37E-04	3.02E-04	6.10E-04	8.16E-04
1.06E-05	6.70E-04	1.67E-03	2.60E-05	8.91E-04	-2.34E-04
1.06E-05	3.83E-04	6.54E-04	5.82E-04	3.45E-04	4.10E-04
1.06E-05	5.83E-04	1.12E-03	2.46E-04	-1.39E-04	-3.21E-04
1.07E-05	3.12E-04	1.24E-03	4.76E-04	-5.07E-04	9.35E-04
1.07E-05	7.09E-04	2.29E-03	9.72E-04	2.70E-04	5.79E-05
1.07E-05	6.47E-04	2.17E-03	2.63E-04	1.89E-04	6.14E-04
1.08E-05	5.61E-04	1.21E-03	3.62E-04	-8.26E-05	1.50E-04
1.08E-05	4.30E-04	1.21E-03	4.35E-04	2.42E-06	4.70E-04
1.08E-05	4.68E-04	1.07E-03	3.16E-04	6.68E-04	8.78E-04
1.09E-05	5.76E-04	1.50E-03	2.66E-04	4.37E-04	6.42E-04
1.09E-05	2.89E-04	1.69E-03	7.55E-05	2.44E-04	1.12E-03
1.09E-05	4.89E-04	6.08E-04	1.64E-04	-2.23E-04	2.01E-04

1.09E-05	4.00E-04	5.81E-04	7.79E-04	5.62E-05	3.69E-04
1.10E-05	5.07E-04	9.09E-04	6.00E-04	1.49E-04	8.06E-04
1.10E-05	4.55E-04	8.19E-04	4.52E-04	6.84E-04	-1.37E-04
1.10E-05	5.15E-04	1.70E-03	2.77E-04	2.44E-04	4.86E-04
1.11E-05	1.48E-04	6.48E-04	2.33E-04	-7.74E-05	3.63E-04
1.11E-05	3.95E-04	1.09E-03	5.98E-04	7.40E-05	4.65E-04
1.11E-05	6.01E-04	1.20E-03	5.55E-04	3.32E-04	1.28E-04
1.12E-05	4.04E-04	6.04E-04	4.61E-04	2.17E-04	2.86E-04
1.12E-05	9.56E-04	1.11E-03	6.09E-04	8.43E-04	2.02E-04
1.12E-05	4.60E-04	1.21E-03	5.27E-04	-5.28E-05	2.63E-04
1.13E-05	3.93E-04	1.37E-03	-3.68E-04	1.37E-04	9.16E-04
1.13E-05	3.80E-04	1.54E-03	9.85E-04	5.09E-04	5.17E-04
1.13E-05	3.52E-04	9.05E-04	4.25E-04	4.99E-04	3.39E-04
1.14E-05	2.94E-04	1.70E-03	1.90E-04	1.50E-04	2.15E-04
1.14E-05	7.10E-04	1.36E-03	3.39E-04	3.20E-04	6.89E-04
1.14E-05	5.84E-04	9.02E-04	9.24E-04	7.47E-04	6.08E-04
1.15E-05	4.41E-04	1.13E-03	1.17E-03	-1.68E-04	3.65E-04
1.15E-05	3.55E-04	1.31E-03	8.04E-04	-1.63E-06	8.51E-04
1.15E-05	5.75E-04	1.34E-03	1.71E-04	4.54E-05	6.65E-04
1.16E-05	7.96E-04	8.50E-04	3.18E-04	1.73E-04	2.98E-04
1.16E-05	5.70E-04	8.18E-04	2.98E-04	2.71E-04	9.66E-04
1.16E-05	5.89E-04	1.61E-03	7.66E-04	-3.08E-04	7.14E-04
1.17E-05	7.87E-04	8.94E-04	4.63E-04	-2.27E-04	1.36E-03
1.17E-05	6.38E-04	5.72E-04	1.77E-04	-1.77E-04	3.27E-04
1.17E-05	3.90E-04	1.30E-03	7.48E-04	2.11E-04	1.09E-03
1.17E-05	3.86E-04	1.51E-03	3.02E-04	-1.53E-04	6.99E-04
1.18E-05	5.01E-04	1.85E-03	5.29E-04	-5.18E-05	9.93E-04
1.18E-05	4.48E-04	1.01E-03	2.17E-04	-4.63E-06	1.16E-03
1.18E-05	6.02E-04	7.01E-04	1.61E-04	-1.35E-04	1.08E-03
1.19E-05	1.60E-04	1.41E-03	6.48E-05	4.49E-05	9.40E-04
1.19E-05	5.42E-04	9.33E-04	5.03E-04	1.43E-04	1.25E-04
1.19E-05	4.16E-04	1.74E-03	4.08E-04	7.94E-05	-3.25E-05
1.20E-05	6.16E-04	6.31E-04	3.89E-04	-2.25E-04	5.39E-04
1.20E-05	4.91E-04	8.29E-04	-8.22E-05	-7.46E-05	1.95E-04
1.20E-05	4.17E-04	1.35E-03	9.08E-05	2.52E-04	9.21E-04
1.21E-05	5.06E-04	1.29E-03	8.04E-04	-1.39E-04	4.92E-04
1.21E-05	4.96E-04	1.82E-03	6.32E-04	1.37E-04	8.11E-04
1.21E-05	4.73E-04	6.42E-04	1.03E-04	-1.54E-04	2.32E-04
1.22E-05	9.27E-05	1.88E-03	2.44E-04	1.29E-04	1.22E-03
1.22E-05	7.03E-04	1.21E-03	3.15E-04	4.14E-04	1.30E-04
1.22E-05	2.97E-04	4.38E-04	5.99E-04	4.11E-04	5.01E-04
1.23E-05	3.62E-04	1.39E-03	2.82E-05	-8.88E-05	7.92E-04
1.23E-05	4.95E-04	1.32E-03	2.71E-04	-1.13E-04	1.40E-03
1.23E-05	1.59E-04	1.11E-03	4.92E-04	2.19E-04	7.22E-04
1.24E-05	3.54E-04	1.65E-03	7.03E-04	8.02E-06	2.97E-04
1.24E-05	2.75E-04	4.52E-04	2.33E-04	3.18E-04	3.64E-04
1.24E-05	4.83E-04	1.04E-03	2.71E-04	3.53E-04	-1.81E-04
1.25E-05	4.02E-04	9.42E-04	-3.25E-06	-6.29E-05	6.31E-04
1.25E-05	3.66E-04	1.07E-03	2.07E-04	-1.17E-04	9.50E-04
1.25E-05	3.23E-04	5.51E-04	9.91E-05	2.98E-04	8.90E-04
1.25E-05	3.81E-04	1.93E-03	6.69E-04	-1.19E-04	8.69E-04
1.26E-05	4.39E-04	1.47E-03	1.80E-04	4.67E-04	7.97E-04
1.26E-05	5.61E-04	1.35E-03	3.12E-04	7.10E-05	6.41E-04
1.26E-05	1.63E-04	1.06E-03	4.74E-04	-2.20E-04	3.02E-04

1.27E-05	3.63E-04	9.98E-04	-3.27E-04	-1.91E-04	-2.26E-05
1.27E-05	3.98E-04	1.18E-03	3.35E-04	9.14E-05	5.11E-04
1.27E-05	5.63E-04	1.10E-03	5.60E-04	1.31E-05	5.13E-04
1.28E-05	1.21E-04	1.08E-03	2.56E-04	5.15E-04	8.24E-04
1.28E-05	4.40E-04	9.59E-04	5.42E-04	3.52E-04	1.24E-04
1.28E-05	5.91E-04	1.07E-03	5.30E-04	1.86E-04	6.18E-04
1.29E-05	6.28E-04	1.12E-03	3.22E-04	-2.64E-05	1.10E-03
1.29E-05	5.40E-04	8.11E-04	4.59E-04	5.53E-04	9.69E-05
1.29E-05	5.14E-04	1.12E-03	1.10E-04	-1.09E-04	3.99E-04
1.30E-05	3.97E-04	6.90E-04	4.23E-04	-2.66E-04	6.25E-04
1.30E-05	5.98E-04	1.76E-03	2.34E-04	2.16E-04	1.17E-03
1.30E-05	2.16E-04	1.36E-03	5.17E-04	-3.61E-04	7.16E-04
1.31E-05	6.03E-04	7.75E-04	2.56E-04	1.02E-04	7.24E-04
1.31E-05	4.29E-04	1.87E-03	3.06E-04	-1.16E-04	5.10E-04
1.31E-05	8.03E-04	1.17E-03	5.46E-04	4.13E-05	3.36E-04
1.32E-05	4.64E-04	7.42E-04	5.97E-04	-1.87E-04	3.64E-04
1.32E-05	7.10E-04	1.29E-03	1.96E-04	1.37E-04	5.50E-04
1.32E-05	5.29E-04	1.39E-03	2.30E-04	3.22E-05	1.10E-03
1.33E-05	4.93E-04	1.53E-03	-2.03E-04	-9.70E-05	8.03E-04
1.33E-05	8.86E-05	7.46E-04	1.16E-03	-3.26E-04	1.14E-03
1.33E-05	4.80E-04	1.29E-03	9.00E-05	1.04E-04	8.65E-04
1.33E-05	1.58E-04	1.18E-03	3.38E-04	-9.47E-05	6.65E-04
1.34E-05	3.87E-04	1.03E-03	1.04E-04	-1.48E-04	7.99E-04
1.34E-05	5.24E-04	1.21E-03	6.45E-04	-1.10E-04	6.40E-04
1.34E-05	3.73E-04	5.51E-04	2.76E-04	-3.00E-04	3.48E-04
1.35E-05	3.95E-04	9.70E-04	3.18E-04	6.97E-06	1.04E-03
1.35E-05	2.79E-04	9.42E-04	5.18E-04	1.37E-04	1.15E-03
1.35E-05	2.56E-04	1.33E-03	2.25E-04	-4.22E-04	1.15E-03
1.36E-05	4.18E-04	7.35E-04	3.81E-04	-2.33E-04	1.36E-04
1.36E-05	2.74E-04	9.52E-04	1.57E-04	2.83E-04	3.93E-04
1.36E-05	-1.76E-05	9.83E-04	5.25E-05	-2.11E-04	4.06E-04
1.37E-05	2.40E-04	1.23E-03	2.97E-04	-1.43E-04	1.11E-03
1.37E-05	2.82E-04	1.70E-03	7.70E-05	-4.42E-05	4.46E-04
1.37E-05	5.80E-04	1.01E-03	1.01E-04	-2.10E-04	5.70E-04
1.38E-05	7.45E-04	1.38E-03	2.37E-04	-1.60E-04	6.99E-04
1.38E-05	7.33E-04	1.62E-03	3.66E-05	-9.96E-05	5.23E-04
1.38E-05	-1.10E-04	1.34E-03	6.06E-04	1.15E-04	-3.93E-05
1.39E-05	3.77E-04	8.38E-04	2.56E-04	1.41E-04	8.32E-04
1.39E-05	4.69E-04	1.22E-03	1.19E-04	-3.01E-04	4.68E-04
1.39E-05	1.07E-04	1.21E-03	2.05E-04	1.22E-04	6.78E-04
1.40E-05	4.18E-04	1.15E-03	1.94E-04	-3.51E-04	4.21E-04
1.40E-05	6.25E-04	1.20E-03	1.40E-04	-1.18E-04	6.35E-04
1.40E-05	5.43E-04	9.79E-04	1.89E-04	8.25E-05	4.85E-04
1.41E-05	5.12E-04	8.34E-04	-1.40E-04	2.00E-04	2.38E-05
1.41E-05	2.03E-04	9.52E-04	3.50E-05	-2.31E-04	1.63E-04
1.41E-05	3.27E-04	1.36E-03	2.39E-04	3.57E-04	7.74E-04
1.41E-05	5.10E-05	1.27E-03	1.25E-04	-1.37E-04	6.31E-04
1.42E-05	2.36E-04	7.37E-04	4.69E-05	3.73E-04	1.05E-03
1.42E-05	3.23E-04	1.19E-03	2.45E-04	-1.63E-04	8.60E-04
1.42E-05	2.75E-04	1.10E-03	2.94E-04	4.30E-05	6.39E-04
1.43E-05	1.45E-04	9.28E-04	2.66E-04	-2.44E-05	4.08E-04
1.43E-05	-4.74E-05	1.10E-03	1.09E-04	-2.82E-04	7.93E-04
1.43E-05	1.55E-04	1.02E-03	3.25E-05	-5.30E-05	-1.35E-04
1.44E-05	5.53E-04	8.40E-04	-1.63E-05	6.99E-05	8.09E-04

1.44E-05	1.38E-04	1.10E-03	2.69E-04	-1.60E-04	5.58E-04
1.44E-05	5.49E-04	8.96E-04	1.78E-04	-1.21E-04	6.02E-04
1.45E-05	4.26E-04	1.31E-03	4.23E-04	-7.99E-05	6.11E-04
1.45E-05	3.10E-04	1.04E-03	-9.14E-06	-2.86E-04	4.40E-04
1.45E-05	3.83E-04	1.07E-03	6.54E-04	-4.52E-04	3.82E-04
1.46E-05	4.41E-04	1.21E-03	3.03E-04	1.03E-04	5.30E-04
1.46E-05	3.32E-04	1.35E-03	3.63E-04	6.34E-06	-1.43E-04
1.46E-05	3.66E-04	1.40E-03	6.49E-04	-2.29E-05	4.48E-04
1.47E-05	3.70E-04	1.63E-03	7.44E-04	-5.24E-05	5.76E-04
1.47E-05	3.06E-05	8.43E-04	7.12E-04	-1.98E-04	6.30E-04
1.47E-05	5.98E-04	1.01E-03	5.37E-04	2.98E-04	4.30E-04
1.48E-05	5.54E-04	1.21E-03	1.95E-04	-9.82E-05	5.35E-04
1.48E-05	1.51E-04	1.11E-03	1.64E-04	1.40E-04	4.86E-04
1.48E-05	-4.32E-05	9.43E-04	1.49E-04	2.32E-04	9.56E-04
1.49E-05	3.07E-04	8.60E-04	1.67E-05	-4.85E-04	7.72E-04
1.49E-05	7.91E-04	1.04E-03	6.86E-05	-9.47E-05	4.57E-04
1.49E-05	3.24E-04	1.17E-03	7.67E-04	-1.16E-04	5.35E-04
1.49E-05	4.96E-04	1.11E-03	-1.79E-04	-1.40E-04	7.05E-04
1.50E-05	1.88E-04	8.14E-04	9.39E-05	-2.18E-04	3.01E-04

Table SI.16: Primary kinetic data for the reaction of 1,1,3,3-tetramethylurea (**S16**) with CumO• generated by 355 nm LFP in MeCN (**Figure SI.16**).

t / s	ΔA 1,1,3,3-tetramethylurea (S16)				
	0.0332M	0.0988M	0.163M	0.227M	0.290M
-1E-06	0.00028	-0.0005	-4E-05	2.8E-05	-5E-06
-1E-06	0.00077	0.00024	-0.0002	-0.0001	0.00016
-9E-07	0.00101	0.00026	-0.0005	-0.0007	0.00124
-9E-07	0.00089	-1E-05	-0.0006	-0.0007	-4E-05
-9E-07	0.00153	0.00044	-0.0005	-0.0004	0.00109
-8E-07	0.00045	0.00053	-0.0001	0.00037	0.00087
-8E-07	0.00011	-2E-05	-9E-05	0.00064	0.00084
-8E-07	0.00037	-0.0001	0.00038	-0.0012	9.2E-05
-7E-07	0.00094	0.00066	-0.0001	-0.0009	0.00083
-7E-07	0.00096	0.00028	-0.0003	-0.0002	0.00034
-7E-07	0.00036	-0.0002	-0.0009	8.2E-05	0.00058
-6E-07	0.00077	0.00036	-0.0008	-0.0006	0.00103
-6E-07	0.00094	-0.0004	-0.0007	-0.0006	0.00029
-6E-07	0.00054	-7E-05	-0.0005	-0.0003	0.00026
-5E-07	0.00068	-0.0005	-0.0001	0.00024	0.00028
-5E-07	0.00074	9.3E-05	-0.0003	0.0003	0.00103
-5E-07	0.00067	-7E-05	3.8E-05	-0.0006	0.00115
-4E-07	0.00057	-0.0002	0.00012	-8E-05	0.00105
-4E-07	0.00055	4.6E-05	0.00035	-0.0007	0.00135
-4E-07	0.00091	0.00025	0.00029	-4E-05	0.00031
-4E-07	0.00069	-0.0002	-1E-05	0.00088	0.00014
-3E-07	0.0008	-6E-05	-0.0008	-0.0003	0.00053
-3E-07	0.00119	0.00016	-5E-05	-0.0002	0.00051
-3E-07	0.00144	0.00089	-0.0009	-0.001	0.00047
-2E-07	0.00141	-0.0005	-0.0002	-0.0005	0.00053
-2E-07	0.00102	0.00019	0.00013	-0.0006	0.00072
-2E-07	0.00141	-0.0002	0.00026	-0.0004	0.00076
-1E-07	0.00057	0.00041	8E-05	0.00046	0.00099
-1E-07	0.00063	-0.0002	-9E-06	0.00031	0.00094
-6E-08	7.9E-05	-0.0007	3.4E-05	-0.0004	0.00021
-3E-08	0.00012	-0.0004	-0.0003	0.00022	0.0011
-1E-10	0.00036	-0.0009	-0.0013	-0.0009	-0.0006
3.2E-08	0.00609	-0.0023	-0.0088	-0.0132	-0.0151
6.4E-08	0.01641	0.01424	0.01426	0.01219	0.01352
9.6E-08	0.01666	0.0145	0.0146	0.01295	0.01324
1.3E-07	0.01627	0.0142	0.0131	0.01251	0.01359
1.6E-07	0.01579	0.01359	0.01258	0.01197	0.01281
1.9E-07	0.0158	0.01286	0.0124	0.01062	0.01198
2.2E-07	0.01459	0.01262	0.01169	0.00993	0.01136
2.6E-07	0.0139	0.01144	0.01068	0.00907	0.0102
2.9E-07	0.01377	0.01123	0.01041	0.00855	0.00953
3.2E-07	0.01388	0.01106	0.01004	0.00777	0.00929
3.5E-07	0.01307	0.00976	0.00958	0.00764	0.00802
3.8E-07	0.01229	0.00961	0.0094	0.00775	0.00756
4.2E-07	0.0119	0.00957	0.00819	0.00661	0.00648
4.5E-07	0.01219	0.00899	0.00757	0.00656	0.00654
4.8E-07	0.01168	0.00915	0.00711	0.00567	0.00703
5.1E-07	0.01161	0.00884	0.00728	0.00626	0.00566
5.4E-07	0.01117	0.0078	0.00608	0.00481	0.00529

5.8E-07	0.01043	0.00739	0.00634	0.00434	0.00557
6.1E-07	0.01093	0.00797	0.00554	0.00421	0.00466
6.4E-07	0.01011	0.00735	0.0065	0.00329	0.00443
6.7E-07	0.00995	0.00668	0.0047	0.00342	0.00394
7E-07	0.01068	0.00695	0.00441	0.00269	0.00386
7.4E-07	0.0093	0.00609	0.00448	0.0038	0.00357
7.7E-07	0.00921	0.00582	0.00437	0.00287	0.00266
8E-07	0.00882	0.00516	0.00339	0.00232	0.00349
8.3E-07	0.00844	0.00573	0.00375	0.00308	0.00293
8.6E-07	0.00858	0.00528	0.00326	0.00193	0.00267
9E-07	0.00861	0.00539	0.00347	0.00228	0.00233
9.3E-07	0.00837	0.00433	0.00276	0.00227	0.00282
9.6E-07	0.00862	0.00503	0.00325	0.00129	0.0028
9.9E-07	0.00772	0.00479	0.00227	0.00208	0.00176
1E-06	0.00715	0.00437	0.00273	0.00223	0.00205
1.1E-06	0.00737	0.00428	0.00193	0.00127	0.00232
1.1E-06	0.00689	0.00351	0.002	0.00051	0.00179
1.1E-06	0.00646	0.00339	0.00236	0.00146	0.00211
1.2E-06	0.00711	0.00326	0.00129	0.00123	0.00184
1.2E-06	0.00665	0.00319	0.00211	0.00111	0.00145
1.2E-06	0.00713	0.00329	0.00162	0.00128	0.00167
1.2E-06	0.00574	0.00302	0.00148	0.00105	0.00196
1.3E-06	0.0061	0.00336	0.00172	0.00163	0.00148
1.3E-06	0.00563	0.00267	0.00112	0.00108	0.00135
1.3E-06	0.00544	0.00297	0.00139	0.00089	0.00146
1.4E-06	0.00547	0.0023	0.00112	0.00049	0.00114
1.4E-06	0.00512	0.00271	0.0013	0.00119	0.00177
1.4E-06	0.0053	0.0027	0.00085	0.00147	0.00166
1.5E-06	0.00534	0.00193	0.00126	-0.0001	0.00143
1.5E-06	0.00516	0.00244	0.00082	0.00018	0.00191
1.5E-06	0.0048	0.0028	0.00084	-0.0003	0.00082
1.6E-06	0.00531	0.00287	0.00089	0.00061	0.00112
1.6E-06	0.00524	0.00176	0.00099	0.00127	0.00173
1.6E-06	0.00437	0.00155	0.00048	0.00015	0.001
1.7E-06	0.00418	0.00167	0.00041	0.00029	0.00148
1.7E-06	0.00458	0.00165	0.00054	0.00019	0.00094
1.7E-06	0.00501	0.00254	0.00082	0.00056	0.00051
1.8E-06	0.00413	0.00181	-0.0001	0.00096	0.0013
1.8E-06	0.00409	0.00176	3.5E-05	-0.0005	0.00098
1.8E-06	0.00346	0.00212	5.3E-05	0.00045	0.00073
1.9E-06	0.00381	0.00193	0.0005	0.00012	0.00121
1.9E-06	0.00384	0.00122	0.00075	0.00051	0.00066
1.9E-06	0.00321	0.00148	0.00067	1.8E-05	-0.0005
2E-06	0.00392	0.0009	4.5E-05	-0.0001	0.00082
2E-06	0.00343	0.00101	0.00028	0.00031	0.00173
2E-06	0.00286	0.00114	0.00047	-5E-05	0.00061
2E-06	0.00296	0.00139	0.00086	-0.0001	0.00112
2.1E-06	0.00362	0.00066	0.00011	0.00037	0.00043
2.1E-06	0.00357	0.00089	-0.0003	-6E-06	0.00101
2.1E-06	0.00357	0.00084	-0.0004	0.00093	0.00018
2.2E-06	0.00313	0.00094	0.00043	-0.0003	0.00083
2.2E-06	0.00296	0.00067	0.00065	-0.0005	0.00129
2.2E-06	0.00287	0.00138	-0.0004	8E-06	0.00173
2.3E-06	0.00252	0.00094	-0.0001	1.7E-05	0.00116

2.3E-06	0.00245	0.00095	-0.0003	-0.0001	0.0006
2.3E-06	0.00298	0.0018	0.00015	0.00024	0.00047
2.4E-06	0.00307	0.00109	0.00026	0.00061	0.00122
2.4E-06	0.00206	4E-05	-0.0001	-0.0001	0.00039
2.4E-06	0.00213	0.0004	-0.0002	-0.0004	0.00047
2.5E-06	0.0025	4.7E-05	0.00016	-0.0002	0.00114
2.5E-06	0.00289	0.00132	-0.0001	-0.0009	0.00043
2.5E-06	0.00197	0.001	3.8E-05	-0.0006	0.0016
2.6E-06	0.00198	0.00058	-0.0001	0.00022	0.00122
2.6E-06	0.00231	3.1E-05	0.00037	0.00059	0.00034
2.6E-06	0.0025	0.00039	-0.0003	-0.001	0.00066
2.7E-06	0.00131	3.1E-05	-0.0004	-0.0009	0.00022
2.7E-06	0.00204	0.00055	-0.001	-0.0002	0.00102
2.7E-06	0.00211	0.00072	-0.0005	0.00021	0.00099
2.8E-06	0.00234	0.00054	1.9E-05	-0.0005	0.00035
2.8E-06	0.00211	0.0007	-0.0003	0.00045	0.00106
2.8E-06	0.00204	0.00024	-7E-05	-0.0009	0.00082
2.8E-06	0.00216	0.00088	-0.0003	-0.0006	0.00106
2.9E-06	0.00119	0.00057	-0.0004	-0.0004	0.00069
2.9E-06	0.00104	0.00079	-1E-04	-0.0006	0.00052
2.9E-06	0.00195	-0.0001	3.2E-06	5.9E-05	0.0007
3E-06	0.00123	0.00045	-0.0006	-0.0009	0.00128
3E-06	0.00182	0.00042	-0.0002	-0.0002	0.00064
3E-06	0.00212	0.00026	8.6E-06	-0.0007	0.00058
3.1E-06	0.0016	0.00071	-0.0001	-0.0001	0.00066
3.1E-06	0.00215	0.00085	-0.0008	6.8E-05	0.00059
3.1E-06	0.00168	0.00026	0.00038	0.00035	0.0006
3.2E-06	0.00193	-4E-05	0.00054	-0.0012	0.00069
3.2E-06	0.00202	0.00046	0.00013	-0.0012	0.00083
3.2E-06	0.00183	0.00014	-0.0002	-0.0003	0.00064
3.3E-06	0.00184	5.7E-05	0.00042	0.00017	0.00041
3.3E-06	0.00174	-0.0003	-0.0008	0.00017	0.00049
3.3E-06	0.00144	9.7E-05	-0.0004	-0.0004	4.1E-05
3.4E-06	0.00195	7E-06	-0.0007	0.0003	0.00063
3.4E-06	0.00218	0.00039	-0.0008	-0.0002	6.9E-05
3.4E-06	0.00217	0.00024	-8E-05	-0.0006	0.00033
3.5E-06	0.00162	0.00011	-0.0007	-0.0005	0.00044
3.5E-06	0.00136	-1E-04	-9E-06	-0.0006	0.0007
3.5E-06	0.00096	0.00065	-0.0002	0.00052	0.0008
3.6E-06	0.00095	0.00024	1.6E-05	0.00021	0.00091
3.6E-06	0.00115	0.0005	6.3E-05	0.00016	0.00115
3.6E-06	0.00156	-0.0003	0.00017	-0.0006	0.00079
3.6E-06	0.00183	-0.0006	-5E-05	-0.0002	0.00118
3.7E-06	0.00174	1.7E-05	-0.0004	9.7E-05	0.00066
3.7E-06	0.0015	0.0002	-0.0007	-9E-05	5.8E-05
3.7E-06	0.00189	-0.0003	-0.0003	0.00039	0.001
3.8E-06	0.00069	-0.0003	-0.0007	1.3E-05	0.00076
3.8E-06	0.00107	0.00028	-0.001	-0.0006	0.00091
3.8E-06	0.00138	0.0007	-3E-05	0.0004	0.00048
3.9E-06	0.00139	0.00045	-0.0007	0.00019	0.00121
3.9E-06	0.00159	0.00065	-0.0008	-0.0002	0.00077
3.9E-06	0.00067	8.3E-05	-0.0009	-9E-05	0.001
4E-06	0.0014	-7E-05	-0.0005	-0.0006	0.00144
4E-06	0.00141	-2E-05	-0.0008	-0.0007	0.00125

4E-06	0.00105	-0.0005	-0.0008	-0.0002	0.00123
4.1E-06	0.00128	0.00023	-0.001	-0.0002	0.00062
4.1E-06	0.00151	0.0007	-0.0004	-0.0005	0.00047
4.1E-06	0.00135	6.1E-05	-0.0008	0.0002	0.00049
4.2E-06	0.00079	7.1E-05	-0.0008	-0.0002	0.00093
4.2E-06	0.00119	0.00034	-0.0003	0.00061	3.6E-05
4.2E-06	0.00026	0.00017	-0.0008	0.0004	0.001
4.3E-06	0.00089	0.00035	-0.0011	0.00022	4.6E-05
4.3E-06	0.00099	0.00014	-0.0003	-0.0007	0.00051
4.3E-06	0.00104	0.00051	-0.0001	0.00027	0.00069
4.4E-06	0.00098	-0.0004	0.00017	-0.0012	0.00012
4.4E-06	0.00143	0.00027	-1E-05	-0.0004	0.00136
4.4E-06	6.4E-05	6.6E-05	-0.0004	0.00026	0.00052
4.4E-06	0.00092	9.9E-05	-0.0002	-0.0002	0.00085
4.5E-06	0.00033	-2E-06	-0.0002	-0.0005	0.00089
4.5E-06	0.00071	0.00134	-0.0004	-0.0001	0.00015
4.5E-06	0.00066	-0.0004	0.00064	-0.0002	0.00132
4.6E-06	0.00094	-0.0002	0.00043	-0.0002	0.00073
4.6E-06	0.0004	0.00077	-0.0001	0.00029	0.00031
4.6E-06	0.00099	-0.0002	-0.0004	-0.0008	0.0006
4.7E-06	0.00129	-0.0002	-0.0004	-0.0004	0.00056
4.7E-06	0.00154	-0.0002	-0.0001	-0.0003	0.00108
4.7E-06	0.00074	0.00031	-0.0005	2.4E-05	0.0009
4.8E-06	0.00103	0.00073	-1E-04	-0.0005	0.00104
4.8E-06	0.00116	-2E-05	0.00017	0.00045	0.00103
4.8E-06	0.00145	-0.0006	-0.0009	-0.0006	0.0009
4.9E-06	0.00095	-0.0005	-0.0008	-0.0002	0.00088
4.9E-06	0.00082	-6E-05	-0.0001	8.6E-07	0.00067
4.9E-06	0.00063	0.00076	-0.0004	-0.0004	0.00063
5E-06	0.00116	0.00023	-0.0005	-0.0005	0.00066
5E-06	0.00092	-0.0003	4.5E-05	-0.0001	0.00067
5E-06	0.00119	-0.0007	-0.0003	-0.0007	0.00055
5.1E-06	0.00081	0.00045	1.5E-05	-0.0005	0.00053
5.1E-06	0.00085	0.00055	-0.0006	-0.0004	0.00108
5.1E-06	0.00159	0.00014	-7E-05	0.00033	0.00059
5.2E-06	0.00113	-0.0001	-0.0006	-0.0002	0.00187
5.2E-06	0.00045	0.0007	-0.0008	-0.0006	0.00132
5.2E-06	0.00134	7.5E-05	-0.0004	9.4E-05	0.00073
5.2E-06	0.00085	-0.0004	-0.0006	-0.0005	0.00016
5.3E-06	9.5E-05	4.4E-05	-0.0003	-0.0005	0.00143
5.3E-06	0.00082	0.00057	0.00029	4.8E-05	0.00106
5.3E-06	0.00062	-7E-05	-0.0012	-1E-04	0.00026
5.4E-06	0.00034	-0.0005	-7E-05	-0.0005	0.00059
5.4E-06	7.1E-05	3.1E-05	-0.0008	-0.0002	0.00094
5.4E-06	0.0007	0.00077	-0.0001	-0.0009	0.0009
5.5E-06	0.00046	-3E-05	0.0002	-0.0005	0.0003
5.5E-06	0.0009	-4E-05	-0.0006	-0.0004	0.00168
5.5E-06	0.00015	1.7E-05	-0.0004	-0.0003	0.00046
5.6E-06	0.00025	-0.0004	-0.001	-0.0001	0.00098
5.6E-06	7.1E-05	-0.0002	-0.0009	0.00073	0.00055
5.6E-06	-0.0003	0.00036	-0.0004	-0.0004	0.00126
5.7E-06	0.00069	-0.0005	-0.0008	-0.0004	0.00076
5.7E-06	0.00095	0.00039	-0.0002	-0.0006	0.00095
5.7E-06	0.00034	-0.0003	-0.0006	-0.0008	0.001

5.8E-06	0.0008	-3E-06	-0.0007	-0.0005	0.00093
5.8E-06	0.00095	3.8E-05	-0.0003	8.6E-05	0.00107
5.8E-06	-0.0002	0.00046	-0.0003	-0.0002	1.7E-05
5.9E-06	0.00086	-2E-05	-0.0004	7.1E-05	0.00066
5.9E-06	0.00037	8E-05	-0.0002	0.00027	8.8E-05
5.9E-06	0.00087	-0.0003	-0.0002	0.00012	0.00056
6E-06	0.00057	0.00035	-0.001	-0.0004	0.00084
6E-06	0.00075	0.00056	-0.0007	-8E-05	0.0015
6E-06	0.00073	0.00022	-0.0009	-0.0002	0.00073
6E-06	0.00035	-2E-05	-0.0007	-0.0002	0.00084
6.1E-06	0.00025	7.2E-05	-0.001	-0.0007	0.00083
6.1E-06	0.00045	4.5E-05	-0.0002	-0.0008	0.00128
6.1E-06	0.00064	-0.0001	-0.0004	-0.0009	0.00034
6.2E-06	-0.0005	0.00014	0.00026	-0.0008	0.00047
6.2E-06	0.00056	-1E-04	-0.0002	-0.0001	0.00018
6.2E-06	0.00081	0.00077	-0.0008	0.00017	0.00066
6.3E-06	0.00065	0.00016	0.00026	0.00028	0.00048
6.3E-06	0.00025	-3E-05	-0.001	0.00017	0.00021
6.3E-06	0.00053	-0.0002	-0.0001	-0.0002	0.0012
6.4E-06	0.00034	-0.0002	-0.0005	-0.001	0.00075
6.4E-06	0.00047	0.00066	-0.001	-0.0006	0.00074
6.4E-06	-0.0002	0.00068	-0.0007	1.7E-05	2.8E-05
6.5E-06	0.00048	-6E-05	-0.0002	-0.0005	0.00046
6.5E-06	0.00094	0.00029	-0.0006	-0.0003	0.00033
6.5E-06	0.00127	-0.0001	-0.0003	-0.0005	0.00012
6.6E-06	0.00032	-0.0003	-0.0003	-0.001	0.00131
6.6E-06	0.00046	-0.0002	-3E-05	0.00011	0.00118
6.6E-06	0.00082	-0.0001	-0.0003	-0.0005	0.0004
6.7E-06	-0.0001	0.0006	-0.0005	-0.0004	0.00113
6.7E-06	0.00075	3.1E-05	-0.0001	-0.0004	0.00104
6.7E-06	0.00068	-0.0004	-0.0001	-0.0006	0.00056
6.8E-06	0.00037	0.00047	-0.0007	-0.0007	0.00035
6.8E-06	0.00033	2.9E-06	-0.0003	-0.0002	0.00062
6.8E-06	8.6E-05	0.00011	-0.0002	0.0001	0.00089
6.8E-06	0.00112	0.00048	-0.0007	-8E-05	-0.0002
6.9E-06	0.00097	4.3E-05	-0.0006	-0.0008	0.00058
6.9E-06	0.00012	-0.0004	-0.0002	-0.0004	0.00083
6.9E-06	0.00043	-0.0003	-0.0006	-0.0005	0.00091
7E-06	0.00076	-0.0003	-0.0006	0.00021	0.00065
7E-06	-0.0005	0.00013	-0.0007	-0.0001	0.00017
7E-06	0.00049	-0.001	0.00019	-0.001	0.00084
7.1E-06	0.00045	7.8E-06	-0.0007	-0.0007	0.00067
7.1E-06	0.00031	0.00041	-0.0007	8.6E-06	0.00097
7.1E-06	0.00076	0.00048	-0.0004	-0.0009	0.00063
7.2E-06	0.00034	-0.0003	-0.0004	-0.0004	0.00045
7.2E-06	8.3E-05	0.0008	-4E-05	0.00049	0.00089
7.2E-06	0.00051	0.00045	-0.0002	-0.0002	0.0009
7.3E-06	0.00065	0.00034	-0.0011	-0.0004	0.00074
7.3E-06	0.00092	0.00017	-0.0004	-0.0003	0.00032
7.3E-06	0.00019	0.00063	6.4E-05	-0.0004	0.00083
7.4E-06	0.00113	4.8E-05	0.00018	-0.0007	0.00076
7.4E-06	0.00038	6.8E-05	-0.0002	-0.0005	0.00094
7.4E-06	0.00089	-0.0005	0.00022	-9E-05	0.00089
7.5E-06	0.001	0.00016	-0.0004	0.00014	0.00081

7.5E-06	0.00048	0.0002	3.5E-05	0.00035	0.00183
7.5E-06	0.00056	-9E-05	-0.0004	7E-05	0.00095
7.6E-06	0.00054	0.00056	-0.0006	-8E-05	0.00114
7.6E-06	0.00022	-1E-04	-0.0009	0.00025	0.00086
7.6E-06	0.00074	-0.0003	-0.0004	0.00024	0.00071
7.6E-06	0.00045	0.00023	-0.0005	-9E-05	0.00014
7.7E-06	0.0004	-6E-05	0.00022	-0.0003	0.00093
7.7E-06	0.00028	9.7E-05	-0.0007	9.1E-05	0.00037
7.7E-06	-0.0003	-0.0004	-0.0004	0.00042	0.00074
7.8E-06	0.001	-0.0001	-0.0005	-0.0004	0.0013
7.8E-06	0.0004	9.9E-05	-0.0001	-0.0001	0.00084
7.8E-06	0.00041	7.1E-05	-0.0003	0.00022	-5E-05
7.9E-06	0.0006	-0.0005	-0.0005	-1E-04	0.0006
7.9E-06	0.00091	0.00034	-0.001	-0.0008	0.00064
7.9E-06	0.00071	0.00047	5.7E-05	-0.0008	0.00018
8E-06	0.0007	0.00033	-0.0003	0.00033	0.00093
8E-06	0.00107	0.00043	-0.0002	7.6E-05	0.00044
8E-06	0.00034	7.9E-05	-0.0005	-8E-05	0.00068
8.1E-06	0.00068	0.00031	-0.0005	0.00018	0.00048
8.1E-06	0.00033	-0.0005	-0.0002	-0.0006	0.00079
8.1E-06	0.00089	0.00063	0.00048	3.3E-05	0.00091
8.2E-06	0.00016	0.00011	-0.0001	-0.0005	0.00033
8.2E-06	0.00046	0.00013	0.00026	-0.0007	0.00078
8.2E-06	0.0006	0.00063	-0.0008	0.00073	0.00076
8.3E-06	0.00028	-0.0001	-0.0002	-0.0008	0.00051
8.3E-06	0.0003	2.8E-05	-3E-05	-0.0012	0.00074
8.3E-06	0.00094	-6E-05	-0.0006	-0.0002	0.00129
8.4E-06	0.00118	-9E-05	-0.0005	-0.0005	0.00125
8.4E-06	0.00041	0.0006	-0.0002	-1E-05	0.0008
8.4E-06	0.00096	0.00012	-0.0009	-0.0003	0.00078
8.4E-06	0.00061	0.0001	-0.0003	2.1E-05	0.00084
8.5E-06	0.0007	-6E-05	-4E-05	-0.0006	0.00036
8.5E-06	0.00029	1.6E-05	0.00024	0.00071	0.0014
8.5E-06	0.00081	0.00101	-0.0005	-0.0005	0.00149
8.6E-06	0.00056	0.00046	-0.001	-0.0005	0.00086
8.6E-06	0.0009	-0.0003	-0.0003	-0.0002	0.0007
8.6E-06	0.00076	-0.0002	-0.0007	-6E-06	0.00088
8.7E-06	0.0005	-7E-05	-0.0004	-0.0001	0.00118
8.7E-06	0.00061	0.00052	-0.0011	-0.0003	0.00051
8.7E-06	0.00059	0.00072	-0.0006	-0.0001	0.00149
8.8E-06	0.0001	0.00025	-0.0005	-0.0008	0.00112
8.8E-06	0.00027	0.00057	-0.0009	-0.0009	0.00085
8.8E-06	0.00039	0.0005	-0.0006	-6E-05	0.00064
8.9E-06	0.00057	0.00019	-0.0009	-0.0006	0.00097
8.9E-06	0.00079	0.00069	0.00029	-0.0002	0.00194
8.9E-06	0.00014	0.00018	-0.0005	-0.001	0.00118
9E-06	0.00102	0.00017	3.2E-06	-0.0005	0.00079
9E-06	0.00065	-0.0002	-0.001	-0.0004	0.00119
9E-06	0.00072	-0.0001	0.00017	-0.0005	0.00079
9.1E-06	0.00072	0.00032	-3E-05	-0.0004	0.00055
9.1E-06	0.00122	0.0002	8.9E-06	-0.0004	0.00017
9.1E-06	-9E-05	0.00107	0.00012	-0.0005	0.00139
9.2E-06	3.2E-05	0.00072	0.00017	-0.0002	0.00115
9.2E-06	0.00036	-6E-05	-0.0004	-0.0011	0.0011

9.2E-06	0.00075	0.00026	-0.001	-0.0006	0.001
9.2E-06	0.00026	-0.0007	-0.0003	-0.0002	0.00086
9.3E-06	-0.0002	0.00073	-0.0002	-0.0011	0.0009
9.3E-06	0.00023	9.2E-05	-0.0009	-0.0005	0.00088
9.3E-06	0.00067	0.00052	-0.0009	-0.0007	0.00088
9.4E-06	0.00078	0.00028	0.00012	-0.0006	0.0008
9.4E-06	0.00044	-0.0005	-0.0006	-0.0008	0.00049
9.4E-06	0.00029	-0.0014	-0.0006	-0.0005	0.00115
9.5E-06	0.00028	-0.0006	-0.0001	-0.0002	0.0014
9.5E-06	0.00055	-0.0002	-0.0006	-0.0007	0.00115
9.5E-06	0.00017	-0.0001	-0.0003	-0.0006	7E-05
9.6E-06	0.00042	-0.0005	-0.0003	-0.0004	0.00071
9.6E-06	0.00064	-7E-07	-0.0006	-0.0002	0.00053
9.6E-06	0.00013	-0.0002	7.3E-05	0.00022	0.00119
9.7E-06	0.00027	-0.0008	-0.0003	-0.0003	0.00088
9.7E-06	0.00035	8.6E-05	-0.0004	-0.0002	0.00086
9.7E-06	0.00037	0.00026	-0.0006	-0.0002	0.00036
9.8E-06	0.00077	0.00041	-0.0001	-0.0004	0.00076
9.8E-06	0.00069	0.0003	-0.0008	-0.0006	0.00113
9.8E-06	0.00099	-0.0002	-0.001	2.2E-05	0.00081
9.9E-06	0.00115	8.5E-05	-0.0003	-0.0005	5.3E-05
9.9E-06	0.00084	-5E-05	-7E-05	-0.0004	0.00083
9.9E-06	0.00056	0.00037	0.00042	-0.0005	0.00134
1E-05	0.00076	0.00087	-0.0004	-0.0006	0.0007
1E-05	0.00039	0.00016	-0.0005	6.6E-05	0.00097
1E-05	0.00043	6.5E-05	0.00015	-0.0006	0.00111
1E-05	0.00019	0.00022	7.7E-05	-0.0001	0.00096
1E-05	0.00067	0.00093	5.9E-05	-9E-05	0.00056
1E-05	0.00092	-0.0003	-0.0009	-0.0008	0.00059
1E-05	0.00096	0.0003	-0.0006	-0.0004	0.00085
1E-05	0.00081	0.00029	-0.0007	-0.0008	0.00065
1E-05	0.00087	0.00068	-0.0002	-0.0007	0.00085
1E-05	0.0002	-0.0009	-0.0003	-0.0006	0.00063
1E-05	0.00088	0.00061	-0.0005	-0.0002	0.00036
1E-05	0.00027	4.7E-05	-0.0001	0.0001	0.00068
1E-05	0.00046	-0.0006	-0.0004	-0.0007	0.00078
1E-05	0.00059	-0.0004	-0.0012	-0.0005	0.0007
1E-05	0.00012	-0.0003	-0.0006	-0.0004	0.00053
1E-05	0.00043	0.00017	-0.0006	0.00034	0.00047
1E-05	-0.0003	-8E-05	-0.0005	0.00017	0.00044
1.1E-05	0.00071	-0.0004	-0.0013	-0.0002	0.00085
1.1E-05	0.00046	-0.0002	-0.0006	-0.0013	0.00104
1.1E-05	0.00032	0.00039	-6E-05	-0.0004	0.00099
1.1E-05	0.00078	0.00024	-0.0009	-0.0002	0.00023
1.1E-05	0.00113	0.0003	-0.0001	-8E-05	0.00087
1.1E-05	0.0009	0.00016	-0.0005	0.0001	0.00075
1.1E-05	0.00055	-0.0003	-0.0002	-0.0003	0.00031
1.1E-05	0.00029	0.00069	-0.0004	-0.0001	0.00096
1.1E-05	0.00101	7.4E-05	-0.0012	0.00023	0.00083
1.1E-05	0.00016	0.00021	-0.0004	0.00073	0.00096
1.1E-05	0.00035	0.00013	6.9E-05	-0.0004	-0.0001
1.1E-05	0.00022	2.3E-05	0.00036	-0.0004	0.00012
1.1E-05	0.00113	7.7E-05	-4E-05	-2E-05	0.00099
1.1E-05	0.00108	0.00022	8.1E-05	0.00014	0.00077

1.1E-05	0.0007	-0.0002	-0.0009	-2E-05	-4E-05
1.1E-05	0.0005	0.00047	-0.001	-0.0003	0.00082
1.1E-05	0.00095	-0.0005	-0.0004	-0.0006	0.00077
1.1E-05	0.00072	-0.0001	-0.0004	-0.0003	0.00174
1.1E-05	0.00021	0.00011	-0.0007	-0.0005	0.0009
1.1E-05	0.00084	-0.0002	-0.0009	8.6E-05	0.00028
1.1E-05	0.00079	-0.0001	-0.0012	0.00014	0.00071
1.1E-05	0.00067	-0.0002	-0.0004	-0.0002	0.00041
1.1E-05	-9E-05	3.6E-05	-0.0003	0.00067	0.00137
1.1E-05	0.00021	-0.0007	-0.001	-0.0006	0.0004
1.1E-05	0.00029	0.0004	0.00026	-0.0003	0.00075
1.1E-05	0.0002	0.00062	-0.0004	0.00022	0.00053
1.1E-05	0.00055	-7E-06	-0.0006	0.00023	0.0012
1.1E-05	0.00051	0.00047	-0.0008	-0.0002	0.0009
1.1E-05	0.00058	-0.0004	-0.0002	-0.0006	0.00048
1.1E-05	0.00064	0.00021	-0.0009	-0.0003	0.00092
1.1E-05	0.00077	0.00014	-0.0005	6.1E-05	0.00031
1.1E-05	0.00038	-0.0002	-0.0006	-0.0002	0.00057
1.2E-05	0.00093	-0.0001	-0.0005	-0.0007	0.00101
1.2E-05	0.00074	0.00088	4.1E-05	-1E-05	0.00125
1.2E-05	0.00023	4.9E-05	-2E-05	-0.0008	0.00115
1.2E-05	0.0007	0.00033	-1E-05	-0.0004	0.00063
1.2E-05	0.00067	-0.0006	-8E-05	-0.0008	0.00061
1.2E-05	0.00032	0.00013	-0.0009	0.00032	0.00072
1.2E-05	0.00052	0.00063	-0.0008	-0.0008	0.00089
1.2E-05	0.00082	0.00012	-0.0007	-0.001	0.00078
1.2E-05	0.00078	0.00041	-5E-05	-0.0005	0.00029
1.2E-05	0.00054	0.00074	-0.0003	-0.0005	0.00071
1.2E-05	0.00063	-0.0005	-0.0007	-0.0007	0.00078
1.2E-05	0.00023	-0.0005	-0.0008	2.3E-05	-0.0001
1.2E-05	0.00031	-0.0003	-0.0009	0.00016	0.00128
1.2E-05	0.00049	-0.0005	-0.001	0.00045	0.00094
1.2E-05	0.00041	-0.0005	-0.0009	-0.0005	0.00028
1.2E-05	0.00059	-0.0004	-0.0004	2.7E-05	0.00083
1.2E-05	0.00021	-0.0003	-0.0003	7.6E-05	0.00037
1.2E-05	-8E-05	-0.0006	-0.0006	0.00041	0.00055
1.2E-05	0.00084	0.00011	-0.0007	8.5E-05	0.00111
1.2E-05	0.00018	-0.0002	0.00035	-0.0001	0.00058
1.2E-05	0.00032	-0.0004	-0.0003	-0.0006	0.00054
1.2E-05	0.00056	3.6E-05	-0.0002	-7E-05	0.00111
1.2E-05	0.00024	0.00011	-0.0008	0.00032	0.00129
1.2E-05	0.00041	0.0004	-0.0008	-0.0005	0.0006
1.2E-05	0.00092	-0.0001	-0.0005	0.00043	0.0012
1.2E-05	0.00121	-0.0005	-0.0001	-0.0003	0.00073
1.2E-05	0.00082	0.00027	-0.0004	0.00054	0.00108
1.2E-05	0.00097	-0.0001	-0.0005	-0.0004	0.00085
1.2E-05	0.00077	-0.0004	-0.0003	-0.0005	0.00051
1.2E-05	0.0003	-0.0001	-0.0003	-0.0003	0.00065
1.2E-05	0.00039	-0.0002	-0.0009	0.00073	0.00136
1.3E-05	0.00037	0.00045	-0.0012	-0.0003	0.00177
1.3E-05	0.00051	0.00048	-0.0009	-0.0005	0.00051
1.3E-05	0.00069	6.2E-05	-0.0006	8.8E-06	0.00039
1.3E-05	0.00059	-3E-05	-0.0004	0.00015	0.00044
1.3E-05	0.0001	-0.0002	-0.0005	6.6E-05	0.00094

1.3E-05	1.7E-05	7.9E-07	-0.0007	0.00052	0.00076
1.3E-05	0.00035	0.00038	-0.0008	-0.0004	0.00024
1.3E-05	0.00098	0.00037	-0.0003	-8E-06	0.00065
1.3E-05	0.00072	-0.0001	-0.0011	3.5E-05	0.00057
1.3E-05	0.00073	0.00035	-0.001	-2E-05	0.00109
1.3E-05	0.0008	0.00031	0.00011	-0.0011	0.00091
1.3E-05	0.00117	-0.0001	-0.0009	-0.0017	0.00027
1.3E-05	0.00073	0.00014	-0.0003	-0.0007	0.00042
1.3E-05	1.2E-05	4.6E-05	-0.0008	-0.0003	0.00097
1.3E-05	0.00074	-4E-05	-0.0004	-7E-05	0.00107
1.3E-05	0.00065	-0.0005	-0.0008	-0.0006	0.00063
1.3E-05	0.00077	0.0002	0.00018	-0.0004	0.00046
1.3E-05	0.00074	-2E-05	-0.0007	-0.0003	-0.0002
1.3E-05	0.00082	0.0003	-0.0005	-0.0004	0.00082
1.3E-05	0.0008	-0.0002	-0.0003	0.00031	0.00056
1.3E-05	0.00077	2.2E-06	-0.0002	-0.0001	0.00056
1.3E-05	2E-05	0.00018	-0.0007	0.00013	0.00032
1.3E-05	0.00034	0.00079	-0.0008	-0.0007	0.00042
1.3E-05	0.00033	0.00046	-0.0005	-0.0003	5E-05
1.3E-05	-0.0002	0.00051	-0.0005	-3E-05	0.00066
1.3E-05	0.00011	3.7E-06	-0.0005	-0.0006	0.00101
1.3E-05	0.0005	-0.0006	-0.0005	-0.0006	0.00124
1.3E-05	0.00017	-2E-05	-0.0008	-0.0006	0.00045
1.3E-05	0.00074	-0.0004	3.1E-05	-0.0002	0.00076
1.3E-05	0.0009	-0.0007	-0.0004	0.00077	0.00065
1.3E-05	0.00046	-9E-05	-0.0007	-0.0002	0.00055
1.4E-05	0.00082	-9E-05	-0.001	0.00026	0.00078
1.4E-05	0.00028	5.4E-05	0.00046	4.6E-05	0.00093
1.4E-05	0.001	-8E-05	0.00038	-0.0006	0.00056
1.4E-05	0.00094	3.4E-05	-0.0003	0.00022	0.00112
1.4E-05	0.00079	-0.0002	-0.0001	-0.0005	0.00169
1.4E-05	0.00052	-0.0002	-0.001	-0.0008	0.00085
1.4E-05	0.0007	-0.0007	0.0003	-0.0002	0.00094
1.4E-05	0.00052	-3E-06	-0.0002	0.00024	0.00049
1.4E-05	-0.0001	-0.0006	-5E-05	-0.0009	0.00071
1.4E-05	0.0004	-0.0006	-0.0003	-0.0004	0.00094
1.4E-05	0.00071	0.00019	-0.0006	0.00034	0.00075
1.4E-05	0.00132	-0.0002	0.00033	8.2E-05	-6E-05
1.4E-05	5.2E-05	0.00033	-0.0006	-0.0008	0.00106
1.4E-05	0.00059	-0.0003	-0.0006	0.00047	0.00106
1.4E-05	0.00056	0.00051	-0.0002	-6E-05	0.00061
1.4E-05	8.3E-06	-0.0005	-0.0004	-0.0007	0.00119
1.4E-05	0.00075	0.0005	-0.0005	-0.0002	0.00041
1.4E-05	6.9E-05	-0.0002	-0.0006	-0.0005	0.0013
1.4E-05	0.00076	-0.0006	3.4E-05	-0.0002	0.00036
1.4E-05	0.00032	-0.0005	-0.0001	4.8E-05	0.00057
1.4E-05	0.0002	5.8E-05	-0.0007	-0.0003	0.00055
1.4E-05	0.00058	-0.0003	-0.0005	0.0003	-6E-06
1.4E-05	0.00035	0.00048	-0.0002	-6E-05	0.00081
1.4E-05	0.00064	-0.0007	7.9E-06	1.6E-05	0.00049
1.4E-05	0.00042	0.00015	-0.0009	0.00022	0.00044
1.4E-05	0.00082	-0.0001	0.00054	-0.0004	0.00114
1.4E-05	0.00059	-0.0004	-0.0006	-0.0011	0.00078
1.4E-05	0.0004	-7E-05	-0.0004	-0.0004	0.00098

1.4E-05	0.00039	0.00039	-0.0009	-0.0002	0.00089
1.4E-05	0.00016	-0.0005	-0.0004	0.00023	0.00143
1.4E-05	9E-05	0.00017	-0.0002	-0.0001	0.00068
1.5E-05	0.00019	-0.0006	0.00019	3.5E-05	0.0008
1.5E-05	0.00053	0.00053	-0.0005	-0.0007	0.00148
1.5E-05	0.00025	0.00028	-0.0009	-0.0006	0.00098
1.5E-05	0.00087	0.0004	-0.0004	-0.0002	0.00119
1.5E-05	0.00025	0.00034	-0.0006	9.9E-05	0.00122
1.5E-05	0.00023	3.6E-05	-0.0008	-9E-05	0.00144
1.5E-05	0.0004	-0.0002	-0.0007	-0.0002	0.00091
1.5E-05	0.0002	0.00031	-0.0006	2.9E-05	0.00035
1.5E-05	0.00056	0.00013	-0.0004	0.0005	0.00098
1.5E-05	0.00019	0.00037	-0.0005	1.5E-05	0.00057
1.5E-05	0.00029	0.00023	-0.0007	-0.0004	0.00109
1.5E-05	-8E-05	0.00055	-0.0008	-0.0003	0.00054
1.5E-05	0.00043	0.00017	-0.0005	-0.001	0.00051
1.5E-05	0.00022	0.00012	-0.0006	-0.0005	0.00068
1.5E-05	0.00085	0.00091	-0.0006	-0.0002	0.00117
1.5E-05	0.00034	0.00025	-0.0002	0.00021	0.00097

Table SI.17: Primary kinetic data for the reaction of 2-oxazolidinone (**S17**) with CumO• generated by 355 nm LFP in MeCN (**Figure SI.17**).

**t / s**	**ΔA 2-oxazolidinone (S17)**			
	**0.595M**	**0.982M**	**1.10M**	**1.761M**
-1E-06	0.00038	0.00011	-0.0014	-0.0001
-1E-06	0.00058	0.00029	-0.0022	0.00058
-9E-07	-0.0002	7.1E-05	-0.0024	3.3E-05
-9E-07	-0.0004	-0.0009	-0.0011	-0.0002
-9E-07	-0.0006	-0.0009	-0.0014	-0.0011
-8E-07	0.00021	-0.0002	-0.0019	-0.0009
-8E-07	3.7E-05	-0.0004	-0.0013	-0.0008
-8E-07	-0.0003	-0.0005	-0.0013	0.00028
-7E-07	0.00016	-0.0008	-0.0019	-0.0009
-7E-07	2.8E-05	0.00024	-0.0026	-0.0005
-7E-07	-0.0003	-7E-05	-0.0007	-0.0011
-6E-07	-0.0002	-0.0002	-0.0014	-2E-05
-6E-07	-0.0001	-0.0007	-0.002	7.9E-05
-6E-07	-0.0004	-0.0001	-0.0012	0.00011
-5E-07	-0.0007	-0.001	-0.0016	-0.0016
-5E-07	0.00011	0.00013	-0.0024	-0.0003
-5E-07	0.00035	-0.0005	-0.0021	-0.0003
-4E-07	-0.0001	-0.0002	-0.0024	-6E-05
-4E-07	-0.0005	-0.0009	-0.0022	-0.0005
-4E-07	-0.0006	0.00021	-0.0013	-0.0009
-4E-07	-0.0004	-8E-05	-0.0008	0.0001
-3E-07	0.00062	-0.0002	-0.0022	9.8E-05
-3E-07	-0.0002	-1E-05	-0.0017	-0.0006
-3E-07	0.00036	0.0003	-0.0015	-0.001
-2E-07	0.00016	0.00086	-0.0007	-9E-05
-2E-07	6.8E-05	-0.0003	-0.0012	-0.0007
-2E-07	0.00057	-0.0004	-0.0016	-0.0008
-1E-07	0.00019	0.00025	-0.0013	1.4E-05
-1E-07	-0.0004	-4E-05	-0.0019	0.00018
-6E-08	-2E-05	0.00052	-0.0019	-0.0003
-3E-08	0.0004	-0.0007	-0.0019	-0.0002
-5E-10	-0.0021	-0.0041	-0.0065	-0.0059
3.2E-08	-0.0081	-0.0103	-0.0147	-0.0168
6.4E-08	0.02674	0.02169	0.02069	0.02101
9.6E-08	0.0281	0.02533	0.02272	0.02355
1.3E-07	0.02698	0.02433	0.02156	0.02251
1.6E-07	0.02533	0.02298	0.02028	0.02105
1.9E-07	0.02347	0.02236	0.01844	0.01874
2.2E-07	0.02383	0.02144	0.01836	0.01767
2.6E-07	0.02286	0.01949	0.01719	0.01606
2.9E-07	0.02117	0.01822	0.01565	0.01515
3.2E-07	0.02093	0.01648	0.01447	0.0137
3.5E-07	0.01928	0.0174	0.01418	0.01236
3.8E-07	0.01909	0.01573	0.01278	0.01037
4.2E-07	0.01701	0.01572	0.01245	0.01162
4.5E-07	0.01721	0.01422	0.01188	0.01021
4.8E-07	0.01623	0.0132	0.01011	0.01061
5.1E-07	0.01664	0.01305	0.00954	0.00967
5.4E-07	0.01456	0.0122	0.00878	0.00847

5.8E-07	0.01433	0.01104	0.00872	0.0071
6.1E-07	0.01475	0.01052	0.00891	0.00705
6.4E-07	0.01364	0.00983	0.00775	0.00641
6.7E-07	0.01318	0.01082	0.00794	0.00613
7E-07	0.01301	0.00964	0.00676	0.00579
7.4E-07	0.01169	0.00897	0.00665	0.00485
7.7E-07	0.01191	0.009	0.00688	0.0049
8E-07	0.01139	0.0081	0.00603	0.00422
8.3E-07	0.01069	0.00705	0.00473	0.00423
8.6E-07	0.00978	0.00695	0.00495	0.00406
9E-07	0.00958	0.00729	0.0041	0.00367
9.3E-07	0.00979	0.00724	0.00365	0.00348
9.6E-07	0.00908	0.00547	0.00473	0.00288
9.9E-07	0.00876	0.00589	0.00391	0.00268
1E-06	0.00847	0.00575	0.00316	0.00318
1.1E-06	0.00809	0.00492	0.00334	0.00323
1.1E-06	0.00784	0.00534	0.00262	0.00345
1.1E-06	0.00769	0.00527	0.00301	0.00315
1.2E-06	0.00684	0.00498	0.00229	0.00206
1.2E-06	0.00644	0.00489	0.00278	0.00263
1.2E-06	0.0075	0.00435	0.00168	0.00205
1.2E-06	0.00641	0.00402	0.00202	0.00197
1.3E-06	0.00662	0.00361	0.00239	0.0014
1.3E-06	0.00631	0.00378	0.00192	0.00202
1.3E-06	0.00559	0.00403	0.0022	0.00139
1.4E-06	0.0053	0.00339	0.00146	0.00135
1.4E-06	0.00525	0.00313	0.00131	0.00151
1.4E-06	0.00554	0.00376	0.00118	0.0013
1.5E-06	0.00549	0.00342	0.00161	0.00223
1.5E-06	0.00458	0.00338	0.001	0.0011
1.5E-06	0.00489	0.0032	0.00111	0.00233
1.6E-06	0.00526	0.00287	0.00098	0.00145
1.6E-06	0.00395	0.00262	0.0008	0.0014
1.6E-06	0.0041	0.00223	0.00036	0.00059
1.7E-06	0.00445	0.00201	0.00132	0.00166
1.7E-06	0.00433	0.00238	0.00125	0.00123
1.7E-06	0.0038	0.00192	0.00068	0.00043
1.8E-06	0.00328	0.00203	0.00043	0.00048
1.8E-06	0.00346	0.00256	5.7E-05	0.00048
1.8E-06	0.00416	0.00186	0.00053	0.00081
1.9E-06	0.0032	0.00139	0.00041	0.00026
1.9E-06	0.00408	0.00228	-0.0003	0.00063
1.9E-06	0.00317	0.00124	-0.0001	0.00033
2E-06	0.00318	0.00156	-0.0008	0.00038
2E-06	0.00281	0.00202	0.00048	0.00045
2E-06	0.00312	0.00217	-0.0002	0.00125
2E-06	0.00312	0.00215	0.00022	0.00071
2.1E-06	0.00286	0.00301	6.5E-06	-0.0001
2.1E-06	0.00331	0.00179	-0.0009	0.00076
2.1E-06	0.00172	0.00171	-0.0009	0.0006
2.2E-06	0.00216	0.0014	-0.0008	0.00043
2.2E-06	0.00246	0.00123	-0.0002	-0.0003
2.2E-06	0.0028	0.00124	-2E-05	0.00043
2.3E-06	0.00259	0.00162	-0.0012	8.6E-05

2.3E-06	0.00329	0.00071	-0.0002	0.00064
2.3E-06	0.00206	0.0008	-0.0005	0.00021
2.4E-06	0.00226	8.9E-05	-0.0002	0.00132
2.4E-06	0.00259	0.00026	-0.0009	9.4E-05
2.4E-06	0.00287	0.00085	-0.0007	-0.0003
2.5E-06	0.00206	0.00115	3E-05	0.0005
2.5E-06	0.00232	0.00125	-3E-05	0.00058
2.5E-06	0.00184	0.00051	0.00015	0.00053
2.6E-06	0.00261	0.00091	-0.0008	0.00044
2.6E-06	0.00239	0.00092	-0.0007	0.00062
2.6E-06	0.00182	0.00089	-0.0004	0.00042
2.7E-06	0.00191	0.00049	-0.0005	0.00033
2.7E-06	0.00216	0.00086	0.00011	0.00038
2.7E-06	0.00228	0.00054	-0.0005	0.00102
2.8E-06	0.00198	0.00054	-0.0005	0.00073
2.8E-06	0.00106	0.00104	-0.0006	5.4E-05
2.8E-06	0.00221	0.00064	5.4E-05	0.00076
2.8E-06	0.00139	0.00142	-0.001	0.00073
2.9E-06	0.00161	0.00086	-0.0011	0.00049
2.9E-06	0.00129	0.00039	-0.0006	0.00123
2.9E-06	0.00131	0.00048	-0.0009	-0.0005
3E-06	0.00134	0.00025	-0.0006	0.00044
3E-06	0.0013	0.00144	-0.0009	0.00088
3E-06	0.00128	0.00123	-0.0009	0.00123
3.1E-06	0.00191	0.00049	-0.0008	0.00014
3.1E-06	0.00115	0.00102	-0.0004	1.3E-05
3.1E-06	0.00178	0.00096	-0.0012	0.00056
3.2E-06	0.00208	0.00108	-0.0013	0.00089
3.2E-06	0.00153	3E-05	-0.0007	0.00031
3.2E-06	0.00187	0.00061	-0.0002	0.0009
3.3E-06	0.00191	0.00092	-0.0006	-0.0002
3.3E-06	0.00184	-0.0004	-0.0014	0.00024
3.3E-06	0.00188	0.0009	-0.0006	0.00039
3.4E-06	0.00128	0.00087	-0.0013	0.00042
3.4E-06	0.00133	0.00016	-0.001	0.00038
3.4E-06	0.0018	0.00102	-0.0003	0.00055
3.5E-06	0.00182	0.00049	-0.0009	0.00048
3.5E-06	0.00247	0.00028	-0.0008	0.00077
3.5E-06	0.00132	0.00044	-0.0007	0.00045
3.6E-06	0.00133	0.00045	-0.0006	0.00035
3.6E-06	0.00077	0.00088	-0.0009	0.00069
3.6E-06	0.0013	0.00082	-0.0008	0.00064
3.6E-06	0.00085	0.00074	-0.0003	-6E-05
3.7E-06	0.00066	0.00029	-0.0011	8.6E-05
3.7E-06	0.00102	0.00058	-0.002	0.0008
3.7E-06	0.00138	0.00062	-0.0008	0.00115
3.8E-06	0.00097	0.00075	-0.0001	0.00103
3.8E-06	0.00066	0.00087	-0.0014	0.00015
3.8E-06	0.0006	0.00044	-0.0005	0.00034
3.9E-06	0.00037	0.00081	-0.0005	0.00039
3.9E-06	0.00077	0.00098	-0.0015	1.9E-05
3.9E-06	0.00138	0.00083	-0.0009	0.00069
4E-06	0.0006	0.00072	-0.0015	0.00035
4E-06	0.00179	0.00049	-0.0015	0.00044

4E-06	0.00054	0.00094	-0.0013	0.0009
4.1E-06	0.00129	0.00094	-0.0017	0.00014
4.1E-06	0.00156	0.00036	-0.001	0.00035
4.1E-06	0.00095	0.00041	-0.001	0.00047
4.2E-06	0.0008	0.0011	-0.0009	0.0006
4.2E-06	0.00042	0.00061	-0.0009	0.00023
4.2E-06	0.00066	0.00017	-0.0007	7.6E-05
4.3E-06	0.00051	0.00084	-0.0016	2E-05
4.3E-06	0.00025	0.00178	-0.0006	9.6E-06
4.3E-06	0.00094	0.00085	-0.0011	-0.0001
4.4E-06	0.00021	0.0009	-0.0007	0.0005
4.4E-06	0.00044	0.00067	-0.0008	0.00067
4.4E-06	0.00068	0.00085	-0.0007	0.00048
4.4E-06	0.00048	0.0007	-0.0009	6.5E-05
4.5E-06	0.00044	0.00046	-0.001	0.00069
4.5E-06	0.00118	0.00075	-0.0012	-4E-05
4.5E-06	0.00077	0.00105	-0.0011	-8E-05
4.6E-06	0.00145	0.00047	-0.0013	0.00016
4.6E-06	0.00117	0.00055	-0.0014	-4E-05
4.6E-06	-0.0003	0.00119	-0.0012	0.00062
4.7E-06	0.00074	0.00046	-0.001	-0.0005
4.7E-06	0.00097	0.0016	-0.002	0.00018
4.7E-06	0.00118	3.4E-05	-0.0005	-0.0006
4.8E-06	0.00037	0.00084	-0.0001	0.00011
4.8E-06	0.00092	0.00059	-0.0011	3.7E-05
4.8E-06	0.00029	0.0012	-0.0007	0.00025
4.9E-06	0.00088	-2E-05	-0.0013	0.00022
4.9E-06	0.00101	0.00063	-0.0015	0.00047
4.9E-06	0.00038	0.00149	-0.0006	-0.0003
5E-06	0.00126	0.0009	-0.0014	0.00036
5E-06	0.00121	0.00068	-0.0007	1.5E-05
5E-06	-5E-05	-0.0001	-0.0014	0.00011
5.1E-06	0.00016	0.00066	-0.0011	-0.0005
5.1E-06	0.00112	0.00013	-0.0006	-0.0002
5.1E-06	0.00105	0.00027	-0.0007	0.00025
5.2E-06	0.00062	0.00064	-0.0014	0.00022
5.2E-06	0.00084	0.00039	-0.0017	-6E-05
5.2E-06	0.00111	0.0004	-0.0002	0.00078
5.2E-06	0.00065	0.00061	-0.0018	0.0002
5.3E-06	0.00045	-3E-05	-0.0016	0.0002
5.3E-06	0.0009	0.00073	-0.0015	-0.0002
5.3E-06	0.00094	0.00061	-0.0006	-0.0006
5.4E-06	0.00059	0.00129	-0.001	-0.0004
5.4E-06	0.00108	0.0011	-0.0009	0.00081
5.4E-06	0.00142	0.0006	-0.0016	0.00011
5.5E-06	0.00078	0.00126	-0.0007	-0.0004
5.5E-06	0.00043	0.00085	-0.0009	0.00033
5.5E-06	0.00046	0.00046	-0.0013	0.00095
5.6E-06	0.00136	0.00075	-0.001	0.00127
5.6E-06	0.00143	0.00036	-0.0007	5.2E-05
5.6E-06	0.00138	0.00018	-0.0016	0.0006
5.7E-06	0.00128	0.00018	-0.002	-0.0002
5.7E-06	0.00111	0.00022	-0.0007	-0.0002
5.7E-06	-0.0002	0.0007	-0.0008	-0.0001

5.8E-06	0.00075	0.001	-0.0015	0.00026
5.8E-06	0.00155	0.00072	-0.001	0.00032
5.8E-06	0.00016	0.001	-0.0018	0.00038
5.9E-06	0.00142	0.00122	-0.001	0.00064
5.9E-06	0.00064	0.00047	-0.0014	-0.0002
5.9E-06	0.0007	0.00013	-0.001	0.00065
6E-06	0.00072	0.00071	-0.001	0.00075
6E-06	0.00132	0.00088	-0.002	0.00058
6E-06	0.00115	0.00088	-0.0011	0.0005
6E-06	0.0011	0.00068	-0.0005	0.00077
6.1E-06	0.0009	0.00084	-0.0009	0.0005
6.1E-06	0.00025	0.00034	-0.0007	0.00116
6.1E-06	0.00113	0.00014	-0.0006	0.00109
6.2E-06	0.00084	0.00039	-0.0016	-2E-05
6.2E-06	-2E-06	0.00126	-0.0014	-0.0003
6.2E-06	3.4E-05	0.00049	-0.0011	0.00087
6.3E-06	0.00051	0.00091	-0.0016	0.00126
6.3E-06	0.00154	0.00024	-0.0009	0.00046
6.3E-06	0.0007	0.001	-0.0007	0.0007
6.4E-06	0.00104	0.00122	-0.0012	6.7E-05
6.4E-06	0.0016	0.00034	-0.0002	0.00024
6.4E-06	0.00148	0.00078	-0.0008	0.00065
6.5E-06	0.00111	0.00091	-0.0015	0.00013
6.5E-06	7E-05	0.0003	-0.001	0.00056
6.5E-06	0.00061	0.00073	-0.0013	0.00128
6.6E-06	0.00068	0.0004	-0.0006	0.00093
6.6E-06	0.00067	0.00028	-0.0005	-0.0005
6.6E-06	0.00097	0.00083	-0.0009	-0.0002
6.7E-06	0.00053	0.00064	-0.0002	-3E-05
6.7E-06	0.00086	0.00069	-0.001	0.00018
6.7E-06	0.00025	0.00038	-0.0011	0.00038
6.8E-06	0.00083	-3E-05	-0.0013	0.00015
6.8E-06	0.00091	-2E-05	-0.0003	0.00043
6.8E-06	0.00085	0.00097	-0.0009	-6E-05
6.8E-06	0.0009	0.00104	-0.0008	-2E-05
6.9E-06	0.00042	0.00057	-0.0012	-0.0004
6.9E-06	0.00113	0.00022	-0.0005	0.00023
6.9E-06	-1E-04	0.00069	-0.0013	-8E-05
7E-06	0.0007	0.00134	-0.0012	0.0002
7E-06	0.00159	0.00047	-0.002	0.00095
7E-06	9.5E-05	0.0002	-0.0015	-0.0004
7.1E-06	0.00052	0.00017	-0.0011	0.00037
7.1E-06	0.00081	0.00029	-0.0017	-0.0002
7.1E-06	0.00074	-1E-05	-0.001	-0.0003
7.2E-06	0.00072	0.00046	-0.0012	-0.0004
7.2E-06	0.00087	0.00055	-0.0013	0.00055
7.2E-06	0.00057	0.00042	-0.0009	0.00051
7.3E-06	0.00049	0.00076	-0.0011	0.00102
7.3E-06	0.00018	0.00035	-0.0008	-0.0003
7.3E-06	0.00069	0.00073	-0.0018	0.00033
7.4E-06	0.00067	0.00081	-0.001	-0.0003
7.4E-06	-0.0002	0.0002	-0.0011	0.00029
7.4E-06	0.00114	0.0001	-0.0017	-0.0006
7.5E-06	0.00099	0.00117	-0.0014	-0.0006

7.5E-06	0.0008	0.0011	-0.0015	-0.0007
7.5E-06	6.9E-05	0.0009	-0.0011	0.0006
7.6E-06	0.00135	0.00108	-0.0005	0.0002
7.6E-06	0.00057	0.00064	-0.0015	-0.0005
7.6E-06	0.00074	0.00014	-0.0009	-0.0008
7.6E-06	0.00059	-7E-05	0.00011	0.00043
7.7E-06	-0.0004	0.00034	-0.0001	0.00031
7.7E-06	0.00055	0.00029	-0.0009	-0.0001
7.7E-06	0.00062	0.0008	-0.0004	-3E-05
7.8E-06	-5E-05	0.00065	-0.0008	-0.0005
7.8E-06	0.00057	0.00045	-0.0017	-0.0007
7.8E-06	0.00039	6.6E-05	-0.0016	-0.0002
7.9E-06	0.00078	0.00042	-0.001	0.00026
7.9E-06	0.00043	0.00101	-0.0007	-0.0002
7.9E-06	-7E-05	0.00115	-0.0016	0.00033
8E-06	-9E-05	0.00049	-0.0011	-0.0004
8E-06	0.00022	0.00065	-0.0007	6.8E-05
8E-06	0.0006	0.00027	-0.0012	-0.0001
8.1E-06	0.00074	-7E-05	-0.0008	0.00021
8.1E-06	0.00032	0.0002	-0.0002	0.00048
8.1E-06	0.00059	0.00065	-0.0016	0.00049
8.2E-06	0.00049	0.00061	-0.0007	0.00014
8.2E-06	0.00098	0.0003	-0.0009	0.00018
8.2E-06	-3E-06	0.0014	-0.0008	0.00019
8.3E-06	0.00117	-0.0002	-0.0015	0.00043
8.3E-06	0.00087	0.00038	-0.0016	0.0003
8.3E-06	0.00038	0.00052	-0.0011	0.0001
8.4E-06	0.00023	-0.0002	-0.0009	0.00048
8.4E-06	0.00099	0.00071	-0.0019	0.00048
8.4E-06	0.00092	0.00106	-0.002	0.00026
8.4E-06	0.00081	0.00161	-0.001	-0.0009
8.5E-06	0.00129	0.00135	-0.0015	0.00048
8.5E-06	0.00028	0.00077	-0.001	-0.0001
8.5E-06	0.00096	0.00097	-0.0017	-0.0004
8.6E-06	0.00117	0.00131	-0.0016	-0.0008
8.6E-06	0.00081	0.00067	-0.0011	-0.0003
8.6E-06	0.00083	0.0003	-0.0009	3E-05
8.7E-06	0.00049	0.00088	-0.0015	-0.0002
8.7E-06	0.00123	0.00043	-0.0012	0.00042
8.7E-06	-0.0003	0.00158	-0.0008	7.4E-05
8.8E-06	-4E-05	0.00085	-0.0007	-0.0006
8.8E-06	0.00045	0.00084	-0.0009	-0.0003
8.8E-06	0.00073	0.00062	-0.0013	0.00038
8.9E-06	0.00026	0.0004	-0.001	-0.0005
8.9E-06	0.00051	-0.0002	-0.0006	7.1E-05
8.9E-06	0.0008	0.00055	-0.0017	0.00027
9E-06	0.00068	0.00092	-0.001	-9E-06
9E-06	0.00111	7.1E-05	-0.0008	0.00021
9E-06	0.00112	0.00076	-0.0004	0.00016
9.1E-06	0.00127	0.00128	-0.001	-9E-05
9.1E-06	0.00063	0.00063	-0.0013	0.00093
9.1E-06	0.00062	0.00075	-0.0008	0.00129
9.2E-06	0.00034	5.6E-05	-0.001	0.00011
9.2E-06	1.8E-05	0.00012	-0.0009	4.9E-05

9.2E-06	0.00082	0.00088	-0.0007	-0.0007
9.2E-06	0.00093	8.3E-05	-0.0015	0.00026
9.3E-06	0.00134	0.00083	-0.0002	-0.0003
9.3E-06	0.00076	0.00142	-0.0008	-0.0001
9.3E-06	0.00068	0.00123	-0.0006	0.00021
9.4E-06	0.00045	0.00101	-0.0009	3.4E-05
9.4E-06	0.00042	0.00184	-0.0026	0.00094
9.4E-06	0.00101	0.00083	-0.0006	0.00013
9.5E-06	0.00053	0.00051	-0.0008	0.00032
9.5E-06	0.00079	0.00077	-0.0004	0.00033
9.5E-06	2.1E-05	0.00015	-0.0016	3.6E-05
9.6E-06	0.00013	0.00027	-0.0014	8E-05
9.6E-06	0.00033	0.00036	-0.0014	0.00016
9.6E-06	0.00061	0.00113	-0.001	-7E-05
9.7E-06	0.00043	0.00061	-0.0007	-1E-05
9.7E-06	0.00044	0.00013	-0.0019	-0.0008
9.7E-06	0.00011	0.00082	-0.001	-0.0009
9.8E-06	0.00092	0.00091	-0.001	-0.0004
9.8E-06	0.00086	0.00091	-0.0022	-0.0007
9.8E-06	0.00145	0.00095	-0.0019	0.00011
9.9E-06	0.00096	0.0005	-0.0005	0.00076
9.9E-06	0.0006	0.0002	-0.0014	-5E-05
9.9E-06	0.00126	0.00072	-0.0008	0.00028
1E-05	0.001	0.00151	-0.0009	-0.0004
1E-05	0.00056	0.00031	-0.0011	-0.0002
1E-05	0.00053	0.00083	-0.0013	-0.0005
1E-05	1.8E-05	-0.0002	-0.0006	-0.0008
1E-05	0.00036	-0.0001	-0.001	-0.0009
1E-05	0.0008	0.00066	-0.0008	0.00028
1E-05	0.00077	0.00049	-0.0002	7E-05
1E-05	8.2E-05	0.00104	-0.0009	-0.0004
1E-05	0.00082	-0.0001	-0.0017	-2E-05
1E-05	0.00027	0.00055	-0.0013	6.2E-05
1E-05	0.00103	0.0008	-0.0013	-0.0003
1E-05	0.00041	-1E-04	-0.0008	0.00021
1E-05	0.00065	-0.0002	-0.0013	0.00016
1E-05	0.00079	-0.0005	-0.0008	-0.0003
1E-05	0.00069	0.00042	-0.0009	-0.0005
1E-05	0.00047	0.00062	-0.0013	-0.0003
1E-05	0.00115	0.00015	-0.0009	-0.0007
1.1E-05	0.0003	0.00035	-0.0005	-0.0006
1.1E-05	0.00103	0.0002	-0.0008	0.00044
1.1E-05	0.00105	0.00043	-0.0014	0.00023
1.1E-05	0.00068	0.0004	-0.0008	0.00071
1.1E-05	0.00088	0.00011	-0.0012	0.00011
1.1E-05	0.00019	0.00124	-0.0008	-0.0007
1.1E-05	0.00065	0.00055	-0.0012	3E-05
1.1E-05	0.00029	-0.0005	-0.0013	-0.0004
1.1E-05	-0.0002	-0.0002	-0.0009	-0.0004
1.1E-05	0.00062	0.00034	-0.0009	-8E-05
1.1E-05	0.00039	0.00054	-0.0004	0.0003
1.1E-05	0.00061	-0.0006	-0.0015	-0.0003
1.1E-05	0.00025	8.9E-05	-0.0006	-0.0007
1.1E-05	0.00108	-0.0001	-0.0009	-0.0006

1.1E-05	0.00065	0.00027	-0.0008	-0.0004
1.1E-05	0.00064	0.00055	-0.0014	-0.0006
1.1E-05	0.00084	0.00044	-0.0011	-8E-05
1.1E-05	0.00037	-0.0006	-0.001	-0.0003
1.1E-05	0.00055	0.00071	0.00015	-0.0007
1.1E-05	0.00073	0.00041	-0.0011	8.6E-05
1.1E-05	0.0007	0.00023	-0.001	0.00063
1.1E-05	0.00031	0.00033	-0.0008	5.6E-05
1.1E-05	0.00095	0.00066	-0.0011	-0.0007
1.1E-05	0.00027	0.00091	-0.0011	0.00045
1.1E-05	0.00079	0.00029	-0.0006	-0.0003
1.1E-05	0.00081	0.00092	-0.0013	-0.0001
1.1E-05	-2E-05	0.00103	-0.0013	-0.0003
1.1E-05	-0.0002	0.00089	-0.0006	-0.0002
1.1E-05	-0.0003	0.00053	-0.0015	-0.0003
1.1E-05	3.9E-05	8E-05	-0.0008	0.00024
1.1E-05	0.00066	0.00035	-0.0008	0.0003
1.1E-05	0.00033	0.00044	-0.0014	0.00023
1.2E-05	0.00082	0.00033	-0.0004	6.6E-05
1.2E-05	0.00064	0.00072	-0.0007	0.00066
1.2E-05	-0.0002	0.00025	-0.0015	0.0007
1.2E-05	7.7E-05	0.00027	-0.0013	-0.0007
1.2E-05	0.00123	0.00035	-7E-05	0.00036
1.2E-05	0.00121	0.00018	-0.0016	-0.0004
1.2E-05	0.00114	0.00073	-0.0016	-0.0003
1.2E-05	6.5E-05	0.00085	-0.0004	0.00018
1.2E-05	0.00107	0.00065	-0.0011	0.00031
1.2E-05	0.00098	-5E-05	-0.001	0.00015
1.2E-05	0.00127	-0.0002	-0.0012	-0.0004
1.2E-05	0.0002	-0.0002	-0.0016	0.0003
1.2E-05	0.00033	0.00025	-0.001	0.00017
1.2E-05	-0.0003	0.00031	-0.0006	-0.0007
1.2E-05	0.00039	0.0013	-0.0009	-0.0005
1.2E-05	0.00021	0.00031	-0.0012	0.00035
1.2E-05	0.00023	0.00029	-0.0014	-0.001
1.2E-05	-0.0004	0.00029	-0.0012	1.9E-05
1.2E-05	0.00098	0.00052	-0.0016	-0.0001
1.2E-05	0.00034	0.00033	-0.001	-0.0007
1.2E-05	0.00065	0.00067	-0.0014	-0.0005
1.2E-05	0.00083	0.00086	-0.0008	-0.0008
1.2E-05	0.00052	0.00021	-0.0003	0.00037
1.2E-05	0.00012	0.00045	-0.0014	4.4E-05
1.2E-05	0.00059	0.00099	-0.0011	6.3E-05
1.2E-05	0.00019	0.00049	-0.0007	-0.0006
1.2E-05	0.00026	0.0002	-0.0017	0.00103
1.2E-05	0.00036	0.00057	-0.0018	-0.0006
1.2E-05	0.00057	0.00066	-0.0014	-0.0002
1.2E-05	0.00049	0.00133	4.1E-05	0.00032
1.2E-05	0.00119	0.00075	-0.0012	-0.0004
1.3E-05	-0.0001	0.00035	-0.0009	-0.0002
1.3E-05	-4E-05	0.00031	-0.0013	0.00041
1.3E-05	0.00051	0.00094	-0.001	0.00042
1.3E-05	0.00017	0.00076	-0.0006	-0.001
1.3E-05	0.00051	0.00039	-0.0015	-0.0006

1.3E-05	0.00027	0.0009	-0.0007	0.00038
1.3E-05	0.00128	-2E-07	-0.0012	-1E-04
1.3E-05	0.00045	0.00081	-0.001	-0.0003
1.3E-05	0.00082	0.00052	-0.0009	0.00014
1.3E-05	0.00041	0.00055	-0.0007	-0.0002
1.3E-05	0.00032	0.0007	-0.0005	-0.0008
1.3E-05	3.7E-05	-0.0003	-0.0011	-0.0008
1.3E-05	0.00017	0.00032	-0.0005	-0.0002
1.3E-05	0.00111	0.001	-0.0011	-0.0011
1.3E-05	2.1E-05	-0.0003	-0.0019	-0.0006
1.3E-05	0.00037	0.00092	-0.0013	0.00017
1.3E-05	0.00105	0.00057	-0.0018	0.00028
1.3E-05	0.00082	0.0004	-0.0013	-0.0004
1.3E-05	0.00073	-0.0003	-0.0008	0.00051
1.3E-05	0.00056	-5E-07	-0.0015	4.3E-05
1.3E-05	0.00101	0.00017	-0.0013	-0.0005
1.3E-05	0.00097	0.00054	-0.0019	-0.0001
1.3E-05	2.1E-05	0.00017	-0.0012	-0.0009
1.3E-05	0.00042	0.00147	-0.0006	-5E-05
1.3E-05	0.00017	0.00064	-0.0003	-0.0003
1.3E-05	0.00159	0.00091	-0.0012	-0.0004
1.3E-05	-0.0002	0.00051	-0.0002	-4E-05
1.3E-05	0.00067	0.00036	-0.0005	0.00095
1.3E-05	0.00067	0.00051	-0.0004	0.0008
1.3E-05	0.0004	0.00083	-0.0008	0.00104
1.3E-05	7.3E-05	0.00065	-0.0009	0.00074
1.4E-05	0.00045	0.00028	-0.0016	0.00061
1.4E-05	-7E-05	0.00034	-0.0013	0.0004
1.4E-05	-0.0008	0.00028	-0.001	0.00077
1.4E-05	-0.0001	6.1E-05	-0.001	0.00052
1.4E-05	0.00024	0.00029	-0.001	-2E-05
1.4E-05	7.8E-05	0.00039	-0.0005	-0.0007
1.4E-05	0.00137	0.00044	-0.0017	-0.0001
1.4E-05	0.00069	0.00075	-0.0015	-0.0003
1.4E-05	0.00047	-0.0003	-0.001	0.00016
1.4E-05	-9E-05	0.00025	-0.0016	0.00051
1.4E-05	-0.0001	0.00067	-0.0005	0.00029
1.4E-05	0.00093	0.00014	-0.0008	0.00075
1.4E-05	0.00094	0.00071	-0.0003	-0.0002
1.4E-05	0.00019	0.00017	-0.0009	0.0007
1.4E-05	0.00094	0.00064	-0.0013	-0.0002
1.4E-05	0.00069	0.0008	-0.0007	0.00011
1.4E-05	0.00072	0.00057	-0.0022	0.0008
1.4E-05	0.00039	0.00051	-0.0014	0.00036
1.4E-05	0.001	0.00037	-0.0006	-7E-05
1.4E-05	0.00051	0.00086	-0.0016	0.00027
1.4E-05	-0.0008	0.00038	-0.001	0.0002
1.4E-05	0.00011	0.00082	-0.0014	0.00072
1.4E-05	0.00075	0.00068	-0.0013	-0.0002
1.4E-05	0.00039	0.00099	-0.0011	0.00019
1.4E-05	0.00033	0.00083	-0.0016	-0.0002
1.4E-05	-0.0003	0.00013	-0.0007	6.5E-05
1.4E-05	0.00061	-8E-05	-0.0014	-0.0001
1.4E-05	0.00107	0.0008	-0.001	0.00069

1.4E-05	0.00061	0.00073	-0.0012	0.00021
1.4E-05	0.00025	0.00104	-0.0002	0.00054
1.4E-05	0.00039	0.00072	-0.0011	0.00013
1.5E-05	0.00077	0.00072	-0.0003	-0.0001
1.5E-05	-0.0001	3.4E-05	-0.0006	-0.0004
1.5E-05	-0.0004	0.00091	-0.0017	0.00052
1.5E-05	3.8E-05	0.00066	-0.0008	-0.0004
1.5E-05	-0.0001	0.00072	-0.0015	-0.0002
1.5E-05	-0.0003	0.00099	-0.001	-0.0004
1.5E-05	0.0004	0.00024	-0.0006	-0.0004
1.5E-05	7.8E-05	0.00057	-0.0017	7.4E-05
1.5E-05	-0.0003	0.00055	-0.0013	-0.0002
1.5E-05	0.00023	0.00029	-0.0012	-1E-04
1.5E-05	0.00137	0.00081	-0.0012	-0.0002
1.5E-05	0.00042	0.00086	-0.0013	-0.0007
1.5E-05	0.00067	0.00034	-0.0015	0.00055
1.5E-05	0.00045	0.00093	-0.0004	0.00052
1.5E-05	0.00045	0.00065	-0.0008	0.00011
1.5E-05	0.00102	0.00068	-0.0008	-0.0002

Table SI.18: Primary kinetic data for the reaction of 1-methyl-2-oxazolidinone (**S18**) with CumO$^•$ generated by 355 nm LFP in MeCN (**Figure SI.18**).

t / s	ΔA 1-methyl-2-oxazolidinone (S18)				
	**0.115M**	**0.285M**	**0.449M**	**0.608M**	**0.814M**
-1E-06	0.00044	-0.0004	-0.0007	3.3E-05	0.00096
-1E-06	0.00106	-0.0006	-0.0004	0.00035	-0.0008
-9E-07	0.00087	-7E-05	-0.0005	-0.0006	0.00081
-9E-07	0.00112	-0.0002	0.00036	-2E-05	0.00027
-9E-07	0.00042	-0.0011	-0.0002	0.00019	0.00013
-8E-07	0.00096	-0.0008	0.00038	0.00024	0.00047
-8E-07	0.00102	1.3E-05	-0.0005	9E-05	0.00142
-8E-07	0.00036	-0.0007	0.00024	-0.0003	0.00059
-7E-07	0.00035	-0.0009	-9E-05	-4E-06	0.00062
-7E-07	0.0003	-0.0006	0.00028	0.00096	0.00109
-7E-07	0.00048	-0.0003	-0.0004	-0.0002	0.00044
-6E-07	0.0008	-0.0009	8.4E-05	0.00026	0.00131
-6E-07	0.00096	-0.0006	-0.0008	-0.0008	-0.0001
-6E-07	0.00147	-0.0005	0.00028	-4E-05	0.00022
-5E-07	0.00013	-0.0012	-0.0002	-0.0008	0.00034
-5E-07	0.00078	-0.0008	-0.0003	-0.0007	0.00106
-5E-07	0.00029	-0.0006	0.00071	0.00054	0.0001
-4E-07	0.00063	-0.0006	-0.0004	0.00115	0.00037
-4E-07	0.00095	-0.0004	-0.0002	0.00074	-0.0003
-4E-07	0.001	-0.0008	-5E-05	-0.0003	-0.0002
-4E-07	0.00137	-0.0009	0.00027	0.00014	0.00022
-3E-07	0.00061	-0.0005	0.00033	0.00044	-0.0003
-3E-07	0.00099	-0.0007	-0.0003	0.00017	0.00027
-3E-07	0.0008	-1E-04	-0.0001	-0.0004	0.00088
-2E-07	0.00055	-0.0007	-0.0005	7.9E-05	0.00012
-2E-07	0.00097	-0.0001	4.5E-05	0.00014	0.00101
-2E-07	0.00081	-0.001	-0.0003	-0.0003	6.9E-05
-1E-07	0.00111	-0.0006	-0.0001	0.00025	-0.0004
-1E-07	0.00077	-0.0004	-0.0006	0.00046	-0.0003
-6E-08	0.00067	-9E-05	-0.0003	0.00122	0.00032
-3E-08	8.1E-05	-0.0008	0.00018	-0.0008	0.00065
-3E-10	-0.003	-0.0041	-0.0038	-0.004	-0.0035
3.2E-08	-0.0082	-0.0145	-0.0141	-0.0178	-0.0176
6.4E-08	0.02646	0.02258	0.02165	0.01921	0.01621
9.6E-08	0.02773	0.02686	0.02711	0.025	0.02402
1.3E-07	0.02722	0.02509	0.02574	0.02398	0.02162
1.6E-07	0.02725	0.02377	0.02436	0.02281	0.02056
1.9E-07	0.02566	0.02383	0.02243	0.02108	0.01904
2.2E-07	0.02519	0.02159	0.02102	0.01964	0.01793
2.6E-07	0.02493	0.02152	0.01991	0.01843	0.01752
2.9E-07	0.0231	0.02058	0.01958	0.0171	0.01587
3.2E-07	0.02238	0.01937	0.01765	0.01645	0.01387
3.5E-07	0.02222	0.01882	0.01701	0.0151	0.01352
3.8E-07	0.02152	0.01774	0.016	0.01394	0.01261
4.2E-07	0.02038	0.01676	0.01504	0.01291	0.01143
4.5E-07	0.02043	0.01559	0.01492	0.01323	0.01087
4.8E-07	0.01946	0.01516	0.01372	0.01173	0.01059
5.1E-07	0.01827	0.01461	0.01302	0.01103	0.009
5.4E-07	0.01785	0.01475	0.01211	0.01056	0.00926

5.8E-07	0.01754	0.01315	0.01157	0.00999	0.00828
6.1E-07	0.01793	0.01259	0.01018	0.00979	0.00807
6.4E-07	0.01665	0.01221	0.01058	0.00879	0.00673
6.7E-07	0.0167	0.01183	0.01021	0.00785	0.00709
7E-07	0.01549	0.01088	0.00971	0.00735	0.00621
7.4E-07	0.01552	0.01131	0.0096	0.00736	0.00656
7.7E-07	0.0148	0.0101	0.0087	0.00689	0.00566
8E-07	0.01416	0.01022	0.00828	0.0066	0.00497
8.3E-07	0.0133	0.00892	0.00743	0.00625	0.0049
8.6E-07	0.01325	0.00916	0.0076	0.0056	0.00409
9E-07	0.01283	0.00917	0.00727	0.00533	0.0042
9.3E-07	0.01201	0.00873	0.00704	0.00461	0.00335
9.6E-07	0.01207	0.00752	0.0066	0.00497	0.00366
9.9E-07	0.0119	0.00773	0.00603	0.00423	0.00344
1E-06	0.01149	0.00715	0.0058	0.00451	0.0031
1.1E-06	0.01108	0.00671	0.00513	0.00407	0.00336
1.1E-06	0.0114	0.00648	0.00496	0.00425	0.00263
1.1E-06	0.01028	0.00594	0.00468	0.0031	0.0032
1.2E-06	0.01002	0.00634	0.00559	0.00328	0.00298
1.2E-06	0.00962	0.00569	0.00421	0.0024	0.00312
1.2E-06	0.00949	0.00527	0.00421	0.0026	0.00303
1.2E-06	0.01036	0.00482	0.0042	0.00264	0.00296
1.3E-06	0.00911	0.00498	0.00296	0.00261	0.00253
1.3E-06	0.00901	0.00557	0.0035	0.00279	0.00252
1.3E-06	0.00892	0.00493	0.00331	0.00294	0.00274
1.4E-06	0.00864	0.00447	0.00329	0.00236	0.00228
1.4E-06	0.00911	0.00423	0.004	0.00301	0.00235
1.4E-06	0.00774	0.00383	0.00337	0.00242	0.0027
1.5E-06	0.00795	0.00449	0.00347	0.00253	0.00263
1.5E-06	0.00783	0.00362	0.00266	0.00182	0.00187
1.5E-06	0.00778	0.00502	0.00272	0.00177	0.00144
1.6E-06	0.0074	0.0031	0.00275	0.00199	0.00182
1.6E-06	0.00716	0.0035	0.00285	0.00183	0.00262
1.6E-06	0.00707	0.00329	0.00296	0.00136	0.00267
1.7E-06	0.00651	0.00352	0.00207	0.00097	0.00207
1.7E-06	0.00585	0.0028	0.00257	0.00135	0.00167
1.7E-06	0.00595	0.00358	0.00203	0.00136	0.0021
1.8E-06	0.00631	0.00302	0.00251	0.00107	0.00218
1.8E-06	0.00609	0.00198	0.00238	0.00156	0.00167
1.8E-06	0.00606	0.00177	0.0016	0.00081	0.00117
1.9E-06	0.006	0.00269	0.00159	0.00129	0.00091
1.9E-06	0.00639	0.00271	0.00181	0.00142	0.0014
1.9E-06	0.00565	0.0023	0.00198	0.00107	0.00215
2E-06	0.00578	0.00272	0.00237	0.00123	0.0018
2E-06	0.00523	0.00168	0.00121	0.00137	0.00198
2E-06	0.00494	0.00133	0.0012	0.00176	0.00093
2E-06	0.00473	0.0016	0.00072	0.00088	0.00166
2.1E-06	0.00527	0.00188	0.00068	0.00185	0.00144
2.1E-06	0.00432	0.00138	0.0009	0.0007	0.00173
2.1E-06	0.00457	0.00111	0.00158	0.00128	0.00167
2.2E-06	0.00502	0.00145	0.00115	0.00131	0.00144
2.2E-06	0.00449	0.00102	0.00077	0.00109	0.00158
2.2E-06	0.00411	0.00135	0.00126	0.00018	0.00144
2.3E-06	0.0039	0.00083	0.00132	0.00117	0.00129

2.3E-06	0.00412	0.00156	0.0017	0.00116	0.00193
2.3E-06	0.00446	0.00089	0.00121	0.00087	0.00194
2.4E-06	0.00349	0.00081	0.00045	0.00075	0.0013
2.4E-06	0.00407	0.00043	0.00074	0.00066	0.00104
2.4E-06	0.00358	0.00122	0.00026	0.00028	0.00199
2.5E-06	0.00374	0.00165	0.00053	0.00079	0.00215
2.5E-06	0.00449	0.00178	-2E-06	0.00124	0.00163
2.5E-06	0.00368	0.00167	0.0006	0.00068	0.00171
2.6E-06	0.00366	0.00176	0.00092	0.00088	0.00139
2.6E-06	0.00333	0.00021	0.00115	0.00064	0.00152
2.6E-06	0.00259	0.00067	0.00024	0.00112	0.00132
2.7E-06	0.00307	0.0003	0.0003	0.00033	0.00106
2.7E-06	0.00343	0.00094	0.00026	-6E-05	0.0014
2.7E-06	0.00254	0.00103	0.00038	2.8E-05	0.00143
2.8E-06	0.00229	0.00056	0.00085	-0.0001	0.00152
2.8E-06	0.00279	0.00024	0.00098	0.00048	0.00118
2.8E-06	0.00345	0.00065	0.00041	0.00035	0.00207
2.8E-06	0.00383	0.00103	8.4E-06	0.00053	0.00186
2.9E-06	0.00303	0.00062	0.00024	0.00061	0.00105
2.9E-06	0.00353	0.00109	0.00042	0.00042	0.00161
2.9E-06	0.00336	0.00091	-0.0004	-2E-05	0.00158
3E-06	0.0031	0.00093	0.00101	-0.0003	0.00115
3E-06	0.0028	0.00044	0.00068	-0.0006	0.00176
3E-06	0.00251	0.00026	-0.0003	-8E-05	0.00112
3.1E-06	0.00225	0.00115	0.00012	0.00129	0.0016
3.1E-06	0.00327	0.00032	0.00027	0.00136	0.00121
3.1E-06	0.00222	0.00031	-0.0006	0.00062	0.00125
3.2E-06	0.00258	0.00028	0.00017	0.00067	0.00171
3.2E-06	0.00296	0.00092	-0.0002	0.00076	0.00067
3.2E-06	0.00272	0.00047	0.00028	-4E-05	0.0021
3.3E-06	0.00266	0.00075	4.2E-05	0.00066	0.0011
3.3E-06	0.00196	2.8E-05	0.00055	0.00035	0.00016
3.3E-06	0.0032	0.00011	-0.0001	0.00031	0.00179
3.4E-06	0.00211	0.00073	0.00045	-0.0001	0.00118
3.4E-06	0.00247	3.3E-05	0.00044	0.0012	0.00153
3.4E-06	0.00187	0.00023	0.00018	0.00046	0.00148
3.5E-06	0.0022	-0.0002	-0.0002	5.9E-05	0.00172
3.5E-06	0.00208	0.00064	0.00029	0.00025	0.00151
3.5E-06	0.00167	-0.0007	-0.0007	-0.0004	0.0014
3.6E-06	0.00185	-0.0002	-0.0002	-9E-05	0.00187
3.6E-06	0.00149	0.0001	8.3E-05	-4E-05	0.00083
3.6E-06	0.00118	0.00014	-1E-04	0.00135	0.00122
3.6E-06	0.00164	-0.0005	0.00112	0.00096	0.00163
3.7E-06	0.00154	0.00024	0.00036	0.00091	0.0013
3.7E-06	0.00144	-2E-05	-0.0001	7.6E-05	0.00159
3.7E-06	0.00203	0.0009	0.00044	0.00024	0.00119
3.8E-06	0.00203	0.00065	-0.0006	0.0006	0.00164
3.8E-06	0.00191	9.3E-05	0.00051	7.9E-05	0.00179
3.8E-06	0.00183	0.00013	0.00033	0.00018	0.00161
3.9E-06	0.00226	0.00032	0.00022	0.00115	0.00125
3.9E-06	0.0021	0.00015	3.2E-05	0.00056	0.00077
3.9E-06	0.00176	-0.0006	0.00107	0.00056	0.00085
4E-06	0.00117	0.00037	5.1E-05	0.00104	0.00104
4E-06	0.00148	2.2E-05	-9E-05	0.00026	0.00105

4E-06	0.00107	-0.0005	-5E-05	-0.0003	0.00152
4.1E-06	0.00109	-0.0005	-0.0002	0.0003	0.00215
4.1E-06	0.00124	8.5E-05	0.0004	0.00095	0.00205
4.1E-06	0.00165	-4E-05	-0.0001	0.00033	0.001
4.2E-06	0.00137	0.00036	0.00028	0.00049	0.00122
4.2E-06	0.00186	0.0004	-0.0007	0.00033	0.00046
4.2E-06	0.00144	-0.0008	-0.0014	-2E-05	0.00124
4.3E-06	0.00105	5.1E-05	-0.0004	0.0005	0.00093
4.3E-06	0.0017	0.00017	0.00065	0.0001	0.00188
4.3E-06	0.00168	0.00037	0.00042	0.00016	0.00171
4.4E-06	0.00133	0.00035	6.9E-05	0.00115	0.00154
4.4E-06	0.0011	6.4E-05	-8E-05	0.00085	0.00128
4.4E-06	0.00173	0.00019	0.00047	0.00111	0.00186
4.4E-06	0.00189	3.2E-05	5.3E-05	0.00036	0.00136
4.5E-06	0.00087	-0.0003	-0.0003	0.00063	0.00143
4.5E-06	0.00158	6.3E-05	0.00043	0.00075	0.00162
4.5E-06	0.00128	-9E-05	4E-05	0.00084	0.00094
4.6E-06	0.00146	0.00015	-0.0004	0.00052	0.00129
4.6E-06	0.00159	-0.0011	0.00024	0.00039	0.00073
4.6E-06	0.00086	-0.0007	-0.0003	0.00028	0.00203
4.7E-06	0.00169	-0.0007	-0.0005	0.00106	0.00127
4.7E-06	0.0013	-0.0006	-0.0007	0.00153	0.00144
4.7E-06	0.00174	-0.0003	0.00043	0.00095	0.00094
4.8E-06	0.00183	-0.0002	-0.0007	0.00119	0.00193
4.8E-06	0.00117	-0.0004	-0.0007	0.00119	0.00153
4.8E-06	0.00141	-1E-05	4.1E-05	0.00086	0.00094
4.9E-06	0.00175	7.5E-05	0.00031	0.00126	0.0009
4.9E-06	0.00136	0.00037	0.0002	-0.0004	0.00082
4.9E-06	0.00148	-0.0013	0.00017	0.00114	0.00095
5E-06	0.00149	-0.0002	0.00019	0.00076	0.00107
5E-06	0.00138	-0.0001	-9E-05	0.00057	0.00162
5E-06	0.00227	-0.0004	-0.0004	0.001	0.00206
5.1E-06	0.00152	-0.0002	-0.0002	0.00104	0.00107
5.1E-06	0.00176	-0.0002	0.0003	0.00122	0.00181
5.1E-06	0.00128	-0.0005	0.00116	0.00116	0.00135
5.2E-06	0.00182	-0.0008	-3E-05	0.00099	0.00085
5.2E-06	0.00107	-0.0007	0.00025	0.00056	0.00089
5.2E-06	0.00123	-0.0008	-8E-05	0.00039	0.00114
5.2E-06	0.00119	5.4E-05	-0.0006	0.00049	0.00149
5.3E-06	0.00091	0.00016	0.00017	0.00045	0.00125
5.3E-06	0.00092	-0.0014	-0.0002	0.00055	0.00154
5.3E-06	0.00125	-9E-05	-0.0002	0.0004	0.00147
5.4E-06	0.00049	0.00024	0.00034	0.00063	0.0012
5.4E-06	0.00091	-0.0004	-1E-05	0.0003	0.00104
5.4E-06	0.00103	-0.0008	0.00034	0.0007	0.00157
5.5E-06	0.00165	-0.0003	-0.0007	0.00074	0.00138
5.5E-06	0.00046	0.00021	-2E-05	0.00078	0.00112
5.5E-06	0.00057	-0.0003	-0.0004	0.00119	0.00105
5.6E-06	0.00066	-0.001	-5E-05	0.00056	0.00019
5.6E-06	0.00062	-0.0002	-0.0005	0.00021	0.00233
5.6E-06	0.00093	-0.0003	-0.0003	0.00065	0.00112
5.7E-06	0.00136	-0.0002	-0.0002	0.00015	0.00122
5.7E-06	0.00051	-9E-06	0.00043	-0.0001	0.00095
5.7E-06	0.0009	-0.0001	-3E-05	-0.0001	0.00141

5.8E-06	0.00089	-0.0003	-0.0003	0.00046	0.00183
5.8E-06	0.00095	-0.0001	0.00022	7.9E-05	0.00051
5.8E-06	0.00113	0.00016	-0.0009	0.00112	0.00178
5.9E-06	0.00128	-5E-05	-1E-05	0.0005	0.00077
5.9E-06	0.00063	-0.0004	0.0009	0.00103	0.0015
5.9E-06	0.00103	0.00054	-0.0003	0.00072	0.00148
6E-06	0.00113	-0.0008	-0.0004	0.00078	0.00146
6E-06	0.00128	-0.0005	-0.0008	0.00024	0.00106
6E-06	0.00132	-0.0003	0.00052	0.00044	0.00149
6E-06	0.00129	7E-06	-0.0003	0.00053	0.00169
6.1E-06	0.00081	-0.0007	-5E-05	0.00011	0.00139
6.1E-06	0.00079	-0.0002	3.1E-05	-4E-05	0.00088
6.1E-06	0.001	-0.0001	-0.0004	0.00058	0.00162
6.2E-06	0.00062	0.00017	-0.0008	0.00023	0.00112
6.2E-06	0.00039	-0.0005	-0.0004	0.00014	0.0015
6.2E-06	0.00126	-0.0006	0.00038	0.00107	0.00092
6.3E-06	0.00028	-0.001	-0.0004	0.00085	0.00134
6.3E-06	0.00062	0.00065	0.00062	0.00043	0.00103
6.3E-06	0.00098	-9E-06	-0.001	0.00074	0.00098
6.4E-06	0.00121	-0.0002	0.00012	0.0006	0.00113
6.4E-06	0.00043	-0.0003	-0.0006	9.4E-05	0.00057
6.4E-06	0.00065	0.00013	-0.0003	-0.0006	0.00075
6.5E-06	0.00091	-0.0012	8.9E-05	1.4E-05	0.00074
6.5E-06	0.00072	-0.0004	0.00016	0.00073	0.00023
6.5E-06	0.00109	-0.0001	1.3E-05	5.9E-05	0.00071
6.6E-06	0.00138	1.4E-05	0.00018	8.2E-05	0.00109
6.6E-06	0.00069	-0.0002	0.00038	0.00053	0.00163
6.6E-06	0.00036	-0.0012	-0.0004	6.8E-06	0.00136
6.7E-06	-0.0003	-0.0004	-0.0006	0.00074	0.00209
6.7E-06	0.0008	-0.0005	-0.0005	1.2E-05	0.00183
6.7E-06	0.00166	-0.0006	-0.0002	0.00043	0.00155
6.8E-06	0.0007	7.2E-05	0.00041	0.00016	0.00142
6.8E-06	0.00138	-0.0002	-1E-05	0.00107	0.00136
6.8E-06	0.00054	-0.0001	-0.0004	0.00084	0.00154
6.8E-06	0.00105	-0.0008	-0.0003	0.0014	0.00113
6.9E-06	-0.0001	-0.0001	-0.0006	0.00021	0.00132
6.9E-06	0.0009	-0.0003	-0.0005	3.5E-05	0.00076
6.9E-06	0.00137	-0.0012	0.00084	0.00059	0.00169
7E-06	0.00062	-0.0003	-4E-06	0.00038	0.00095
7E-06	0.0008	-0.0013	-0.0007	0.00016	0.00037
7E-06	0.00031	-0.0006	-0.0008	-0.0001	0.00111
7.1E-06	0.00144	-0.0002	0.00012	0.00036	0.00067
7.1E-06	0.00043	-3E-05	-0.0001	0.00026	0.00133
7.1E-06	-4E-05	5.5E-05	0.00026	-8E-05	0.00116
7.2E-06	4.4E-05	-0.0001	-0.0003	-3E-05	0.00159
7.2E-06	0.00056	-0.0002	-0.0006	-7E-05	0.00112
7.2E-06	0.00056	8.1E-06	-0.0002	0.00097	0.00095
7.3E-06	0.00082	-0.0007	-2E-05	0.00017	0.00165
7.3E-06	0.00088	-0.0007	-0.0001	0.00098	0.00059
7.3E-06	0.00058	-9E-05	0.00031	8.5E-05	0.00052
7.4E-06	0.00107	-0.0007	-0.0005	0.00034	0.00128
7.4E-06	-0.0001	-0.0005	0.00013	0.00076	0.0005
7.4E-06	0.00109	-0.0007	-0.0004	0.00061	0.00092
7.5E-06	0.00147	-4E-05	-9E-05	0.0004	0.0013

7.5E-06	0.00042	-7E-05	0.00031	0.00036	0.00168
7.5E-06	0.00079	-0.0001	-0.0001	0.00012	0.00101
7.6E-06	0.001	-9E-05	-0.0012	0.0012	0.00171
7.6E-06	0.00047	-0.0005	-4E-05	0.00031	0.00103
7.6E-06	-9E-05	-0.0006	-0.0008	0.00053	0.00122
7.6E-06	0.00089	0.00025	-0.0006	0.00103	-0.0001
7.7E-06	0.00028	8.3E-05	-0.0017	0.00072	0.00095
7.7E-06	0.00026	-0.0008	-0.0008	-0.0003	0.00093
7.7E-06	0.001	-0.0011	-0.0002	0.00016	0.00019
7.8E-06	0.00092	-0.0003	-0.0001	0.00105	0.00011
7.8E-06	0.0004	-0.0007	-0.0007	0.00061	0.00125
7.8E-06	0.00075	-0.0003	0.00022	0.00042	0.00185
7.9E-06	0.00037	-0.0003	2E-05	0.00066	0.00092
7.9E-06	0.00043	-0.0001	-0.0001	0.00041	0.00129
7.9E-06	0.00028	-0.0007	-0.0001	0.0006	0.00142
8E-06	0.00057	0.0002	-0.0005	-0.0003	0.00135
8E-06	0.00099	-0.0006	-0.0007	0.00013	0.00129
8E-06	-0.0001	0.00022	-0.0005	0.00015	0.00032
8.1E-06	0.00067	-2E-05	-0.0004	0.00032	0.00133
8.1E-06	0.00061	-0.0001	3.4E-06	0.00037	0.00072
8.1E-06	0.00107	-9E-05	-0.0007	-0.0002	0.00108
8.2E-06	0.00065	1.2E-05	0.00025	-0.0002	0.00062
8.2E-06	0.0011	-0.0004	-0.0014	0.0013	0.00122
8.2E-06	0.00101	-0.0004	-2E-05	0.00011	0.00045
8.3E-06	0.00098	-0.0003	-0.0006	0.00066	0.00058
8.3E-06	0.00068	-0.0003	-0.0009	0.00049	0.00052
8.3E-06	0.00055	-0.0001	-0.0006	0.00068	0.00064
8.4E-06	0.00118	-0.0008	-0.0004	-0.0005	0.00127
8.4E-06	0.00041	-0.0005	-0.0003	0.00047	0.00105
8.4E-06	0.00083	-0.0008	-0.001	0.00082	0.00117
8.4E-06	0.00087	-0.0009	0.00023	-4E-05	0.00061
8.5E-06	0.00064	0.00047	-0.0006	0.00092	0.001
8.5E-06	0.00077	0.00048	0.00053	-0.0001	0.00052
8.5E-06	0.00091	-0.0008	-0.0003	0.00034	0.00071
8.6E-06	0.00098	-0.0003	0.00033	0.00038	0.00118
8.6E-06	0.00059	-0.0003	-0.0007	0.00044	0.0005
8.6E-06	0.00119	-0.0007	-0.0002	0.00078	0.00108
8.7E-06	0.00132	-0.0006	-0.0003	0.00103	2.8E-05
8.7E-06	-0.0001	-0.0011	-0.0003	0.00105	0.00087
8.7E-06	0.00099	-2E-05	-0.0015	0.00058	0.00187
8.8E-06	0.00067	-0.0004	-0.0011	6.7E-05	0.00096
8.8E-06	0.00069	-0.0007	-0.0003	0.00063	0.00037
8.8E-06	0.00047	0.00022	-0.0006	0.00037	0.00109
8.9E-06	0.00018	-0.0003	-0.0005	0.00035	0.00067
8.9E-06	0.00077	-0.0002	-0.0008	0.00077	0.00077
8.9E-06	0.00117	0.00013	0.0002	0.00137	0.00046
9E-06	4.9E-05	-0.0003	-0.0005	0.00091	0.00068
9E-06	0.0004	-0.0002	0.00026	0.00022	0.00124
9E-06	0.00059	-0.0003	-0.0002	0.00013	0.00063
9.1E-06	0.00084	-0.0007	-0.0009	0.00086	0.00097
9.1E-06	0.00095	-0.0007	-1E-04	0.00067	0.00019
9.1E-06	0.00083	-0.0006	8.5E-05	0.00076	0.0012
9.2E-06	0.00062	0.00017	-0.0008	0.00055	0.00058
9.2E-06	0.00082	-0.0004	-0.0006	0.00049	0.00071

9.2E-06	0.00028	-0.0009	-0.0008	0.00064	0.00075
9.2E-06	0.00074	0.00011	-0.0014	-0.0002	0.0012
9.3E-06	0.001	0.00012	-0.0008	0.00057	0.00113
9.3E-06	0.00063	0.00025	-0.0005	0.00014	0.00123
9.3E-06	0.00069	-0.0005	-0.0013	0.00074	0.00137
9.4E-06	0.00048	-7E-05	-0.0007	0.00051	0.00137
9.4E-06	0.00093	-0.0004	-0.0003	0.00032	0.00027
9.4E-06	0.00045	-0.0004	-0.0005	0.00055	0.00087
9.5E-06	0.00029	-9E-05	-0.0004	4.6E-05	0.00135
9.5E-06	0.00052	-0.0005	-0.0003	0.00085	0.00112
9.5E-06	0.00061	-0.0004	-0.0006	0.00079	0.00089
9.6E-06	0.00035	-9E-05	-0.0011	0.00043	0.00214
9.6E-06	0.00084	-0.0003	-0.0011	-0.0002	0.00117
9.6E-06	9.4E-05	-0.0007	-0.0004	9.4E-05	0.00151
9.7E-06	9.4E-05	-0.0004	9E-05	0.0004	0.00134
9.7E-06	-0.0005	-0.0003	-0.0002	0.00091	0.00105
9.7E-06	0.00069	-0.0004	2.5E-05	0.00019	0.00111
9.8E-06	4E-05	-0.0005	-0.0007	-0.0002	0.00078
9.8E-06	0.0003	1.7E-05	-0.0005	0.00032	0.00059
9.8E-06	0.00046	-0.0004	-0.0002	0.00079	0.00117
9.9E-06	0.00016	0.00012	-0.0003	1.1E-05	0.00079
9.9E-06	0.00086	-0.0009	-0.0004	-0.0002	0.0012
9.9E-06	0.00159	-0.0007	-0.0003	0.0002	0.00038
1E-05	0.00043	-0.0005	-7E-05	0.00012	0.00099
1E-05	0.00041	-0.0004	-0.0005	4.7E-05	0.0009
1E-05	0.00126	0.0002	0.00039	0.00044	0.00091
1E-05	0.00031	-0.0007	5.2E-05	0.00038	0.00058
1E-05	0.00035	-0.0007	0.00014	0.00095	0.00095
1E-05	0.00053	-0.0014	-0.0003	0.00031	0.00051
1E-05	0.00127	-0.0004	-0.0009	0.00071	0.00116
1E-05	0.00068	9.4E-05	-0.001	0.00014	0.00037
1E-05	0.00058	-0.0006	-0.0005	-0.0003	0.00083
1E-05	0.00051	0.00022	0.0001	0.00021	0.00045
1E-05	0.00103	-0.0014	-0.0009	0.00047	0.00032
1E-05	0.00062	-0.0003	0.00021	0.0005	0.00088
1E-05	0.00013	-0.001	-0.0004	0.00019	0.00156
1E-05	0.00053	-0.0009	-0.0014	-6E-05	0.00095
1E-05	0.00119	-0.0006	-0.0006	-0.0002	0.00045
1E-05	0.00152	-0.0002	-0.0006	0.00076	0.0006
1E-05	0.001	-0.0001	-0.0002	0.00054	0.00058
1.1E-05	0.00082	-0.0002	-0.0004	4.4E-05	0.00129
1.1E-05	0.00052	-0.0006	-0.0006	0.00033	0.00073
1.1E-05	0.00052	1.9E-05	0.00071	0.00043	0.00085
1.1E-05	0.00034	7.3E-05	-0.0001	0.0019	0.00115
1.1E-05	-5E-05	-0.0009	-0.0006	-0.0005	0.0007
1.1E-05	0.00049	-0.0011	-0.0007	0.00114	0.00054
1.1E-05	0.00024	-0.0006	-0.0004	0.00073	0.00034
1.1E-05	0.00044	-0.0002	-0.001	0.00019	0.00117
1.1E-05	0.00018	-0.0004	-0.0005	7.8E-05	0.00033
1.1E-05	0.00103	-0.0004	-0.0007	0.00047	0.00075
1.1E-05	0.00029	-0.0005	-0.0015	-0.0002	0.00042
1.1E-05	-3E-05	-0.0009	-0.0005	0.00091	0.00065
1.1E-05	0.00086	-0.0015	-0.001	0.00048	0.00135
1.1E-05	0.00088	0.00017	-0.0002	0.00024	0.00084

1.1E-05	0.00129	-0.0001	-1E-05	-9E-05	0.00061
1.1E-05	0.00088	0.00085	-0.0013	0.00087	0.00083
1.1E-05	0.00101	0.00033	-0.0013	-0.0002	0.00043
1.1E-05	0.00095	-0.0005	-0.0002	0.00037	0.00068
1.1E-05	0.00052	-0.0005	-0.0007	-2E-05	0.00099
1.1E-05	0.00153	-1E-05	-0.0001	-0.0002	0.00128
1.1E-05	0.00057	-0.0016	-0.0007	0.00016	0.00103
1.1E-05	0.00052	-0.0007	-0.0006	-0.0001	0.00078
1.1E-05	0.00047	-0.0009	-0.0003	-0.0003	0.00052
1.1E-05	0.00043	-0.0009	-0.0004	0.00021	0.00032
1.1E-05	0.00074	-0.0002	-0.0006	0.00026	0.00106
1.1E-05	0.00039	-0.0006	-0.0007	0.00049	0.00068
1.1E-05	0.00124	-0.001	-0.0003	0.0004	0.00162
1.1E-05	0.00082	-0.0006	-0.001	0.001	0.00069
1.1E-05	0.00112	-0.0006	0.00056	0.00071	0.0007
1.1E-05	8.3E-05	-0.0006	-0.0006	0.00031	0.00088
1.1E-05	0.00064	0.00017	-0.0004	0.00017	0.00088
1.1E-05	0.00054	-0.0006	-0.0004	-9E-05	0.00121
1.2E-05	0.00042	-0.0002	-0.0005	0.00065	0.0007
1.2E-05	0.00159	-0.0011	0.00033	0.00016	0.0012
1.2E-05	0.00115	-0.0002	-0.0003	-1E-04	0.00101
1.2E-05	0.00025	0.00051	-4E-05	0.00138	0.00123
1.2E-05	0.001	0.00049	-0.0005	0.00022	5.7E-05
1.2E-05	0.00041	-0.0003	-0.001	0.00131	0.00076
1.2E-05	7.4E-05	-0.0005	-0.0005	0.00038	0.00085
1.2E-05	0.00023	-0.0003	-0.0004	-0.0004	0.00073
1.2E-05	0.0007	-0.0004	-0.0001	0.00111	0.00014
1.2E-05	0.00064	-0.0007	0.0002	0.00074	0.00098
1.2E-05	0.00097	-0.0005	-0.001	-9E-06	9.8E-05
1.2E-05	0.00026	-0.001	-7E-05	0.00016	0.00084
1.2E-05	0.00025	-5E-05	0.0002	0.00119	0.00099
1.2E-05	0.00131	-0.0003	-0.0014	0.00099	0.00057
1.2E-05	0.00162	-0.0001	-0.0004	0.00038	9.4E-05
1.2E-05	0.00182	-0.0012	0.00079	0.00075	0.0008
1.2E-05	0.00149	0.00044	-5E-05	0.00019	0.00076
1.2E-05	0.00042	-0.0009	-0.0008	0.00043	0.00157
1.2E-05	0.00115	-0.0005	-0.0001	-0.0003	0.00087
1.2E-05	0.00046	-0.0005	-0.0009	0.00021	0.00045
1.2E-05	0.00164	-0.0003	-0.0008	-0.0003	0.00116
1.2E-05	0.00094	-0.0009	-0.0006	0.00064	0.00041
1.2E-05	0.0003	0.00032	-0.0007	0.00108	0.0006
1.2E-05	0.0012	0.00014	-0.0004	0.00072	0.00071
1.2E-05	0.00127	-0.0004	-0.0007	0.00024	0.00085
1.2E-05	0.00051	-0.001	-0.0003	0.00034	0.00092
1.2E-05	0.00094	-0.0005	0.00026	0.00021	0.00101
1.2E-05	0.00078	-0.0006	-0.0006	-0.0002	0.00051
1.2E-05	0.00137	-0.0001	-0.0004	0.00057	0.00041
1.2E-05	7.5E-06	-0.0007	-0.0003	0.00025	0.00087
1.2E-05	0.00056	-0.0004	-0.0005	-0.0002	0.00119
1.3E-05	0.00038	-0.001	8.9E-05	-0.0001	0.00062
1.3E-05	0.00017	-0.0004	-0.0005	7.2E-05	0.00033
1.3E-05	0.00019	-0.0006	-0.0003	0.00042	0.0008
1.3E-05	0.00113	-0.0005	0.00041	0.00045	0.0005
1.3E-05	-0.0004	-0.0009	-0.0003	6.5E-05	0.00063

1.3E-05	-0.0005	-0.0006	-0.0004	-3E-05	0.00086
1.3E-05	0.00115	-0.0005	-0.0006	0.00016	0.00104
1.3E-05	0.00118	-0.0008	-0.0006	4.9E-05	0.00059
1.3E-05	0.00059	0.00013	-0.0003	0.00042	0.00078
1.3E-05	0.00104	-0.0004	3.7E-05	-1E-05	0.00058
1.3E-05	0.00045	-0.0005	0.00076	0.00048	-0.0002
1.3E-05	0.00106	-0.0008	-6E-05	0.00032	0.00102
1.3E-05	0.00071	-0.0006	0.00012	0.00034	0.00034
1.3E-05	0.00043	-0.0004	-0.0002	0.00056	0.00058
1.3E-05	0.00061	-0.0002	-2E-05	0.00015	0.00067
1.3E-05	0.00046	-0.0008	-0.0009	0.00055	0.00113
1.3E-05	0.00144	-0.0007	-0.001	0.00011	-0.0002
1.3E-05	0.00175	-0.0011	-0.0008	0.00071	0.0007
1.3E-05	0.00141	-0.0008	0.00048	0.00047	0.00061
1.3E-05	0.00112	-0.0007	-0.0007	0.0007	0.0008
1.3E-05	0.00046	-0.0005	-0.0011	0.00021	0.00076
1.3E-05	0.00119	-0.0011	-0.0007	0.0005	0.00058
1.3E-05	0.00029	0.00033	0.00078	0.00081	0.00043
1.3E-05	0.00138	-0.0002	-0.0001	0.00052	0.00087
1.3E-05	0.00057	-0.0005	-0.0002	0.00058	-0.0001
1.3E-05	0.00045	-0.001	-0.0002	0.00063	0.00047
1.3E-05	-0.0002	-0.0009	-0.0004	0.00014	0.00014
1.3E-05	0.00068	-0.0005	5.4E-05	6.4E-05	0.00074
1.3E-05	0.00012	-0.0013	-0.0001	0.00033	0.00059
1.3E-05	0.00081	-0.0006	-0.0004	0.00098	0.00071
1.3E-05	0.00025	-0.0008	-0.0001	0.00081	0.00049
1.4E-05	0.00032	-0.0004	8E-05	0.00036	0.00018
1.4E-05	0.00034	-0.0003	-0.0002	-0.0003	0.00068
1.4E-05	0.00037	0.00078	-2E-05	0.00035	0.00068
1.4E-05	0.00021	-0.0002	-0.0003	0.00041	0.00072
1.4E-05	0.00156	-0.0004	0.0008	-0.0006	0.00138
1.4E-05	0.00034	8.7E-05	-0.0005	0.00046	0.00085
1.4E-05	0.00074	-0.001	9E-05	0.00054	0.00069
1.4E-05	0.0005	-0.0003	-0.0003	-0.0003	0.0006
1.4E-05	0.0015	-0.0006	-5E-05	0.00012	-0.0001
1.4E-05	0.00039	-0.0001	-0.0008	-0.0003	0.00079
1.4E-05	0.0007	-0.0007	-4E-06	0.00058	0.00091
1.4E-05	0.00072	-0.0006	-0.0011	0.00026	0.00039
1.4E-05	0.00149	-0.0007	-0.0007	-7E-05	0.00085
1.4E-05	0.00038	-0.0008	-0.0004	7.3E-05	0.00058
1.4E-05	0.00049	-0.0004	-0.0003	0.00035	0.00126
1.4E-05	0.00113	-0.0012	-0.0005	0.00043	0.00071
1.4E-05	0.00093	-0.0001	-0.0012	-0.0003	0.00046
1.4E-05	0.00124	-0.0005	-0.0006	0.00026	0.00064
1.4E-05	0.00116	-0.001	-0.0003	-0.0006	0.00107
1.4E-05	0.00085	-0.0009	-6E-05	0.00104	0.00025
1.4E-05	0.00088	-0.0005	-0.0002	0.00022	0.00076
1.4E-05	0.00131	-0.0005	-4E-05	-0.0003	0.00024
1.4E-05	0.00088	-0.0006	-0.0001	9.8E-05	0.00061
1.4E-05	0.00069	-0.0003	-5E-05	-2E-05	5.1E-05
1.4E-05	0.00125	-0.0002	5.8E-05	0.00067	0.00106
1.4E-05	0.0004	-0.0006	0.00024	-0.0001	5.9E-06
1.4E-05	0.00045	-0.0004	-0.0002	0.00016	0.00093
1.4E-05	-0.0003	-0.0002	-0.0011	0.00049	0.00153

1.4E-05	0.00075	-0.0007	-0.0007	0.00057	0.00081
1.4E-05	0.00134	8.2E-05	-0.0006	0.00072	0.00063
1.4E-05	0.00117	-0.0003	-0.0003	-5E-06	0.00109
1.5E-05	0.00055	0.00012	-2E-05	9.5E-05	0.00109
1.5E-05	0.0009	0.00037	0.00016	0.00092	0.00062
1.5E-05	-1E-05	0.00042	0.00018	-0.0003	0.00047
1.5E-05	0.00079	-0.0002	-0.0001	0.0006	0.00131
1.5E-05	0.00129	-0.0013	0.00053	0.0004	0.00134
1.5E-05	-0.0001	-0.001	-0.0005	0.00035	0.00126
1.5E-05	0.00085	-0.0003	-3E-05	-0.0004	0.00075
1.5E-05	0.00133	-0.0008	-7E-05	-0.0003	0.00101
1.5E-05	0.00087	-0.0001	-0.0002	-0.0002	0.00165
1.5E-05	0.00024	-0.0007	7.9E-05	0.00053	0.00072
1.5E-05	0.00034	-0.0002	-0.0002	0.00082	0.00043
1.5E-05	0.00028	-0.0007	-0.0007	-0.0008	0.00084
1.5E-05	0.00039	0.00018	-0.0007	0.00019	0.00108
1.5E-05	0.0005	-0.0005	-0.0002	-0.0003	0.00053
1.5E-05	1.9E-05	9E-05	-6E-05	-2E-06	0.00085
1.5E-05	-0.0002	-0.0004	-0.0003	0.00067	0.00056

Table SI.19: Primary kinetic data for the reaction of succinimide (**S19**) with CumO• generated by 355 nm LFP in DMSO (**Figure SI.19**).

t / s	ΔA succinimmide (S19)	
	0.105M	1.4M
-1E-06	0.00048	-0.0005
-1E-06	0.00117	-0.0003
-9E-07	0.00117	-0.0006
-9E-07	0.00063	-3E-05
-9E-07	0.00059	-0.0004
-8E-07	0.00166	-0.001
-8E-07	0.00217	-0.0002
-8E-07	0.00074	-0.0002
-7E-07	0.00053	-0.0005
-7E-07	0.0012	-0.0012
-7E-07	0.00103	-0.0014
-6E-07	0.00062	-0.0014
-6E-07	0.00128	-0.001
-6E-07	0.00093	-0.0009
-5E-07	0.00097	-0.0005
-5E-07	0.00099	-0.0004
-5E-07	0.00128	-0.0012
-4E-07	0.00123	-0.0008
-4E-07	0.00066	-0.0002
-4E-07	-1E-06	-0.0001
-4E-07	0.00096	-0.0006
-3E-07	0.0005	4.3E-05
-3E-07	0.0004	-0.0003
-3E-07	0.00051	-0.0006
-2E-07	0.0003	8.1E-05
-2E-07	0.00132	-0.0002
-2E-07	0.00133	-0.0005
-1E-07	0.00132	-0.0003
-1E-07	0.00085	-0.0005
-6E-08	0.00178	-0.0009
-3E-08	0.00086	-0.0004
-2E-10	-0.002	-0.0053
3.2E-08	-0.0112	-0.022
6.4E-08	0.02184	-0.0145
9.6E-08	0.02557	0.01667
1.3E-07	0.02602	0.01955
1.6E-07	0.02415	0.01918
1.9E-07	0.02396	0.0187
2.2E-07	0.02297	0.01703
2.6E-07	0.02278	0.01631
2.9E-07	0.02274	0.01585
3.2E-07	0.02265	0.01542
3.5E-07	0.02102	0.0153
3.8E-07	0.02099	0.01484
4.2E-07	0.02061	0.01512
4.5E-07	0.01958	0.01445
4.8E-07	0.01938	0.01317
5.1E-07	0.01874	0.01262
5.4E-07	0.01838	0.01361

5.8E-07	0.01788	0.01231
6.1E-07	0.01798	0.01199
6.4E-07	0.01788	0.01156
6.7E-07	0.01695	0.01144
7E-07	0.01713	0.0105
7.4E-07	0.01585	0.01076
7.7E-07	0.01626	0.01006
8E-07	0.01533	0.01032
8.3E-07	0.0139	0.00944
8.6E-07	0.01421	0.01068
9E-07	0.01454	0.01035
9.3E-07	0.01357	0.00995
9.6E-07	0.0128	0.00868
9.9E-07	0.01349	0.00855
1E-06	0.01269	0.00808
1.1E-06	0.01263	0.00813
1.1E-06	0.01239	0.00803
1.1E-06	0.01254	0.00776
1.2E-06	0.01243	0.00851
1.2E-06	0.01207	0.00839
1.2E-06	0.01267	0.00719
1.2E-06	0.01096	0.00718
1.3E-06	0.01038	0.00689
1.3E-06	0.01101	0.00782
1.3E-06	0.01036	0.00707
1.4E-06	0.01058	0.00728
1.4E-06	0.0097	0.00661
1.4E-06	0.01026	0.00641
1.5E-06	0.01036	0.00635
1.5E-06	0.00968	0.00606
1.5E-06	0.01041	0.00592
1.6E-06	0.00944	0.00592
1.6E-06	0.00887	0.00634
1.6E-06	0.00858	0.00548
1.7E-06	0.00851	0.00537
1.7E-06	0.00881	0.00673
1.7E-06	0.00811	0.00619
1.8E-06	0.00825	0.00583
1.8E-06	0.00824	0.00557
1.8E-06	0.00748	0.00537
1.9E-06	0.0085	0.00478
1.9E-06	0.00803	0.00463
1.9E-06	0.00819	0.00497
2E-06	0.00771	0.0052
2E-06	0.00751	0.00394
2E-06	0.00738	0.00446
2E-06	0.00778	0.00428
2.1E-06	0.0075	0.00431
2.1E-06	0.00693	0.00533
2.1E-06	0.0073	0.00418
2.2E-06	0.00777	0.00421
2.2E-06	0.00739	0.00322
2.2E-06	0.00716	0.0039
2.3E-06	0.0066	0.00464

2.3E-06	0.00633	0.00443
2.3E-06	0.00665	0.00376
2.4E-06	0.00658	0.00404
2.4E-06	0.00588	0.0038
2.4E-06	0.00555	0.00404
2.5E-06	0.00629	0.00383
2.5E-06	0.00561	0.00368
2.5E-06	0.00619	0.00369
2.6E-06	0.00564	0.0036
2.6E-06	0.00622	0.00372
2.6E-06	0.00537	0.00361
2.7E-06	0.00493	0.00275
2.7E-06	0.00557	0.00307
2.7E-06	0.0058	0.00329
2.8E-06	0.00569	0.00267
2.8E-06	0.00516	0.00341
2.8E-06	0.00565	0.00257
2.8E-06	0.00586	0.0033
2.9E-06	0.00443	0.00274
2.9E-06	0.00476	0.00357
2.9E-06	0.00481	0.003
3E-06	0.00455	0.00308
3E-06	0.00391	0.00297
3E-06	0.00396	0.00255
3.1E-06	0.0049	0.00342
3.1E-06	0.00512	0.00231
3.1E-06	0.00356	0.00253
3.2E-06	0.00384	0.0031
3.2E-06	0.004	0.00306
3.2E-06	0.00404	0.00253
3.3E-06	0.0045	0.00256
3.3E-06	0.00461	0.00268
3.3E-06	0.00386	0.00351
3.4E-06	0.00356	0.00288
3.4E-06	0.004	0.0027
3.4E-06	0.00394	0.00233
3.5E-06	0.00348	0.00307
3.5E-06	0.0037	0.00271
3.5E-06	0.00414	0.00212
3.6E-06	0.00382	0.0023
3.6E-06	0.00415	0.00206
3.6E-06	0.00367	0.00165
3.6E-06	0.00366	0.00194
3.7E-06	0.00323	0.00187
3.7E-06	0.00384	0.00184
3.7E-06	0.00403	0.00269
3.8E-06	0.00363	0.00183
3.8E-06	0.00352	0.0018
3.8E-06	0.0036	0.00242
3.9E-06	0.0033	0.00205
3.9E-06	0.00359	0.00203
3.9E-06	0.00383	0.00196
4E-06	0.00395	0.00142
4E-06	0.00351	0.00159

4E-06	0.0033	0.00155
4.1E-06	0.00343	0.00108
4.1E-06	0.00425	0.00158
4.1E-06	0.00325	0.00126
4.2E-06	0.00342	0.00166
4.2E-06	0.00348	0.00145
4.2E-06	0.00266	0.0015
4.3E-06	0.0031	0.00152
4.3E-06	0.00301	0.00166
4.3E-06	0.00384	0.00181
4.4E-06	0.00321	0.00218
4.4E-06	0.0035	0.00165
4.4E-06	0.003	0.0016
4.4E-06	0.00297	0.00195
4.5E-06	0.00329	0.00192
4.5E-06	0.00284	0.00162
4.5E-06	0.00327	0.00126
4.6E-06	0.00314	0.00234
4.6E-06	0.0034	0.00153
4.6E-06	0.00328	0.00187
4.7E-06	0.00328	0.00135
4.7E-06	0.003	0.00181
4.7E-06	0.00312	0.00178
4.8E-06	0.00294	0.0019
4.8E-06	0.00318	0.00141
4.8E-06	0.00373	0.00107
4.9E-06	0.0031	0.00113
4.9E-06	0.00364	0.00157
4.9E-06	0.00373	0.00195
5E-06	0.00354	0.00151
5E-06	0.00345	0.00236
5E-06	0.00325	0.00213
5.1E-06	0.00306	0.00185
5.1E-06	0.0033	0.00148
5.1E-06	0.00285	0.00173
5.2E-06	0.00266	0.0018
5.2E-06	0.00313	0.00191
5.2E-06	0.00245	0.00137
5.2E-06	0.0029	0.00109
5.3E-06	0.00232	0.00242
5.3E-06	0.00356	0.00124
5.3E-06	0.00286	0.00171
5.4E-06	0.00365	0.00129
5.4E-06	0.00325	0.00154
5.4E-06	0.00306	0.00144
5.5E-06	0.00298	0.00141
5.5E-06	0.00293	0.00149
5.5E-06	0.00311	0.00165
5.6E-06	0.00286	0.00129
5.6E-06	0.00336	0.00114
5.6E-06	0.00326	0.00209
5.7E-06	0.00326	0.00122
5.7E-06	0.00315	0.00108
5.7E-06	0.00264	0.00109

5.8E-06	0.00326	0.00082
5.8E-06	0.00308	0.00171
5.8E-06	0.00279	0.00066
5.9E-06	0.00257	0.001
5.9E-06	0.00293	0.001
5.9E-06	0.00288	0.00139
6E-06	0.00289	0.00085
6E-06	0.00374	0.00135
6E-06	0.0026	0.00067
6E-06	0.00273	0.00092
6.1E-06	0.00291	0.00263
6.1E-06	0.00294	0.00204
6.1E-06	0.003	0.00218
6.2E-06	0.0033	0.00158
6.2E-06	0.00281	0.00163
6.2E-06	0.00302	0.00171
6.3E-06	0.00312	0.00142
6.3E-06	0.00345	0.00134
6.3E-06	0.00344	0.00196
6.4E-06	0.00244	0.00107
6.4E-06	0.00276	0.00109
6.4E-06	0.00176	0.00174
6.5E-06	0.00335	0.00173
6.5E-06	0.003	0.00138
6.5E-06	0.00315	0.00201
6.6E-06	0.00259	0.00102
6.6E-06	0.00225	0.00153
6.6E-06	0.00299	0.0009
6.7E-06	0.00258	0.00184
6.7E-06	0.00255	0.00169
6.7E-06	0.00242	0.0005
6.8E-06	0.00256	0.00138
6.8E-06	0.00307	0.00099
6.8E-06	0.00268	0.00102
6.8E-06	0.00297	0.00169
6.9E-06	0.00351	0.00182
6.9E-06	0.00336	0.00189
6.9E-06	0.00295	0.00168
7E-06	0.00268	0.00144
7E-06	0.003	0.00154
7E-06	0.00253	0.00129
7.1E-06	0.00291	0.00031
7.1E-06	0.00273	0.00133
7.1E-06	0.00325	0.00123
7.2E-06	0.00228	0.00138
7.2E-06	0.00359	0.00066
7.2E-06	0.00344	0.00015
7.3E-06	0.00268	0.00036
7.3E-06	0.0026	0.00146
7.3E-06	0.0027	0.00148
7.4E-06	0.00315	0.0017
7.4E-06	0.0029	0.001
7.4E-06	0.00272	0.00114
7.5E-06	0.00345	0.0009

7.5E-06	0.00292	0.00083
7.5E-06	0.00308	0.00125
7.6E-06	0.00318	0.00182
7.6E-06	0.00296	0.00073
7.6E-06	0.00282	0.00186
7.6E-06	0.00326	0.00062
7.7E-06	0.00337	0.0016
7.7E-06	0.00244	0.00093
7.7E-06	0.00298	0.0006
7.8E-06	0.00264	0.00129
7.8E-06	0.00243	0.00038
7.8E-06	0.00295	0.00021
7.9E-06	0.00281	0.0008
7.9E-06	0.00278	0.00195
7.9E-06	0.00255	0.00141
8E-06	0.00235	0.00193
8E-06	0.00255	0.00105
8E-06	0.00291	0.00014
8.1E-06	0.00365	0.00074
8.1E-06	0.00251	0.00117
8.1E-06	0.0032	0.00152
8.2E-06	0.00363	0.00143
8.2E-06	0.00297	0.00128
8.2E-06	0.00259	0.00124
8.3E-06	0.00238	0.00112
8.3E-06	0.00295	0.00146
8.3E-06	0.00241	0.00097
8.4E-06	0.00236	0.00147
8.4E-06	0.00231	0.00077
8.4E-06	0.00233	0.0015
8.4E-06	0.00323	0.00184
8.5E-06	0.00268	0.00165
8.5E-06	0.00318	0.00098
8.5E-06	0.00288	0.00028
8.6E-06	0.00268	0.00171
8.6E-06	0.00264	0.0005
8.6E-06	0.00302	0.00139
8.7E-06	0.00277	0.00073
8.7E-06	0.00291	1E-04
8.7E-06	0.00234	0.00117
8.8E-06	0.00272	0.00037
8.8E-06	0.00232	0.00186
8.8E-06	0.00314	0.00138
8.9E-06	0.00246	0.00132
8.9E-06	0.00279	0.00085
8.9E-06	0.00296	0.00112
9E-06	0.00315	0.00106
9E-06	0.00304	0.00158
9E-06	0.00302	0.00096
9.1E-06	0.00285	0.00054
9.1E-06	0.003	0.00176
9.1E-06	0.003	0.00136
9.2E-06	0.00292	0.00162
9.2E-06	0.00288	0.00125

9.2E-06	0.00301	0.00117
9.2E-06	0.00285	4.3E-05
9.3E-06	0.00281	0.00121
9.3E-06	0.00187	0.00126
9.3E-06	0.00242	0.0015
9.4E-06	0.00183	0.00108
9.4E-06	0.00226	0.00163
9.4E-06	0.00308	0.00115
9.5E-06	0.00363	0.00093
9.5E-06	0.00329	0.00161
9.5E-06	0.00225	0.0008
9.6E-06	0.00269	0.00161
9.6E-06	0.0024	0.00133
9.6E-06	0.00299	0.00101
9.7E-06	0.00239	0.00049
9.7E-06	0.00252	0.00149
9.7E-06	0.00274	0.0014
9.8E-06	0.00323	0.00117
9.8E-06	0.00282	0.00129
9.8E-06	0.00255	0.00014
9.9E-06	0.00259	0.00116
9.9E-06	0.00256	0.00199
9.9E-06	0.00272	0.00188
1E-05	0.00256	0.00106
1E-05	0.00283	0.00153
1E-05	0.00296	0.00047
1E-05	0.00299	0.00088
1E-05	0.00277	0.00122
1E-05	0.00239	-0.0005
1E-05	0.00249	0.0009
1E-05	0.0024	0.00092
1E-05	0.00243	0.00117
1E-05	0.00259	0.00064
1E-05	0.00387	0.0014
1E-05	0.00279	0.00152
1E-05	0.00296	0.00098
1E-05	0.00266	0.00145
1E-05	0.00291	0.00142
1E-05	0.0026	0.00149
1E-05	0.00279	0.00127
1.1E-05	0.00305	0.00071
1.1E-05	0.00248	0.00048
1.1E-05	0.00277	0.00076
1.1E-05	0.00267	0.00065
1.1E-05	0.00237	0.00084
1.1E-05	0.00242	0.00114
1.1E-05	0.00298	0.00151
1.1E-05	0.00222	0.0018
1.1E-05	0.00257	0.00062
1.1E-05	0.00258	0.00108
1.1E-05	0.00262	0.00148
1.1E-05	0.0031	0.00184
1.1E-05	0.00238	0.00081
1.1E-05	0.00242	0.00119

1.1E-05	0.00258	0.0017
1.1E-05	0.00321	0.00118
1.1E-05	0.00214	0.00032
1.1E-05	0.00098	0.00096
1.1E-05	0.00271	0.00099
1.1E-05	0.0025	0.00068
1.1E-05	0.00319	0.00079
1.1E-05	0.00274	0.00198
1.1E-05	0.00212	0.00096
1.1E-05	0.00262	0.00214
1.1E-05	0.00243	0.00094
1.1E-05	0.0023	0.00124
1.1E-05	0.00192	0.00028
1.1E-05	0.00216	0.00068
1.1E-05	0.00317	0.00107
1.1E-05	0.0029	0.00103
1.1E-05	0.00325	0.00103
1.1E-05	0.00295	0.00079
1.2E-05	0.00243	0.00076
1.2E-05	0.00268	0.00062
1.2E-05	0.00232	0.00154
1.2E-05	0.00218	0.00107
1.2E-05	0.00215	0.00039
1.2E-05	0.00202	0.00133
1.2E-05	0.00185	0.00125
1.2E-05	0.0017	0.00115
1.2E-05	0.00242	0.00091
1.2E-05	0.00312	0.00112
1.2E-05	0.00341	0.00046
1.2E-05	0.00316	0.00148
1.2E-05	0.00153	0.00111
1.2E-05	0.00189	0.00103
1.2E-05	0.00251	0.00048
1.2E-05	0.00262	0.00078
1.2E-05	0.002	0.00043
1.2E-05	0.00211	0.00152
1.2E-05	0.00247	0.00164
1.2E-05	0.00208	0.00161
1.2E-05	0.00295	0.00058
1.2E-05	0.00333	0.00195
1.2E-05	0.00262	0.00052
1.2E-05	0.00175	0.00115
1.2E-05	0.00237	0.0005
1.2E-05	0.003	0.00111
1.2E-05	0.00303	0.00077
1.2E-05	0.00246	0.00063
1.2E-05	0.00282	0.00127
1.2E-05	0.00311	0.00109
1.2E-05	0.00273	0.0011
1.3E-05	0.00313	0.00071
1.3E-05	0.00284	0.00068
1.3E-05	0.00202	0.00051
1.3E-05	0.00256	0.00059
1.3E-05	0.0028	0.00107

1.3E-05	0.00217	0.0004
1.3E-05	0.00271	0.00064
1.3E-05	0.00246	0.0014
1.3E-05	0.00309	0.00127
1.3E-05	0.00307	0.00083
1.3E-05	0.00291	0.00039
1.3E-05	0.0031	0.00179
1.3E-05	0.00275	0.00074
1.3E-05	0.00206	0.00061
1.3E-05	0.00235	0.00107
1.3E-05	0.0022	0.00113
1.3E-05	0.00234	0.00151
1.3E-05	0.00206	0.00138
1.3E-05	0.00327	0.00059
1.3E-05	0.00286	0.00033
1.3E-05	0.00357	0.00088
1.3E-05	0.00229	0.00065
1.3E-05	0.003	0.00101
1.3E-05	0.00224	0.0011
1.3E-05	0.00199	0.00051
1.3E-05	0.00315	0.00087
1.3E-05	0.00304	0.00109
1.3E-05	0.00192	0.00129
1.3E-05	0.0022	0.00112
1.3E-05	0.00215	0.00083
1.3E-05	0.00219	0.00084
1.4E-05	0.00244	0.00054
1.4E-05	0.0028	0.00114
1.4E-05	0.00286	0.0003
1.4E-05	0.00222	0.00127
1.4E-05	0.00243	0.00089
1.4E-05	0.00233	0.00122
1.4E-05	0.00271	0.00055
1.4E-05	0.00223	0.00062
1.4E-05	0.00243	0.00085
1.4E-05	0.00297	0.00194
1.4E-05	0.00271	0.00046
1.4E-05	0.00269	0.00042
1.4E-05	0.00296	0.00088
1.4E-05	0.00212	0.0012
1.4E-05	0.00352	0.00124
1.4E-05	0.00277	0.0014
1.4E-05	0.00283	0.00084
1.4E-05	0.00255	0.00097
1.4E-05	0.00236	0.0004
1.4E-05	0.0027	0.00024
1.4E-05	0.00295	0.00042
1.4E-05	0.00172	0.00109
1.4E-05	0.00267	0.00101
1.4E-05	0.00223	0.00031
1.4E-05	0.00234	-5E-05
1.4E-05	0.00266	-0.0004
1.4E-05	0.00182	0.00052
1.4E-05	0.00255	0.00137

1.4E-05	0.00295	0.00033
1.4E-05	0.00216	0.00049
1.4E-05	0.00202	0.00055
1.5E-05	0.0024	0.00087
1.5E-05	0.00256	0.0015
1.5E-05	0.00206	0.00064
1.5E-05	0.00242	0.00131
1.5E-05	0.00228	0.00075
1.5E-05	0.00208	0.00038
1.5E-05	0.00192	0.00048
1.5E-05	0.00262	0.00097
1.5E-05	0.00255	0.00071
1.5E-05	0.00172	0.00114
1.5E-05	0.00277	0.00082
1.5E-05	0.00197	0.00065
1.5E-05	0.00265	0.00147
1.5E-05	0.00305	0.00074
1.5E-05	0.00297	0.00073
1.5E-05	0.00208	0.00069

Table SI.20: Primary kinetic data for the reaction of *N*-methylsuccinimide (**S20**) with CumO• generated by 355 nm LFP in MeCN (**Figure SI.20**).

**t / s**	**ΔA *N*-methylsuccinimmide (S20)**			
	**0.196M**	**0.98M**	**2.0M**	**3.0M**
-1E-06	-8E-05	-0.0002	7E-05	-0.0001
-1E-06	0.00043	-0.0002	0.00078	0.00051
-9E-07	-1E-05	0.00034	0.00011	0.00036
-9E-07	0.00017	-0.0002	0.00081	-0.0013
-9E-07	-1E-05	-0.0003	0.00041	0.00017
-8E-07	-0.0004	-0.0009	0.00024	-0.0006
-8E-07	-0.0006	0.00039	0.00097	-5E-05
-8E-07	0.0002	0.00031	0.00035	-0.0002
-7E-07	-8E-05	-5E-05	0.00039	-0.0004
-7E-07	0.00014	-0.0003	0.00013	-0.0008
-7E-07	-0.0002	-0.0003	0.0006	0.00026
-6E-07	-0.0004	-0.0003	0.0007	-0.0002
-6E-07	-0.0006	-0.0004	0.00045	-0.0005
-6E-07	-0.0005	-0.0004	0.00013	0.00016
-5E-07	-0.0001	0.00054	0.00054	0.00027
-5E-07	-0.0006	-0.0001	0.00057	6.6E-05
-5E-07	-0.0002	-9E-05	-5E-05	-0.0001
-4E-07	-1E-05	0.00042	0.00076	1.2E-05
-4E-07	-0.0005	-3E-05	0.00104	0.00014
-4E-07	-0.0006	-0.0008	0.00072	0.00052
-4E-07	6.9E-05	-8E-05	0.00117	0.00035
-3E-07	-0.0002	0.00013	-0.0003	2.1E-05
-3E-07	-0.0005	0.00042	0.00048	-0.0001
-3E-07	-6E-05	-7E-05	-0.0006	0.00046
-2E-07	-0.0001	-0.0003	-0.0001	-2E-05
-2E-07	6.6E-05	-0.0001	0.00055	0.00031
-2E-07	-4E-06	-0.0001	0.00033	-0.0004
-1E-07	-0.0003	-0.0004	9.9E-05	0.00044
-1E-07	-0.0002	-0.0008	0.00048	0.00041
-6E-08	-8E-05	0.00016	0.00067	-0.0007
-3E-08	-0.0002	0.00043	0.00062	-0.0007
-2E-10	-0.0026	-0.0035	-0.003	-0.0034
3.2E-08	-0.0099	-0.0188	-0.0174	-0.0164
6.4E-08	0.02477	0.01876	0.01048	0.00062
9.6E-08	0.02909	0.02933	0.02909	0.02881
1.3E-07	0.02972	0.02851	0.03156	0.03029
1.6E-07	0.02782	0.02723	0.02968	0.0279
1.9E-07	0.02588	0.02698	0.02828	0.0272
2.2E-07	0.02586	0.02673	0.02746	0.02766
2.6E-07	0.02539	0.02545	0.02628	0.02562
2.9E-07	0.02417	0.02384	0.02563	0.02438
3.2E-07	0.02384	0.02424	0.02425	0.02302
3.5E-07	0.02335	0.02273	0.02317	0.02162
3.8E-07	0.02228	0.02202	0.02319	0.02062
4.2E-07	0.02249	0.02135	0.02141	0.02018
4.5E-07	0.02163	0.02033	0.02058	0.02042
4.8E-07	0.02087	0.02103	0.0211	0.02
5.1E-07	0.01982	0.01972	0.01982	0.01907
5.4E-07	0.0208	0.01935	0.01979	0.01879

5.8E-07	0.01912	0.01926	0.01875	0.01739
6.1E-07	0.01867	0.01868	0.01804	0.01682
6.4E-07	0.01782	0.01734	0.01722	0.01689
6.7E-07	0.01758	0.01723	0.01733	0.01656
7E-07	0.01694	0.01747	0.01698	0.01551
7.4E-07	0.01691	0.01617	0.01623	0.01493
7.7E-07	0.01628	0.01654	0.01603	0.01462
8E-07	0.01671	0.01596	0.01523	0.01403
8.3E-07	0.01625	0.0153	0.01505	0.01431
8.6E-07	0.01526	0.01456	0.01498	0.01313
9E-07	0.01511	0.01366	0.01433	0.01287
9.3E-07	0.01416	0.01361	0.01403	0.01283
9.6E-07	0.01467	0.01388	0.01287	0.01261
9.9E-07	0.01481	0.01286	0.01325	0.01149
1E-06	0.0144	0.01308	0.01323	0.01095
1.1E-06	0.01309	0.01283	0.01317	0.01085
1.1E-06	0.01262	0.01289	0.01296	0.01057
1.1E-06	0.01285	0.01083	0.01235	0.01125
1.2E-06	0.0122	0.01174	0.01241	0.01055
1.2E-06	0.01242	0.0115	0.01158	0.01098
1.2E-06	0.01239	0.01161	0.01167	0.01019
1.2E-06	0.01122	0.01102	0.01112	0.01029
1.3E-06	0.01158	0.01089	0.01178	0.00951
1.3E-06	0.01037	0.01031	0.01135	0.00966
1.3E-06	0.01139	0.01056	0.0109	0.00889
1.4E-06	0.01048	0.01026	0.0104	0.0093
1.4E-06	0.01111	0.00992	0.01065	0.00925
1.4E-06	0.01033	0.00916	0.00969	0.00904
1.5E-06	0.01027	0.00899	0.00939	0.00851
1.5E-06	0.00982	0.00867	0.00997	0.00913
1.5E-06	0.00941	0.00896	0.00936	0.00825
1.6E-06	0.00955	0.00922	0.00876	0.0087
1.6E-06	0.0088	0.00946	0.00857	0.00852
1.6E-06	0.00945	0.00845	0.00927	0.00844
1.7E-06	0.00889	0.0087	0.00895	0.00828
1.7E-06	0.00912	0.00822	0.00903	0.00777
1.7E-06	0.00863	0.00799	0.0082	0.00733
1.8E-06	0.00766	0.00832	0.00845	0.00758
1.8E-06	0.00792	0.008	0.00848	0.00757
1.8E-06	0.00782	0.00742	0.00816	0.00579
1.9E-06	0.00758	0.0068	0.00762	0.00671
1.9E-06	0.00796	0.00649	0.00704	0.00695
1.9E-06	0.00731	0.00784	0.00757	0.0069
2E-06	0.00769	0.00649	0.00788	0.00703
2E-06	0.00633	0.00619	0.0075	0.00658
2E-06	0.00736	0.00702	0.0072	0.00641
2E-06	0.00632	0.00658	0.00745	0.00607
2.1E-06	0.00664	0.00637	0.00694	0.0063
2.1E-06	0.00689	0.00618	0.00721	0.00586
2.1E-06	0.00588	0.00612	0.0067	0.0061
2.2E-06	0.00679	0.00588	0.00687	0.00617
2.2E-06	0.00656	0.00647	0.00675	0.00525
2.2E-06	0.00576	0.00556	0.00686	0.00612
2.3E-06	0.00611	0.00573	0.00662	0.00571

2.3E-06	0.00525	0.00548	0.00649	0.00509
2.3E-06	0.00603	0.00558	0.00612	0.00532
2.4E-06	0.00608	0.00533	0.00604	0.00566
2.4E-06	0.00547	0.00527	0.00518	0.00517
2.4E-06	0.00525	0.00494	0.00603	0.00461
2.5E-06	0.00512	0.00491	0.00543	0.00501
2.5E-06	0.00455	0.005	0.00516	0.00525
2.5E-06	0.00569	0.00485	0.00642	0.00454
2.6E-06	0.00501	0.00473	0.00566	0.00433
2.6E-06	0.00476	0.00448	0.00578	0.0042
2.6E-06	0.00511	0.00407	0.00538	0.00464
2.7E-06	0.00512	0.0045	0.00526	0.00492
2.7E-06	0.00499	0.00456	0.0043	0.00401
2.7E-06	0.00499	0.00535	0.00519	0.00493
2.8E-06	0.0043	0.0049	0.00526	0.00582
2.8E-06	0.00416	0.00418	0.0044	0.00365
2.8E-06	0.00439	0.00417	0.00528	0.00422
2.8E-06	0.00405	0.0039	0.00479	0.00451
2.9E-06	0.0041	0.00372	0.00488	0.00509
2.9E-06	0.00381	0.00419	0.00466	0.00426
2.9E-06	0.00419	0.00342	0.00438	0.00382
3E-06	0.00442	0.00327	0.00397	0.00404
3E-06	0.00356	0.00305	0.00466	0.00439
3E-06	0.00416	0.00284	0.00322	0.00435
3.1E-06	0.00387	0.00381	0.00493	0.00385
3.1E-06	0.00325	0.00283	0.00448	0.00333
3.1E-06	0.00436	0.00327	0.00393	0.00294
3.2E-06	0.0036	0.00291	0.00411	0.00315
3.2E-06	0.00334	0.00335	0.00439	0.00363
3.2E-06	0.00336	0.00284	0.0043	0.00355
3.3E-06	0.0031	0.00319	0.00329	0.00351
3.3E-06	0.0039	0.00274	0.00342	0.00303
3.3E-06	0.00288	0.00396	0.00435	0.00237
3.4E-06	0.00306	0.00335	0.00297	0.00308
3.4E-06	0.00315	0.00305	0.0032	0.00306
3.4E-06	0.00384	0.00291	0.00421	0.00346
3.5E-06	0.00329	0.00237	0.00373	0.00352
3.5E-06	0.00378	0.00273	0.0039	0.00294
3.5E-06	0.00205	0.00222	0.00402	0.00262
3.6E-06	0.00217	0.00209	0.00352	0.00279
3.6E-06	0.00206	0.0026	0.00391	0.00331
3.6E-06	0.0027	0.00284	0.00344	0.00424
3.6E-06	0.00248	0.00291	0.00327	0.00274
3.7E-06	0.00256	0.00233	0.0038	0.00302
3.7E-06	0.00269	0.00254	0.00341	0.00392
3.7E-06	0.0024	0.00257	0.00343	0.00269
3.8E-06	0.00182	0.00232	0.00304	0.00313
3.8E-06	0.00214	0.00282	0.00313	0.00299
3.8E-06	0.00252	0.00201	0.00371	0.00297
3.9E-06	0.00218	0.00199	0.00352	0.00317
3.9E-06	0.00281	0.00197	0.00214	0.00292
3.9E-06	0.00213	0.00239	0.00272	0.00323
4E-06	0.00253	0.00228	0.00325	0.00258
4E-06	0.00208	0.00198	0.00311	0.00279

4E-06	0.00162	0.0017	0.00345	0.0022
4.1E-06	0.00237	0.00177	0.0019	0.00268
4.1E-06	0.0015	0.00241	0.00285	0.00199
4.1E-06	0.00201	0.00177	0.00358	0.00242
4.2E-06	0.00186	0.00236	0.00316	0.00276
4.2E-06	0.00213	0.00189	0.00249	0.00232
4.2E-06	0.00166	0.00291	0.00355	0.00236
4.3E-06	0.00182	0.00209	0.00283	0.00333
4.3E-06	0.00211	0.00261	0.0032	0.00235
4.3E-06	0.00162	0.00178	0.00311	0.00328
4.4E-06	0.00152	0.00154	0.00225	0.00273
4.4E-06	0.00176	0.00185	0.00352	0.00224
4.4E-06	0.0015	0.00189	0.00318	0.00205
4.4E-06	0.00187	0.00157	0.00341	0.00202
4.5E-06	0.00225	0.00166	0.00289	0.00317
4.5E-06	0.00192	0.00152	0.00336	0.00261
4.5E-06	0.00133	0.00186	0.00277	0.0022
4.6E-06	0.00089	0.00224	0.00224	0.00181
4.6E-06	0.00159	0.00143	0.00234	0.00168
4.6E-06	0.00093	0.00099	0.0022	0.00283
4.7E-06	0.00111	0.00196	0.00264	0.00222
4.7E-06	0.00139	0.00265	0.00265	0.00193
4.7E-06	0.00155	0.00156	0.00244	0.00233
4.8E-06	0.00146	0.00165	0.00256	0.00259
4.8E-06	0.00198	0.00205	0.00264	0.00221
4.8E-06	0.00136	0.00171	0.00313	0.00254
4.9E-06	0.00168	0.00175	0.00222	0.00238
4.9E-06	0.00119	0.00094	0.00252	0.00184
4.9E-06	0.0006	0.00152	0.00313	0.00185
5E-06	0.00139	0.00105	0.00297	0.00233
5E-06	0.00127	0.00108	0.00293	0.00171
5E-06	0.00094	0.00138	0.00268	0.00233
5.1E-06	0.00117	0.00173	0.00209	0.00215
5.1E-06	0.00091	0.00175	0.00177	0.00244
5.1E-06	0.00097	0.00144	0.00217	0.00276
5.2E-06	0.00154	0.00198	0.00236	0.00217
5.2E-06	0.00129	0.00039	0.00142	0.00182
5.2E-06	0.00121	0.00018	0.00203	0.00202
5.2E-06	0.00089	0.00174	0.00281	0.0019
5.3E-06	0.00136	0.00164	0.00259	0.00228
5.3E-06	0.00175	0.00176	0.00245	0.00215
5.3E-06	0.00123	0.00132	0.00186	0.00226
5.4E-06	0.00115	0.00155	0.00158	0.00209
5.4E-06	0.00123	0.0009	0.00147	0.00204
5.4E-06	0.0014	0.00164	0.00127	0.00264
5.5E-06	0.0002	0.00104	0.0021	0.00197
5.5E-06	0.00156	0.00125	0.00233	0.00197
5.5E-06	0.00137	0.00111	0.00184	0.00196
5.6E-06	0.001	0.00159	0.00198	0.00167
5.6E-06	0.00079	0.0012	0.00188	0.00231
5.6E-06	0.00127	0.00106	0.00253	0.00224
5.7E-06	0.00084	0.00163	0.00181	0.00214
5.7E-06	0.0009	0.0012	0.00148	0.00193
5.7E-06	0.0015	0.00149	0.00187	0.00222

5.8E-06	0.00117	0.00158	0.00167	0.00195
5.8E-06	0.00123	0.00153	0.00076	0.0023
5.8E-06	0.00063	0.00136	0.00223	0.00219
5.9E-06	0.00086	0.00154	0.00244	0.00215
5.9E-06	0.00027	0.00126	0.00147	0.0017
5.9E-06	0.00084	0.00123	0.00163	0.00236
6E-06	1.9E-05	0.00152	0.00191	0.00181
6E-06	0.00042	0.0013	0.00242	0.00222
6E-06	0.00073	0.00101	0.00306	0.00239
6E-06	0.00073	0.00211	0.0013	0.00263
6.1E-06	0.0007	0.00178	0.00196	0.00229
6.1E-06	0.00026	0.00167	0.00247	0.00265
6.1E-06	-0.0001	0.00113	0.00127	0.00212
6.2E-06	0.00042	0.00144	0.00233	0.00186
6.2E-06	0.00038	0.00123	0.00301	0.00222
6.2E-06	0.00044	0.00112	0.00218	0.00247
6.3E-06	0.00046	0.00017	0.0022	0.00229
6.3E-06	0.00026	0.00095	0.00294	0.00201
6.3E-06	0.00043	0.00111	0.00228	0.00261
6.4E-06	0.0008	0.00048	0.00135	0.002
6.4E-06	-0.0001	0.00112	0.00108	0.00212
6.4E-06	0.00108	0.00081	0.00233	0.00229
6.5E-06	0.00019	0.00063	0.00188	0.00176
6.5E-06	0.00075	0.00115	0.00212	0.0016
6.5E-06	0.00073	0.00024	0.00225	0.00246
6.6E-06	0.00064	0.00103	0.0025	0.00247
6.6E-06	0.00059	0.00079	0.0031	0.00196
6.6E-06	0.00084	0.00129	0.00295	0.00202
6.7E-06	-1E-05	0.00142	0.00171	0.00192
6.7E-06	0.0007	0.00063	0.00209	0.00222
6.7E-06	0.00028	0.00171	0.00191	0.00213
6.8E-06	0.00071	0.00095	0.00243	0.00227
6.8E-06	5.8E-05	0.00086	0.00146	0.0018
6.8E-06	0.00134	0.00058	0.00167	0.0024
6.8E-06	0.0015	0.00161	0.00176	0.00253
6.9E-06	-8E-06	0.00103	0.00188	0.00269
6.9E-06	0.00059	0.00134	0.00144	0.00265
6.9E-06	0.001	0.00184	0.00158	0.00145
7E-06	0.00065	0.00142	0.00223	0.00164
7E-06	0.00097	0.00101	0.00187	0.00176
7E-06	0.00033	0.00081	0.00134	0.00228
7.1E-06	0.00067	0.00177	0.00178	0.00226
7.1E-06	0.00059	0.0006	0.00253	0.00229
7.1E-06	0.00025	0.00071	0.00117	0.00104
7.2E-06	0.00028	0.00066	0.00246	0.00152
7.2E-06	0.00102	0.00209	0.00267	0.00212
7.2E-06	0.00075	0.00072	0.00272	0.0019
7.3E-06	0.0004	0.00131	0.00292	0.00207
7.3E-06	0.00032	0.0013	0.00183	0.00185
7.3E-06	0.00074	0.00143	0.00221	0.00145
7.4E-06	0.00017	0.00105	0.00241	0.00153
7.4E-06	0.00044	0.00096	0.00145	0.00198
7.4E-06	0.00048	0.00092	0.00146	0.00203
7.5E-06	0.00056	0.00089	0.002	0.00179

7.5E-06	0.00016	0.00098	0.00116	0.00237
7.5E-06	0.00013	0.00128	0.00169	0.00191
7.6E-06	-0.0006	0.00042	0.00167	0.00319
7.6E-06	0.00116	0.00114	0.00214	0.00226
7.6E-06	0.00099	0.00132	0.0013	0.00185
7.6E-06	0.00026	0.00099	0.00216	0.00209
7.7E-06	0.0003	0.00068	0.00171	0.00154
7.7E-06	0.0007	0.00124	0.00187	0.00231
7.7E-06	0.00064	0.00115	0.00133	0.00206
7.8E-06	0.00084	0.0012	0.00109	0.00247
7.8E-06	0.0004	0.00117	0.00193	0.00242
7.8E-06	0.00065	0.00036	0.00117	0.00164
7.9E-06	0.00102	0.0011	0.00172	0.00264
7.9E-06	8.6E-05	0.00046	0.00141	0.00184
7.9E-06	0.00027	0.00046	0.00099	0.00161
8E-06	-0.0002	0.00136	0.00143	0.00193
8E-06	-0.0004	0.00099	0.00164	0.00236
8E-06	2.7E-05	0.001	0.00152	0.00142
8.1E-06	0.00062	0.00058	0.00172	0.00248
8.1E-06	0.00053	0.00094	0.00262	0.00156
8.1E-06	-0.0003	0.00172	0.00163	0.00169
8.2E-06	0.00019	0.00028	0.00155	0.00161
8.2E-06	0.00054	0.00094	0.00227	0.00161
8.2E-06	0.00023	0.00083	0.00287	0.00203
8.3E-06	0.00058	0.00115	0.00176	0.00206
8.3E-06	0.00083	0.00132	0.00155	0.00117
8.3E-06	-0.0004	0.00076	0.00186	0.00207
8.4E-06	-3E-05	0.00059	0.00206	0.00158
8.4E-06	0.00069	0.00136	0.00163	0.00181
8.4E-06	0.00062	0.00185	0.00052	0.00231
8.4E-06	0.00063	0.00042	0.00109	0.00195
8.5E-06	0.00117	0.00072	0.00115	0.00236
8.5E-06	-0.0006	0.00029	0.00134	0.00214
8.5E-06	0.0003	0.00066	0.00145	0.00186
8.6E-06	-0.0002	0.00081	0.00149	0.00191
8.6E-06	0.00078	0.00047	0.00103	0.0017
8.6E-06	0.00015	0.00072	0.00125	0.00236
8.7E-06	0.00087	0.00105	0.00174	0.00167
8.7E-06	0.00081	0.00087	0.00212	0.00194
8.7E-06	-1E-06	0.00086	0.00264	0.00163
8.8E-06	0.0001	0.00061	0.00129	0.00287
8.8E-06	-4E-05	0.00123	0.00048	0.00218
8.8E-06	0.00074	0.00149	0.00105	0.00141
8.9E-06	0.00066	0.00088	0.00143	0.00179
8.9E-06	0.00058	0.00074	0.00085	0.00143
8.9E-06	0.00084	0.00127	0.00195	0.0017
9E-06	0.00082	0.00083	0.00155	0.00177
9E-06	-1E-05	0.00125	0.00126	0.00256
9E-06	0.00025	0.00091	0.00078	0.00236
9.1E-06	-0.0005	0.0007	0.00161	0.00163
9.1E-06	-0.0004	0.00081	0.00225	0.00203
9.1E-06	-0.0003	0.0007	0.00302	0.00171
9.2E-06	0.00078	0.00084	0.00217	0.00171
9.2E-06	0.00032	0.00081	0.00152	0.00203

9.2E-06	0.00027	0.00113	0.00158	0.00164
9.2E-06	0.00063	0.00058	0.00164	0.0021
9.3E-06	-0.0001	-5E-05	0.00137	0.00183
9.3E-06	0.00076	0.00129	0.00225	0.00302
9.3E-06	0.00032	0.00108	0.00235	0.00323
9.4E-06	-0.0002	0.00036	0.00176	0.0028
9.4E-06	5.3E-05	0.00034	0.00239	0.00221
9.4E-06	-0.0002	0.00135	0.0013	0.00249
9.5E-06	0.00011	0.00089	0.00133	0.0021
9.5E-06	4.5E-05	0.00034	0.00199	0.0022
9.5E-06	0.00012	0.00058	0.0017	0.00174
9.6E-06	0.00013	0.00042	0.00183	0.00188
9.6E-06	0.00025	0.00058	0.00172	0.00094
9.6E-06	3.8E-05	-0.0001	0.00103	0.00122
9.7E-06	9.4E-05	0.00075	0.00153	0.00238
9.7E-06	-0.0001	0.00015	0.0025	0.00199
9.7E-06	-0.0009	0.00051	0.00161	0.00143
9.8E-06	0.00094	0.0005	0.00181	0.00151
9.8E-06	3E-05	0.00105	0.00202	0.00113
9.8E-06	0.00057	0.00058	0.00179	0.00241
9.9E-06	0.00037	0.00037	0.00167	0.00255
9.9E-06	4.8E-05	0.00046	0.00165	0.00196
9.9E-06	-0.0006	0.0009	0.00169	0.00274
1E-05	-0.0005	0.001	0.00122	0.0013
1E-05	-0.0005	0.00031	0.00234	0.00221
1E-05	0.00062	0.00099	0.00201	0.00193
1E-05	0.00037	0.00054	0.00158	0.00131
1E-05	-0.0001	0.00054	0.00155	0.00119
1E-05	0.00029	0.00025	0.00219	0.00188
1E-05	0.00042	0.00121	0.00218	0.00257
1E-05	0.00032	0.00077	0.00132	0.00162
1E-05	-0.0002	0.00076	0.00164	0.00183
1E-05	0.0001	0.0007	0.00205	0.00166
1E-05	0.00019	0.00081	0.00234	0.00074
1E-05	0.00022	0.00062	0.00213	0.00172
1E-05	-0.0002	0.00053	0.00154	0.00118
1E-05	0.00043	-0.0002	0.00144	0.00137
1E-05	-0.0001	0.00069	0.002	0.00161
1E-05	0.00014	0.00057	0.00144	0.00179
1E-05	0.00037	0.00018	0.00169	0.00272
1.1E-05	0.00057	-0.0001	0.0014	0.00142
1.1E-05	0.00056	0.0016	0.00161	0.00139
1.1E-05	-0.0002	0.00038	0.00147	0.00207
1.1E-05	-0.0001	-7E-05	0.00172	0.00159
1.1E-05	-0.0003	0.00058	0.00198	0.00235
1.1E-05	0.00017	9.9E-05	0.00347	0.00192
1.1E-05	0.00055	-0.0002	0.0017	0.0017
1.1E-05	0.00037	0.00019	0.00144	0.00198
1.1E-05	0.00015	0.0007	0.00186	0.00205
1.1E-05	0.00029	0.00021	0.00158	0.00198
1.1E-05	-4E-05	7.4E-05	0.00168	0.002
1.1E-05	0.00092	0.00033	0.00116	0.00138
1.1E-05	0.00055	0.00105	0.00121	0.00118
1.1E-05	0.00022	0.0009	0.00183	0.00178

1.1E-05	8.5E-05	-7E-05	0.00176	0.00236
1.1E-05	0.00038	0.00046	0.00158	0.00263
1.1E-05	-0.0006	0.00049	0.00132	0.00221
1.1E-05	6.4E-05	0.00072	0.00129	0.00184
1.1E-05	7.5E-05	0.0009	0.00204	0.00249
1.1E-05	-5E-05	5.3E-05	0.00214	0.00128
1.1E-05	-0.0002	5.1E-06	0.00168	0.00238
1.1E-05	-0.0003	0.00025	0.0018	0.00196
1.1E-05	0.00023	0.00073	0.0019	0.00126
1.1E-05	0.00015	0.00038	0.00158	0.00149
1.1E-05	-4E-05	1.1E-05	0.00147	0.00224
1.1E-05	-0.0002	0.00013	0.00161	0.00257
1.1E-05	-9E-05	0.00027	0.00121	0.00165
1.1E-05	0.00077	0.00034	0.00091	0.00208
1.1E-05	0.00064	0.00104	0.00104	0.00144
1.1E-05	-0.001	0.00057	0.00185	0.00216
1.1E-05	0.00043	0.00135	0.00124	0.00181
1.1E-05	-0.0008	0.00033	0.00149	0.00235
1.2E-05	-0.0006	0.00104	0.00153	0.00199
1.2E-05	-2E-05	0.00033	0.00135	0.00246
1.2E-05	-6E-05	9.9E-05	0.00134	0.00089
1.2E-05	-0.0003	0.00075	0.00143	0.00228
1.2E-05	-0.0002	0.00035	0.00176	0.00089
1.2E-05	0.00028	0.00063	0.0015	0.00124
1.2E-05	0.00034	-0.0005	0.00215	0.00168
1.2E-05	0.00031	0.00146	0.00204	0.00171
1.2E-05	-0.0002	0.00039	0.00099	0.00217
1.2E-05	0.00029	2.1E-06	0.00197	0.00194
1.2E-05	0.00036	0.00011	0.00124	0.003
1.2E-05	0.00017	0.00105	0.00147	0.00203
1.2E-05	0.00021	0.00086	0.00194	0.00166
1.2E-05	-0.0006	0.001	0.00206	0.00269
1.2E-05	0.0003	0.00081	0.0012	0.00075
1.2E-05	3.7E-05	0.00017	0.00135	0.00195
1.2E-05	0.00012	0.00035	0.00163	0.00141
1.2E-05	0.00032	0.00137	0.0007	0.00215
1.2E-05	-0.0003	0.0008	0.00112	0.00149
1.2E-05	-0.0002	0.00086	0.00156	0.00197
1.2E-05	0.00038	0.00036	0.00131	0.00183
1.2E-05	5.1E-05	0.00146	0.00188	0.00251
1.2E-05	-0.0006	0.0011	0.00104	0.0017
1.2E-05	-0.0004	0.00045	0.0006	0.00239
1.2E-05	0.00035	0.00052	0.00162	0.00155
1.2E-05	0.00013	0.00034	0.00141	0.00138
1.2E-05	-0.0002	0.0006	0.00138	0.00149
1.2E-05	0.00025	0.00063	0.00164	0.00107
1.2E-05	-3E-05	0.00051	0.0017	0.00123
1.2E-05	0.00063	0.00047	0.00158	0.00176
1.2E-05	-3E-05	0.00074	0.00146	0.00235
1.3E-05	1.8E-05	0.00147	0.00135	0.00238
1.3E-05	0.00015	0.00072	0.00165	0.00098
1.3E-05	-0.0002	0.00087	0.00149	0.00127
1.3E-05	0.00012	-0.0001	0.00226	0.00169
1.3E-05	-0.0004	0.00064	0.00151	0.00162

1.3E-05	0.00094	0.00041	0.00167	0.0017
1.3E-05	0.00086	0.00088	0.00209	0.00248
1.3E-05	-0.0001	0.00012	0.00121	0.00109
1.3E-05	0.00075	0.0015	0.00182	0.00158
1.3E-05	0.0002	0.00121	0.00142	0.00162
1.3E-05	-0.0002	0.00061	0.0021	0.00117
1.3E-05	0.00011	0.00104	0.00231	0.00223
1.3E-05	0.00053	0.00021	0.00139	0.00221
1.3E-05	0.00016	0.00049	0.00178	0.00229
1.3E-05	0.00063	-0.0002	0.00169	0.00158
1.3E-05	0.00019	0.00068	0.00168	0.00102
1.3E-05	-0.0004	0.00071	0.00171	0.00169
1.3E-05	1.7E-05	0.00045	0.00076	0.00188
1.3E-05	-0.0002	0.00113	0.00161	0.0016
1.3E-05	0.00015	0.00022	0.00174	0.00198
1.3E-05	0.00056	0.00042	0.00161	0.0013
1.3E-05	0.00014	0.0013	0.00213	0.00089
1.3E-05	0.00032	0.00124	0.00119	0.00194
1.3E-05	0.00064	0.00017	0.0012	0.0024
1.3E-05	-5E-05	0.00068	0.00174	0.00195
1.3E-05	-0.0004	8E-05	0.00186	0.00207
1.3E-05	0.00027	0.0007	0.00091	0.00166
1.3E-05	5.2E-05	-0.0004	0.00136	0.0015
1.3E-05	-0.0003	-1E-05	0.00195	0.0012
1.3E-05	-0.0004	0.00056	0.00193	0.00151
1.3E-05	-8E-05	0.00051	0.00139	0.00176
1.4E-05	-0.0004	0.0004	0.00159	0.00229
1.4E-05	-0.0005	-0.0004	0.00159	0.00169
1.4E-05	-0.0001	0.0008	0.00176	0.00231
1.4E-05	0.00045	0.00086	0.0014	0.00199
1.4E-05	-0.0003	0.00137	0.0015	0.00129
1.4E-05	-0.0006	0.00108	0.00201	0.00197
1.4E-05	0.00063	0.00069	0.00212	0.00161
1.4E-05	0.00026	0.0009	0.00117	0.00211
1.4E-05	-1E-04	0.00113	0.00146	0.00186
1.4E-05	-0.0002	0.00037	0.00188	0.00114
1.4E-05	0.00059	0.00072	0.00125	0.00148
1.4E-05	-0.0002	0.00173	0.00207	0.00145
1.4E-05	-0.0004	0.00059	0.00185	0.00222
1.4E-05	0.00039	0.0003	0.00156	0.00196
1.4E-05	0.00056	0.00072	0.00174	0.00131
1.4E-05	0.00014	0.00068	0.00175	0.003
1.4E-05	-0.0006	0.00027	0.00172	0.0016
1.4E-05	0.00015	0.00011	0.00141	0.00137
1.4E-05	0.0008	0.00073	0.00186	0.00132
1.4E-05	0.00101	0.00119	0.00153	0.00087
1.4E-05	9.7E-05	0.00125	0.00133	0.00167
1.4E-05	-0.0009	0.00115	0.00142	0.00243
1.4E-05	-0.0004	0.00068	0.00141	0.00137
1.4E-05	0.00015	0.00058	0.00178	0.00052
1.4E-05	0.00065	0.00039	0.00129	0.00119
1.4E-05	-0.0004	0.00075	0.00152	0.00189
1.4E-05	-0.0003	0.00109	0.00193	0.00142
1.4E-05	-9E-05	0.00086	0.00138	0.001

1.4E-05	-0.0002	0.00096	0.00124	0.00119
1.4E-05	0.00027	0.00103	0.00169	0.00172
1.4E-05	0.00051	0.0011	0.00119	0.0011
1.5E-05	0.00026	0.00024	0.00158	0.00121
1.5E-05	0.00048	0.0009	0.0013	0.00151
1.5E-05	-0.0004	0.00018	0.00146	0.00155
1.5E-05	0.00033	0.0003	0.00124	0.0014
1.5E-05	0.0001	0.00093	0.002	0.00274
1.5E-05	0.00018	0.00114	0.00183	0.00218
1.5E-05	0.00086	0.00085	0.0017	0.00076
1.5E-05	-0.0006	0.00112	0.00185	0.00115
1.5E-05	-0.0003	0.00093	0.00125	0.00075
1.5E-05	0.00052	0.00167	0.00128	0.00139
1.5E-05	0.00031	0.00025	0.00191	0.00187
1.5E-05	-0.0005	0.00081	0.00206	0.00103
1.5E-05	5.7E-05	0.00122	0.00146	0.00196
1.5E-05	0.00067	0.00041	0.00165	0.00193
1.5E-05	0.00031	0.0007	0.00176	0.00149
1.5E-05	0.00013	-2E-05	0.00128	0.00182

Table SI.21: Primary kinetic data for the reaction of ethylene carbonate (**S21**) with CumO$^\bullet$ generated by 355 nm LFP in MeCN (**Figure SI.21**).

**t / s**	**ΔA ethylene carbonate (S21)**		
	**0.25 M**	**1.125 M**	**2.5 M**
-9.9E-07	6.1E-04	-3.4E-04	1.1E-04
-9.6E-07	3.7E-04	-3.5E-04	1.1E-04
-9.3E-07	3.0E-04	-5.1E-04	1.3E-04
-9.0E-07	2.5E-04	-1.6E-05	-5.6E-04
-8.6E-07	9.0E-04	-2.7E-04	-3.3E-04
-8.3E-07	9.6E-04	-5.9E-04	-6.1E-04
-8.0E-07	3.1E-04	-4.1E-04	-3.6E-05
-7.7E-07	2.8E-04	1.4E-04	9.8E-06
-7.4E-07	2.1E-04	-9.6E-04	1.1E-04
-7.0E-07	2.5E-04	-1.0E-03	-1.7E-04
-6.7E-07	3.1E-05	-2.7E-04	-2.2E-04
-6.4E-07	2.1E-04	-2.6E-04	-5.1E-05
-6.1E-07	3.9E-04	-7.9E-04	-1.9E-05
-5.8E-07	-8.8E-06	-4.2E-04	-5.0E-04
-5.4E-07	4.9E-04	6.2E-05	-3.1E-04
-5.1E-07	5.8E-04	2.9E-04	9.0E-05
-4.8E-07	1.3E-04	-7.8E-05	-4.4E-05
-4.5E-07	2.7E-04	-2.2E-04	-5.9E-04
-4.2E-07	9.9E-04	1.6E-05	-4.2E-04
-3.8E-07	6.3E-04	-2.9E-04	-2.7E-04
-3.5E-07	4.1E-04	-7.5E-04	-4.1E-04
-3.2E-07	6.3E-04	-1.4E-04	-6.3E-04
-2.9E-07	1.8E-04	-1.0E-04	-2.9E-04
-2.6E-07	4.1E-04	2.0E-04	-5.4E-05
-2.2E-07	3.8E-04	-5.6E-04	-1.7E-04
-1.9E-07	5.2E-04	8.6E-05	-3.3E-04
-1.6E-07	4.5E-04	-1.1E-04	-1.6E-04
-1.3E-07	7.0E-04	3.1E-04	-4.1E-04
-9.6E-08	2.8E-04	-9.6E-05	-3.7E-05
-6.4E-08	8.3E-05	-5.0E-05	-2.9E-04
-3.2E-08	3.3E-04	6.6E-05	-3.7E-04
-1.9E-10	-2.1E-03	-2.9E-03	-3.0E-03
3.2E-08	-3.4E-03	-8.0E-03	-8.8E-03
6.4E-08	1.9E-02	1.6E-02	1.5E-02
9.6E-08	1.9E-02	1.7E-02	1.6E-02
1.3E-07	1.9E-02	1.7E-02	1.6E-02
1.6E-07	1.9E-02	1.6E-02	1.5E-02
1.9E-07	1.8E-02	1.6E-02	1.6E-02
2.2E-07	1.8E-02	1.5E-02	1.5E-02
2.6E-07	1.7E-02	1.5E-02	1.4E-02
2.9E-07	1.6E-02	1.5E-02	1.4E-02
3.2E-07	1.6E-02	1.4E-02	1.3E-02
3.5E-07	1.6E-02	1.4E-02	1.3E-02
3.8E-07	1.6E-02	1.4E-02	1.3E-02
4.2E-07	1.5E-02	1.3E-02	1.2E-02
4.5E-07	1.5E-02	1.3E-02	1.3E-02
4.8E-07	1.4E-02	1.2E-02	1.2E-02
5.1E-07	1.4E-02	1.2E-02	1.2E-02
5.4E-07	1.3E-02	1.2E-02	1.1E-02

5.8E-07	1.3E-02	1.2E-02	1.1E-02
6.1E-07	1.3E-02	1.2E-02	1.0E-02
6.4E-07	1.2E-02	1.1E-02	1.0E-02
6.7E-07	1.3E-02	1.1E-02	1.0E-02
7.0E-07	1.3E-02	1.0E-02	9.8E-03
7.4E-07	1.3E-02	1.0E-02	9.8E-03
7.7E-07	1.2E-02	9.6E-03	9.3E-03
8.0E-07	1.2E-02	9.6E-03	9.1E-03
8.3E-07	1.1E-02	9.9E-03	9.2E-03
8.6E-07	1.1E-02	9.8E-03	9.1E-03
9.0E-07	1.1E-02	9.4E-03	8.5E-03
9.3E-07	1.0E-02	9.5E-03	8.7E-03
9.6E-07	1.0E-02	8.9E-03	8.8E-03
9.9E-07	1.0E-02	8.5E-03	7.9E-03
1.0E-06	9.6E-03	8.8E-03	7.5E-03
1.1E-06	1.0E-02	7.9E-03	7.6E-03
1.1E-06	9.5E-03	7.6E-03	7.4E-03
1.1E-06	8.9E-03	8.2E-03	7.4E-03
1.2E-06	8.5E-03	7.5E-03	6.9E-03
1.2E-06	8.8E-03	7.6E-03	7.4E-03
1.2E-06	8.8E-03	7.2E-03	7.1E-03
1.3E-06	8.5E-03	7.0E-03	6.3E-03
1.3E-06	7.9E-03	7.4E-03	6.5E-03
1.3E-06	8.3E-03	7.0E-03	6.6E-03
1.3E-06	8.3E-03	7.2E-03	6.2E-03
1.4E-06	7.9E-03	6.2E-03	5.8E-03
1.4E-06	7.2E-03	7.0E-03	6.1E-03
1.4E-06	7.4E-03	6.2E-03	5.9E-03
1.5E-06	7.3E-03	5.9E-03	6.2E-03
1.5E-06	7.6E-03	5.9E-03	5.2E-03
1.5E-06	6.4E-03	5.2E-03	5.5E-03
1.6E-06	6.8E-03	5.7E-03	5.4E-03
1.6E-06	6.4E-03	5.9E-03	5.0E-03
1.6E-06	6.3E-03	5.7E-03	5.2E-03
1.7E-06	6.6E-03	5.4E-03	5.3E-03
1.7E-06	6.1E-03	5.0E-03	4.8E-03
1.7E-06	6.3E-03	5.2E-03	4.8E-03
1.8E-06	6.2E-03	5.6E-03	4.9E-03
1.8E-06	6.0E-03	4.7E-03	4.8E-03
1.8E-06	6.0E-03	5.3E-03	4.7E-03
1.9E-06	5.9E-03	4.7E-03	4.9E-03
1.9E-06	5.2E-03	4.8E-03	4.1E-03
1.9E-06	5.2E-03	4.2E-03	3.9E-03
2.0E-06	5.2E-03	5.0E-03	4.6E-03
2.0E-06	5.2E-03	4.3E-03	3.8E-03
2.0E-06	4.7E-03	3.9E-03	3.9E-03
2.1E-06	5.3E-03	4.1E-03	3.3E-03
2.1E-06	4.6E-03	3.3E-03	3.4E-03
2.1E-06	5.4E-03	4.0E-03	3.6E-03
2.1E-06	5.0E-03	4.1E-03	3.3E-03
2.2E-06	4.7E-03	3.4E-03	3.8E-03
2.2E-06	4.7E-03	4.2E-03	3.1E-03
2.2E-06	5.2E-03	3.5E-03	3.0E-03
2.3E-06	4.5E-03	3.8E-03	3.2E-03

2.3E-06	4.5E-03	3.5E-03	2.9E-03
2.3E-06	4.6E-03	4.0E-03	2.8E-03
2.4E-06	4.2E-03	3.1E-03	2.8E-03
2.4E-06	4.1E-03	3.6E-03	3.2E-03
2.4E-06	4.1E-03	3.5E-03	2.8E-03
2.5E-06	3.9E-03	3.0E-03	2.6E-03
2.5E-06	4.2E-03	3.8E-03	2.6E-03
2.5E-06	3.6E-03	3.5E-03	2.9E-03
2.6E-06	4.1E-03	3.1E-03	3.1E-03
2.6E-06	3.3E-03	3.2E-03	2.6E-03
2.6E-06	2.9E-03	3.7E-03	2.5E-03
2.7E-06	3.7E-03	3.0E-03	2.7E-03
2.7E-06	3.9E-03	3.2E-03	3.0E-03
2.7E-06	3.3E-03	2.2E-03	2.2E-03
2.8E-06	3.5E-03	2.8E-03	2.4E-03
2.8E-06	3.6E-03	3.0E-03	2.1E-03
2.8E-06	3.2E-03	3.0E-03	1.9E-03
2.9E-06	3.5E-03	2.8E-03	2.1E-03
2.9E-06	3.5E-03	1.5E-03	2.1E-03
2.9E-06	2.5E-03	2.6E-03	1.5E-03
2.9E-06	3.1E-03	2.1E-03	1.9E-03
3.0E-06	2.8E-03	2.3E-03	1.8E-03
3.0E-06	3.0E-03	1.9E-03	1.6E-03
3.0E-06	2.4E-03	2.3E-03	2.1E-03
3.1E-06	2.8E-03	2.2E-03	2.1E-03
3.1E-06	2.4E-03	1.2E-03	2.1E-03
3.1E-06	2.0E-03	2.8E-03	1.9E-03
3.2E-06	2.2E-03	2.0E-03	1.9E-03
3.2E-06	2.0E-03	2.0E-03	1.6E-03
3.2E-06	2.9E-03	1.5E-03	2.1E-03
3.3E-06	2.1E-03	2.1E-03	1.6E-03
3.3E-06	2.3E-03	2.0E-03	1.8E-03
3.3E-06	2.3E-03	1.6E-03	2.2E-03
3.4E-06	2.5E-03	2.1E-03	2.0E-03
3.4E-06	2.0E-03	1.5E-03	1.7E-03
3.4E-06	2.2E-03	1.4E-03	1.5E-03
3.5E-06	2.2E-03	1.4E-03	1.7E-03
3.5E-06	1.8E-03	2.1E-03	2.1E-03
3.5E-06	2.0E-03	2.1E-03	1.1E-03
3.6E-06	1.8E-03	1.8E-03	1.4E-03
3.6E-06	1.8E-03	1.5E-03	1.6E-03
3.6E-06	1.6E-03	1.4E-03	1.0E-03
3.7E-06	2.4E-03	1.4E-03	1.1E-03
3.7E-06	2.3E-03	1.1E-03	1.5E-03
3.7E-06	1.6E-03	7.7E-04	1.4E-03
3.7E-06	2.1E-03	1.5E-03	8.2E-04
3.8E-06	2.4E-03	1.9E-03	1.1E-03
3.8E-06	2.0E-03	1.6E-03	1.6E-03
3.8E-06	2.5E-03	1.9E-03	1.1E-03
3.9E-06	2.0E-03	1.6E-03	4.1E-04
3.9E-06	2.3E-03	9.6E-04	1.1E-03
3.9E-06	2.0E-03	1.3E-03	8.1E-04
4.0E-06	2.1E-03	2.0E-03	9.3E-04
4.0E-06	1.7E-03	1.3E-03	8.1E-04

4.0E-06	1.5E-03	1.4E-03	8.2E-04
4.1E-06	1.8E-03	1.2E-03	9.3E-04
4.1E-06	2.0E-03	1.5E-03	1.2E-03
4.1E-06	1.8E-03	1.5E-03	9.0E-04
4.2E-06	1.6E-03	1.3E-03	5.4E-04
4.2E-06	2.4E-03	1.8E-03	6.2E-04
4.2E-06	1.5E-03	1.7E-03	8.9E-04
4.3E-06	1.8E-03	1.4E-03	3.3E-04
4.3E-06	2.1E-03	1.2E-03	7.5E-04
4.3E-06	1.5E-03	1.3E-03	5.9E-04
4.4E-06	1.8E-03	1.4E-03	1.0E-03
4.4E-06	1.7E-03	1.3E-03	9.5E-04
4.4E-06	1.5E-03	1.3E-03	4.1E-04
4.5E-06	1.3E-03	2.8E-04	8.2E-04
4.5E-06	1.8E-03	1.1E-03	8.5E-04
4.5E-06	1.6E-03	4.9E-05	8.1E-04
4.5E-06	7.7E-04	3.5E-04	4.9E-04
4.6E-06	1.2E-03	1.4E-03	8.5E-04
4.6E-06	8.4E-04	6.5E-04	7.5E-04
4.6E-06	1.2E-03	1.3E-03	4.4E-04
4.7E-06	1.6E-03	1.0E-03	9.2E-04
4.7E-06	1.1E-03	1.5E-03	4.7E-04
4.7E-06	1.9E-03	1.8E-03	9.9E-04
4.8E-06	1.5E-03	7.8E-04	6.8E-04
4.8E-06	1.6E-03	2.4E-04	5.8E-04
4.8E-06	9.4E-04	4.2E-04	6.9E-04
4.9E-06	1.6E-03	-3.8E-04	3.4E-04
4.9E-06	8.6E-04	7.7E-04	1.0E-03
4.9E-06	1.6E-03	1.0E-03	8.1E-04
5.0E-06	1.3E-03	7.1E-04	6.0E-04
5.0E-06	1.1E-03	6.6E-04	7.3E-04
5.0E-06	9.4E-04	1.6E-03	5.3E-04
5.1E-06	1.1E-03	1.1E-03	9.2E-04
5.1E-06	4.8E-04	1.1E-03	1.3E-03
5.1E-06	9.4E-04	2.5E-04	7.3E-04
5.2E-06	1.5E-03	6.4E-04	1.1E-03
5.2E-06	9.0E-04	1.2E-03	9.2E-04
5.2E-06	1.4E-03	1.1E-03	8.1E-04
5.3E-06	7.7E-04	1.1E-03	1.1E-03
5.3E-06	9.9E-04	6.5E-04	1.6E-04
5.3E-06	1.1E-03	1.2E-03	6.3E-04
5.3E-06	1.5E-03	1.3E-03	5.8E-04
5.4E-06	1.1E-03	7.9E-04	5.1E-04
5.4E-06	1.3E-03	9.6E-04	7.1E-04
5.4E-06	1.3E-03	-1.0E-05	1.2E-03
5.5E-06	5.0E-04	2.4E-04	6.2E-04
5.5E-06	6.8E-04	1.2E-03	1.0E-03
5.5E-06	6.5E-04	1.3E-03	7.1E-04
5.6E-06	7.4E-04	7.5E-04	7.2E-04
5.6E-06	8.5E-04	1.6E-03	7.1E-04
5.6E-06	1.0E-03	8.9E-04	2.3E-04
5.7E-06	9.7E-04	1.1E-03	1.0E-03
5.7E-06	6.7E-04	1.3E-03	7.3E-04
5.7E-06	3.1E-04	6.8E-04	8.7E-04

5.8E-06	5.2E-04	1.1E-03	8.8E-04
5.8E-06	9.1E-04	5.4E-04	8.1E-04
5.8E-06	1.2E-03	5.9E-04	3.6E-04
5.9E-06	1.0E-03	6.2E-04	1.1E-03
5.9E-06	9.8E-04	3.8E-05	3.1E-04
5.9E-06	1.3E-03	9.1E-04	7.7E-04
6.0E-06	7.8E-04	2.9E-04	4.3E-04
6.0E-06	1.0E-03	6.3E-04	9.5E-04
6.0E-06	1.1E-03	-1.7E-04	1.1E-03
6.1E-06	1.1E-03	3.7E-05	8.6E-04
6.1E-06	6.8E-04	7.6E-04	1.9E-05
6.1E-06	1.3E-03	4.1E-04	7.1E-04
6.1E-06	4.4E-04	4.4E-04	5.2E-04
6.2E-06	6.9E-04	4.0E-04	6.0E-04
6.2E-06	4.8E-04	4.3E-04	3.3E-04
6.2E-06	5.5E-04	9.3E-04	6.9E-04
6.3E-06	9.5E-04	4.1E-04	5.6E-04
6.3E-06	5.5E-04	5.8E-04	4.9E-04
6.3E-06	6.8E-04	3.9E-04	3.8E-04
6.4E-06	6.9E-04	7.0E-04	9.9E-04
6.4E-06	7.8E-04	5.8E-04	3.2E-04
6.4E-06	7.1E-04	5.9E-04	-8.0E-05
6.5E-06	4.1E-04	2.7E-04	4.7E-04
6.5E-06	8.6E-04	6.9E-04	4.5E-04
6.5E-06	9.3E-04	7.9E-04	4.9E-04
6.6E-06	9.1E-04	5.2E-04	9.9E-04
6.6E-06	8.3E-04	-2.6E-04	5.2E-04
6.6E-06	5.6E-04	2.4E-04	2.6E-04
6.7E-06	1.7E-04	5.7E-04	8.2E-04
6.7E-06	1.2E-04	2.6E-04	6.7E-04
6.7E-06	9.2E-04	3.7E-04	5.2E-04
6.8E-06	4.9E-04	5.9E-04	5.3E-04
6.8E-06	6.4E-04	5.2E-04	6.0E-04
6.8E-06	6.0E-04	3.8E-04	1.2E-03
6.9E-06	4.9E-04	1.2E-04	5.6E-04
6.9E-06	5.0E-04	-2.2E-04	2.2E-04
6.9E-06	5.2E-04	6.1E-05	-1.0E-04
6.9E-06	8.1E-04	5.5E-04	2.0E-04
7.0E-06	5.1E-04	1.8E-04	1.9E-04
7.0E-06	4.6E-04	1.2E-03	2.1E-04
7.0E-06	8.5E-04	5.3E-04	3.9E-04
7.1E-06	5.9E-04	8.4E-04	4.1E-04
7.1E-06	7.2E-04	4.5E-04	3.2E-04
7.1E-06	4.0E-04	4.5E-04	1.7E-04
7.2E-06	9.8E-04	4.5E-05	4.0E-04
7.2E-06	7.7E-04	4.8E-04	-1.9E-04
7.2E-06	6.8E-04	-2.5E-06	-8.7E-05
7.3E-06	9.7E-04	3.8E-04	5.4E-04
7.3E-06	1.0E-03	1.7E-05	2.4E-04
7.3E-06	1.1E-03	4.4E-05	4.9E-04
7.4E-06	9.0E-04	1.2E-03	2.3E-04
7.4E-06	1.0E-03	6.1E-04	4.0E-04
7.4E-06	4.8E-04	8.8E-05	5.8E-04
7.5E-06	7.5E-04	9.4E-04	6.9E-04

7.5E-06	4.1E-04	4.2E-04	8.3E-04
7.5E-06	1.2E-03	6.2E-04	5.9E-04
7.6E-06	3.8E-04	6.4E-04	3.1E-04
7.6E-06	2.9E-04	1.2E-04	1.6E-04
7.6E-06	8.8E-04	1.1E-04	9.3E-04
7.7E-06	5.8E-04	9.9E-04	6.6E-04
7.7E-06	7.2E-04	4.1E-04	5.1E-04
7.7E-06	5.5E-04	2.9E-04	5.9E-04
7.7E-06	3.1E-04	3.4E-04	4.2E-04
7.8E-06	8.2E-04	7.9E-04	7.5E-04
7.8E-06	7.4E-04	3.6E-04	9.2E-04
7.8E-06	5.5E-04	2.4E-04	8.7E-04
7.9E-06	7.0E-04	5.6E-04	1.4E-04
7.9E-06	7.2E-04	3.6E-04	7.9E-04
7.9E-06	7.7E-04	3.0E-04	7.3E-04
8.0E-06	7.7E-04	2.0E-04	5.9E-04
8.0E-06	7.0E-04	1.3E-04	2.9E-04
8.0E-06	8.1E-04	1.3E-04	3.6E-04
8.1E-06	5.7E-04	2.7E-04	2.1E-04
8.1E-06	9.2E-04	1.3E-04	8.3E-04
8.1E-06	7.8E-04	3.8E-04	7.2E-05
8.2E-06	6.5E-04	2.5E-04	7.1E-04
8.2E-06	2.1E-04	6.8E-05	2.9E-04
8.2E-06	1.7E-04	2.1E-04	4.5E-04
8.3E-06	2.4E-04	1.4E-04	3.9E-04
8.3E-06	9.7E-04	4.4E-04	-7.2E-05
8.3E-06	9.8E-04	3.5E-04	-5.5E-04
8.4E-06	4.5E-04	5.3E-04	-3.5E-04
8.4E-06	8.9E-04	2.7E-04	-3.5E-04
8.4E-06	8.4E-04	7.1E-05	-4.4E-05
8.5E-06	9.5E-04	8.1E-05	3.3E-04
8.5E-06	9.5E-04	1.2E-04	1.4E-04
8.5E-06	4.7E-04	3.0E-04	4.5E-04
8.5E-06	1.2E-03	2.6E-04	3.5E-04
8.6E-06	1.3E-03	6.1E-04	3.8E-04
8.6E-06	8.5E-04	4.1E-04	9.4E-06
8.6E-06	5.7E-04	2.9E-04	-1.1E-04
8.7E-06	3.2E-04	7.2E-04	1.2E-04
8.7E-06	6.2E-04	2.9E-04	-2.8E-04
8.7E-06	9.2E-04	2.1E-04	2.6E-04
8.8E-06	1.1E-03	3.8E-04	2.0E-04
8.8E-06	7.8E-04	7.0E-05	7.0E-04
8.8E-06	6.3E-04	5.2E-05	-2.9E-04
8.9E-06	1.0E-03	1.7E-04	5.6E-04
8.9E-06	1.4E-03	2.6E-04	-2.1E-04
8.9E-06	7.3E-04	4.1E-05	-6.3E-05
9.0E-06	7.7E-04	4.3E-04	1.6E-04
9.0E-06	8.8E-04	4.0E-04	2.0E-04
9.0E-06	1.1E-03	8.7E-05	-3.5E-04
9.1E-06	9.5E-04	2.3E-04	7.2E-04
9.1E-06	5.9E-04	2.1E-04	4.5E-04
9.1E-06	8.0E-04	3.9E-04	2.8E-04
9.2E-06	1.1E-03	-1.9E-04	4.4E-04
9.2E-06	5.9E-04	-1.1E-04	4.5E-04

9.2E-06	5.8E-04	1.8E-04	4.8E-04
9.3E-06	1.1E-03	8.0E-05	1.9E-04
9.3E-06	1.2E-03	-1.7E-04	5.7E-04
9.3E-06	1.0E-03	4.3E-04	6.1E-04
9.3E-06	7.4E-04	1.6E-04	4.2E-04
9.4E-06	6.3E-04	8.8E-05	6.0E-04
9.4E-06	5.8E-04	2.7E-04	2.5E-04
9.4E-06	8.5E-04	3.7E-04	4.2E-04
9.5E-06	2.6E-04	3.0E-04	2.3E-04
9.5E-06	8.2E-04	2.8E-04	1.5E-04
9.5E-06	1.1E-03	5.0E-04	3.5E-04
9.6E-06	5.8E-04	4.2E-04	-1.0E-04
9.6E-06	9.5E-04	9.3E-05	-3.6E-04
9.6E-06	1.4E-03	2.5E-04	3.5E-05
9.7E-06	1.1E-03	3.1E-04	-3.8E-04
9.7E-06	1.2E-03	5.2E-05	9.2E-05
9.7E-06	8.9E-04	3.1E-05	4.0E-04
9.8E-06	1.2E-03	3.4E-04	-1.1E-04
9.8E-06	6.2E-04	3.4E-04	-5.6E-05
9.8E-06	1.1E-03	2.1E-04	-3.4E-04
9.9E-06	5.9E-04	2.1E-04	-2.3E-04
9.9E-06	1.0E-03	3.5E-04	-4.6E-05
9.9E-06	1.0E-03	5.4E-04	4.3E-04
1.0E-05	1.1E-03	1.8E-04	2.2E-04
1.0E-05	1.1E-03	-3.0E-06	-6.9E-05
1.0E-05	5.9E-04	4.4E-04	2.4E-04
1.0E-05	1.2E-03	5.6E-05	-1.3E-04
1.0E-05	8.8E-04	4.9E-04	-1.1E-04
1.0E-05	1.1E-03	1.8E-04	5.7E-04
1.0E-05	1.0E-03	2.3E-04	1.5E-04
1.0E-05	1.1E-03	1.5E-04	3.0E-04
1.0E-05	7.4E-04	4.2E-04	1.5E-04
1.0E-05	9.5E-04	2.2E-04	1.1E-04
1.0E-05	1.0E-03	2.4E-04	-5.0E-05
1.0E-05	9.3E-04	-2.5E-05	1.7E-05
1.0E-05	1.2E-03	1.9E-04	2.0E-04
1.0E-05	1.2E-03	1.8E-04	2.8E-04
1.0E-05	1.3E-03	5.1E-04	2.5E-04
1.0E-05	4.3E-04	-8.5E-05	-2.1E-04
1.1E-05	5.4E-04	3.3E-04	-2.5E-04
1.1E-05	1.2E-03	2.7E-05	-3.8E-04
1.1E-05	9.7E-04	4.1E-04	-9.3E-05
1.1E-05	4.0E-04	2.3E-04	-4.0E-04
1.1E-05	6.4E-04	1.2E-04	-3.2E-04
1.1E-05	3.8E-04	1.1E-04	-5.6E-05
1.1E-05	1.1E-03	-1.6E-05	3.6E-04
1.1E-05	1.1E-03	1.5E-04	-1.9E-04
1.1E-05	1.4E-03	1.4E-04	-1.1E-04
1.1E-05	4.5E-04	3.9E-04	9.4E-05
1.1E-05	1.1E-03	6.1E-04	2.2E-04
1.1E-05	4.7E-04	-4.9E-04	8.2E-05
1.1E-05	3.9E-05	2.1E-04	-2.2E-07
1.1E-05	1.0E-03	-1.3E-04	-9.6E-05
1.1E-05	8.3E-04	3.0E-04	-1.7E-04

1.1E-05	1.0E-03	-2.7E-04	5.4E-05
1.1E-05	7.9E-04	-1.1E-04	4.9E-05
1.1E-05	9.4E-04	1.1E-04	-1.5E-04
1.1E-05	5.5E-04	6.1E-04	-2.1E-04
1.1E-05	1.1E-03	2.0E-04	2.4E-04
1.1E-05	4.6E-04	2.9E-04	-5.7E-05
1.1E-05	1.2E-03	2.5E-04	-3.5E-04
1.1E-05	1.0E-03	8.2E-05	-3.1E-04
1.1E-05	1.0E-03	-2.2E-04	-1.9E-04
1.1E-05	1.1E-03	6.6E-04	-3.6E-05
1.1E-05	7.3E-04	-1.7E-04	2.5E-05
1.1E-05	5.5E-04	8.7E-05	-3.6E-04
1.1E-05	1.2E-03	2.7E-04	3.0E-05
1.1E-05	6.5E-04	3.4E-04	-4.3E-04
1.1E-05	1.0E-03	8.8E-04	-4.0E-04
1.1E-05	9.5E-04	4.6E-04	1.6E-04
1.2E-05	8.0E-04	2.1E-04	-2.0E-04
1.2E-05	-4.1E-05	3.6E-04	-2.9E-04
1.2E-05	9.4E-04	1.4E-04	-4.9E-04
1.2E-05	8.8E-04	-5.1E-04	-3.5E-04
1.2E-05	7.0E-04	2.1E-04	-5.1E-04
1.2E-05	9.4E-05	3.3E-04	1.1E-04
1.2E-05	6.7E-04	-7.1E-04	-2.7E-04
1.2E-05	2.8E-04	4.3E-04	-1.8E-04
1.2E-05	3.8E-04	1.7E-04	3.2E-04
1.2E-05	1.2E-03	3.0E-04	6.7E-05
1.2E-05	9.7E-04	-2.0E-05	-1.6E-04
1.2E-05	2.5E-04	1.1E-04	4.3E-05
1.2E-05	8.4E-04	1.0E-04	3.3E-04
1.2E-05	3.0E-04	4.1E-06	6.7E-04
1.2E-05	7.5E-04	-8.9E-05	-3.1E-04
1.2E-05	8.6E-04	5.5E-04	-8.0E-04
1.2E-05	8.4E-04	3.2E-04	-1.7E-04
1.2E-05	-3.5E-04	-2.3E-04	-2.9E-04
1.2E-05	5.2E-04	5.2E-04	-6.2E-04
1.2E-05	8.6E-04	-6.9E-04	-4.6E-04
1.2E-05	7.8E-04	3.0E-04	1.5E-04
1.2E-05	9.8E-04	4.7E-04	-1.3E-04
1.2E-05	5.8E-04	-2.8E-04	2.1E-04
1.2E-05	7.9E-04	3.0E-05	1.4E-04
1.2E-05	9.2E-04	2.1E-04	-2.9E-04
1.2E-05	4.6E-04	-1.5E-04	-8.0E-05
1.2E-05	5.7E-04	-3.6E-04	7.6E-05
1.2E-05	8.5E-04	8.4E-04	1.7E-04
1.2E-05	1.2E-03	3.9E-05	-1.2E-04
1.2E-05	9.9E-04	2.9E-04	1.6E-05
1.2E-05	5.0E-04	-3.4E-04	2.1E-04
1.3E-05	9.7E-04	4.1E-04	-6.3E-04
1.3E-05	8.4E-04	-2.3E-04	-1.8E-04
1.3E-05	1.0E-03	-6.2E-04	1.8E-05
1.3E-05	8.2E-04	-4.5E-04	2.1E-04
1.3E-05	1.1E-03	5.1E-04	6.3E-05
1.3E-05	8.0E-04	-2.9E-04	3.1E-04
1.3E-05	6.6E-04	2.9E-04	6.4E-04

1.3E-05	2.6E-05	1.1E-04	-7.3E-04
1.3E-05	1.2E-03	-1.0E-04	4.7E-04
1.3E-05	1.0E-03	4.3E-04	2.0E-04
1.3E-05	7.6E-04	-1.3E-04	-4.9E-05
1.3E-05	3.3E-04	2.3E-04	1.5E-04
1.3E-05	1.2E-03	8.2E-04	1.8E-04
1.3E-05	4.4E-04	-6.7E-05	-2.7E-04
1.3E-05	1.1E-03	9.9E-05	-2.5E-04
1.3E-05	1.4E-04	-3.0E-04	-3.5E-04
1.3E-05	9.9E-04	-7.3E-04	5.7E-04
1.3E-05	3.5E-04	4.9E-04	6.6E-05
1.3E-05	6.8E-04	-3.7E-04	-1.5E-04
1.3E-05	8.7E-04	1.4E-05	4.1E-05
1.3E-05	1.2E-03	1.1E-04	-7.0E-05
1.3E-05	5.9E-04	-6.0E-04	-1.3E-04
1.3E-05	9.2E-04	-1.7E-04	-2.3E-04
1.3E-05	5.7E-04	-6.7E-05	3.1E-05
1.3E-05	8.6E-04	7.7E-04	-1.7E-04
1.3E-05	9.8E-04	-2.2E-04	8.4E-05
1.3E-05	6.9E-04	4.5E-04	-5.1E-05
1.3E-05	4.3E-04	2.5E-04	-1.2E-04
1.3E-05	4.2E-04	2.8E-04	3.5E-04
1.3E-05	7.9E-04	5.5E-04	-2.5E-04
1.3E-05	4.4E-04	2.5E-04	1.3E-04
1.3E-05	7.2E-04	2.5E-04	1.7E-04
1.4E-05	1.0E-03	5.9E-04	2.4E-04
1.4E-05	8.8E-04	-2.1E-05	2.1E-04
1.4E-05	6.0E-04	1.3E-05	2.4E-04
1.4E-05	7.8E-04	-2.3E-04	1.6E-04
1.4E-05	4.5E-04	3.2E-05	2.0E-04
1.4E-05	8.9E-04	4.9E-04	2.3E-04
1.4E-05	7.6E-04	-6.1E-04	2.8E-04
1.4E-05	4.6E-04	2.5E-05	9.4E-04
1.4E-05	1.9E-04	-4.7E-04	-6.1E-05
1.4E-05	9.5E-04	-2.1E-04	4.3E-04
1.4E-05	9.5E-04	4.7E-04	1.5E-05
1.4E-05	4.3E-04	4.4E-05	1.1E-04
1.4E-05	7.9E-04	4.0E-04	2.1E-04
1.4E-05	6.3E-04	-4.3E-04	-1.1E-04
1.4E-05	4.6E-04	-2.0E-05	7.1E-04
1.4E-05	3.5E-04	-3.3E-04	6.3E-04
1.4E-05	5.7E-04	-3.6E-05	3.8E-04
1.4E-05	8.0E-04	-1.4E-04	3.9E-05
1.4E-05	4.5E-04	-1.4E-04	3.6E-04
1.4E-05	-3.1E-04	-2.7E-04	-2.4E-04
1.4E-05	5.2E-04	-2.6E-04	2.7E-04
1.4E-05	6.7E-04	-5.9E-04	-2.8E-04
1.4E-05	5.7E-04	2.1E-04	-2.8E-04
1.4E-05	8.2E-04	-5.0E-04	-8.3E-05
1.4E-05	1.1E-03	-1.9E-04	4.9E-05
1.4E-05	1.4E-04	2.7E-04	3.3E-04
1.4E-05	4.3E-05	3.9E-04	8.8E-05
1.4E-05	1.3E-03	-3.5E-04	3.5E-04
1.4E-05	6.0E-04	-1.7E-04	-2.9E-04

1.4E-05	5.7E-04	-1.7E-04	-1.7E-04
1.4E-05	7.6E-04	-3.7E-04	-2.4E-04
1.5E-05	9.4E-04	1.8E-04	2.8E-04
1.5E-05	6.9E-04	-2.9E-04	-3.1E-04
1.5E-05	3.7E-04	2.1E-04	2.4E-04
1.5E-05	1.0E-03	5.0E-06	3.6E-04
1.5E-05	1.4E-04	3.3E-04	1.6E-04
1.5E-05	4.3E-04	-2.9E-04	2.8E-05
1.5E-05	9.9E-04	-5.3E-05	5.5E-04
1.5E-05	1.4E-04	1.3E-04	-4.2E-05
1.5E-05	1.0E-03	5.1E-04	-1.3E-04
1.5E-05	6.9E-04	-2.3E-04	-1.2E-04
1.5E-05	6.2E-04	-3.0E-04	3.8E-04
1.5E-05	5.3E-04	9.2E-05	2.4E-04
1.5E-05	5.9E-04	5.7E-04	7.1E-04
1.5E-05	5.4E-04	6.8E-04	3.2E-04
1.5E-05	1.4E-04	-8.7E-05	2.6E-04
1.5E-05	1.0E-03	-8.3E-05	5.6E-04
1.5E-05	4.6E-04	2.7E-04	2.3E-04
